# Asymmetric remote C-H borylation of internal alkenes via alkene isomerization

**DOI:** 10.1038/s41467-018-06240-y

**Published:** 2018-09-26

**Authors:** Xu Chen, Zhaoyang Cheng, Jun Guo, Zhan Lu

**Affiliations:** Department of Chemistry, Zhejiang University, 310058 Hangzhou, China

## Abstract

Recent years have witnessed the growing interest in the remote functionalization of alkenes for it offers a strategy to activate the challenging C–H bonds distant from the initiation point via alkene isomerization/functionalization. However, the catalytic enantioselective isomerization/functionalization with one single transition metal catalyst remains rare. Here we report a highly regio- and enantioselective cobalt-catalyzed remote C–H bond borylation of internal alkenes via sequential alkene isomerization/hydroboration. A chiral ligand featured twisted pincer, anionic, and non-rigid characters is designed and used for this transformation. This methodology, which is operationally simple using low catalyst loading without additional activator, shows excellent enantioselectivity and can be used to convert various internal alkenes with regio- and stereoisomers to valuable chiral secondary organoboronates with good functional group tolerance.

## Abstract (web summary)

Sequential alkene isomerization/functionalization enables enantioselective transformations of remote C–H bonds. Here, the authors report a chiral cobalt catalytic system for the highly enantioselective, remote C–H borylation of internal alkenes via an isomerization/hydroboration sequence.

## Introduction

**Fig. 1:**
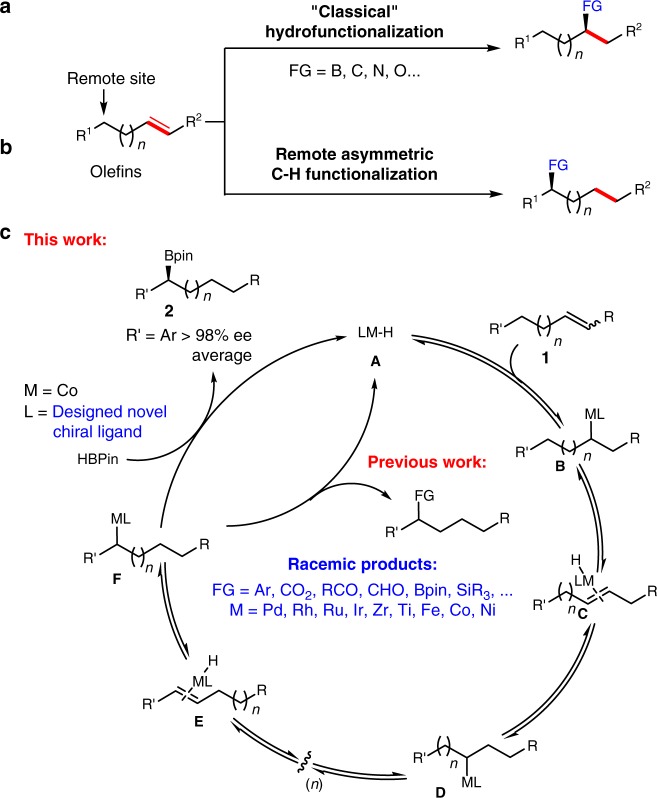
Remote functionalization of alkenes via sequential alkene isomerization/functionalization. **a** Asymmetric hydrofunctionalization of alkenes. **b** The concept of asymmetric remote functionalization of alkenes via isomerization. **c** A general pathway for the remote functionalization of alkenes via isomerization

Alkenes containing multiple unactivated C(*sp*^3^)-H bond are readily available and abundant feedstock starting materials. Catalytic asymmetric strategies based on alkenes for construction of chiral organic molecules are commonly used. Asymmetric hydrofunctionalization of unactivated alkenes via metal-hydride species has been well established for efficient construction of chiral carbon centers (Fig. 1a)^1–3^. Among these transformations, alkene isomerization is considered to be a side-reaction to produce regio- and stereoisomers. However, this sequential alkene isomerization/functionalization offers an opportunity for the direct and enantioselective transformation of remote unactivated C(*sp*^3^)-H bonds to carbon–carbon or carbon–heteroatom bonds, which is fundamentally important and challenging for highly efficient organic synthesis (Fig. 1b)^4–15^. A general pathway for the remote functionalization of alkenes via isomerization is illustrated in Fig. 1c. Alkene **1** undergoes coordination and insertion into metal hydrogen bond to form alkyl metal species **B** that initiates alkene isomerization. Species **B** goes through *β*-H elimination to generate species **C**. After a serial of chain-walking process, a more stable alkyl metal species **F**, such as terminal alkyl or benzylic metal species, is formed as a terminal intermediate. Finally, species **F** could be trapped with a variety of reagents to afford products and regenerate catalyst, which offered a favorable thermodynamic driving force.

Recent years have witnessed the important progress in the field of catalytic alkene isomerization/functionalization with various coupling reagents^5,16^, such as ArX^17–19^, CO_2_^20,21^, CO/H_2_^22^, HBpin^23–26^, R_3_SiH^27–29^, and so on^11,30,31^, to afford the corresponding coupling products. Additionally, the catalytic asymmetric sequential functionalization/isomerization of alkenes terminated by oxygen-motif has been demonstrated by Sigman^32,33^. However, the catalytic enantioselective isomerization/functionalization with one single transition metal catalyst is restricted to only few examples^5,16^. Nishimura and coworkers^34^ used Iridium catalyst to achieve alkene isomerization terminated by ether group and the following asymmetric hydroarylation. The development of asymmetric alkene isomerization/functionalization processes using single catalyst system is highly desirable.

Chiral organoboronates are of significant utility in asymmetric synthesis for constructing a wide range of other functional groups through C-B bond transformation in a stereospecific fashion^35,36^. To date, several strategies^37^, such as stereospecific organoboronate homologation^38,39^, borylation of benzylic electrophiles^40,41^, asymmetric hydrogenation of alkenylboronic esters^42,43^, and asymmetric hydroboration of alkenes^44–53^, have been developed for construction of chiral secondary organoboronates. However, asymmetric hydroboration of a mixture of alkenes isomers to deliver chiral products has not been previously reported. Our group is continuously investigating asymmetric iron- or cobalt-catalyzed hydrofunctionalization of alkenes based on the ligand design^54–59^. Recently, we have developed a cobalt-catalyzed asymmetric sequential hydroboration/hydrogenation of internal alkynes, affording a series of chiral secondary organoboronates^56^. The control experiment demonstrated that cobalt-catalyzed asymmetric hydroboration of internal alkenes afforded secondary organoboronates with poor enantioselectivity. It would be ideal to develop a highly enantioselective cobalt-catalyzed hydroboration of internal alkenes.

Here, we report a cobalt-catalyzed asymmetric remote C–H borylation of internal alkenes via isomerization/hydroboration using a imidazoline phenyl picoliamide (ImPPA) ligand with high enantioselectivity (>97% ee in most cases) (Fig. 1c).

## Results

### Reaction optimization

**Fig. 2:**
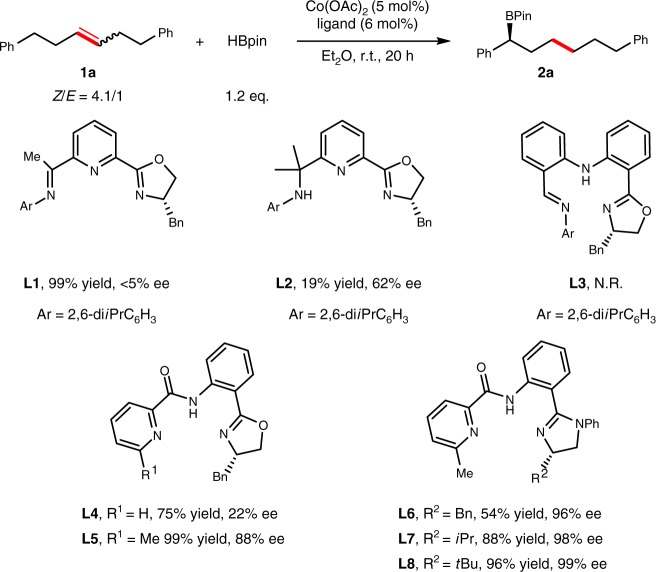
Ligands screen for asymmetric isomerization/hydroboration. Reaction conditions: **1a** (1 mmol), HBpin (1.2 mmol), Co(OAc)_2_ (5.0 mol%), ligand (6.0 mol%), Et_2_O (1 M), r.t., 20 h

The simple internal alkene **1a** was chosen as a model substrate (see Fig. 2 and Supplementary Tables 1, 2). When chiral OIP ligand **L1** was used^54^, the cobalt-catalyzed isomerization/hydroboration reaction of **1a** with HBpin was carried out to deliver **2a** in 99% yield, however, with less than 5% ee. The use of amino-derivated ligand **L2**^60^ or iminoaniline-derivated ligand **L3**^61^ led to a significant drop-off in yield, whereas the use of **L2** improved the enantioselectivity to 62% ee. Using a well-defined ligand **L4**^62^, the remote borylative reaction could afford **2a** in 75% yield with 22% ee. Encouragingly, when ligand **L5** containing a methyl group at 6-position on pyridine was used, the enantioselectivity was dramatically promoted to 88% ee. Replacement of substituents on pyridine or oxazoline improved the enantioselectivity to 93% ee (see Supplementary Table 1). To our delight, the use of a more electron-rich phenyl-protected imidazoline (**L6**) instead of oxazoline led to a significant improvement in enantioselectivity (96% ee). Assessment of various imidazolines showed that **L8** with a more bulky *tert*-butyl group was the most effective ligand to afford **2a** in 96% yield with 99% ee. Catalyst loading could be further decreased to 2.5 mol% to afford **2a** in 90% yield with 98% ee. The standard conditions are identified as 1 mmol of alkene, 1.2 mmol of HBpin, 2.5 mol% of Co(OAc)_2_, 3 mol% of **L8** in 1.0 mL of Et_2_O for 20 h.

### Substrate scope

With the optimized conditions in hand, we explored the scope of the olefins (Table 1). The cyclohexyl alkene could participate to deliver the isomerization/hydroboration product **2b** in 85% yield with 96% ee. The electron-donating and electron-withdrawing groups on phenyl ring were tolerated to afford **2c**–**2l** in 31–84% yields with 97–>99% ee. Particularly, *ortho*-substituted alkene **1k** could also participate in the reaction to afford **2k** in 31% yield with excellent enantioselectivity (>99% ee). The alkenes containing polycyclic ring and heterocycle, such as 2-naphthyl (**1** **m**), 1-naphthyl (**1n**), 3-pyridyl (**1o**) and 3-benzo[*b*]thiophenyl (**1p**), could be converted to the corresponding products **2m–2p** in 64–87% yields with 97–>99% ee. Alkenes containing various functional groups, such as acetal (**1r**), ester (**1s**), amide (**1t**), tertiary alcohol (**1u**), and protected amine (**1w**) could be tolerated to afford corresponding boronic esters in 46–71% yields with 86–99% ee. Particularly, alkene **1v** with primary alcohol could also participate in the reaction and afford the product in 43% yield with 99% ee. The reaction of terminal alkene **1×** with HBpin afforded a mixture of the desired product **2×** with 99% ee and terminal borylated product with a *b*/*l* ratio of 1/1. The alkene with a linear undecyl group could be reacted to afford **2y** in 65% yield and 98% ee with a *b*/*l* ratio of 4/1. The reactions of alkenes containing terminal *tert*-butyl (**1z**) and cyclohexyl group (**1aa**) gave the benzylic borylated products with high regio- and enantioselectivities, even walking over eight carbon–carbon bonds (**2ab**, 58% yield, 99% ee). Remarkably, the trisubstituted alkene **1ac** and **1ad** could also participate in the transformation to afford the corresponding products in 74% yield with 99% ee and 55% yield with >99% ee, respectively. Alkene **1ae** could also be transformed to **2ae** in 67% yield with 11/1 *rr* and 98% ee. Alkene **1af** containing a disubstituted and a trisubstituted olefin has also been tested in the reaction which afforded a mixture of (*E*)-**2af** and (*Z*)-**2af** both in 94% ee. Vitamin E-derivated olefin **1ag** was smoothly converted to the corresponding product **2ag** in 60% yield with 98% de. Due to the unstability, some products (**2m**, **2s**–**2w**, **2ae–2ag**) were obtained after being directly oxidized to the corresponding alcohols. The absolute configuration was verified by comparison of the optical rotation of **2q** with previously reported data and the other products were then assigned by analogy^63^.

**Table 1:**
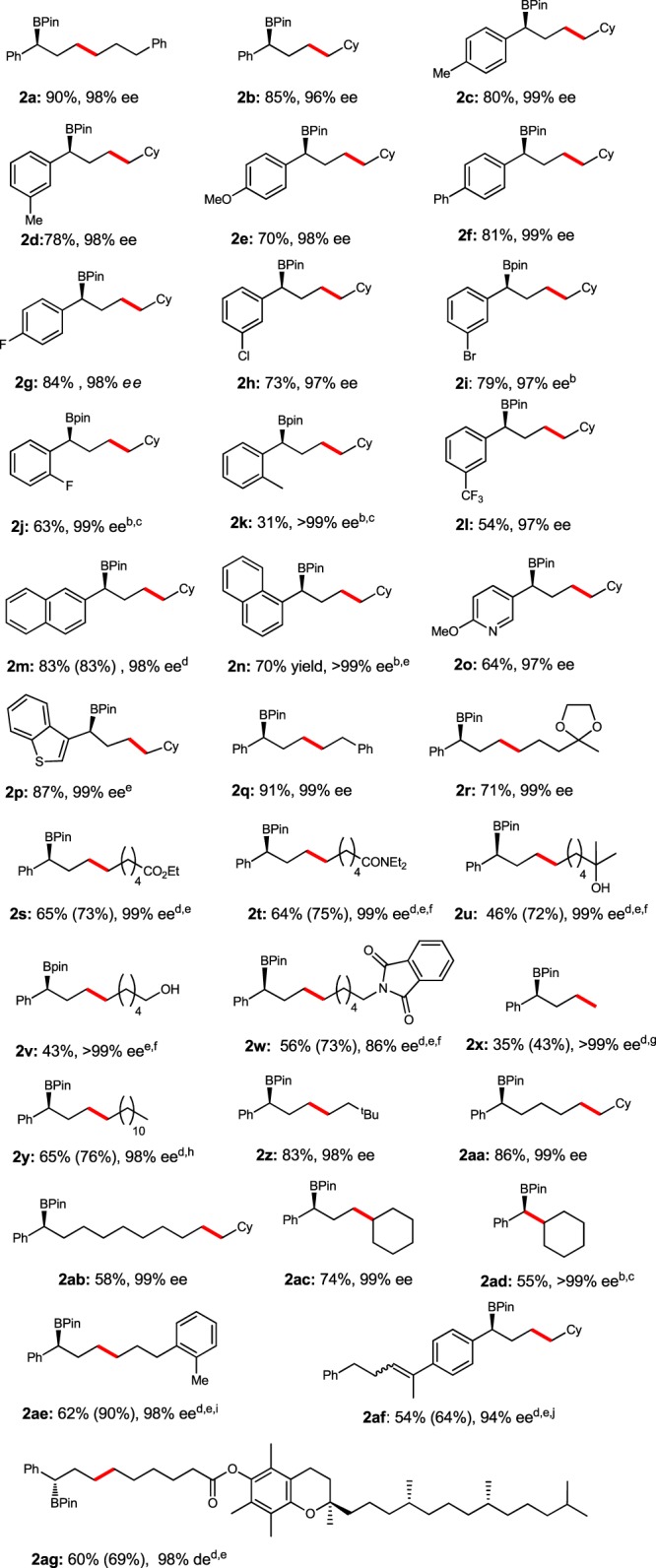
Substrate scope of enantioselective isomerization/hydroboration of alkenes

^a^Standard conditions: **1** (1 mmol), HBpin (1.2 mmol), Co(OAc)_2_ (2.5 mol%), **L8** (3 mol%), Et_2_O (1 M), r.t., 20 h

^b^48 h

^c^Co(OAc)_2_ (10 mol%), **L8** (12 mol%)

^d^NMR yield for boronic ester in the parentheses; isolated yield for corresponding alcohol outside the parentheses

^e^Co(OAc)_2_ (5 mol%), **L8** (6 mol%)

^f^HBpin (2.0 eq.)

^g^1/1 *rr*

^h^4/1 *rr*

^i^11/1 *rr*

^j^*Z*/*E* = 1.2/1

### Gram-scale reaction and synthesis of bioactive molecule

**Fig. 3:**
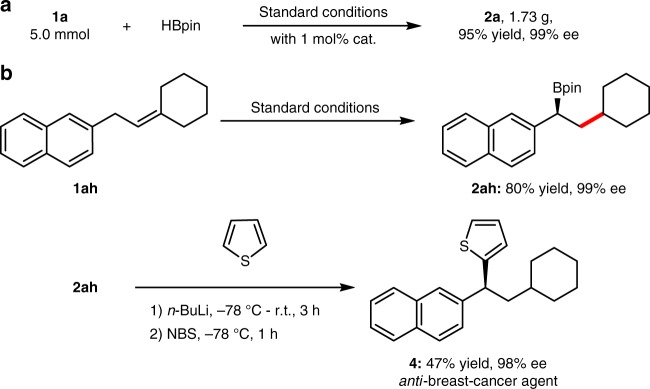
Applications. **a** Gram-scale reaction. **b** Synthesis of *anti*-breast-cancer agent **4**

Notably, the preparation of **2a** could be scaled up in 95% yield with 99% ee using 1 mol% of Co(OAc)_2_ and 1.2 mol% of ligand (Fig. 3a). Alkene (**1ah**) could be transformed smoothly to afford **2ah** in 80% yield with 99% ee which could easily undergo C–C bond cross-coupling in a stereospecific manner^64^ to synthesize *anti*-breast-cancer agent **4** (Fig. 3b).

### Mechanistic study and other applications

**Fig. 4:**
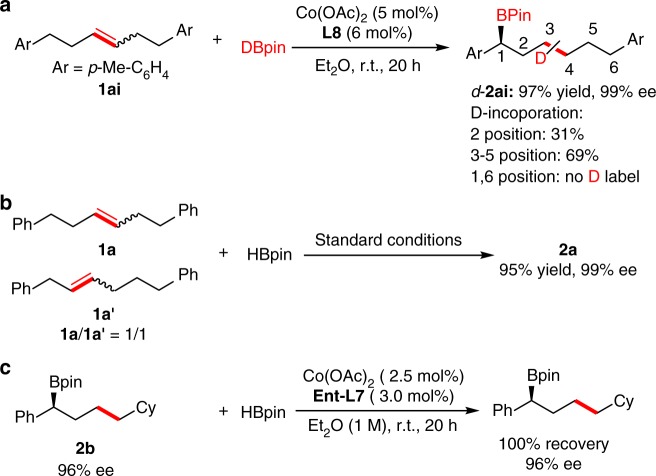
Isotope labeling and control experiments. **a** Deuterium labeling experiment. **b** Utilization of a mixture of geometrical and positional alkene isomers. **c** The reaction of product **2b** with HBpin using enantiomer of **L7**

Cobalt-catalyzed deuterium labeling experiment was also conducted with DBpin (Fig. 4a). Stirring a mixture of **1ai** and DBpin in the presence of 5 mol% of Co(OAc)_2_ and 6 mol% of **L8** furnished *d*-**2ai** with 31% d-incorporation in 2-position. Detectable amounts of deuterium were also located in the interior (3–5) positions with 69% d-incorporation in total. No deuterium was detected at benzylic positions showed that species **E** underwent olefin reinsertion step to prefer to form more stable benzylic cobalt species **F** rather than non-benzylic alkyl cobalt species. It should be note that a mixture of **1a** and **1a**′ (1/1) could be transformed smoothly to a single product **2a** in 95% yield with 99% ee under the standard conditions (Fig. 4b), which demonstrated the power of this catalytic system to utilize a mixture of geometrical and positional alkene isomers. Alkenes (*E*)-**1aj** and (*Z*)-**1aj** were subjected to the reaction system and the result shows that the stereochemistry of the starting olefin has no impact on the kinetics of the reaction (see Supplementary Fig. 246). The reaction of product **2b** under the standard conditions using ligand **Ent-L7** (the enantiomer of **L7**) was conducted and no reaction occurred. The ee value of boronate **2b** did not change, which indicated that the formation of the carbon boron bond was irreversible (Fig. 4c).

### Time course study

**Fig. 5:**
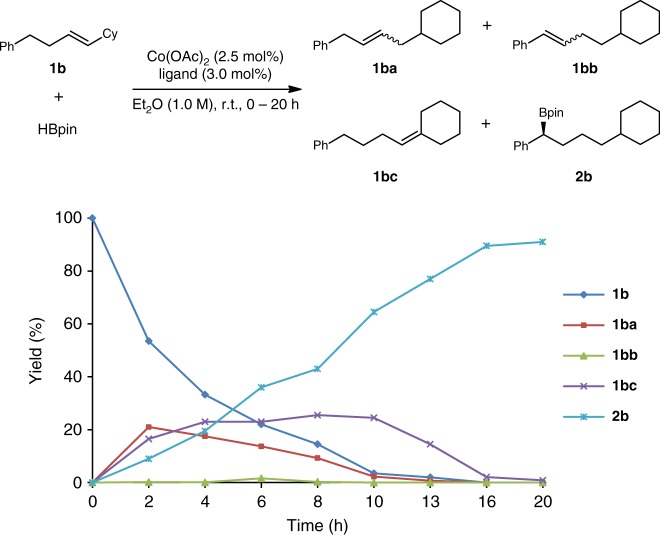
The time course study of **1b**. Reaction conditions: **1b** (0.5 mmol), HBpin (0.6 mmol), Co(OAc)_2_ (2.5 mol%), **L8** (3.0 mol%), Et_2_O (1 M), r.t., 0–20 h

The time course experiment (detail see Supplementary Table 5) of **1b** was conducted (Fig. 5). The observation of alkenes **1ba**, **1bb**, and **1bc** in the process showed that the internal alkene **1b** underwent a double bond walking process to both the benzylic position and cyclohexyl position. Only a small amount of the benzylic alkene **1bb** (<5%) was observed during the whole process, which demonstrated that the benzylic alkyl cobalt species **F** might undergo a rapid *σ*-bond metathesis with HBpin to afford the chiral organoboronic ester **2b**.

## Discussion

In summary, a highly regio- and enantioselective cobalt- catalyzed remote C–H bond borylation of internal alkenes via sequential alkene isomerization/hydroboration is developed. A chiral ImPPA ligand featured twisted pincer, anionic, and non-rigid characters is designed and used. This protocol is operationally simple and additional activator-free. The commonly useless mixture of internal alkenes is used for highly efficient and selective construction of valuable chiral secondary organoboronates with good functional group tolerance. The development of asymmetric transformations based on ligand design will be continuously carried out at our laboratory.

## Methods

### Materials

For NMR spectra of compounds in this manuscript, see Supplementary Figs. 1–204. For HPLC spectra of compounds in this manuscript, see Supplementary Figs. 205–245. For the optimization of reaction conditions and control experiments of alkene **1a**, see Supplementary Tables 1, 2. For the experimental procedures and analytic data of compounds synthesized, see Supplementary Methods.

### General procedure for remote C–H borylation of internal alkenes

To a 25 mL flame-dried Schlenk flask cooled under nitrogen, Co(OAc)_2_ (0.025 mmol), **L8** (0.03 mmol), Et_2_O (1 mL) were added. The mixture was stirred at room temperature for 5 min. Then, alkene (1.0 mmol), HBpin (180 μL, 1.2 mmol) were added in sequence and stirred at room temperature for 20 h. The resulting solution was filtered by a short pad of silica gel and washed by ether (10 mL × 2). The combined filtrate was concentrated and purified by flash column chromatography using PE/EtOAc = 20/1 as the eluent to afford the corresponding product.

## Electronic supplementary material

Supplementary Information

## Data Availability

The authors declare that the data supporting the findings of this study are available within the paper and its Supplementary Information file. The X-ray crystallographic coordinates for structures of **(L**_**8**_**-H)·PdOAc** has been deposited at the Cambridge Crystallographic Data Centre (CCDC) under deposition nos. CCDC 1588226. The data can be obtained free of charge from the Cambridge Crystallographic Data Centre via http://www.ccdc.cam.ac.uk/data_request/cif. The experimental procedures and characterization of all new compounds are provided in the Supplementary Information.
