# Supplementary material for: Asymmetric remote C-H borylation of internal alkenes via alkene isomerization
Source: Nat Commun. 2018 Sep 26;9:3939. doi: 10.1038/s41467-018-06240-y (PMC6158179; doi:10.1038/s41467-018-06240-y)
Supplement: Supplementary file 1 — Supplementary Information [file 41467_2018_6240_MOESM1_ESM.pdf]

Asymmetric Remote C-H Borylation of Internal Alkenes via Alkene Isomerization

Supporting Information

*Xu Chen, Zhaoyang Cheng, Jun Guo, Zhan Lu**

Department of Chemistry, Zhejiang University, Hangzhou 310058, China

Supplementary Figures

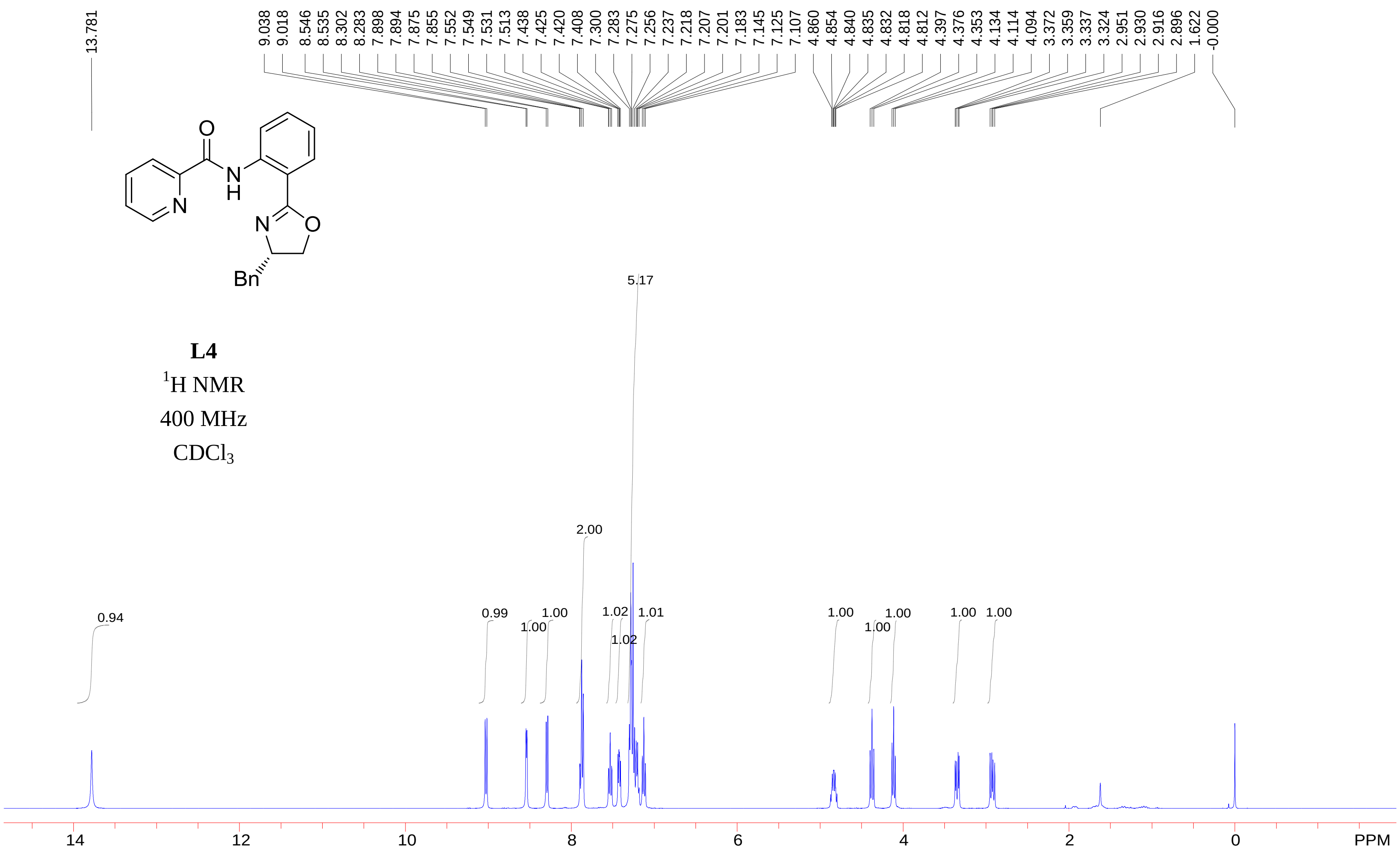

Supplementary Figure 1. ^{1}H NMR spectrum for **L4**

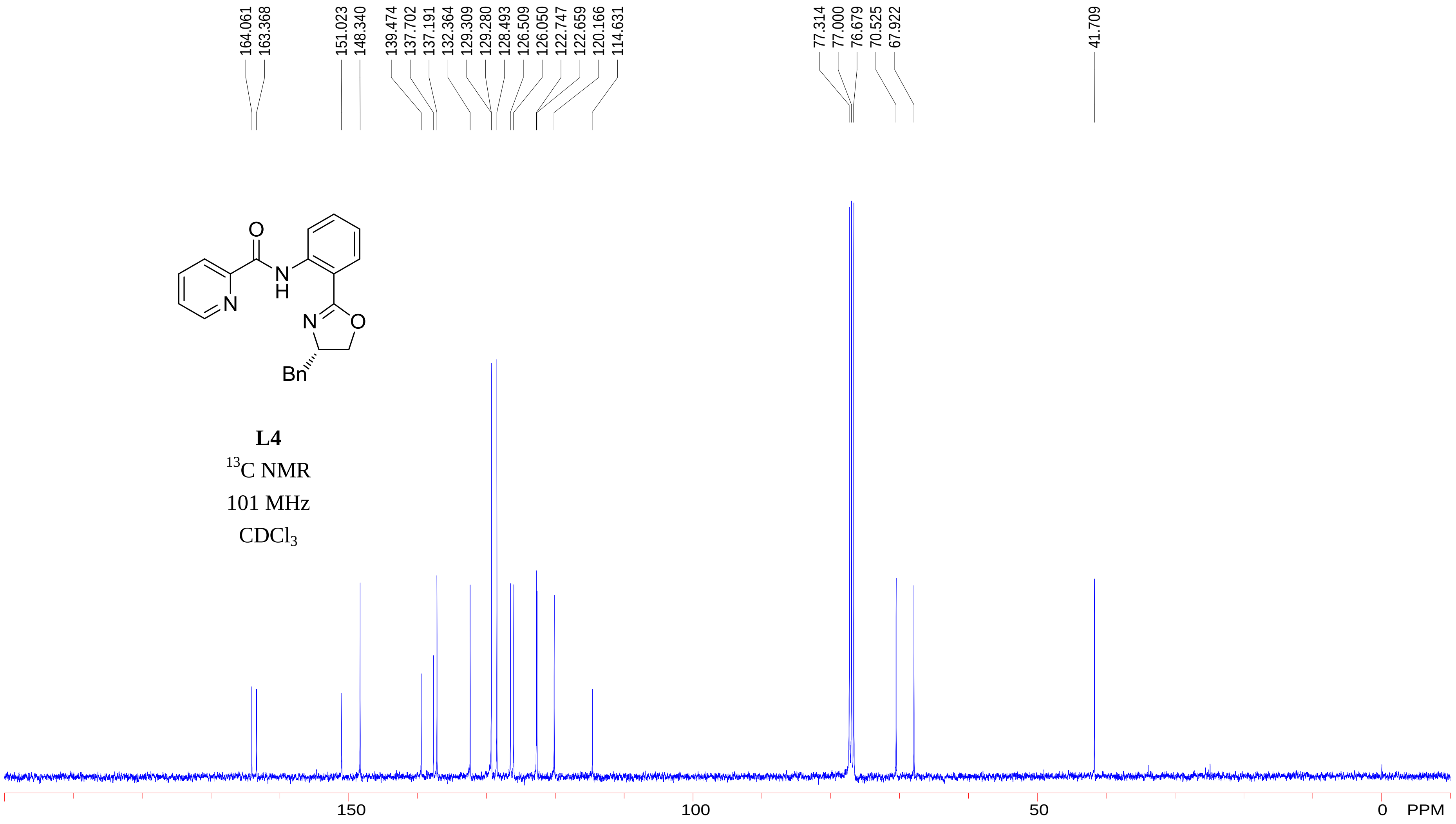

Supplementary Figure 2. ^{13}C NMR spectrum for **L4**

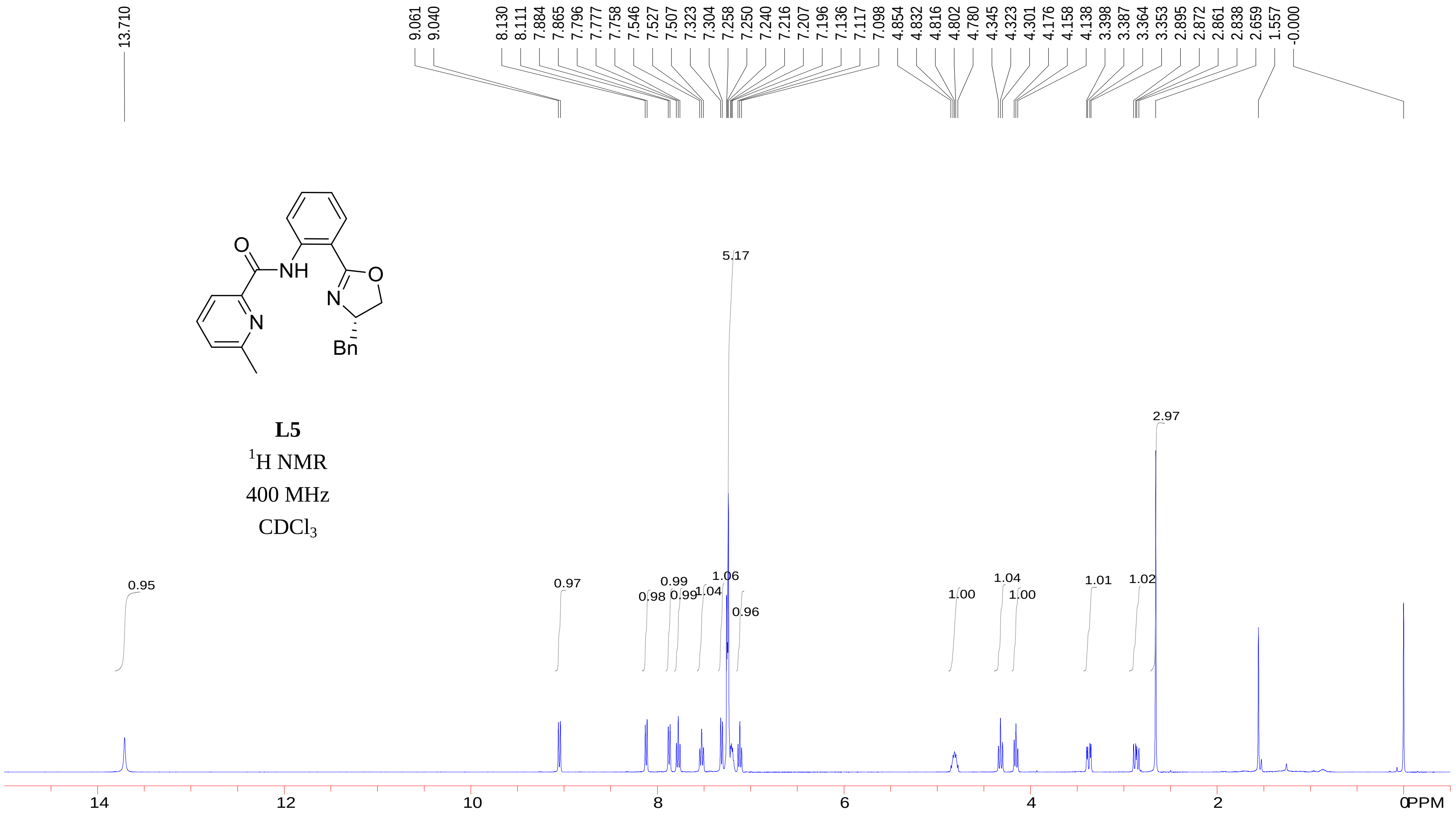

Supplementary Figure 3. ^{1}H NMR spectrum for **L5**

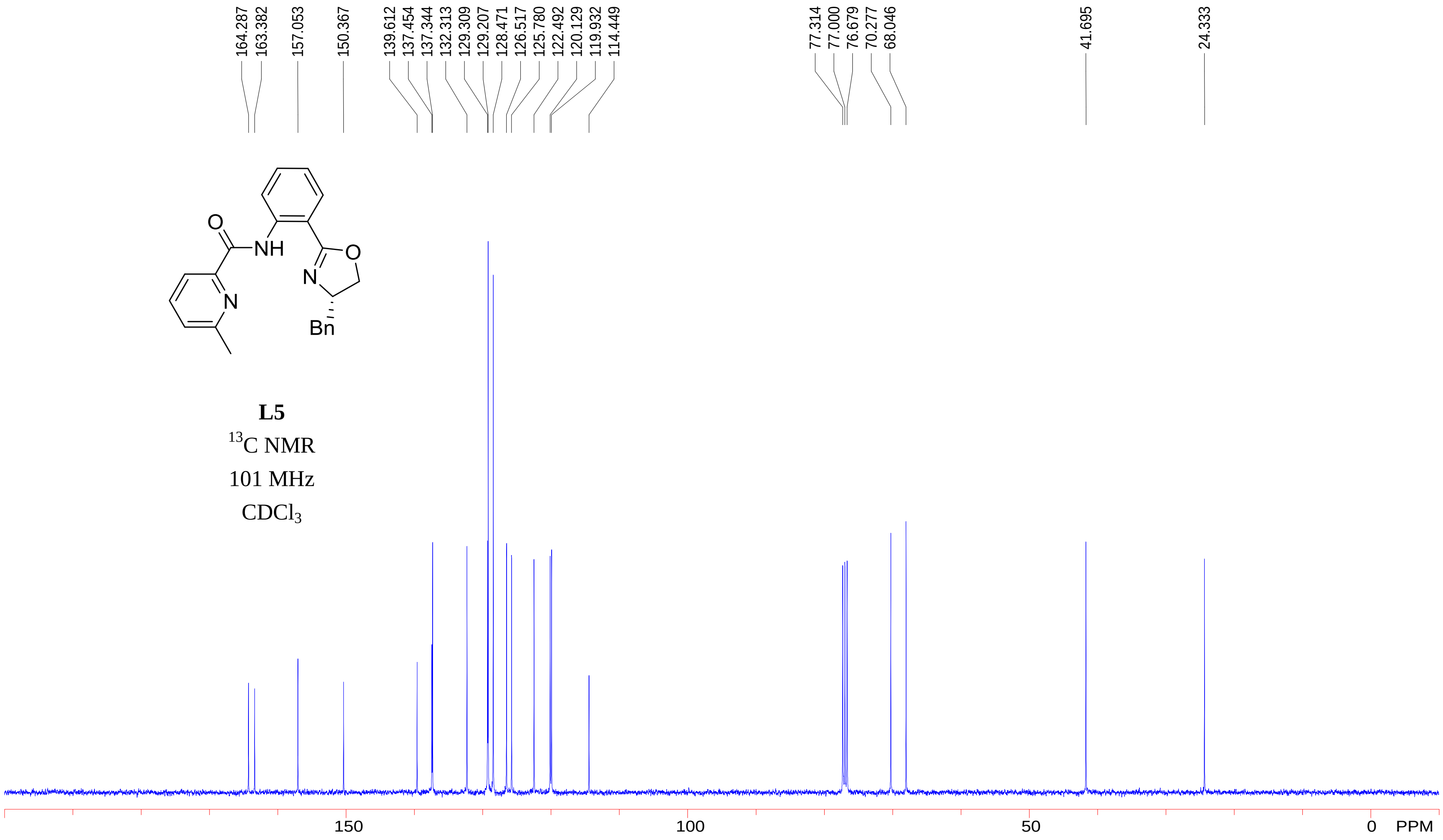

Supplementary Figure 4. ^{13}C NMR spectrum for **L5**

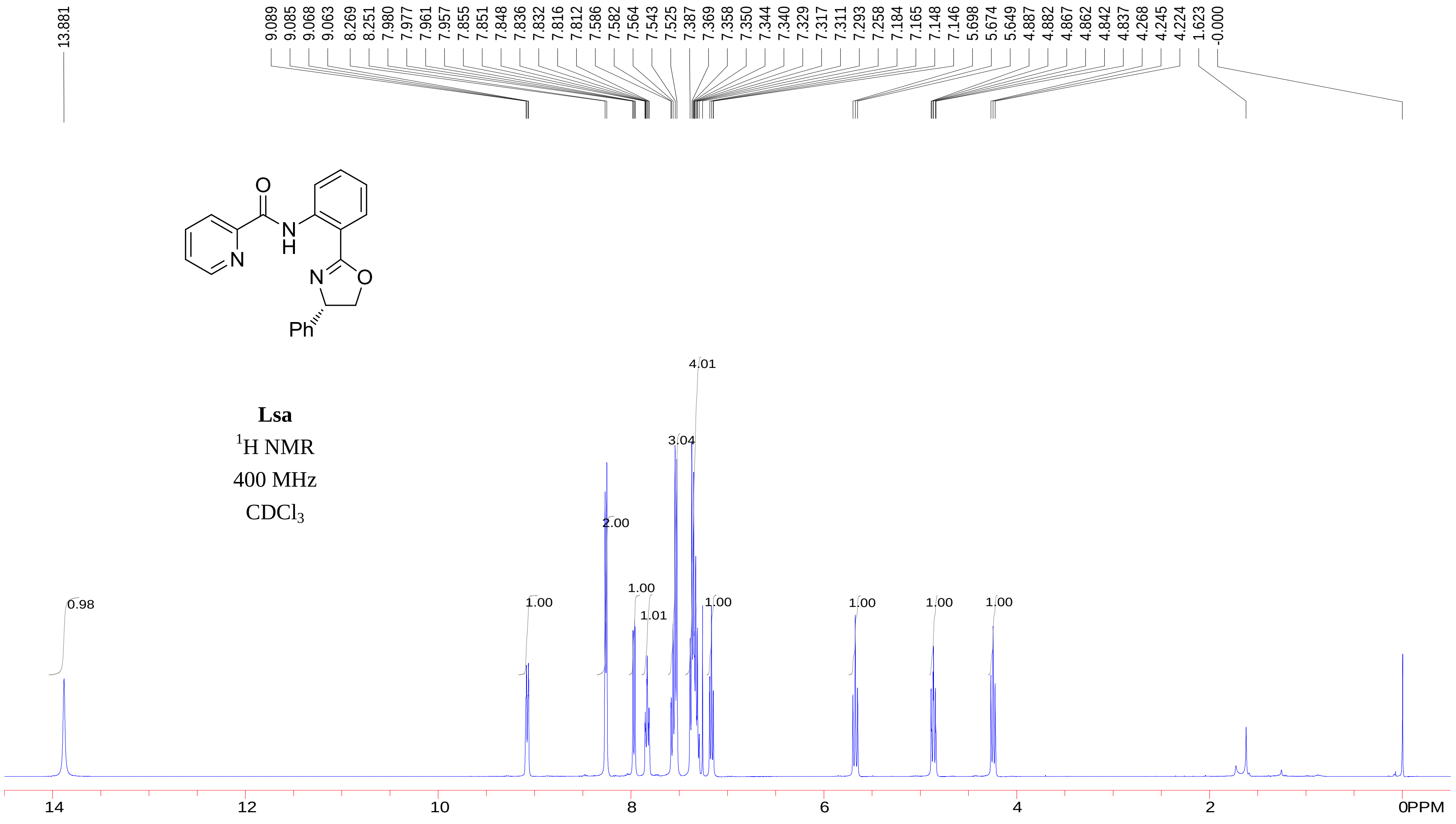

Supplementary Figure 5. ^{1}H NMR spectrum for **Lsa**

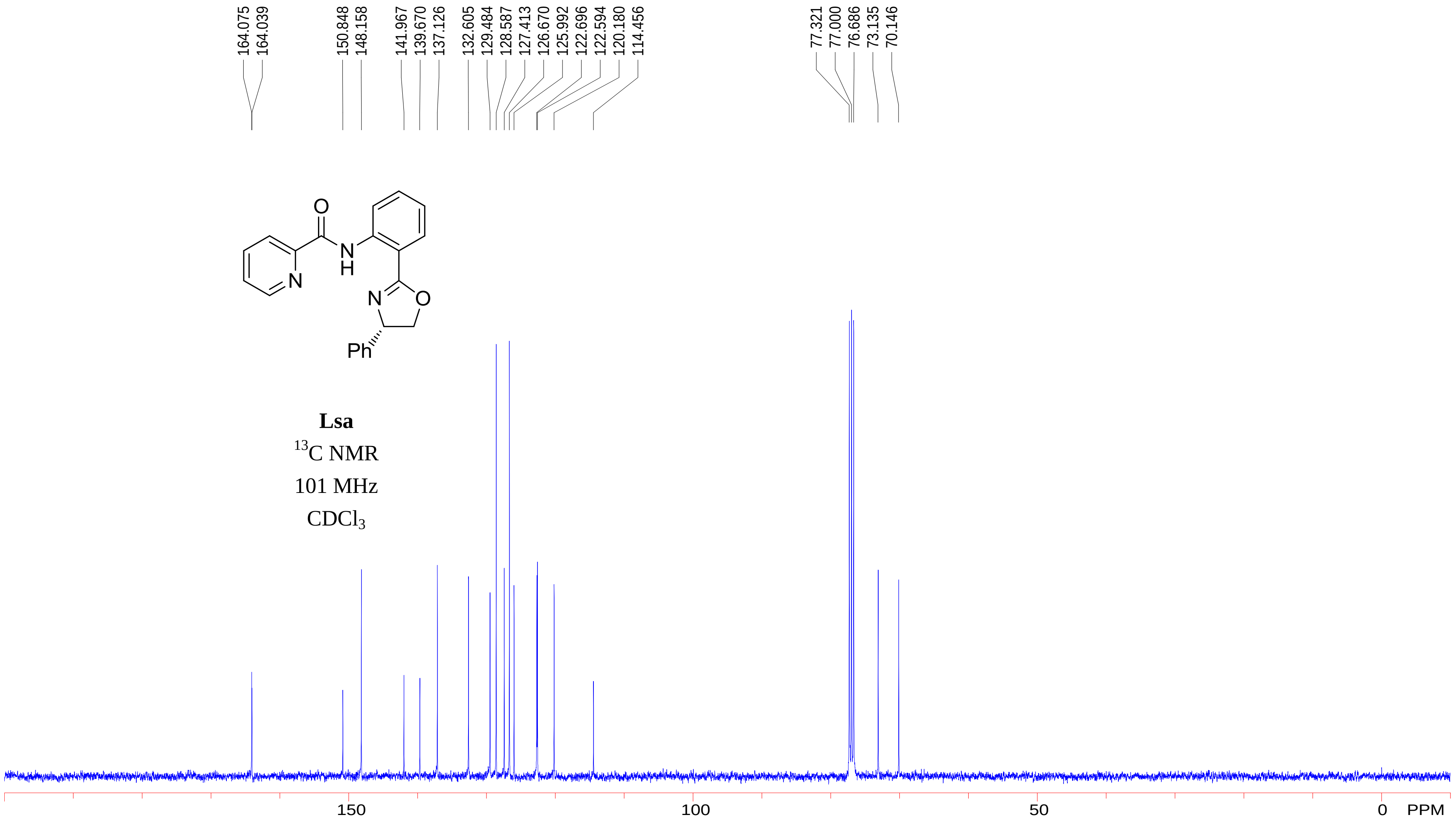

Supplementary Figure 6. ^{13}C NMR spectrum for **Lsa**

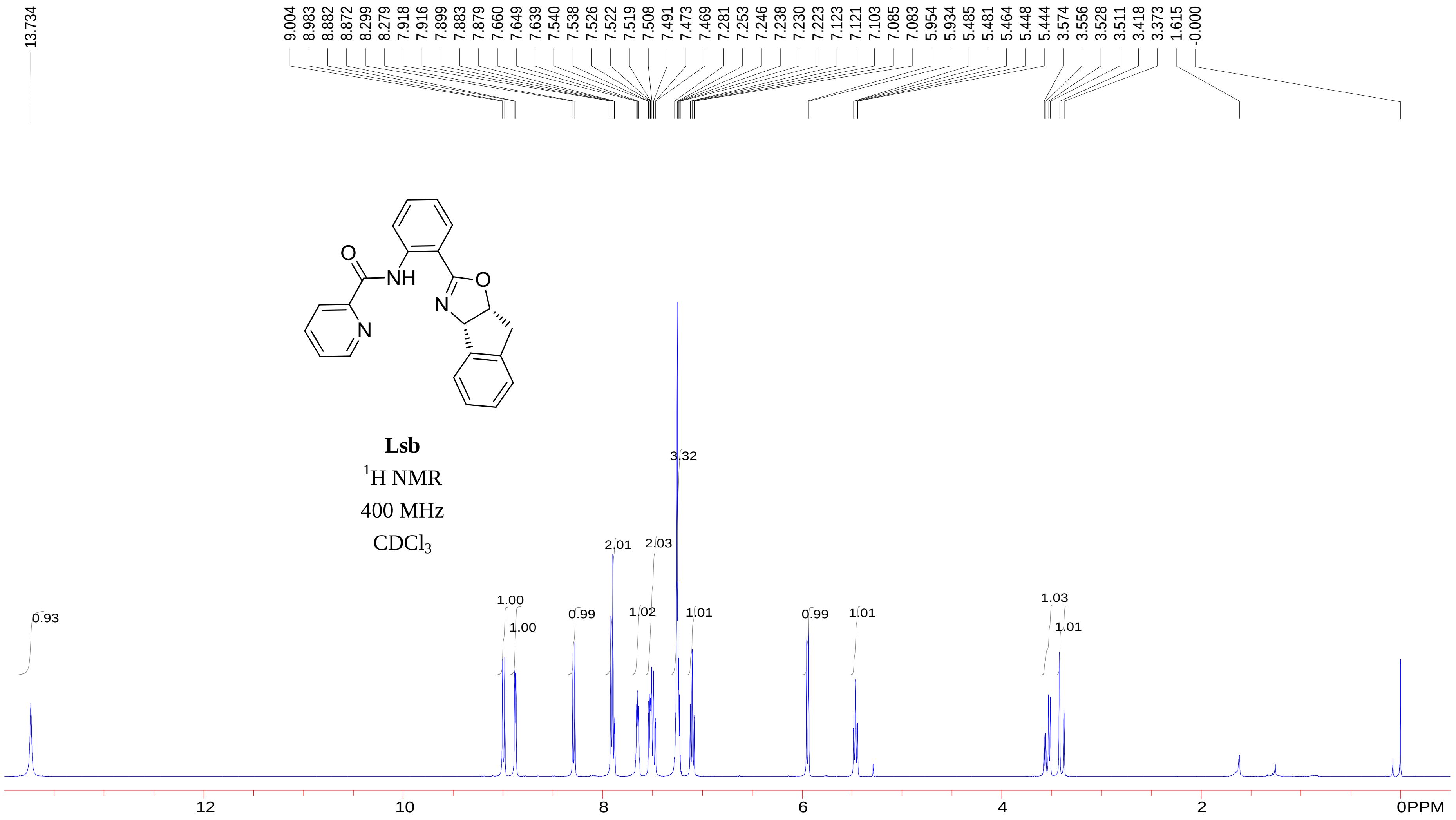

Supplementary Figure 7. ^{1}H NMR spectrum for **Lsb**

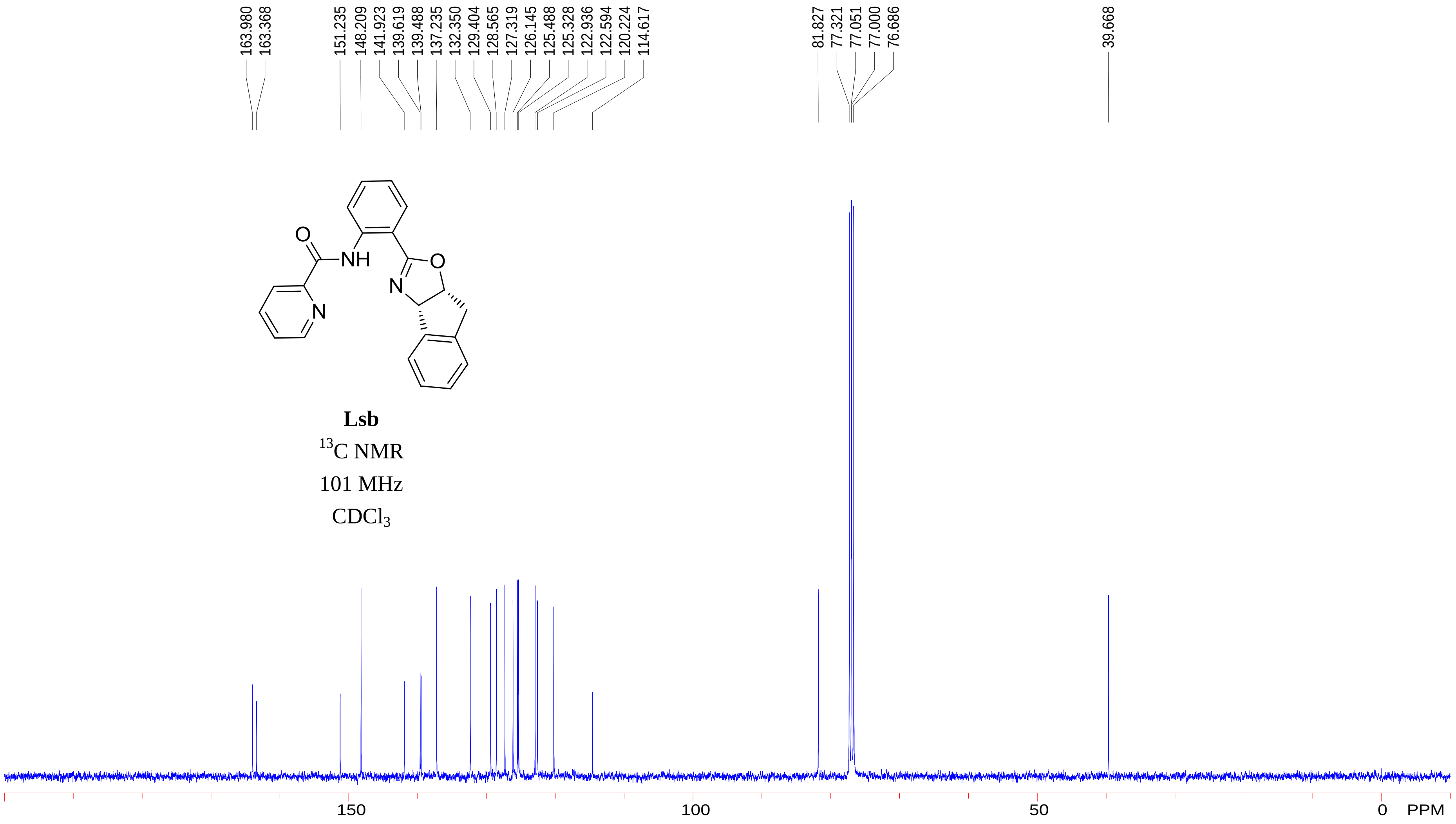

Supplementary Figure 8. ^{13}C NMR spectrum for **Lsb**

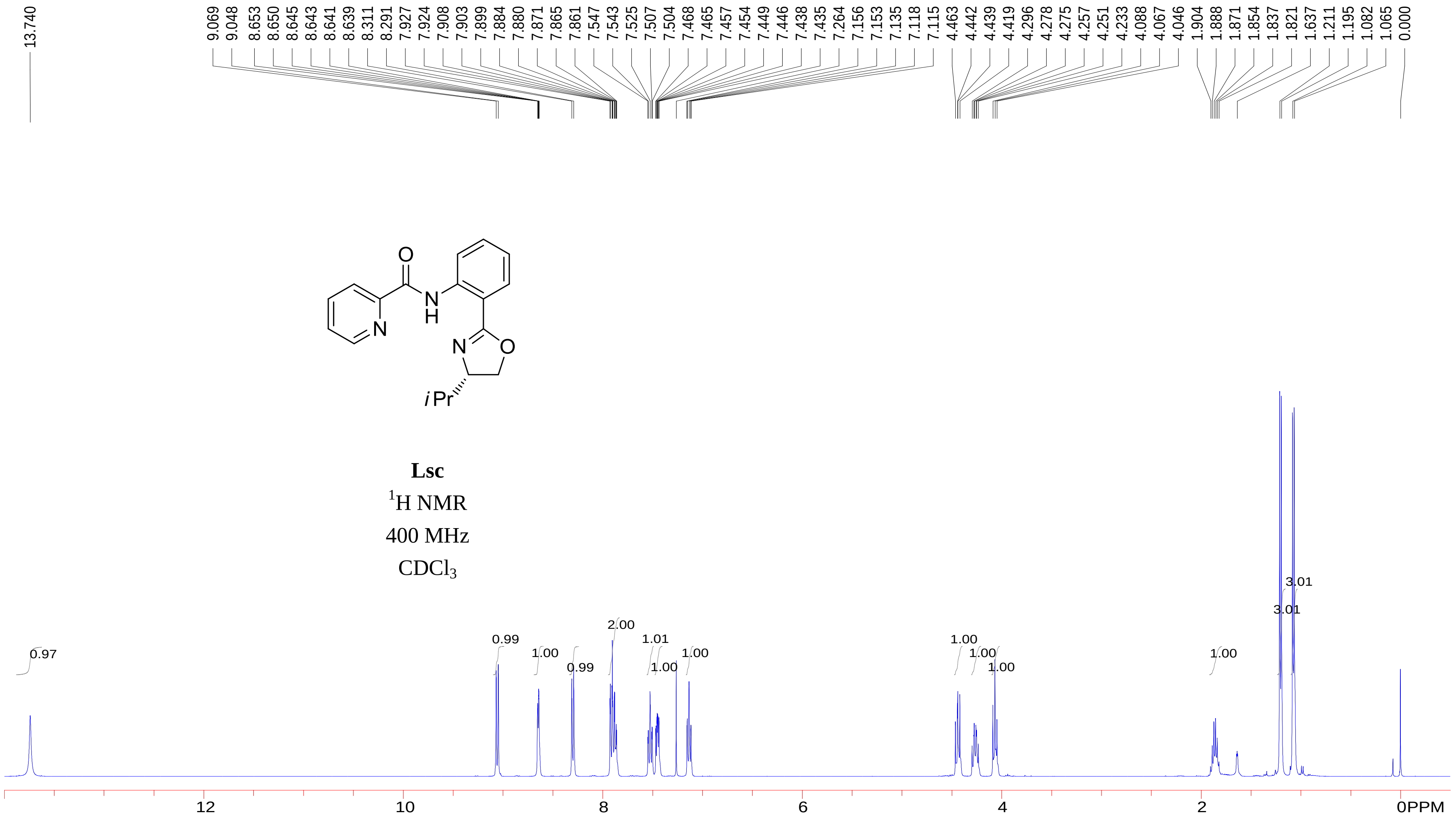

Supplementary Figure 9. ^{1}H NMR spectrum for **Lsc**

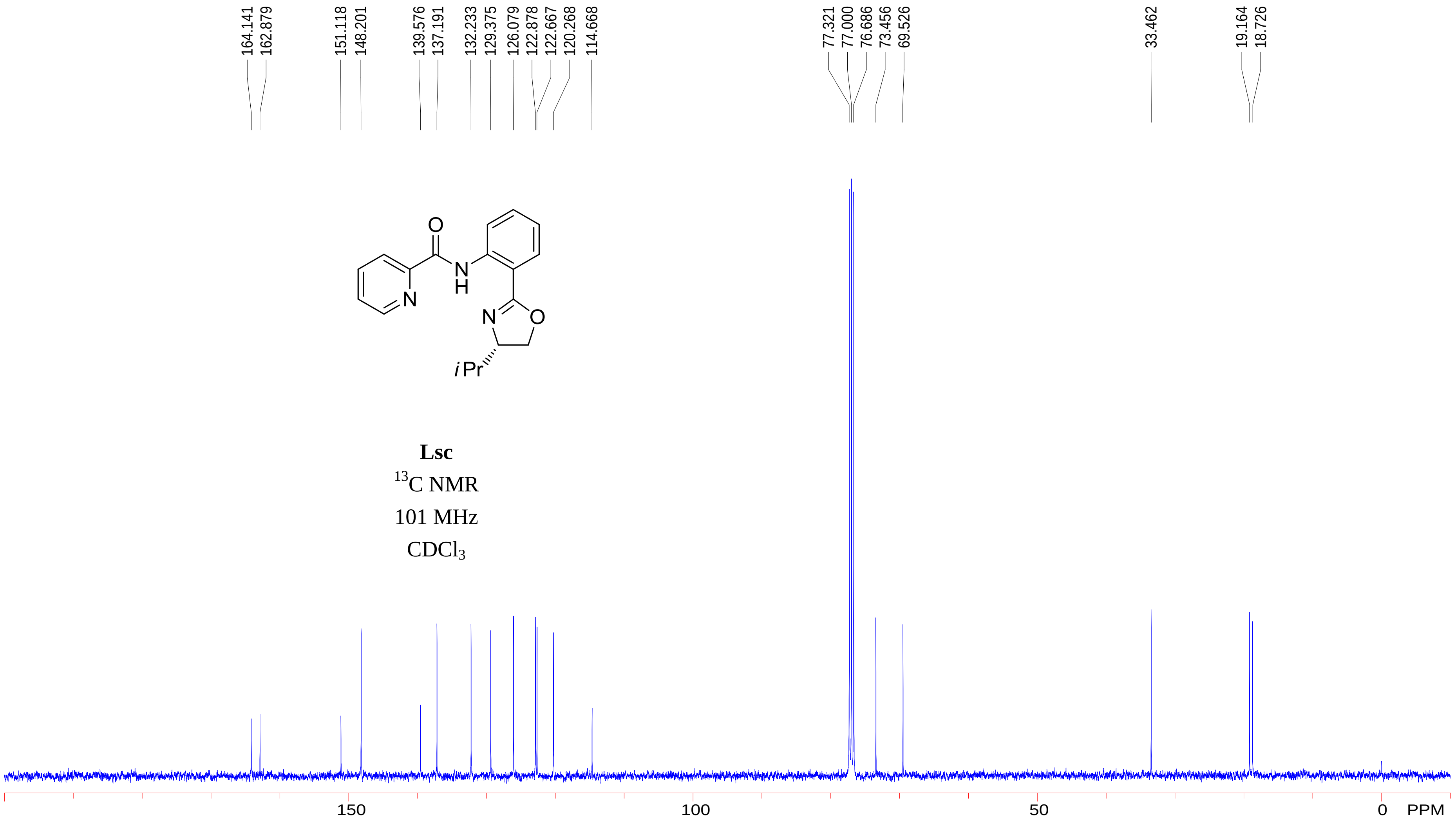

Supplementary Figure 10. ^{13}C NMR spectrum for **Lsc**

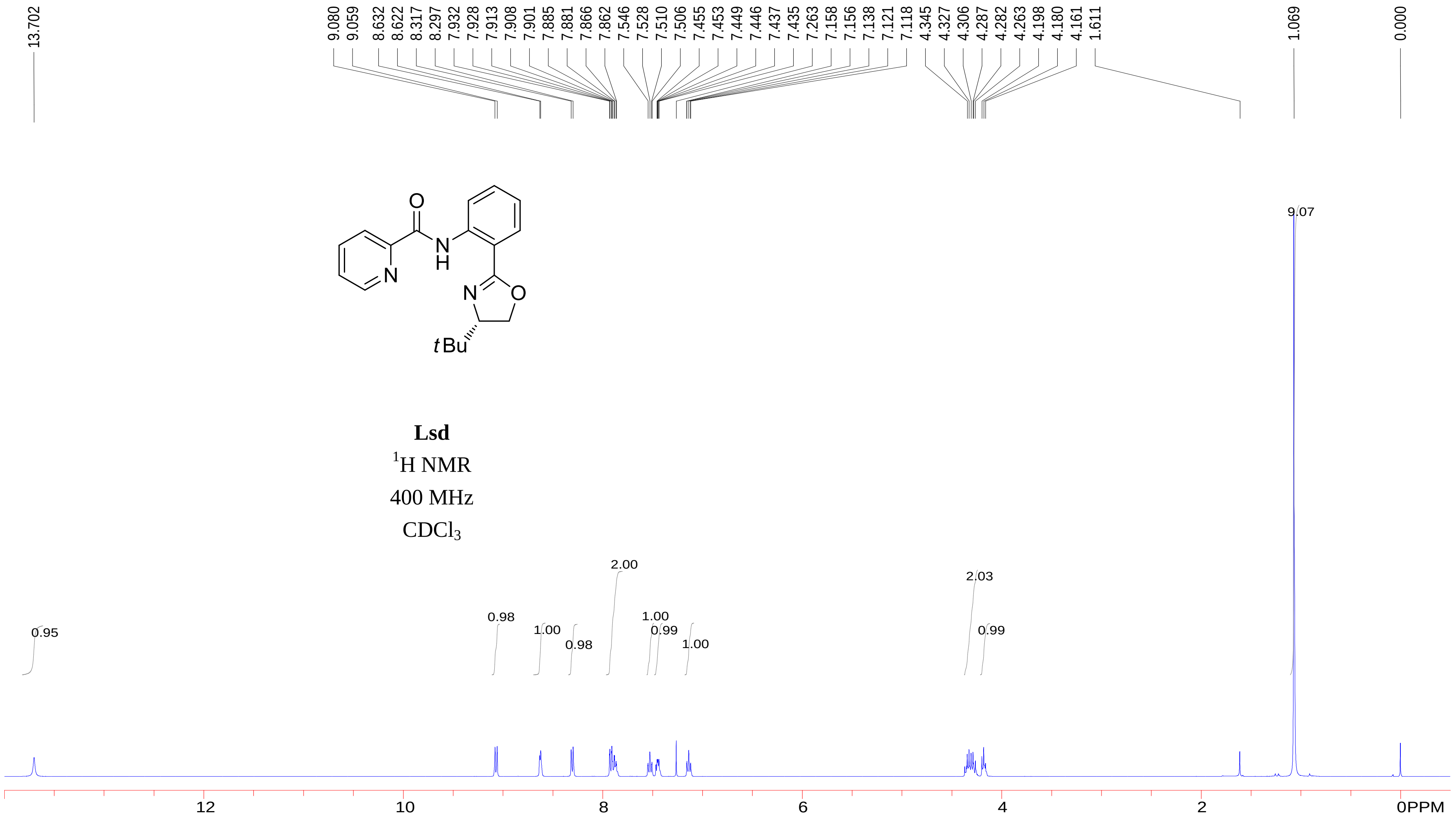

Supplementary Figure 11. ^{1}H NMR spectrum for **Lsd**

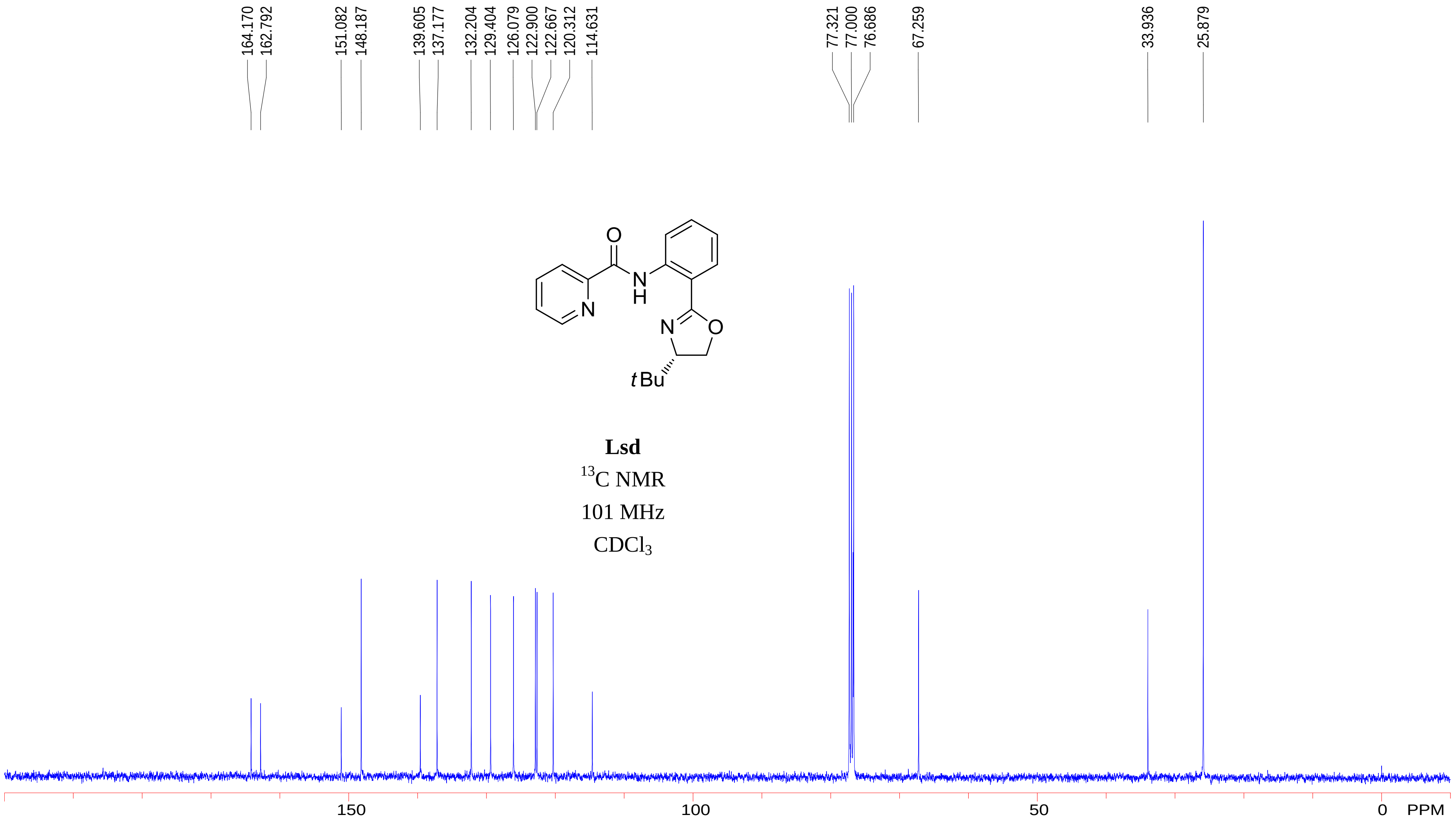

Supplementary Figure 12. ^{13}C NMR spectrum for **Lsd**

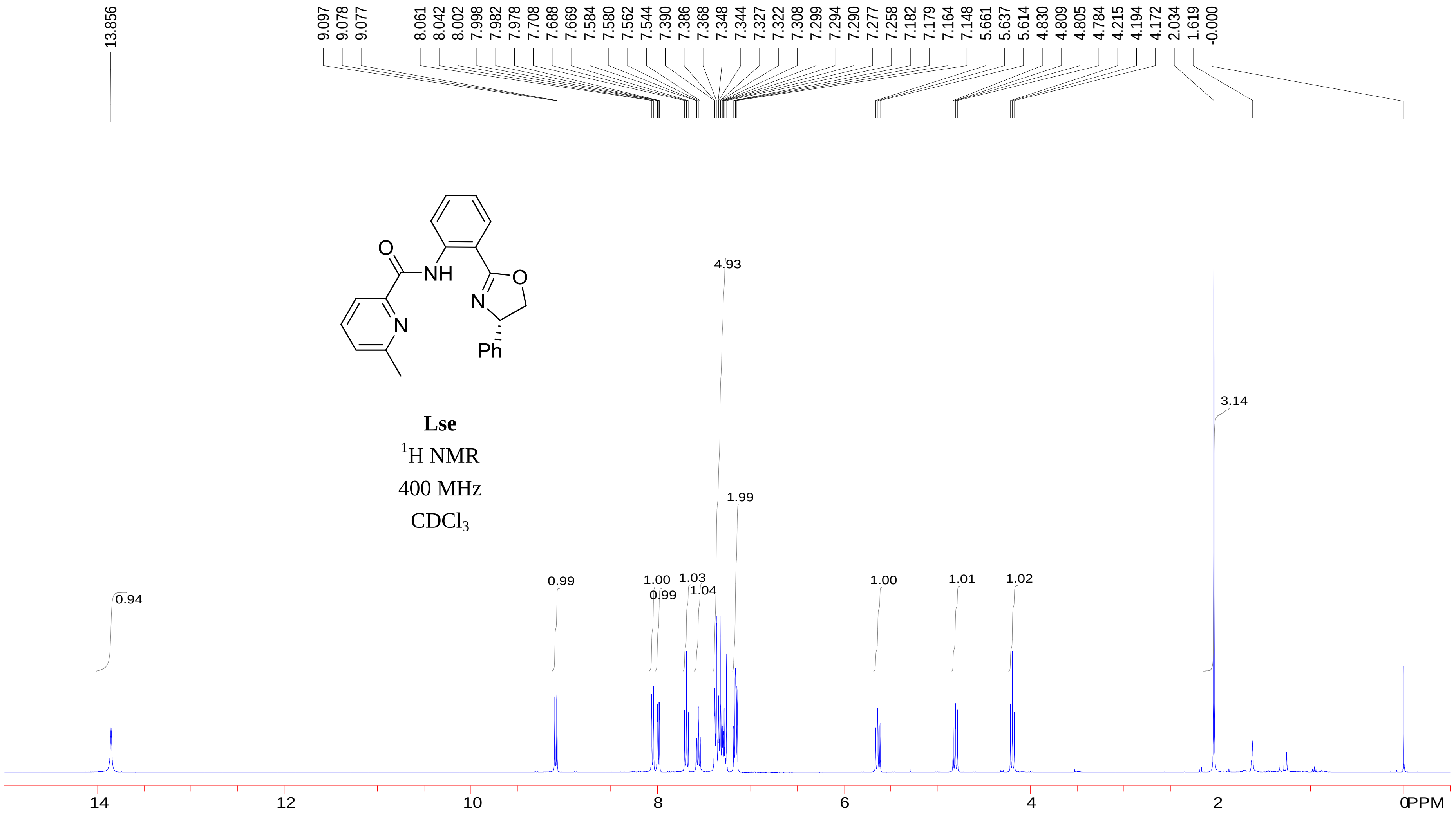

Supplementary Figure 13. ^{1}H NMR spectrum for **Lse**

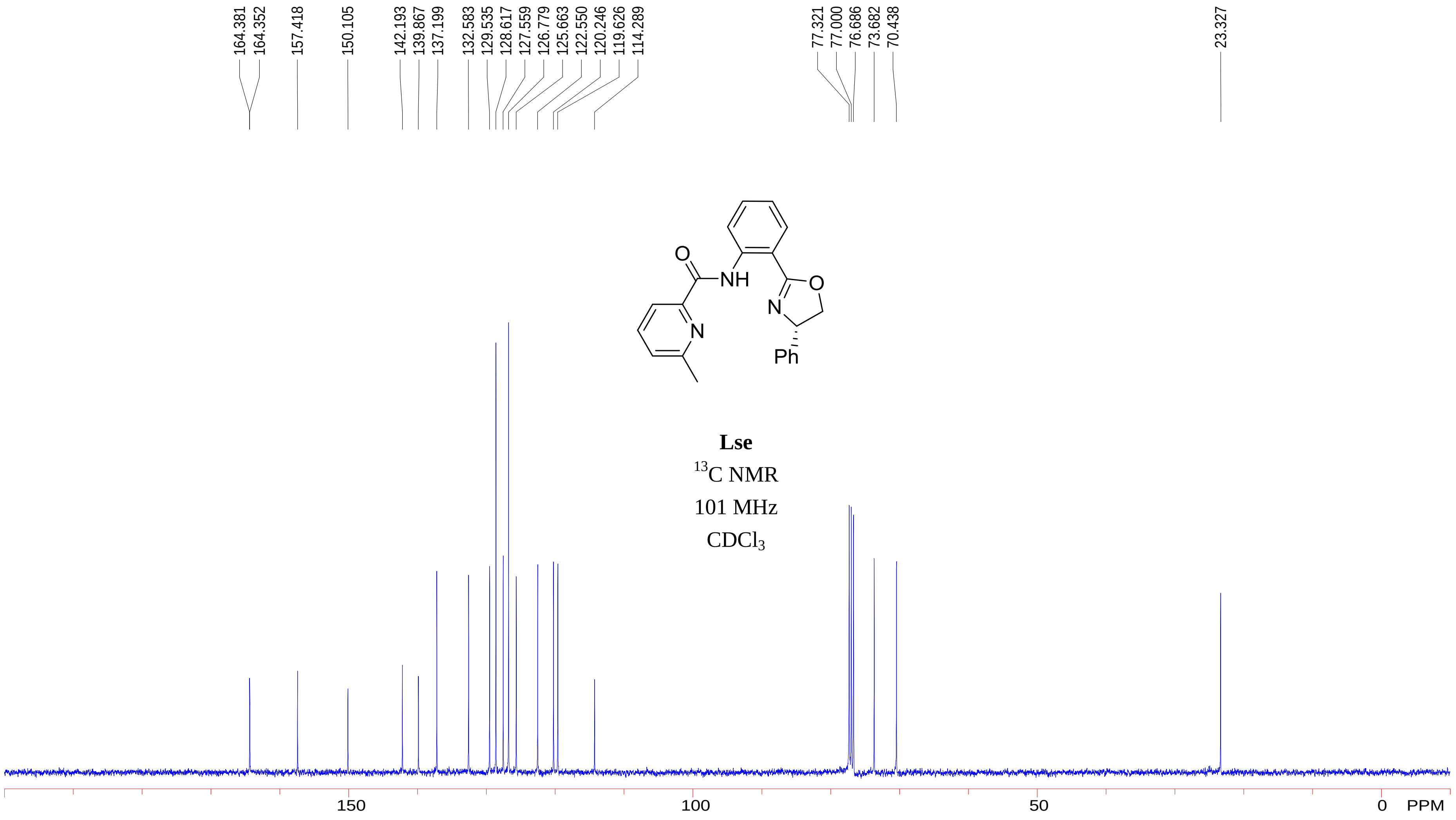

Supplementary Figure 14. ^{13}C NMR spectrum for **Lse**

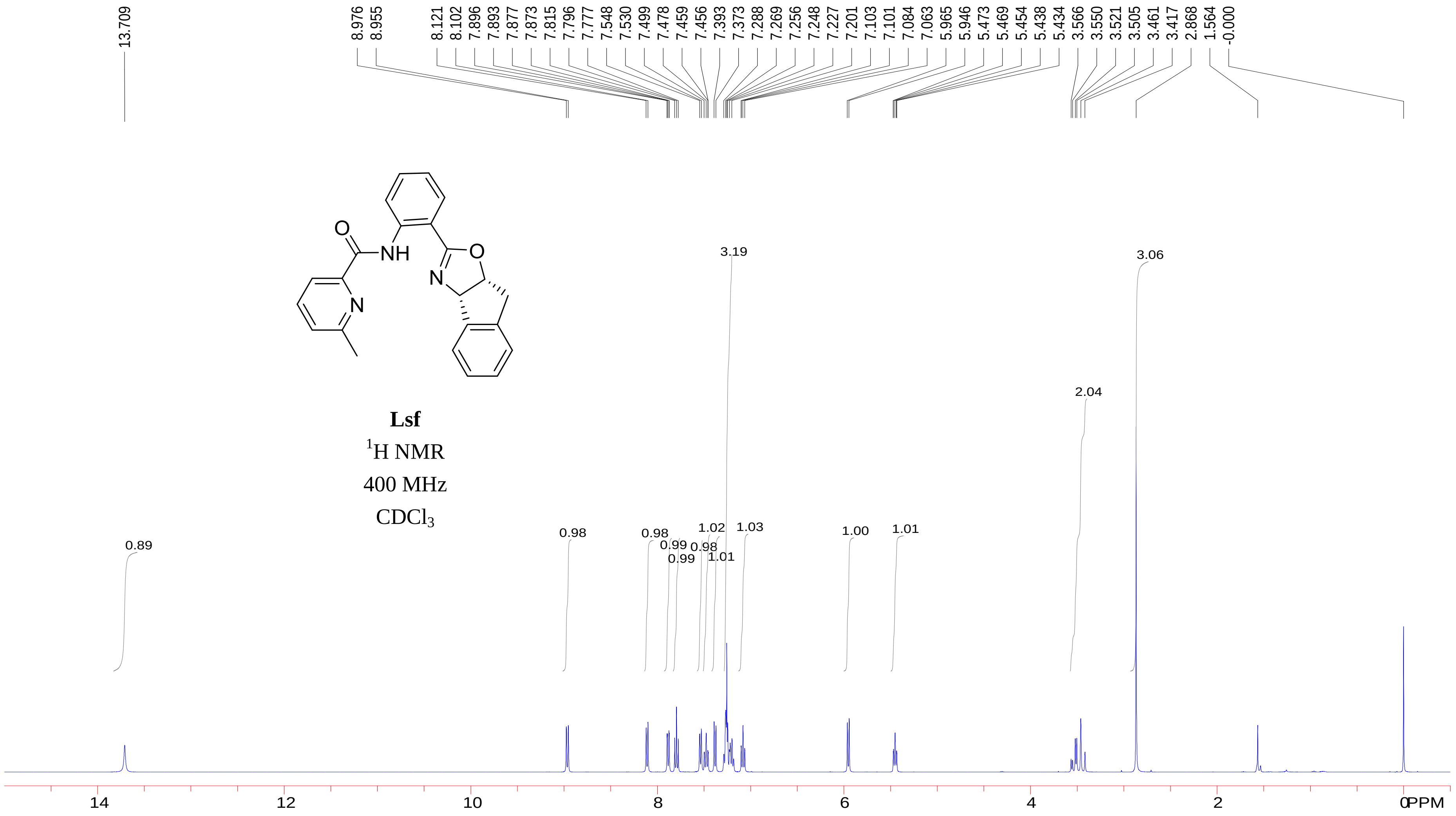

Supplementary Figure 15. ^{1}H NMR spectrum for **Lsf**

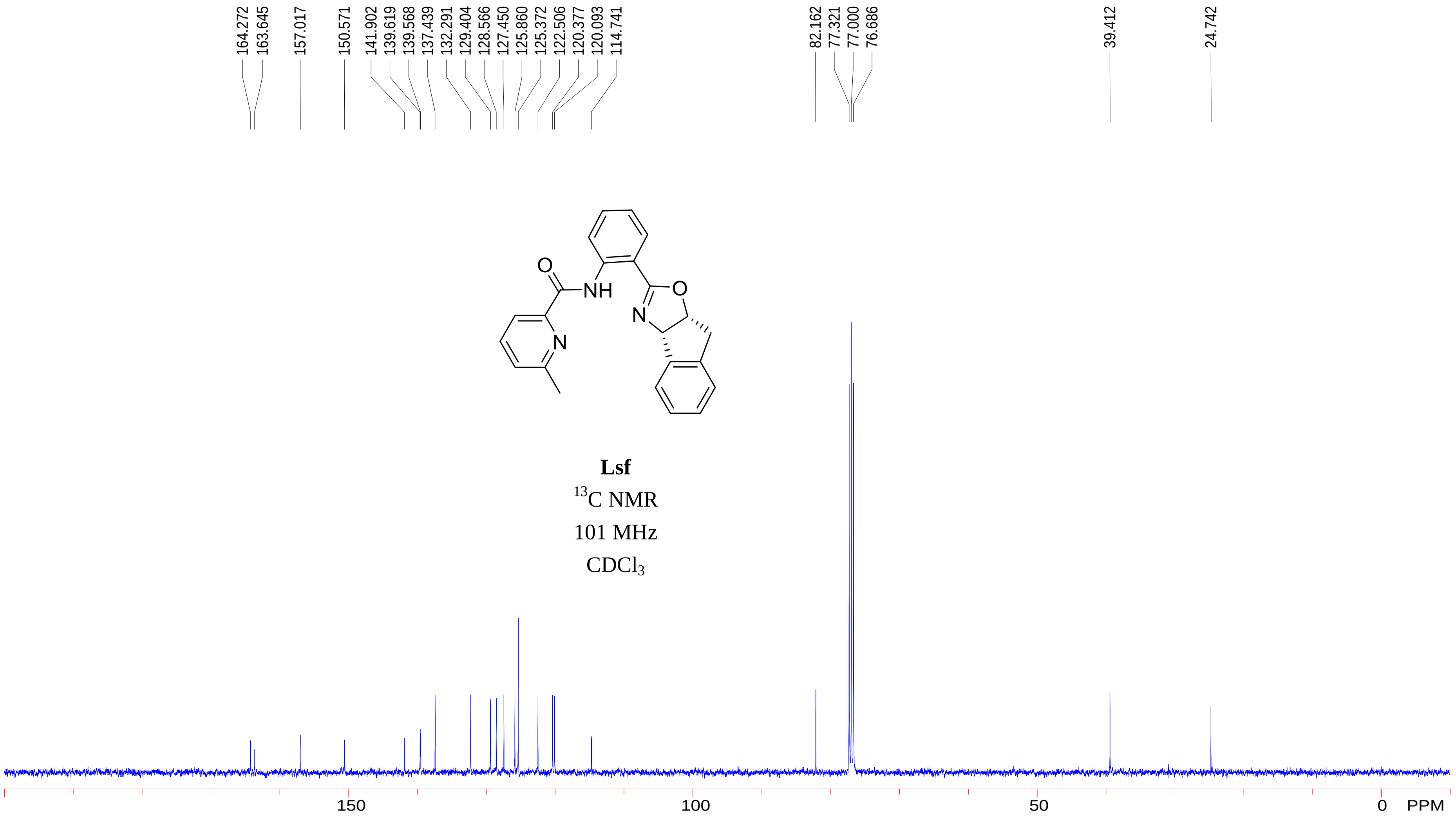

Supplementary Figure 16. ^{13}C NMR spectrum for **Lsf**

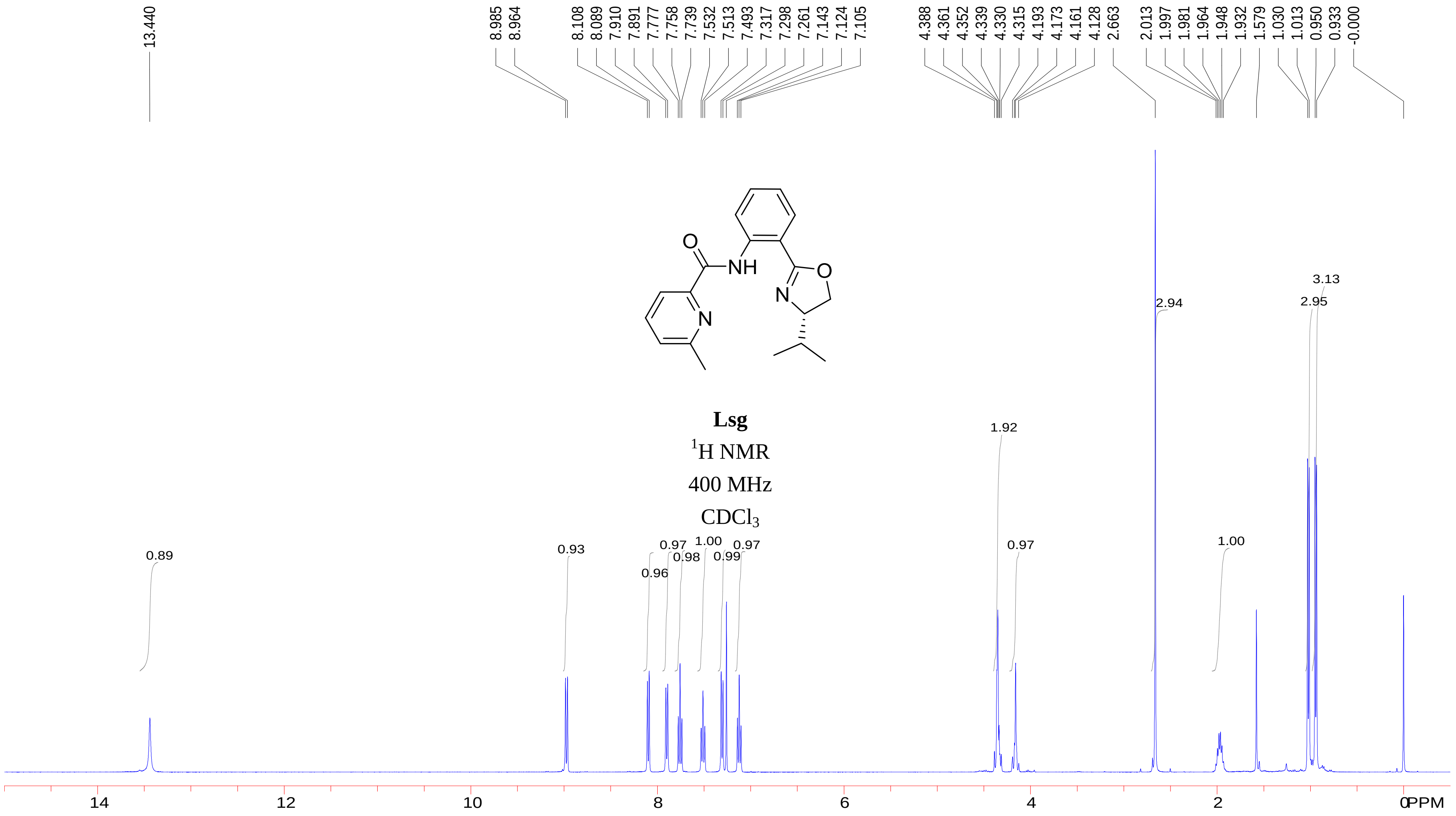

Supplementary Figure 17. ^{1}H NMR spectrum for **Lsd**

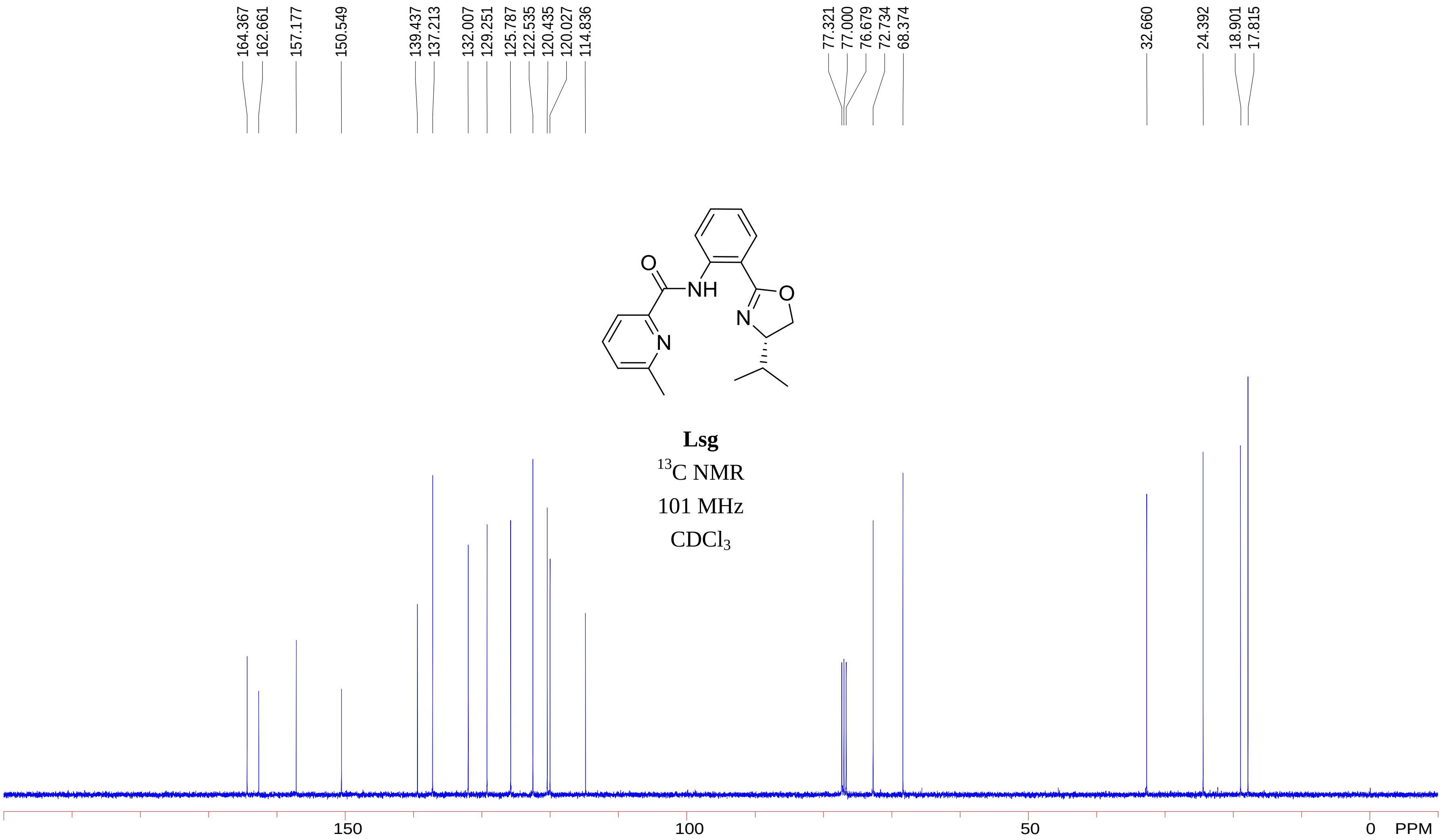

Supplementary Figure 18. ^{13}C NMR spectrum for **Lsg**

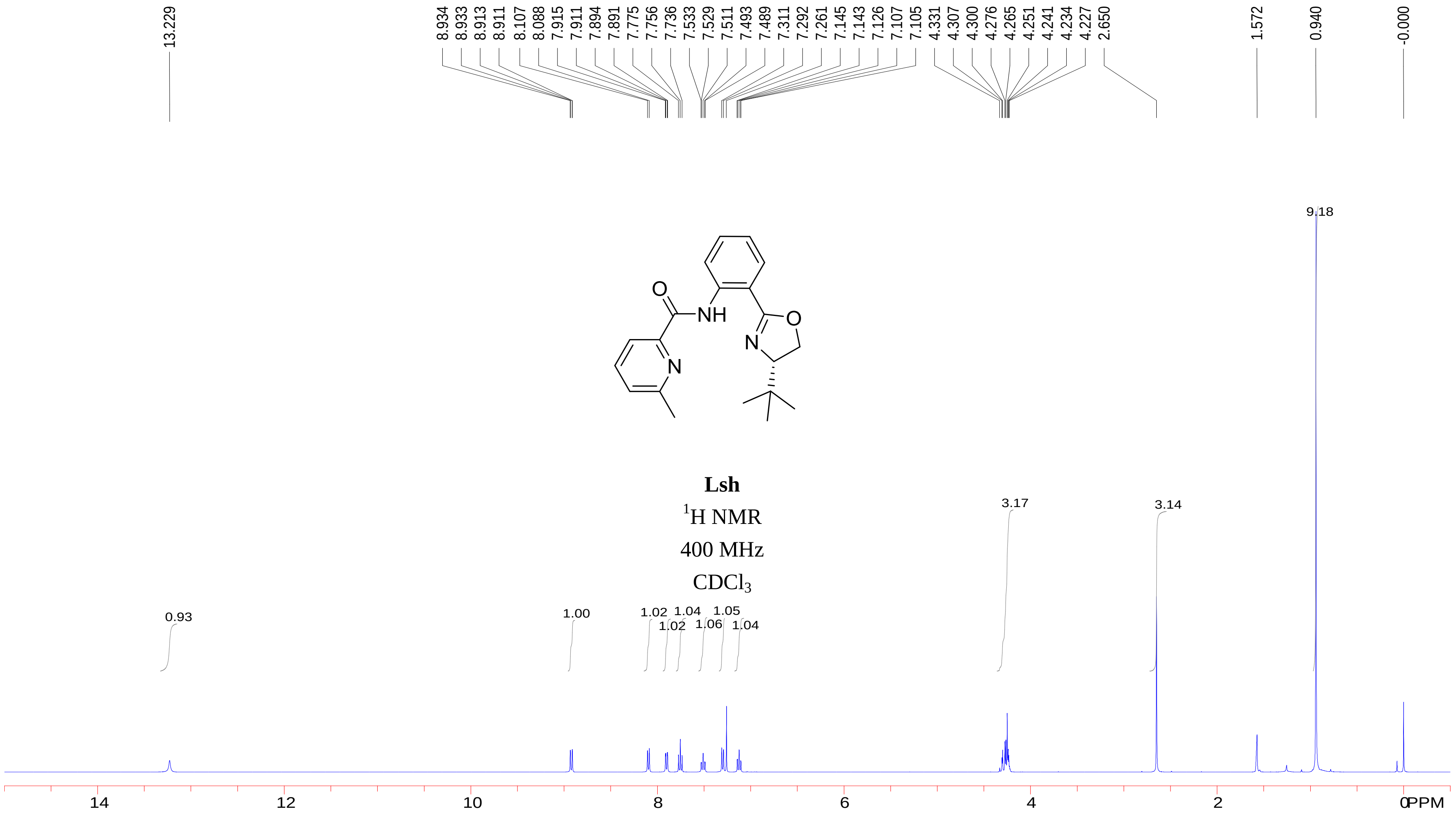

Supplementary Figure 19. ^{1}H NMR spectrum for **Lsh**

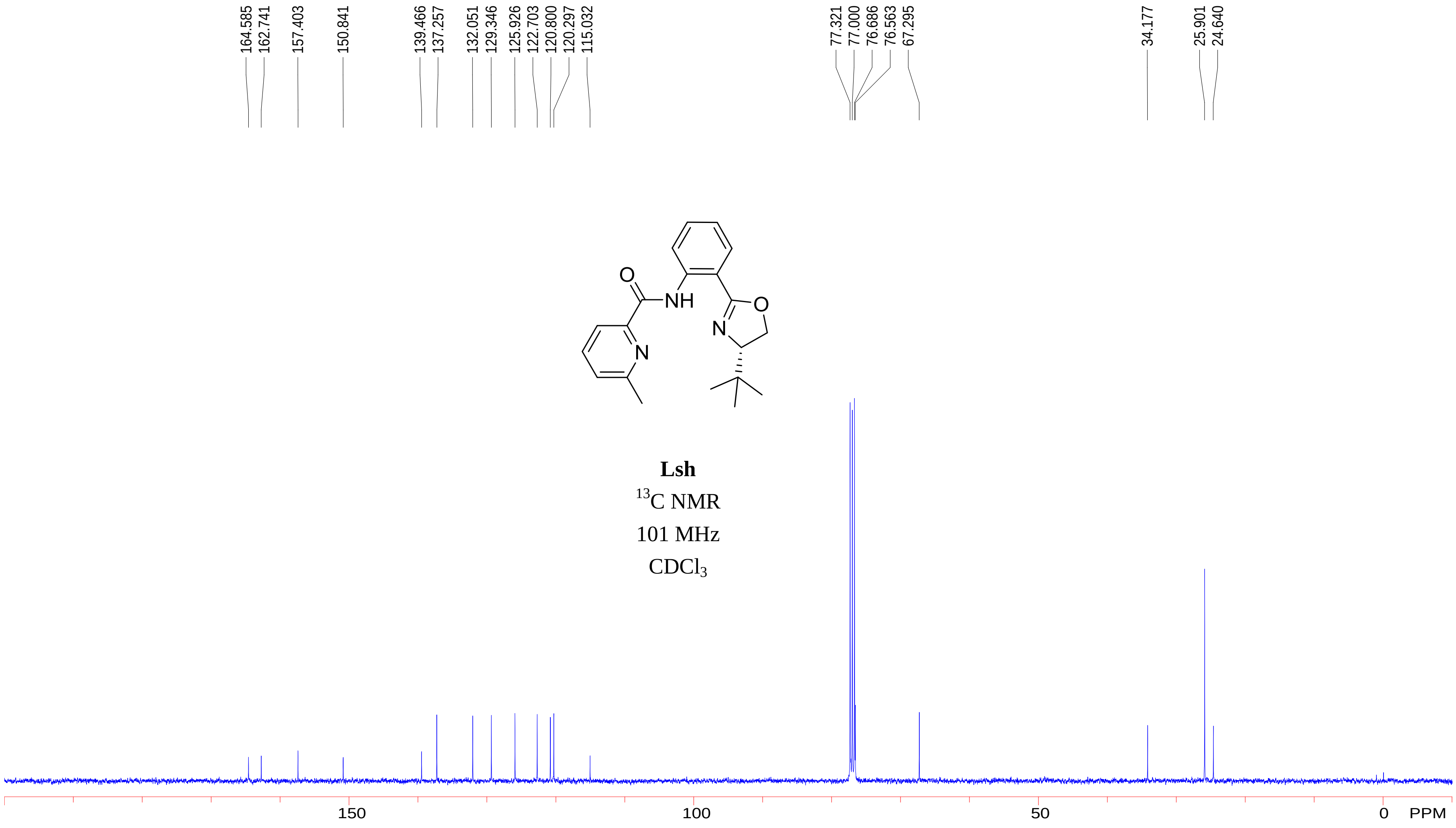

Supplementary Figure 20. ^{13}C NMR spectrum for **Lsh**

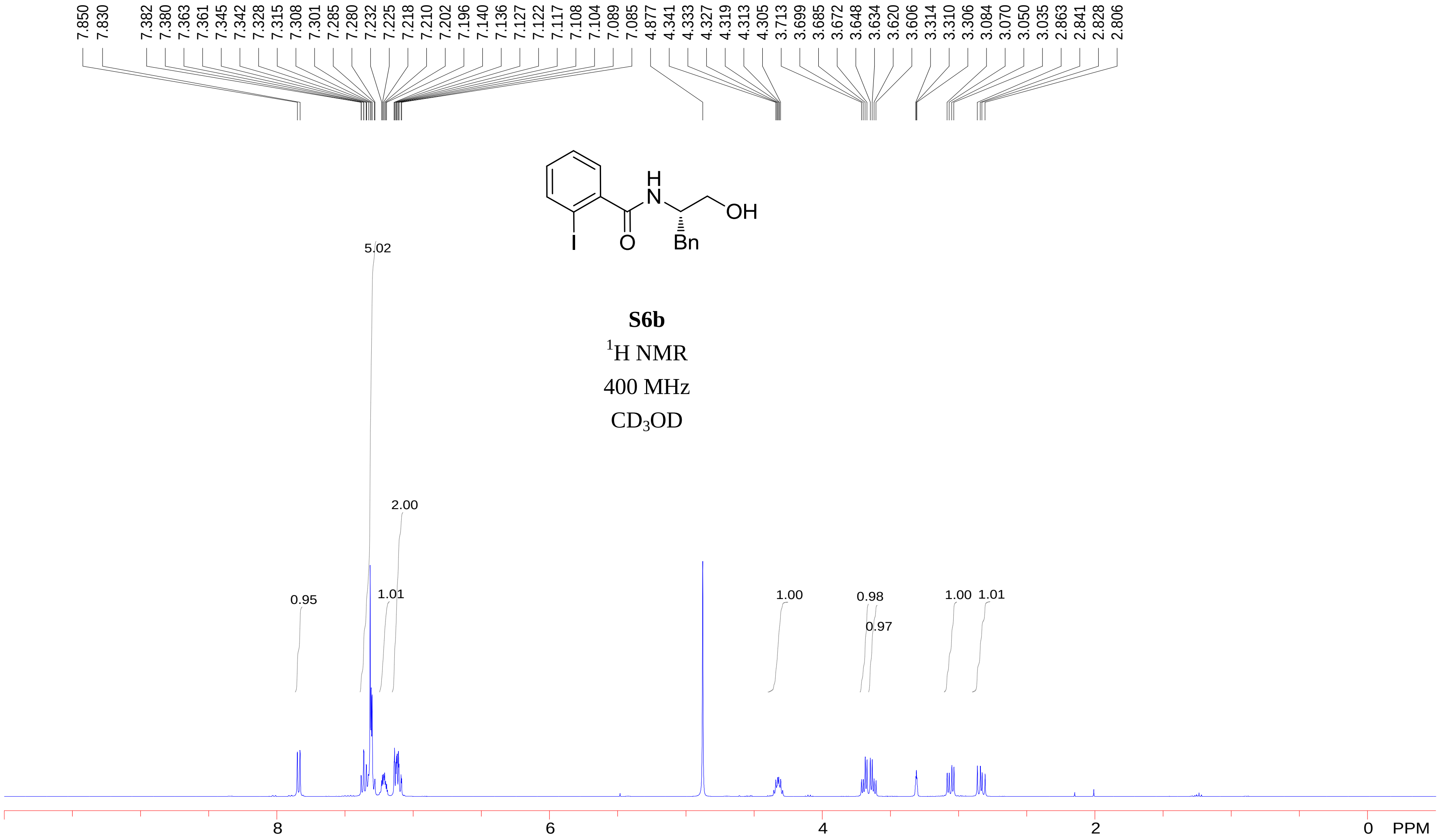

Supplementary Figure 21. ^{1}H NMR spectrum for **s6b**

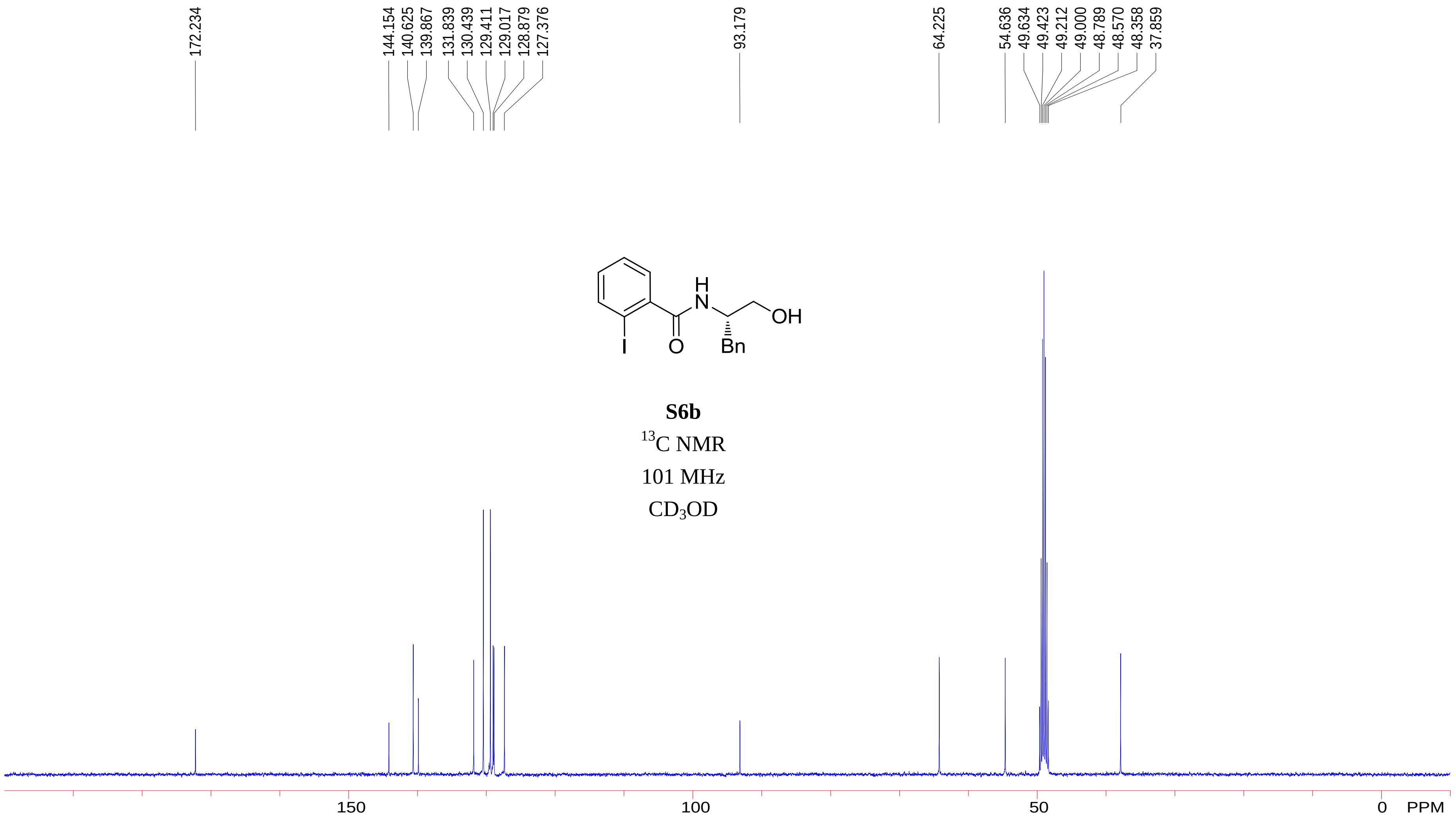

Supplementary Figure 22. ^{13}C NMR spectrum for **s6b**

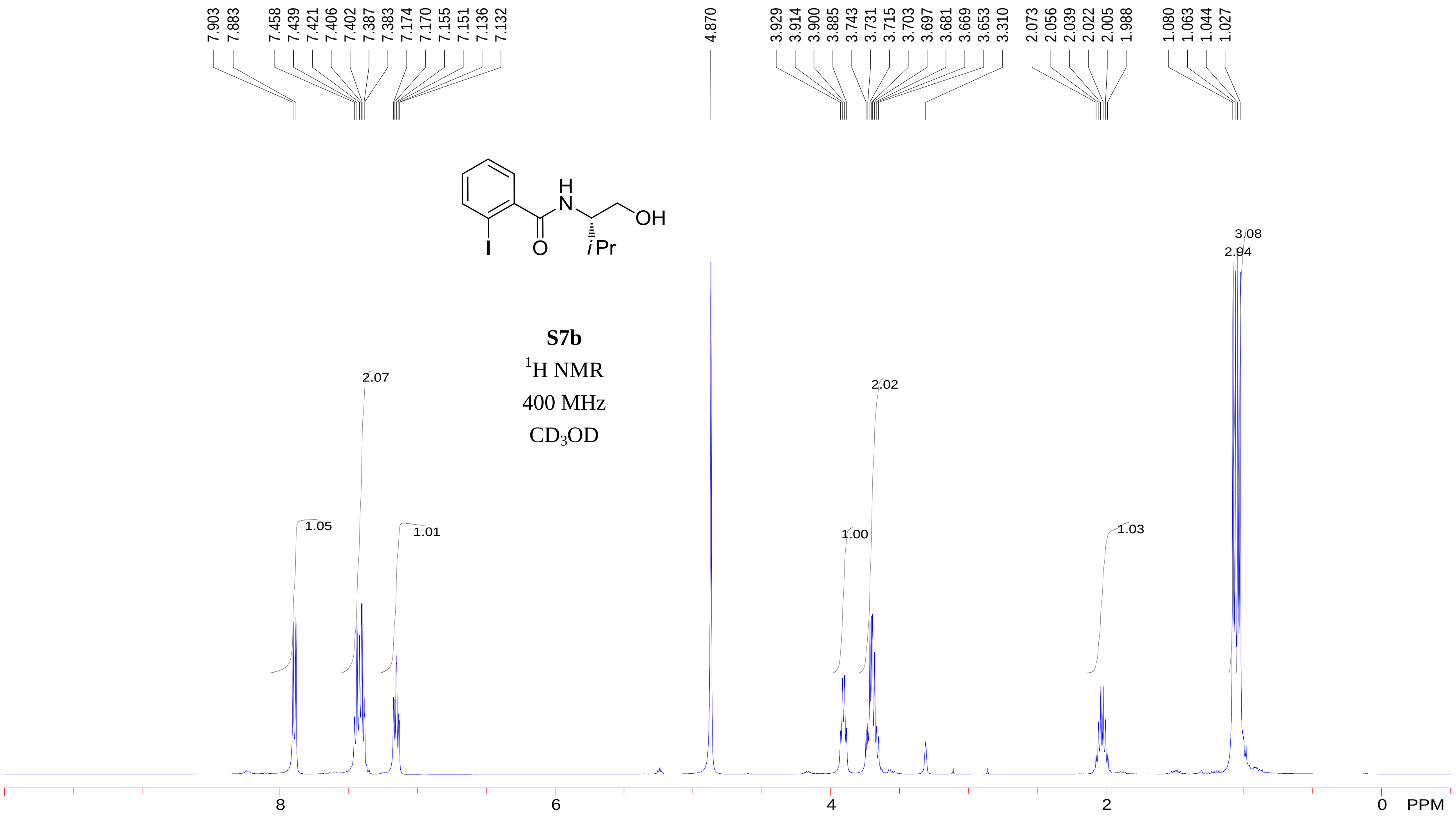

Supplementary Figure 23. ^{1}H NMR spectrum for **s7b**

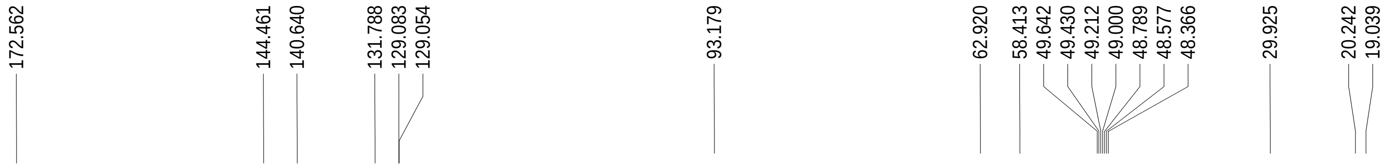

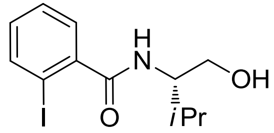

S7b

^{13}C NMR

101 MHz

CD_3OD

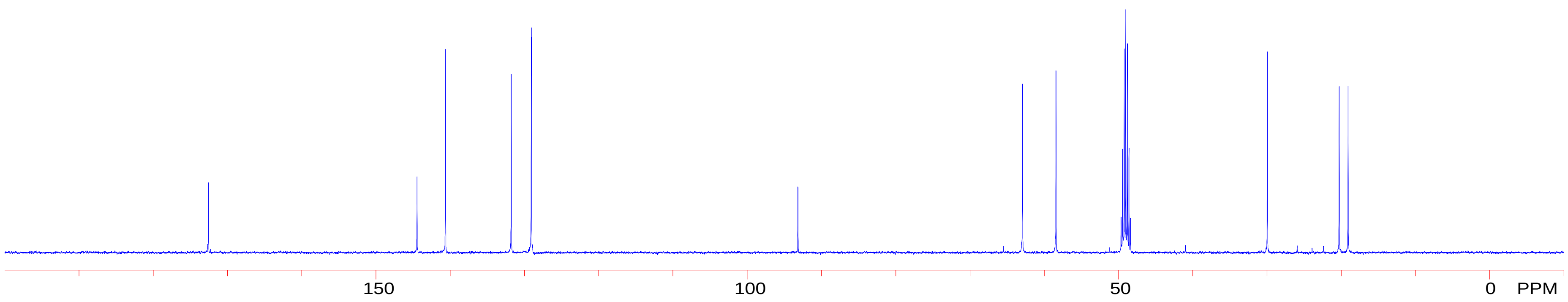

Supplementary Figure 24. ^{13}C NMR spectrum for **s7b**

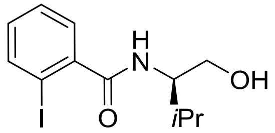

ent-S7b
^{1}H NMR
400 MHz
$CDCl_3$

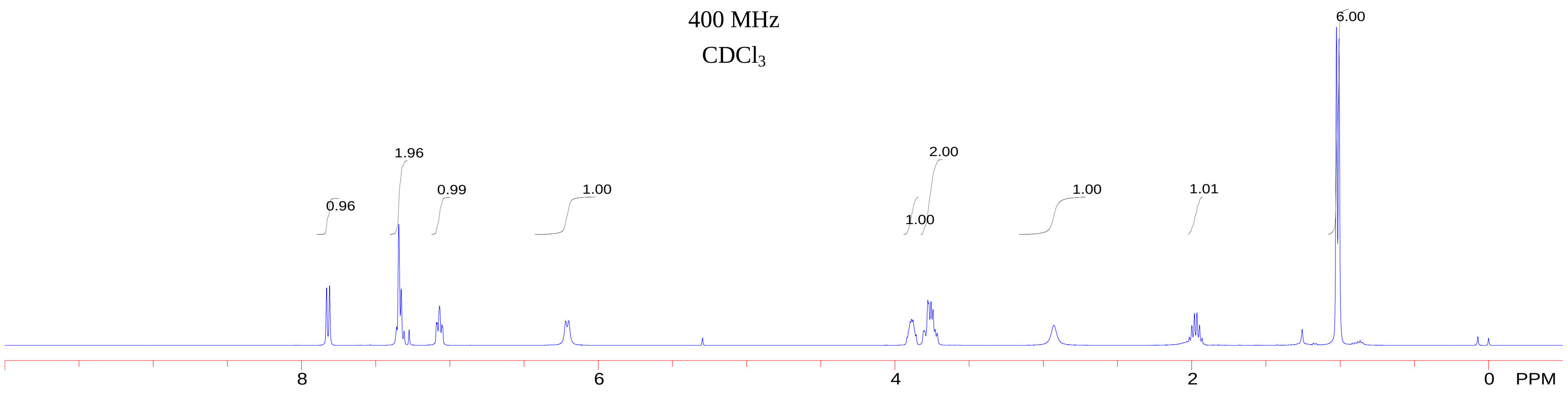

Supplementary Figure 25. ^{1}H NMR spectrum for **ent-S7b**

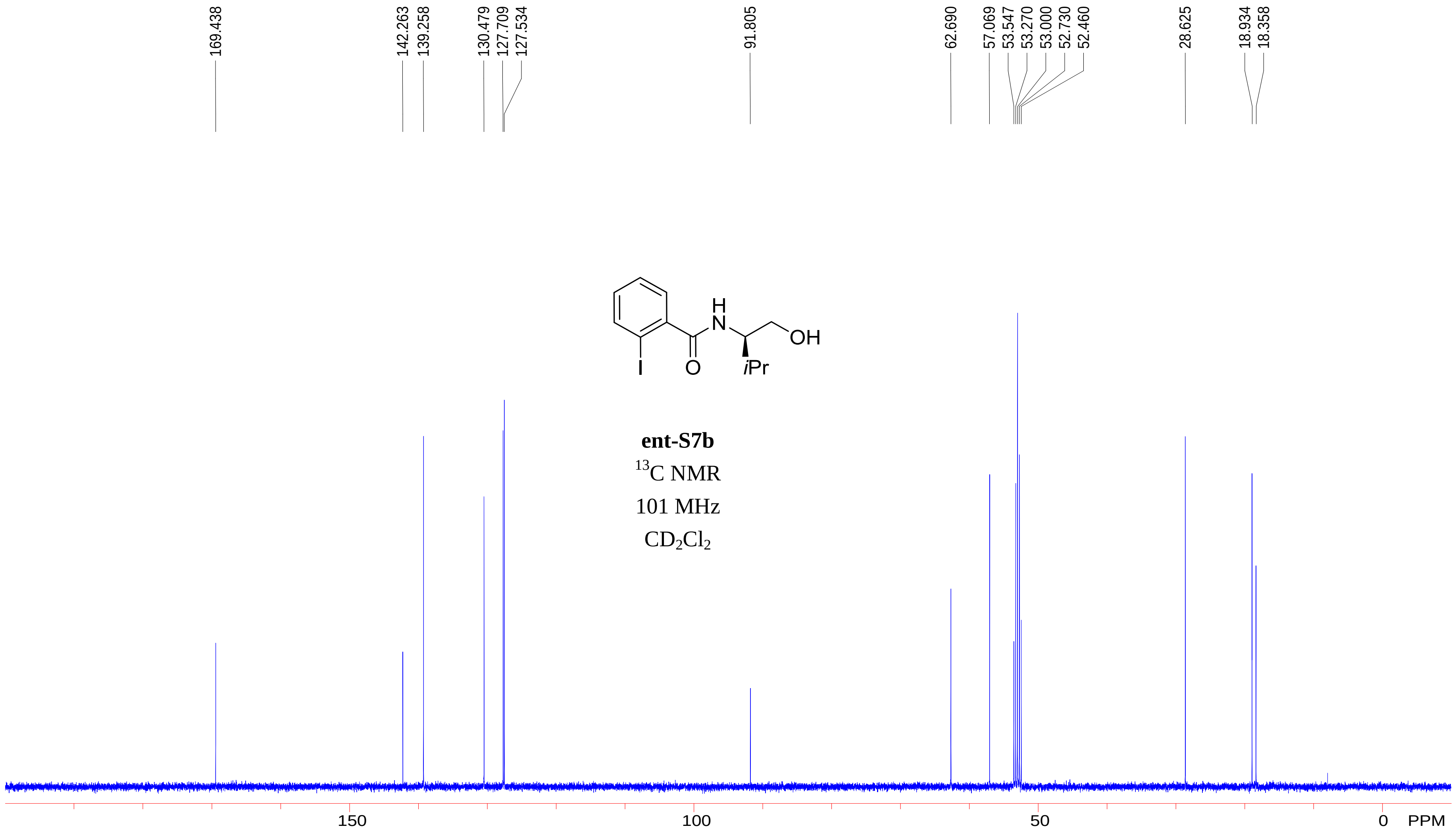

Supplementary Figure 26. ^{13}C NMR spectrum for **ent-s7b**

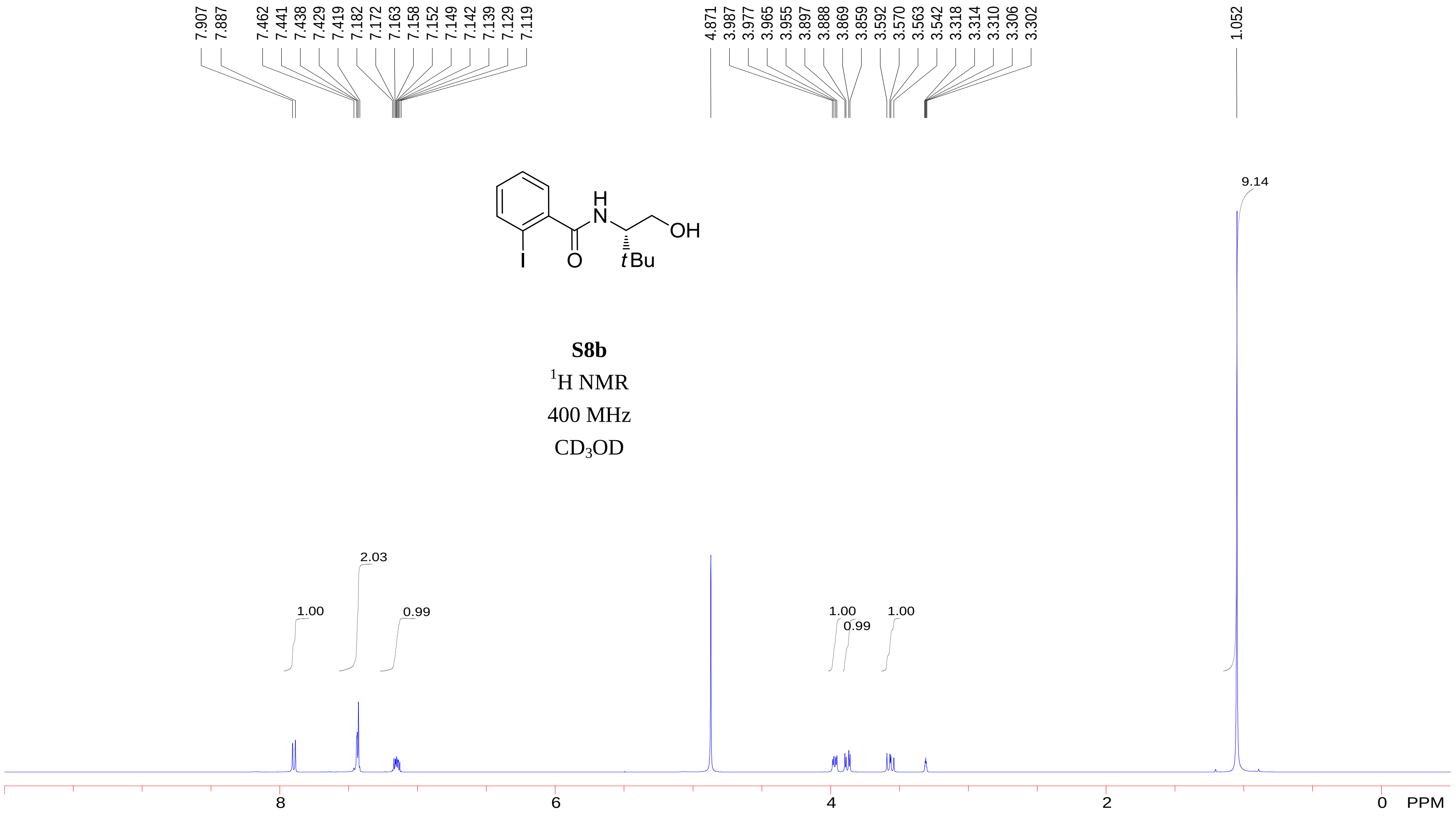

Supplementary Figure 27. ^{1}H NMR spectrum for **S8b**

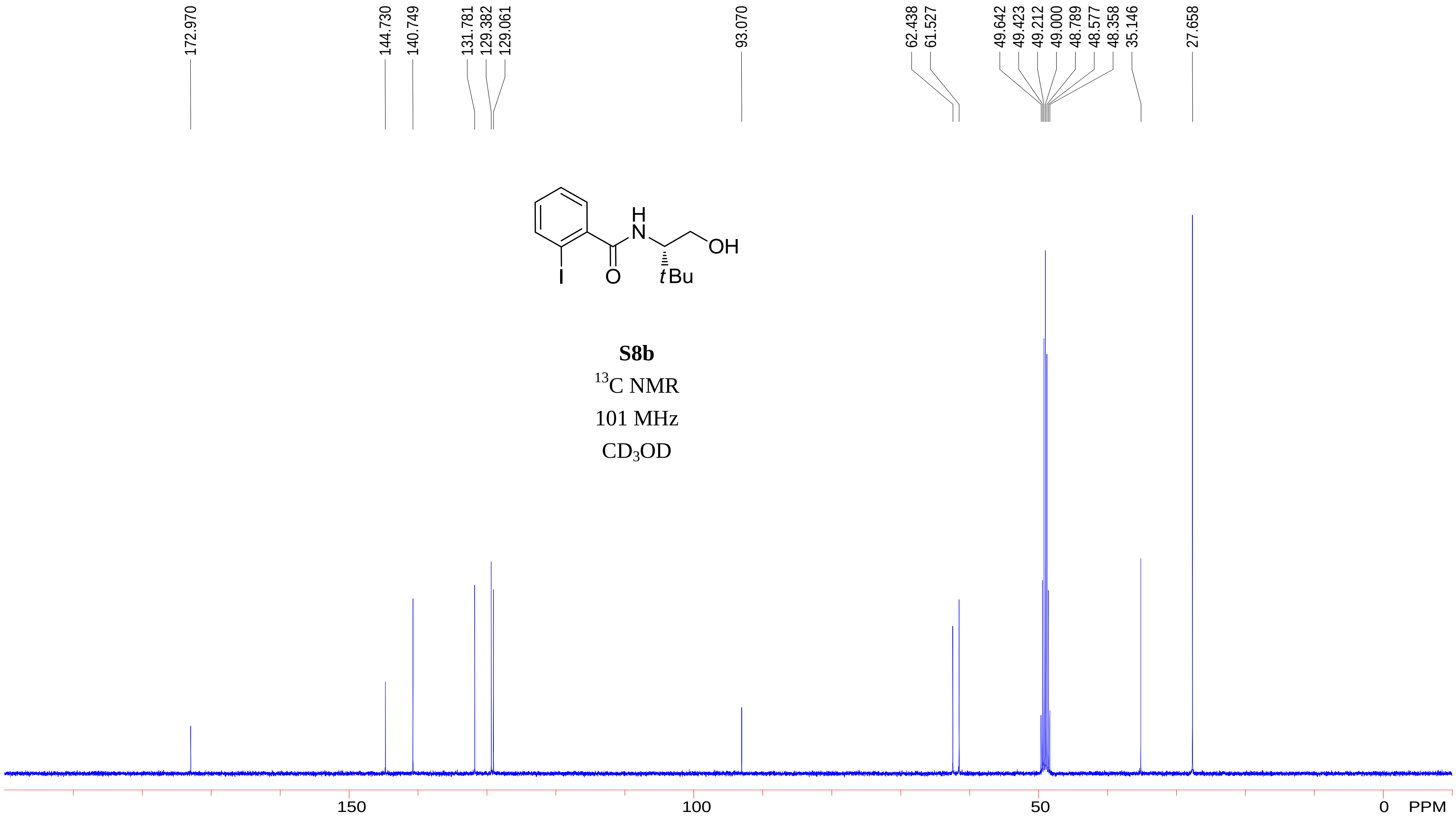

Supplementary Figure 28. ^{13}C NMR spectrum for **S8b**

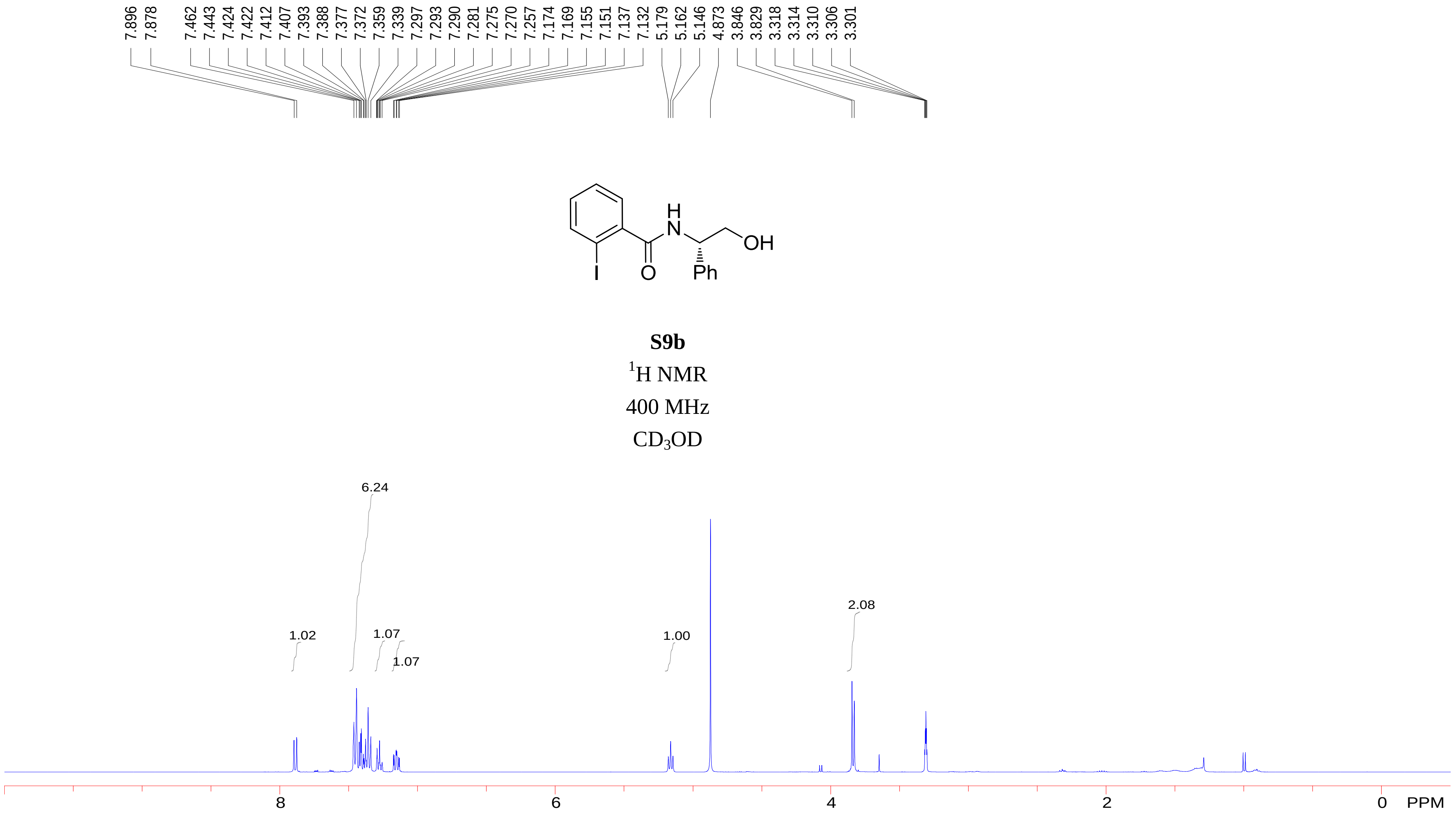

Supplementary Figure 29. ^{1}H NMR spectrum for **S9b**

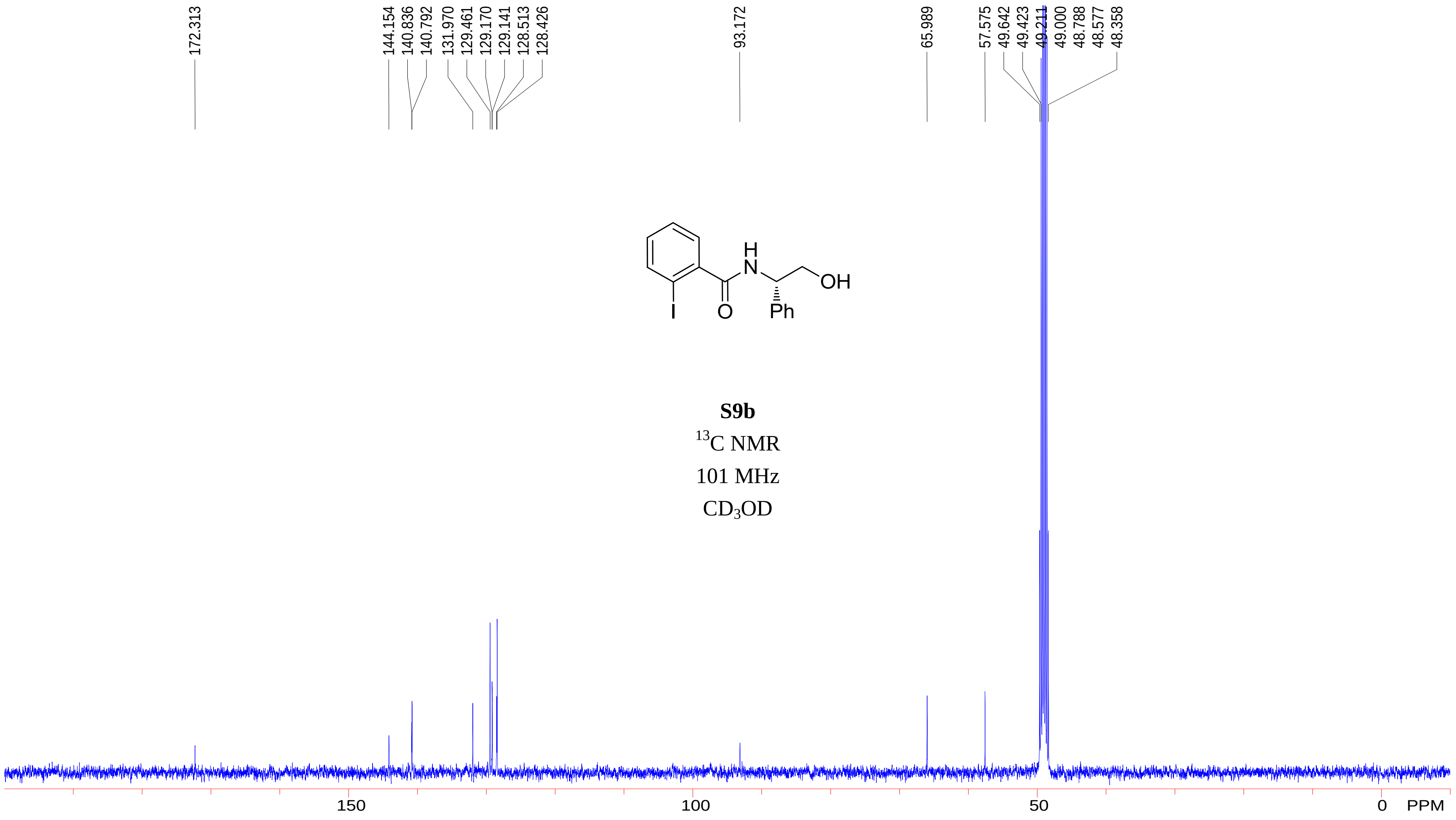

Supplementary Figure 30. ^{13}C NMR spectrum for **S9b**

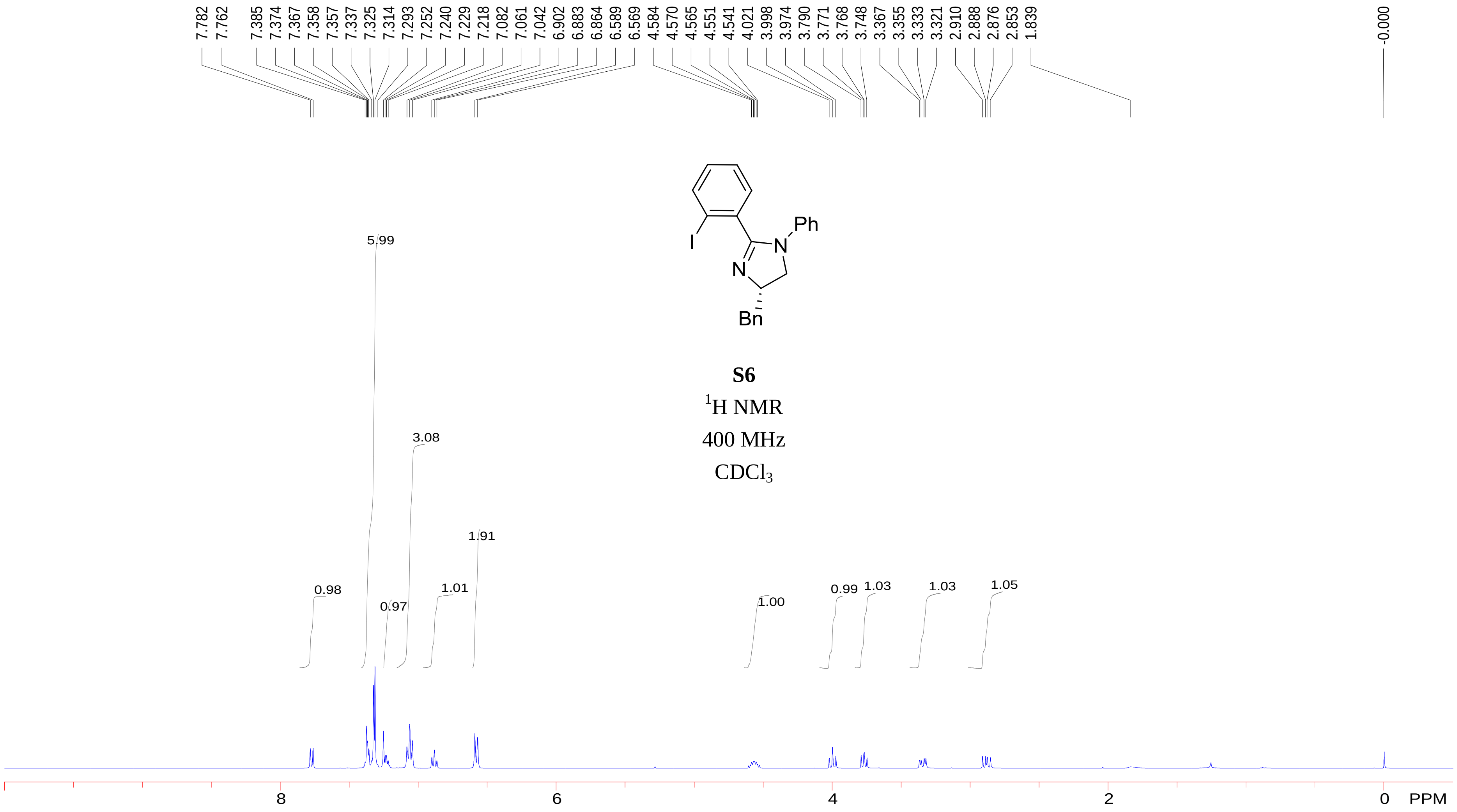

Supplementary Figure 31. ^{1}H NMR spectrum for **S6**

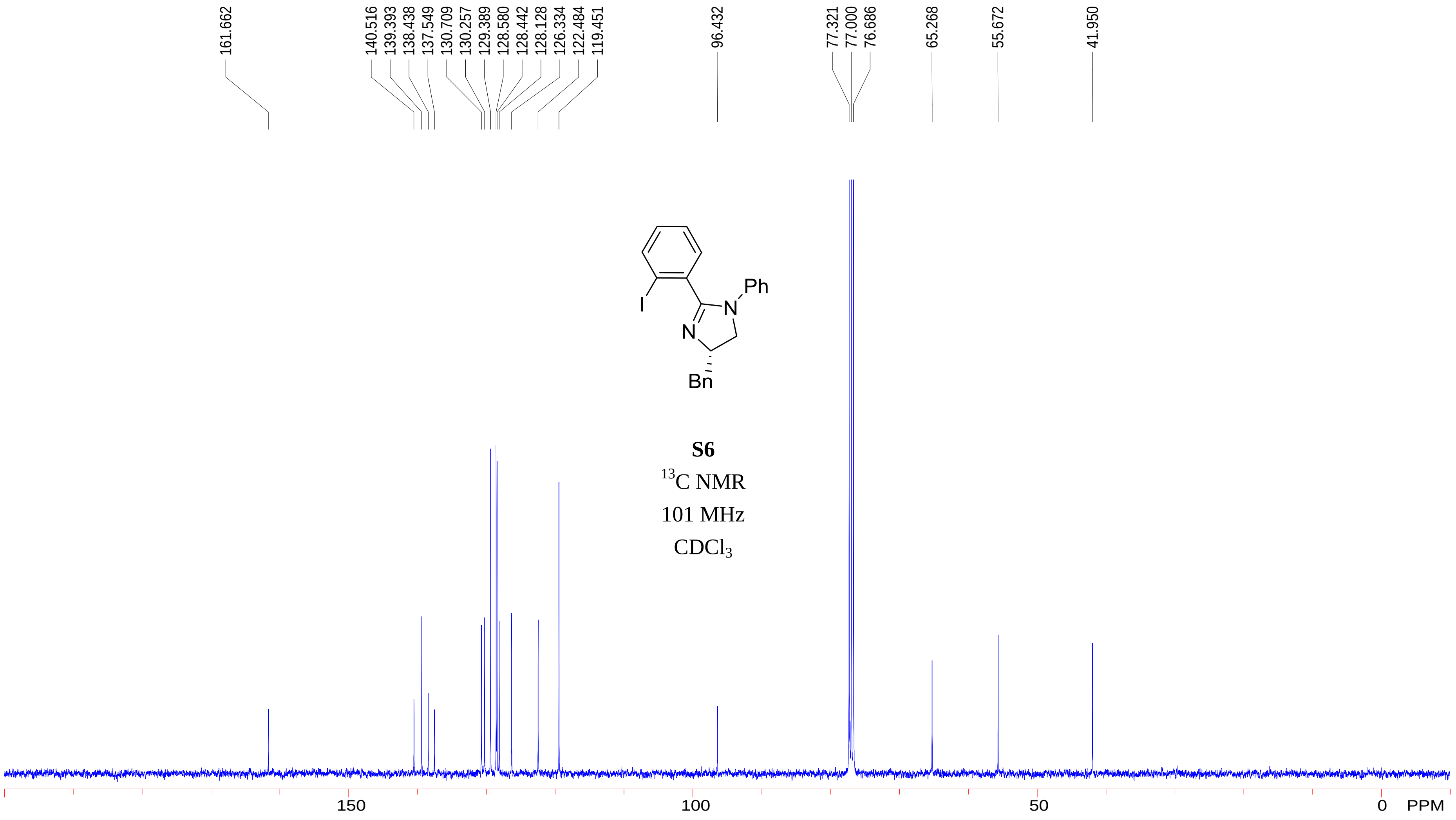

Supplementary Figure 32. ^{13}C NMR spectrum for **S6**

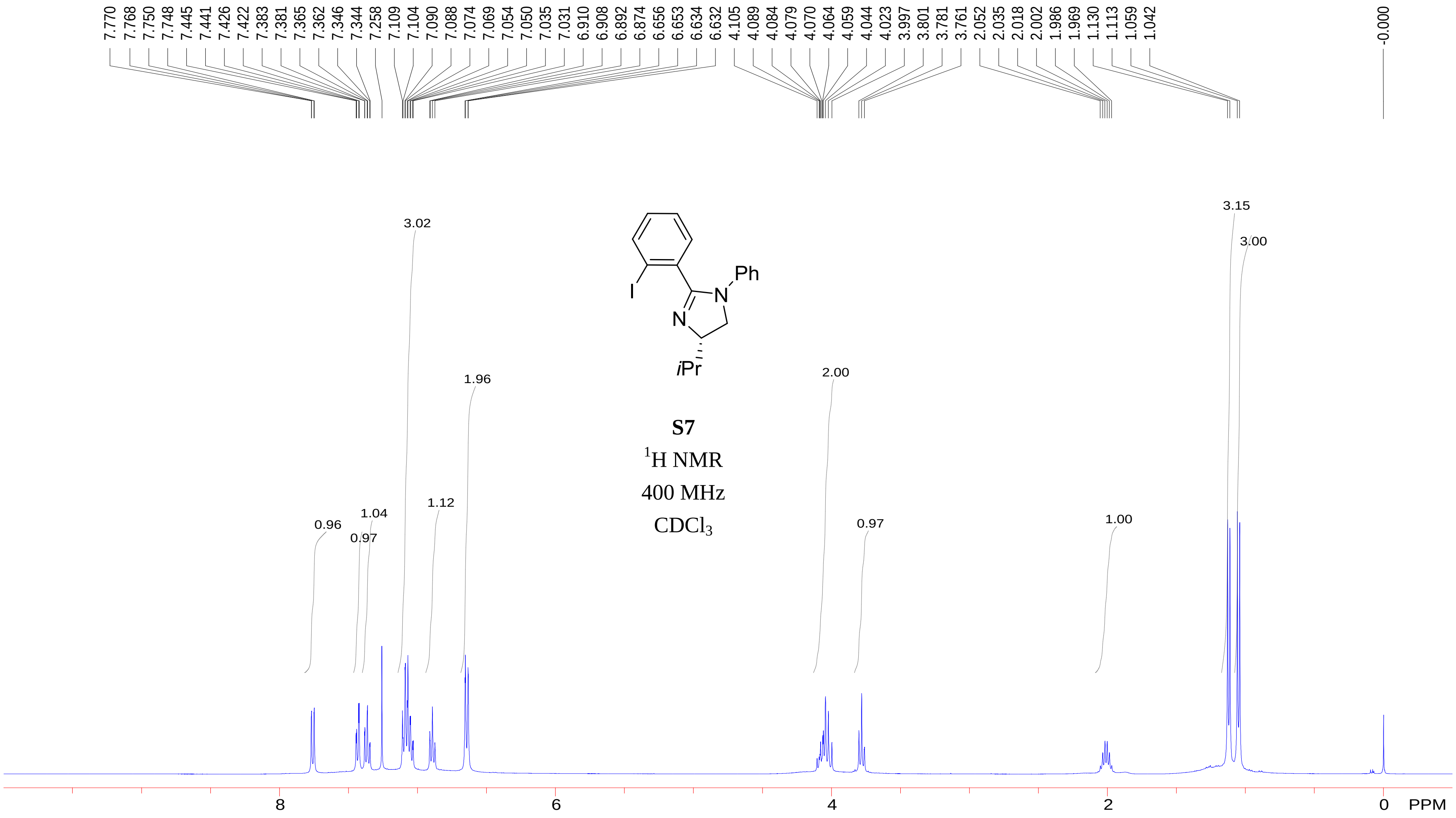

Supplementary Figure 33. ^{1}H NMR spectrum for **S7**

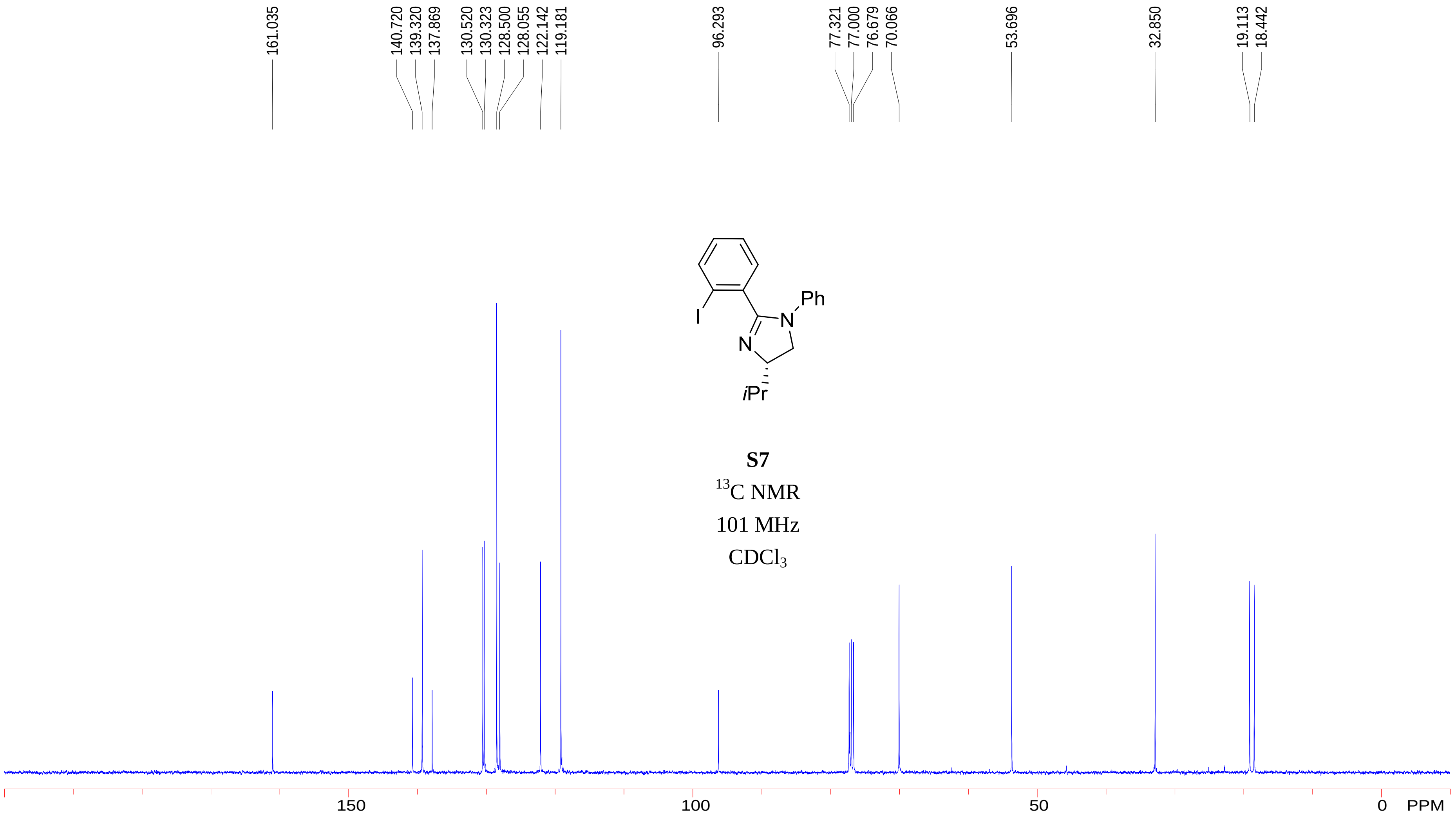

Supplementary Figure 34. ^{13}C NMR spectrum for **S7**

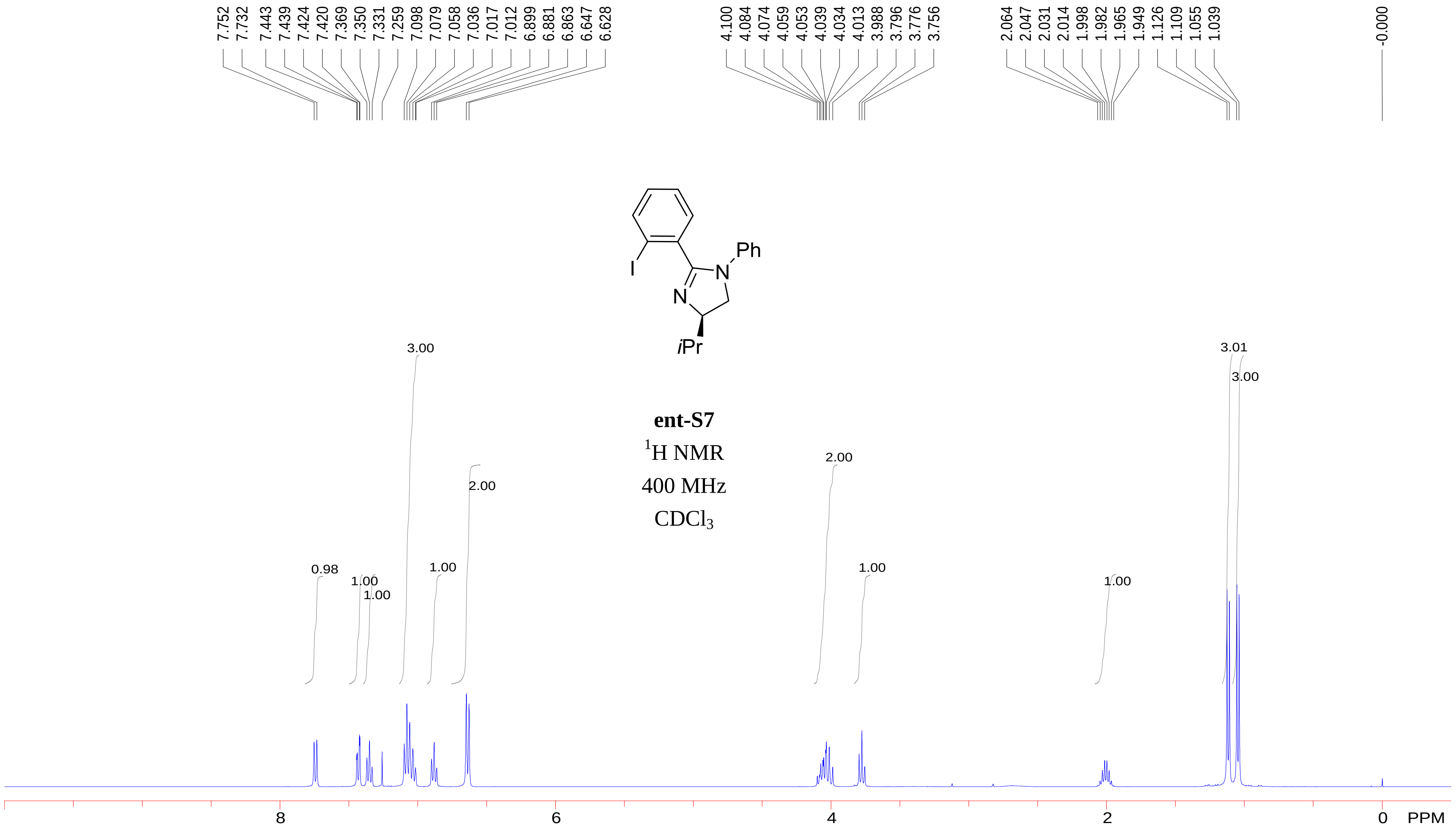

Supplementary Figure 35. ^{1}H NMR spectrum for **ent-S7**

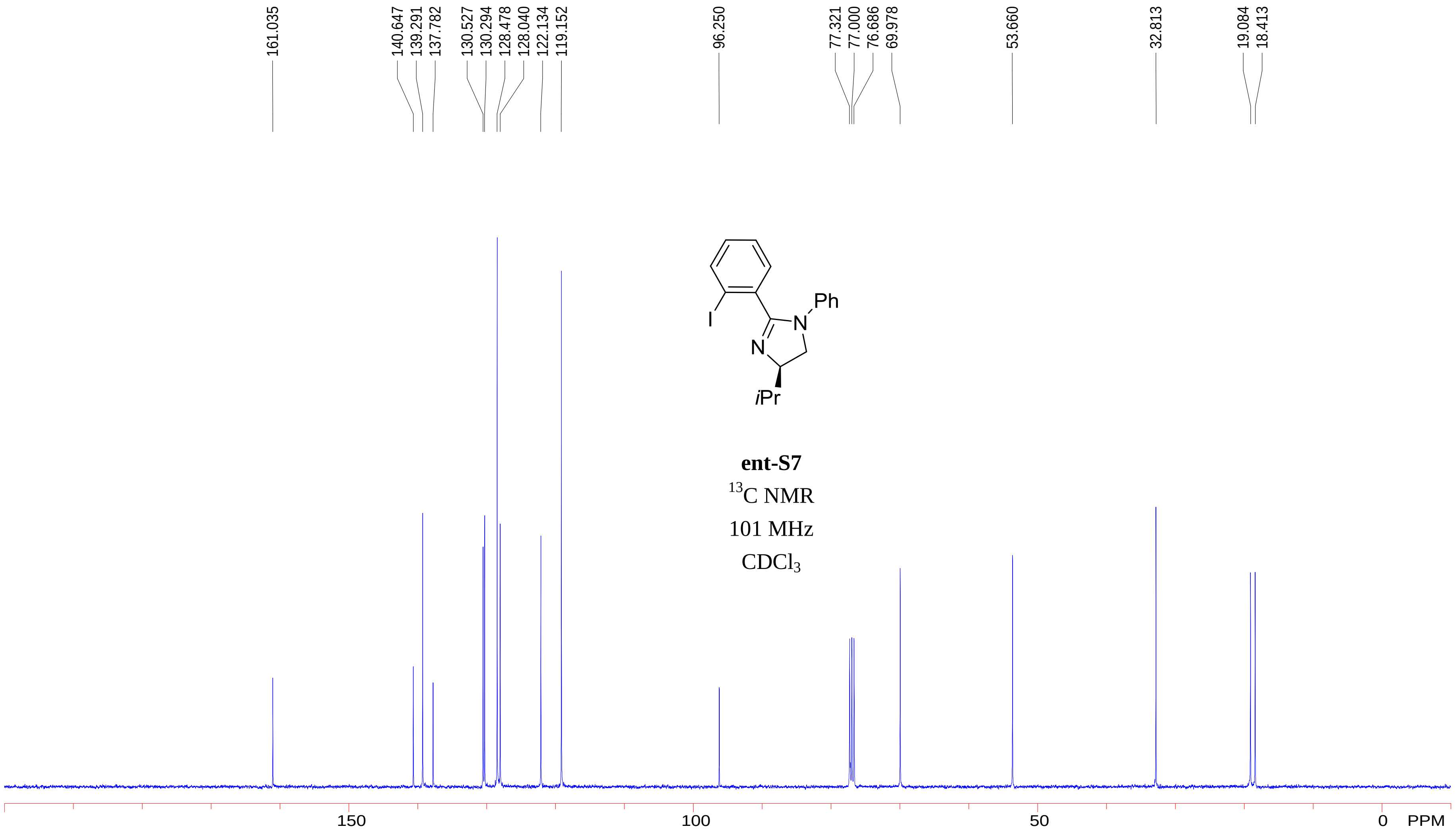

Supplementary Figure 36. ^{13}C NMR spectrum for **ent-S7**

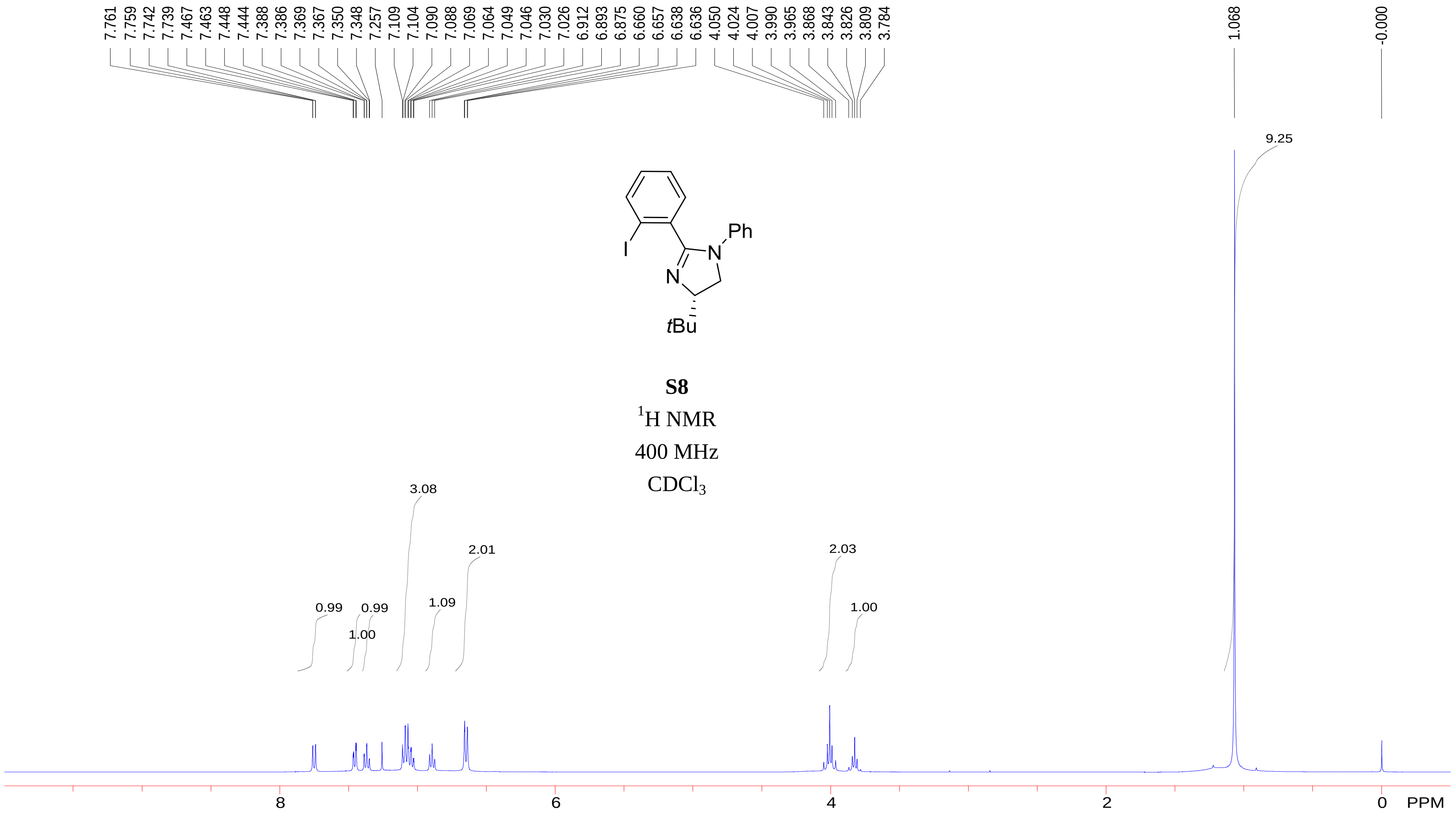

Supplementary Figure 37. ^{1}H NMR spectrum for **S8**

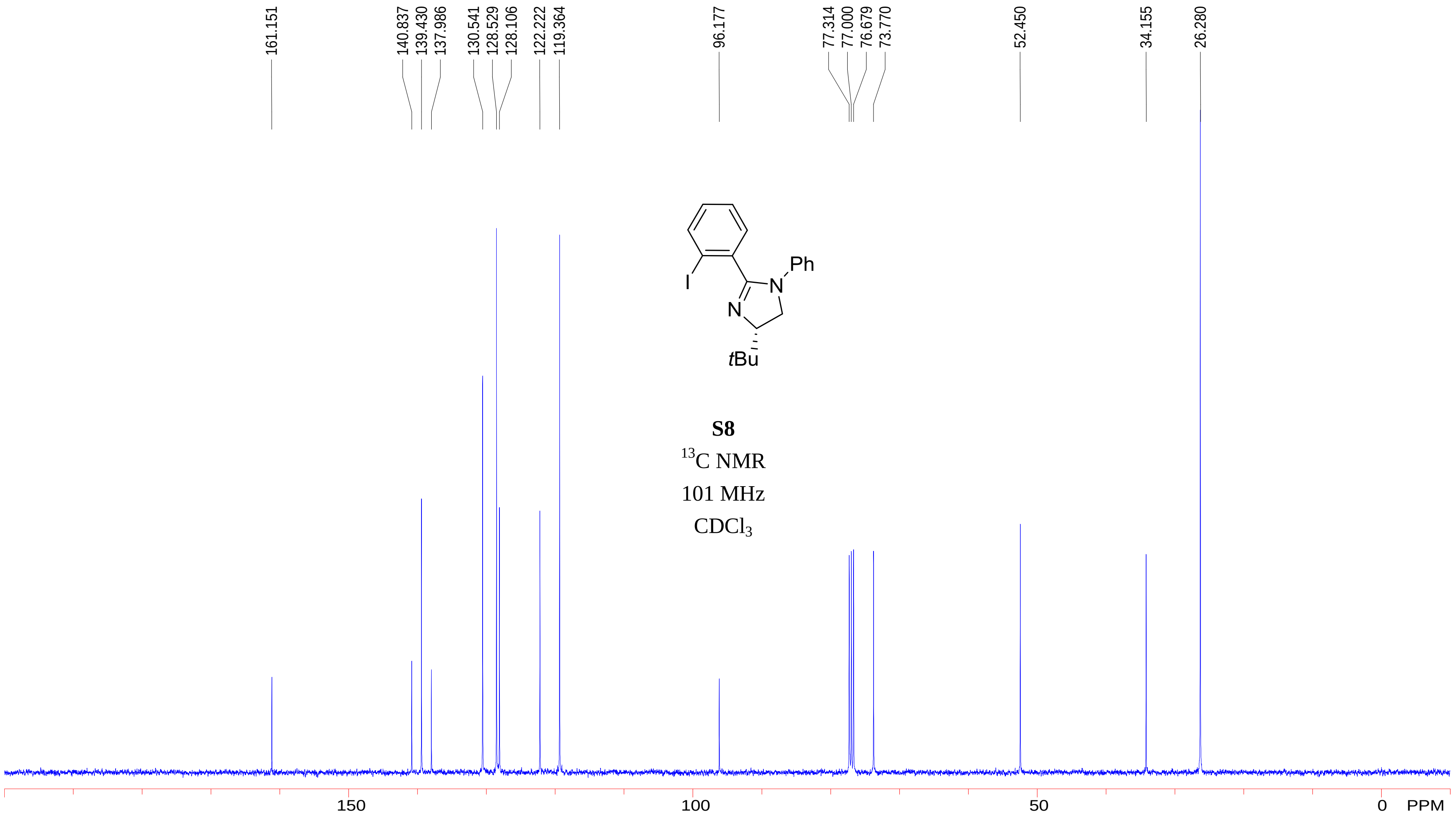

Supplementary Figure 38. ^{13}C NMR spectrum for **S8**

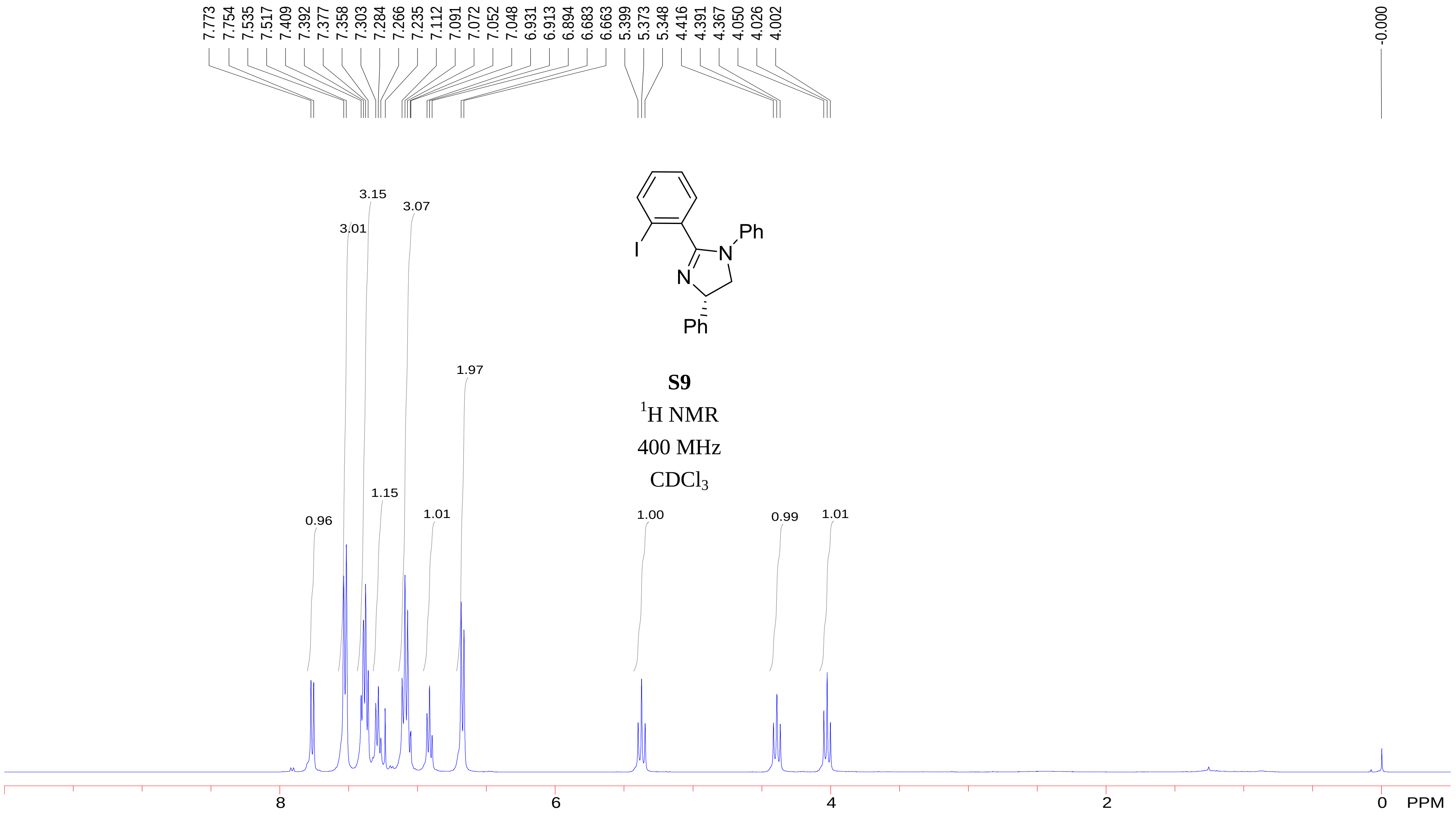

Supplementary Figure 39. ^{1}H NMR spectrum for **S9**

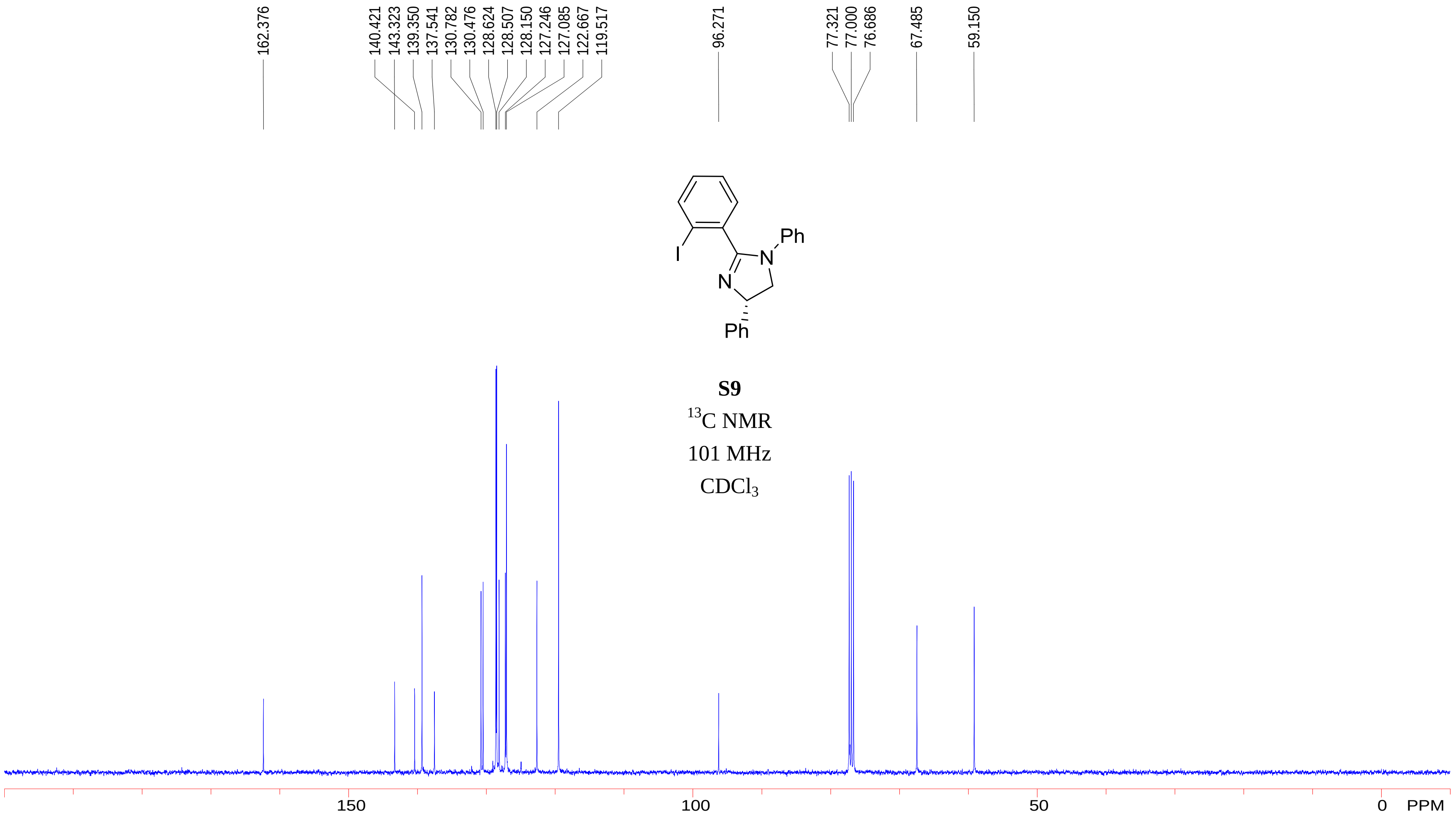

Supplementary Figure 40. ^{13}C NMR spectrum for **S9**

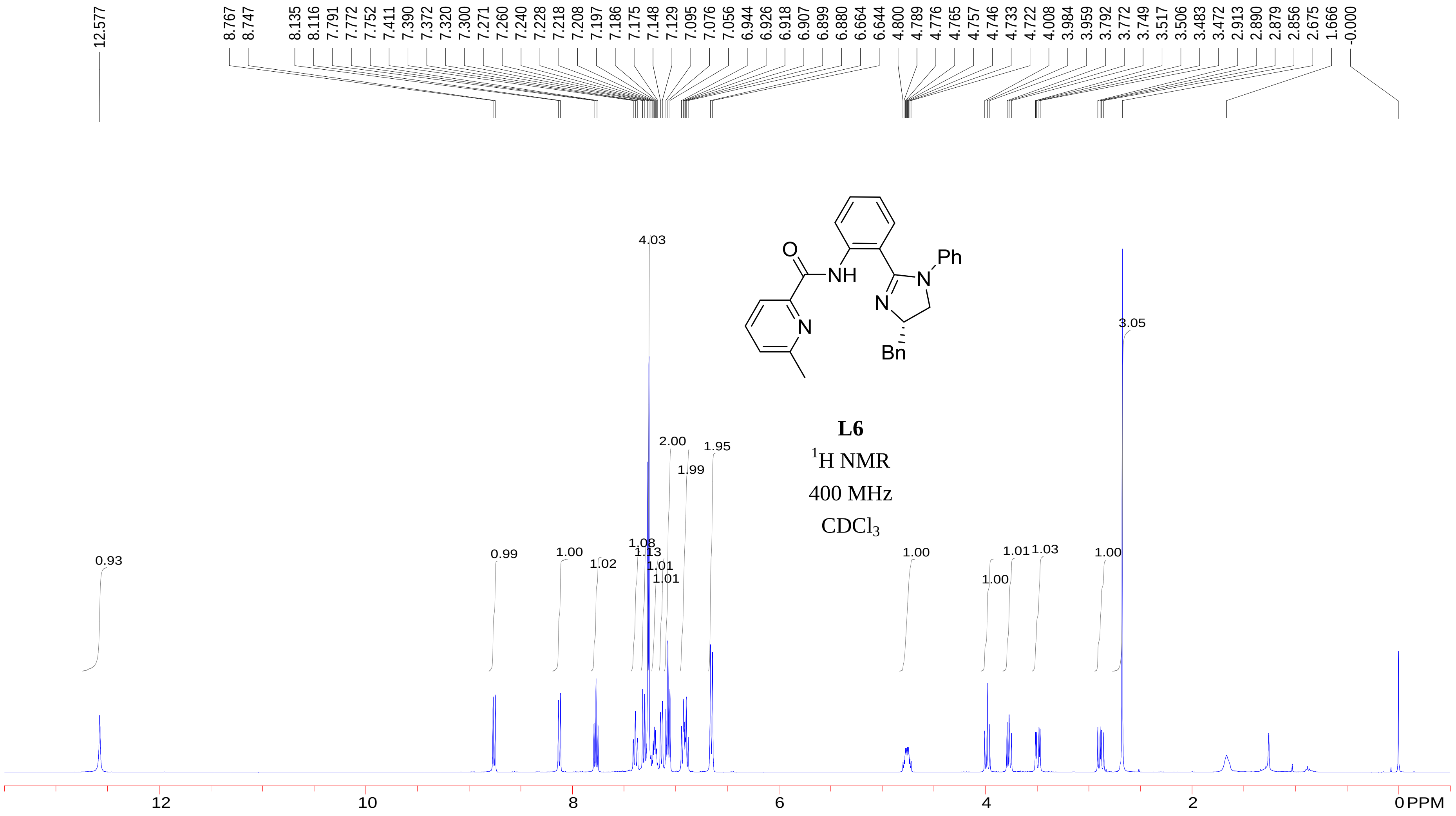

Supplementary Figure 41. ^{1}H NMR spectrum for **L6**

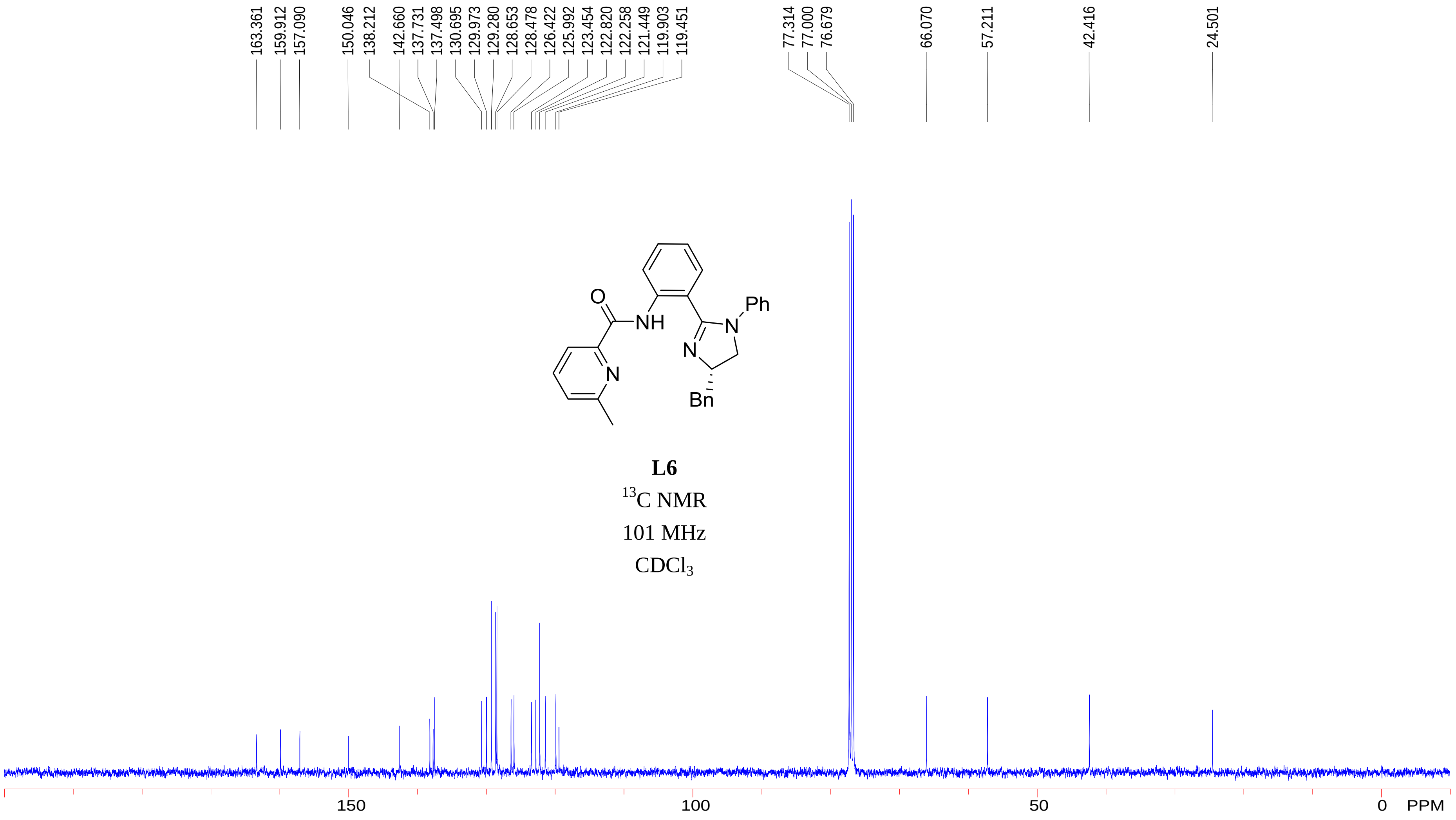

Supplementary Figure 42. ^{13}C NMR spectrum for **L6**

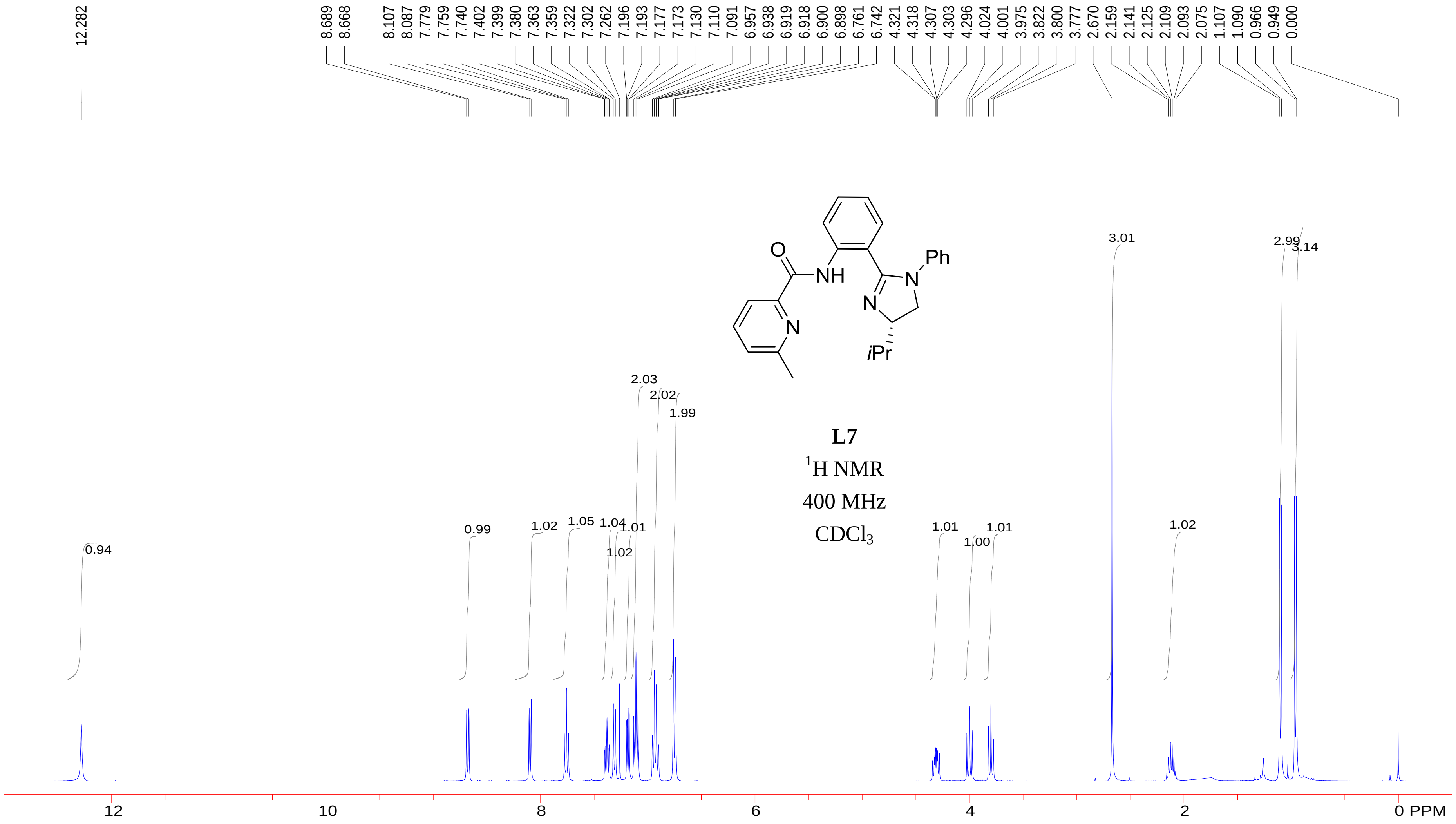

Supplementary Figure 43. ^{1}H NMR spectrum for **L7**

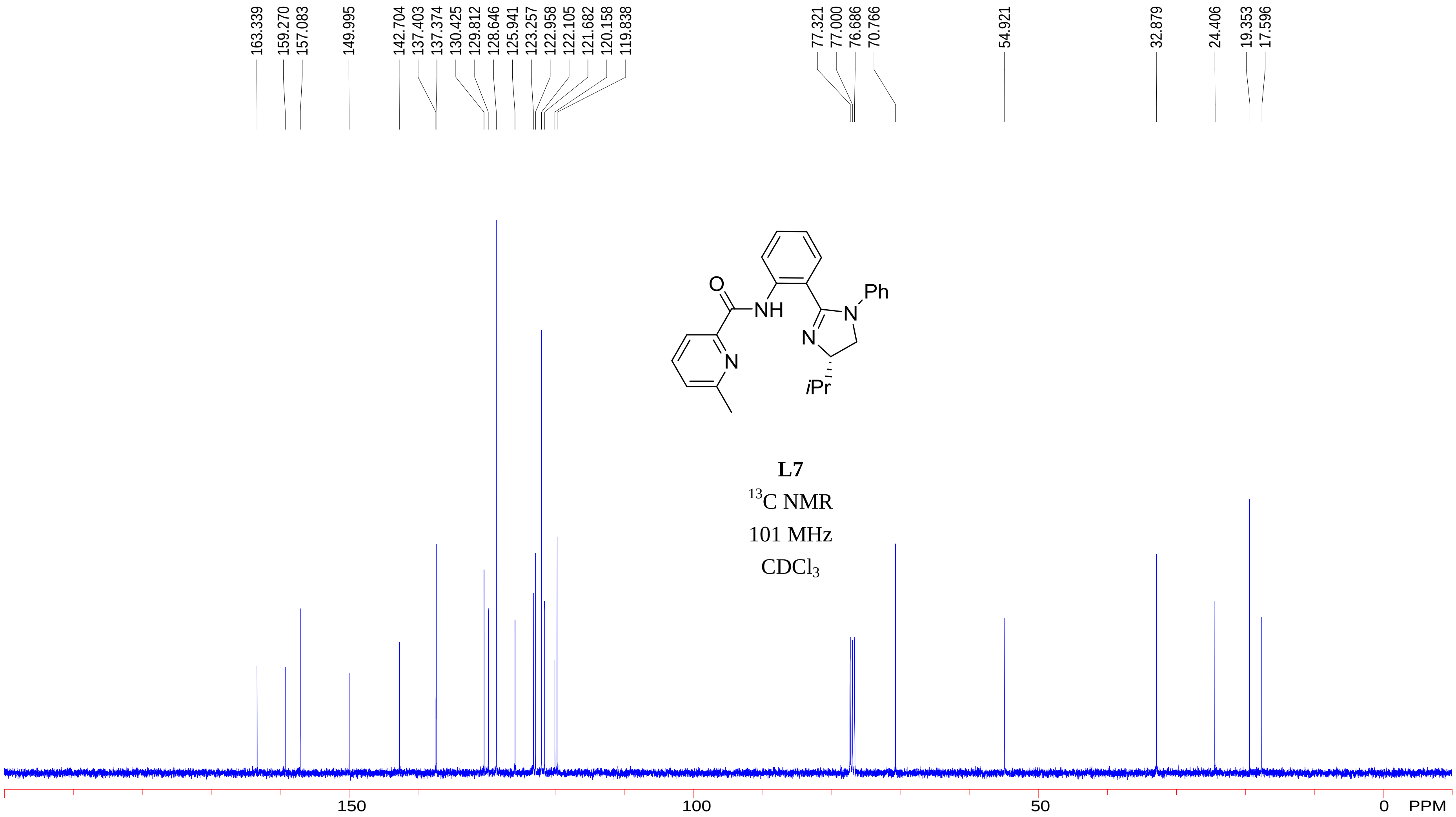

Supplementary Figure 44. ^{13}C NMR spectrum for **L7**

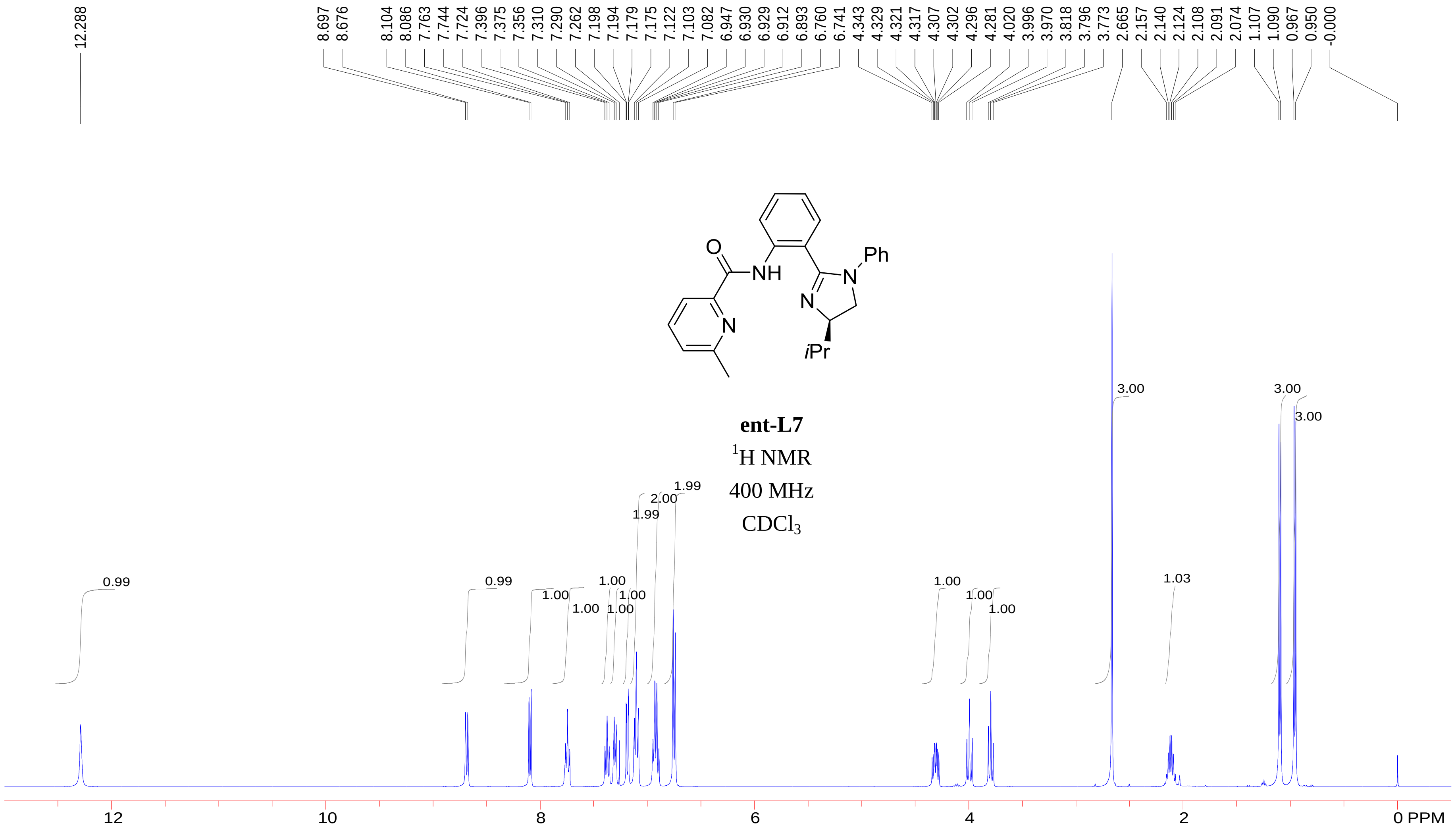

Supplementary Figure 45. ^{1}H NMR spectrum for **ent-L7**

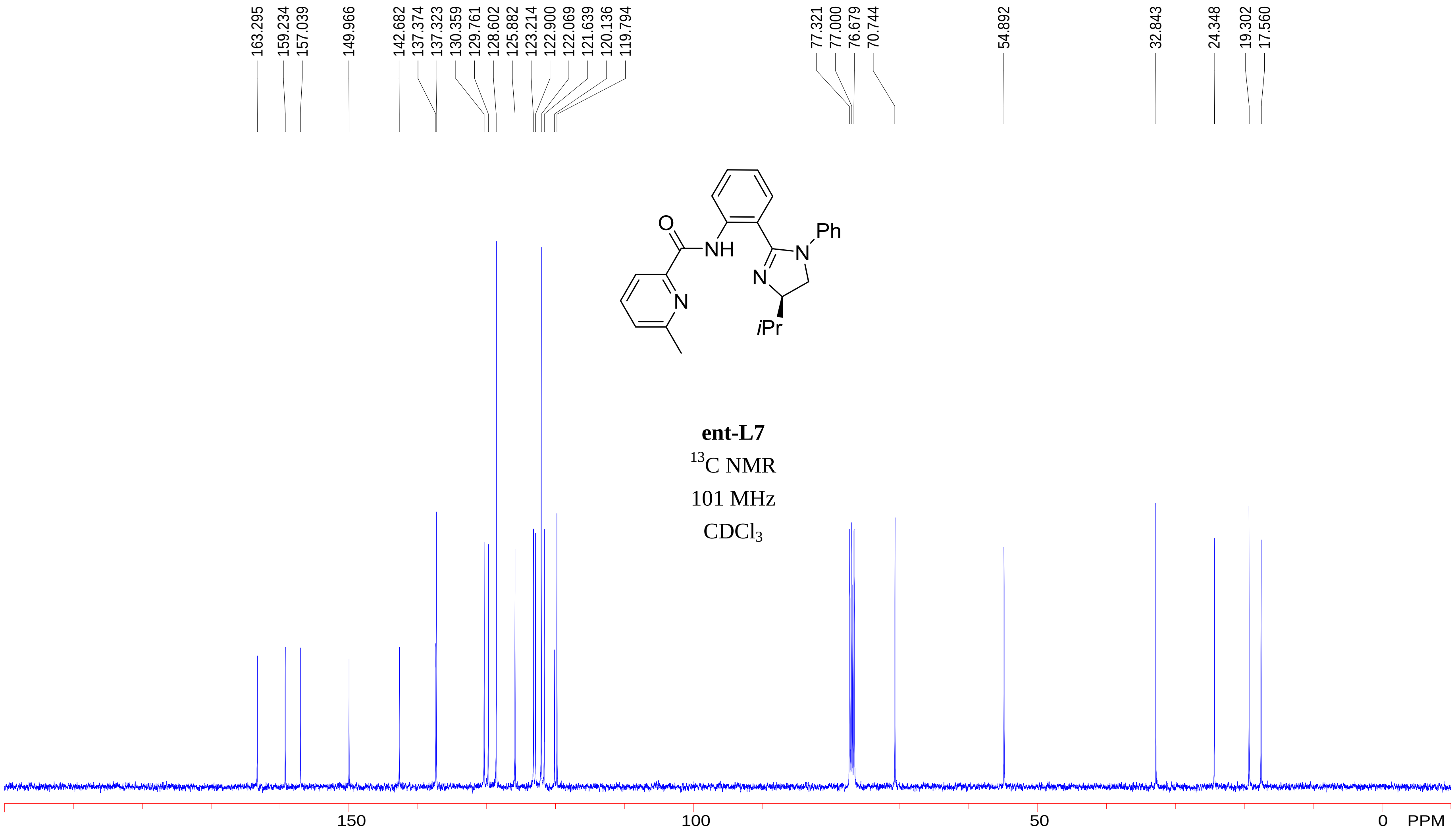

Supplementary Figure 46. ^{13}C NMR spectrum for **ent-L7**

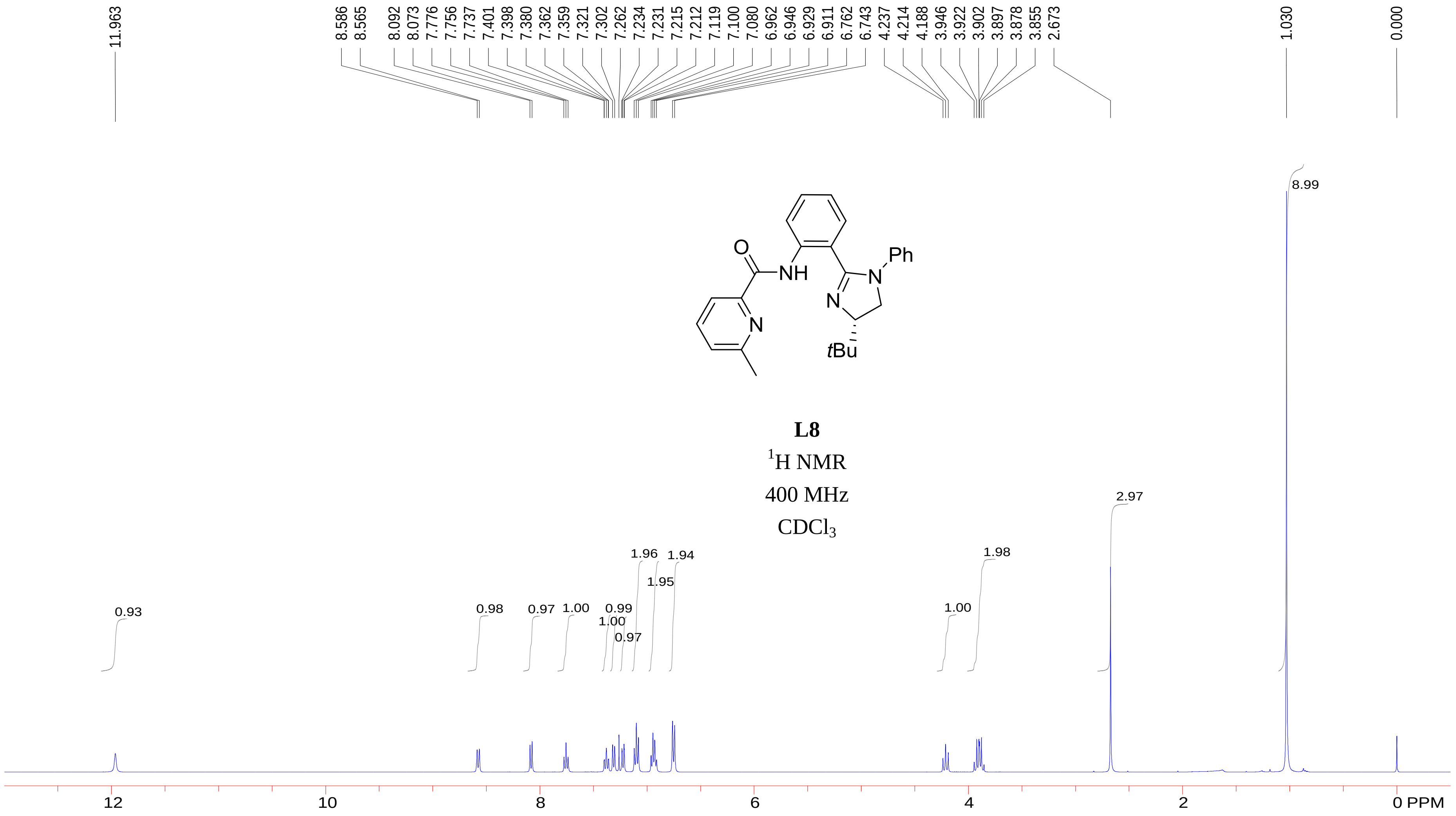

Supplementary Figure 47. ^{1}H NMR spectrum for **L8**

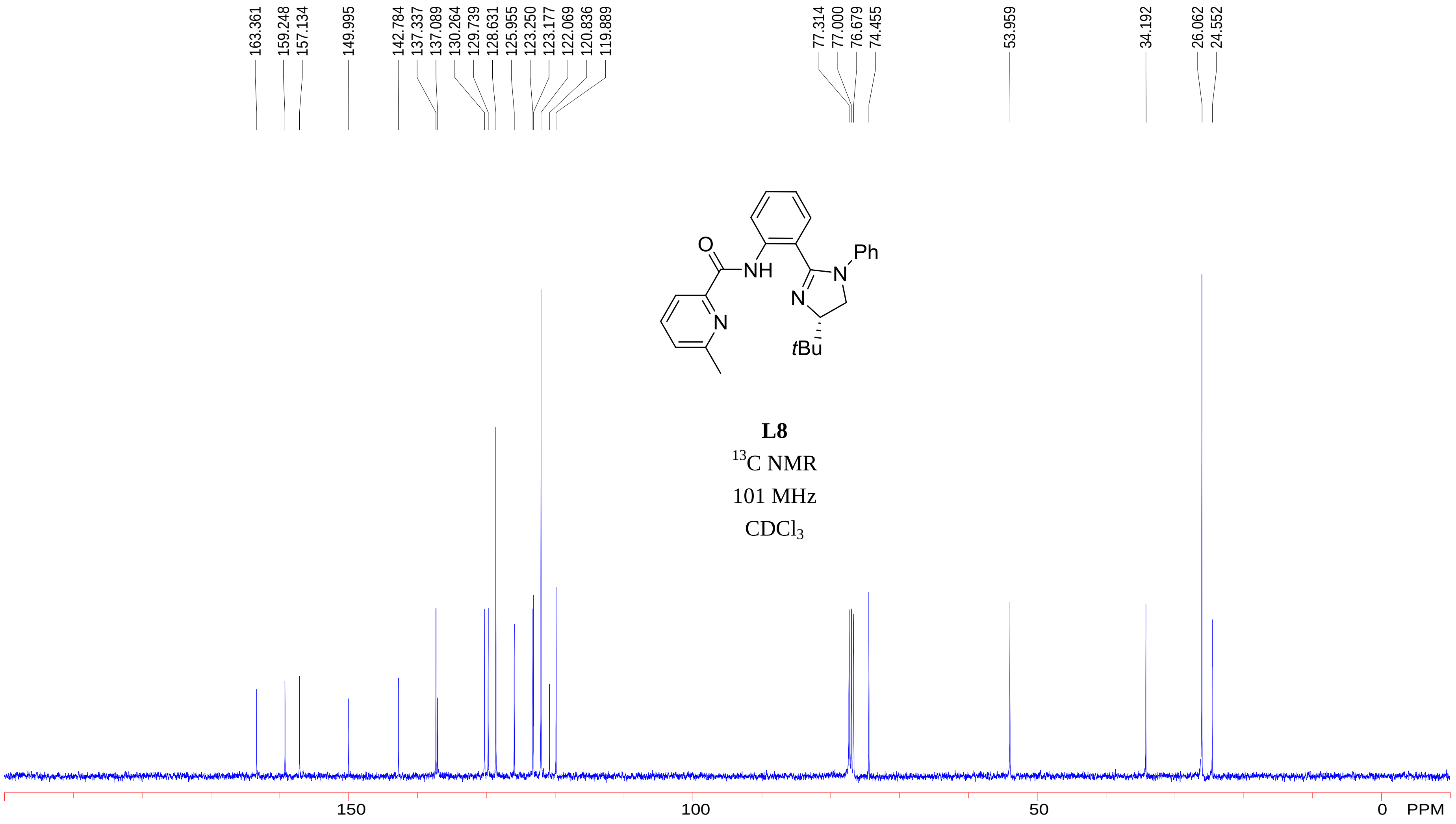

Supplementary Figure 48. ^{13}C NMR spectrum for **L8**

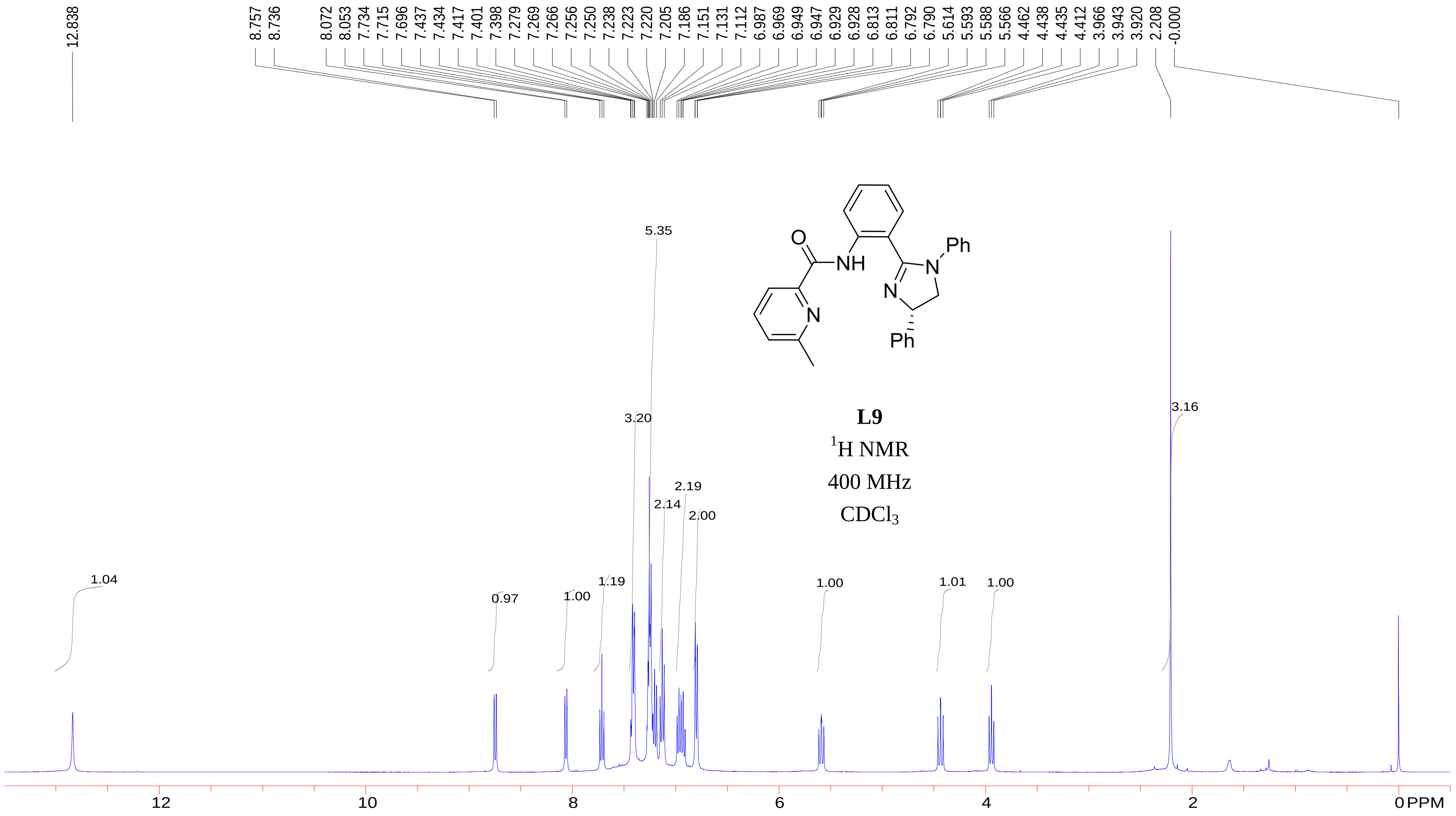

Supplementary Figure 49. ^{1}H NMR spectrum for **L9**

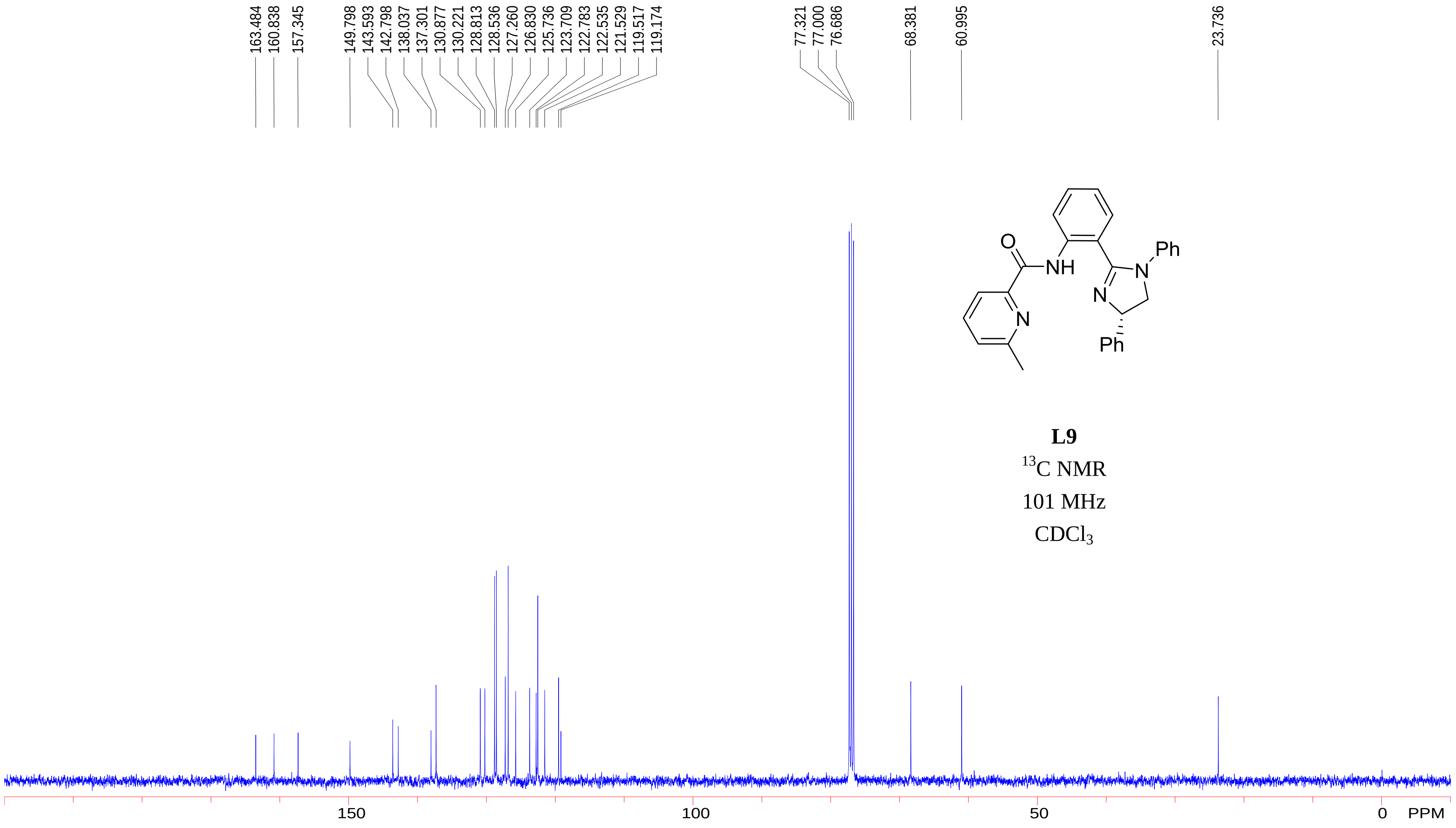

Supplementary Figure 50. ^{13}C NMR spectrum for **L9**

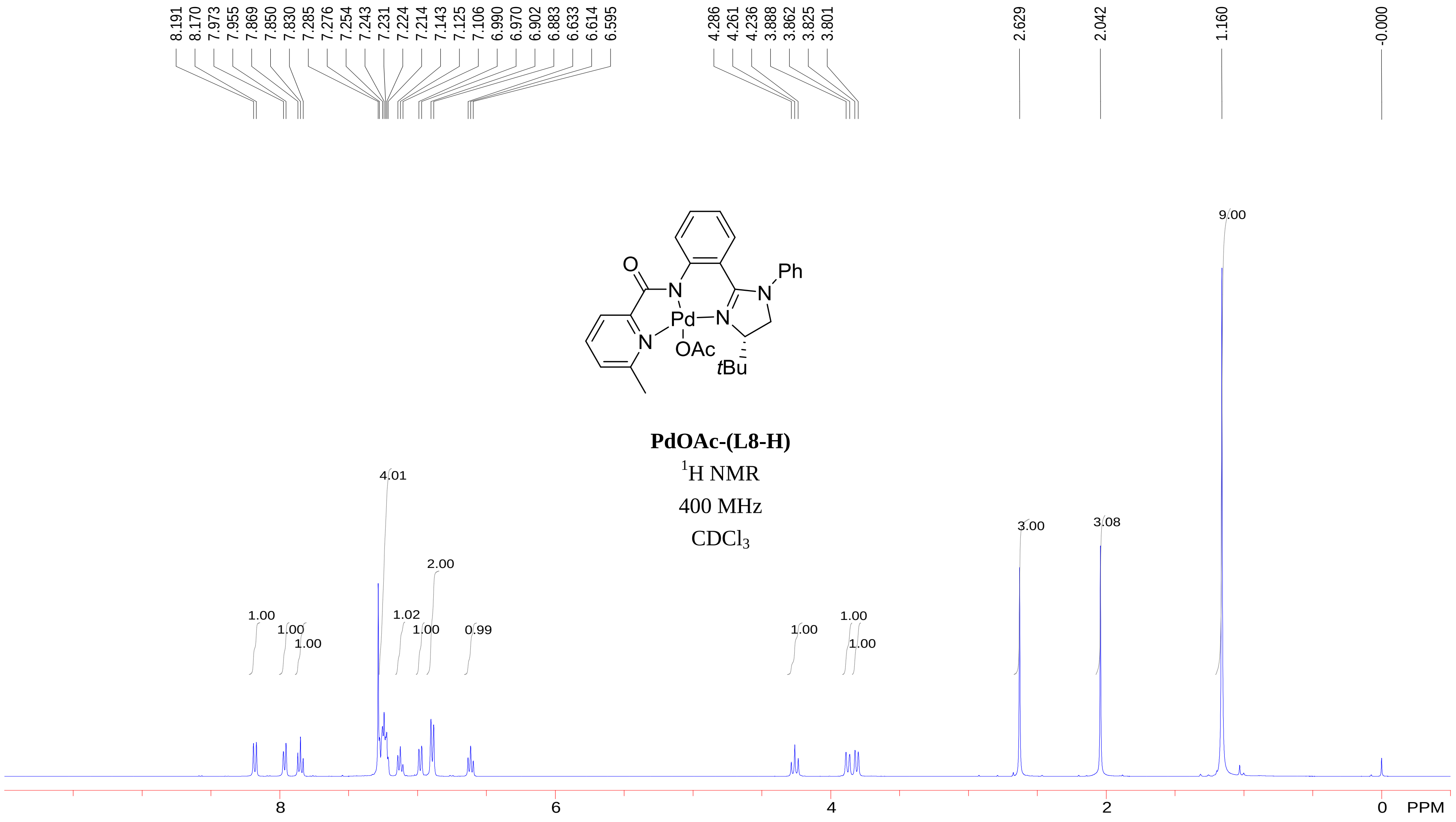

Supplementary Figure 51. ^{1}H NMR spectrum for **PdOAc-(L8-H)**

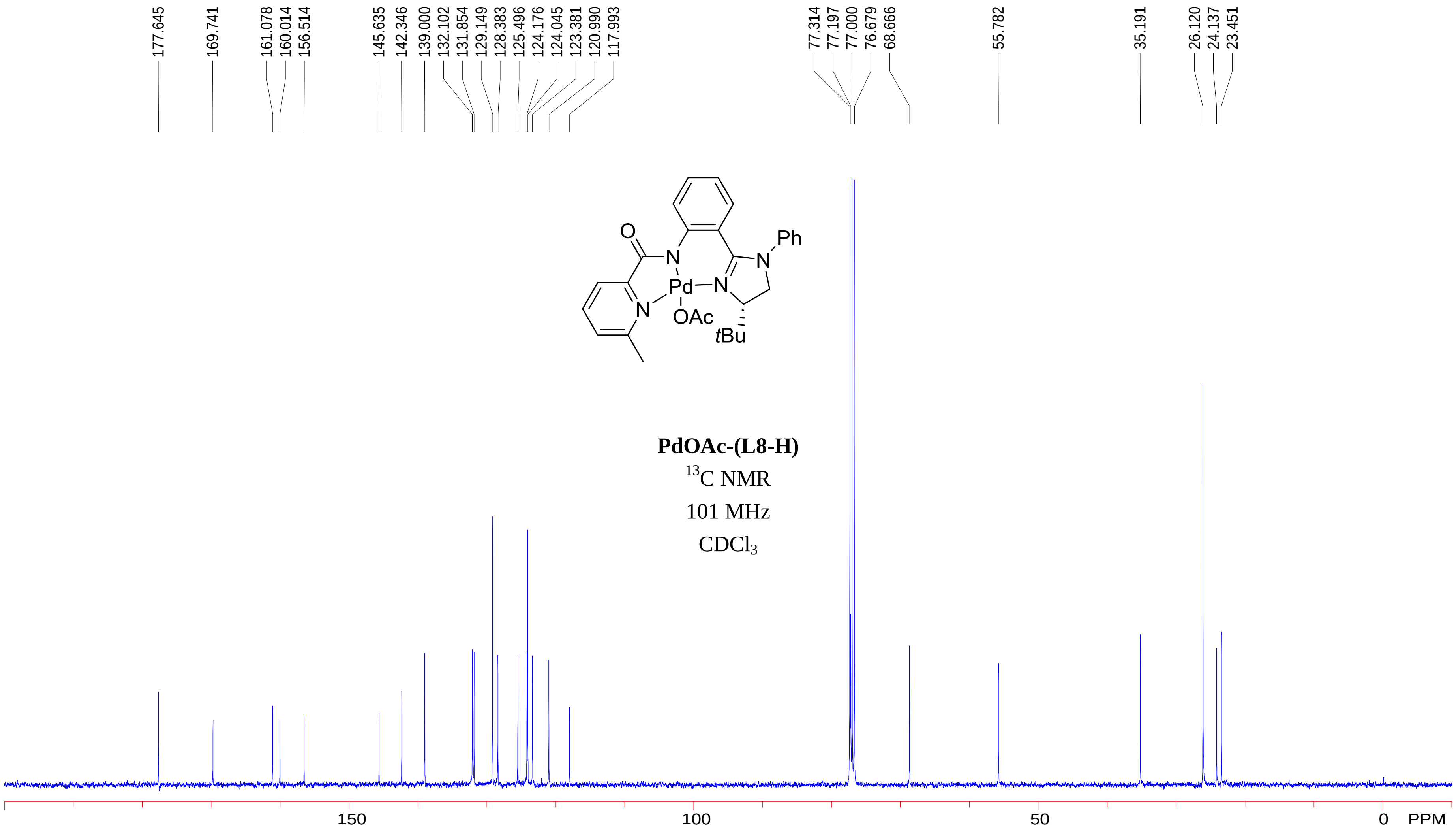

Supplementary Figure 52. ^{13}C NMR spectrum for **PdOAc-(L8-H)**

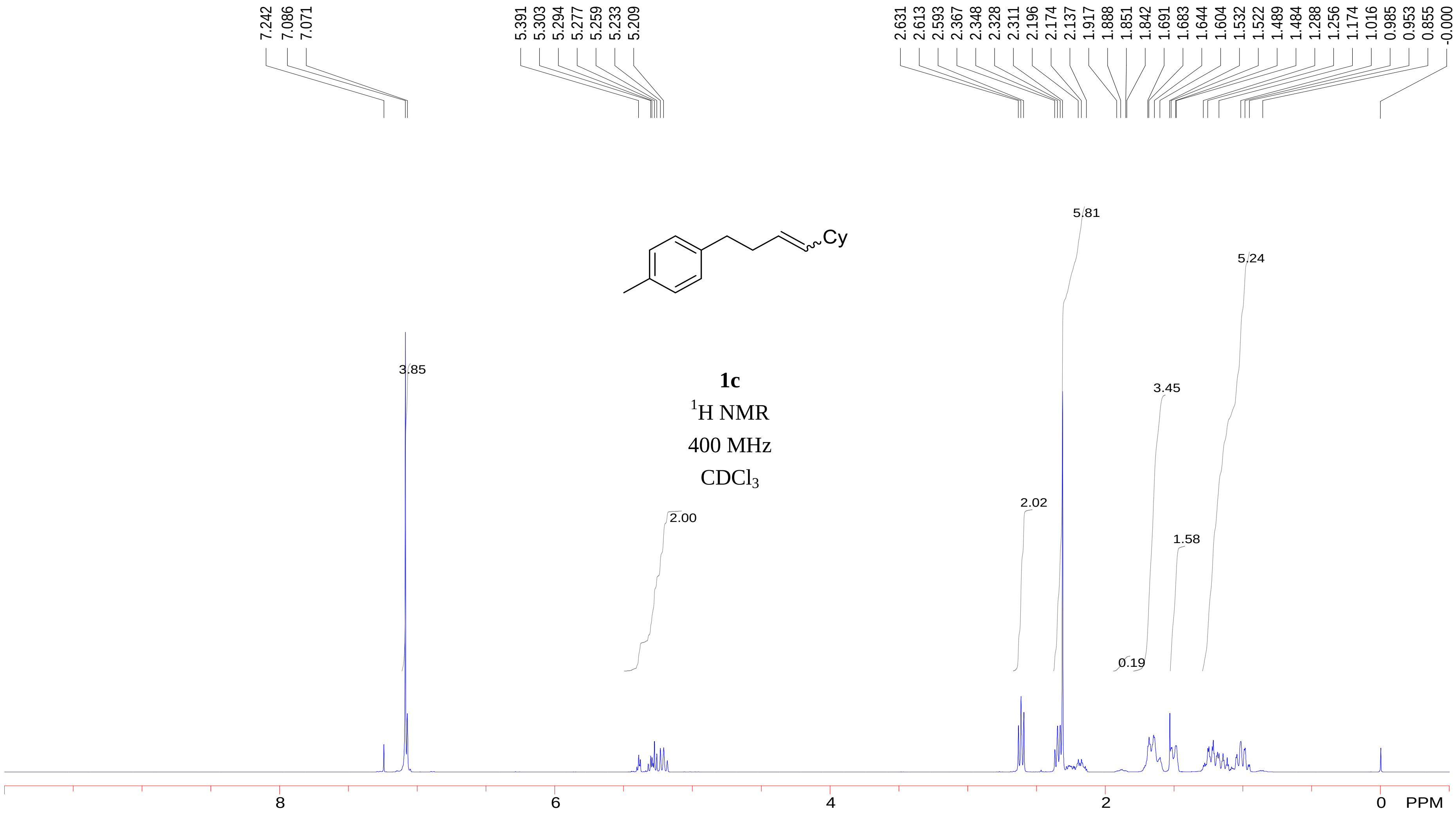

Supplementary Figure 53. ^{1}H NMR spectrum for **1c**

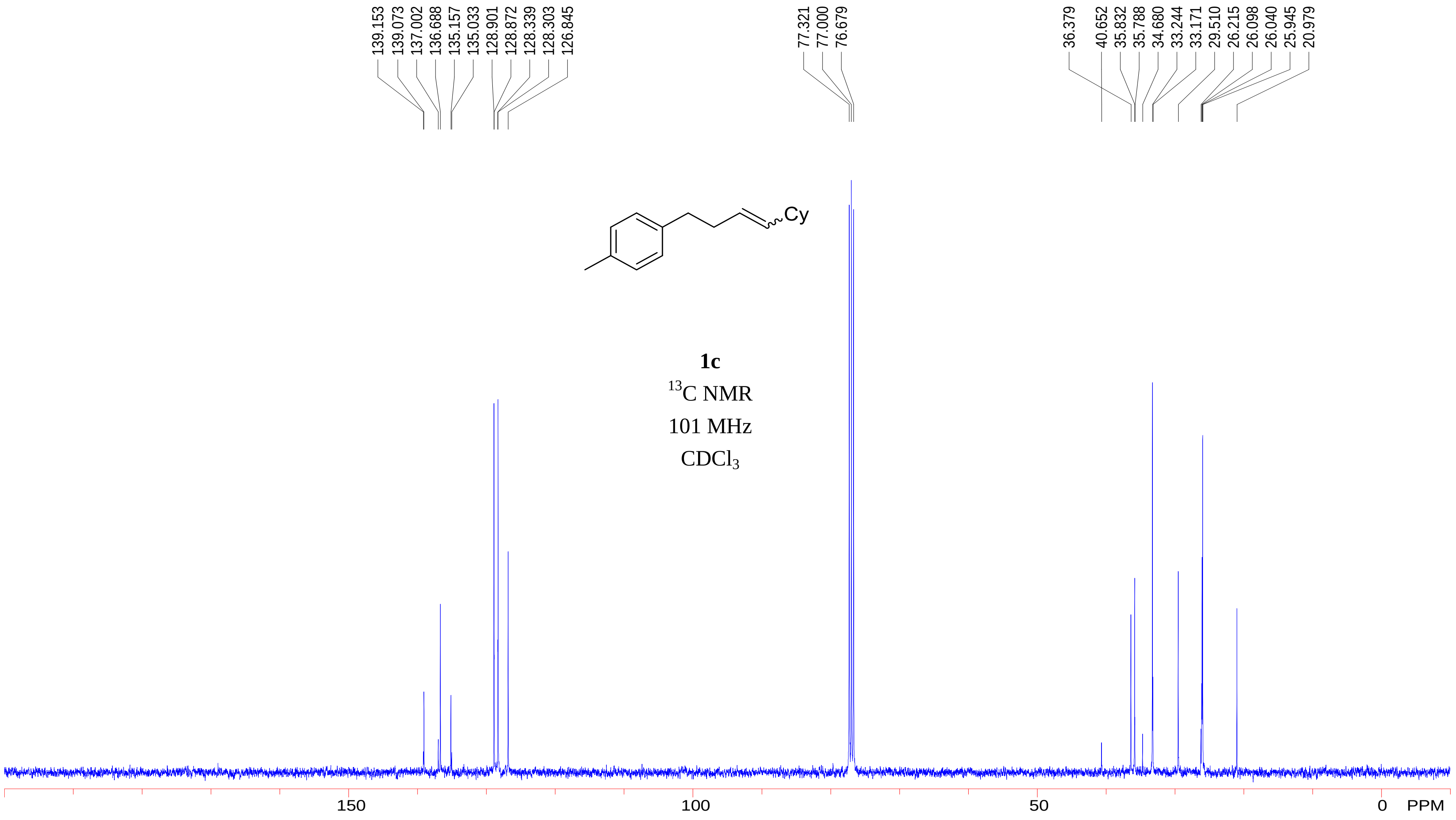

Supplementary Figure 54. ^{13}C NMR spectrum for **1c**

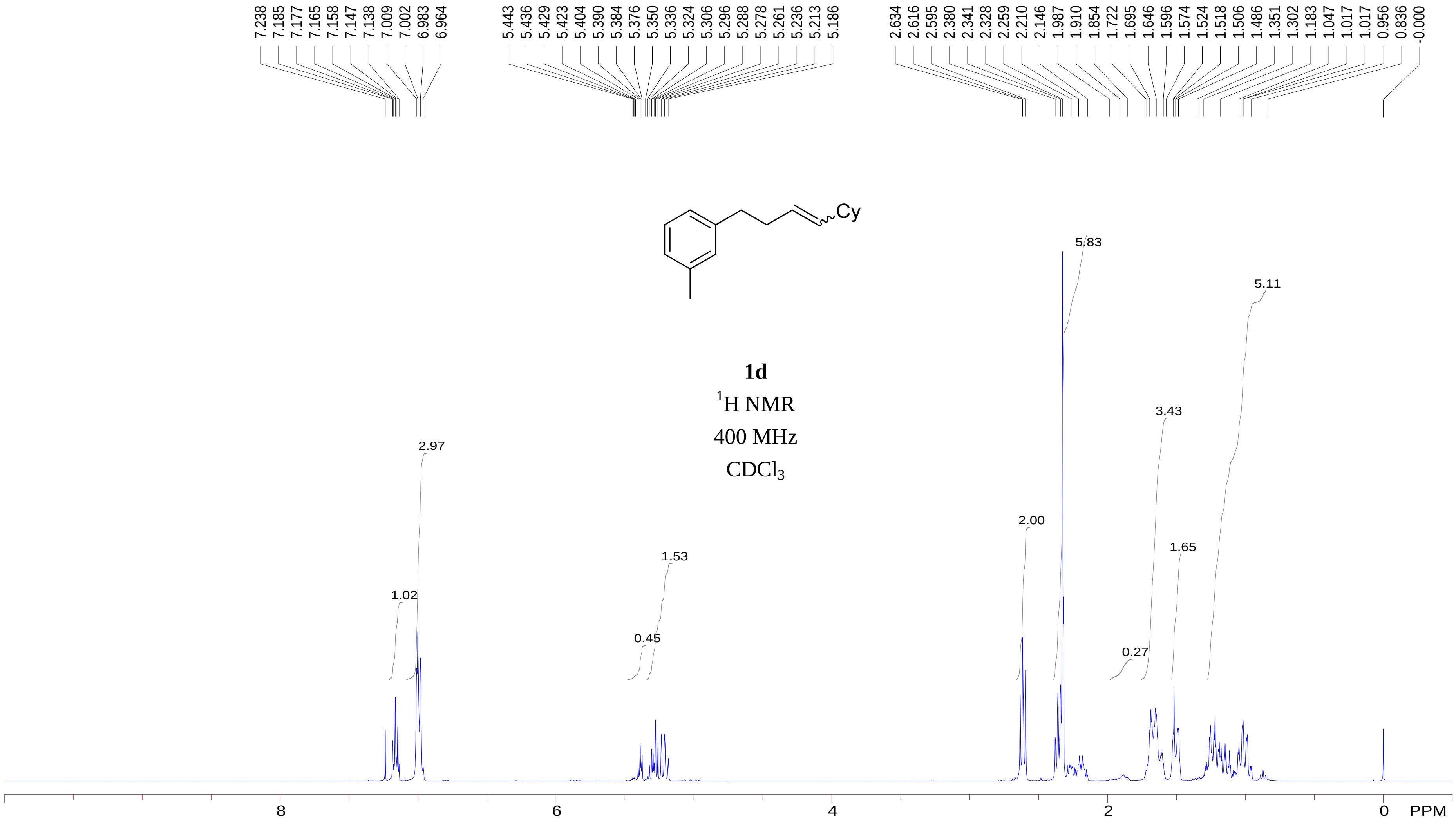

Supplementary Figure 55. ^{1}H NMR spectrum for **1d**

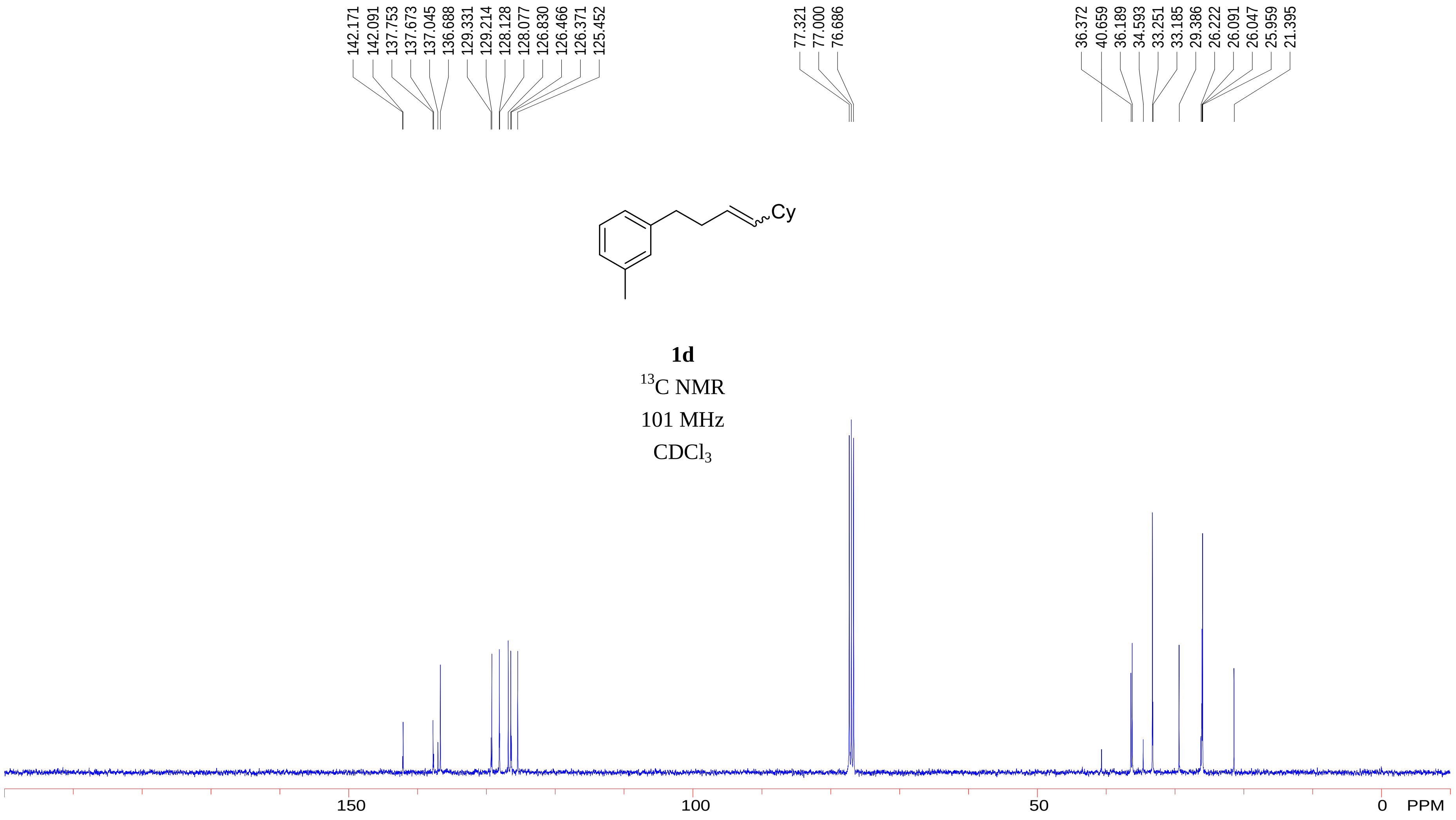

Supplementary Figure 56. ^{13}C NMR spectrum for **1d**

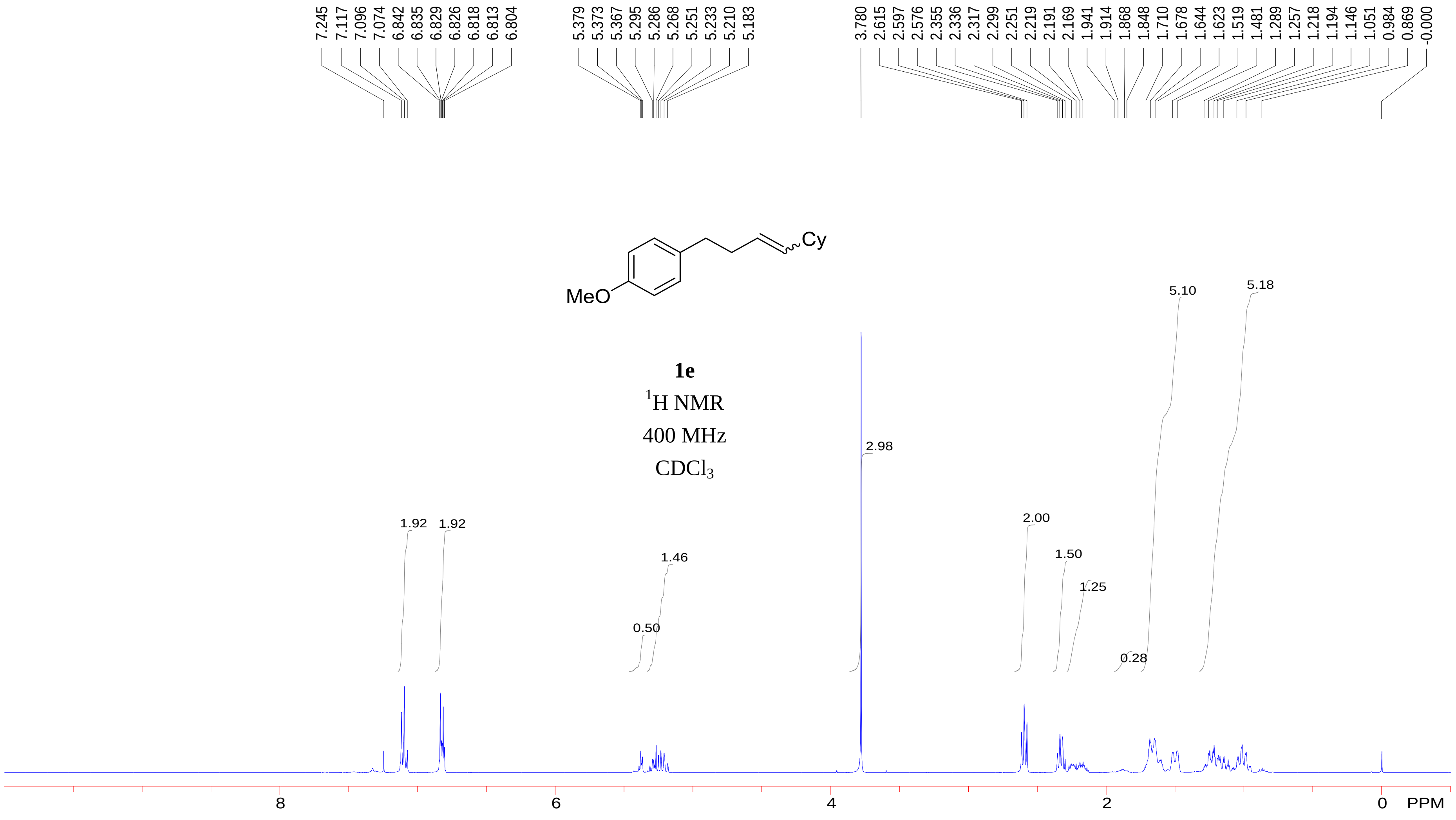

Supplementary Figure 57. ^{1}H NMR spectrum for **1e**

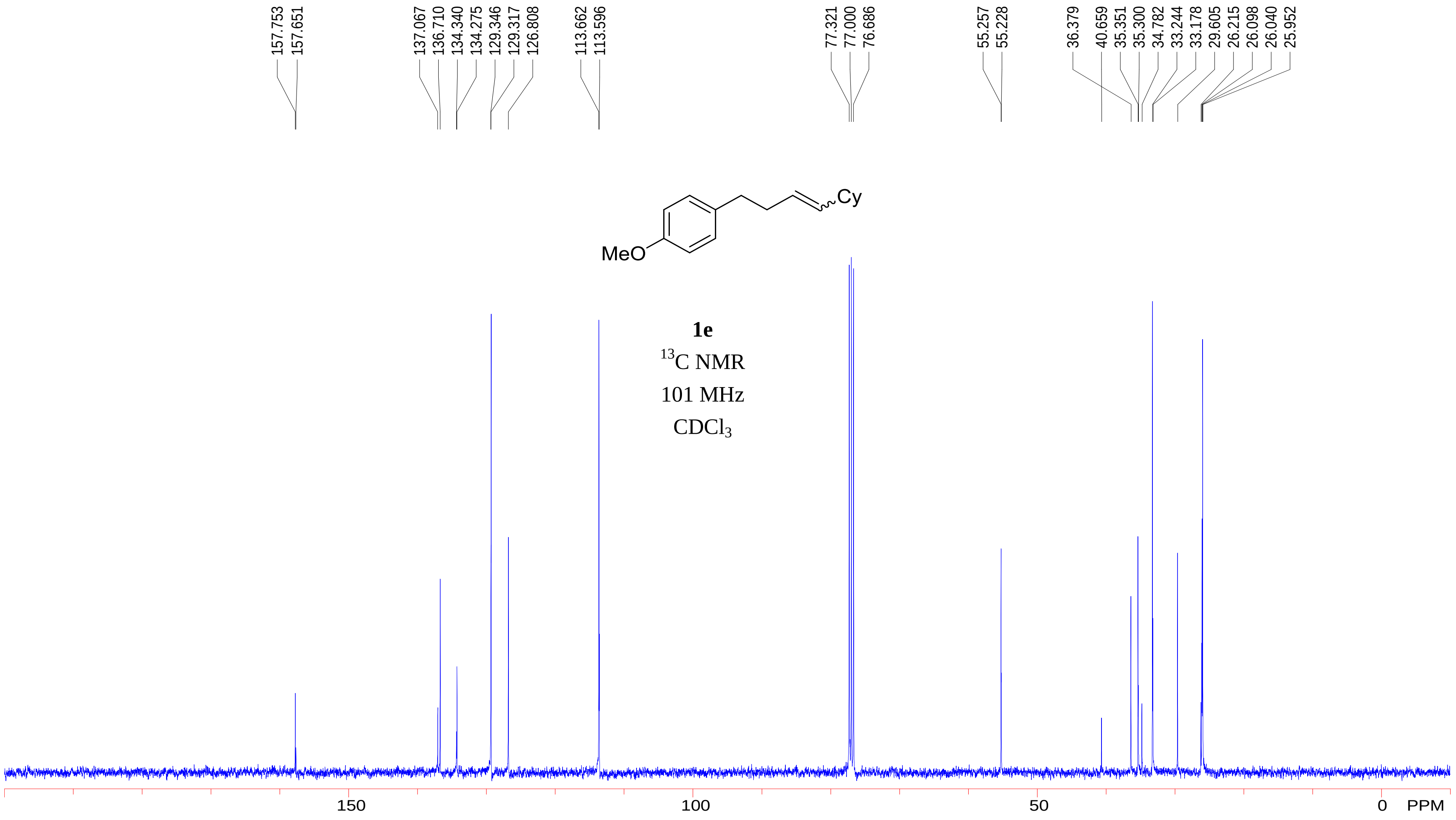

Supplementary Figure 58. ^{13}C NMR spectrum for **1e**

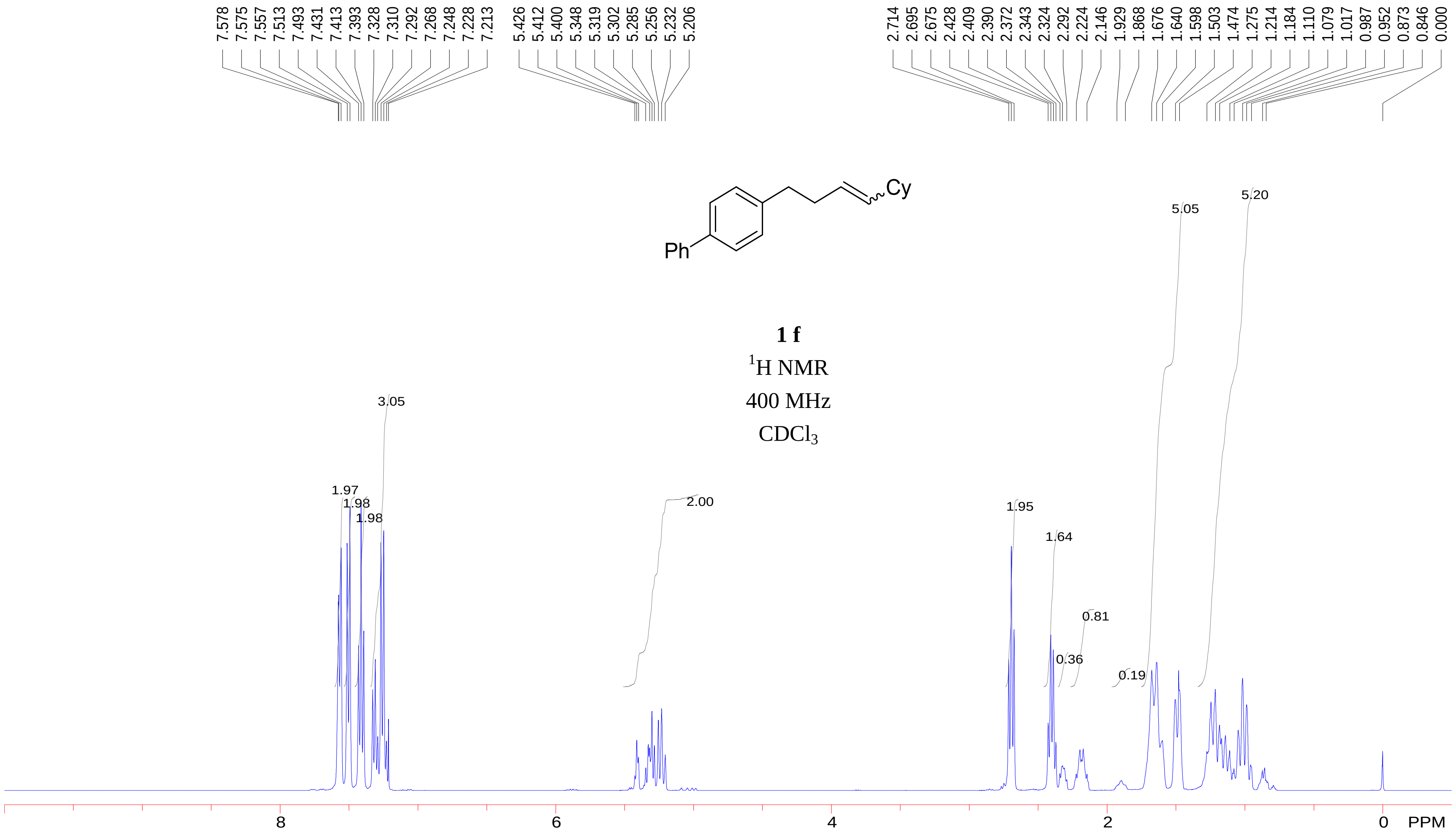

Supplementary Figure 59. ^{1}H NMR spectrum for **1f**

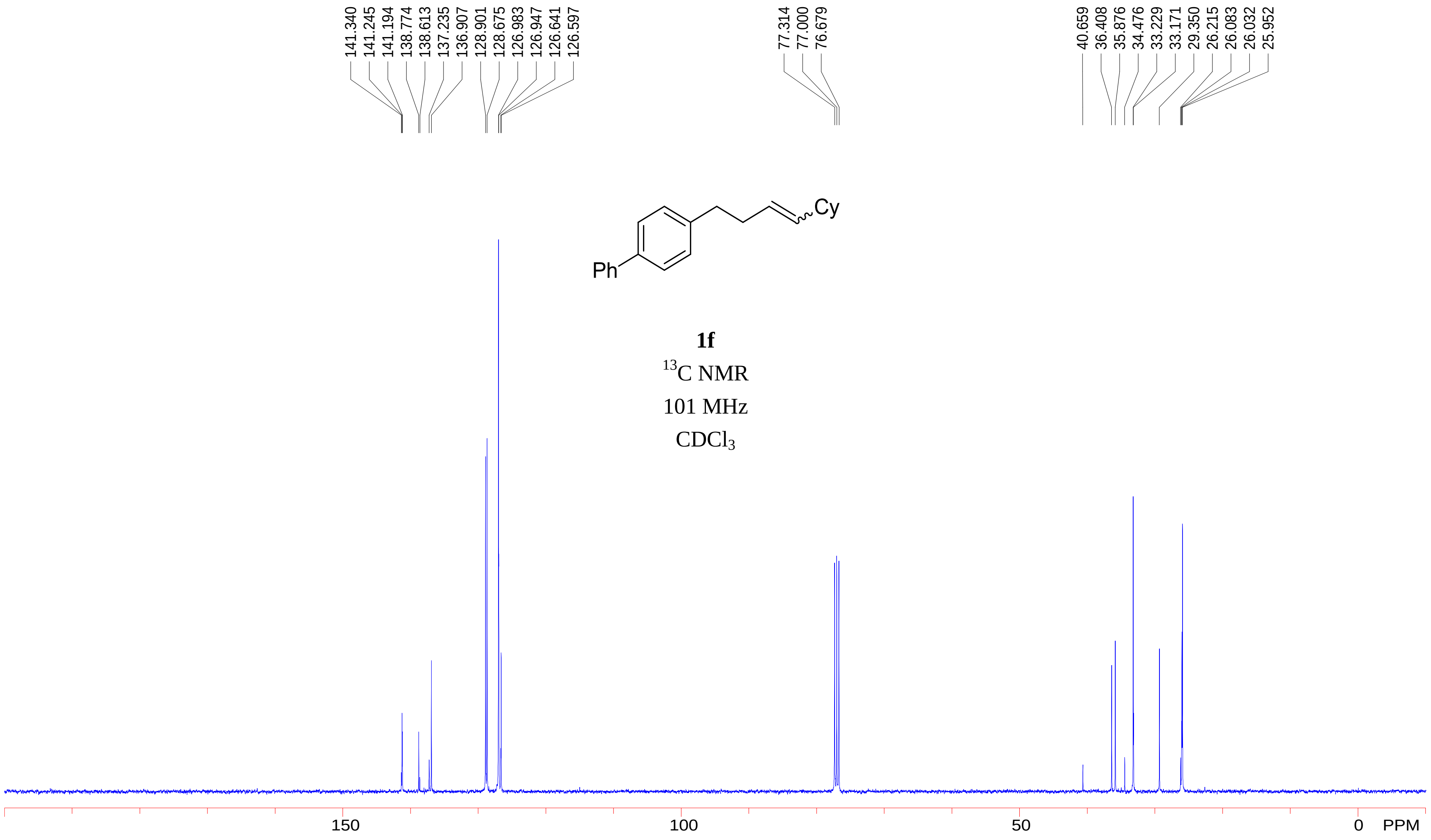

Supplementary Figure 60. ^{13}C NMR spectrum for **1f**

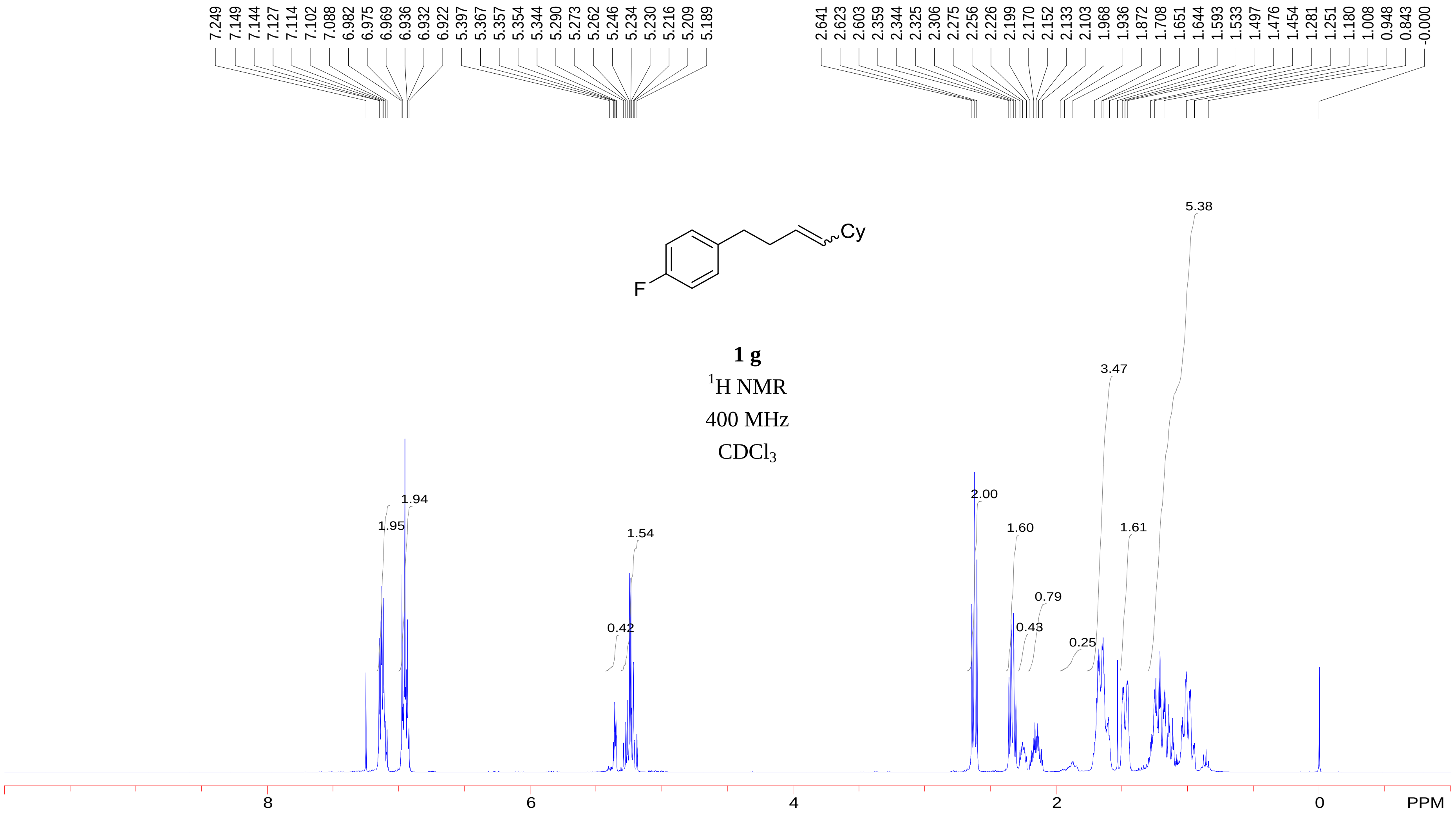

Supplementary Figure 61. ^{1}H NMR spectrum for **1g**

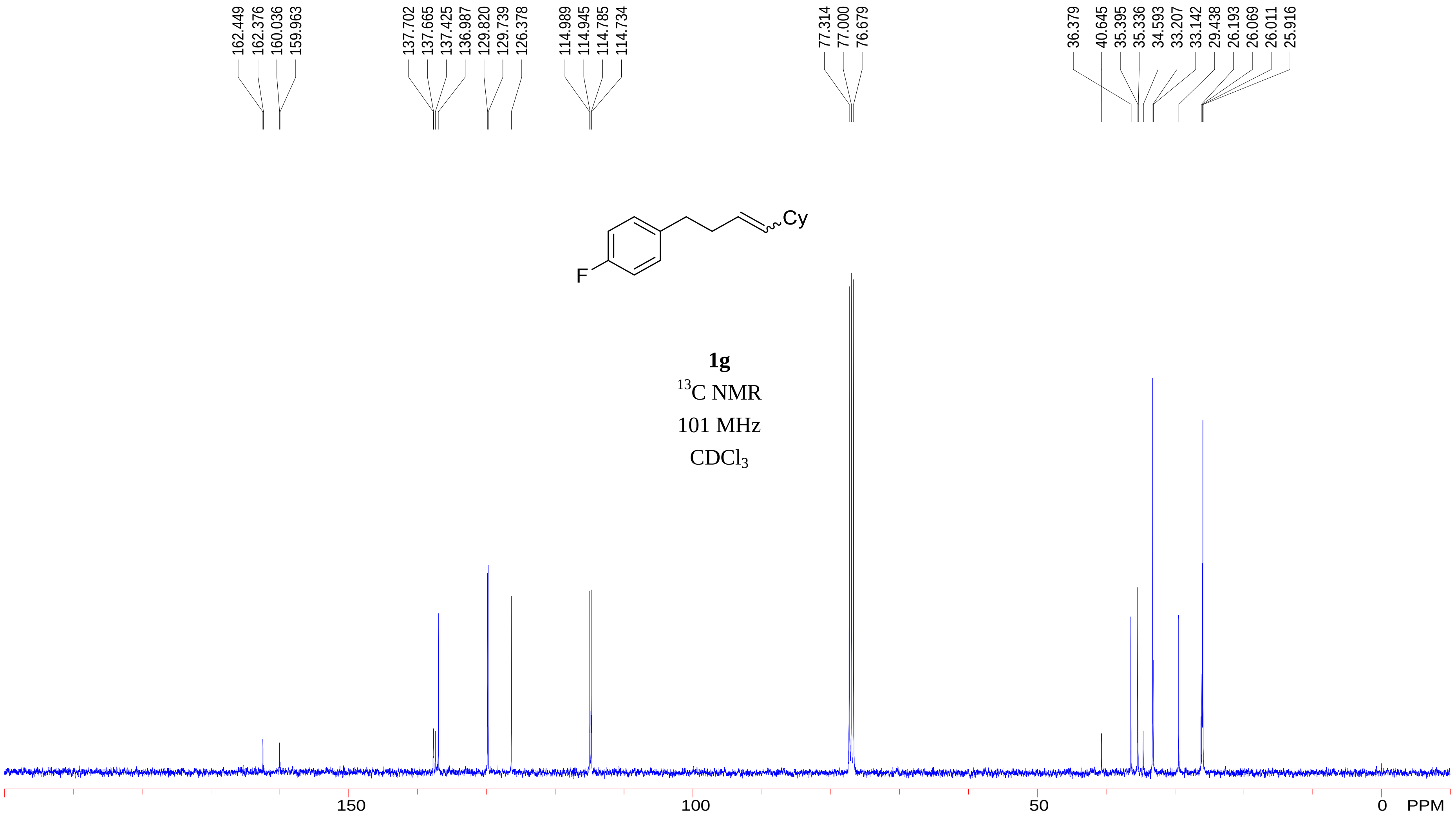

Supplementary Figure 62. ^{13}C NMR spectrum for **1g**

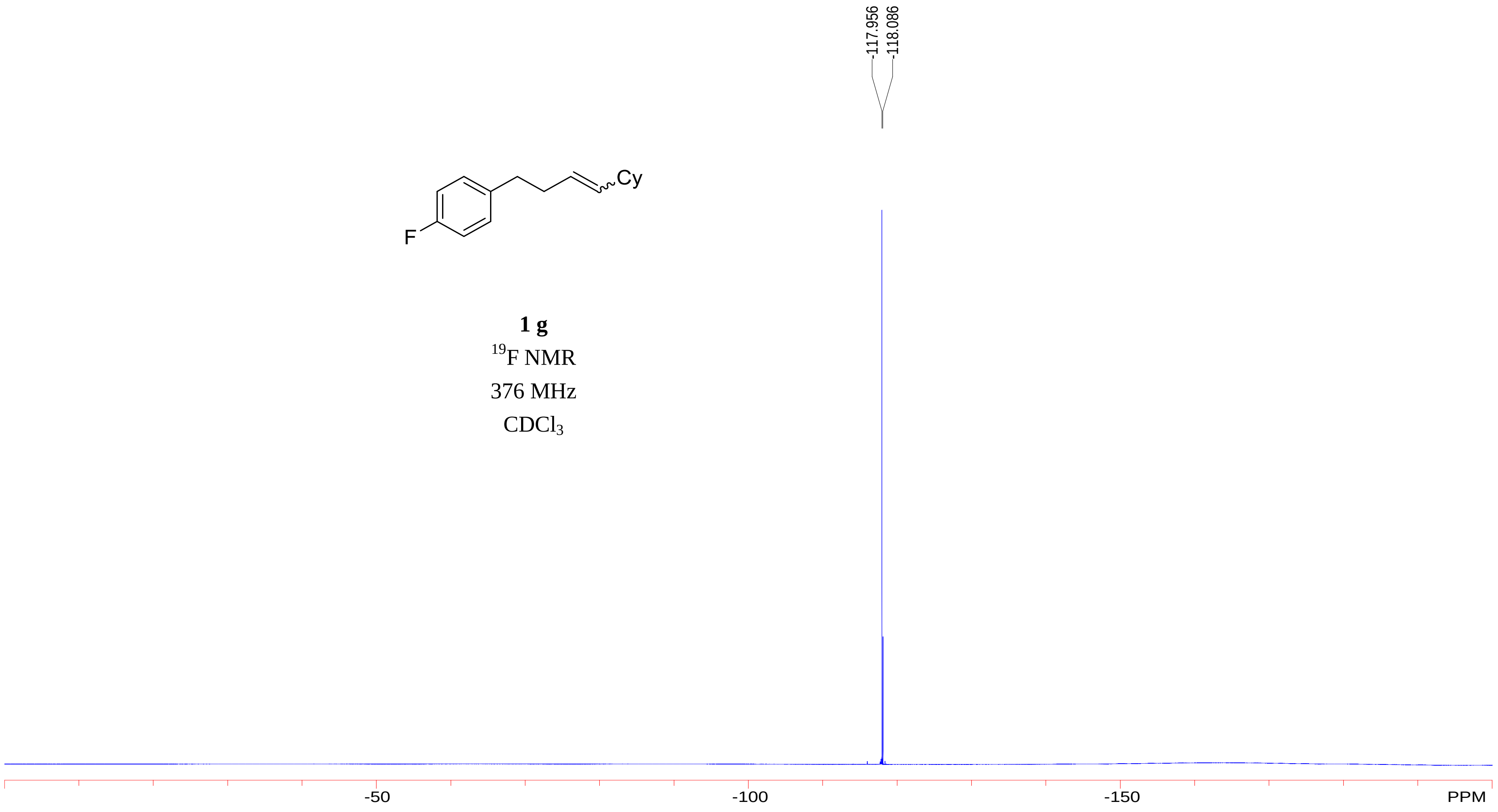

Supplementary Figure 63. ^{19}F NMR spectrum for **1g**

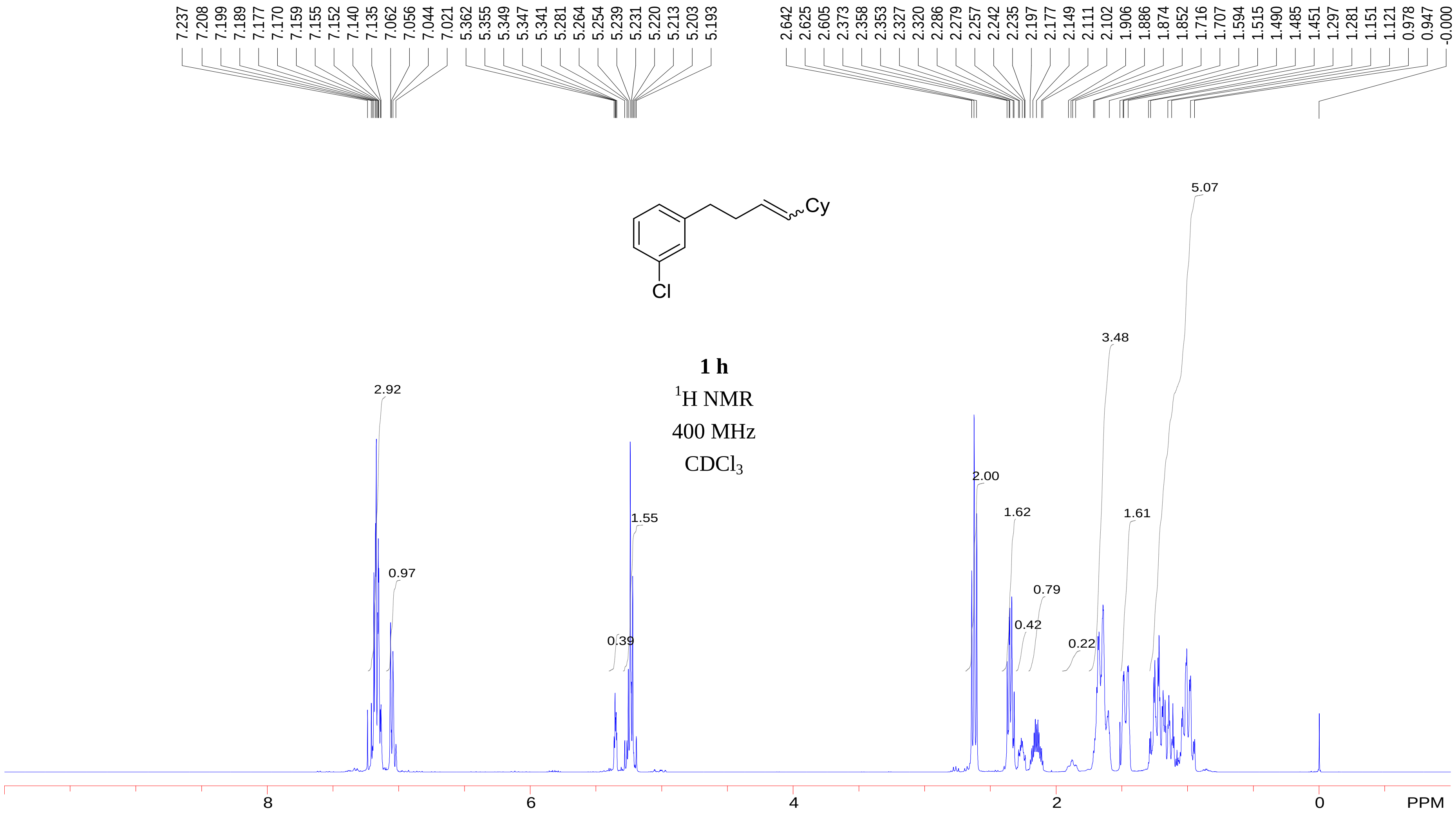

Supplementary Figure 64. ^{1}H NMR spectrum for **1h**

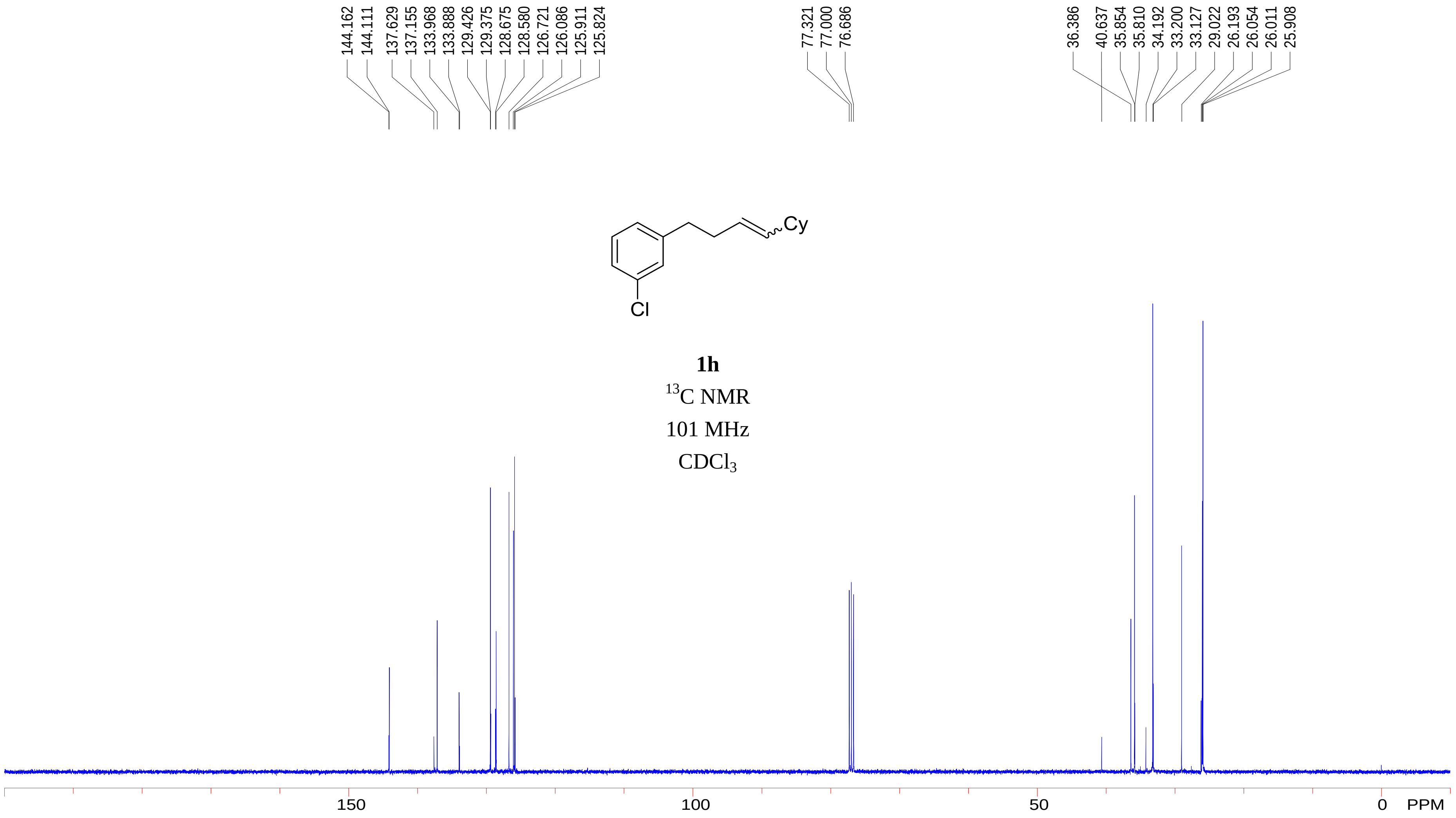

Supplementary Figure 65. ^{13}C NMR spectrum for **1h**

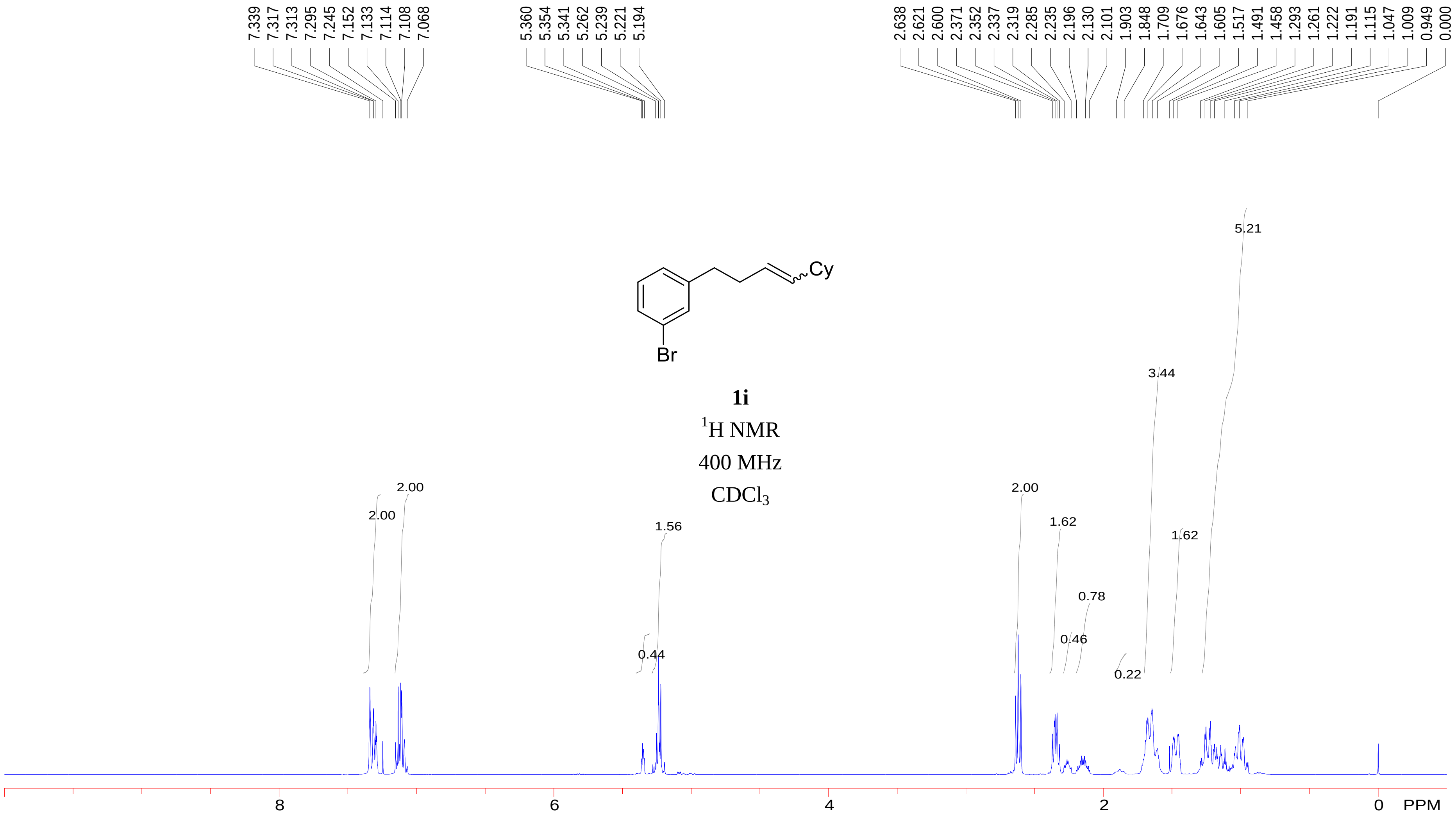

Supplementary Figure 66. ^{1}H NMR spectrum for **1i**

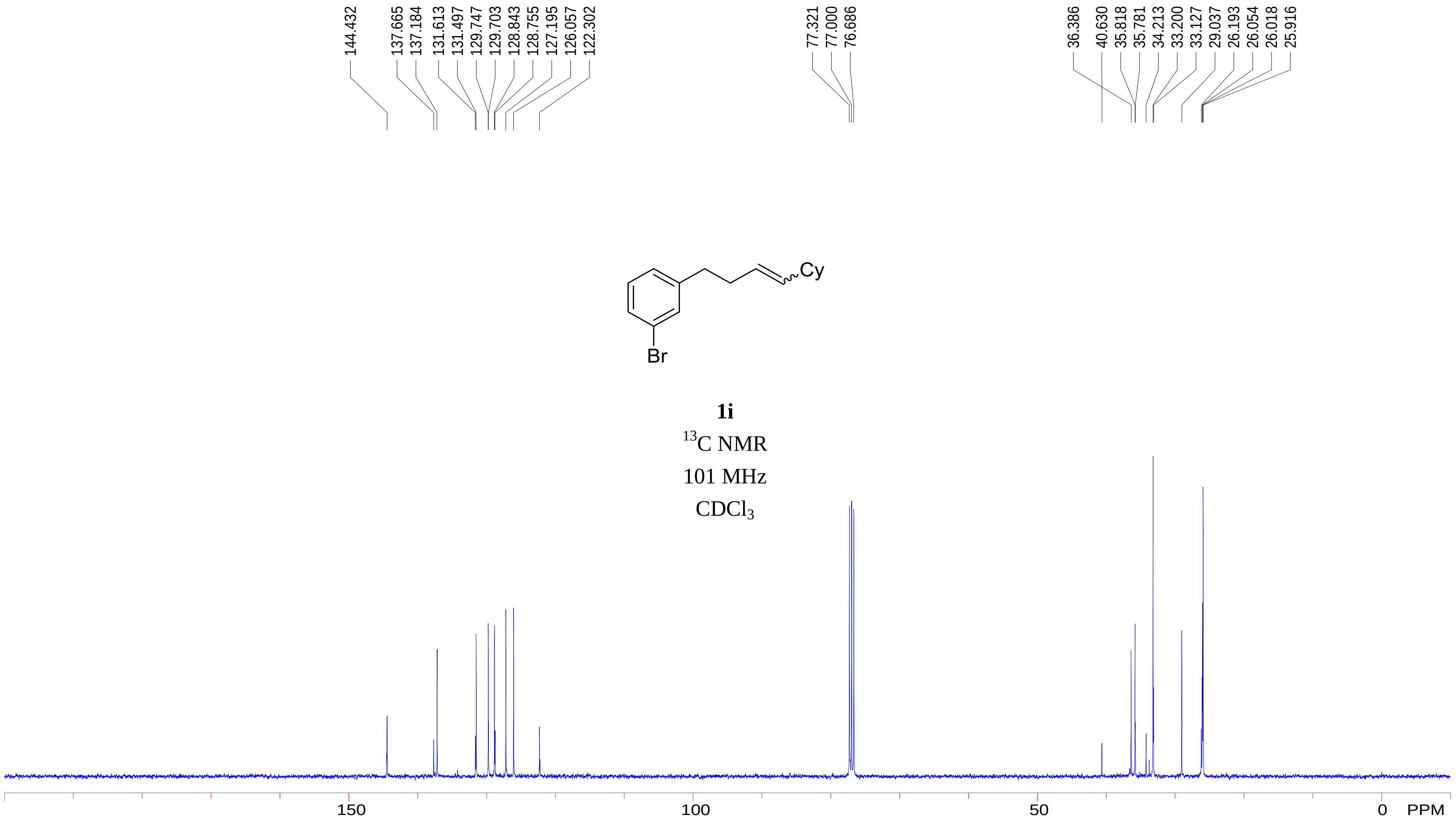

Supplementary Figure 67. ^{13}C NMR spectrum for **1i**

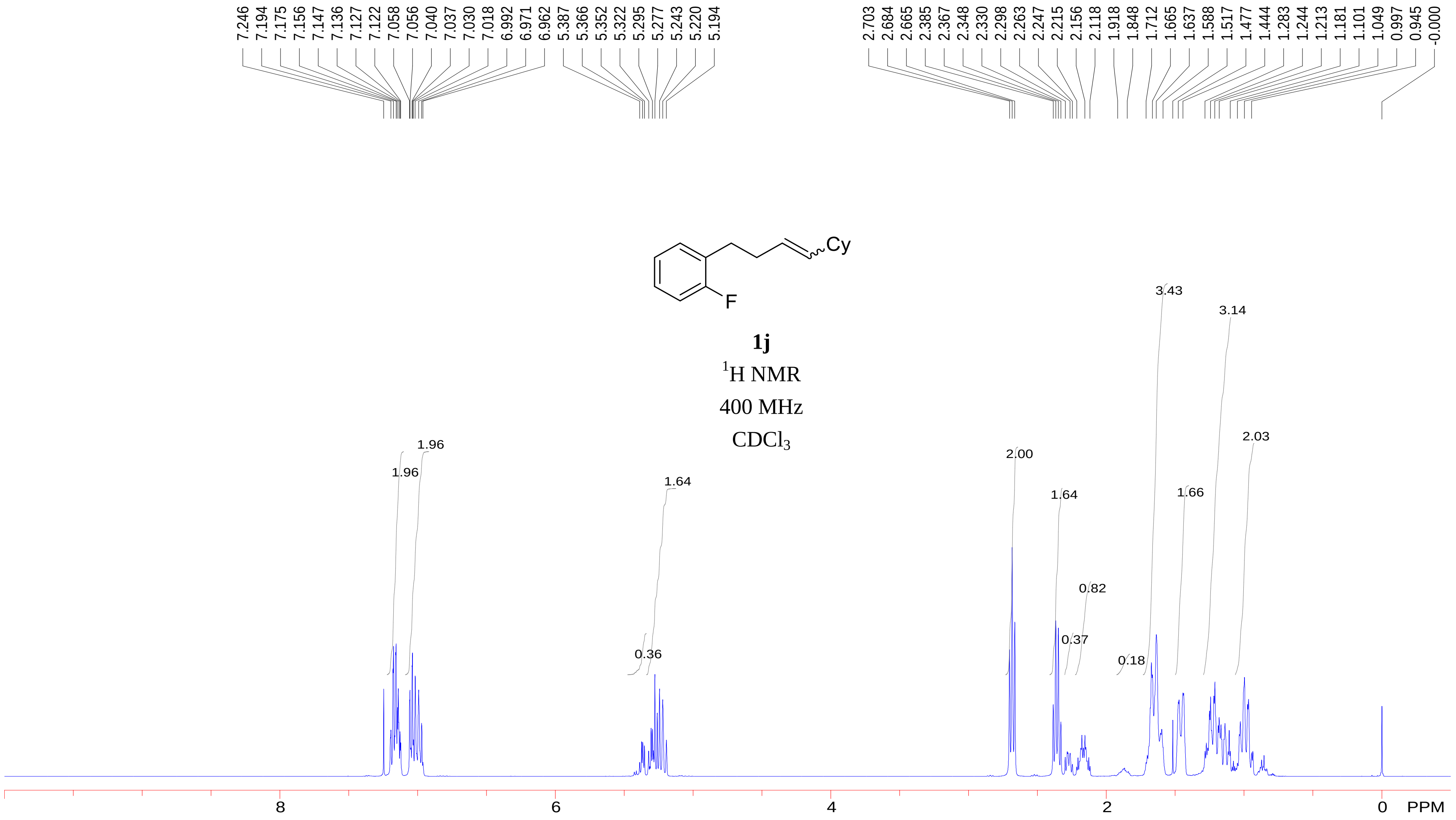

Supplementary Figure 68. ^{1}H NMR spectrum for **1j**

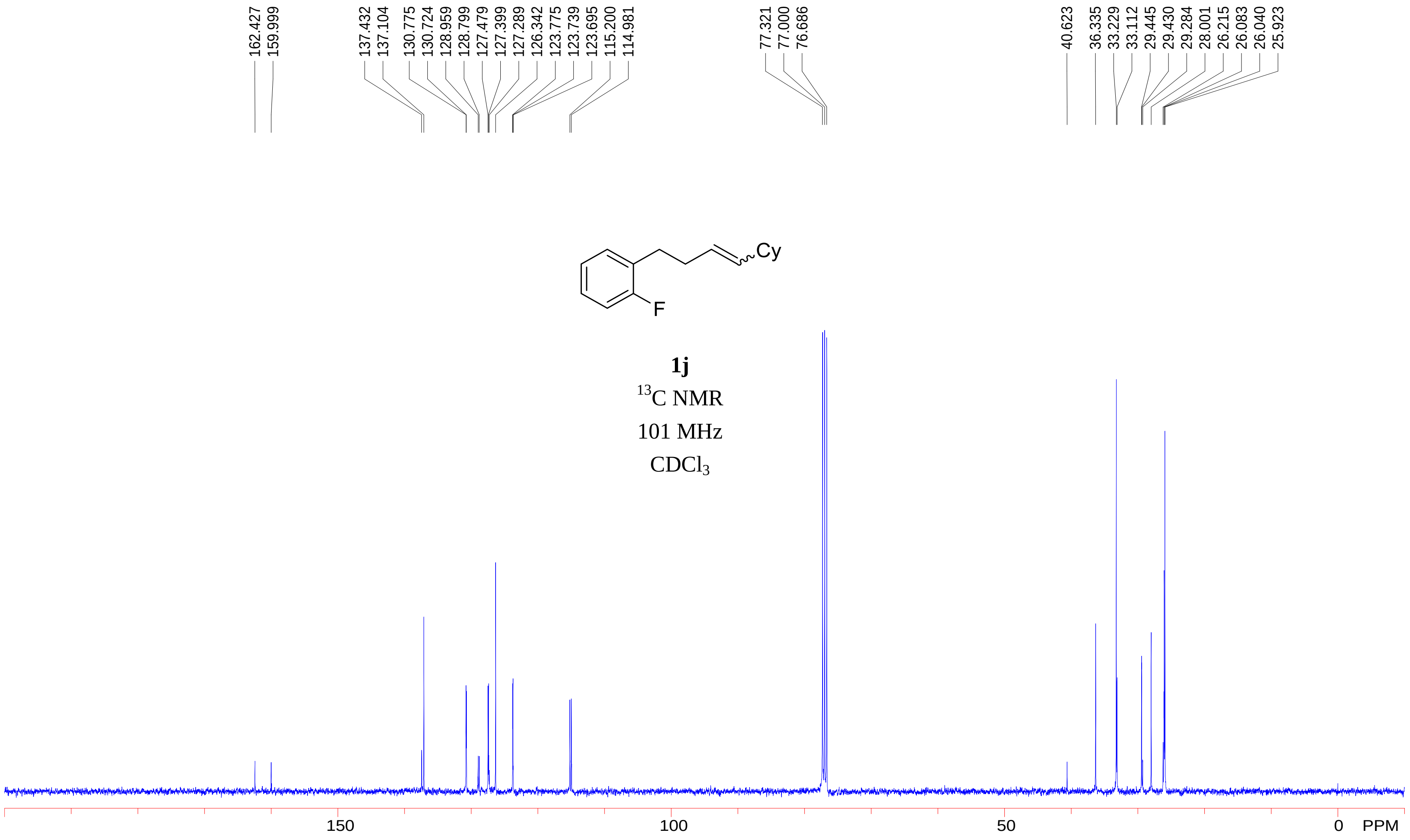

Supplementary Figure 69. ^{13}C NMR spectrum for **1j**

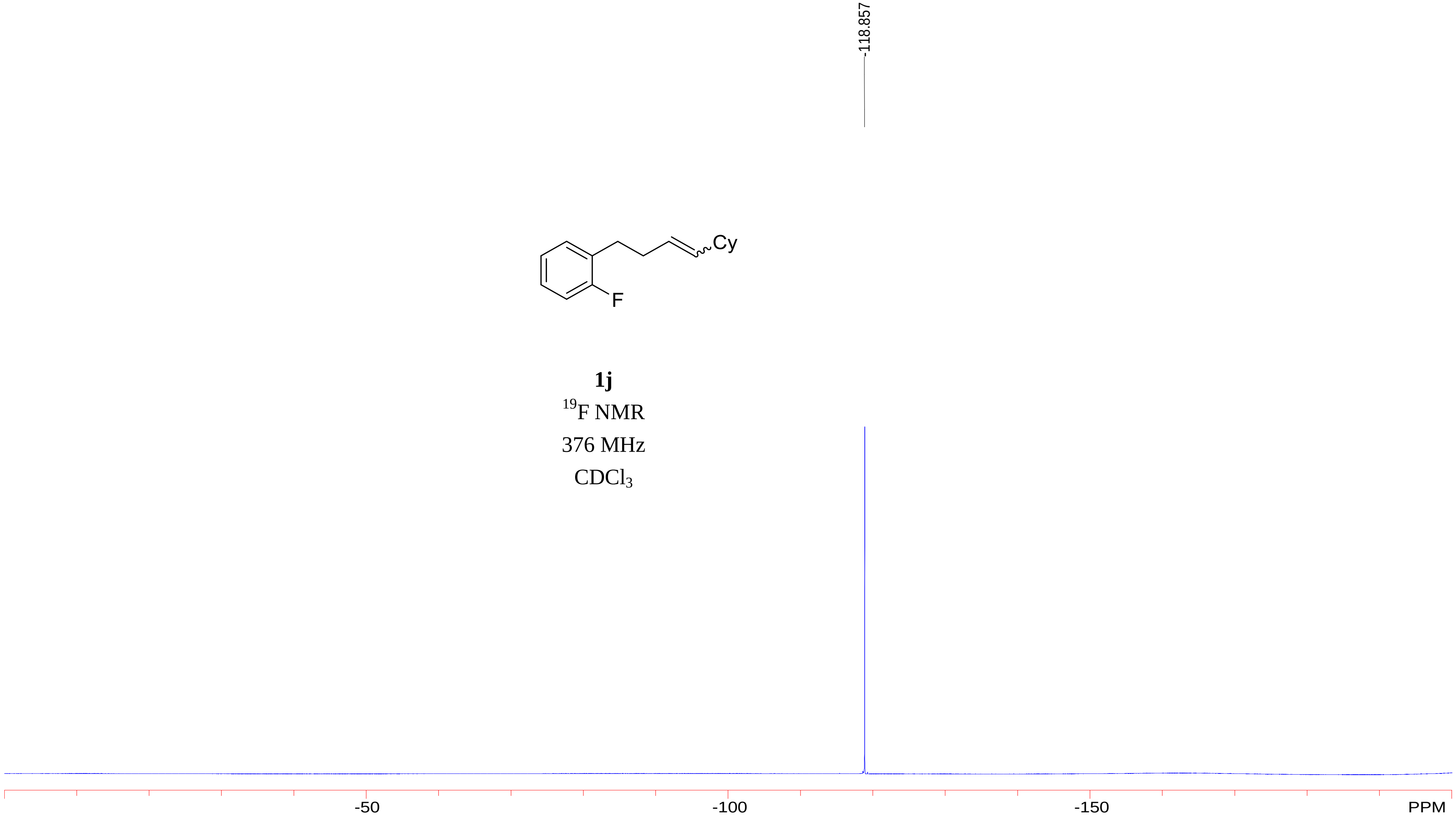

Supplementary Figure 70. ^{19}F NMR spectrum for **1j**

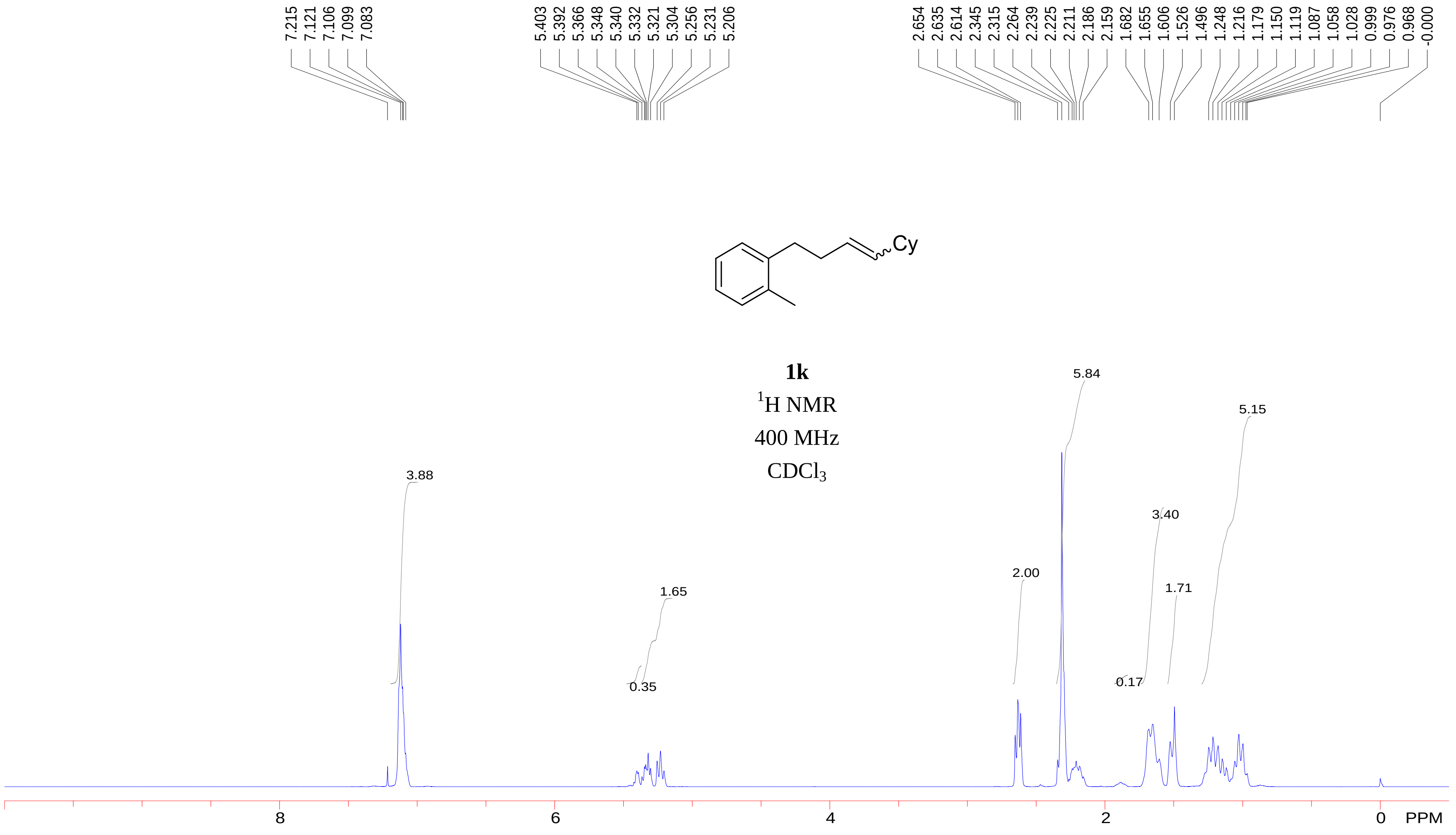

Supplementary Figure 71. ^{1}H NMR spectrum for **1k**

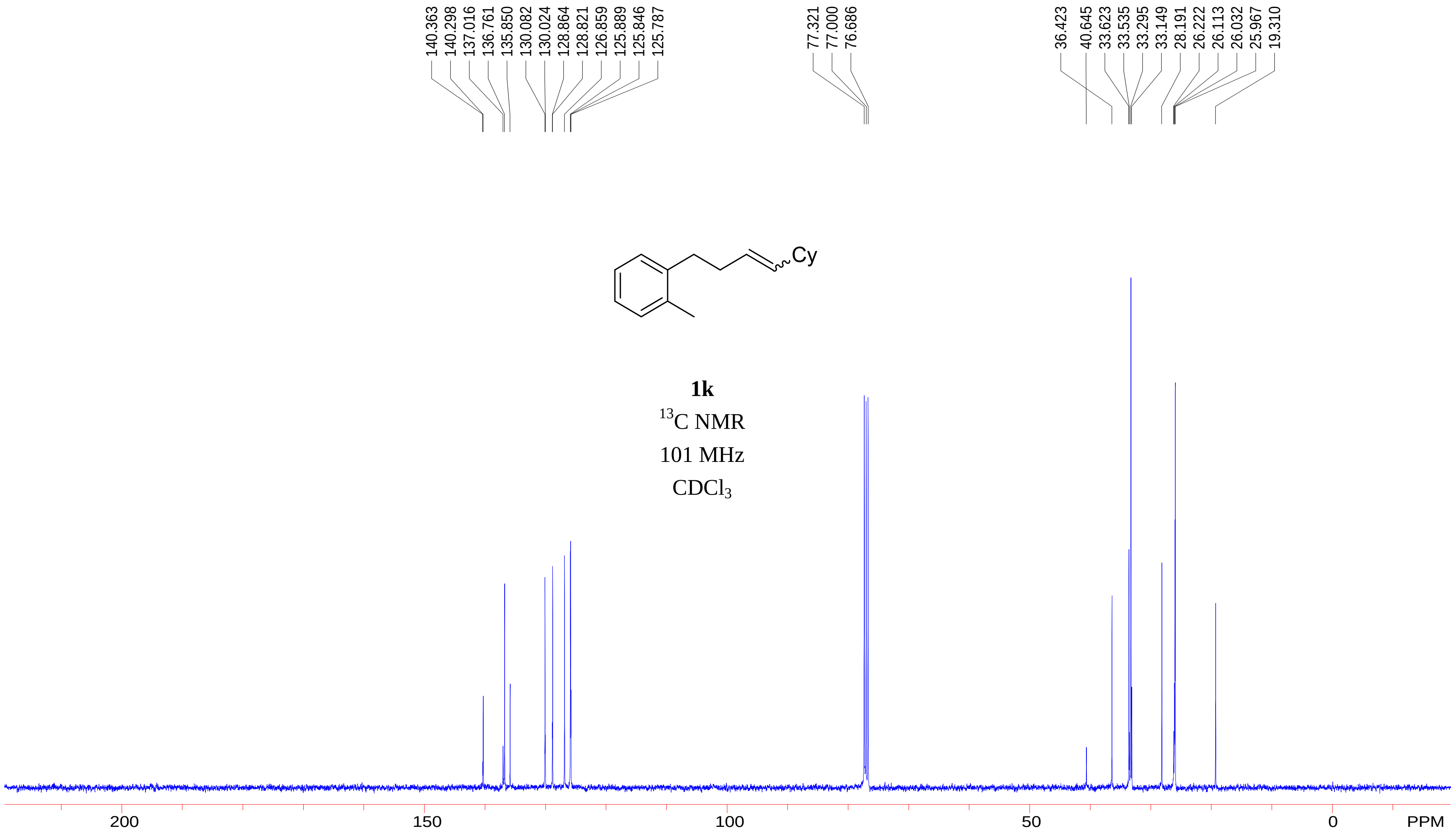

Supplementary Figure 72. ^{13}C NMR spectrum for **1k**

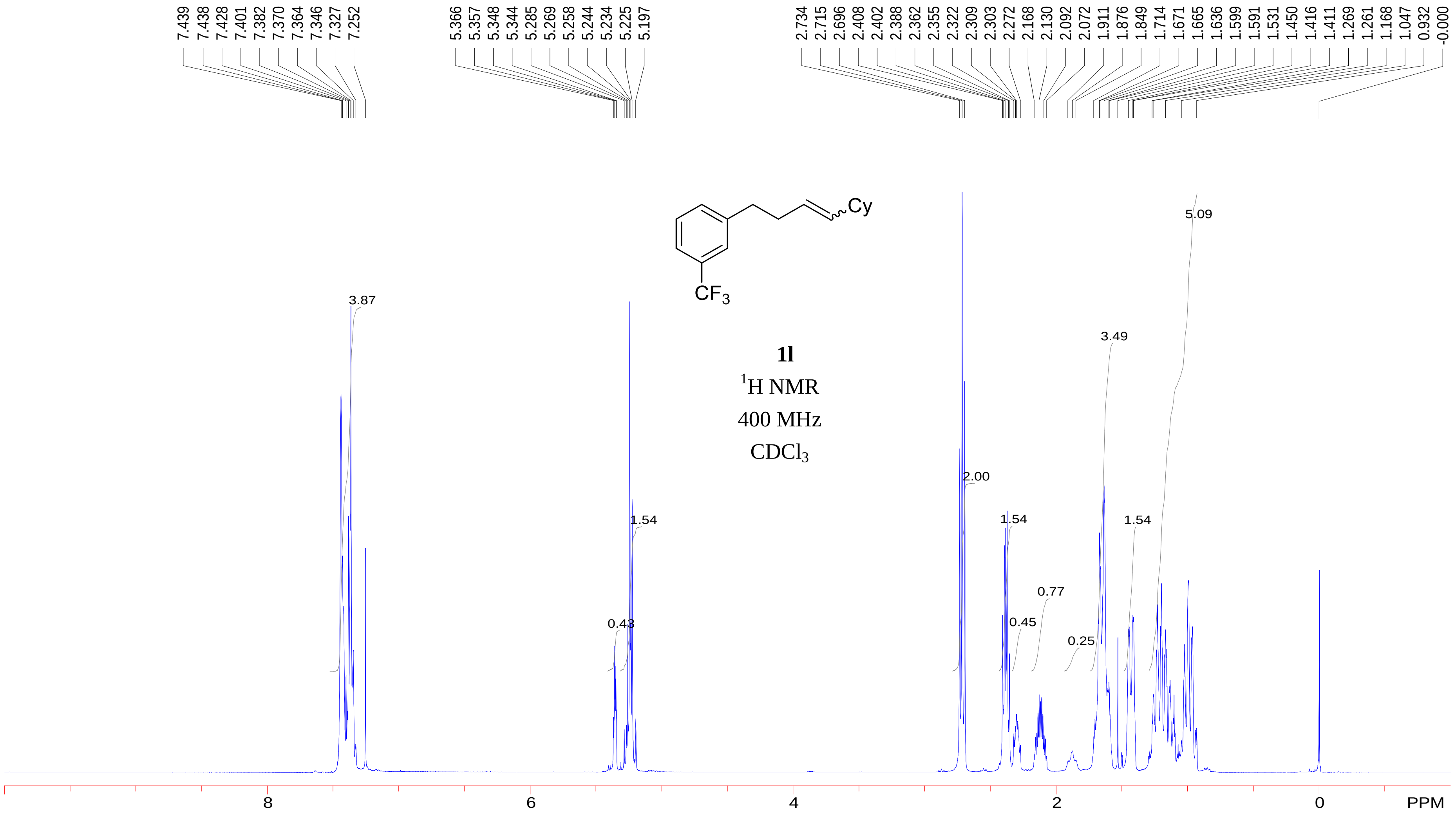

Supplementary Figure 73. ^{1}H NMR spectrum for **1l**

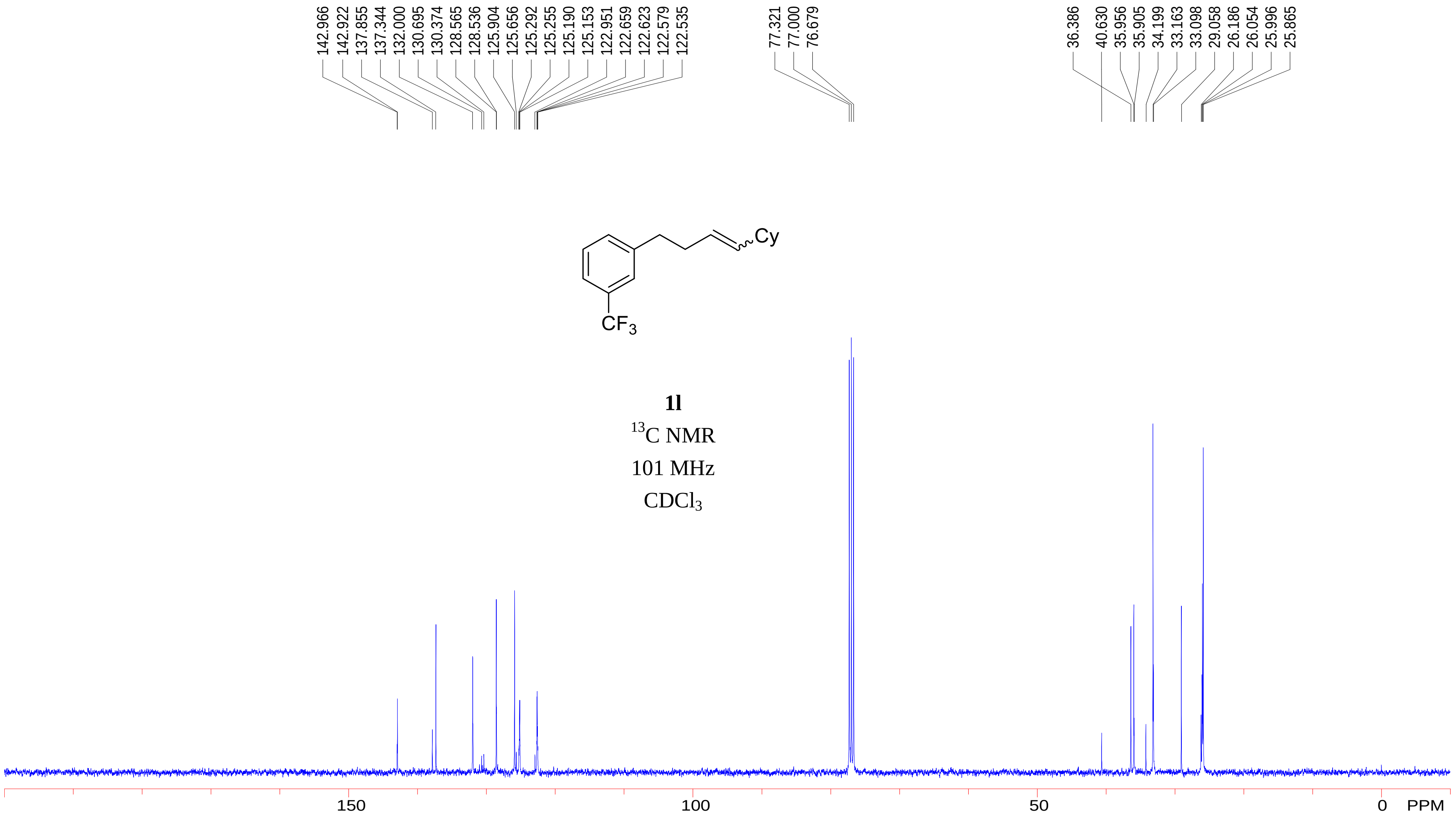

Supplementary Figure 74. ^{13}C NMR spectrum for **1l**

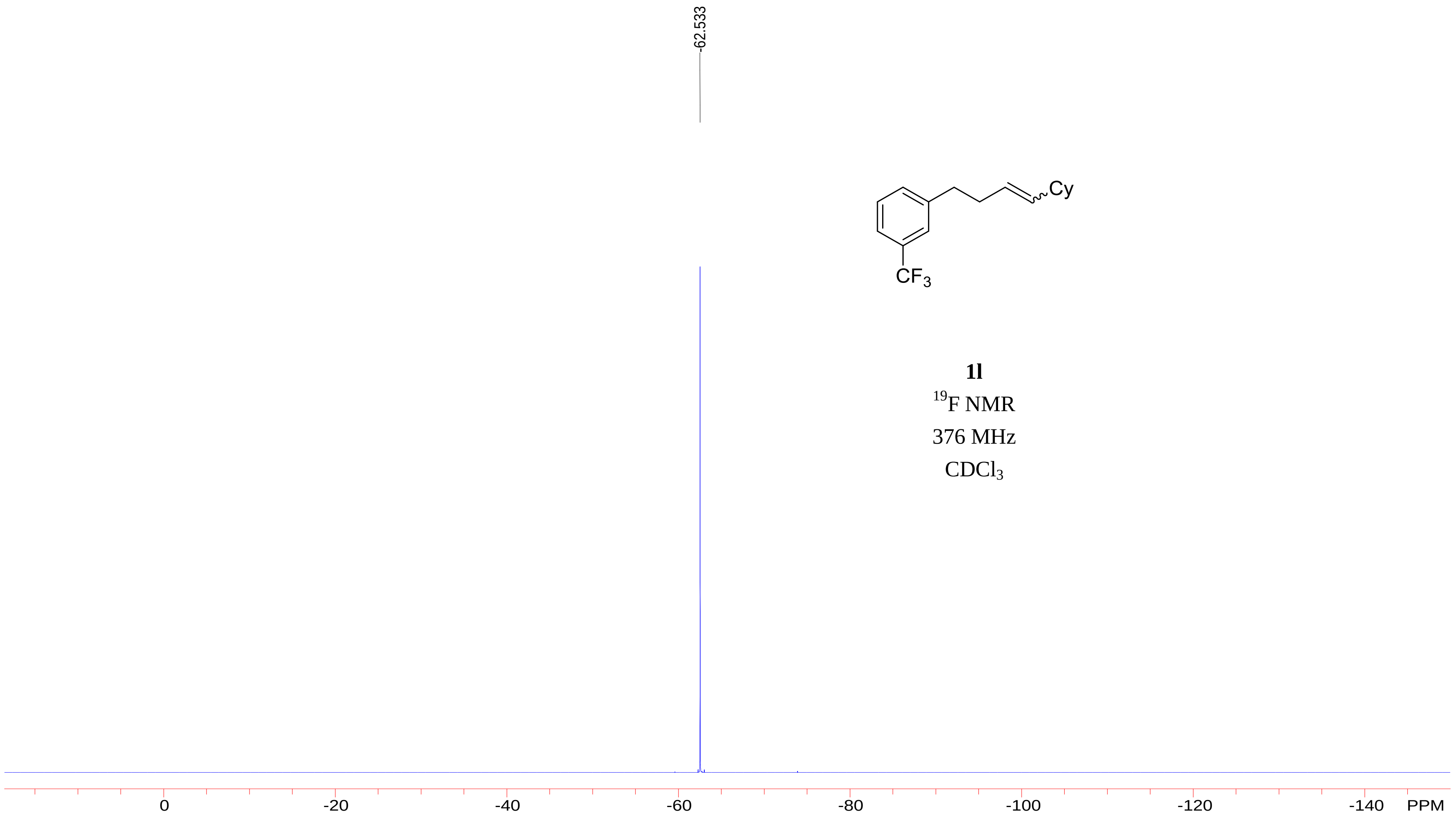

1l

^{19}F NMR

376 MHz

$CDCl_3$

Supplementary Figure 75. ^{19}F NMR spectrum for **1l**

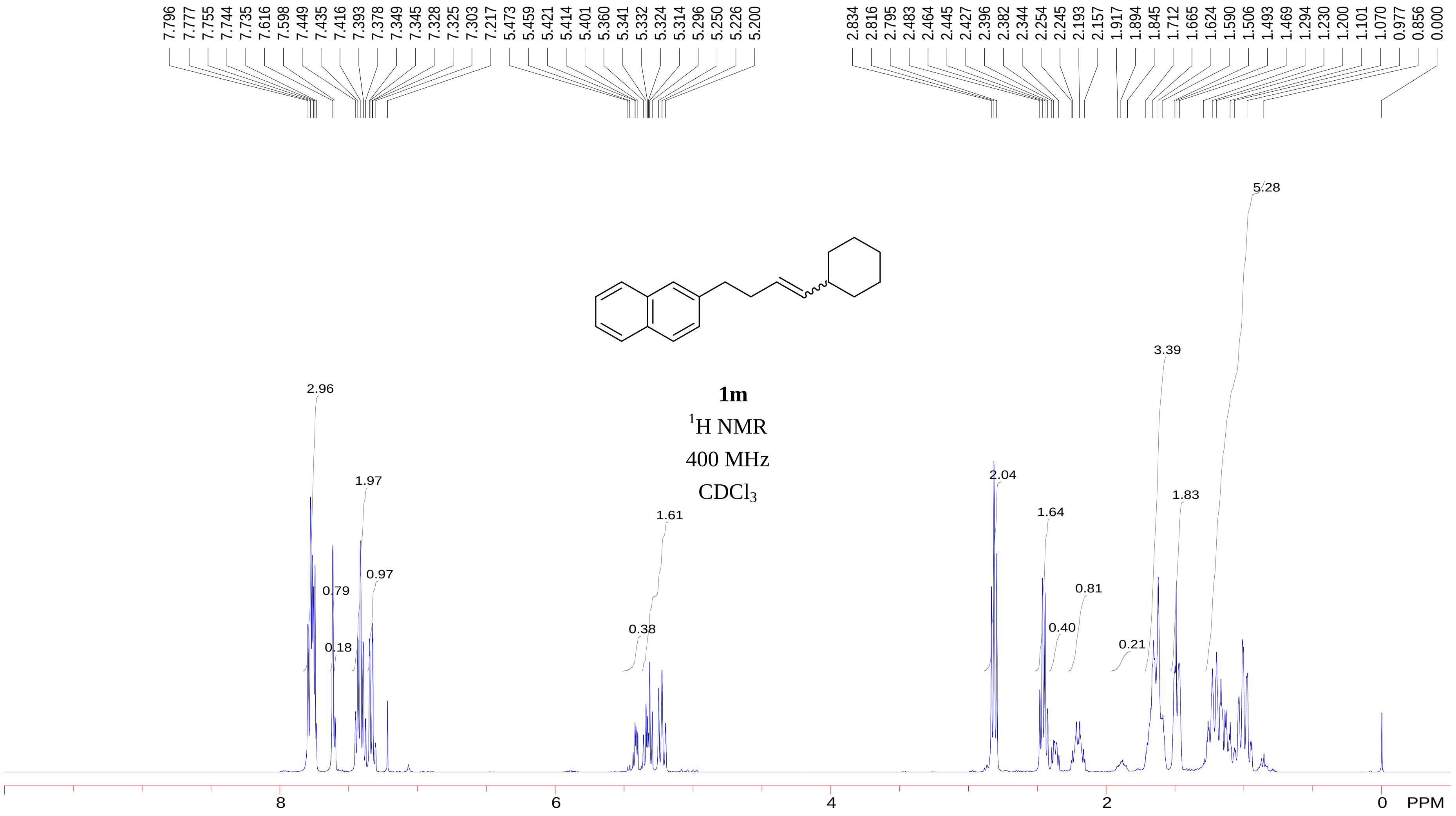

Supplementary Figure 76. ^{1}H NMR spectrum for **1m**

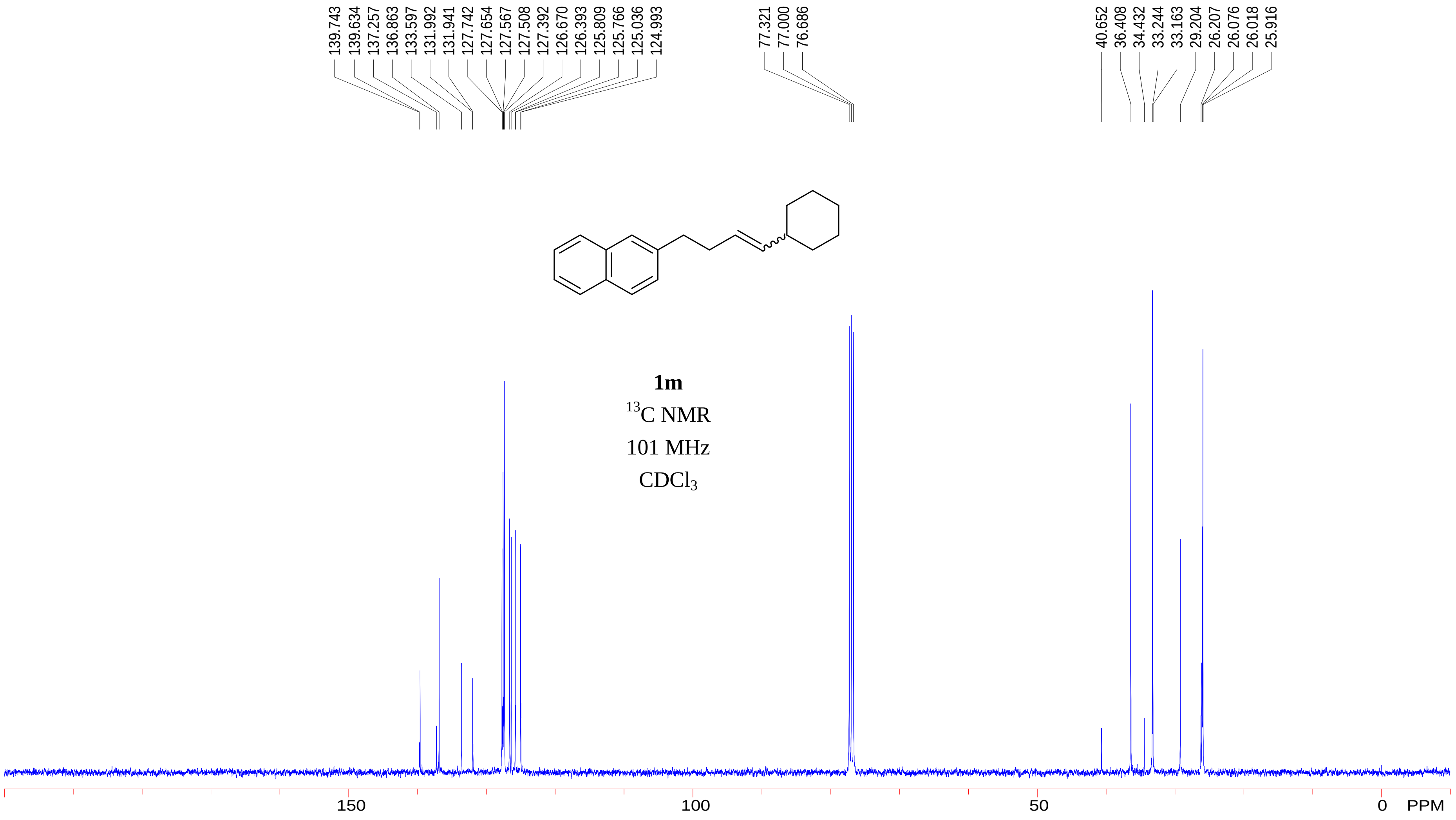

Supplementary Figure 77. ^{13}C NMR spectrum for **1m**

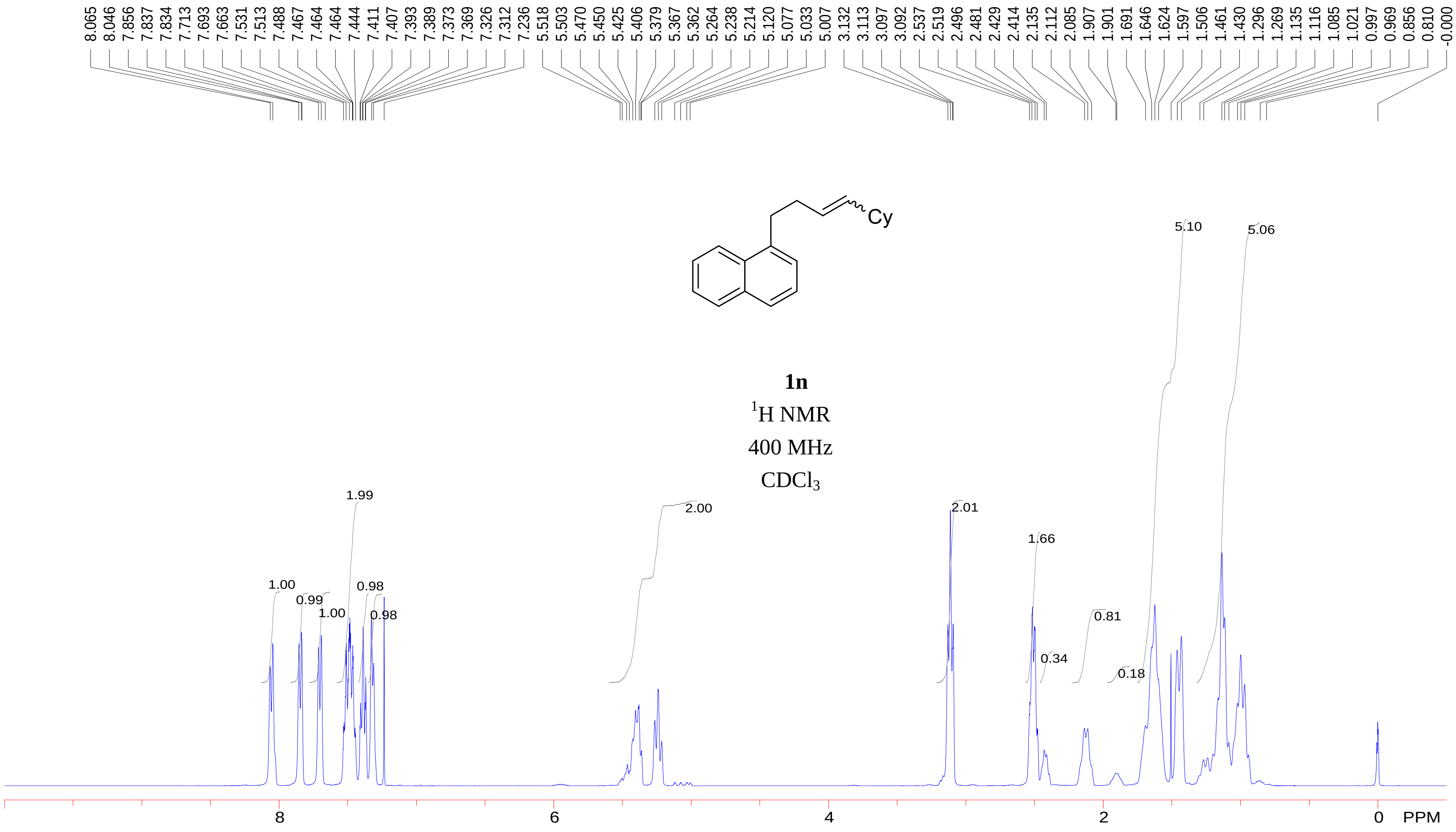

Supplementary Figure 78. ^{1}H NMR spectrum for **1n**

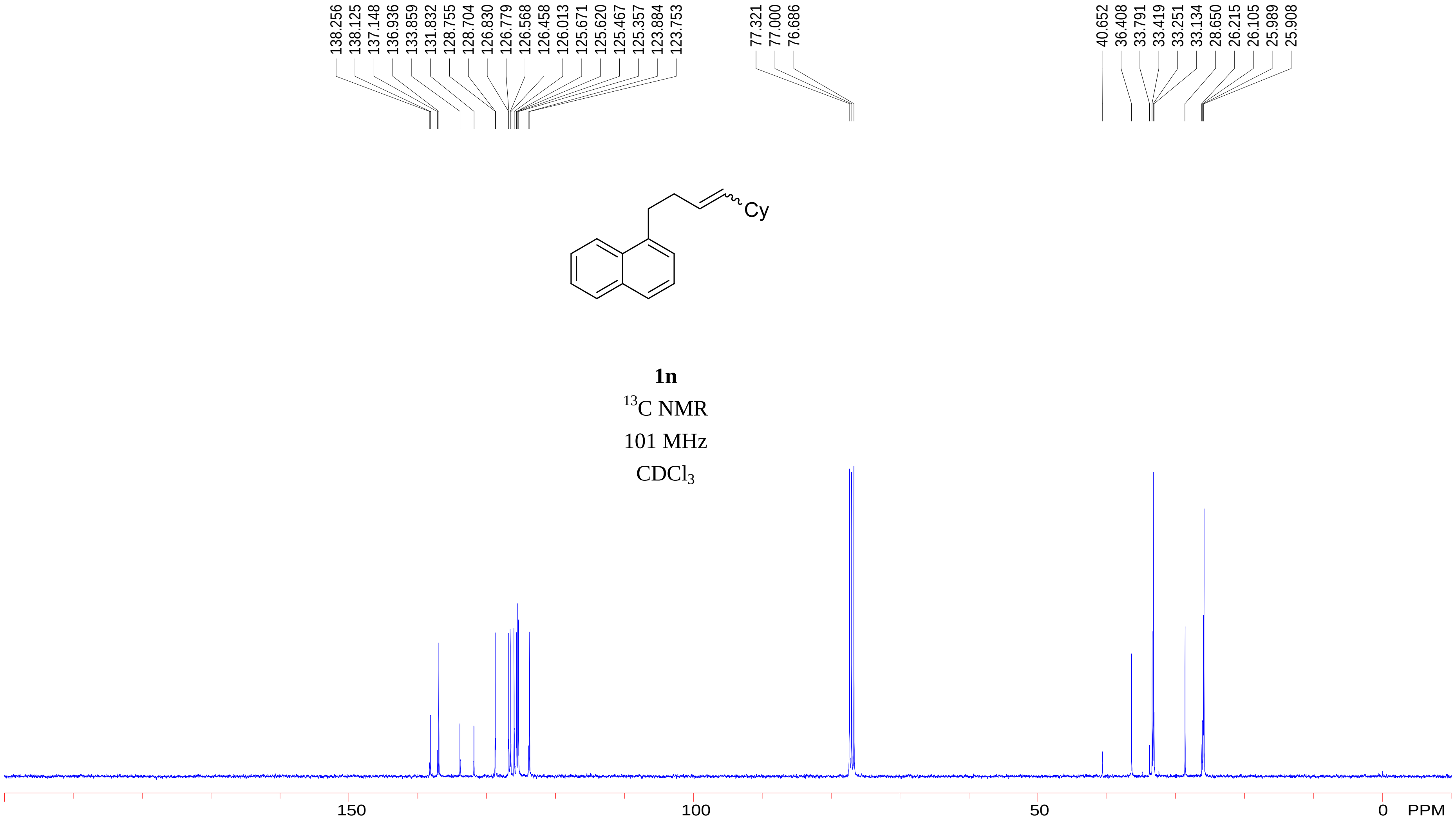

Supplementary Figure 79. ^{13}C NMR spectrum for **1n**

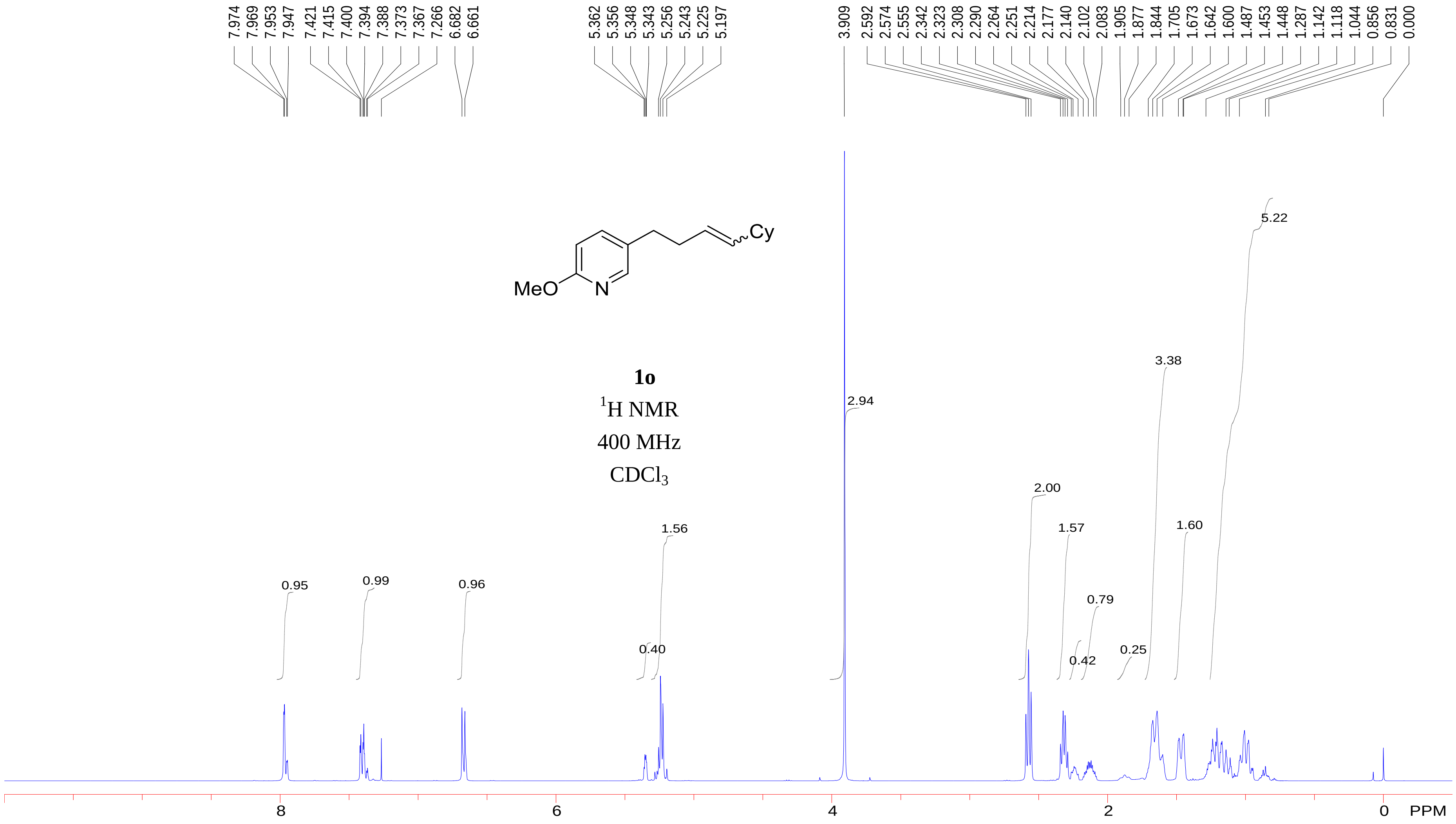

Supplementary Figure 80. ^{1}H NMR spectrum for **1o**

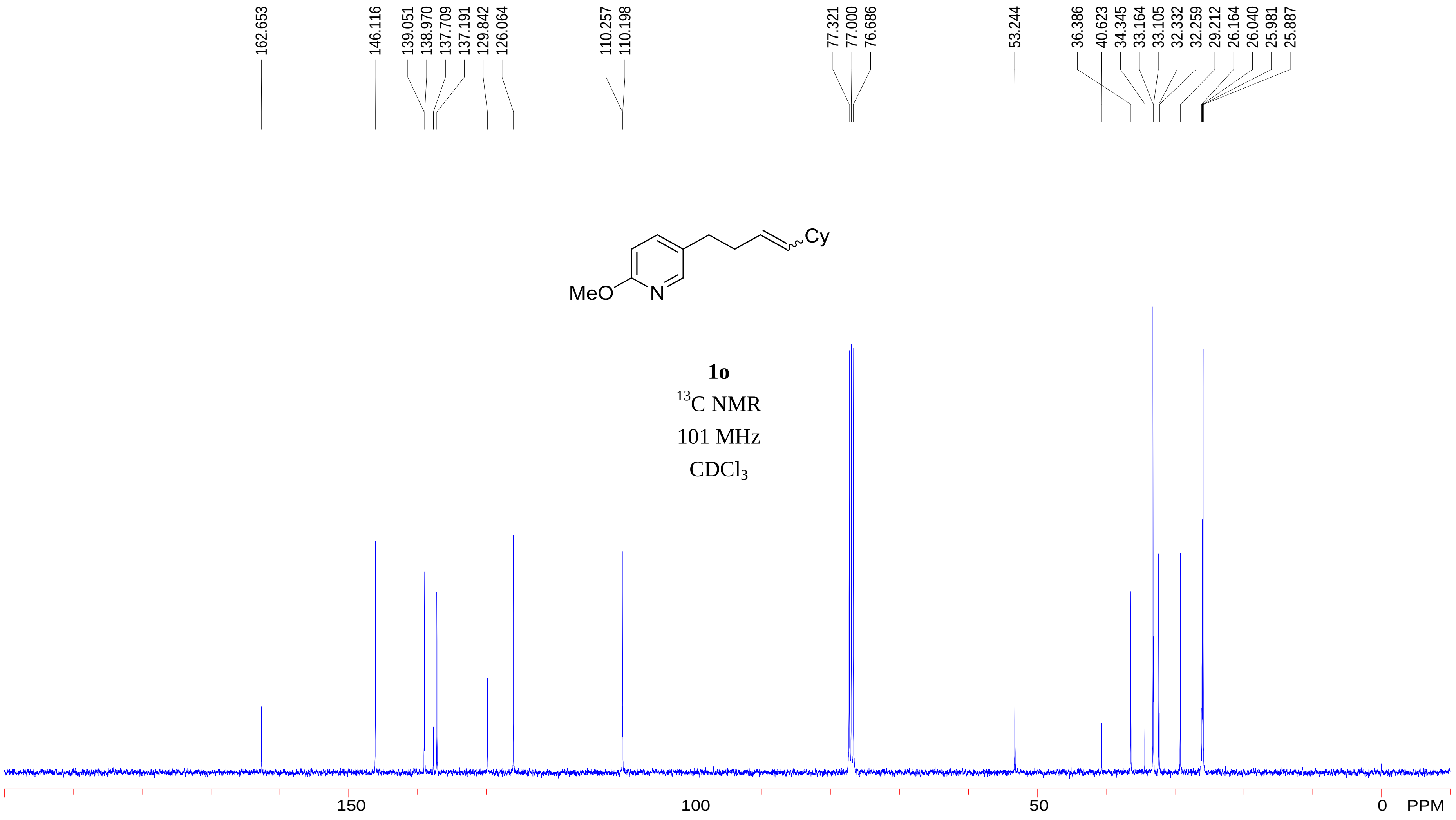

Supplementary Figure 81. ^{13}C NMR spectrum for **1o**

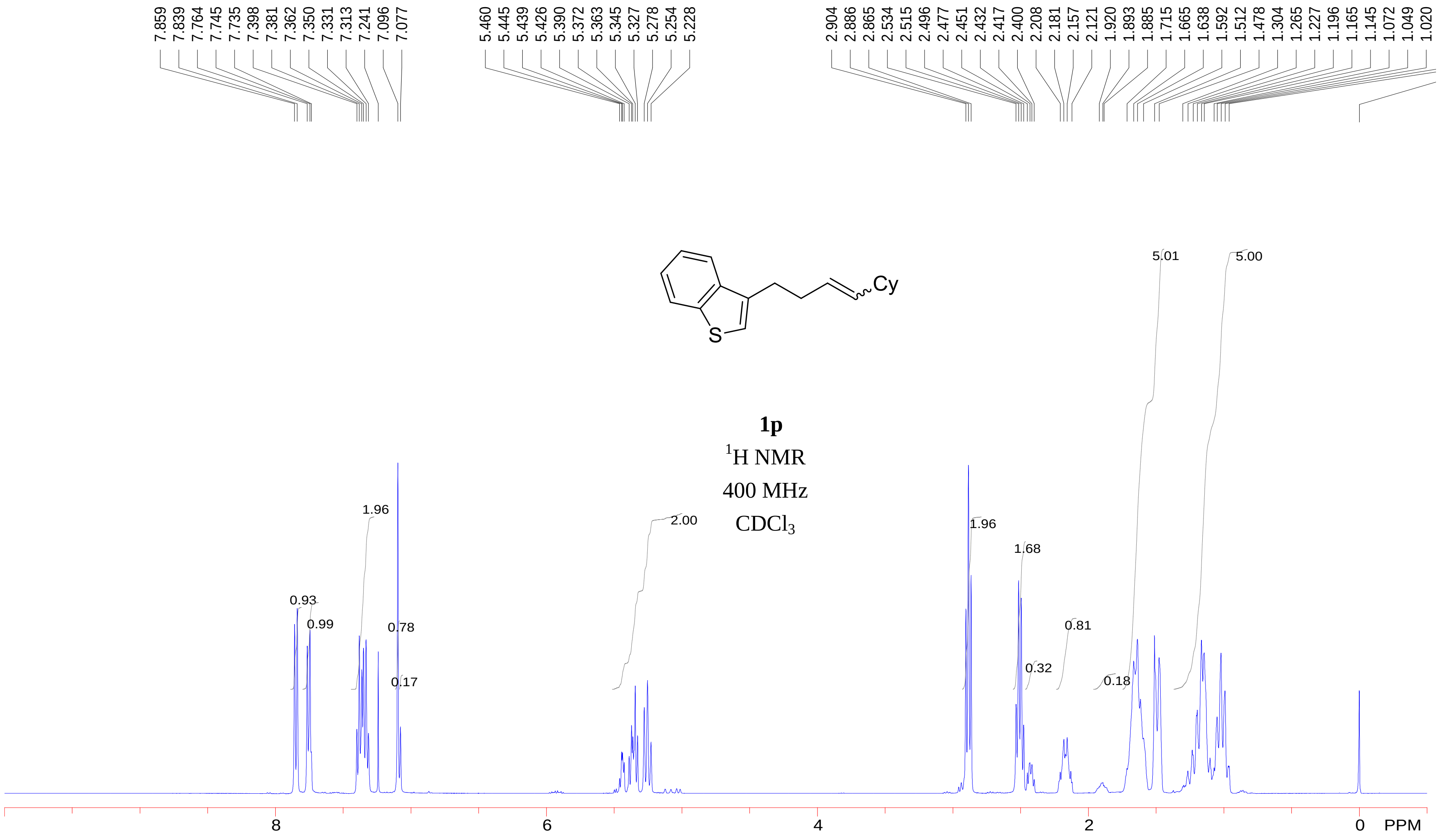

Supplementary Figure 82. ^{1}H NMR spectrum for **1p**

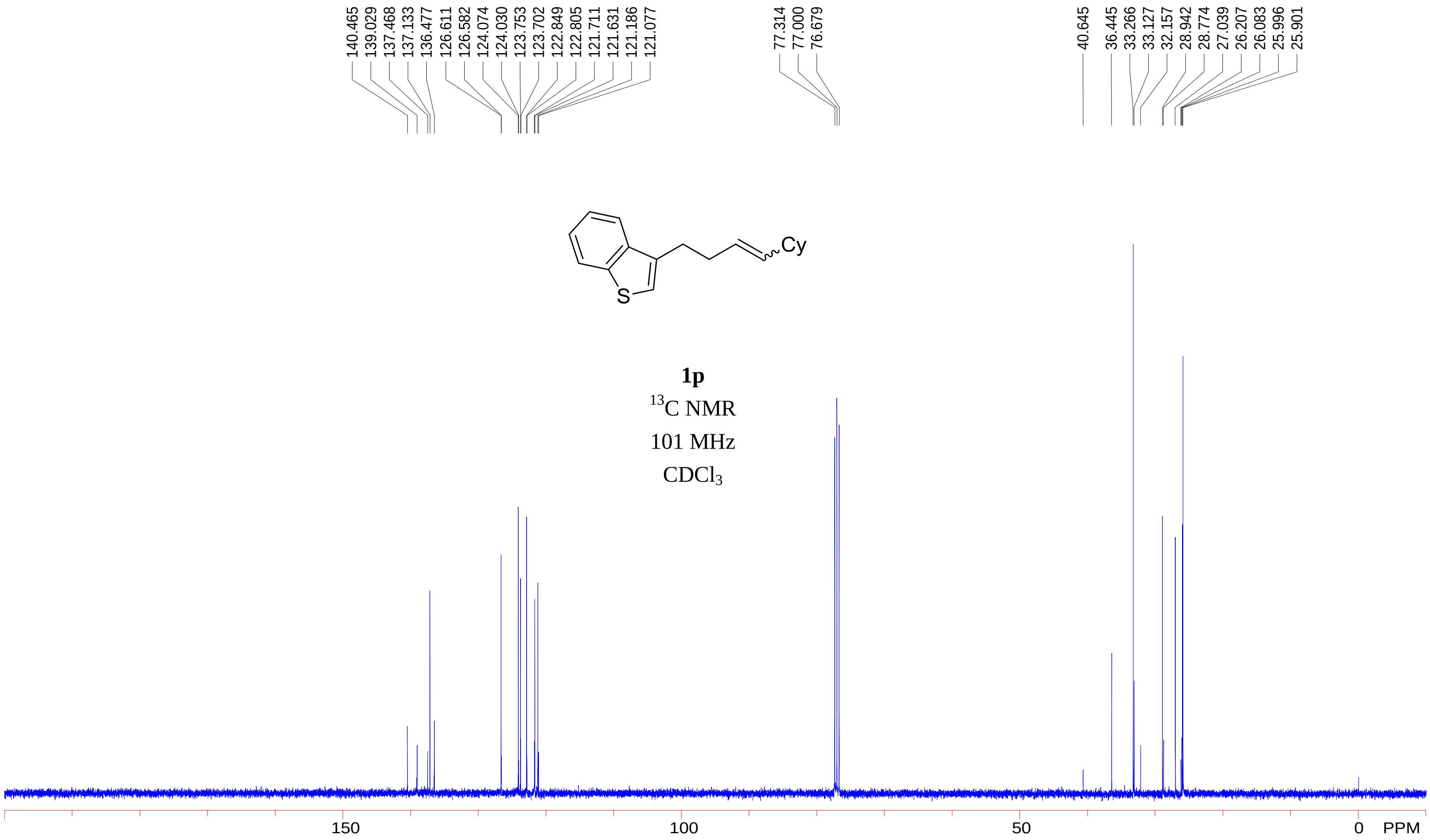

Supplementary Figure 83. ^{13}C NMR spectrum for **1p**

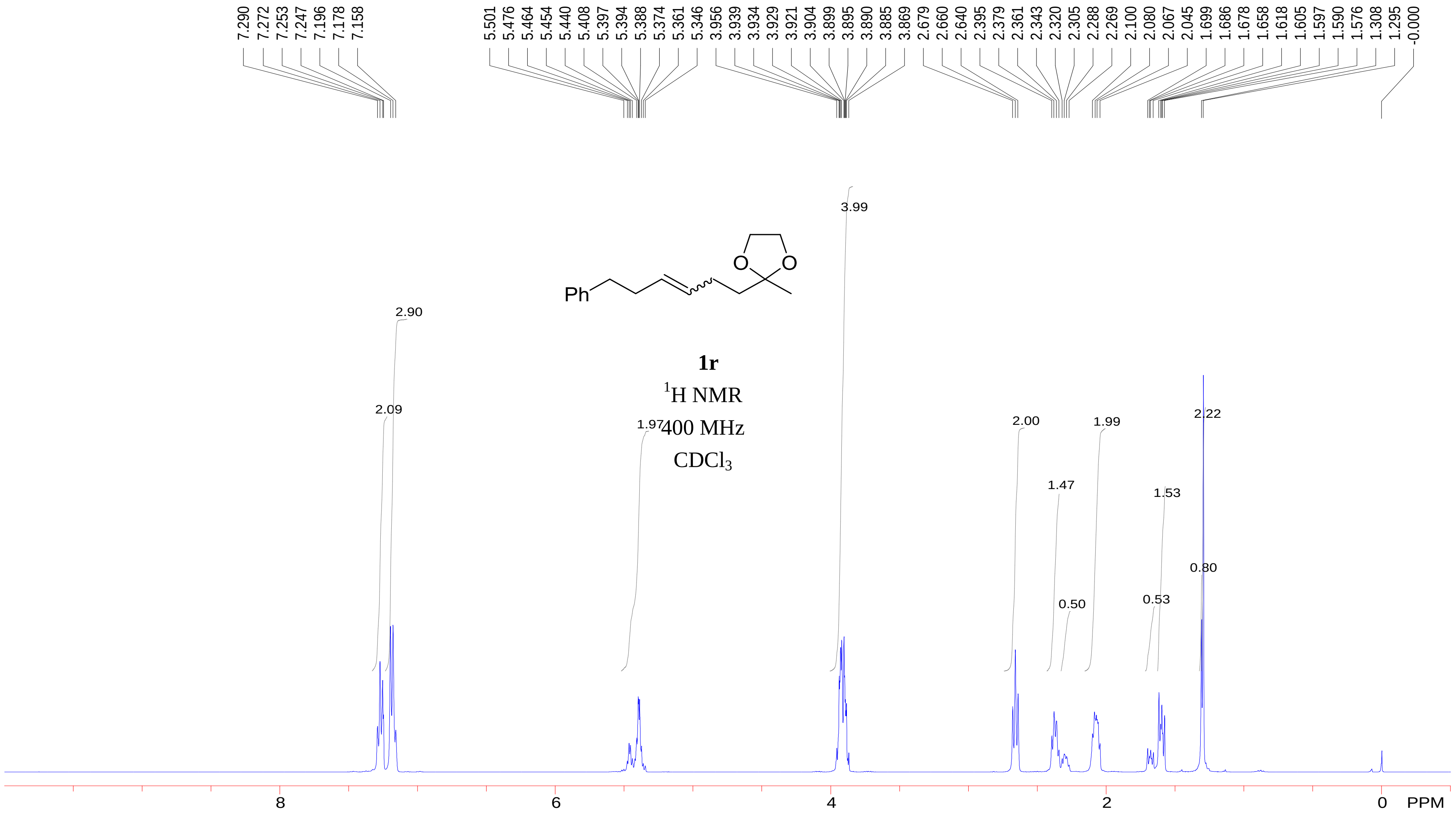

Supplementary Figure 84. ^{1}H NMR spectrum for **1r**

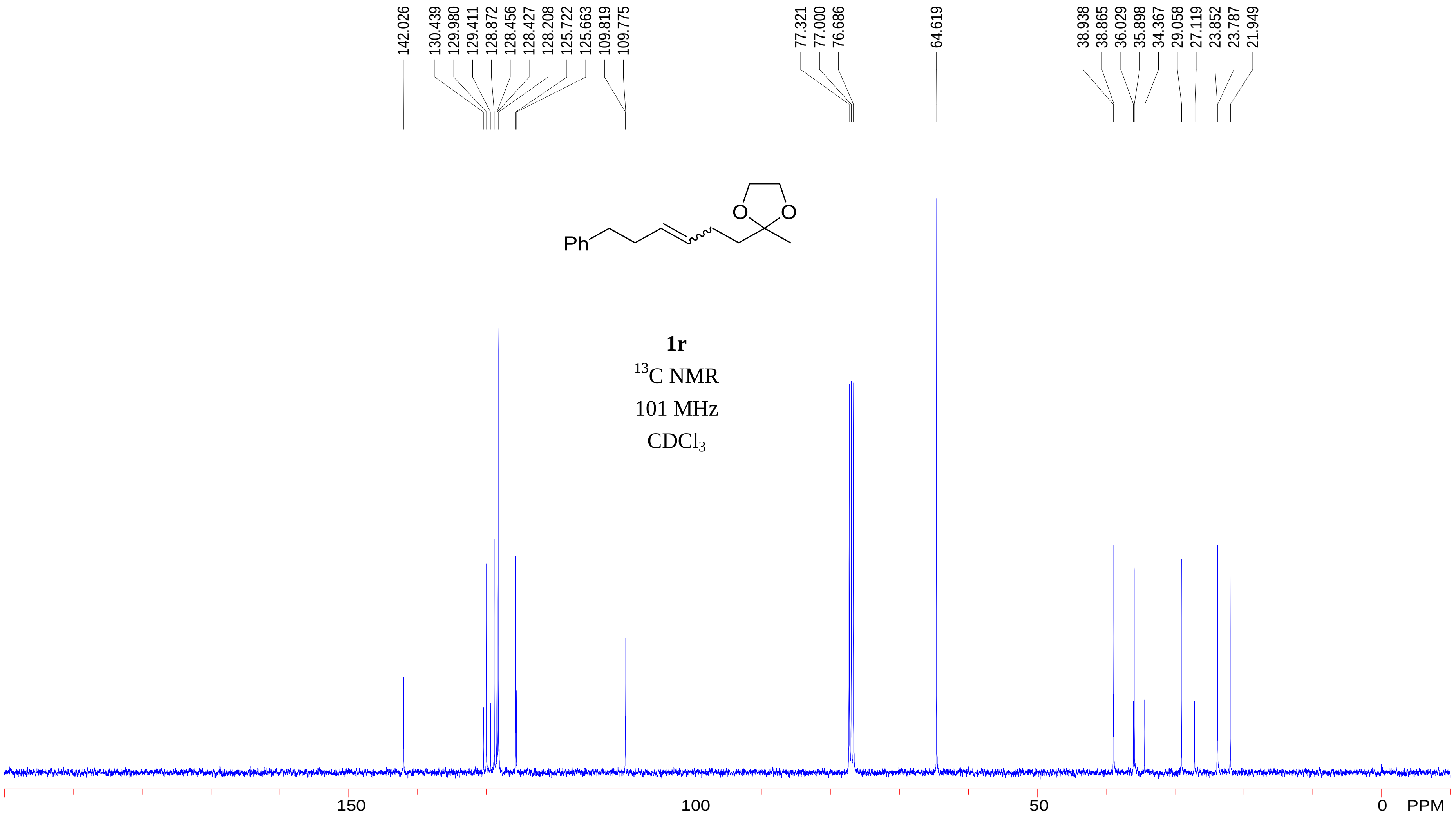

Supplementary Figure 85. ^{13}C NMR spectrum for **1r**

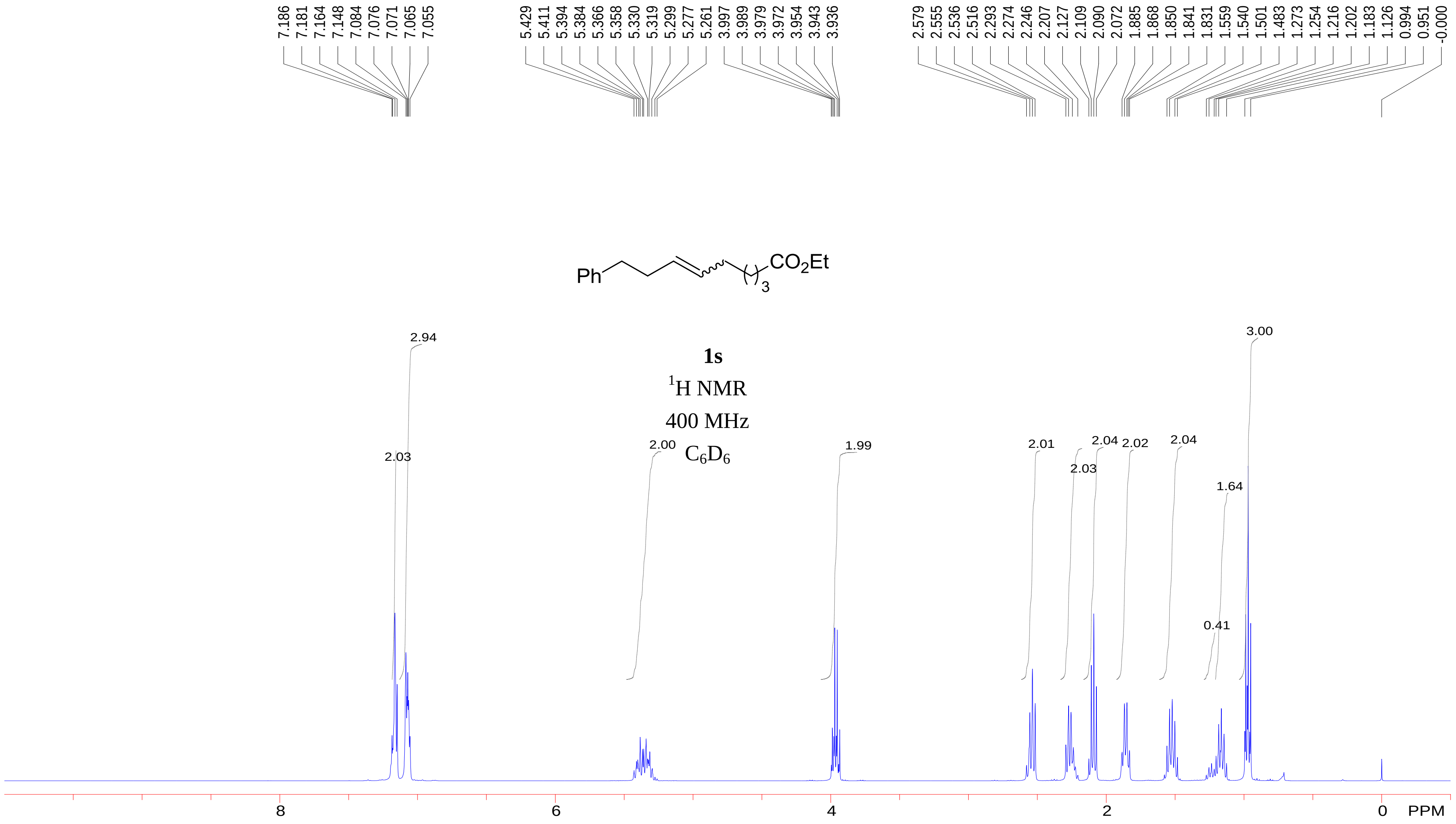

Supplementary Figure 86. ^{1}H NMR spectrum for **1s**

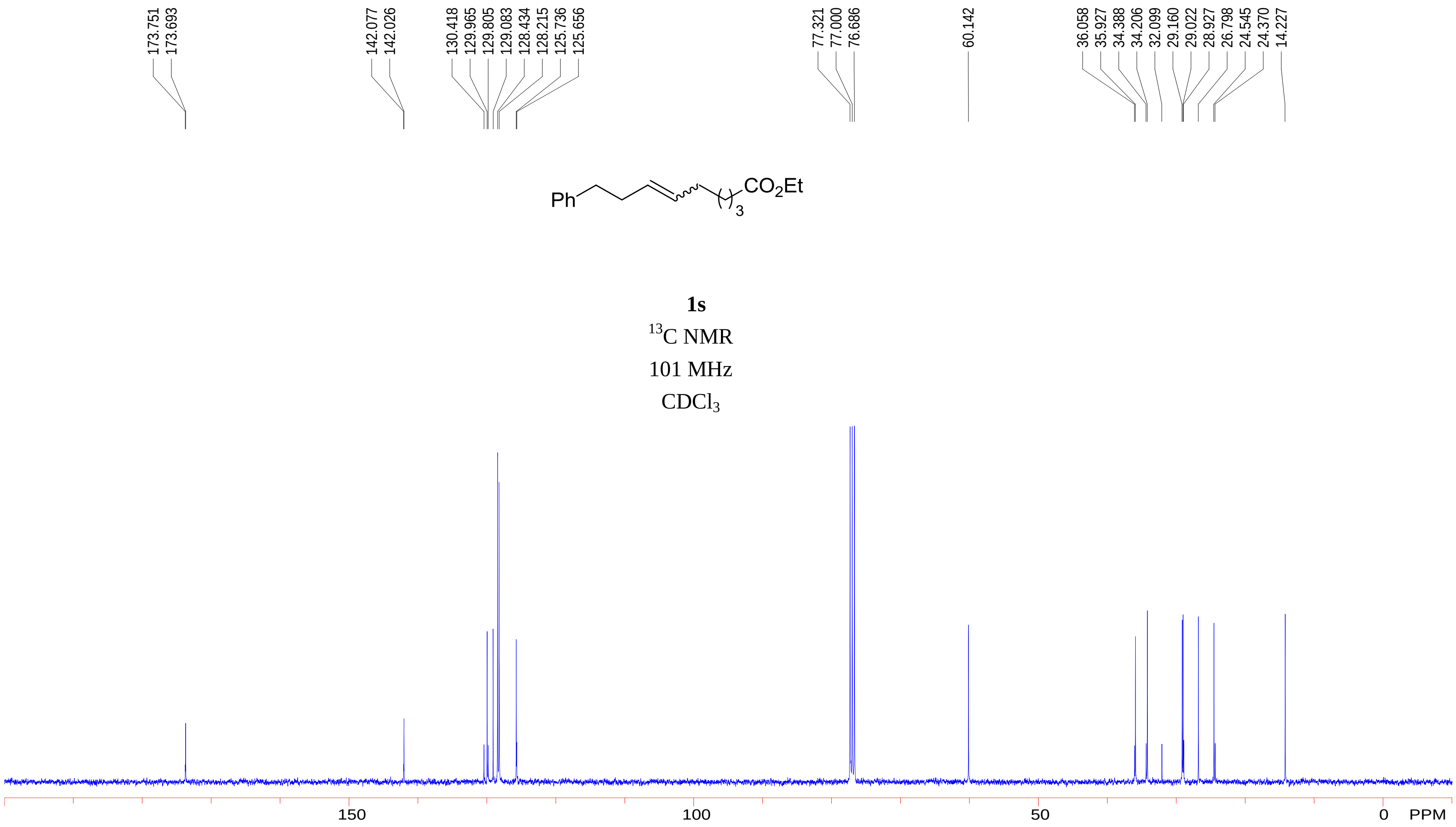

Supplementary Figure 87. ^{13}C NMR spectrum for **1s**

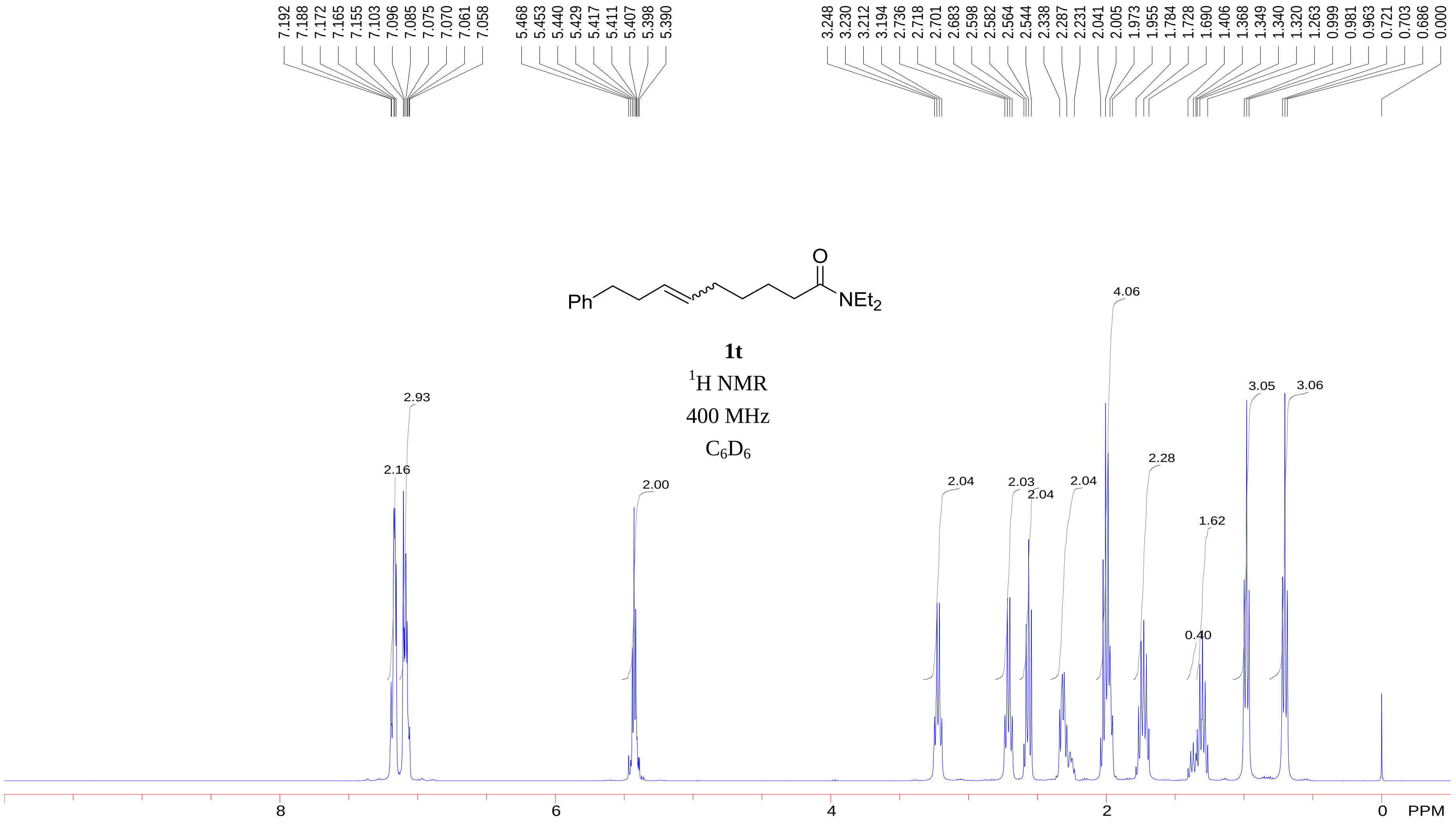

Supplementary Figure 88. ^{1}H NMR spectrum for **1t**

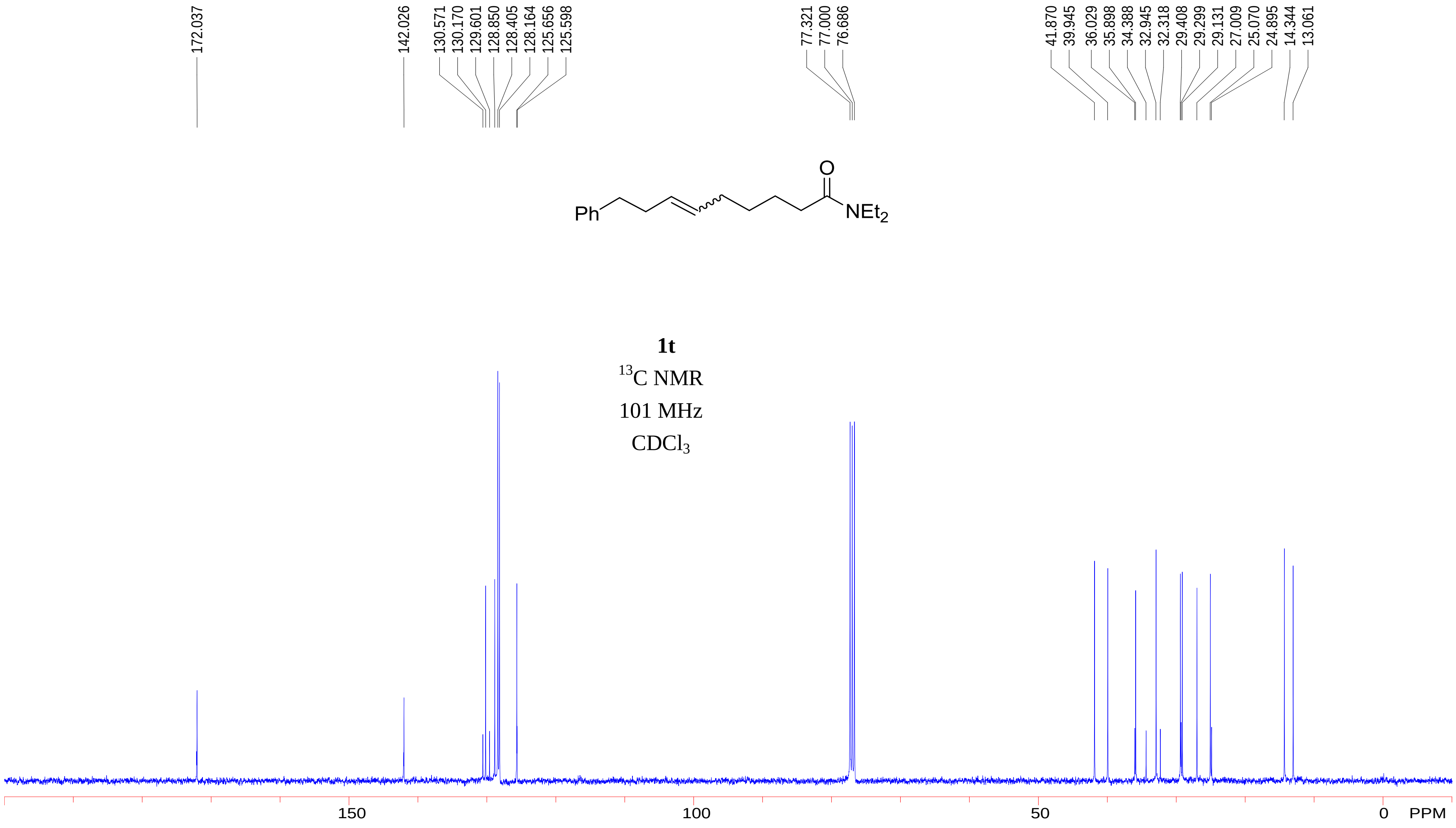

Supplementary Figure 89. ^{13}C NMR spectrum for **1t**

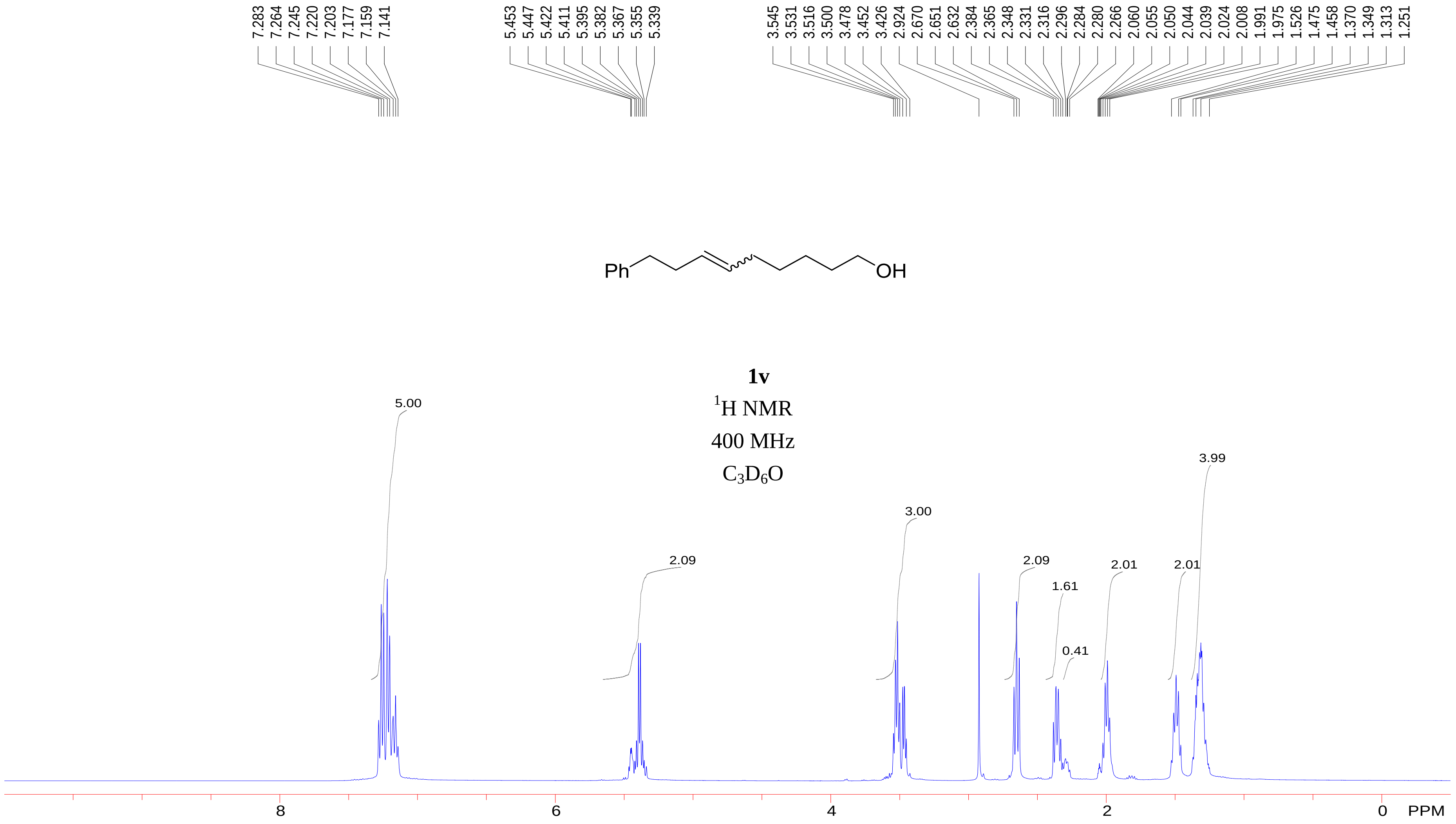

Supplementary Figure 90. ^{1}H NMR spectrum for **1v**

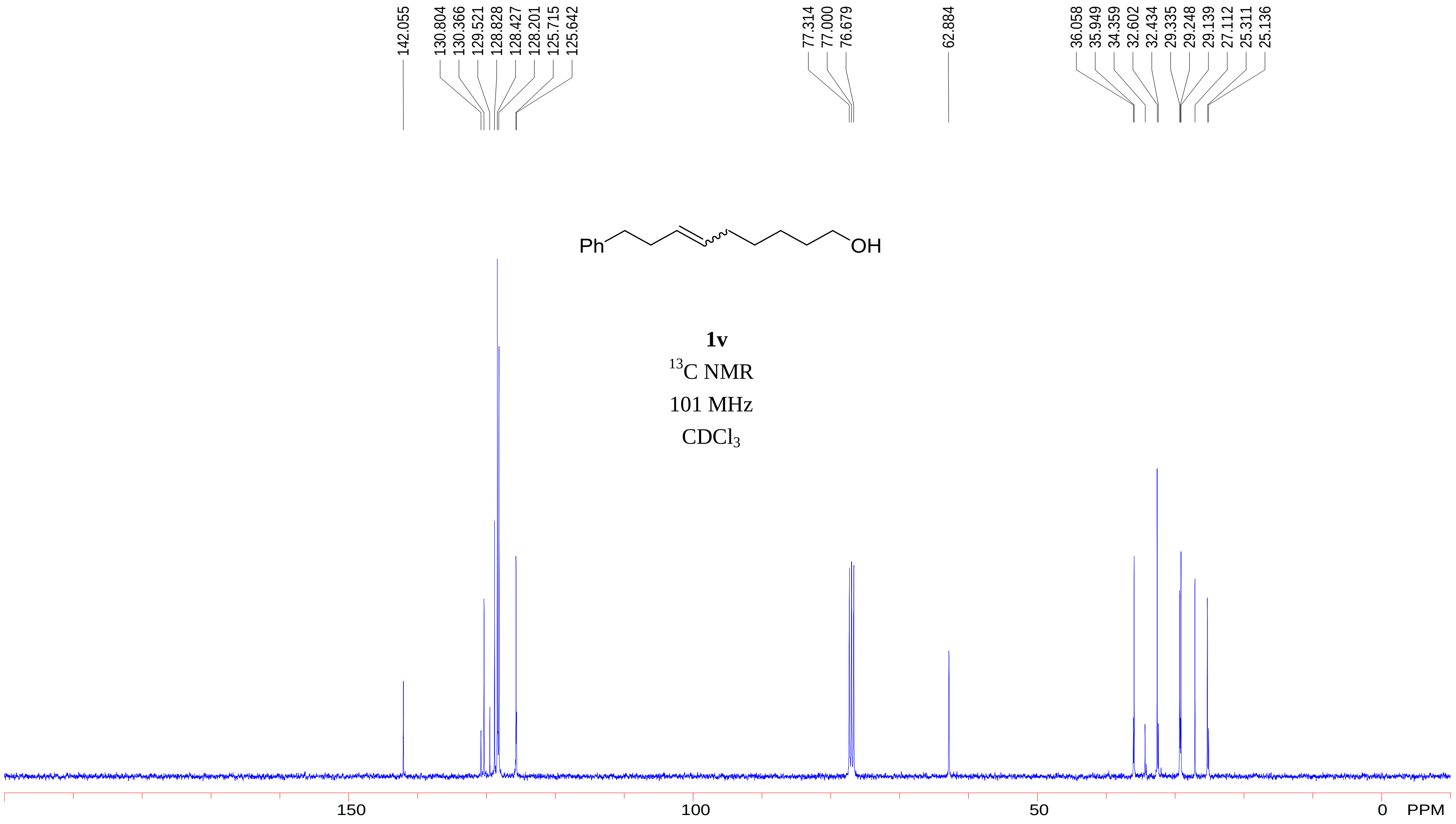

Supplementary Figure 91. ^{13}C NMR spectrum for **1v**

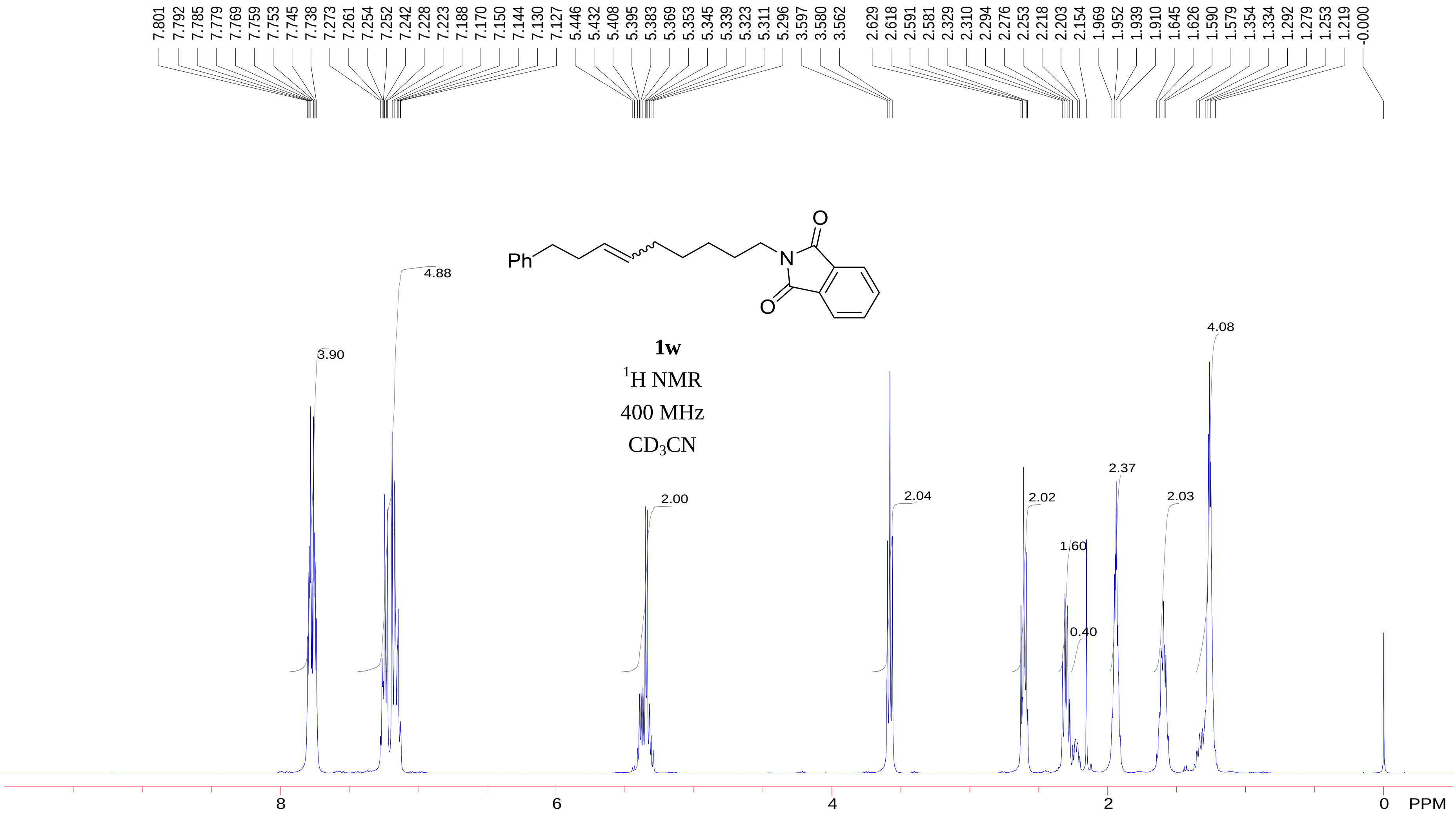

Supplementary Figure 92. ^{1}H NMR spectrum for **1w**

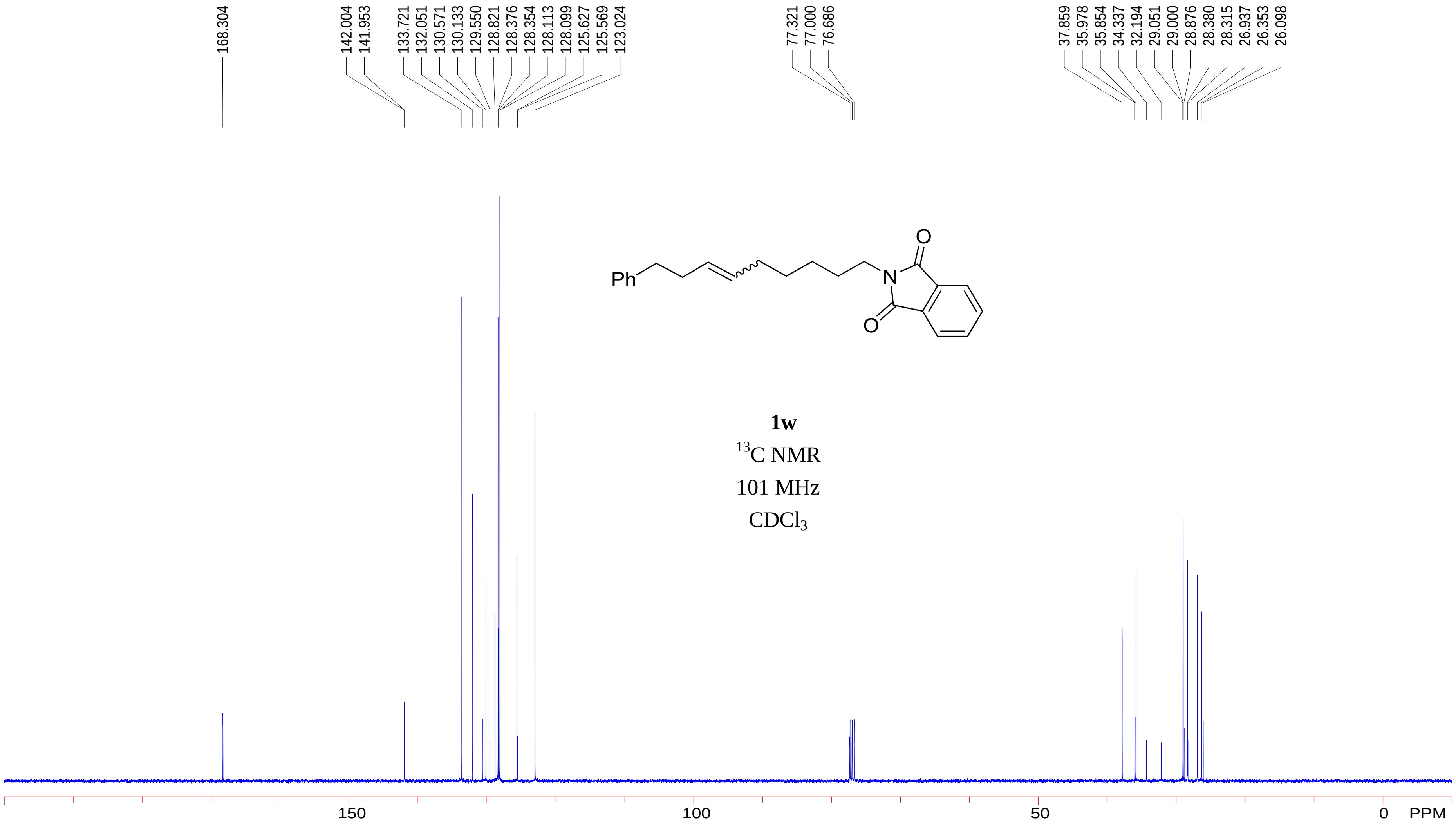

Supplementary Figure 93. ^{13}C NMR spectrum for **1w**

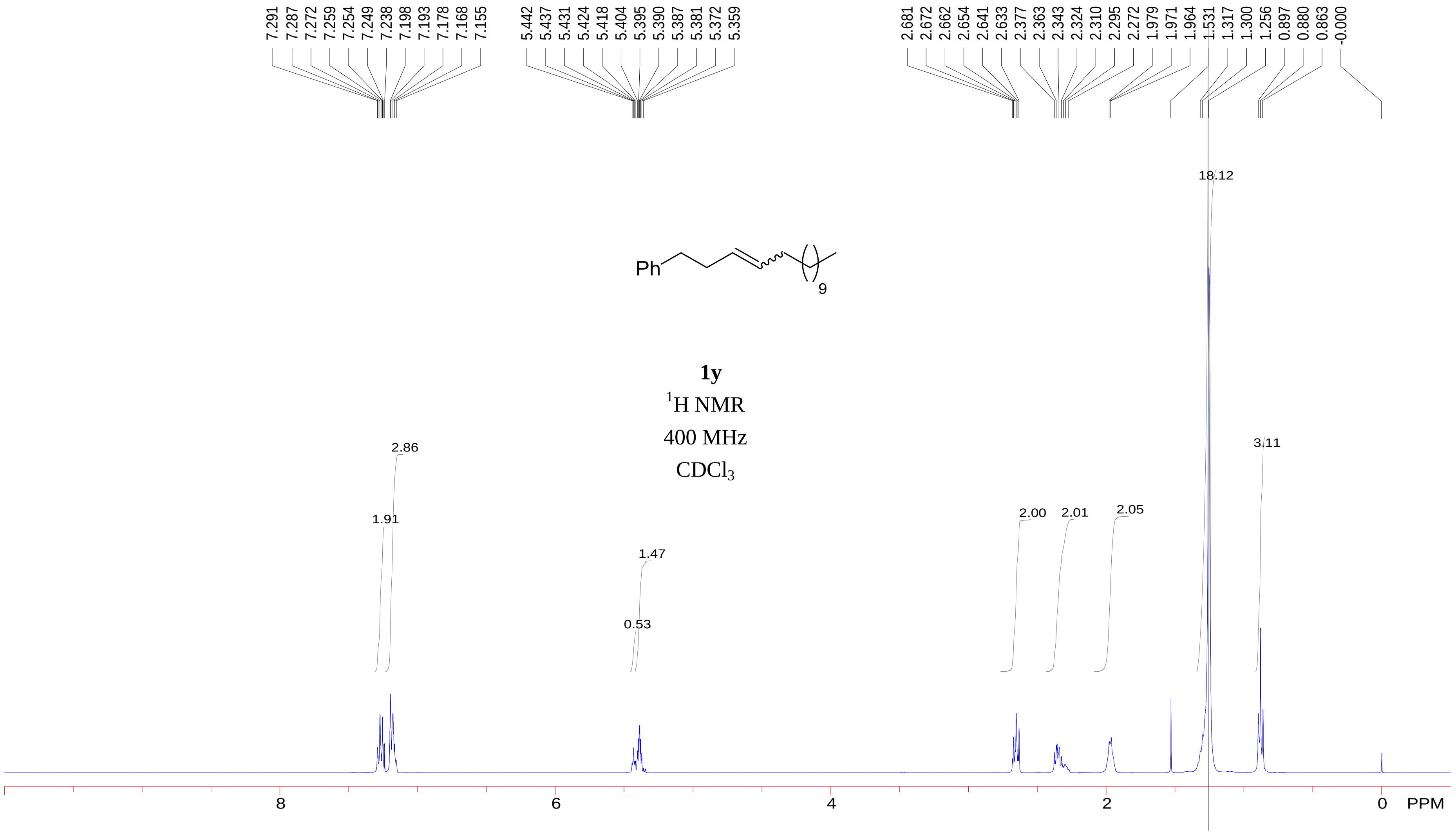

Supplementary Figure 94. ^{1}H NMR spectrum for **1y**

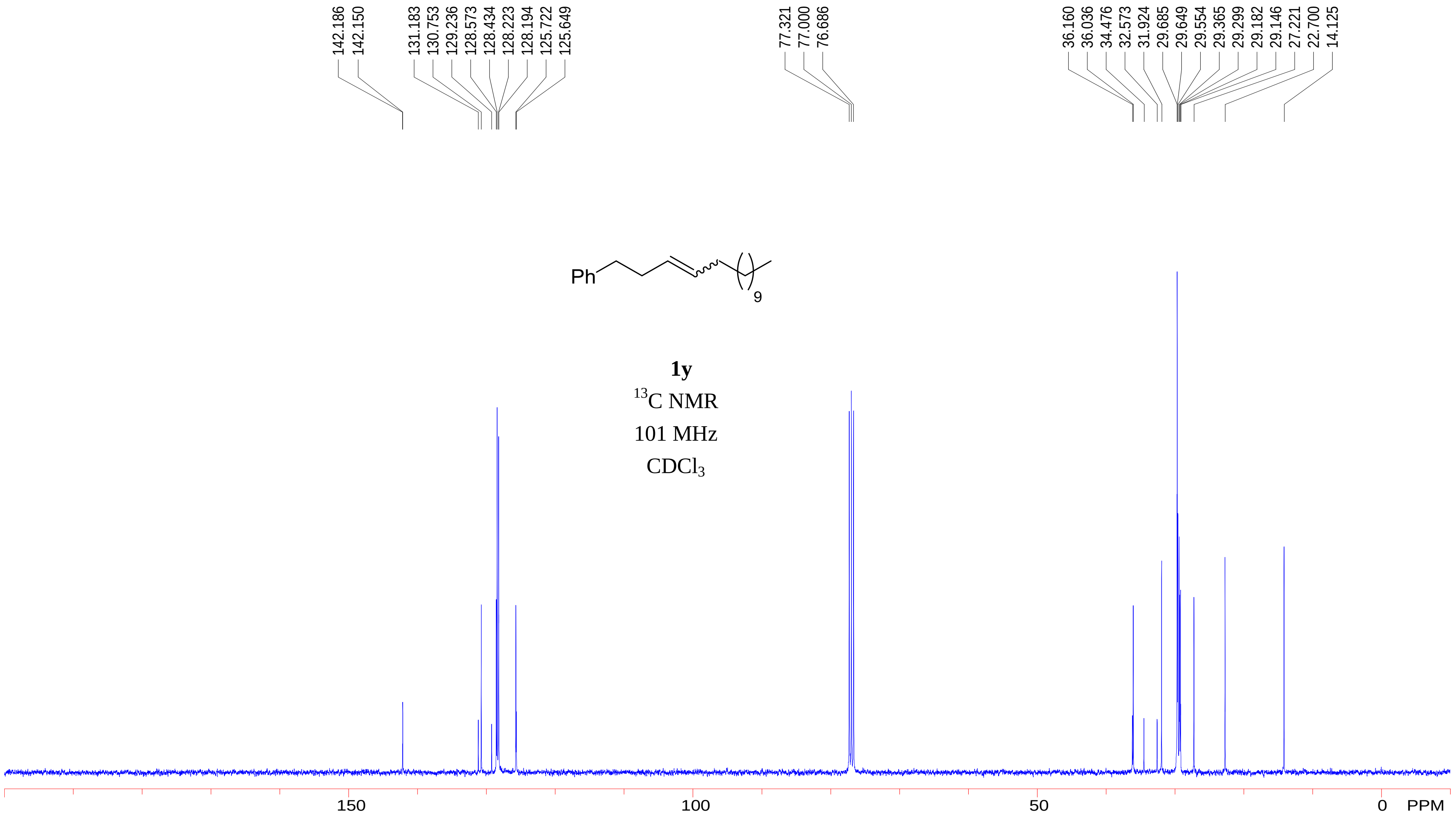

Supplementary Figure 95. ^{13}C NMR spectrum for **1y**

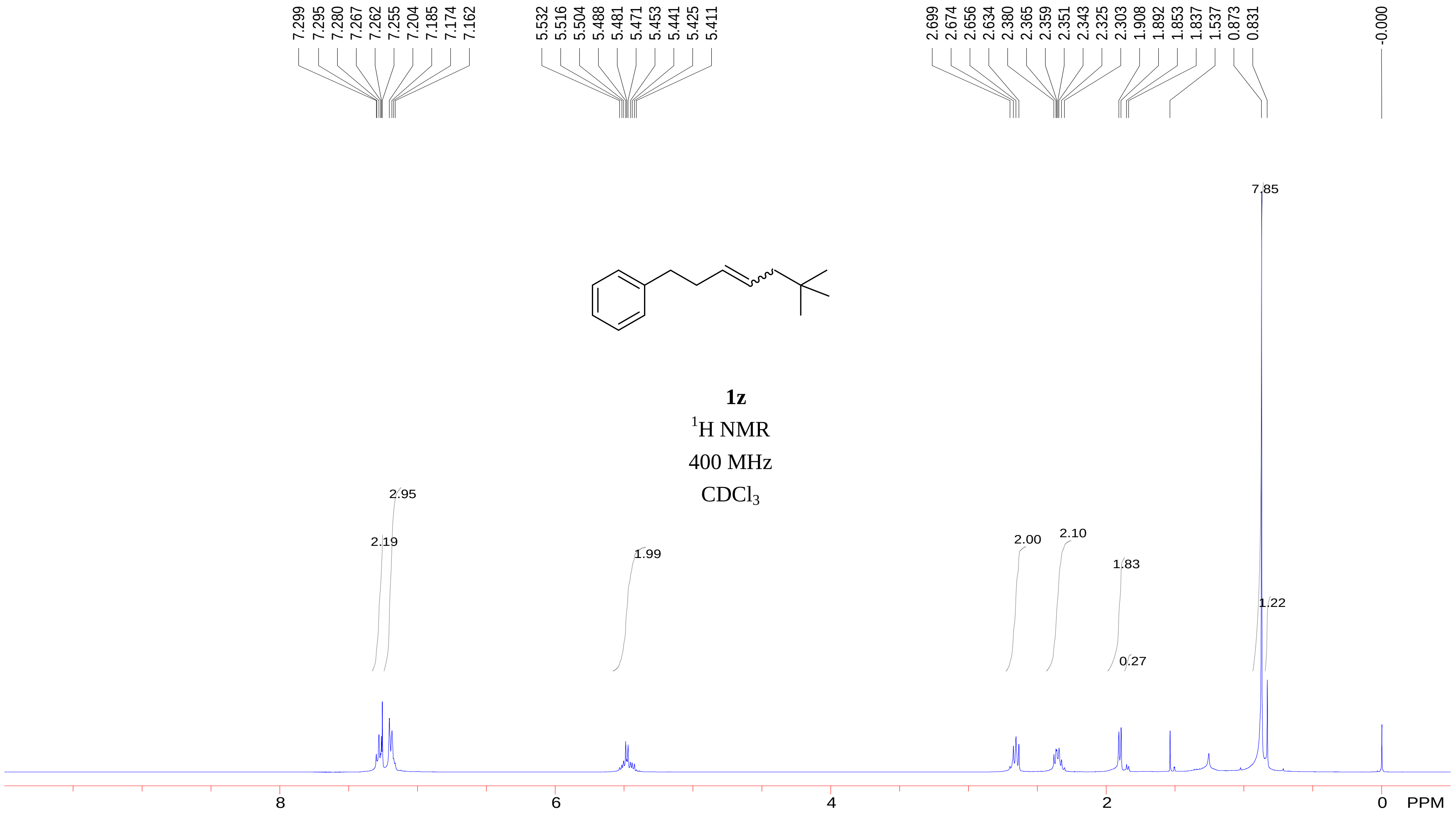

Supplementary Figure 96. ^{1}H NMR spectrum for **1z**

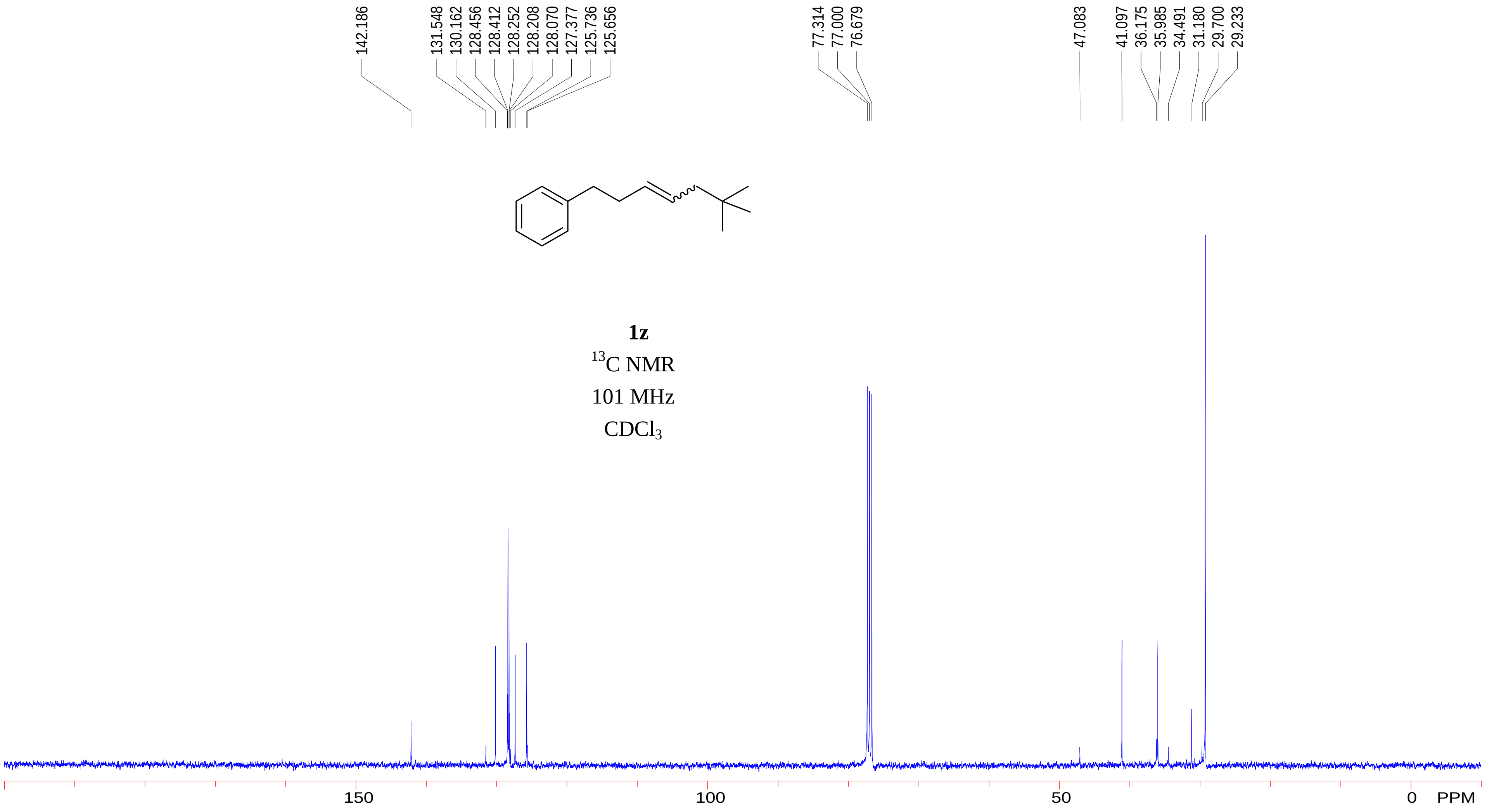

Supplementary Figure 97. ^{13}C NMR spectrum for **1z**

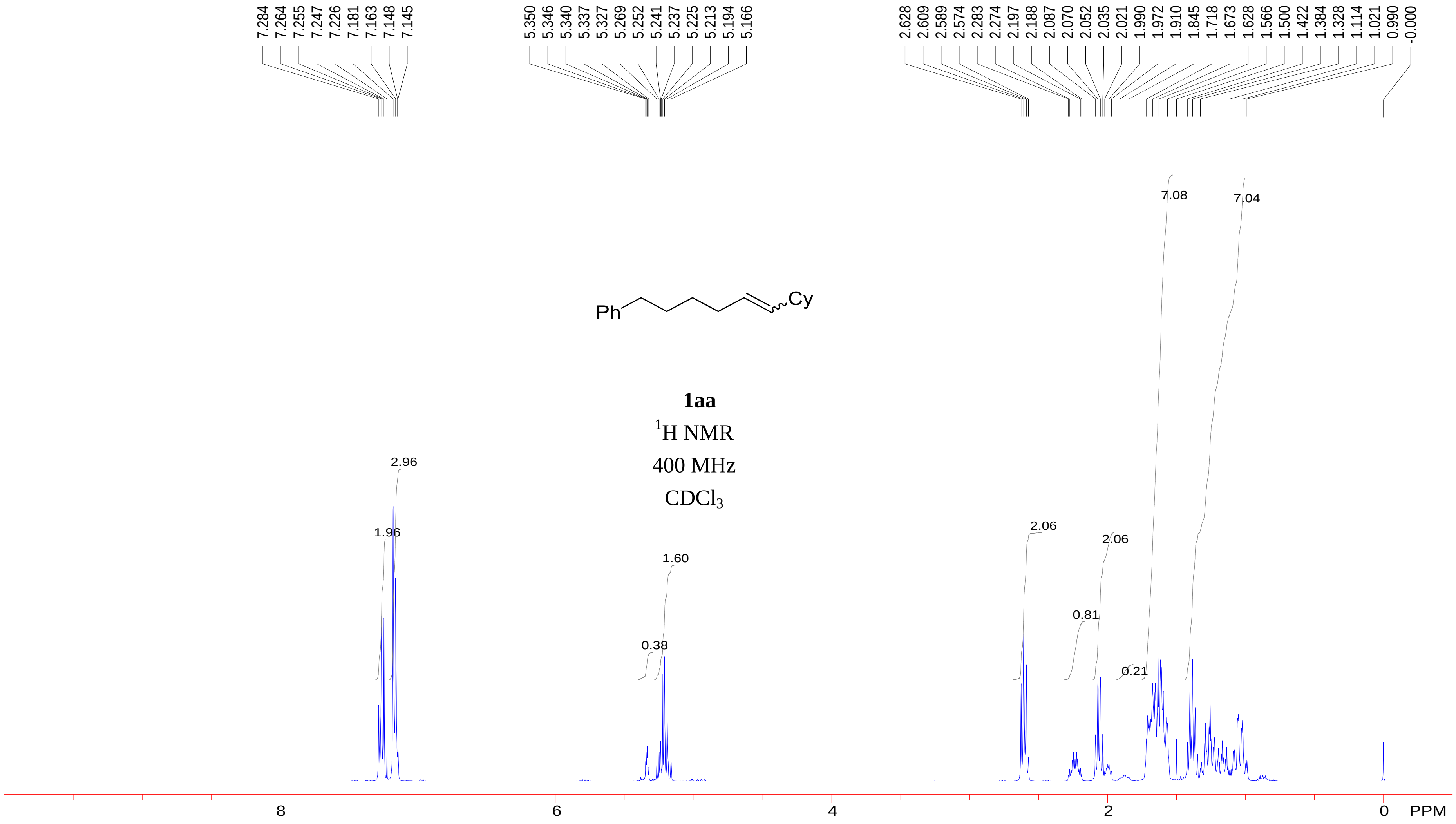

Supplementary Figure 98. ^{1}H NMR spectrum for **1aa**

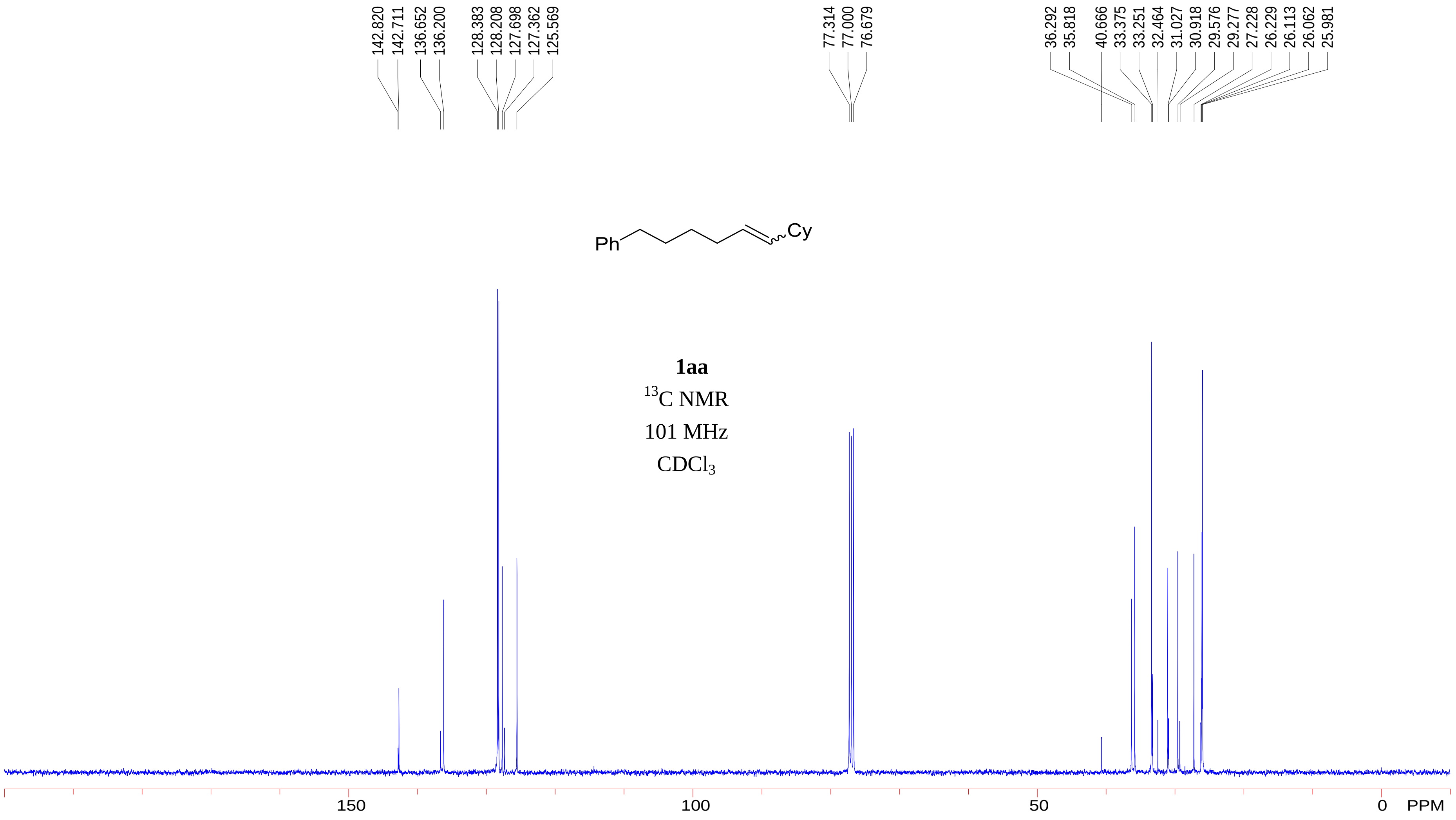

Supplementary Figure 99. ^{13}C NMR spectrum for **1aa**

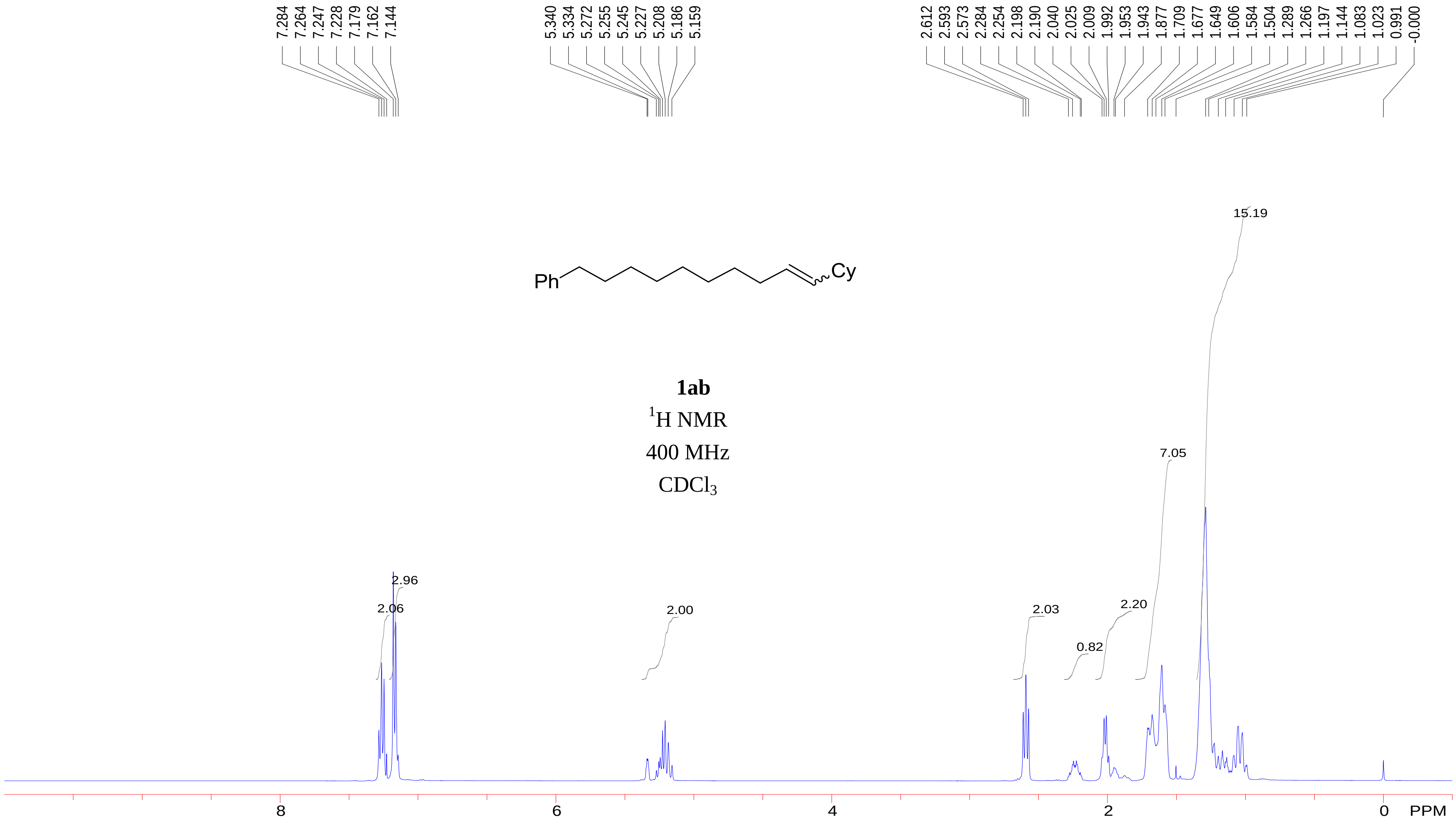

Supplementary Figure 100. ^{1}H NMR spectrum for **1ab**

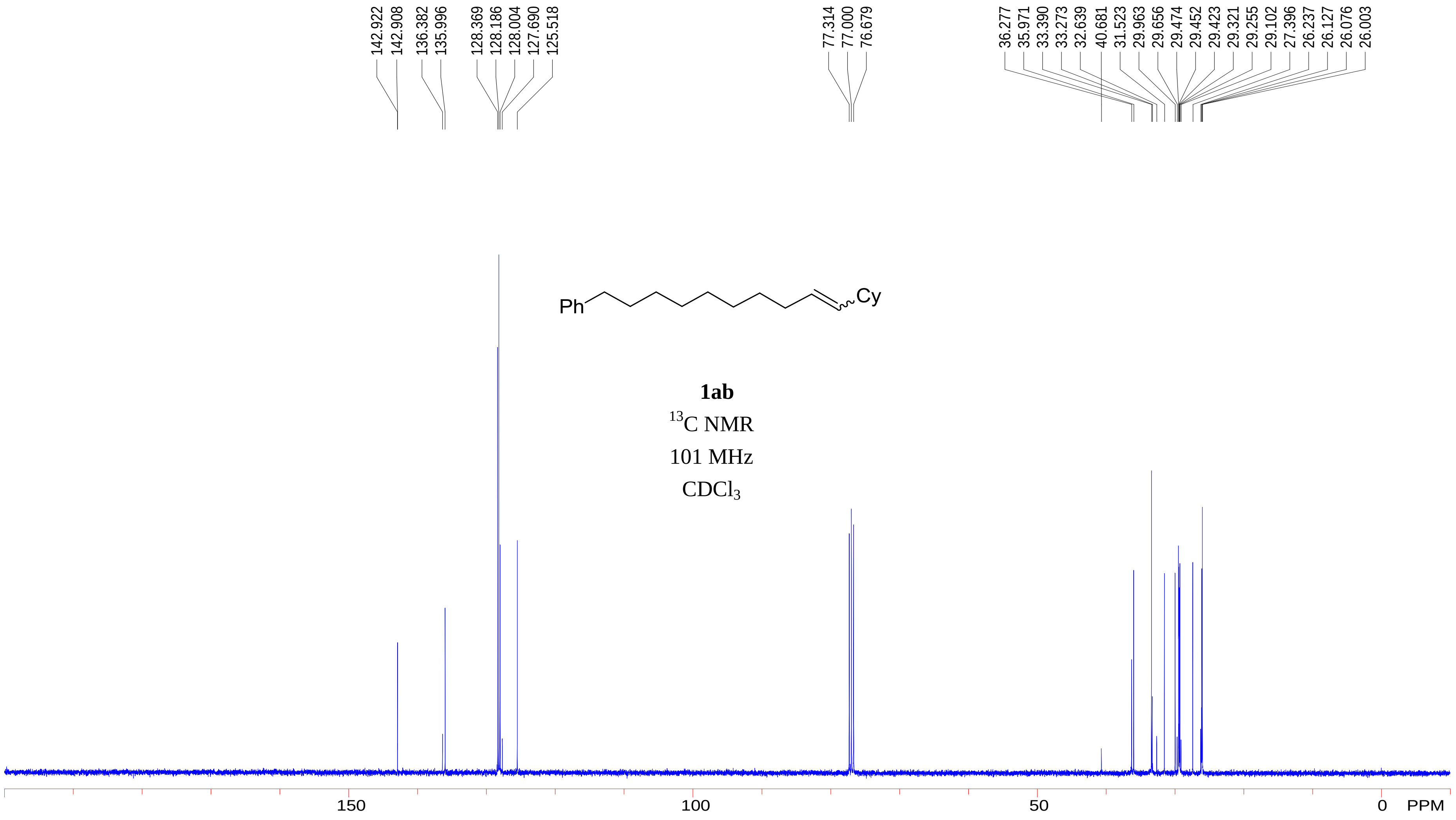

Supplementary Figure 101. ^{13}C NMR spectrum for **1ab**

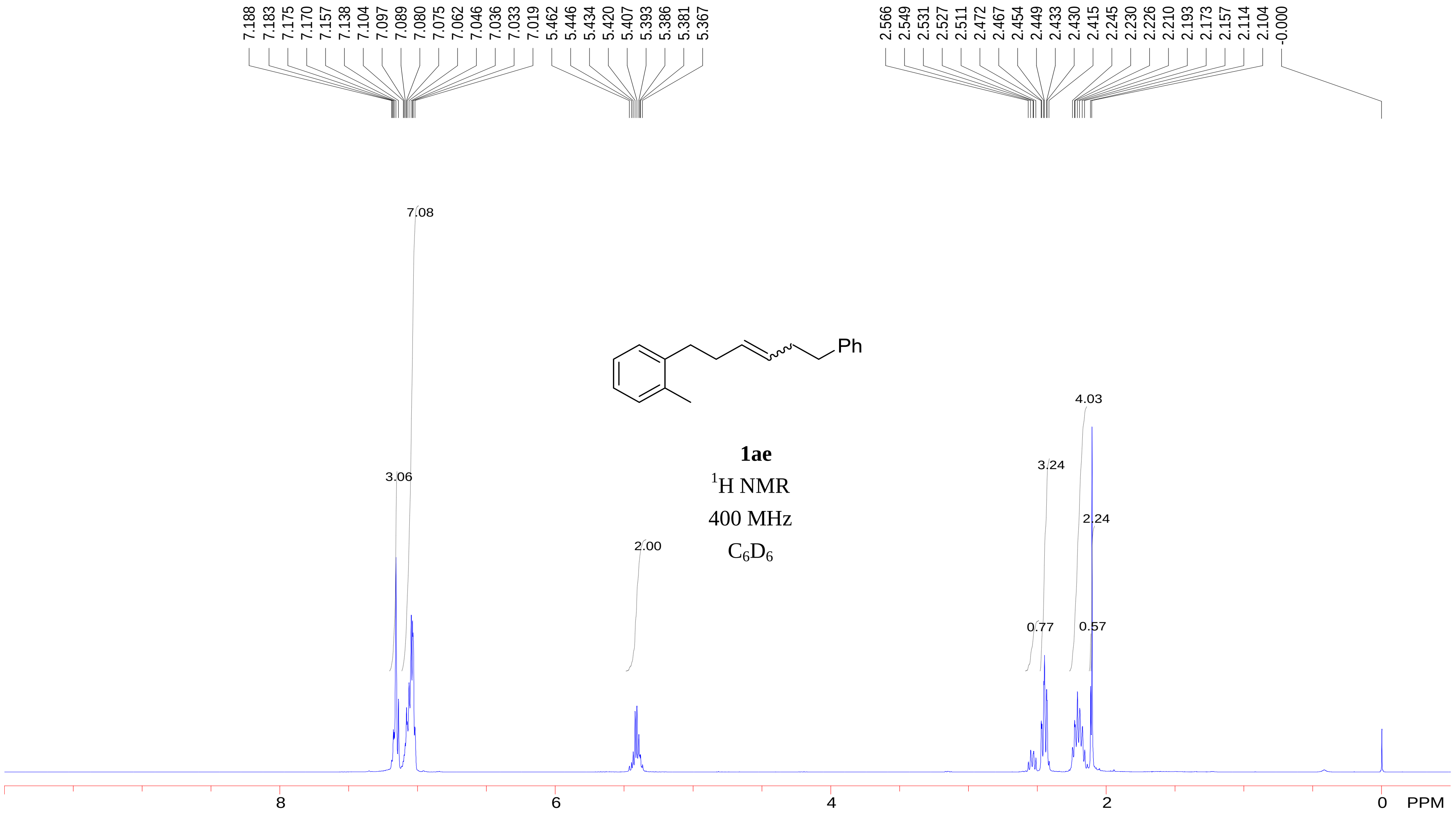

Supplementary Figure 102. ^{1}H NMR spectrum for **1ae**

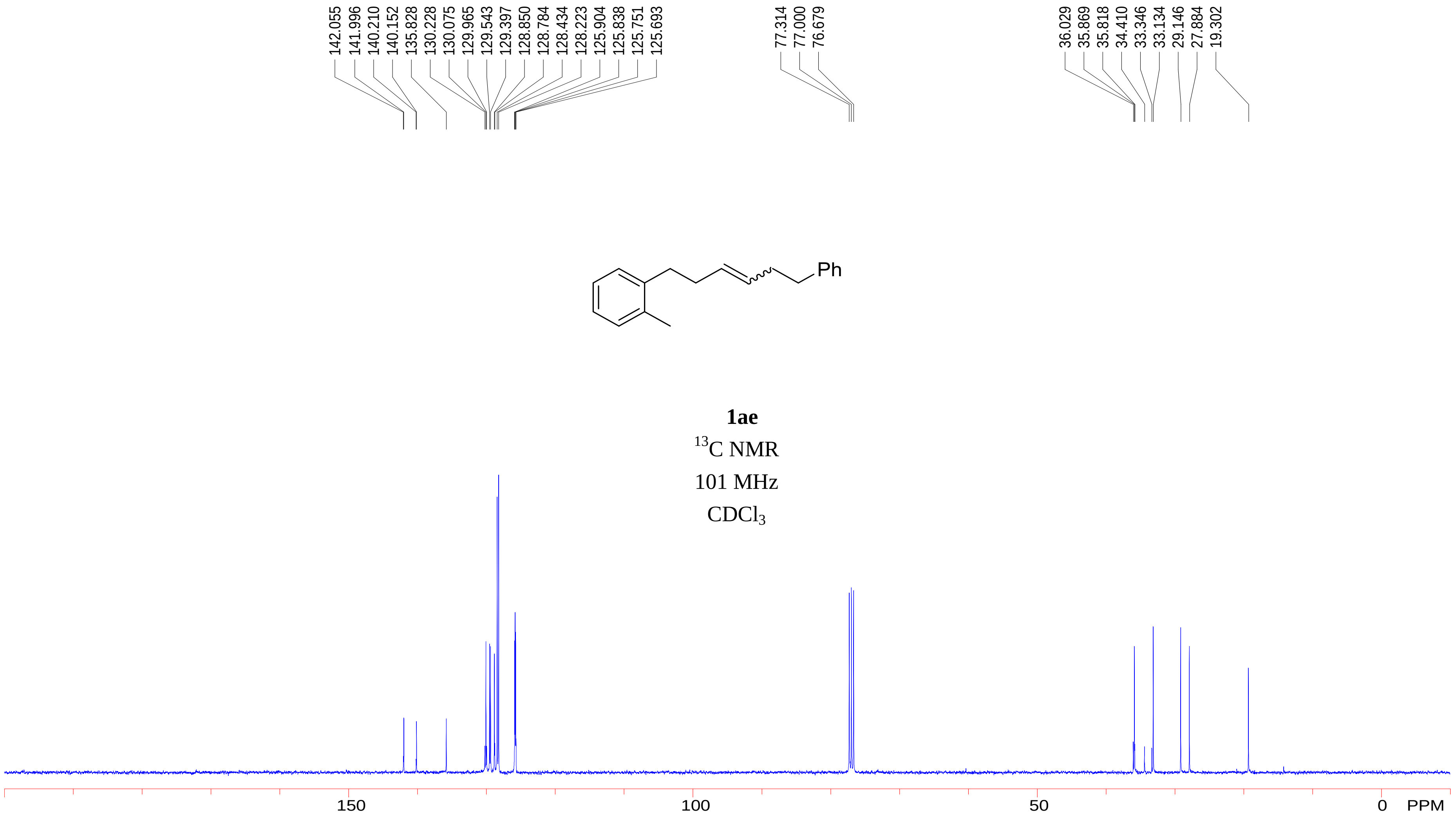

Supplementary Figure 103. ^{13}C NMR spectrum for **1ae**

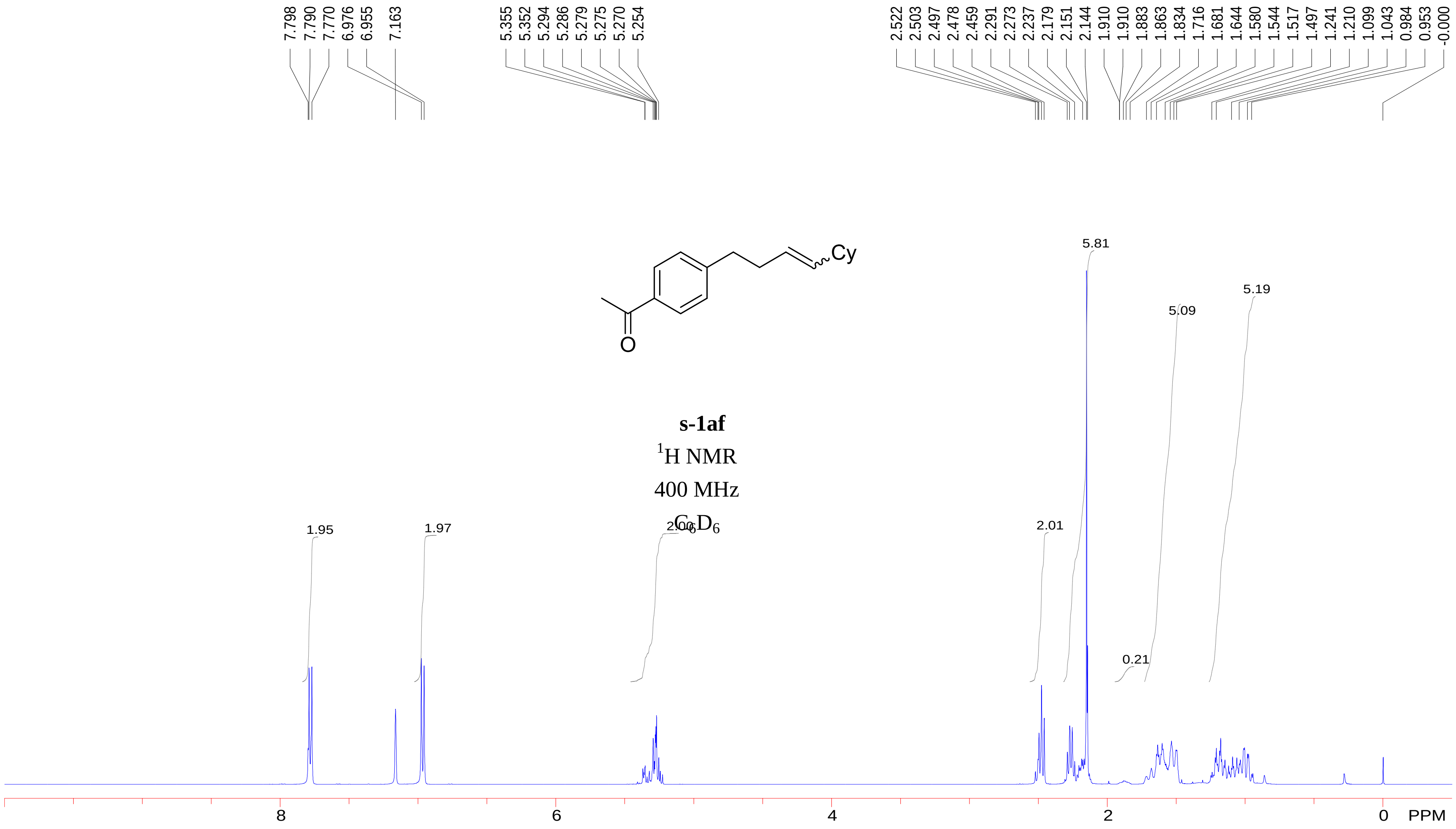

Supplementary Figure 104. ^{1}H NMR spectrum for **s-1af**

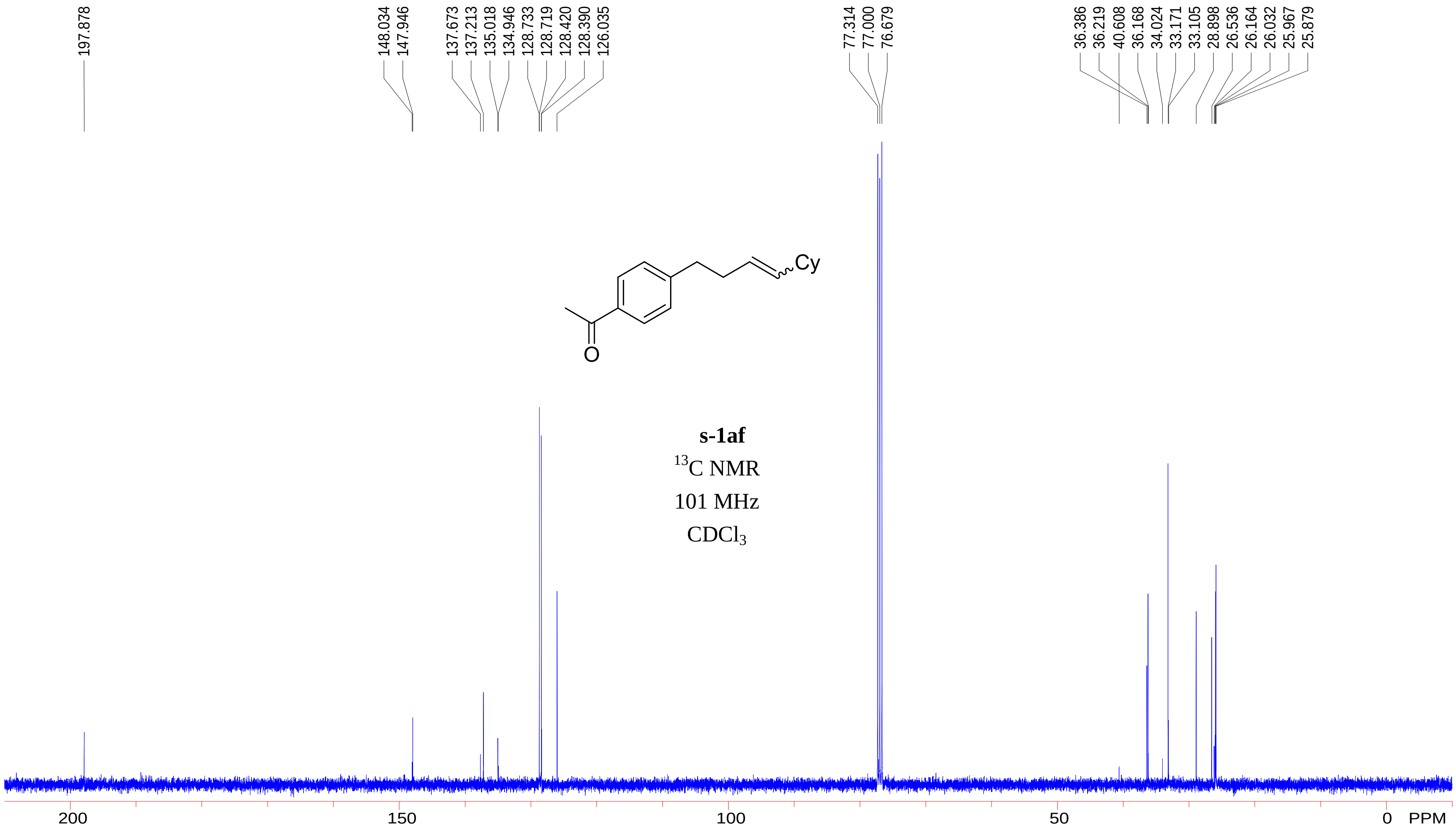

Supplementary Figure 105. ^{13}C NMR spectrum for **s-1af**

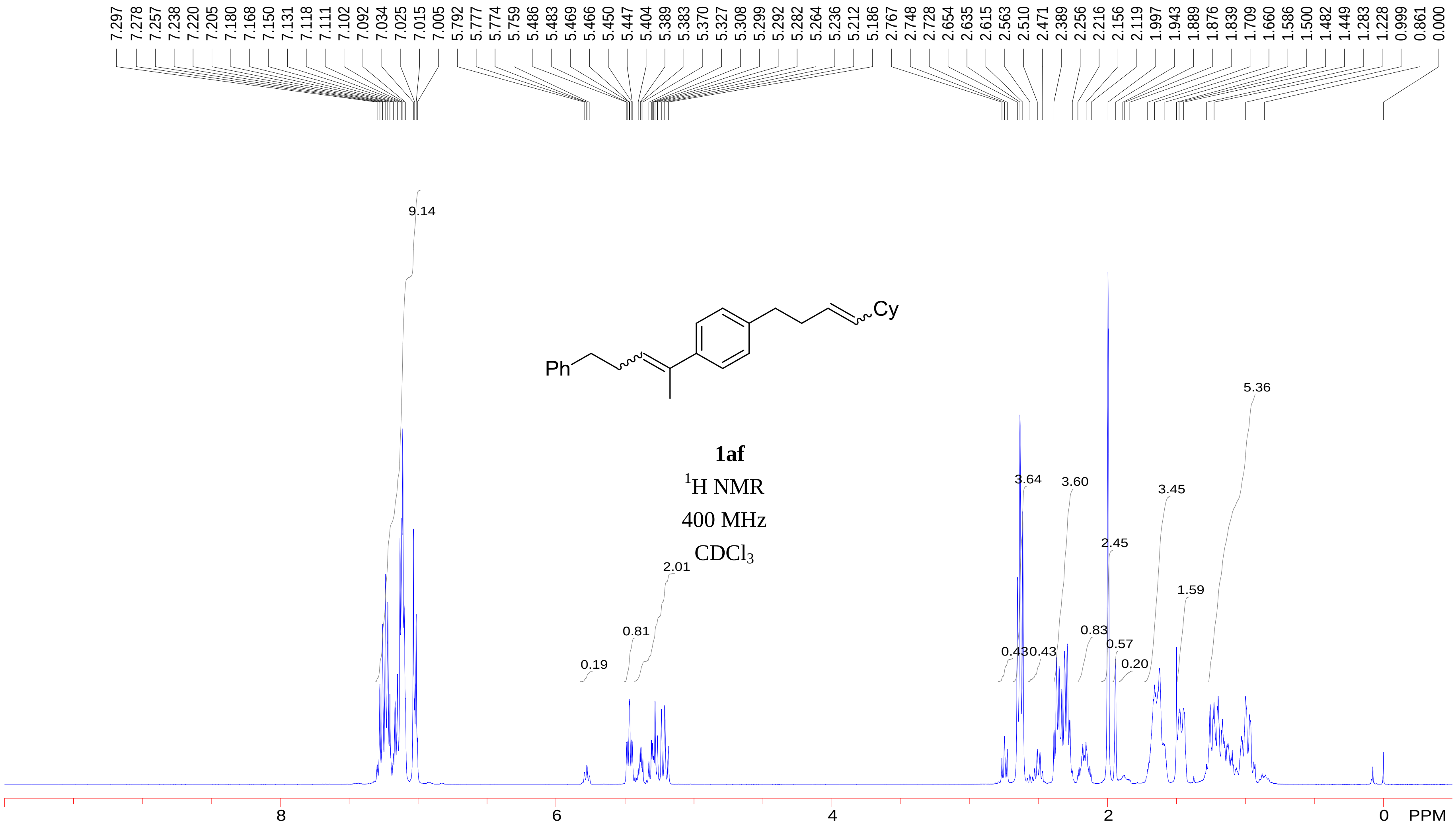

Supplementary Figure 106. ^{1}H NMR spectrum for **1af**

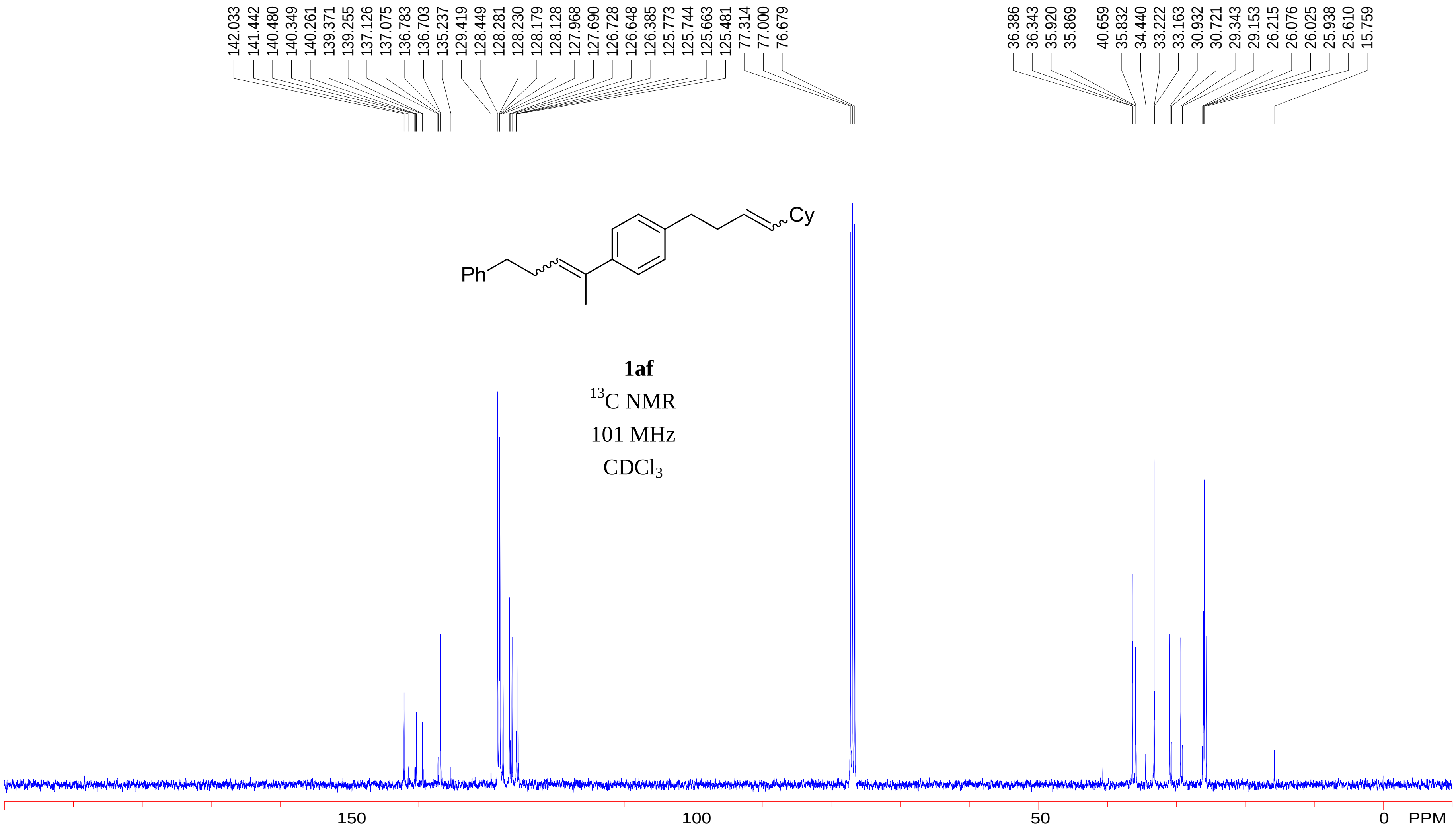

Supplementary Figure 107. ^{13}C NMR spectrum for **1af**

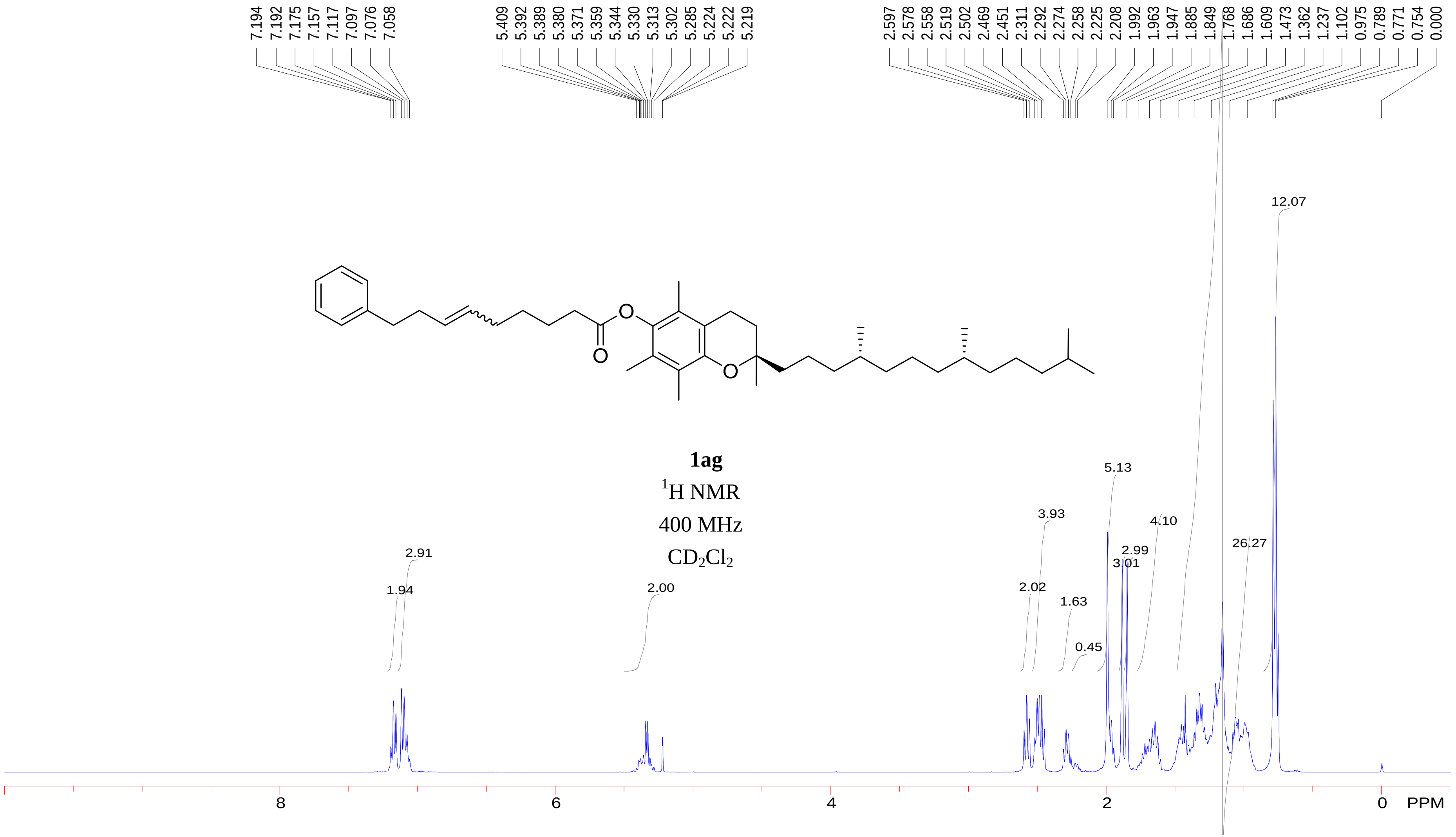

Supplementary Figure 108. ^{1}H NMR spectrum for **1ag**

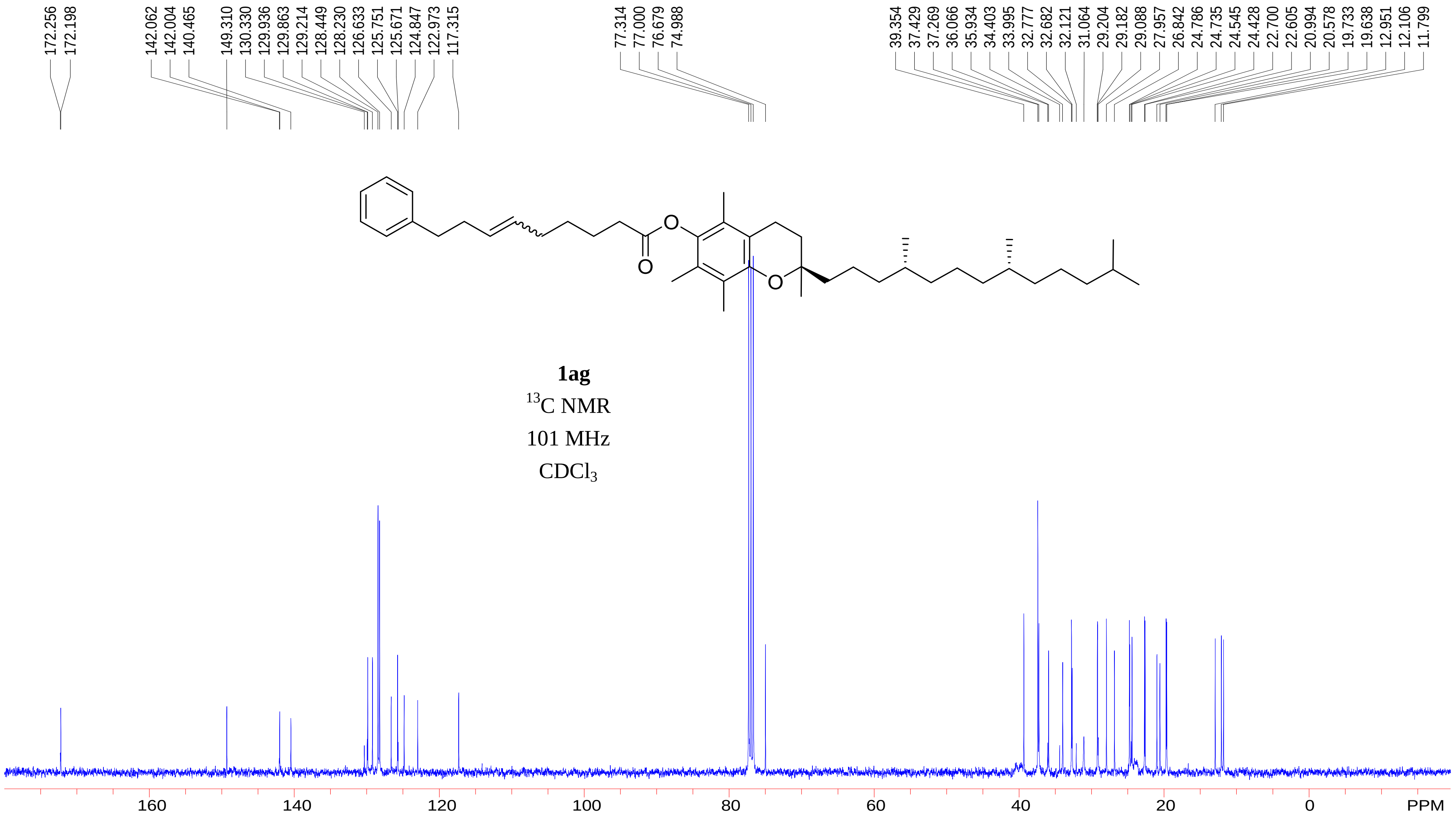

Supplementary Figure 103. ^{13}C NMR spectrum for **1ag**

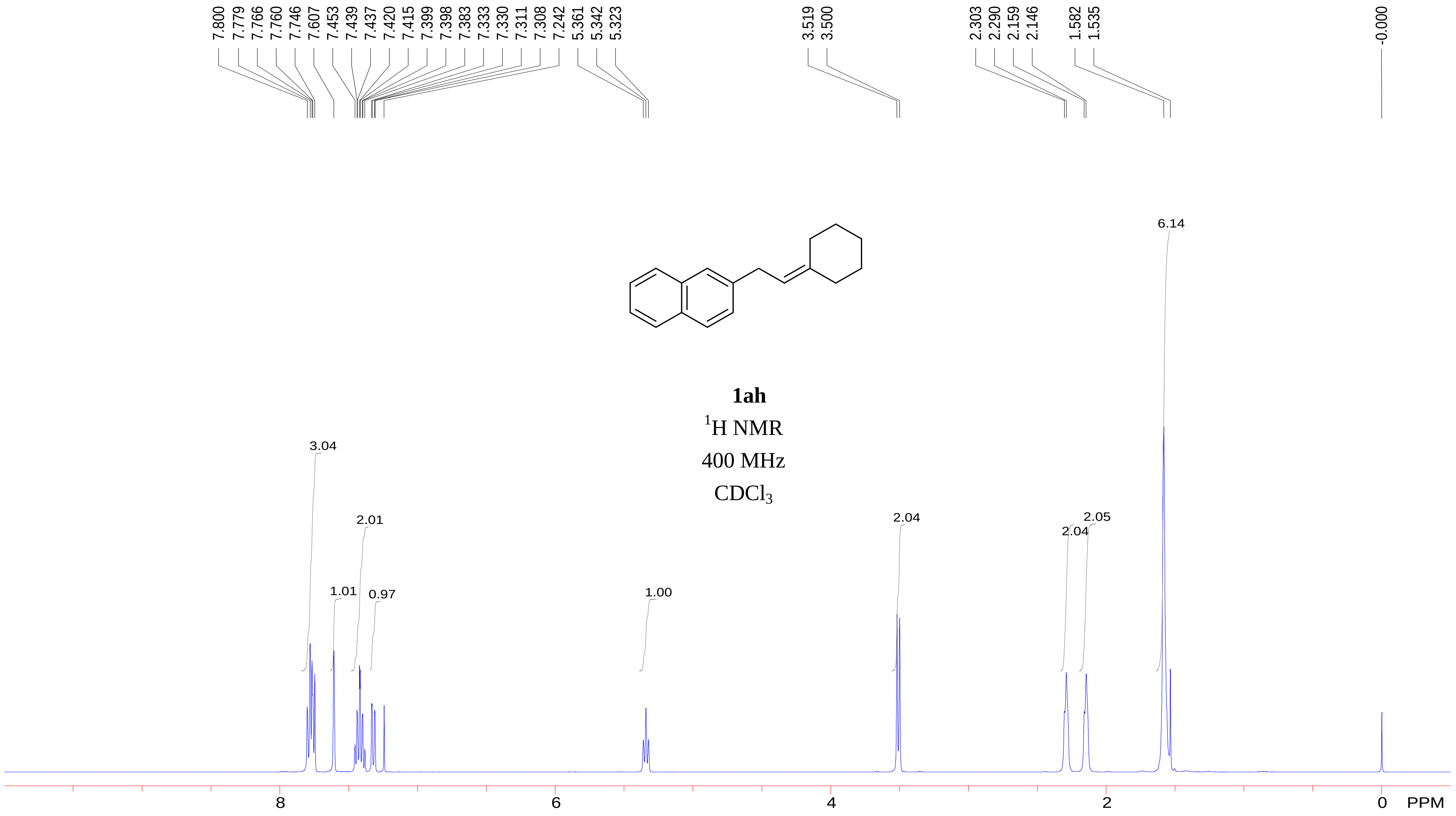

Supplementary Figure 110. ^{1}H NMR spectrum for **1ah**

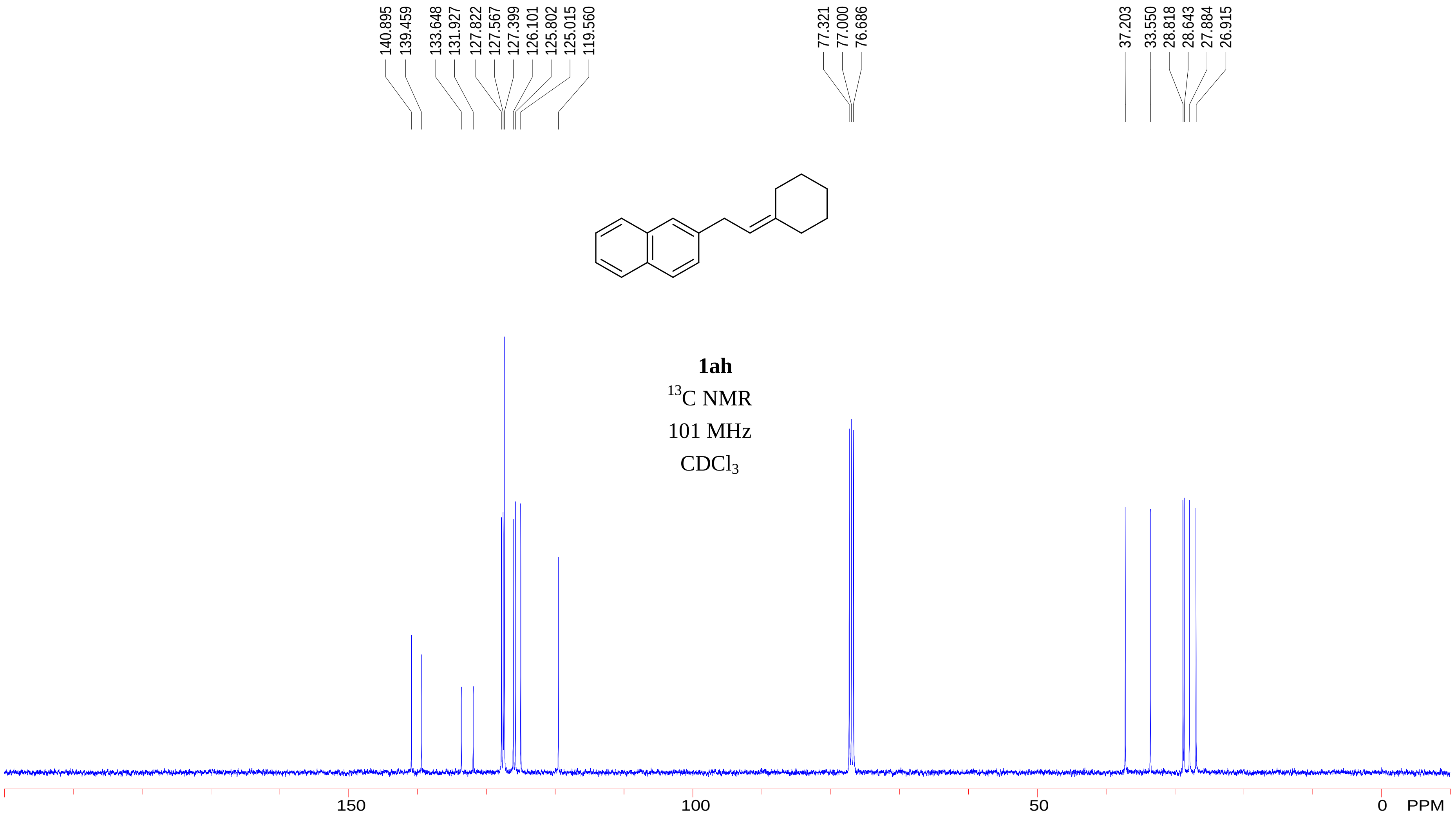

Supplementary Figure 111. ^{13}C NMR spectrum for **1ah**

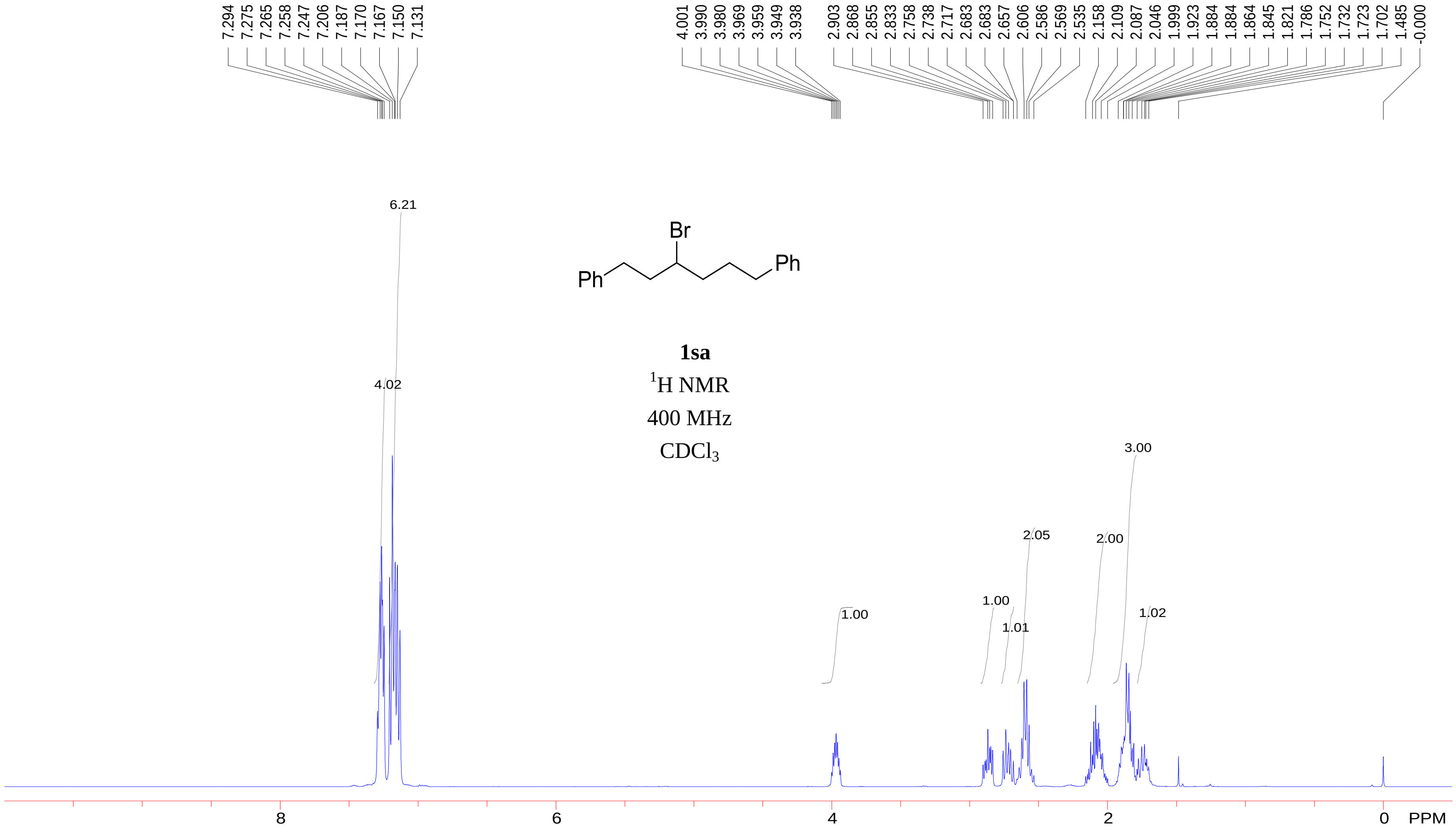

Supplementary Figure 112. ^{1}H NMR spectrum for **1sa**

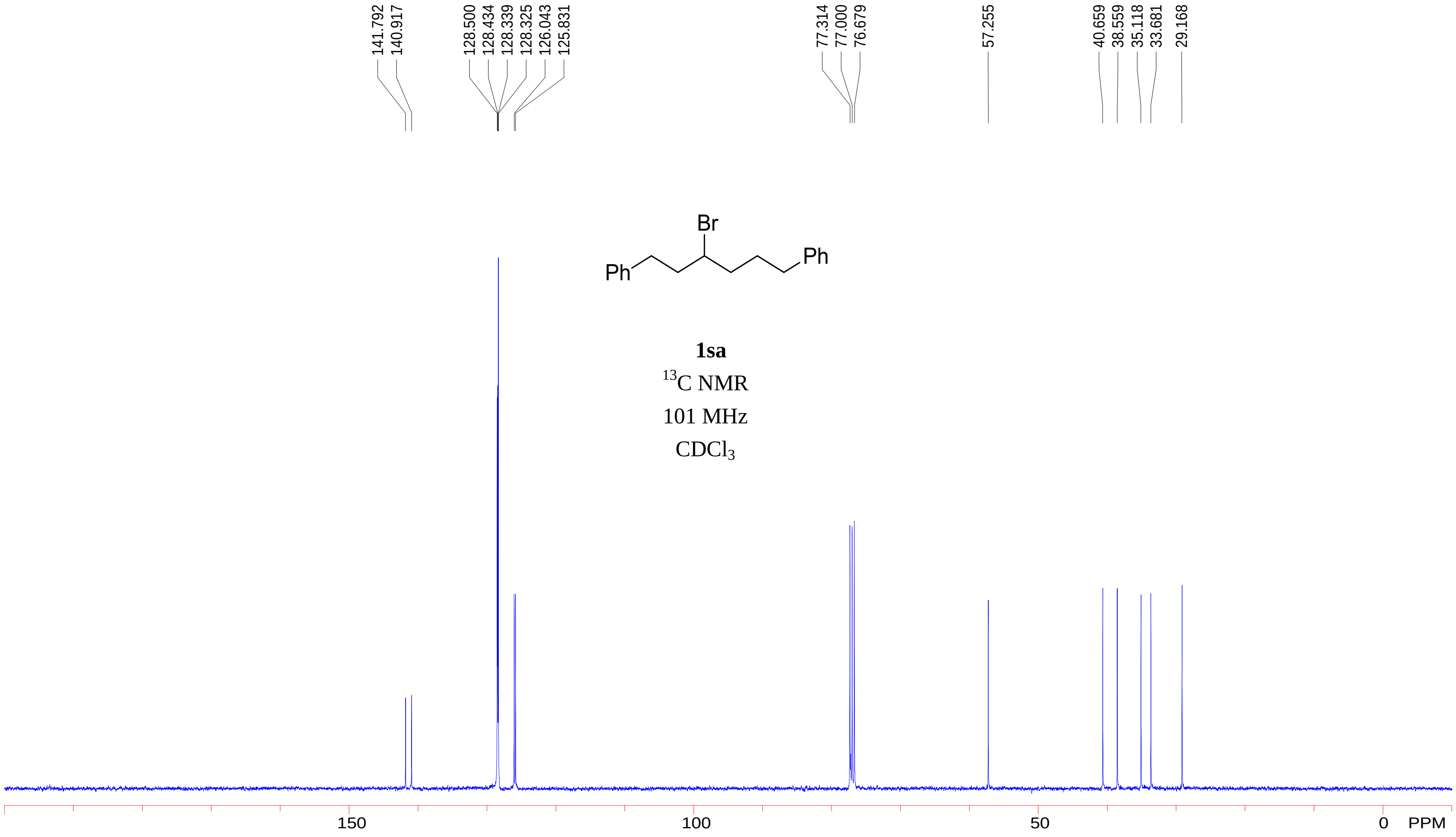

Supplementary Figure 113. ^{13}C NMR spectrum for **1sa**

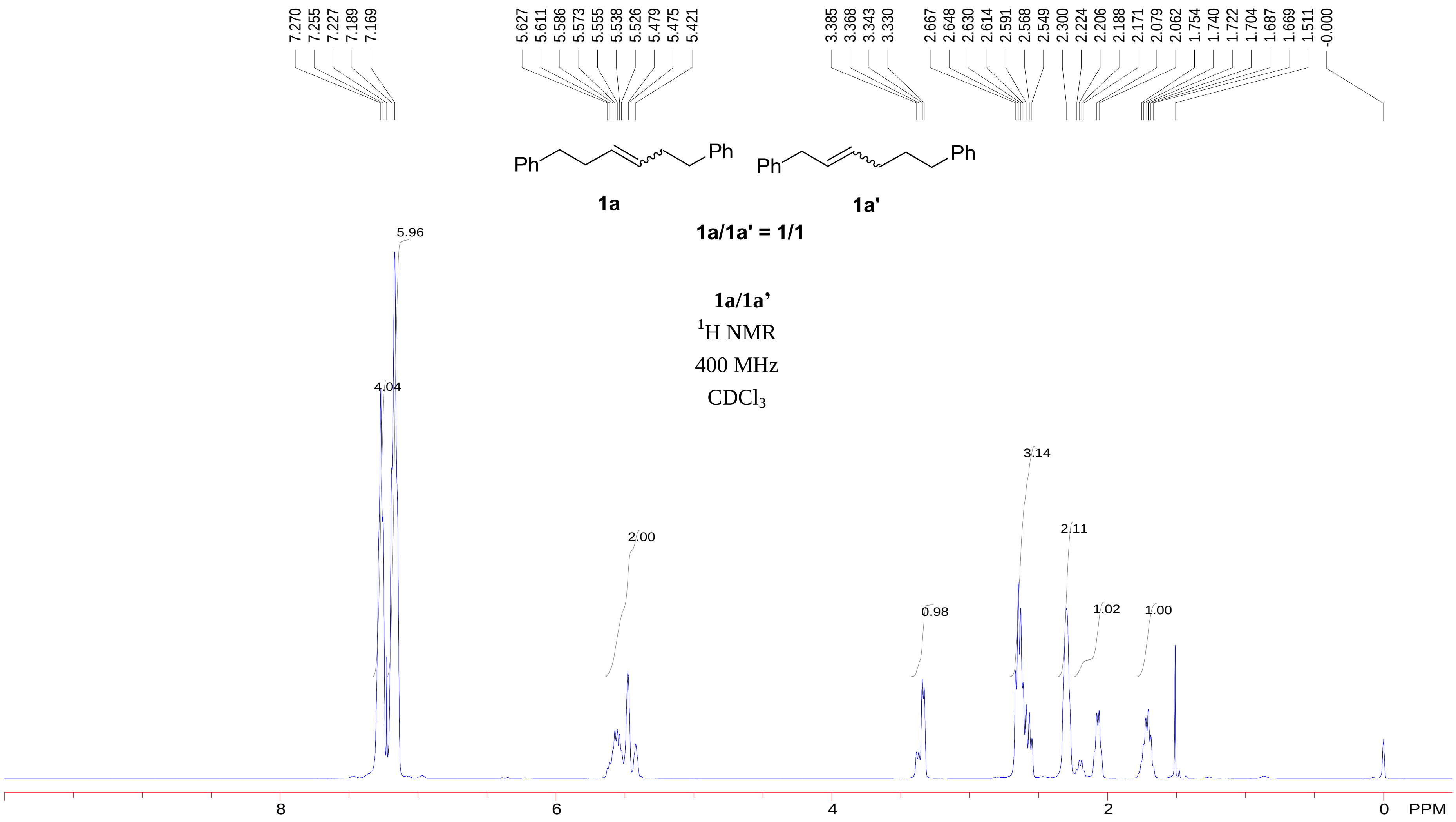

Supplementary Figure 114. ^{1}H NMR spectrum for **1a/1a’**

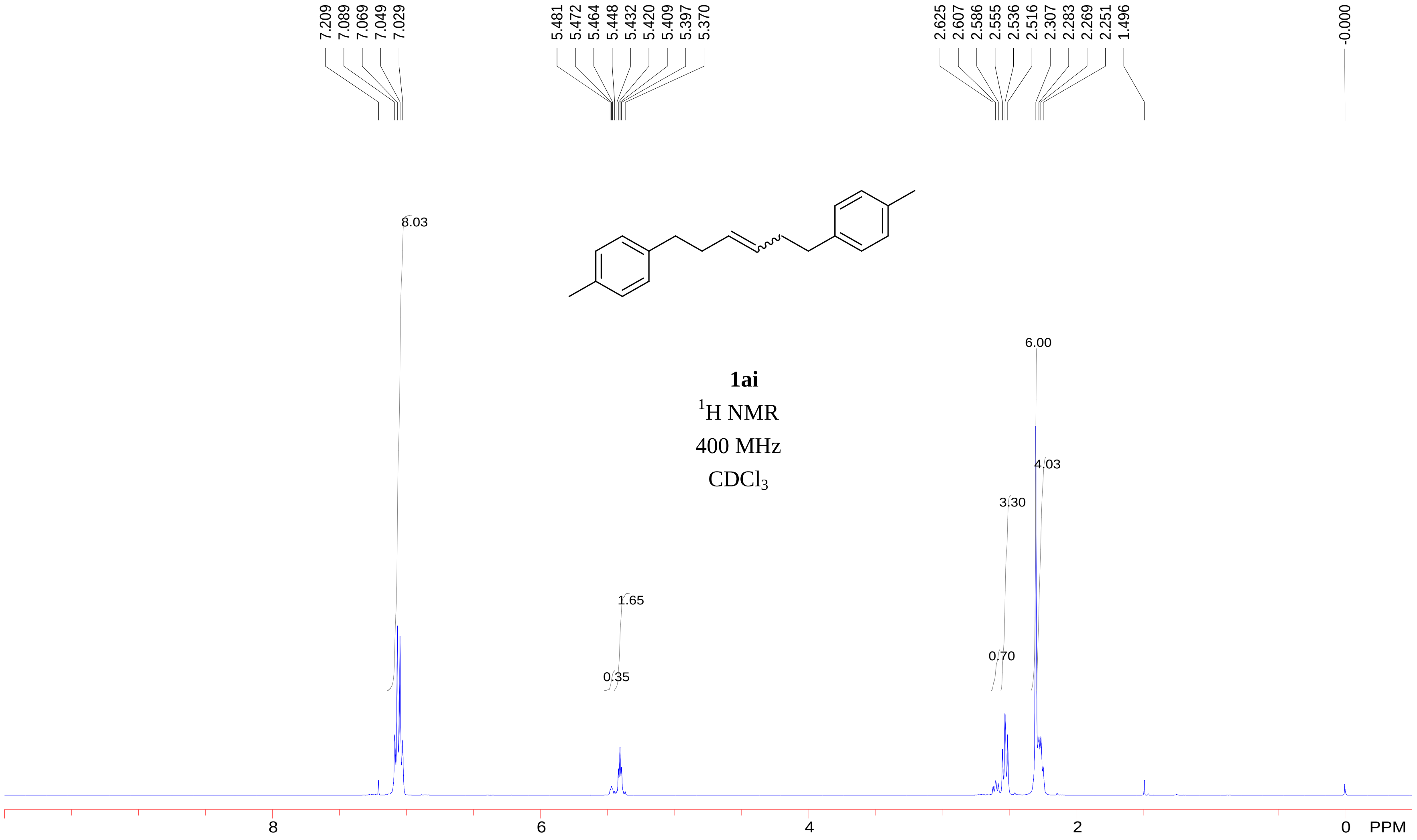

Supplementary Figure 115. ^{1}H NMR spectrum for **1ai**

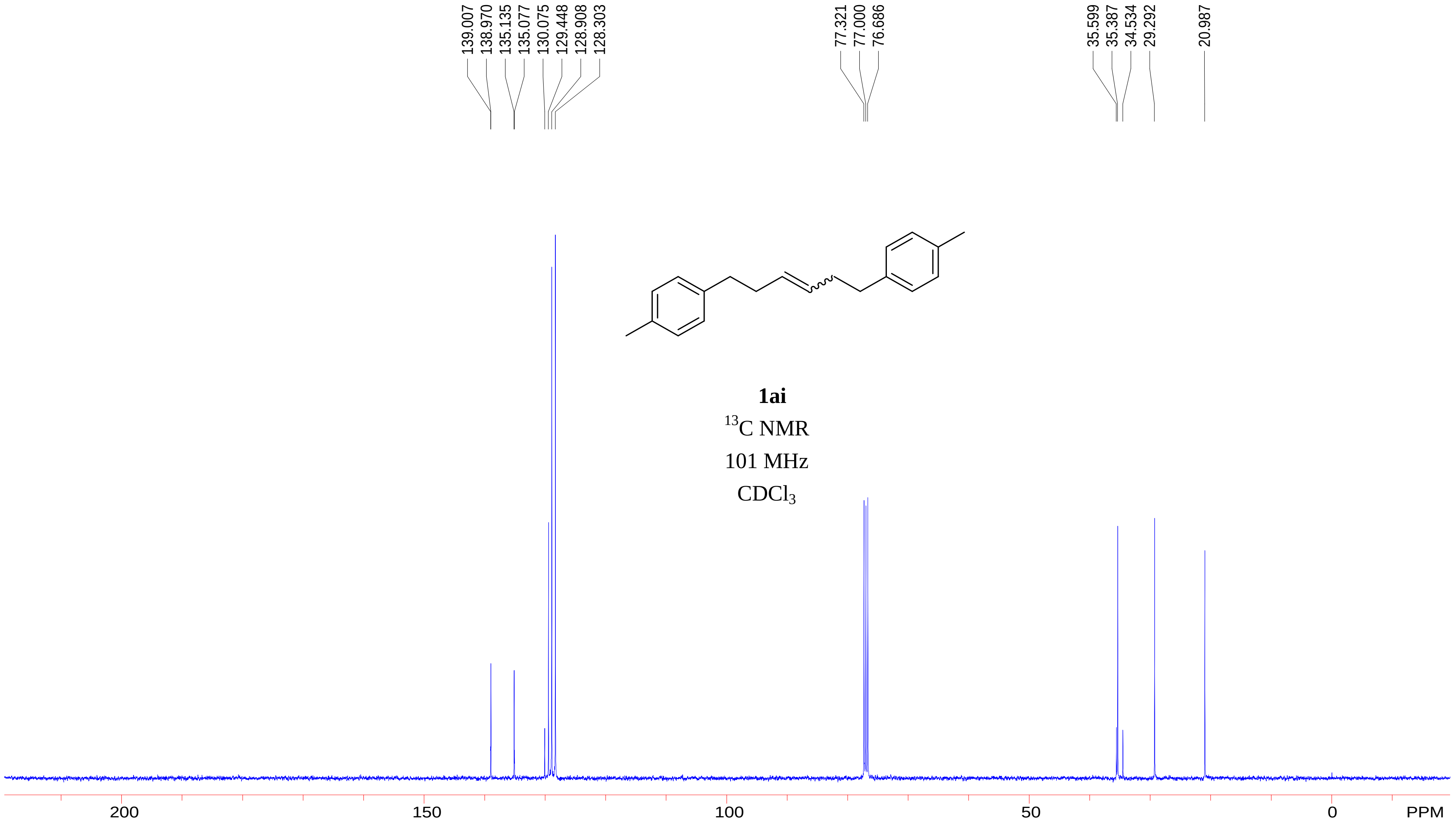

Supplementary Figure 116. ^{13}C NMR spectrum for **1ai**

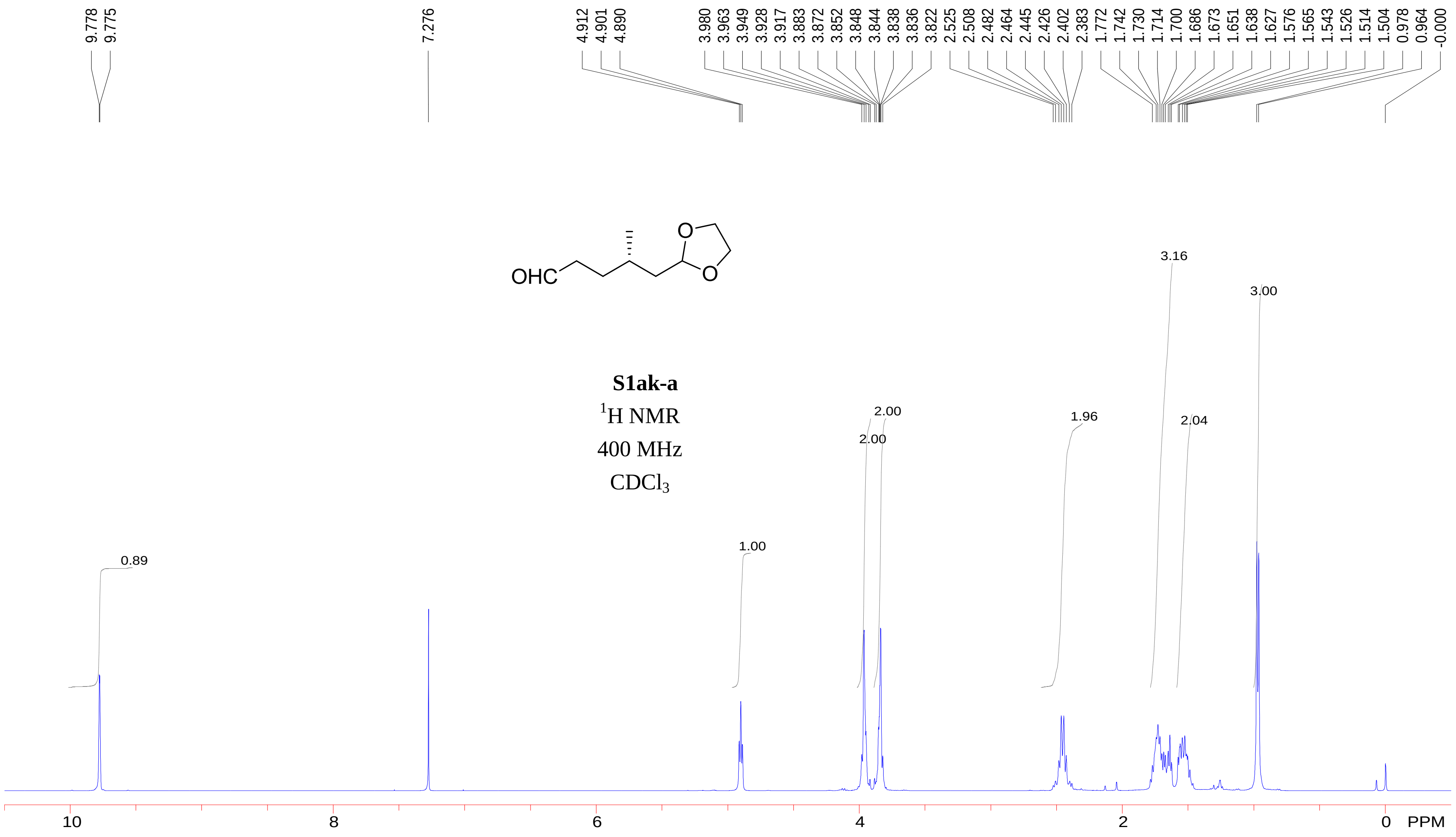

Supplementary Figure 117. ^{1}H NMR spectrum for **s1ak-a**

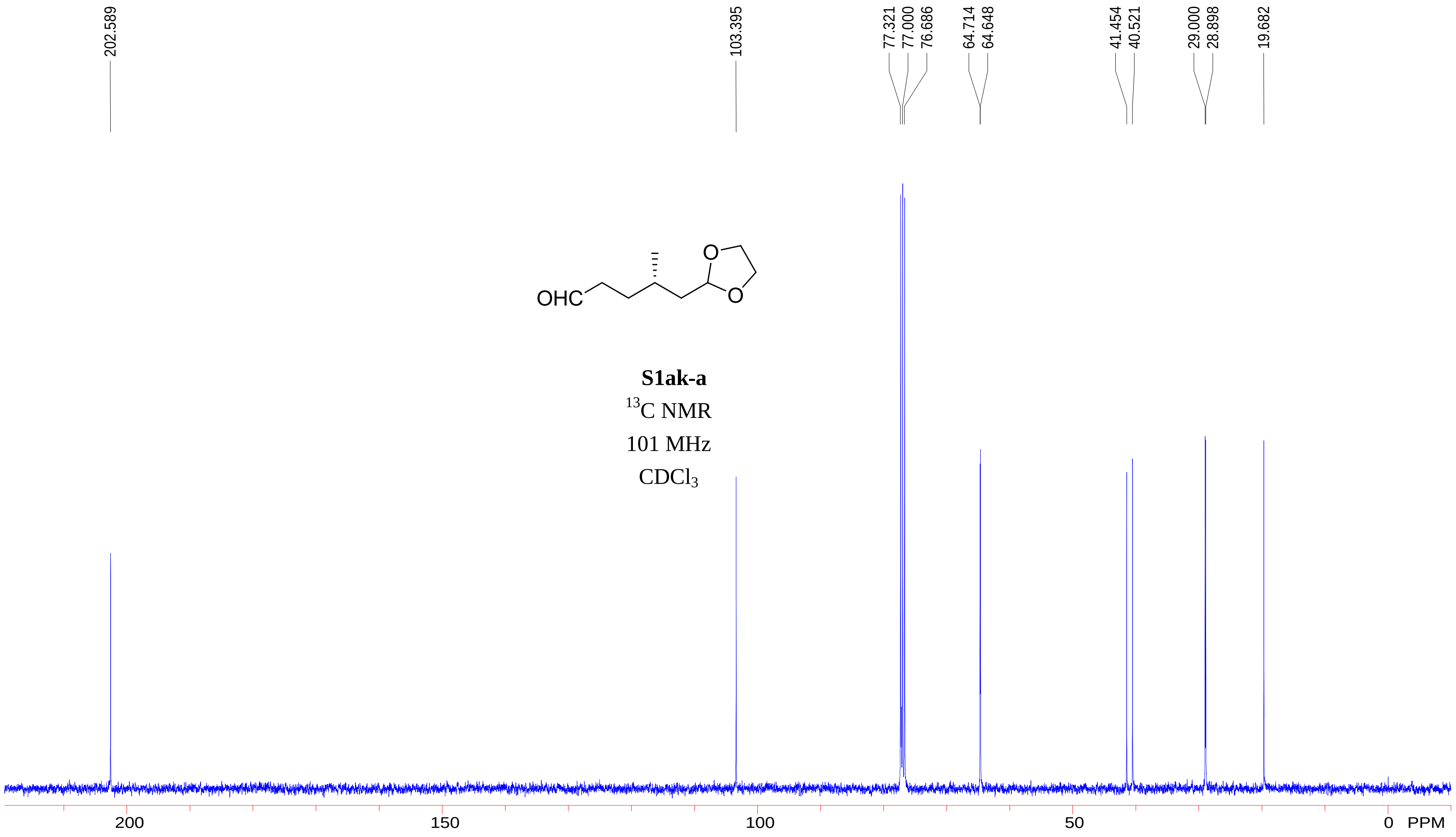

Supplementary Figure 118. ^{13}C NMR spectrum for **s1ak-a**

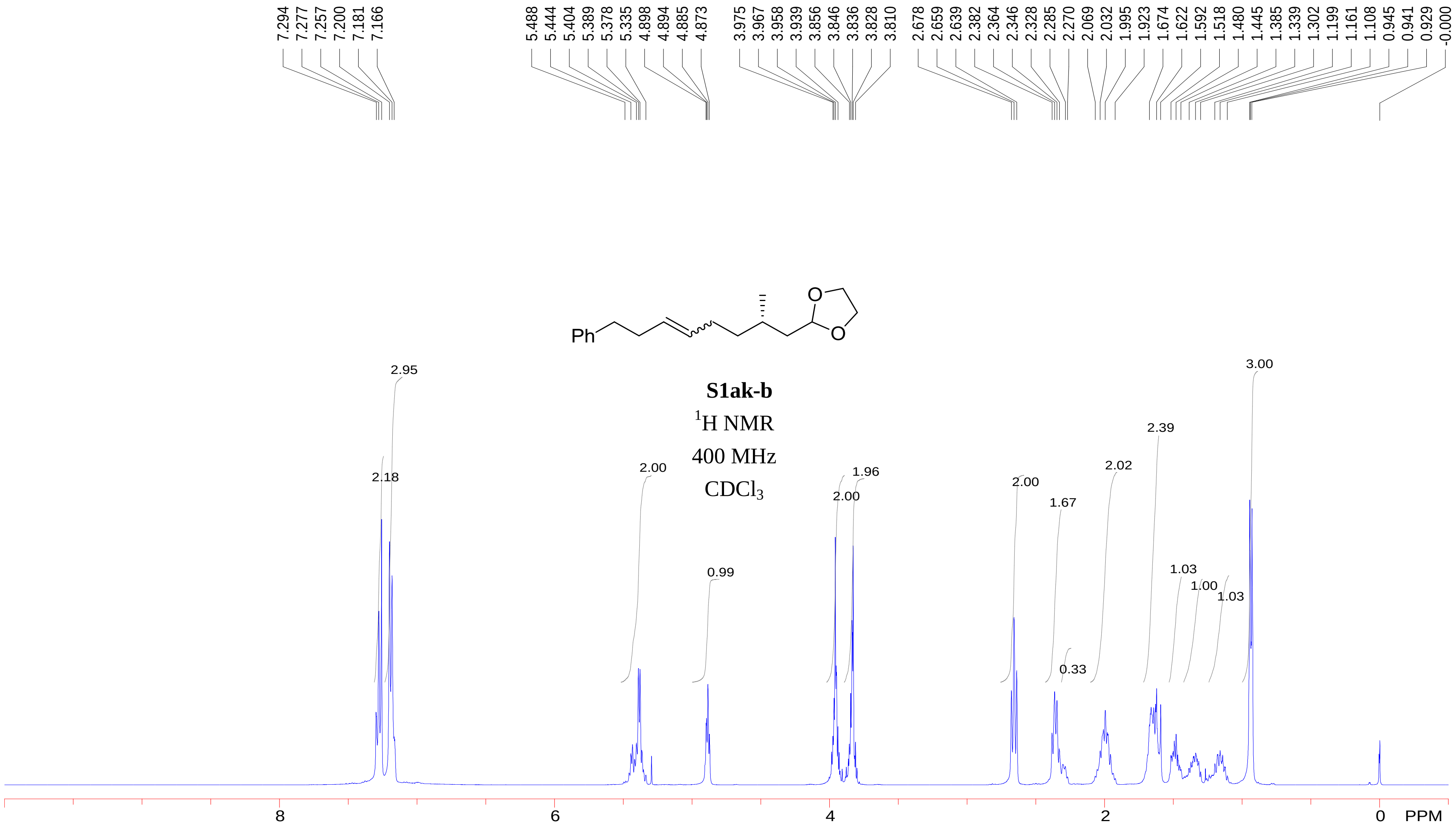

Supplementary Figure 119. ^{1}H NMR spectrum for **s1ak-b**

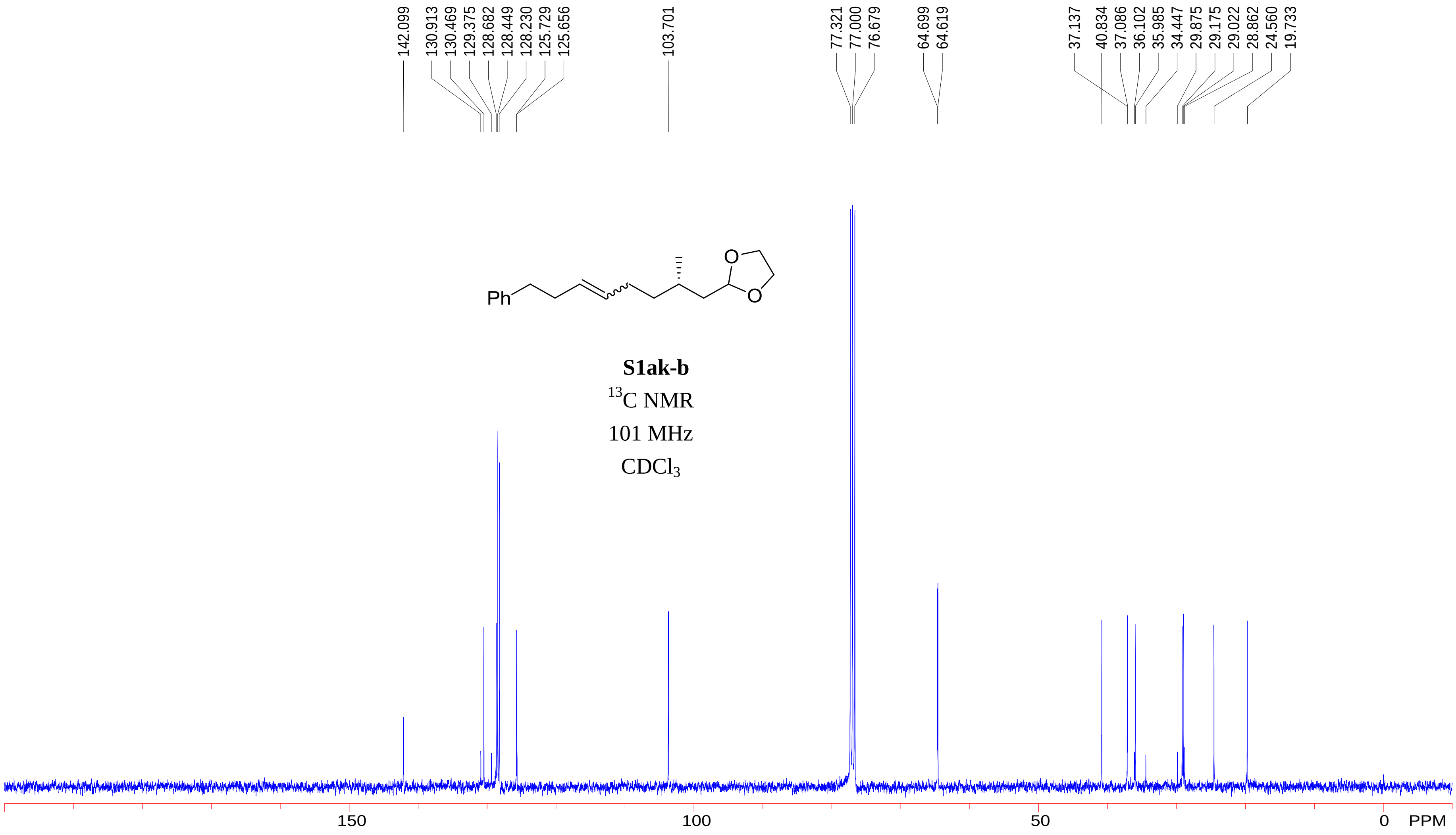

Supplementary Figure 120. ^{13}C NMR spectrum for **s1ak-b**

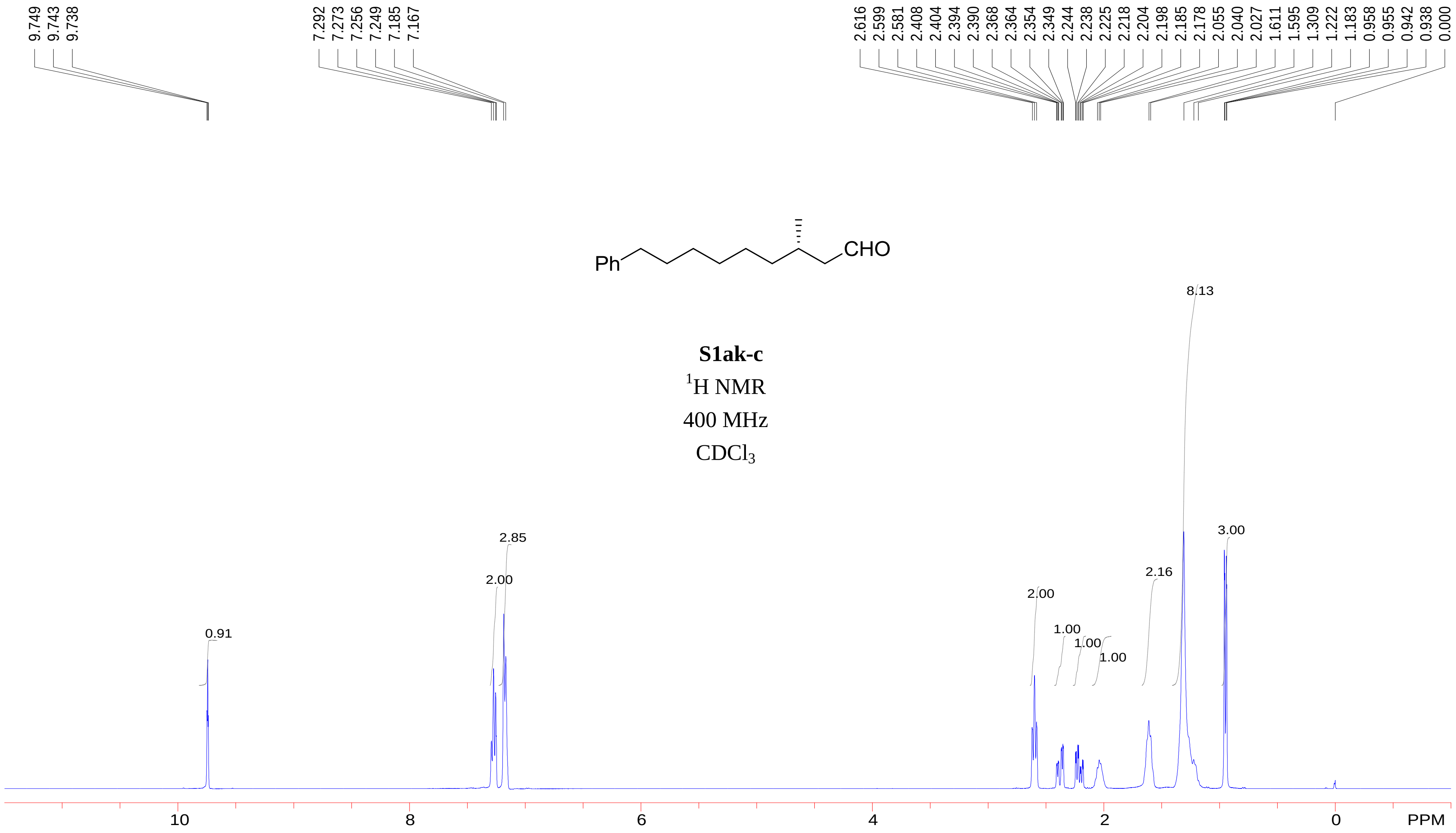

Supplementary Figure 121. ^{1}H NMR spectrum for **s1ak-c**

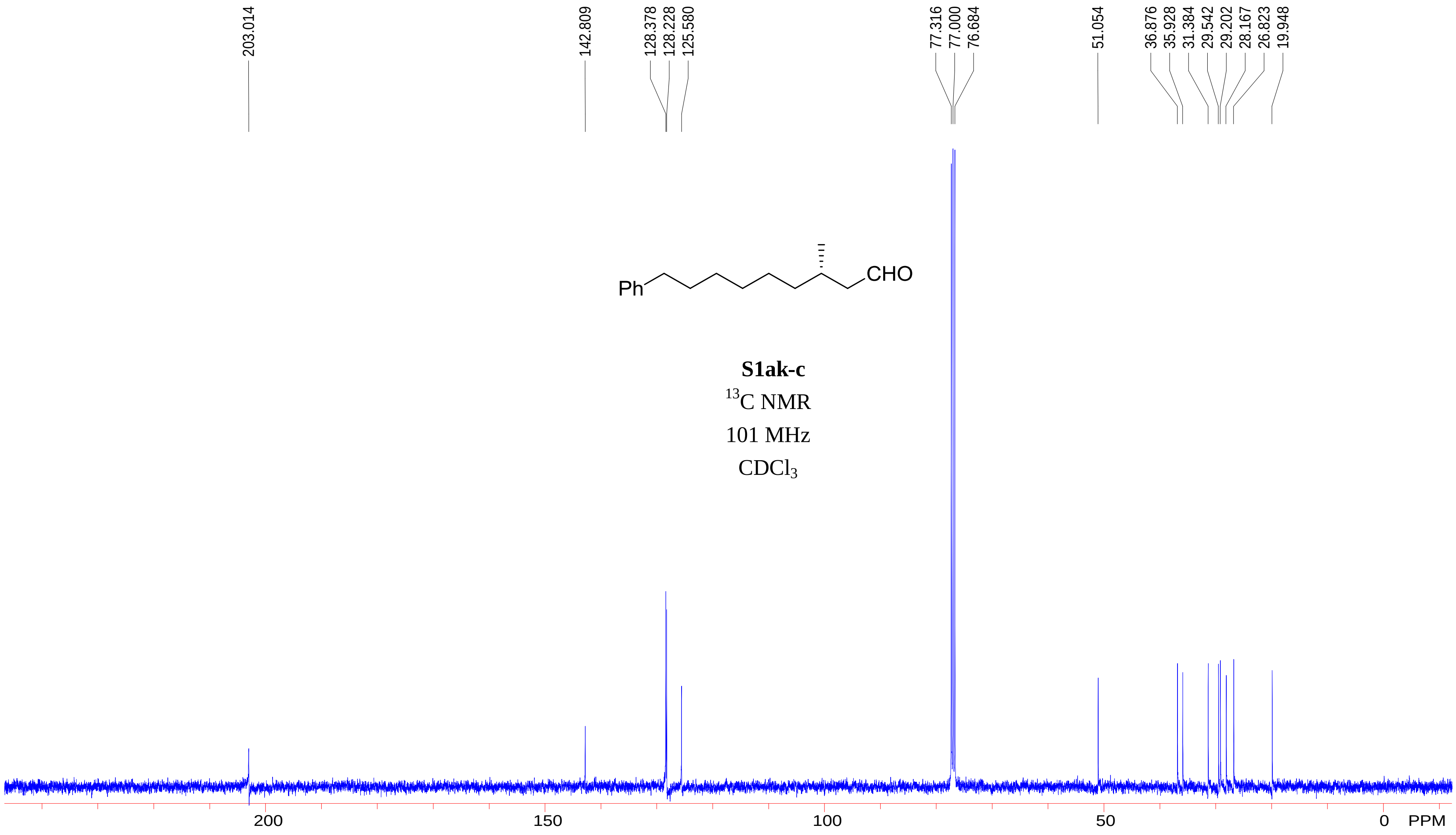

Supplementary Figure 122. ^{13}C NMR spectrum for **S1ak-c**

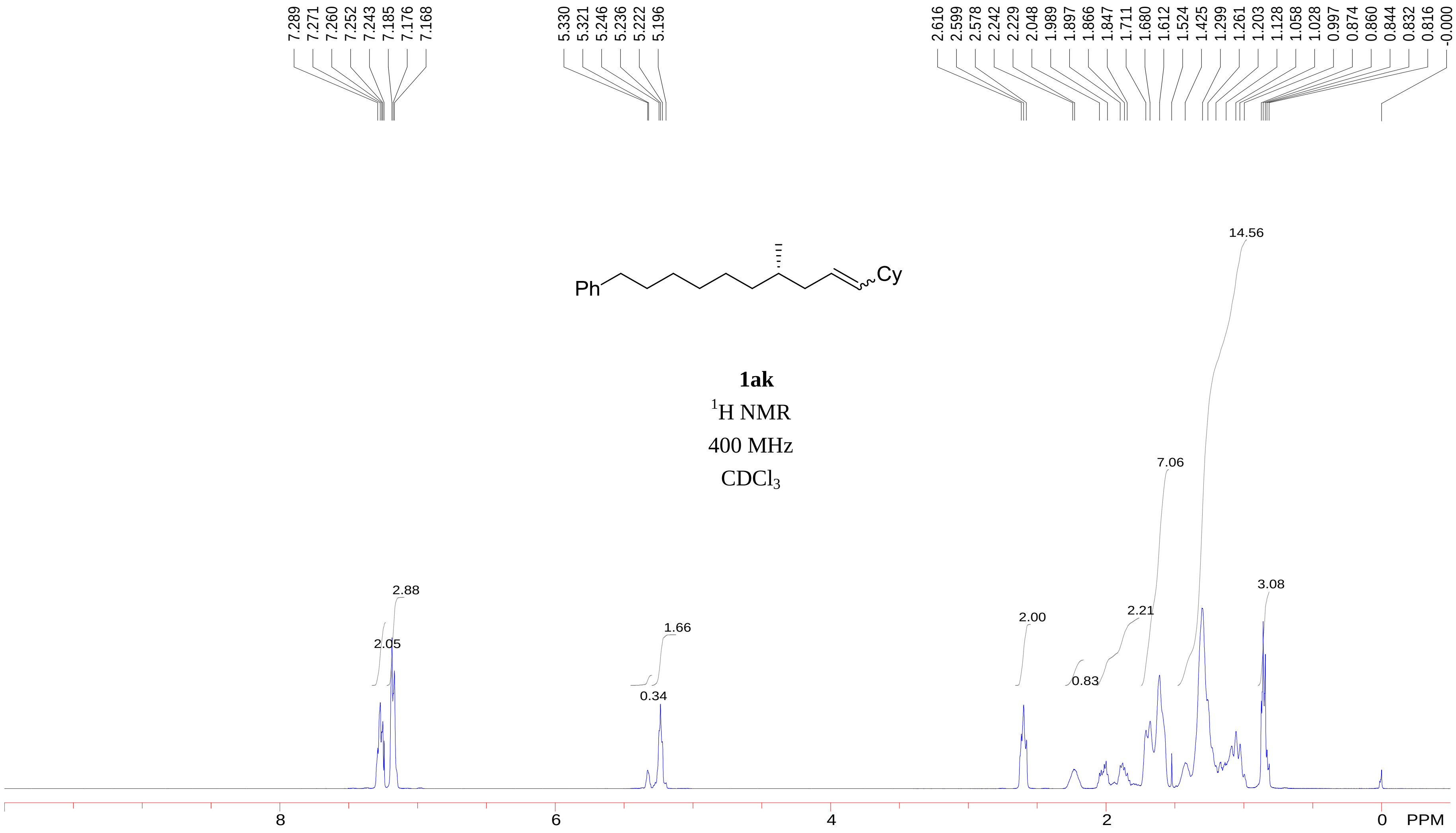

Supplementary Figure 123. ^{1}H NMR spectrum for **1ak**

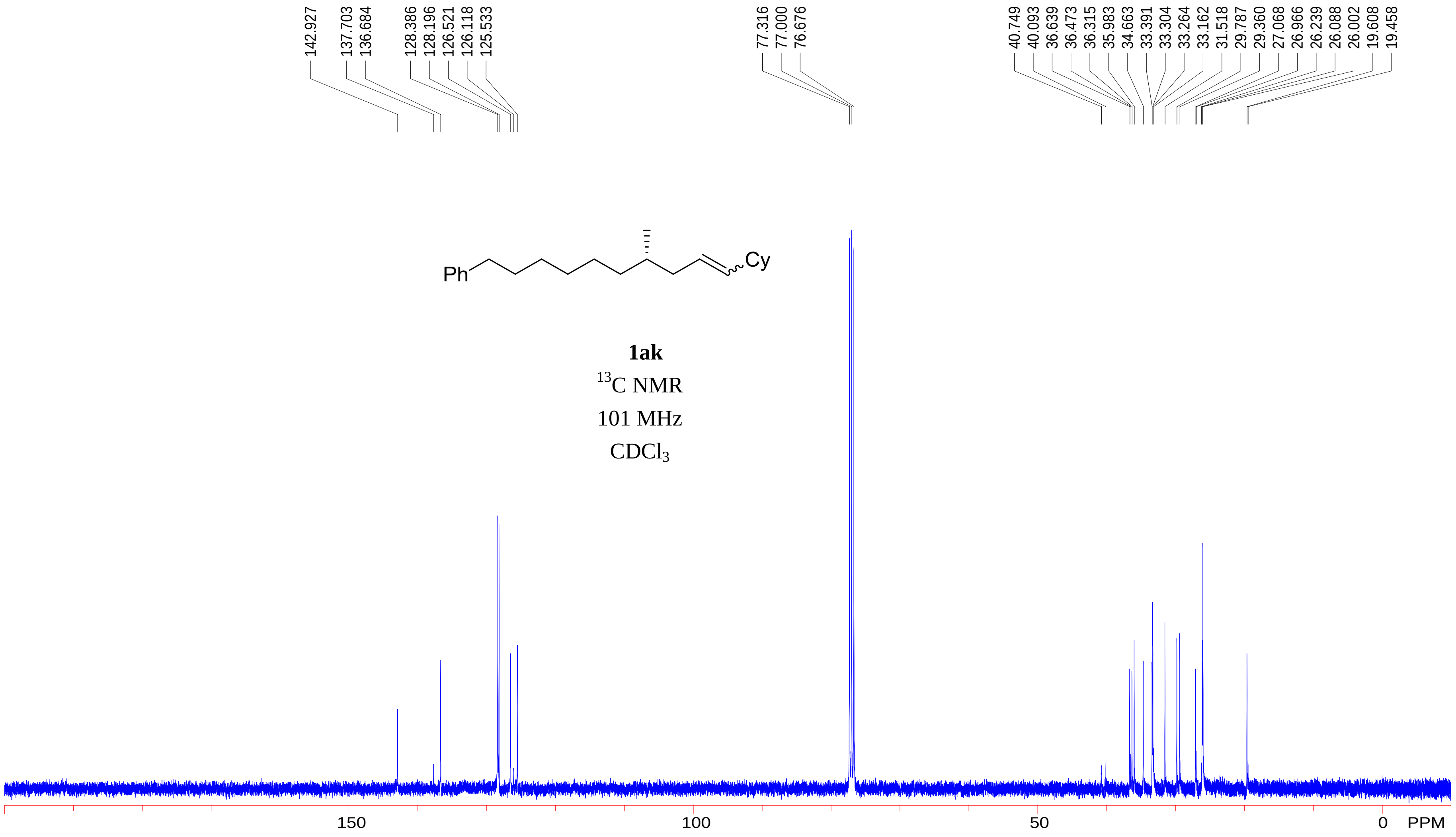

Supplementary Figure 124. ^{13}C NMR spectrum for **1ak**

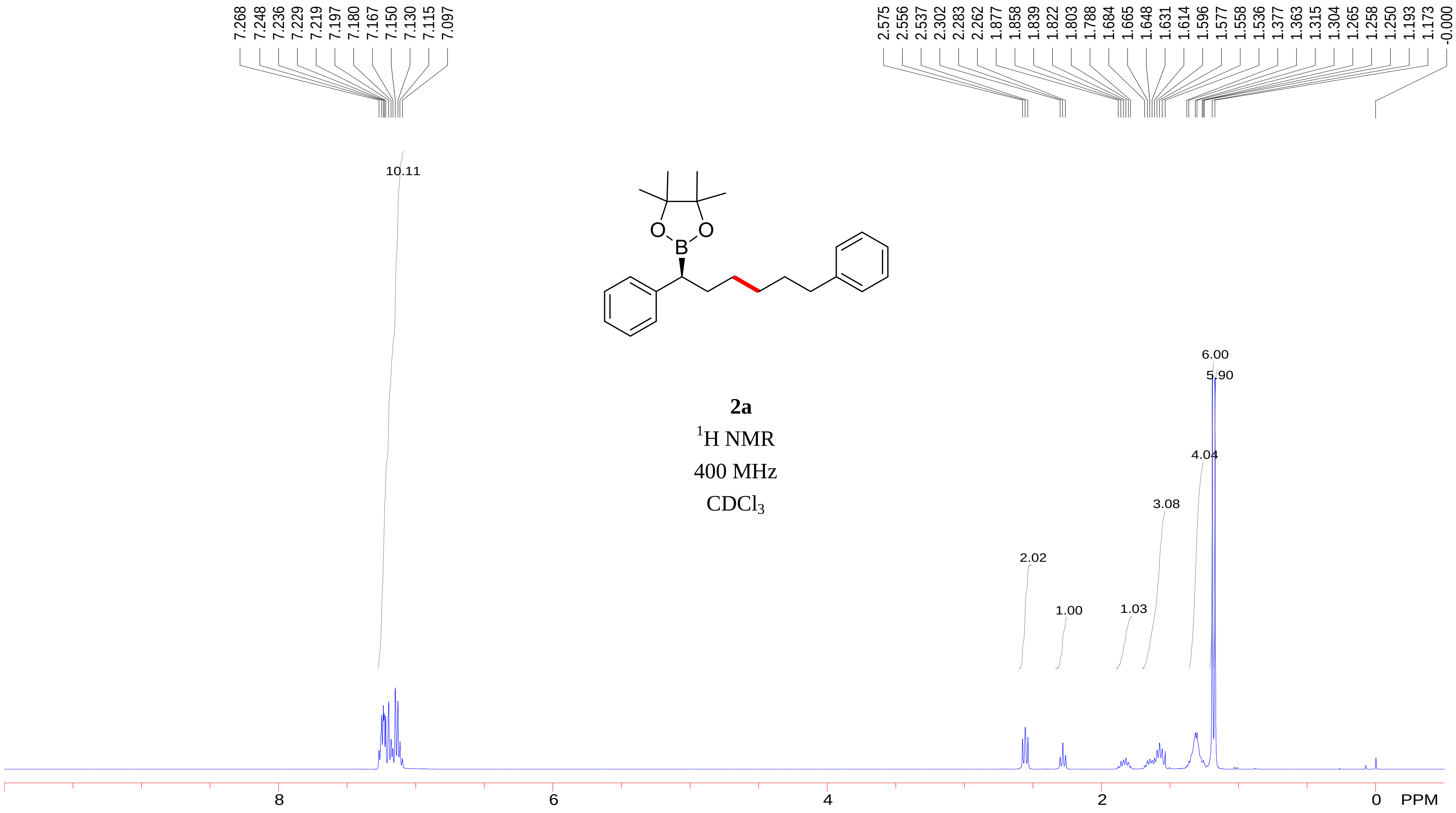

Supplementary Figure 125. ^{1}H NMR spectrum for **2a**

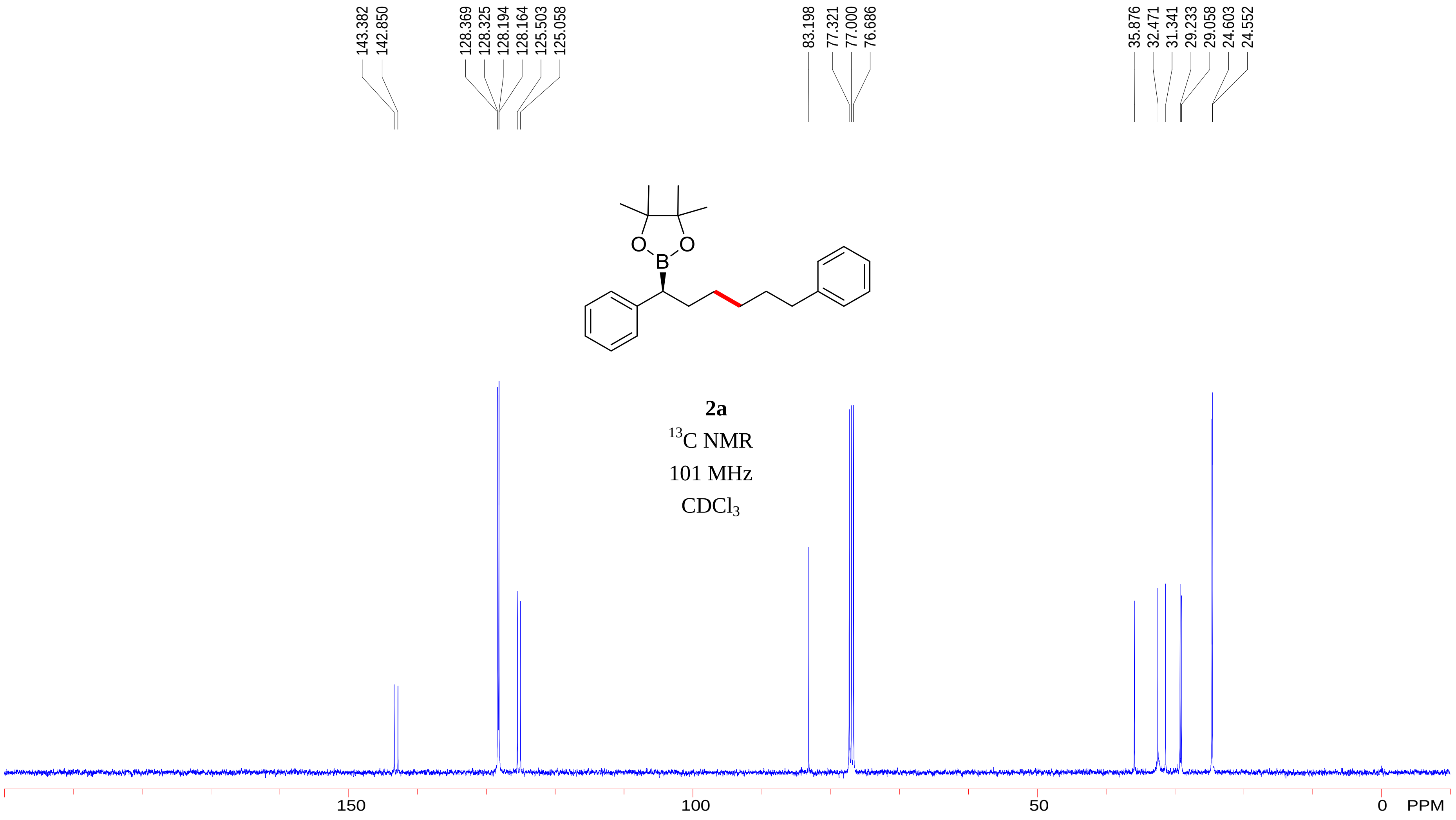

Supplementary Figure 126. ^{13}C NMR spectrum for **2a**

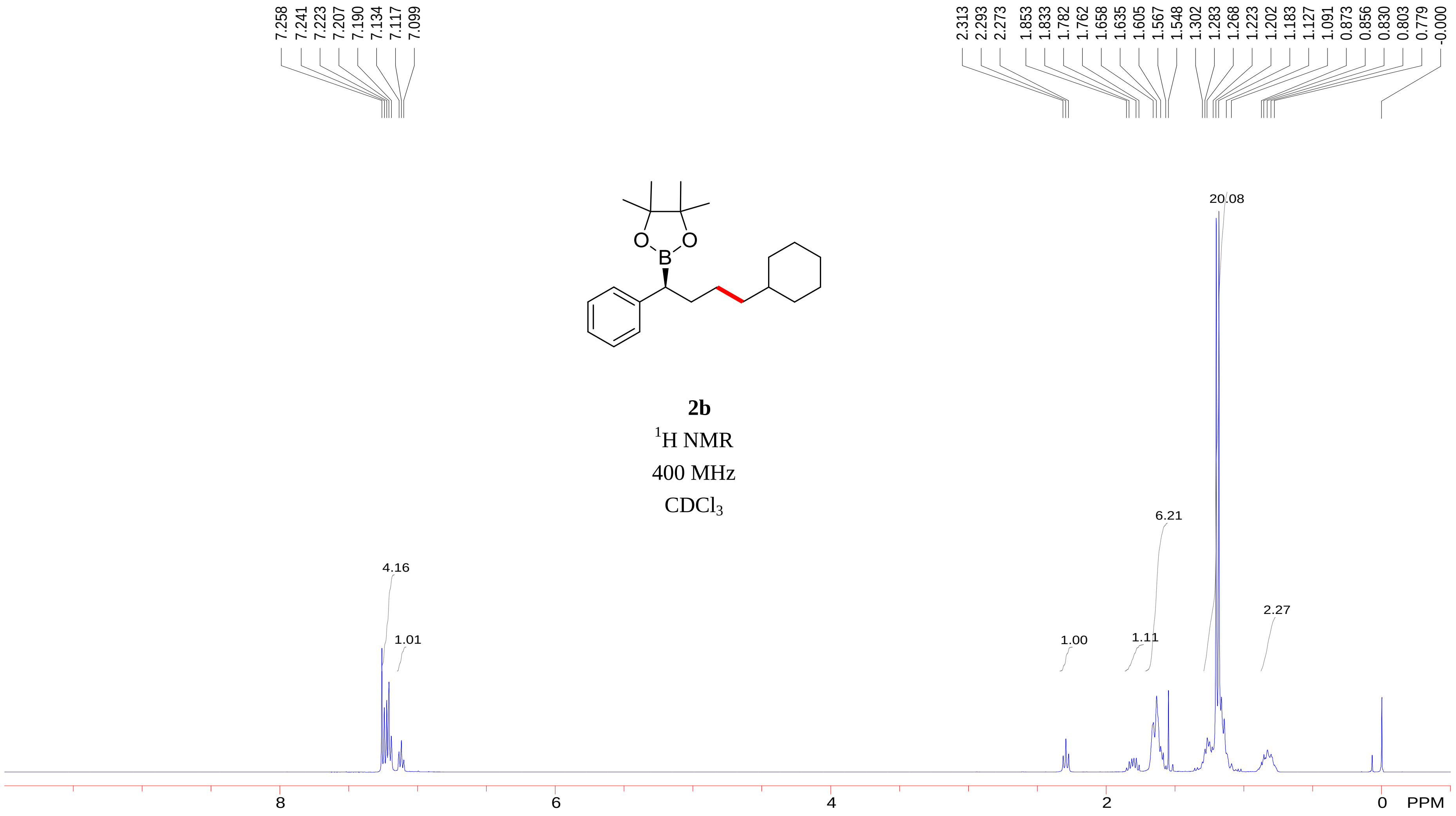

Supplementary Figure 127. ^{1}H NMR spectrum for **2b**

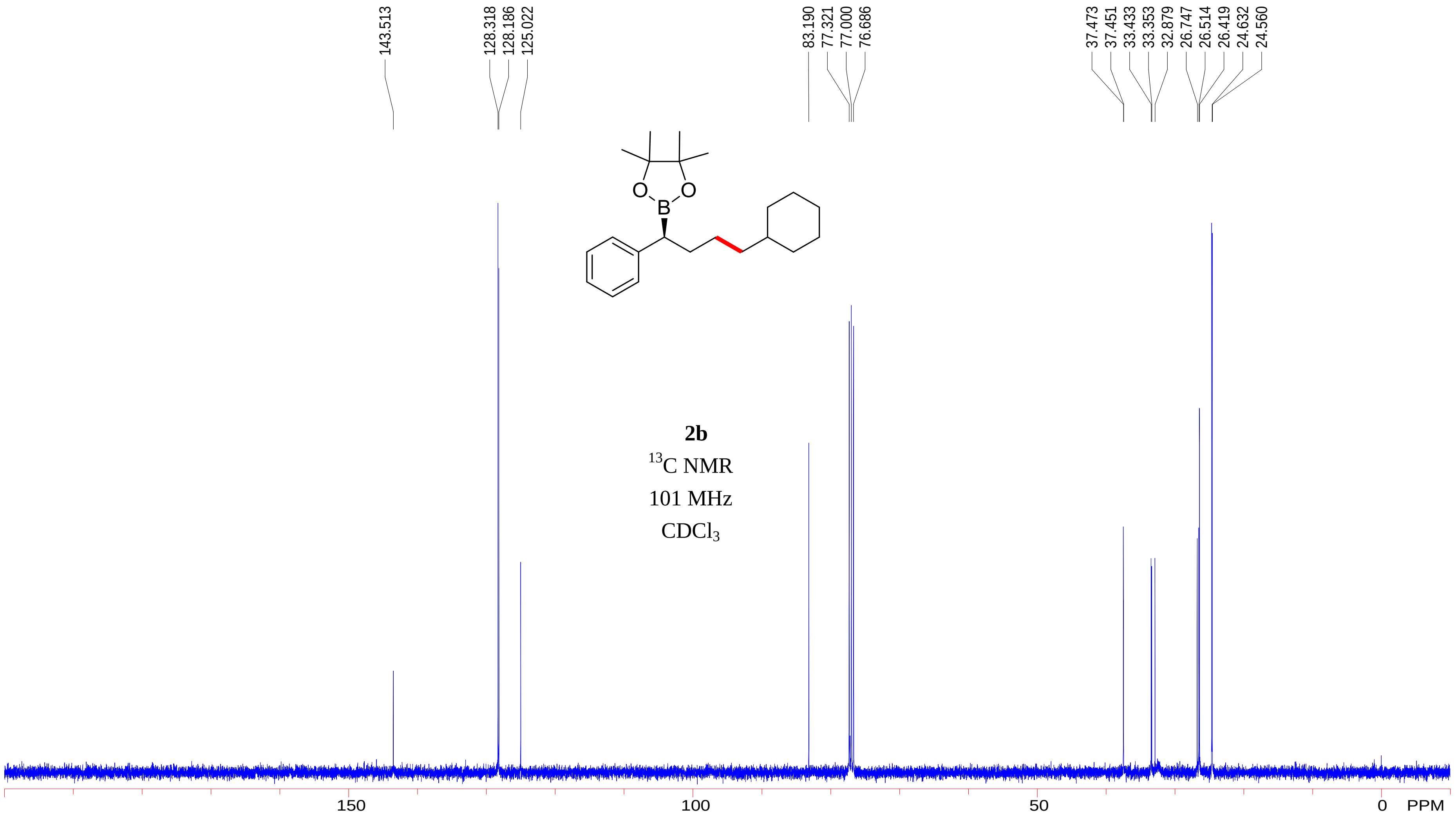

Supplementary Figure 128. ^{13}C NMR spectrum for **2b**

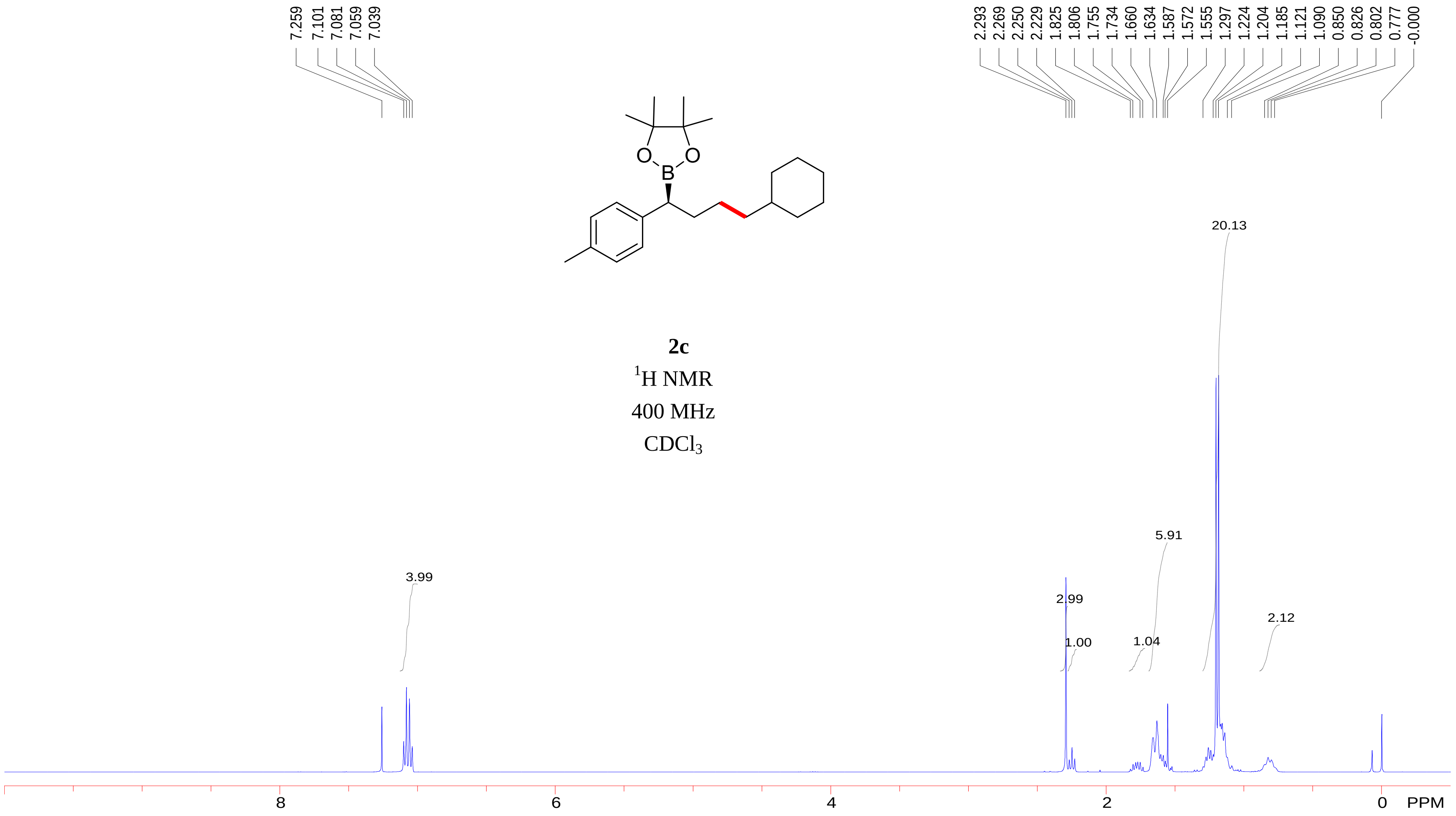

Supplementary Figure 129. ^{1}H NMR spectrum for **2c**

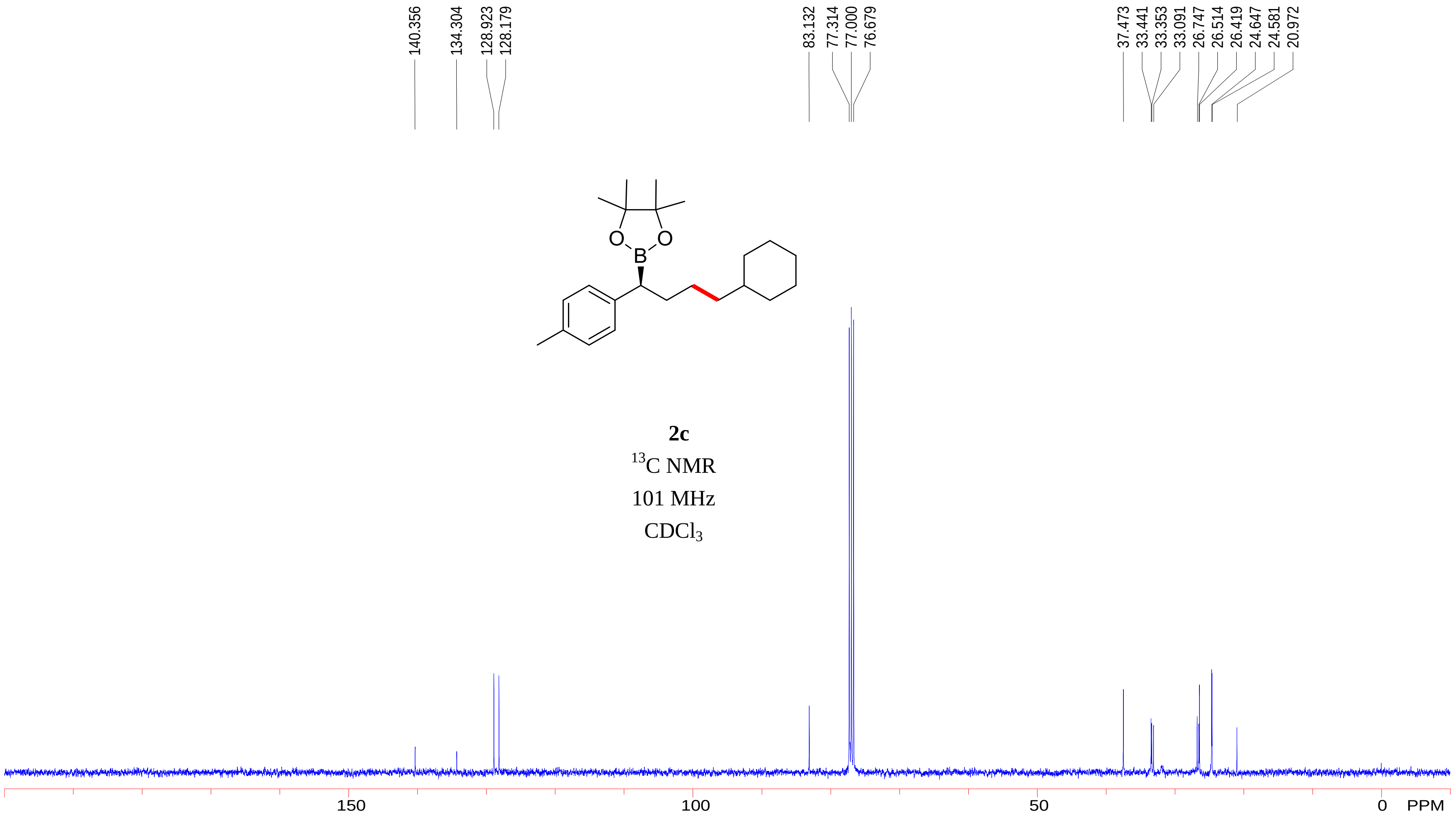

Supplementary Figure 130. ^{13}C NMR spectrum for **2c**

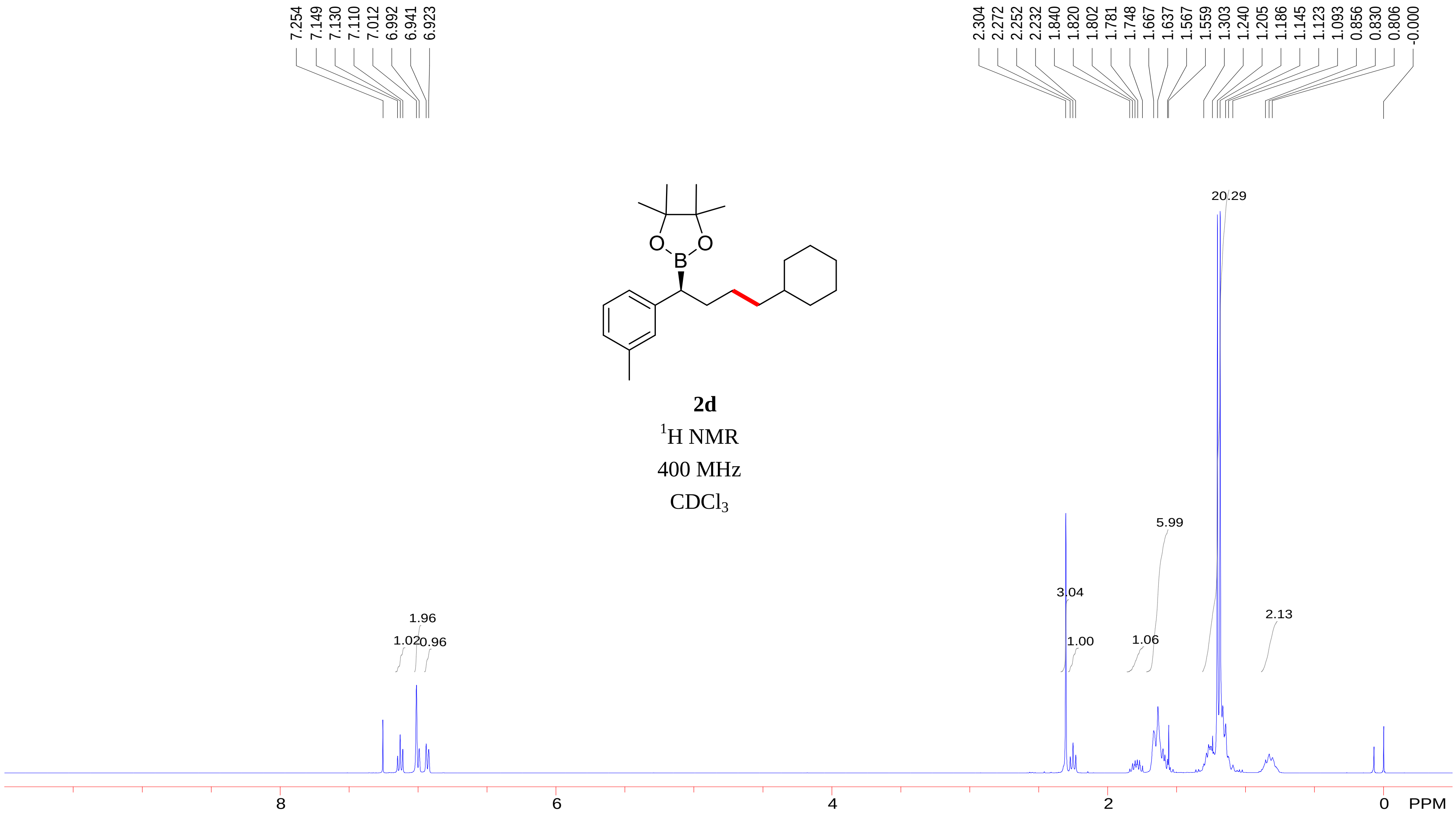

Supplementary Figure 131. ^{1}H NMR spectrum for **2d**

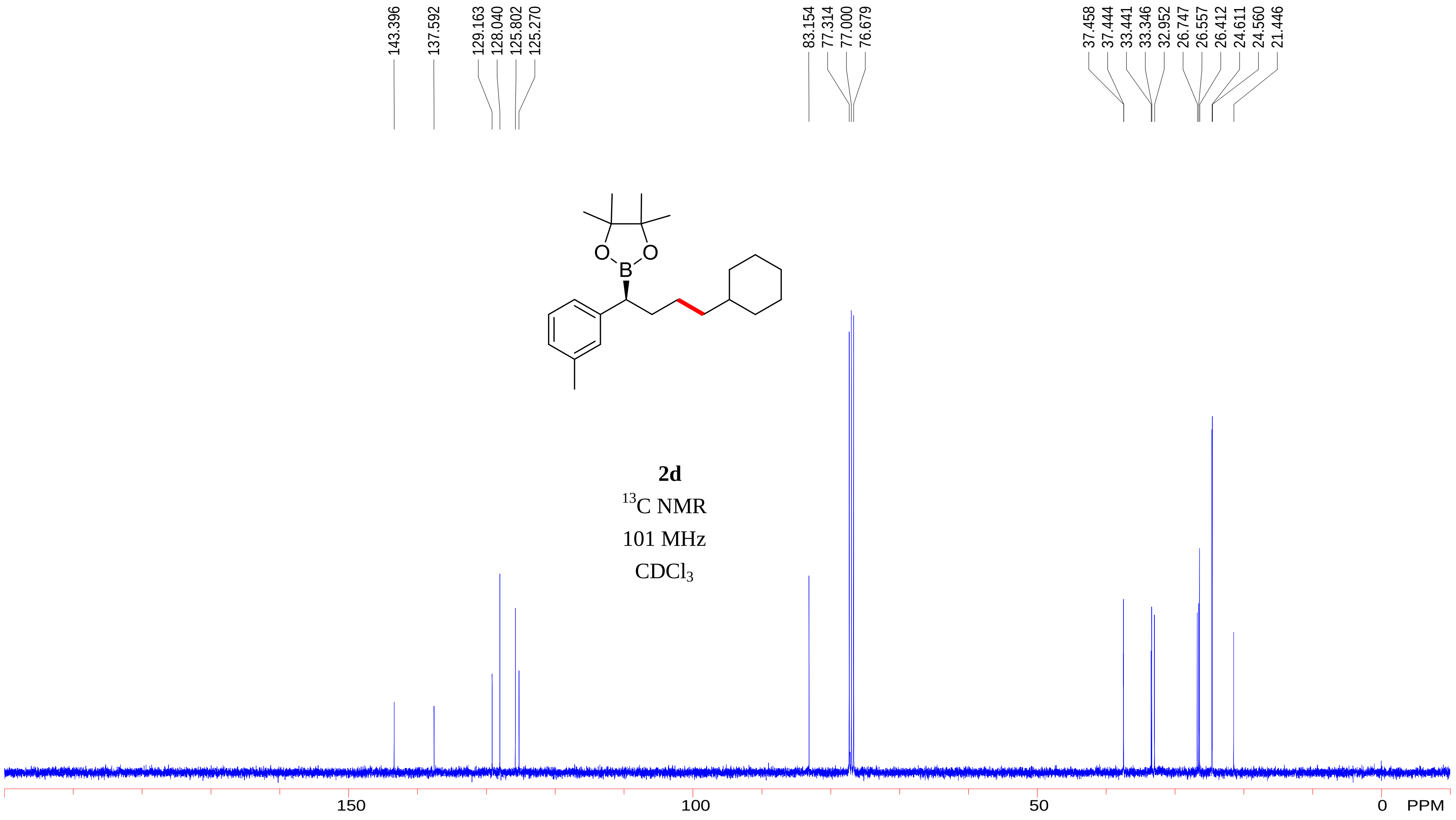

Supplementary Figure 132. ^{13}C NMR spectrum for **2d**

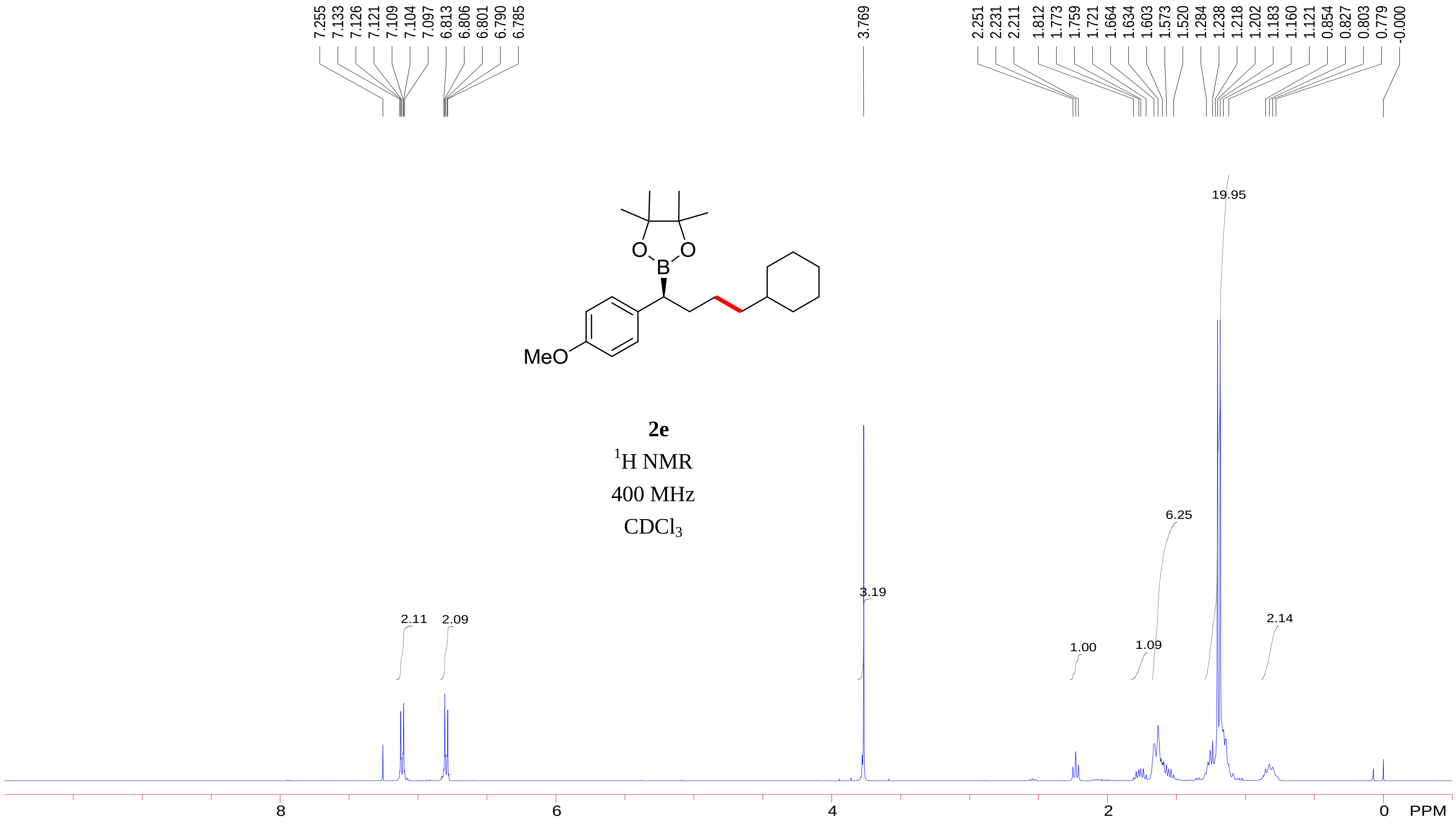

Supplementary Figure 133. ^{1}H NMR spectrum for **2e**

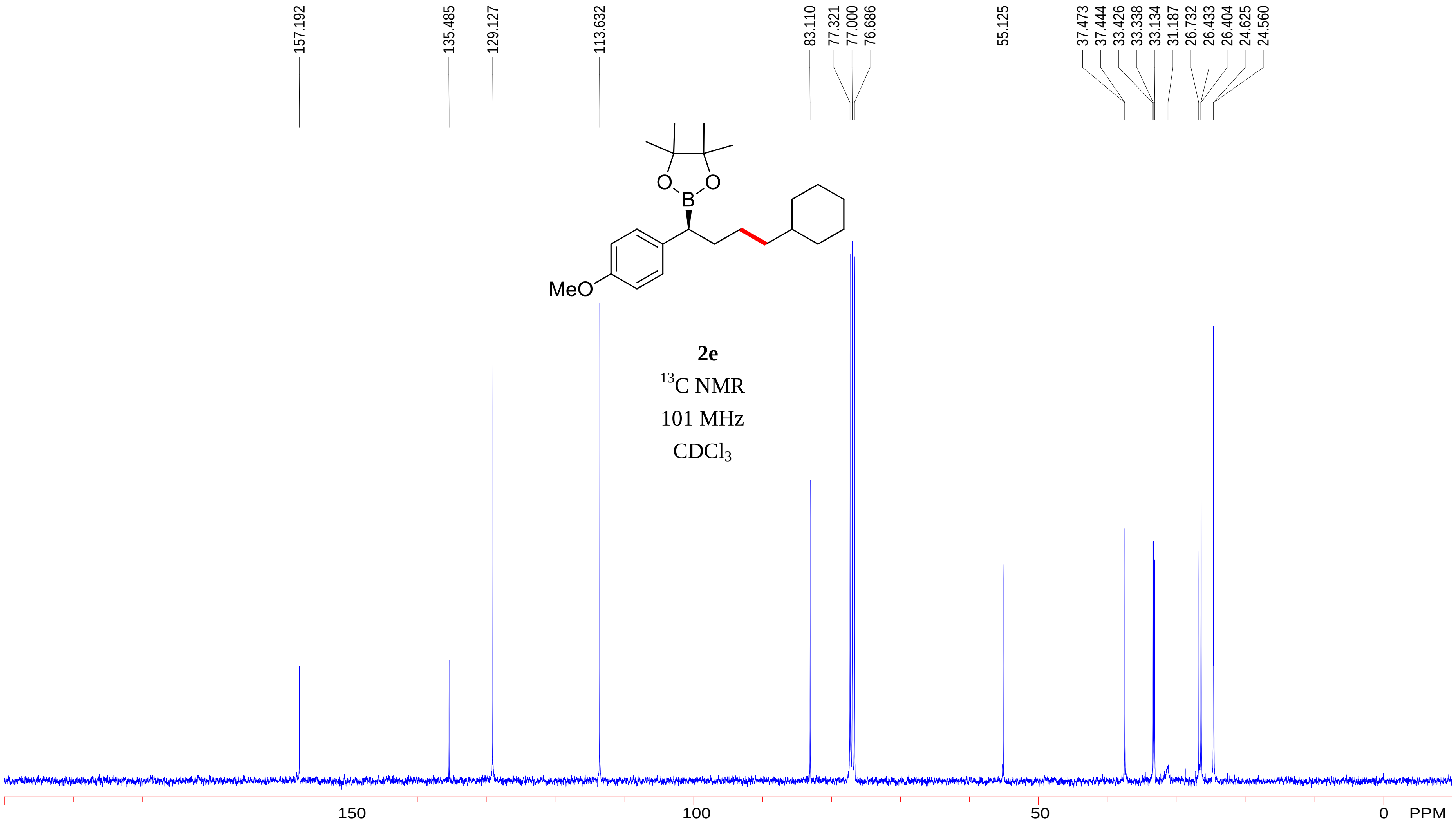

Supplementary Figure 134. ^{13}C NMR spectrum for **2e**

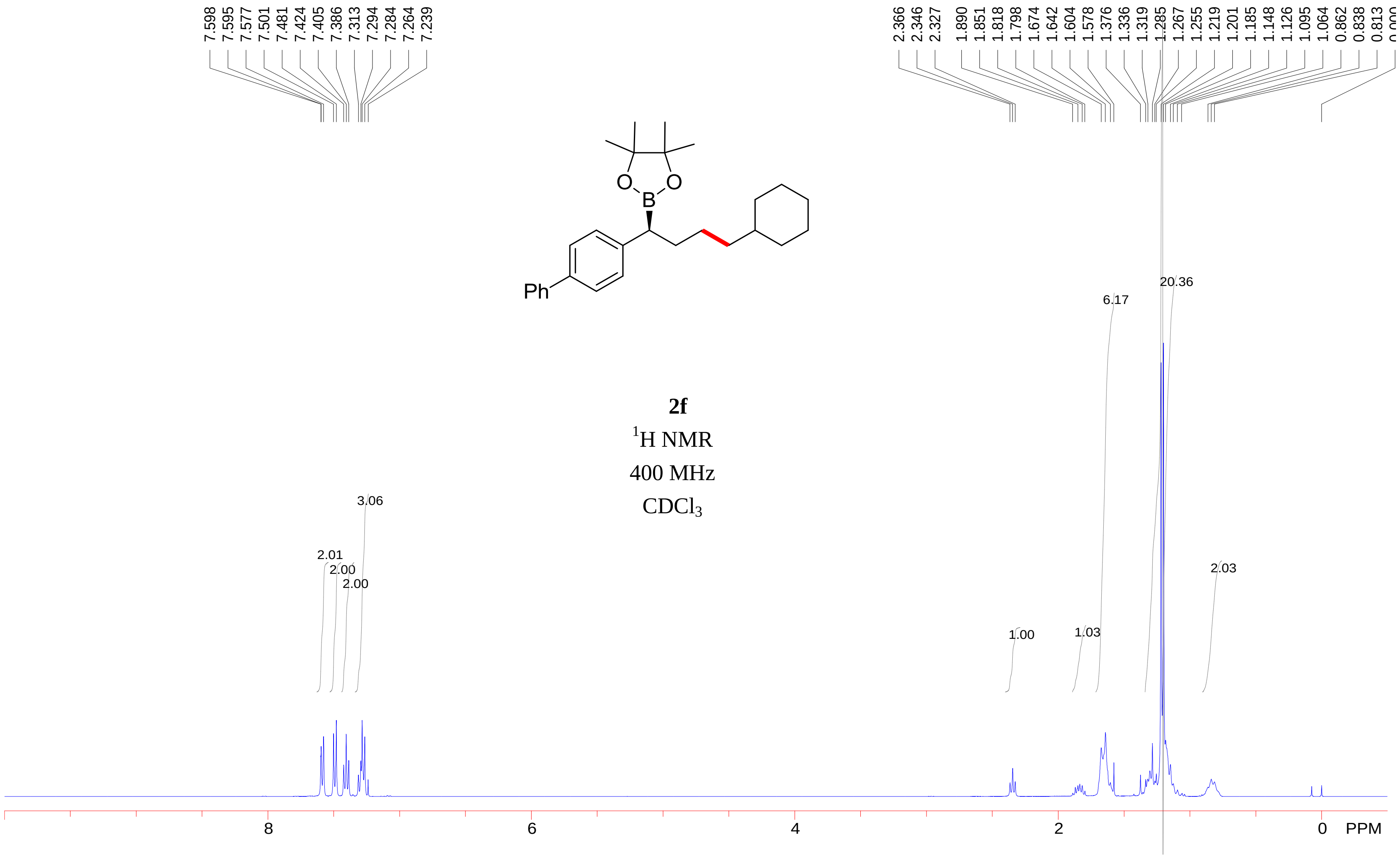

Supplementary Figure 135. ^{1}H NMR spectrum for **2f**

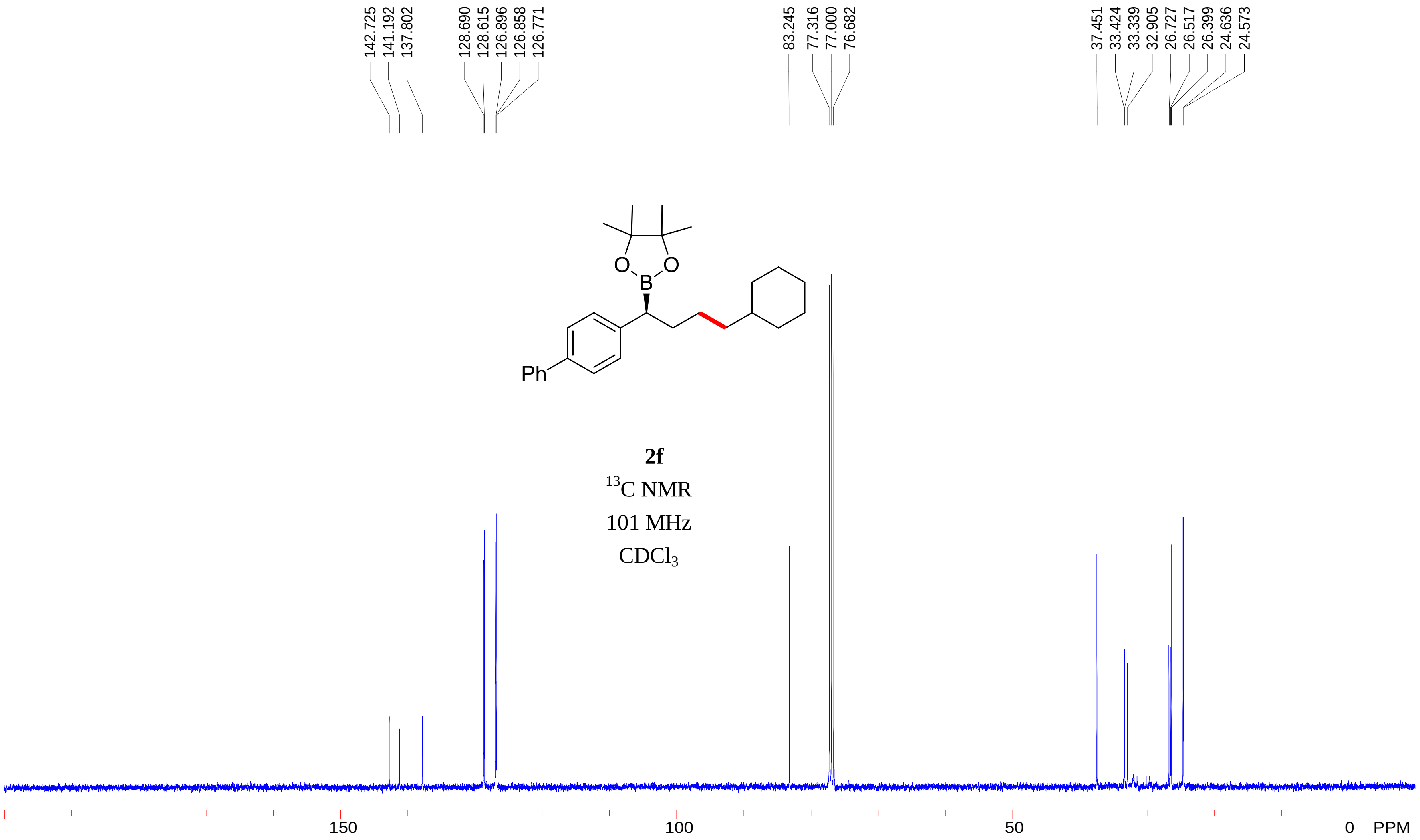

Supplementary Figure 136. ^{13}C NMR spectrum for **2f**

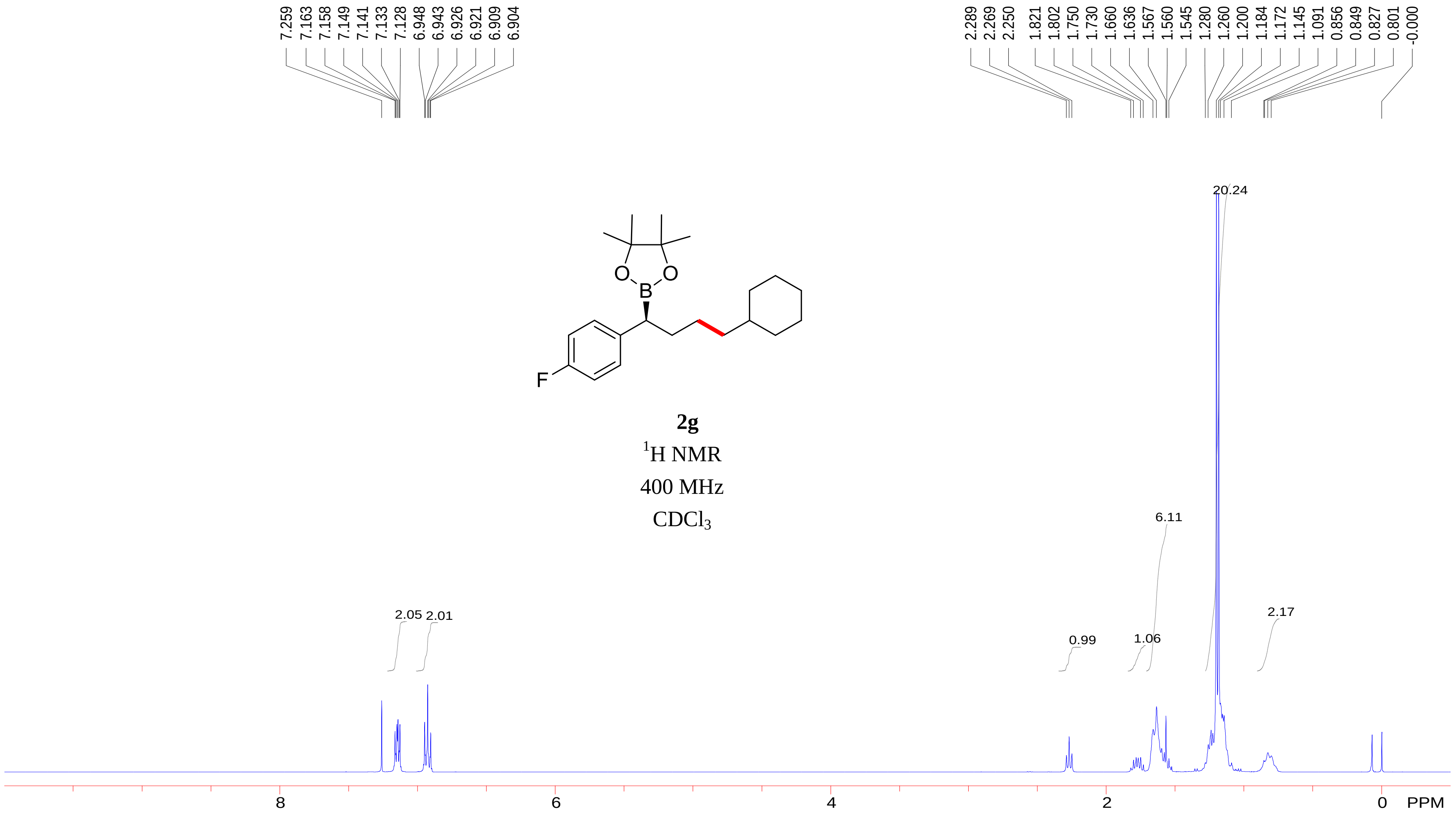

Supplementary Figure 137. ^{1}H NMR spectrum for **2g**

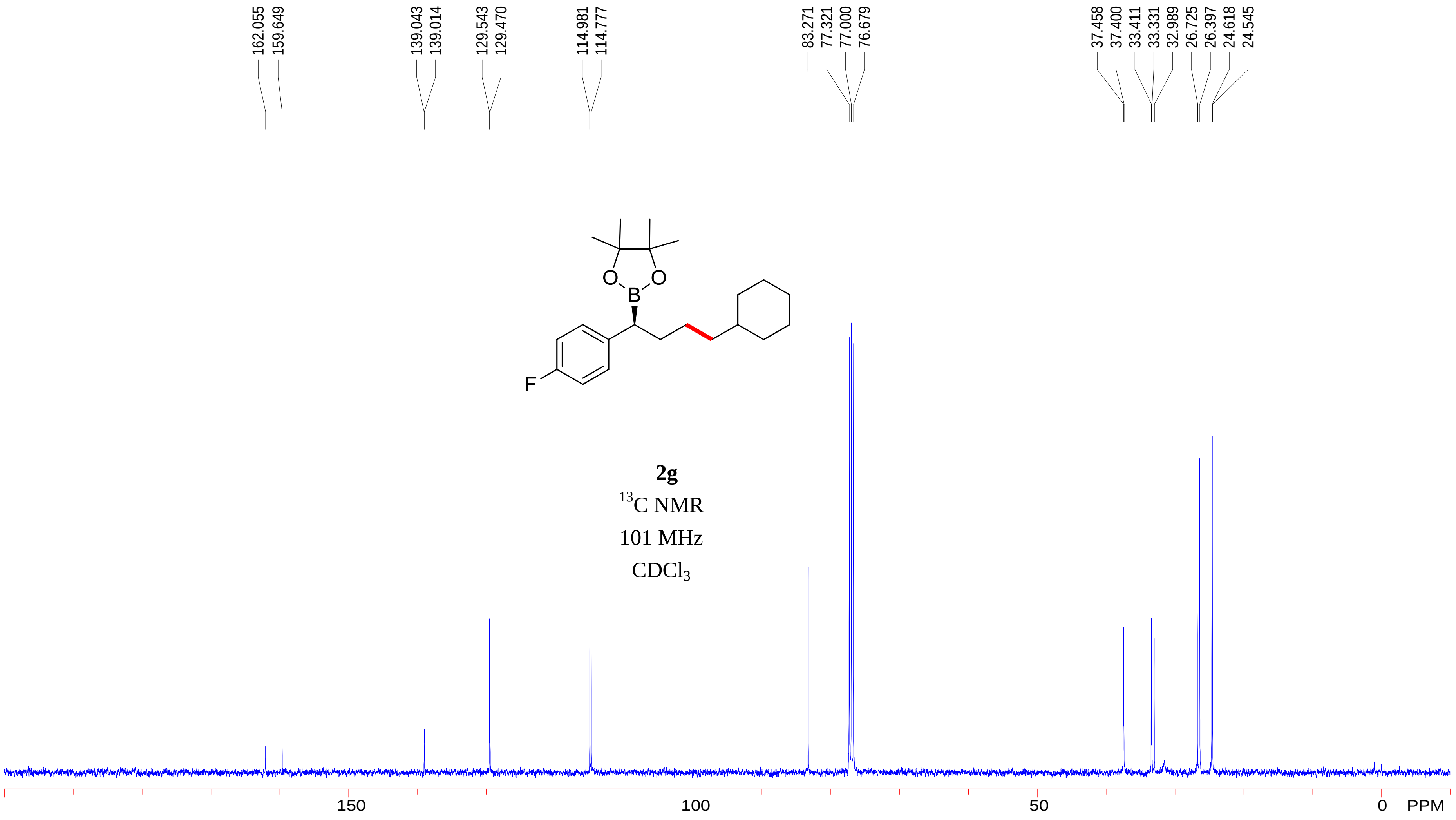

Supplementary Figure 138. ^{13}C NMR spectrum for **2g**

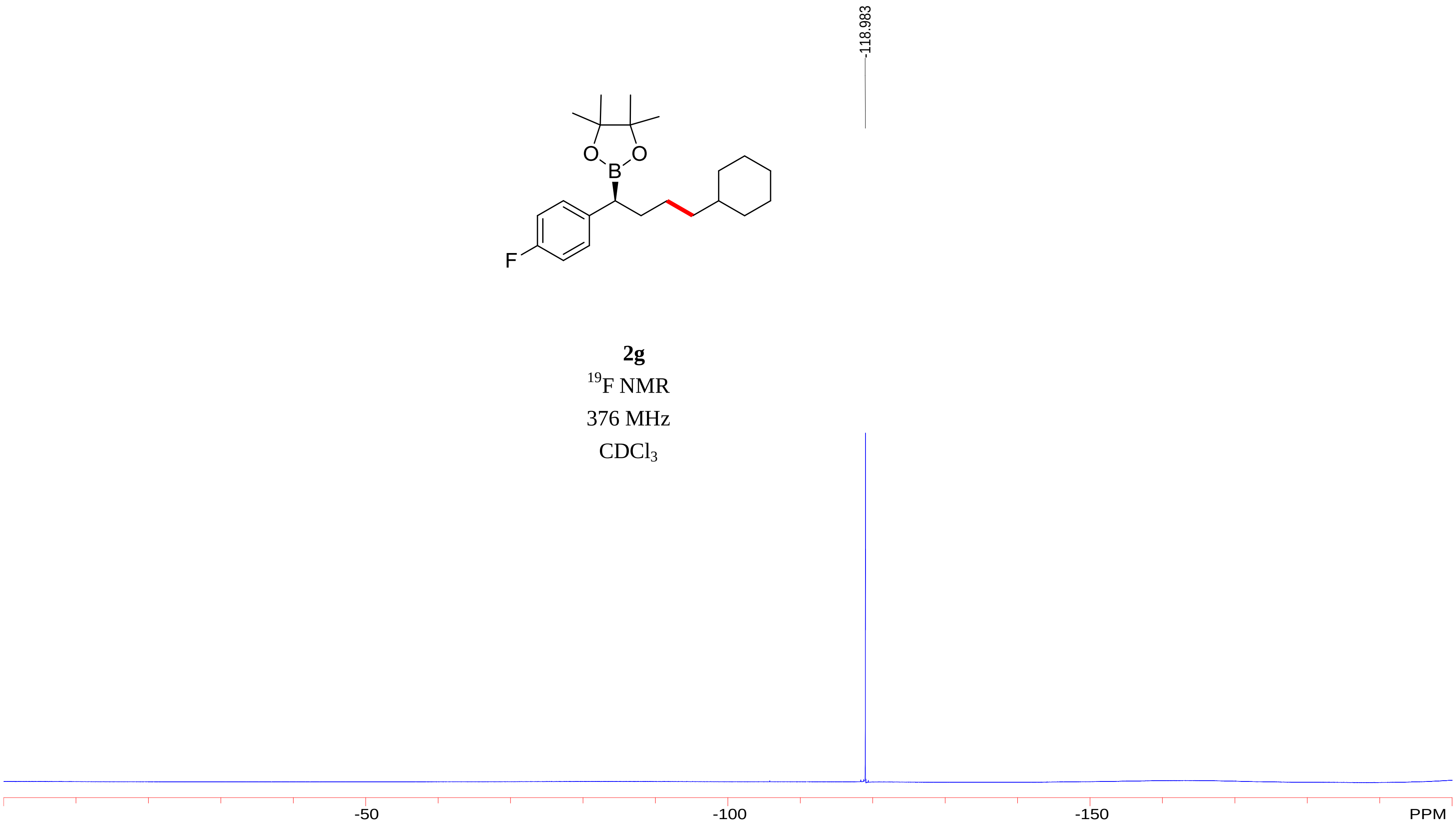

Supplementary Figure 139. ^{19}FNMR spectrum for **2g**

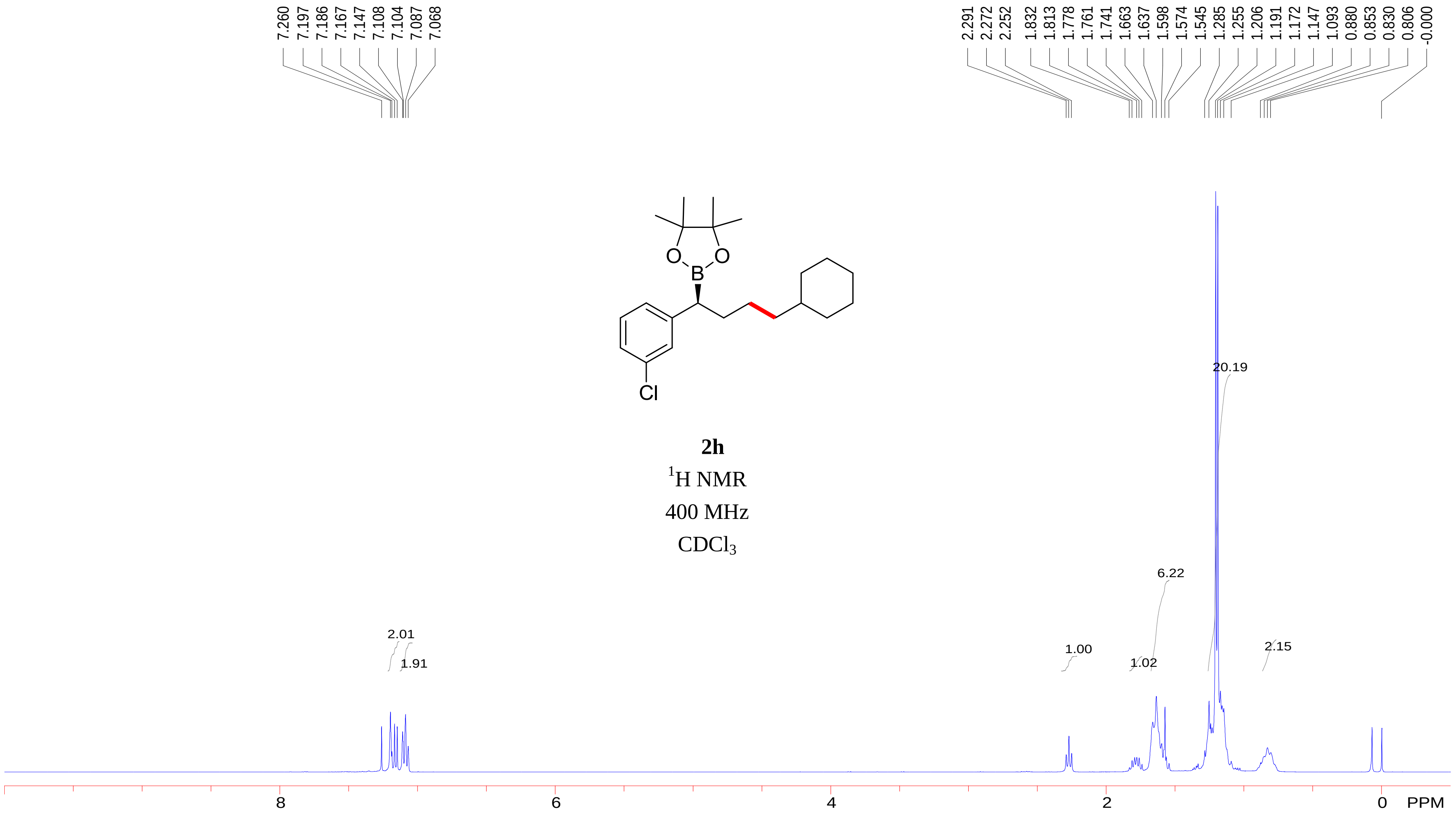

Supplementary Figure 140. ^{1}H NMR spectrum for **2h**

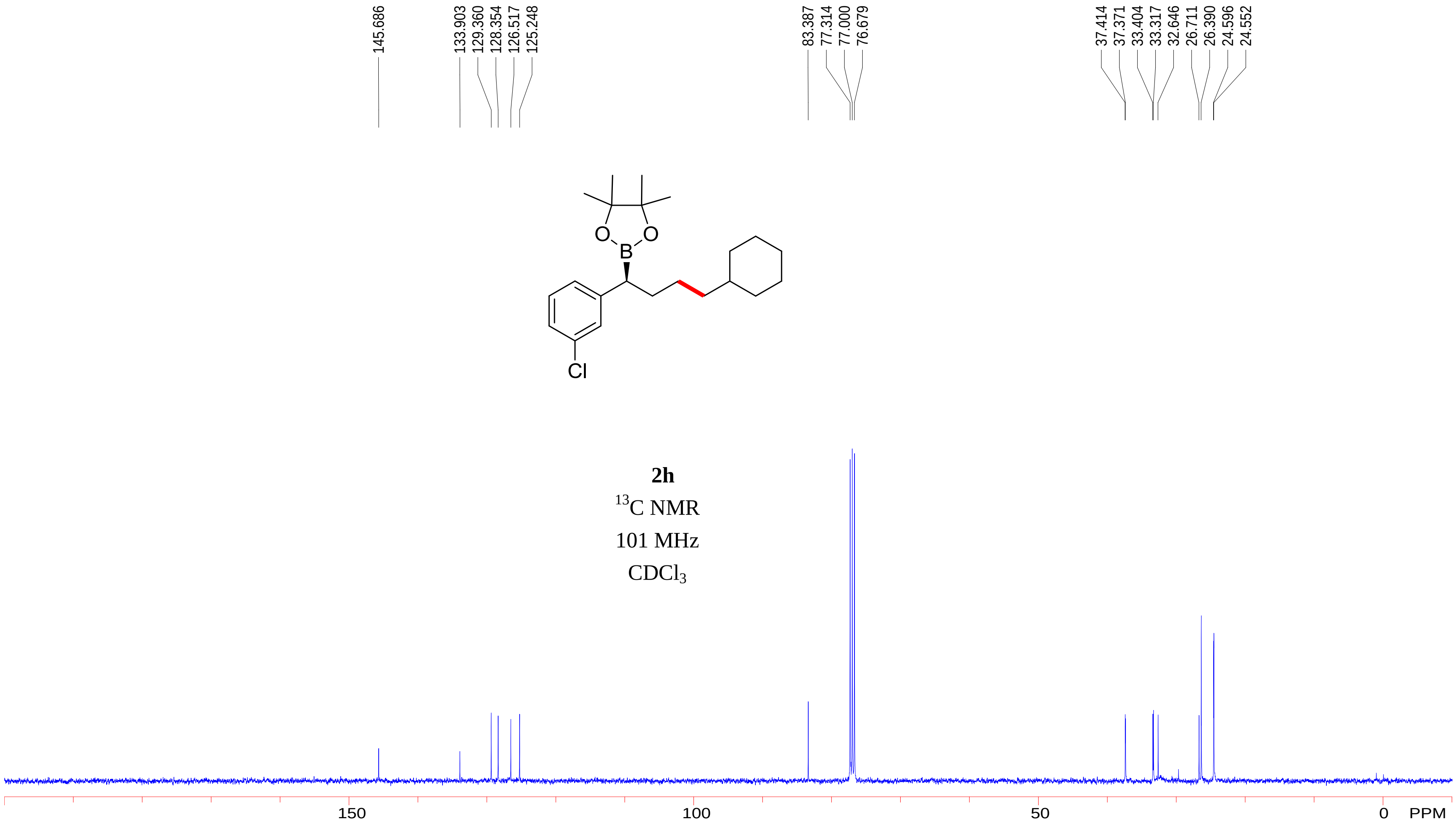

Supplementary Figure 141. ^{13}C NMR spectrum for **2h**

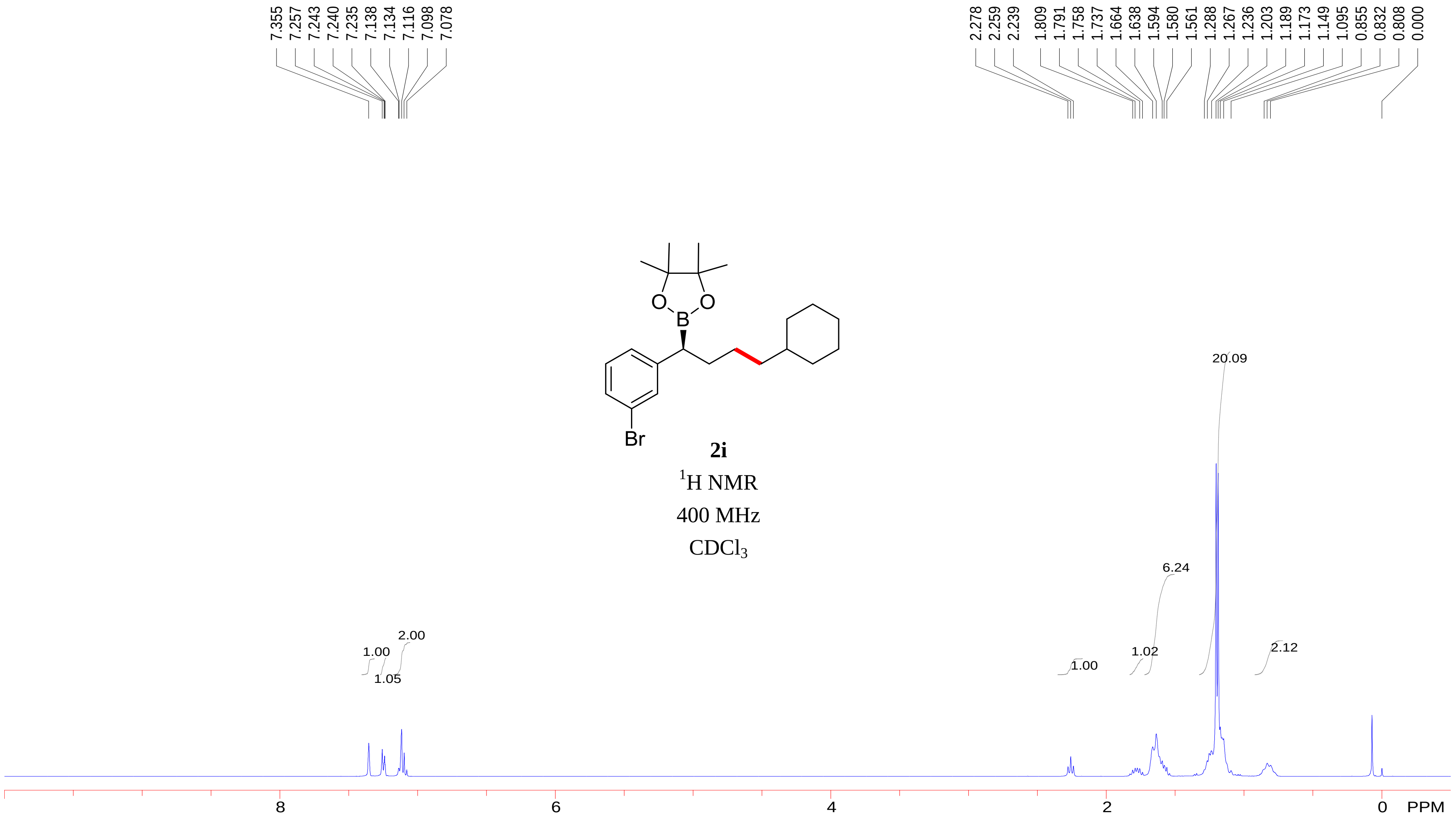

Supplementary Figure 142. ^{1}H NMR spectrum for **2i**

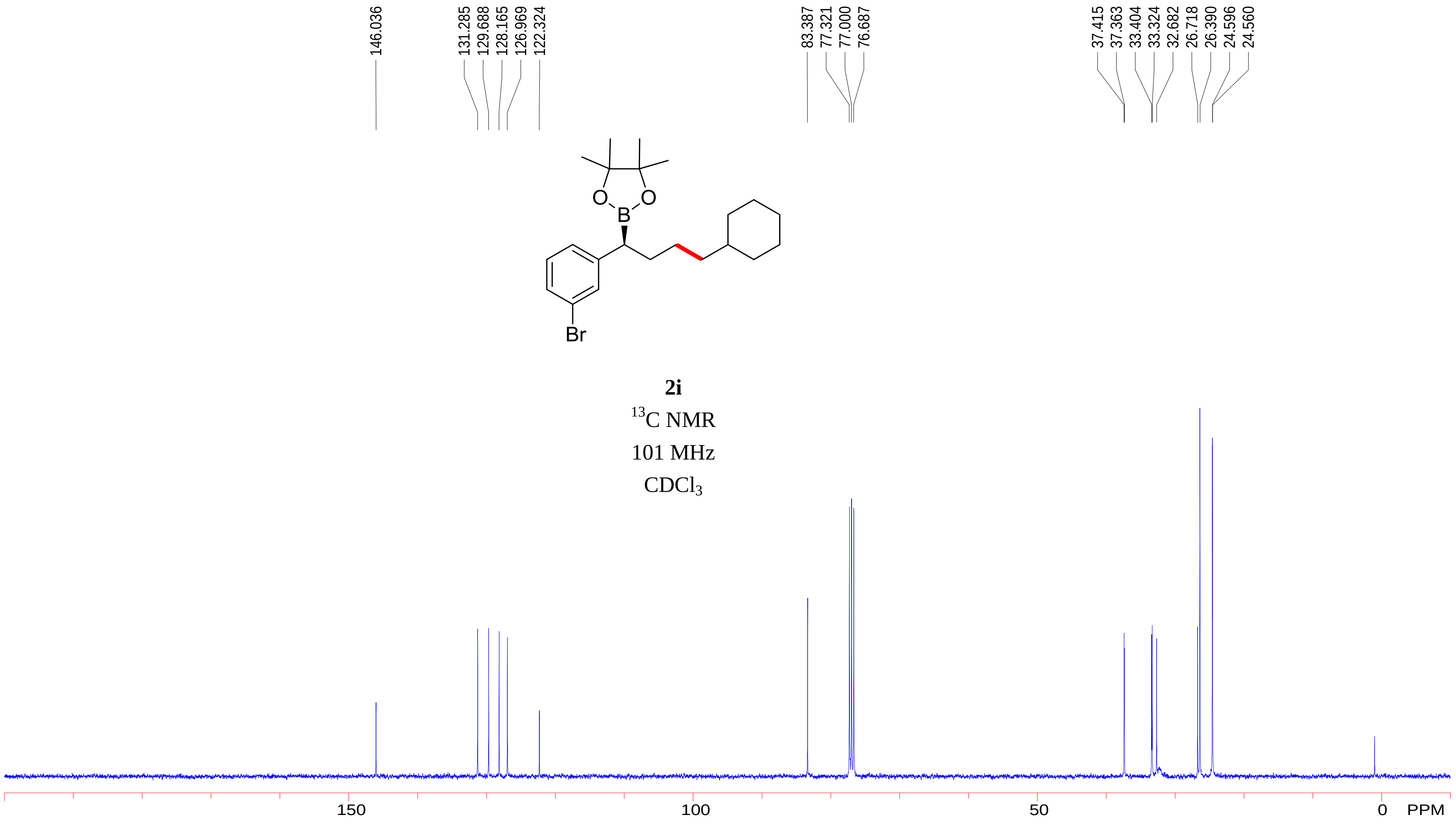

Supplementary Figure 143. ^{13}C NMR spectrum for **2i**

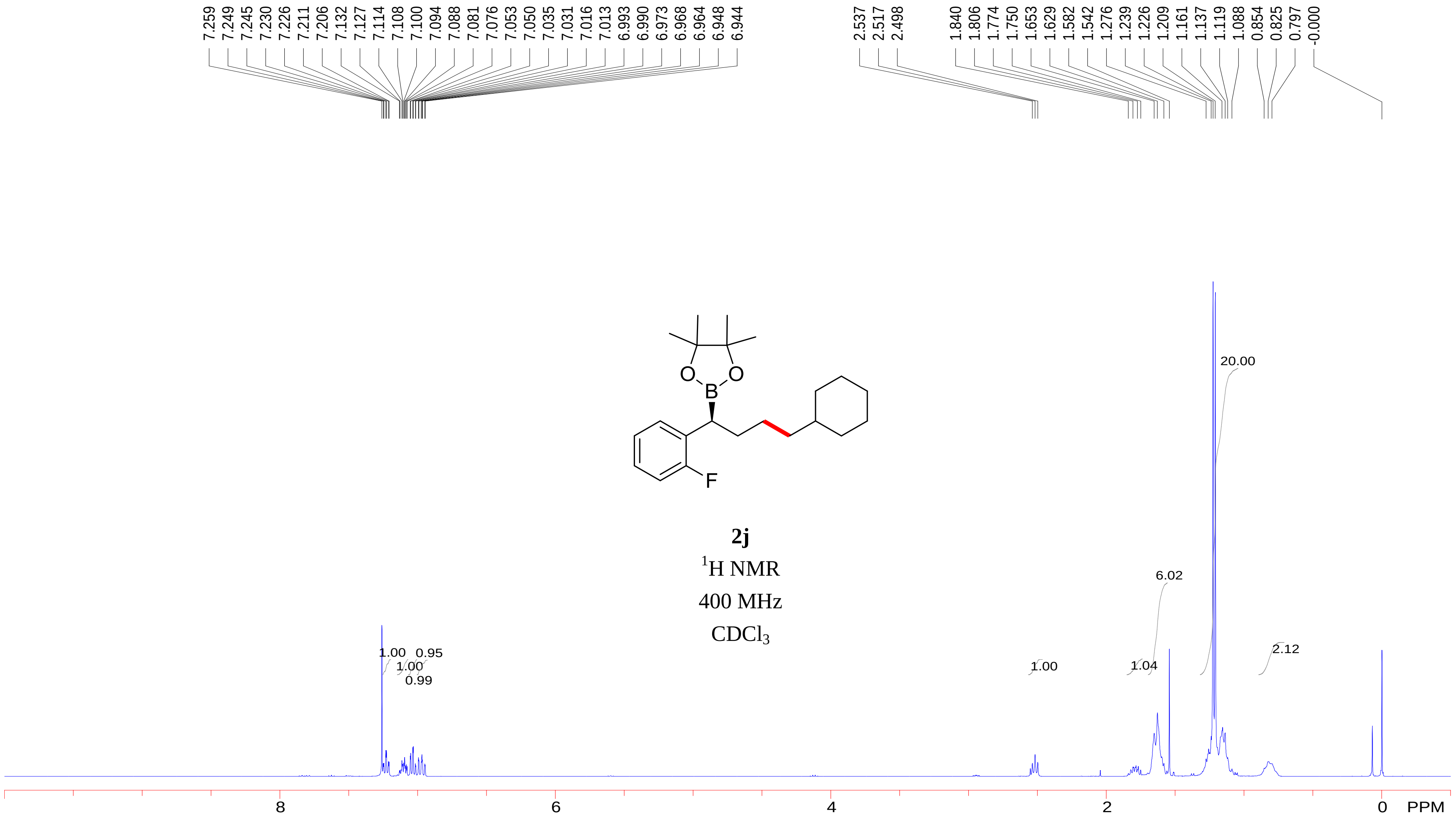

Supplementary Figure 144. ^{1}H NMR spectrum for **2j**

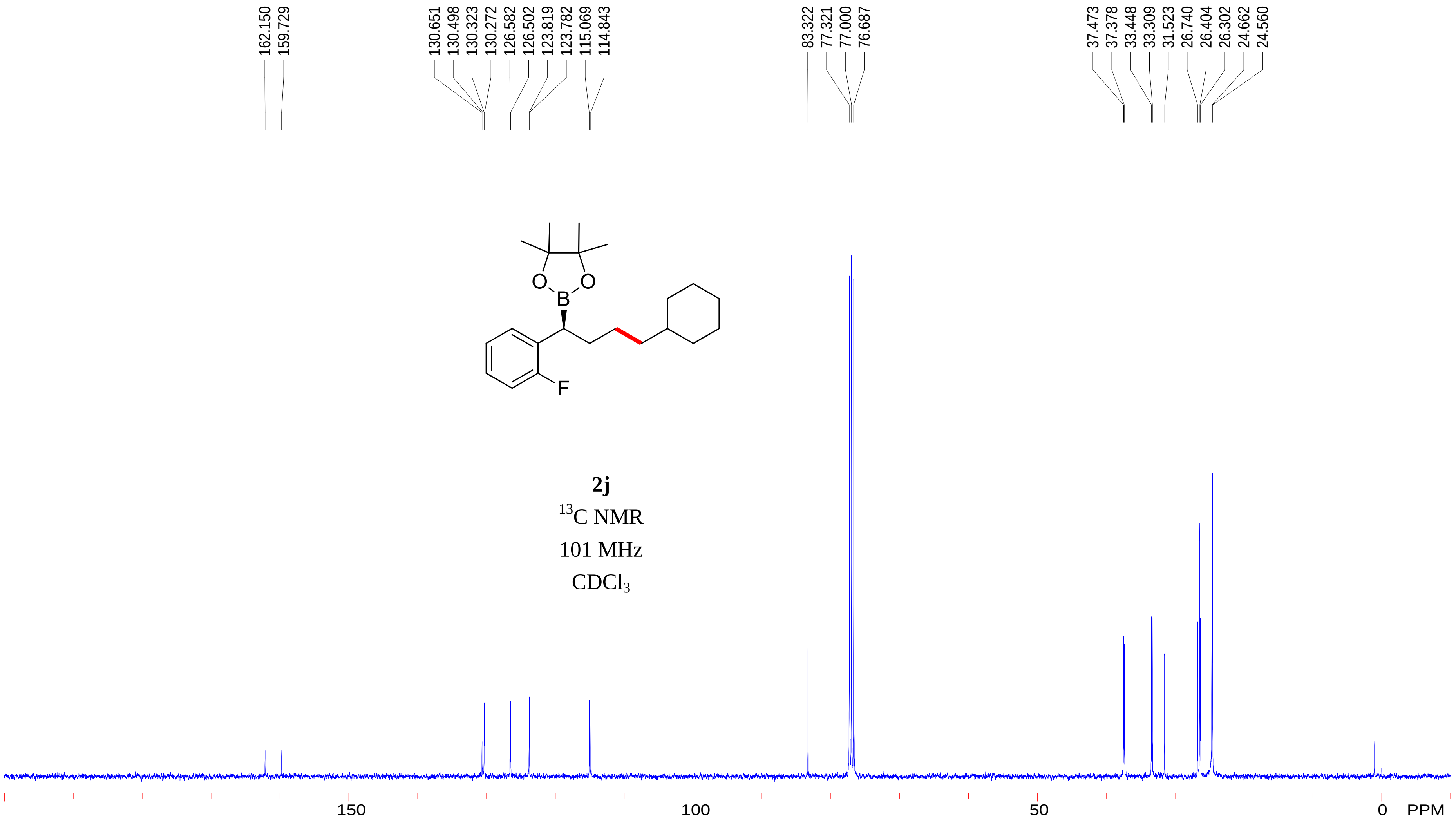

Supplementary Figure 145. ^{13}C NMR spectrum for **2j**

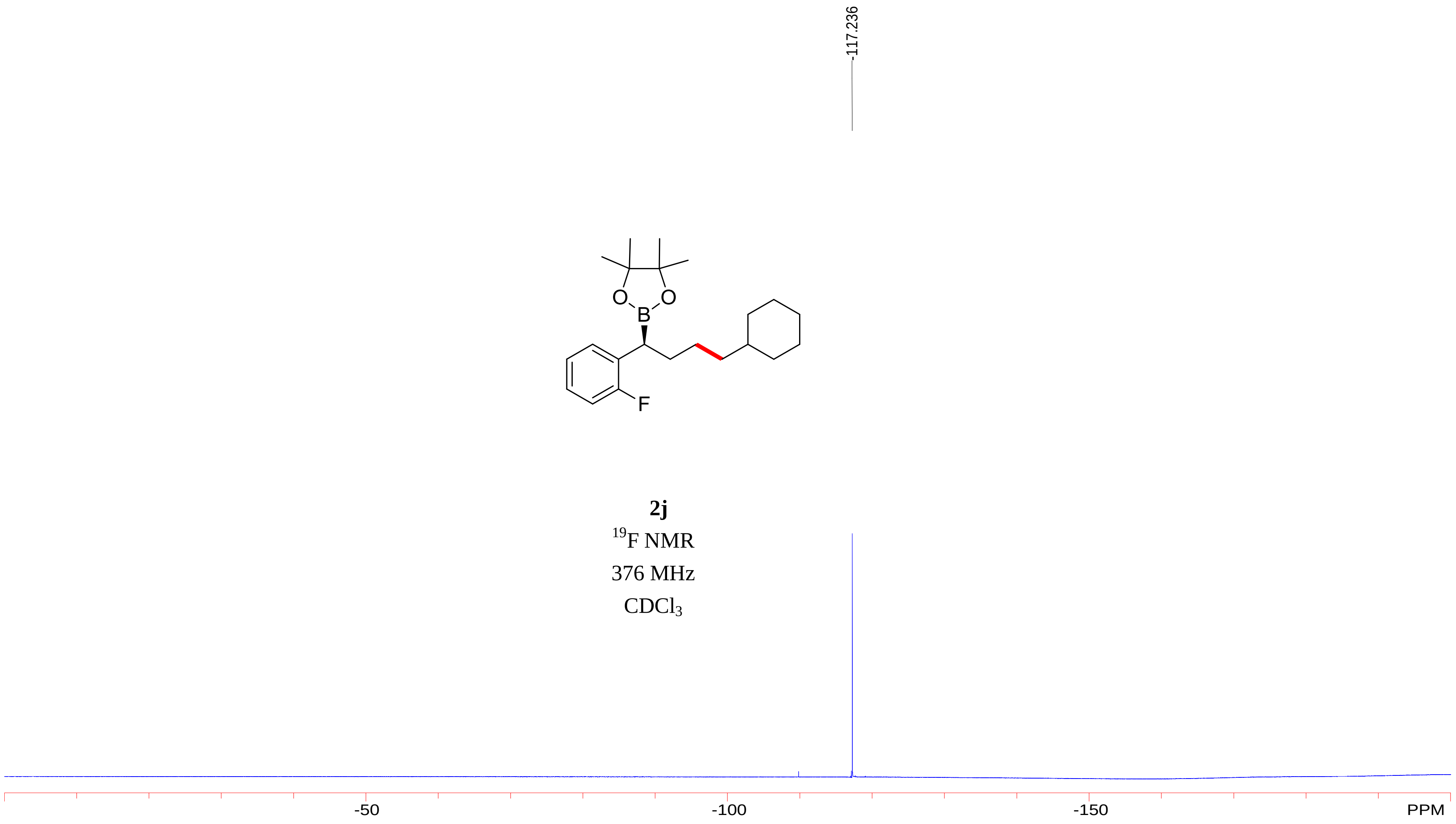

Supplementary Figure 146. ^{19}F NMR spectrum for **2j**

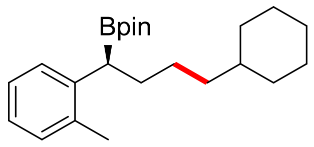

2k
^{1}H NMR
400 MHz
$CDCl_3$

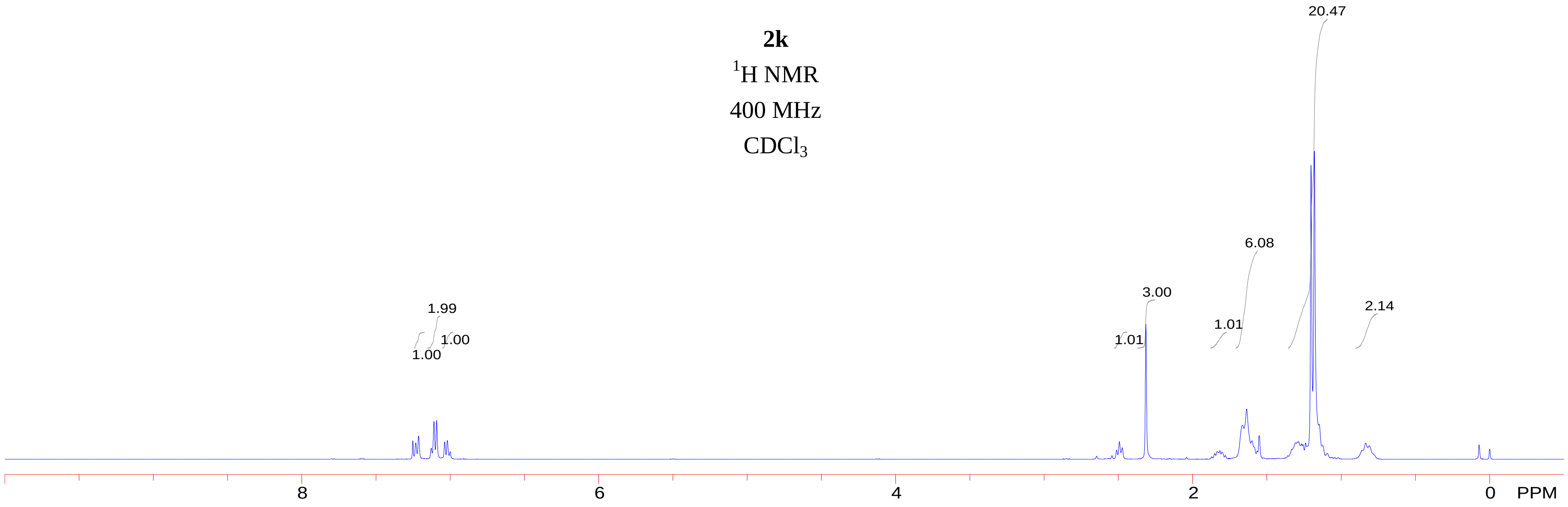

Supplementary Figure 147. ^{1}H NMR spectrum for **2k**

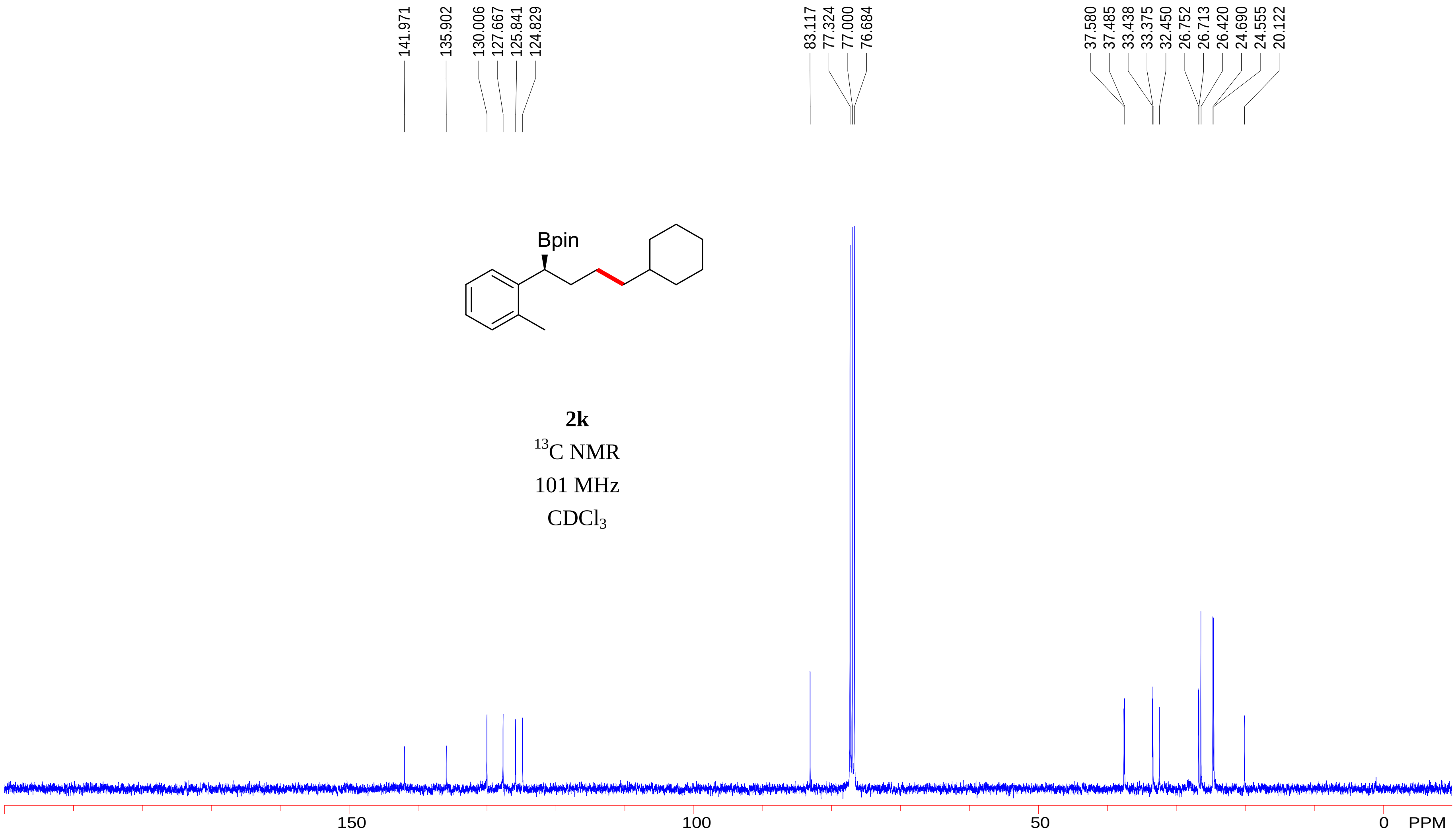

Supplementary Figure 148. ^{13}C NMR spectrum for **2k**

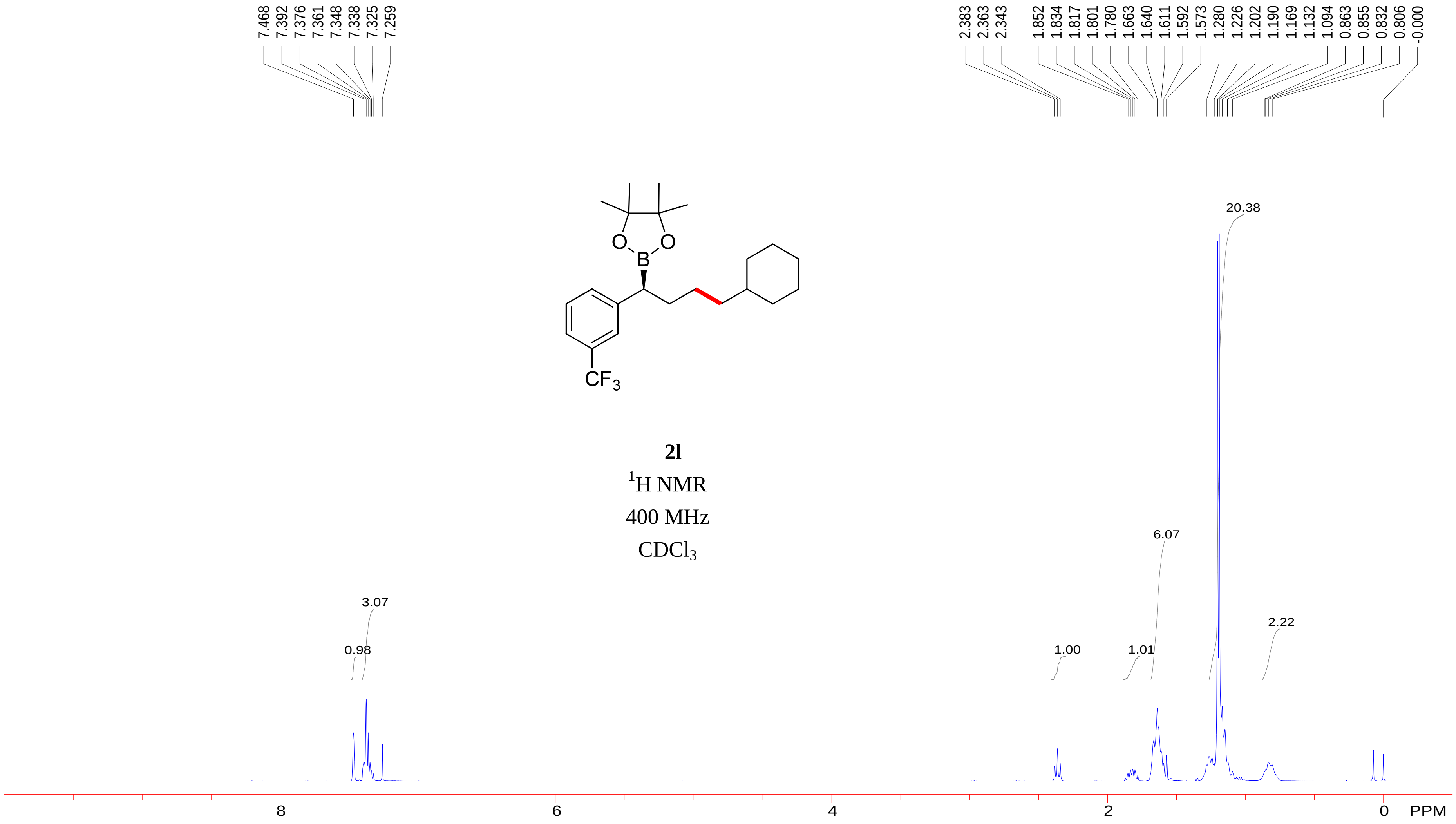

Supplementary Figure 149. ^{1}H NMR spectrum for **2l**

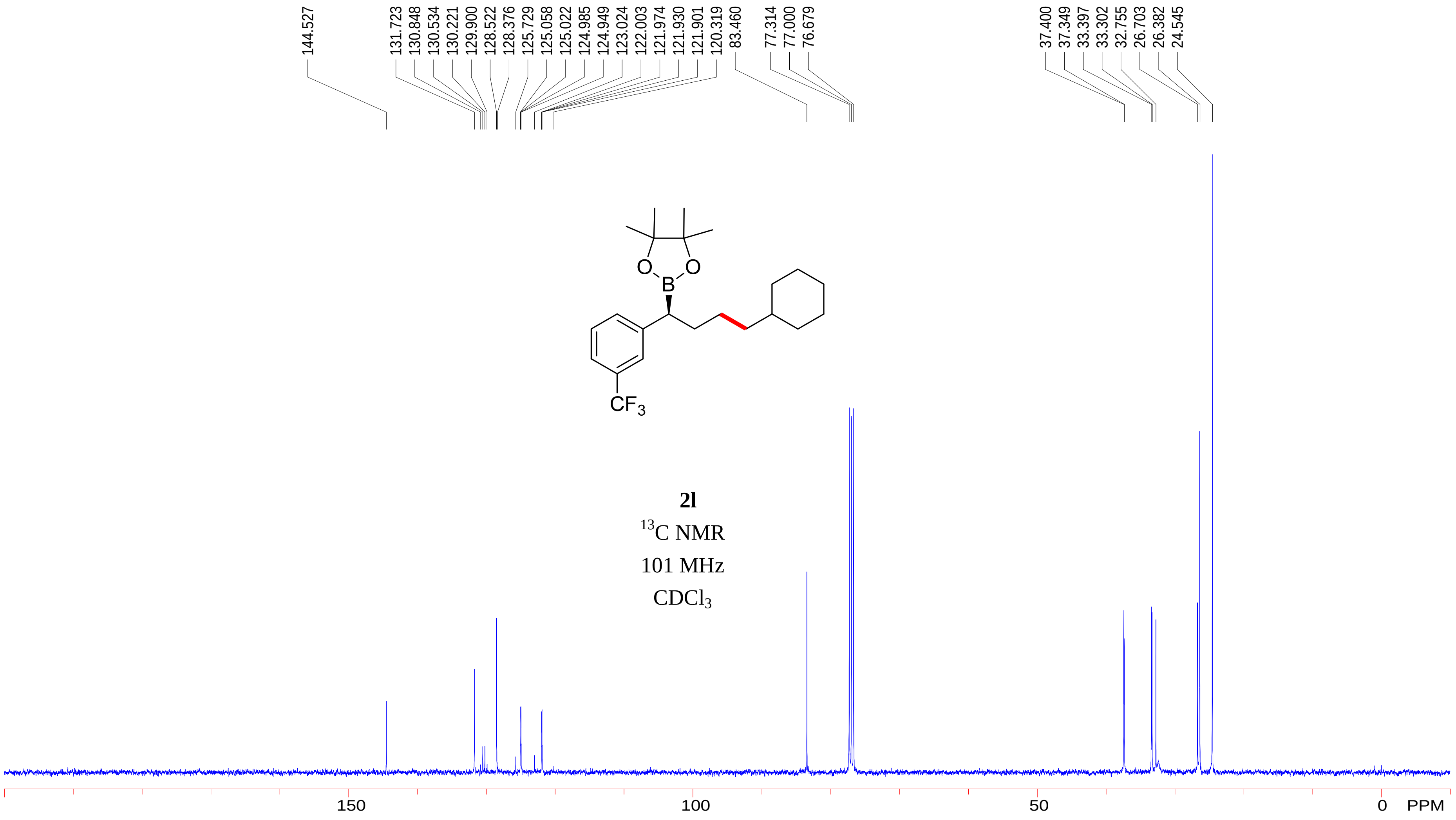

Supplementary Figure 150. ^{13}C NMR spectrum for **2l**

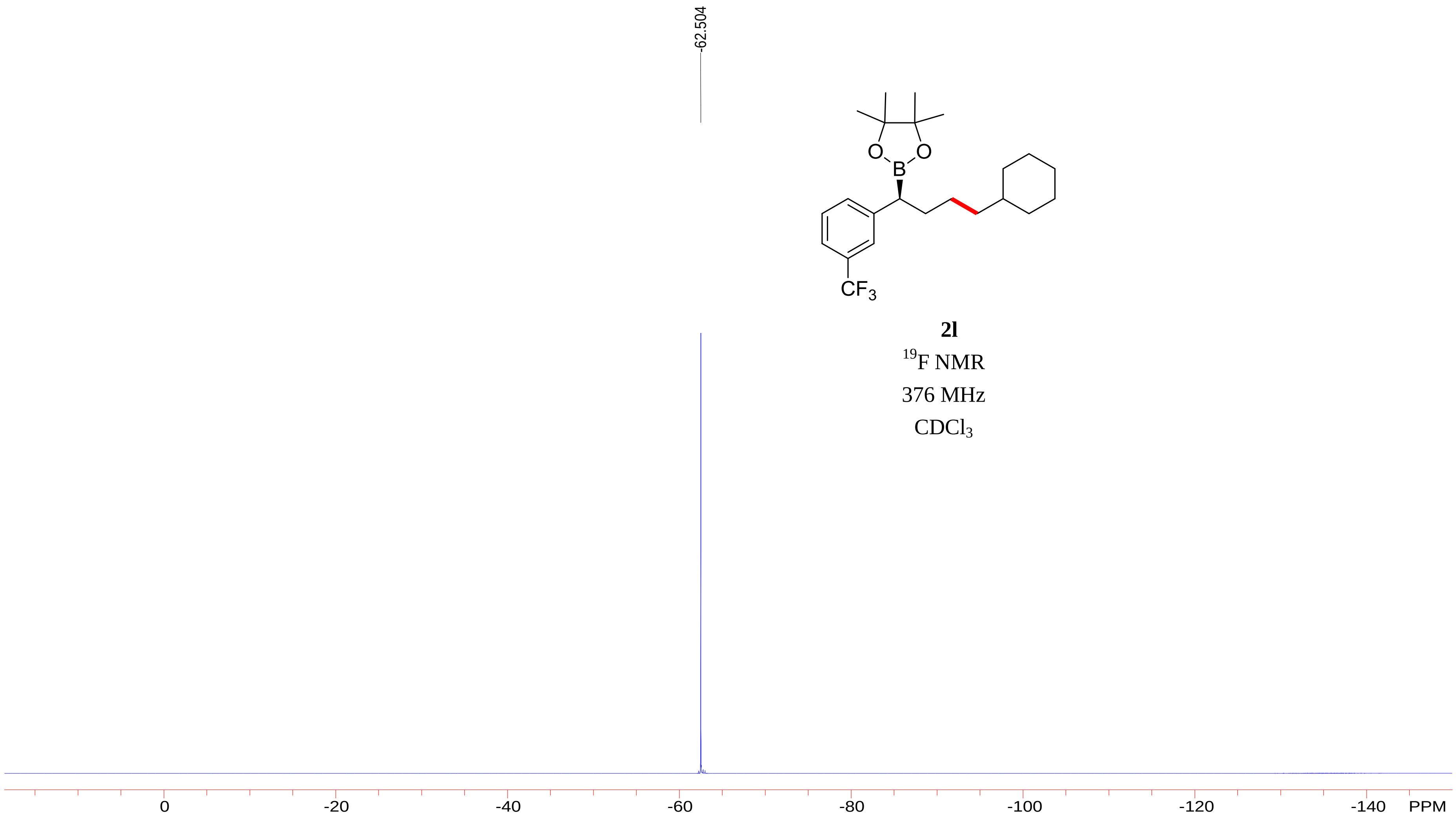

Supplementary Figure 151. ^{19}F NMR spectrum for **2l**

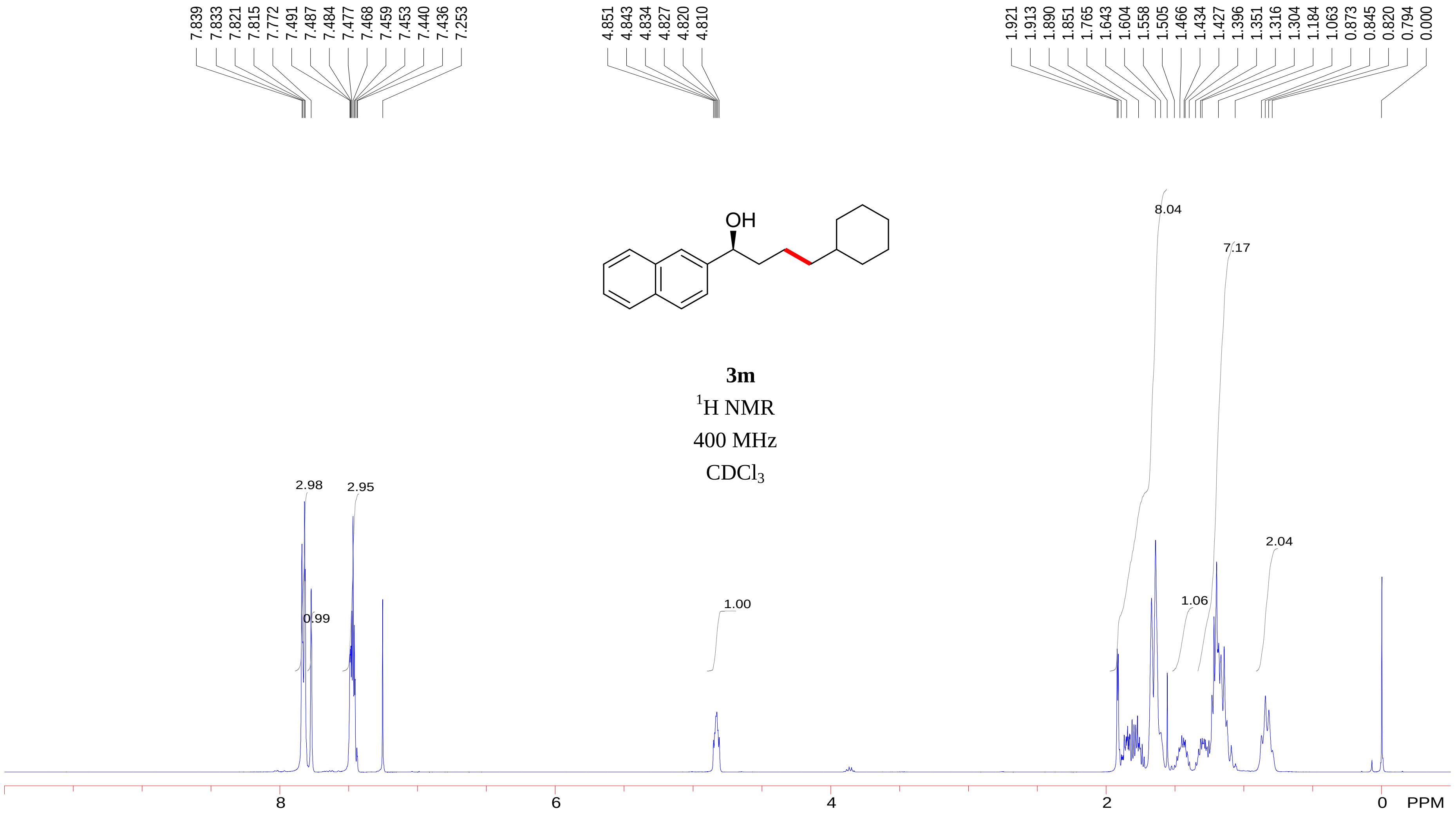

Supplementary Figure 152. ^{1}H NMR spectrum for **3m**

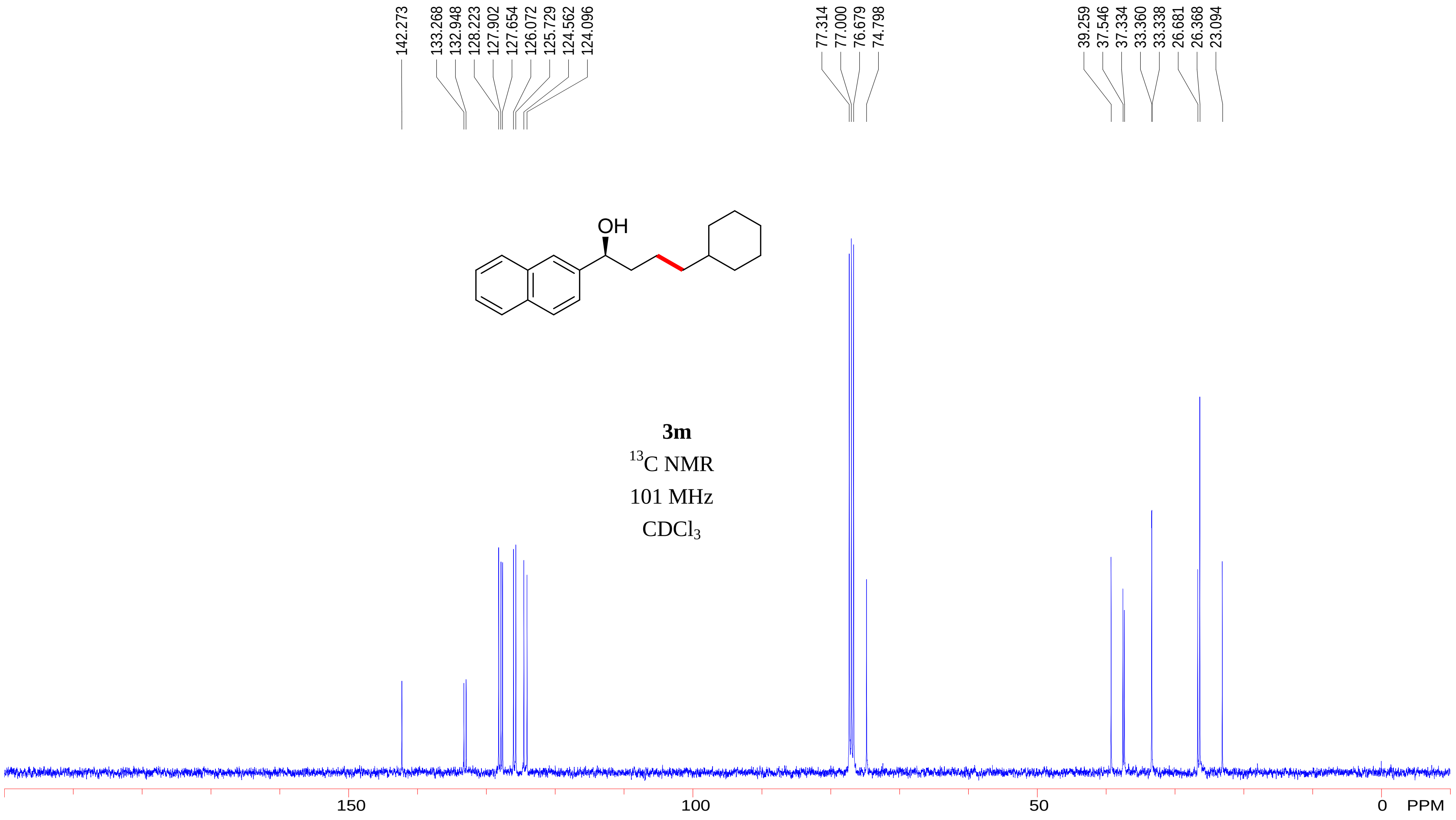

Supplementary Figure 153. ^{13}C NMR spectrum for **3m**

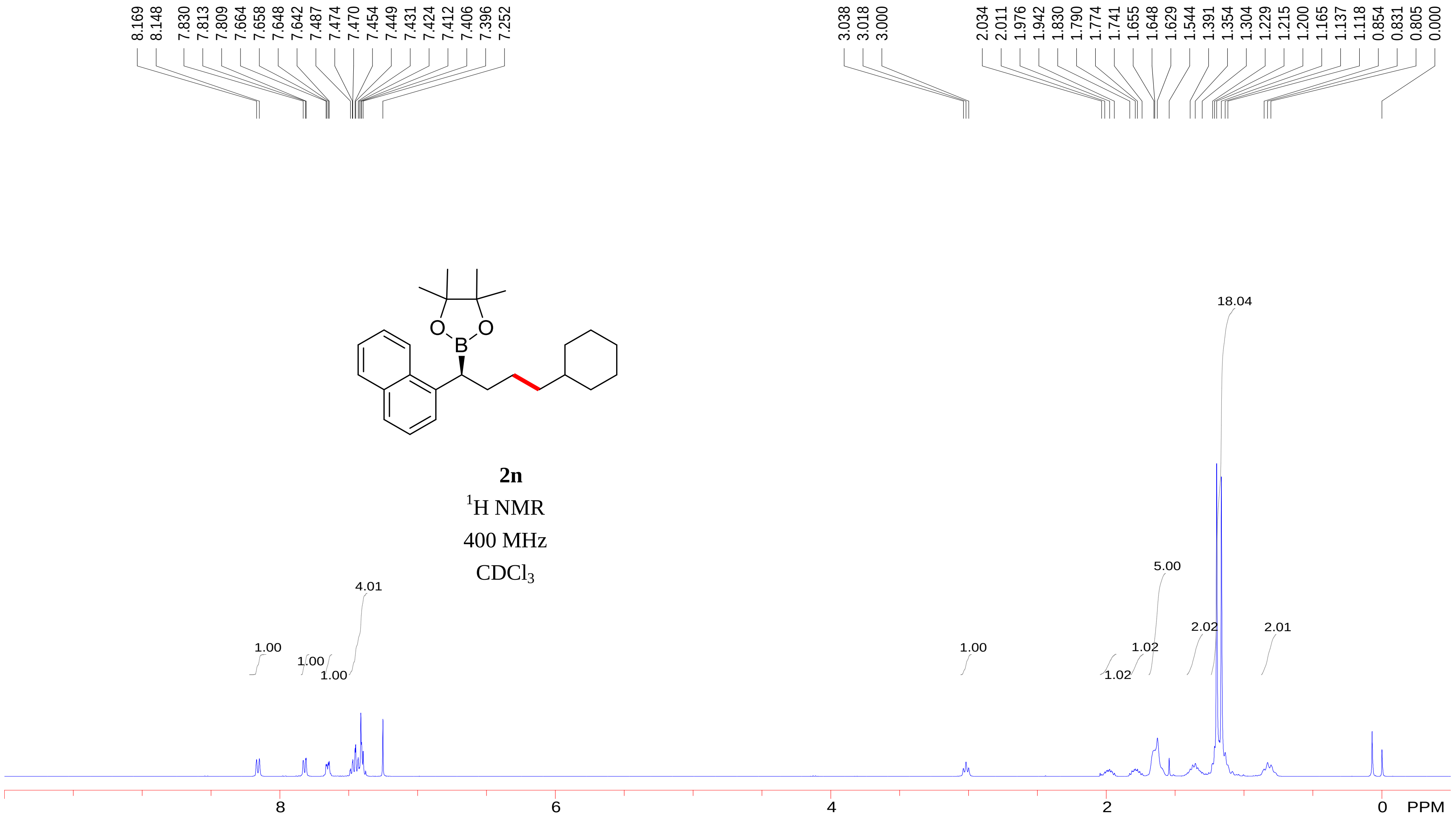

Supplementary Figure 154. ^{1}H NMR spectrum for **2n**

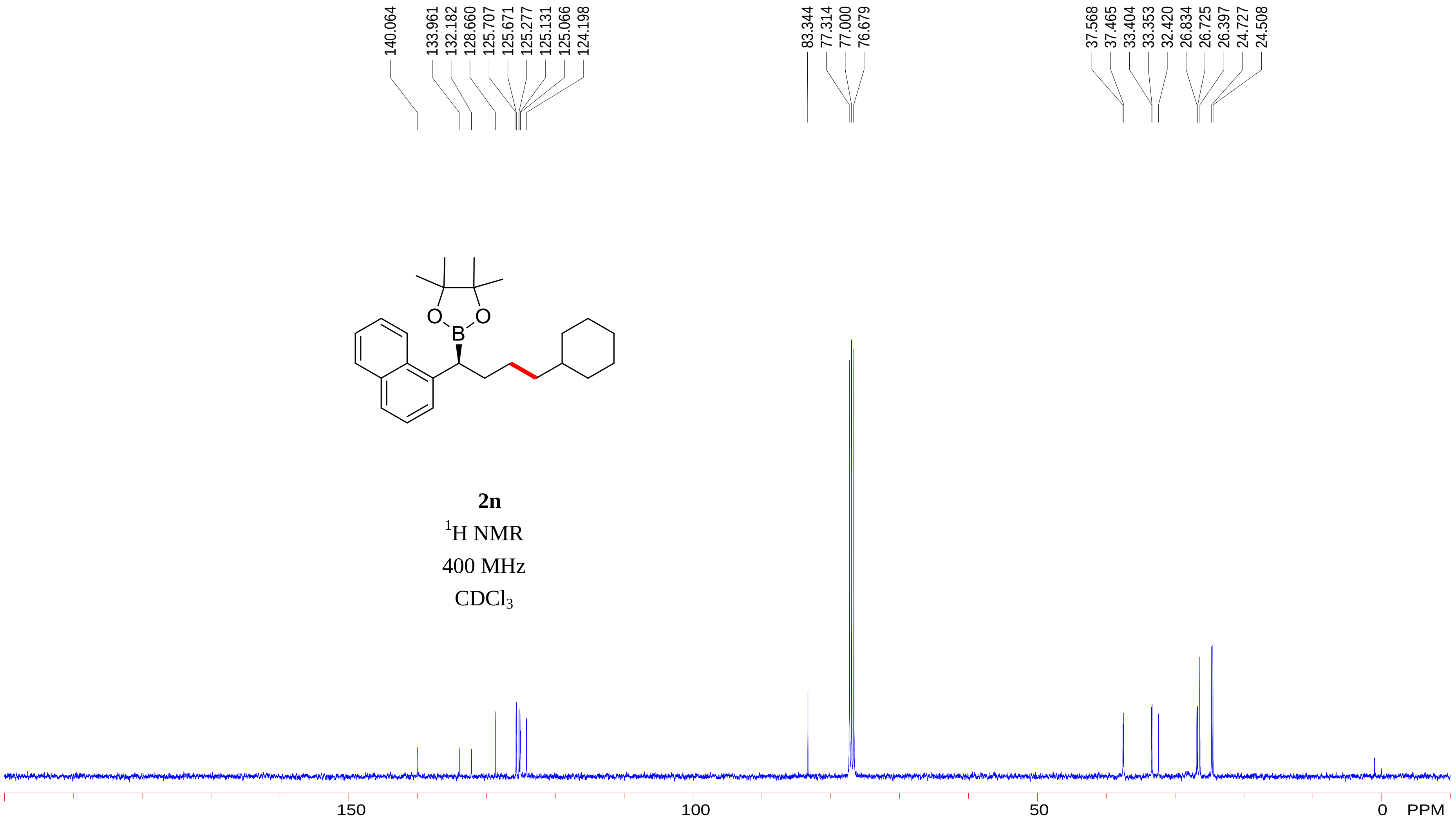

Supplementary Figure 155. ^{13}C NMR spectrum for **2n**

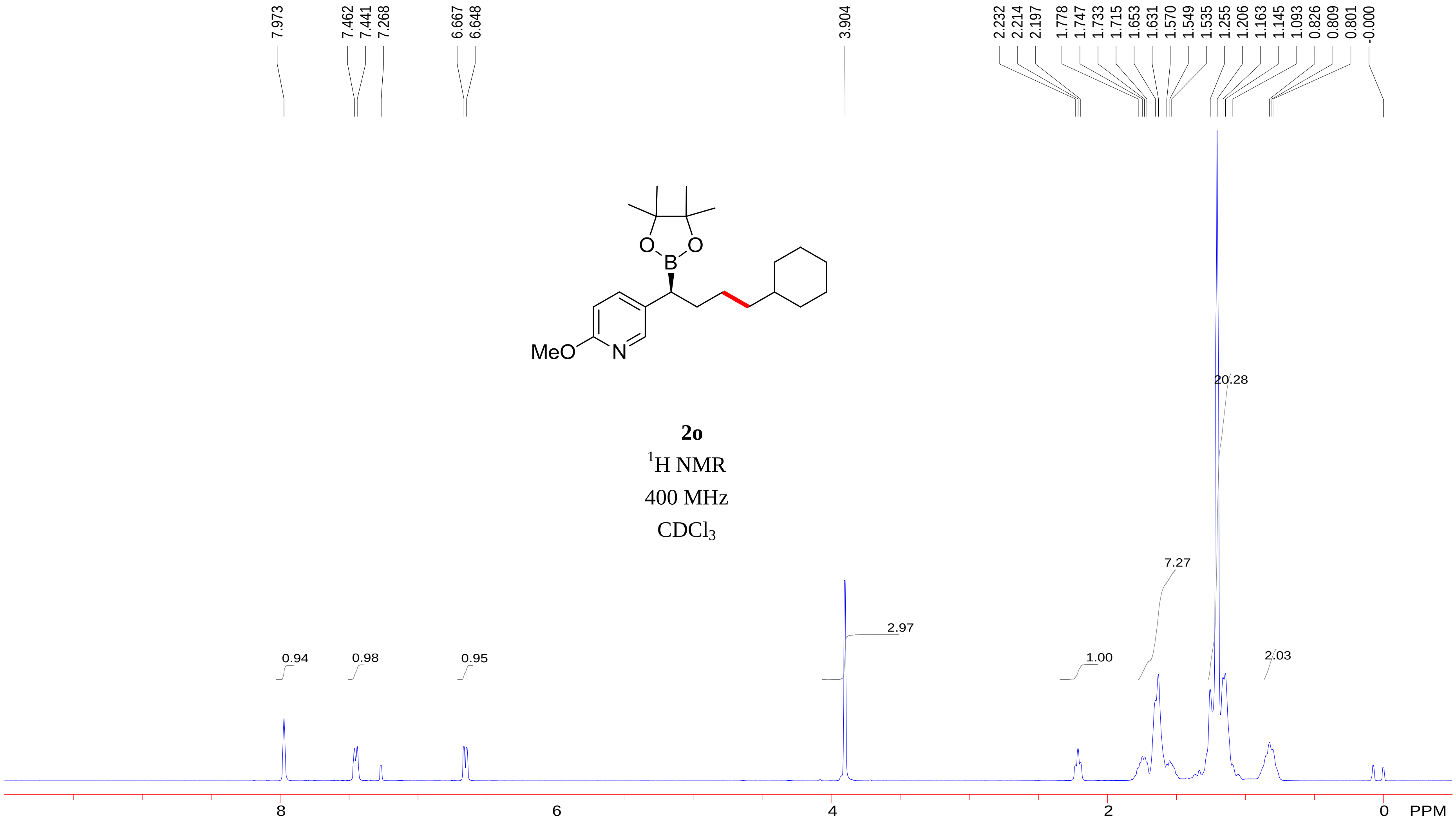

Supplementary Figure 156. ^{1}H NMR spectrum for **2o**

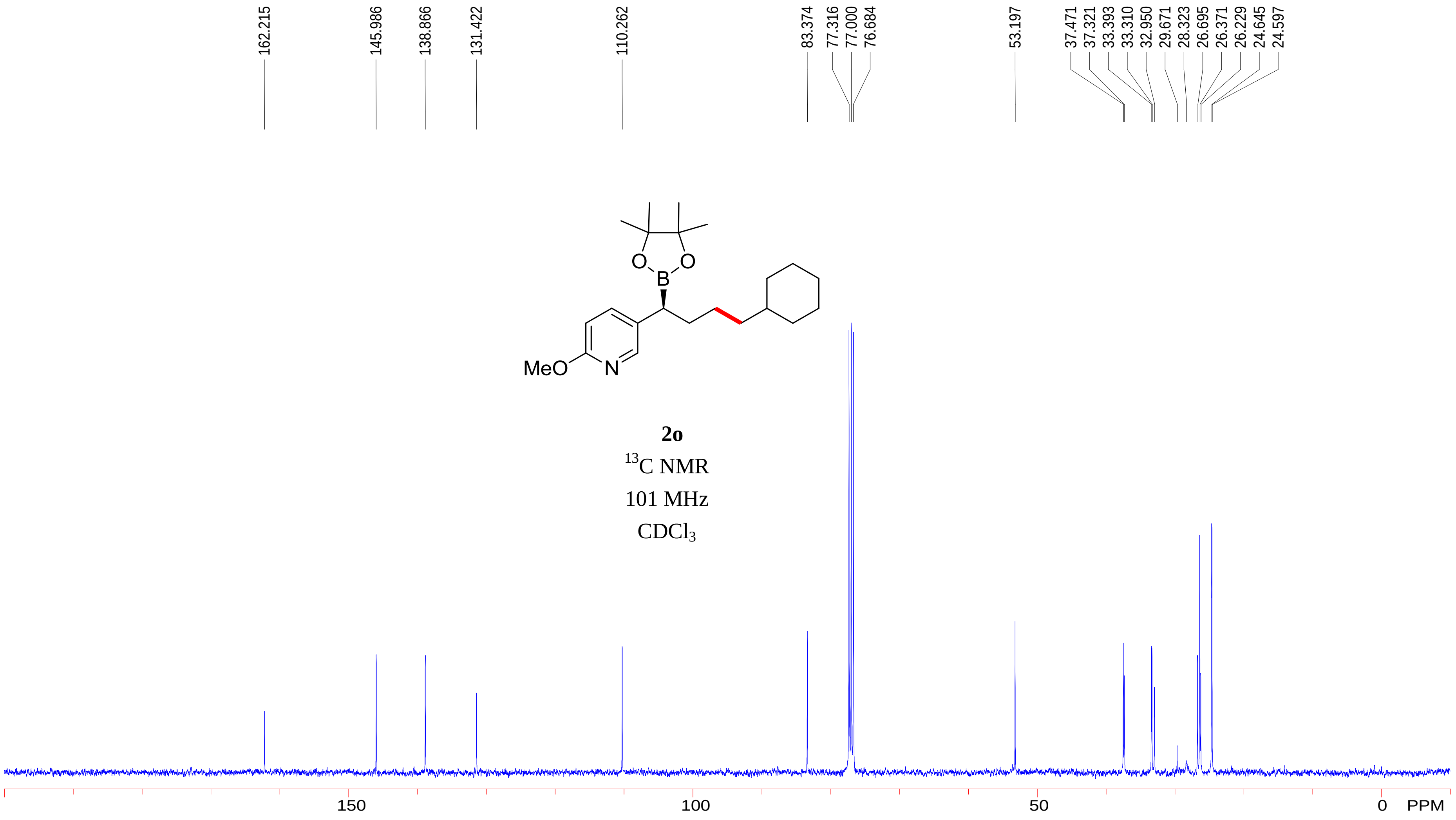

Supplementary Figure 157. ^{13}C NMR spectrum for **2o**

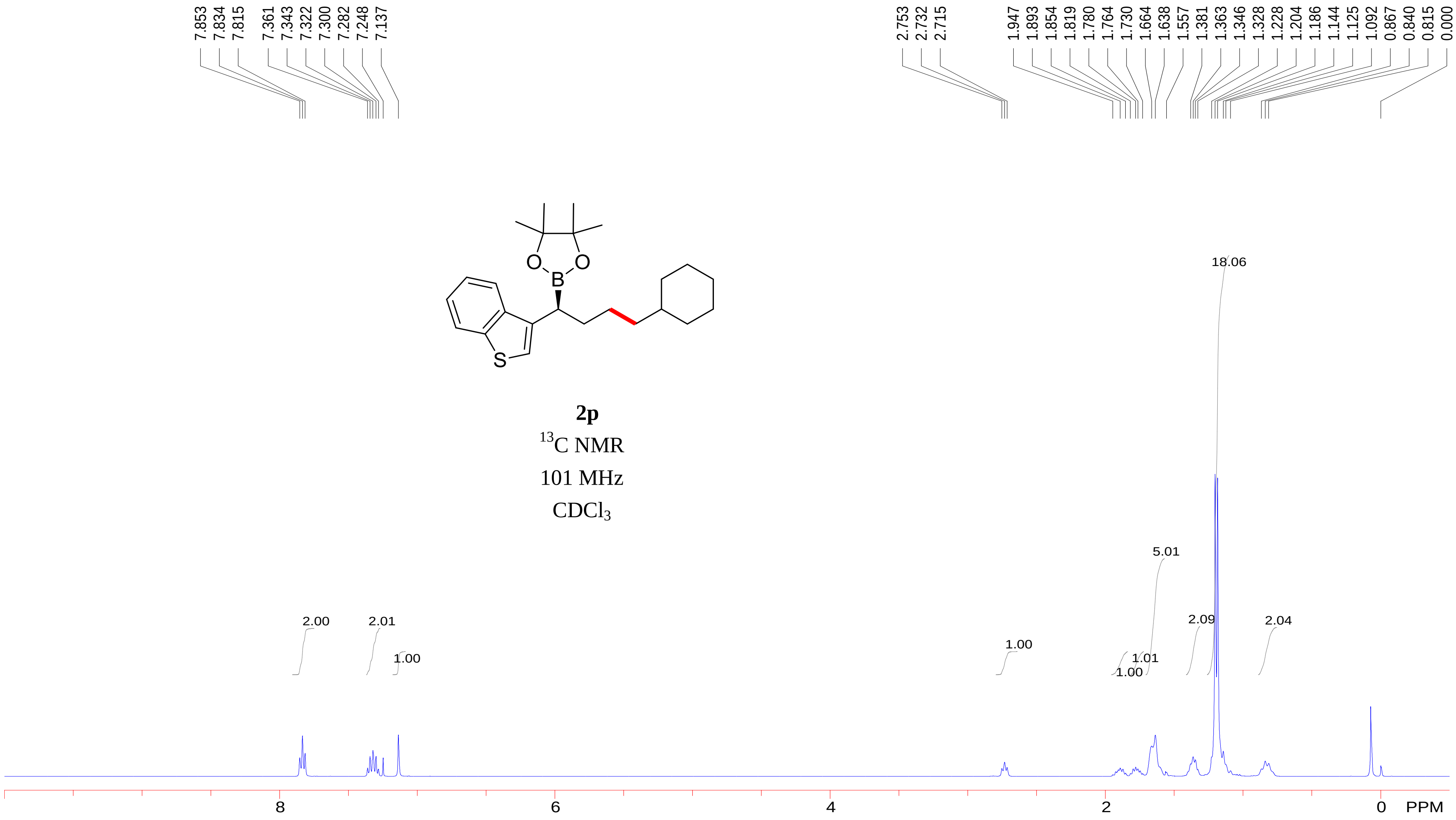

Supplementary Figure 158. ^{1}H NMR spectrum for **2p**

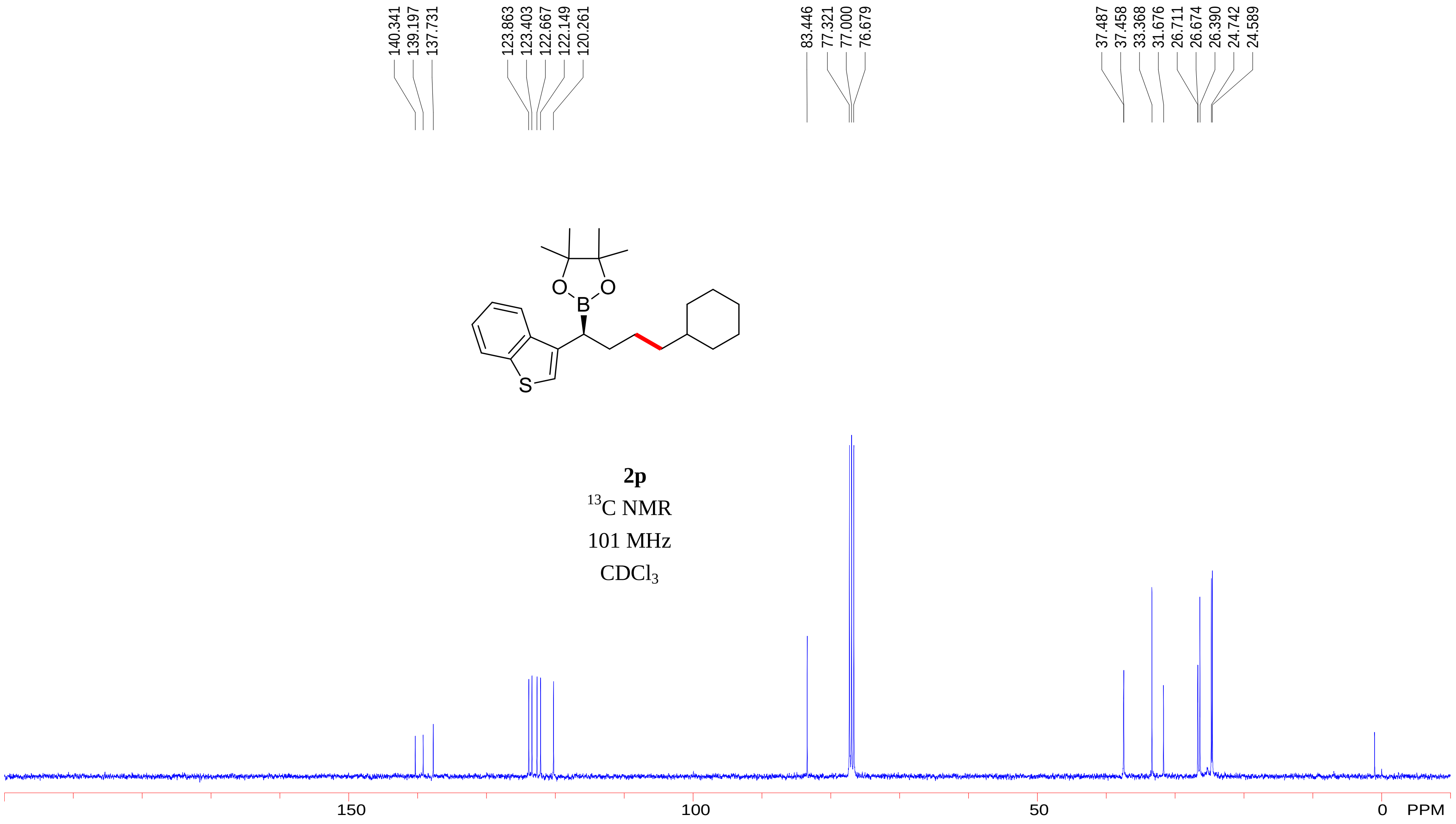

Supplementary Figure 159. ^{13}C NMR spectrum for **2p**

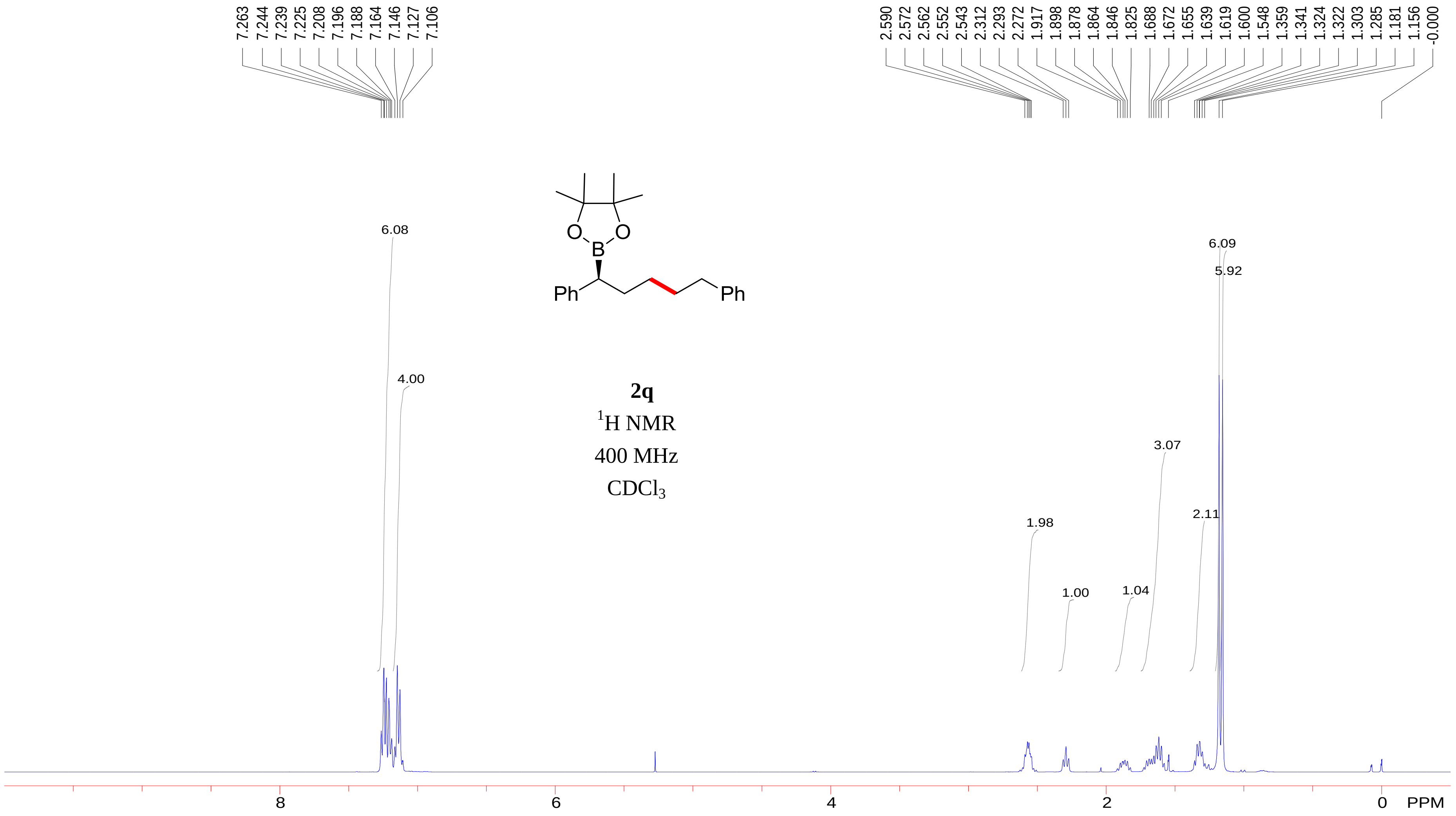

Supplementary Figure 160. ^{1}H NMR spectrum for **2q**

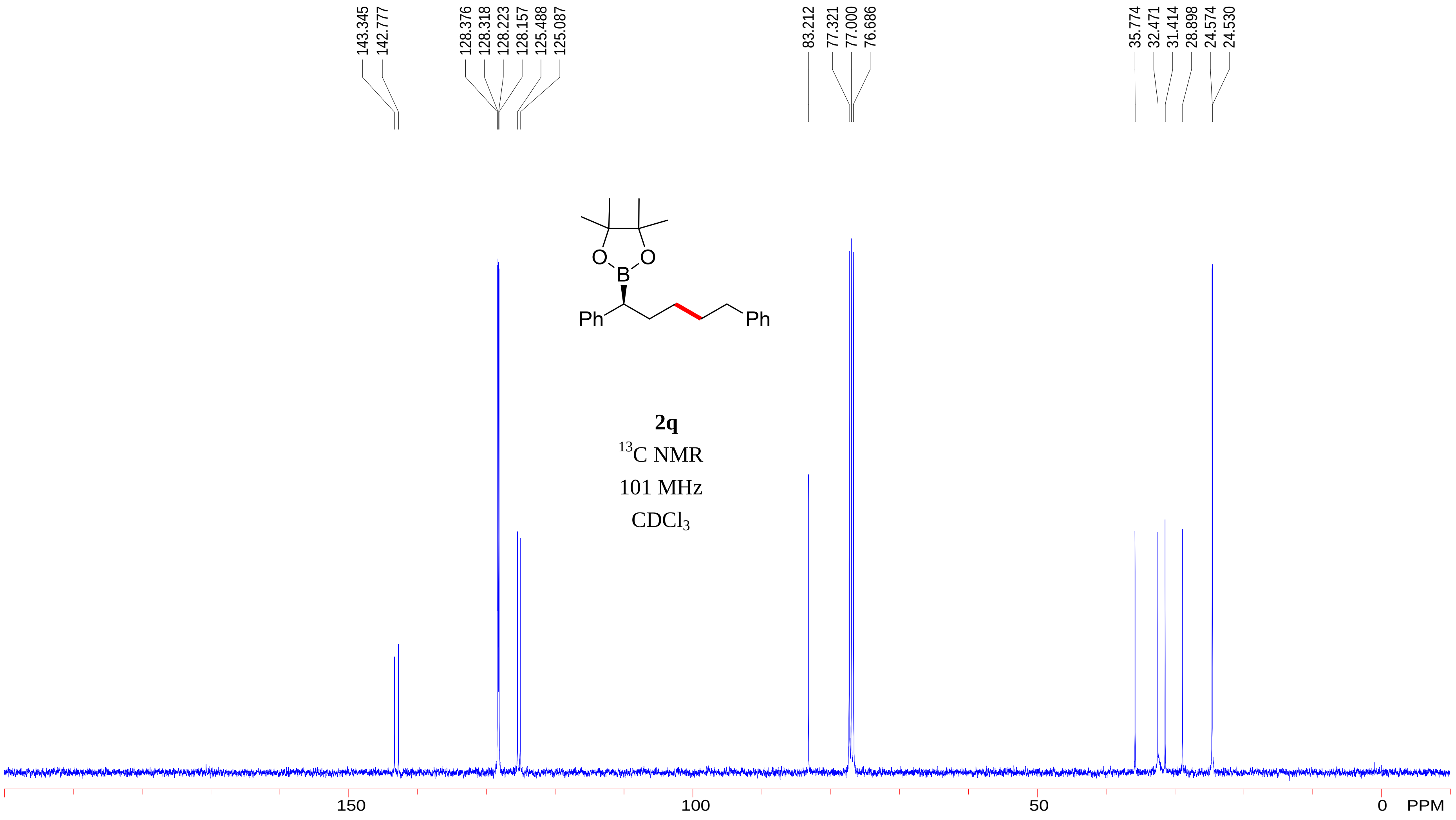

Supplementary Figure 161. ^{13}C NMR spectrum for **2q**

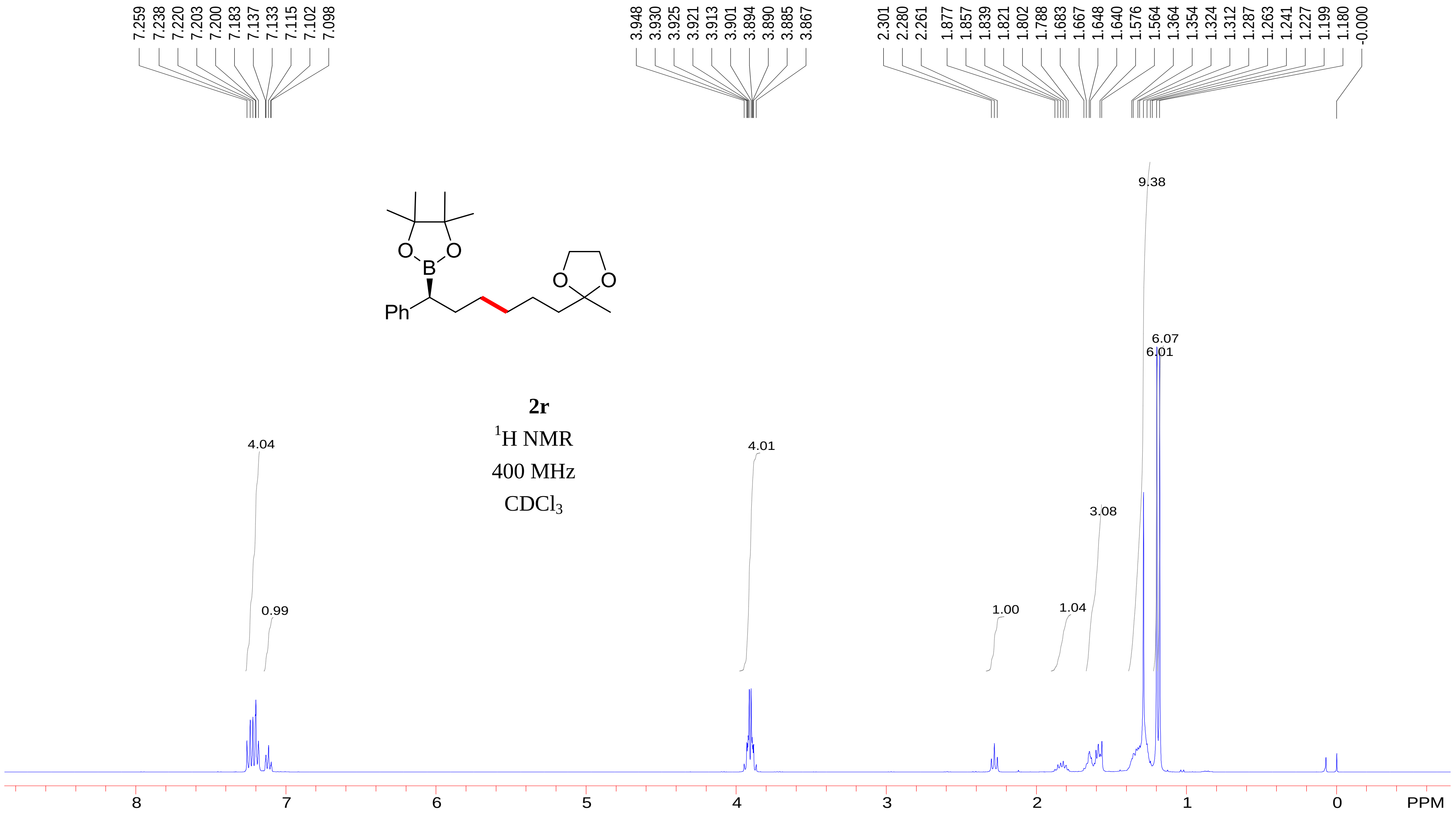

Supplementary Figure 162. ^{1}H NMR spectrum for **2r**

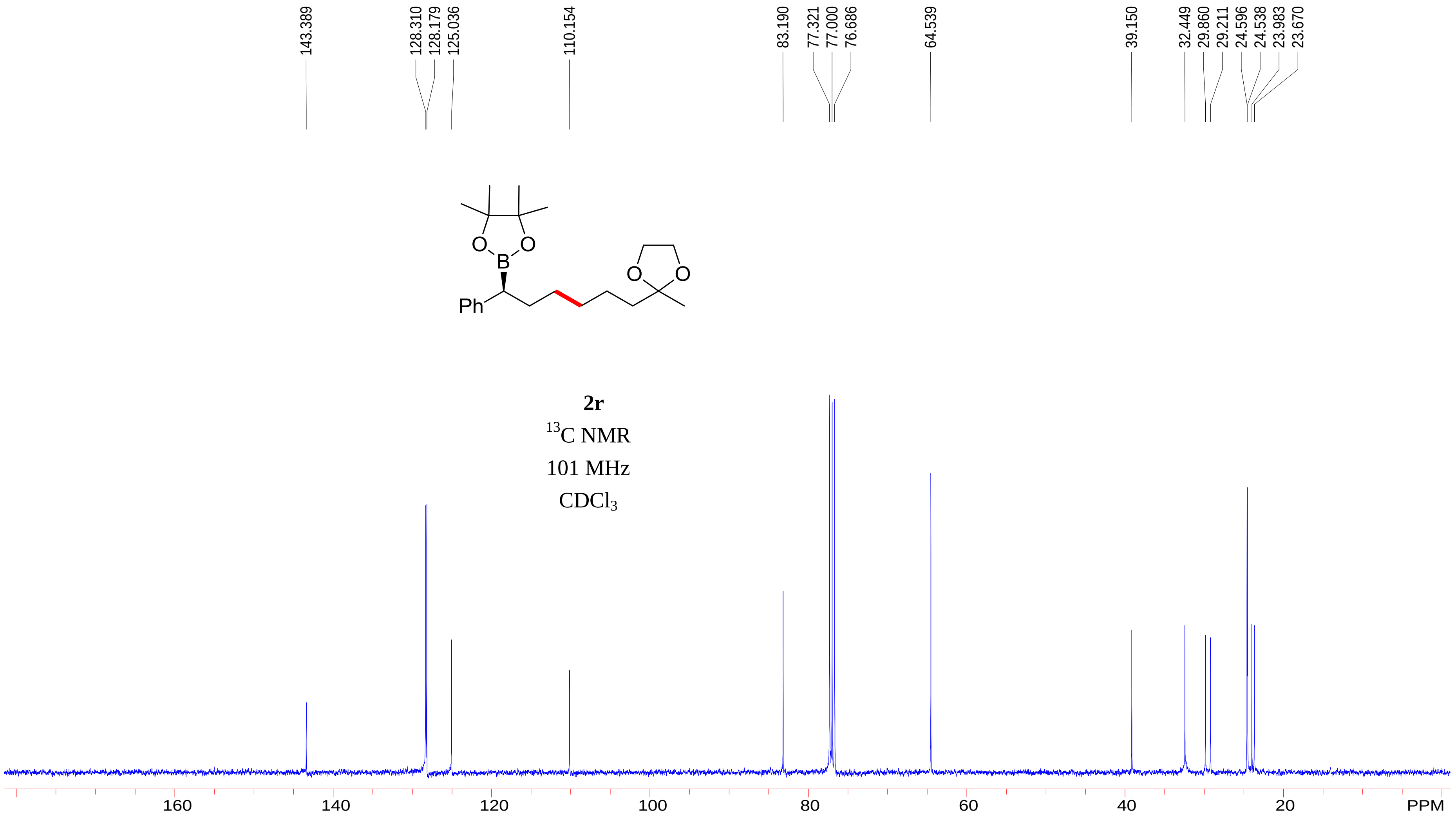

Supplementary Figure 163. ^{13}C NMR spectrum for **2r**

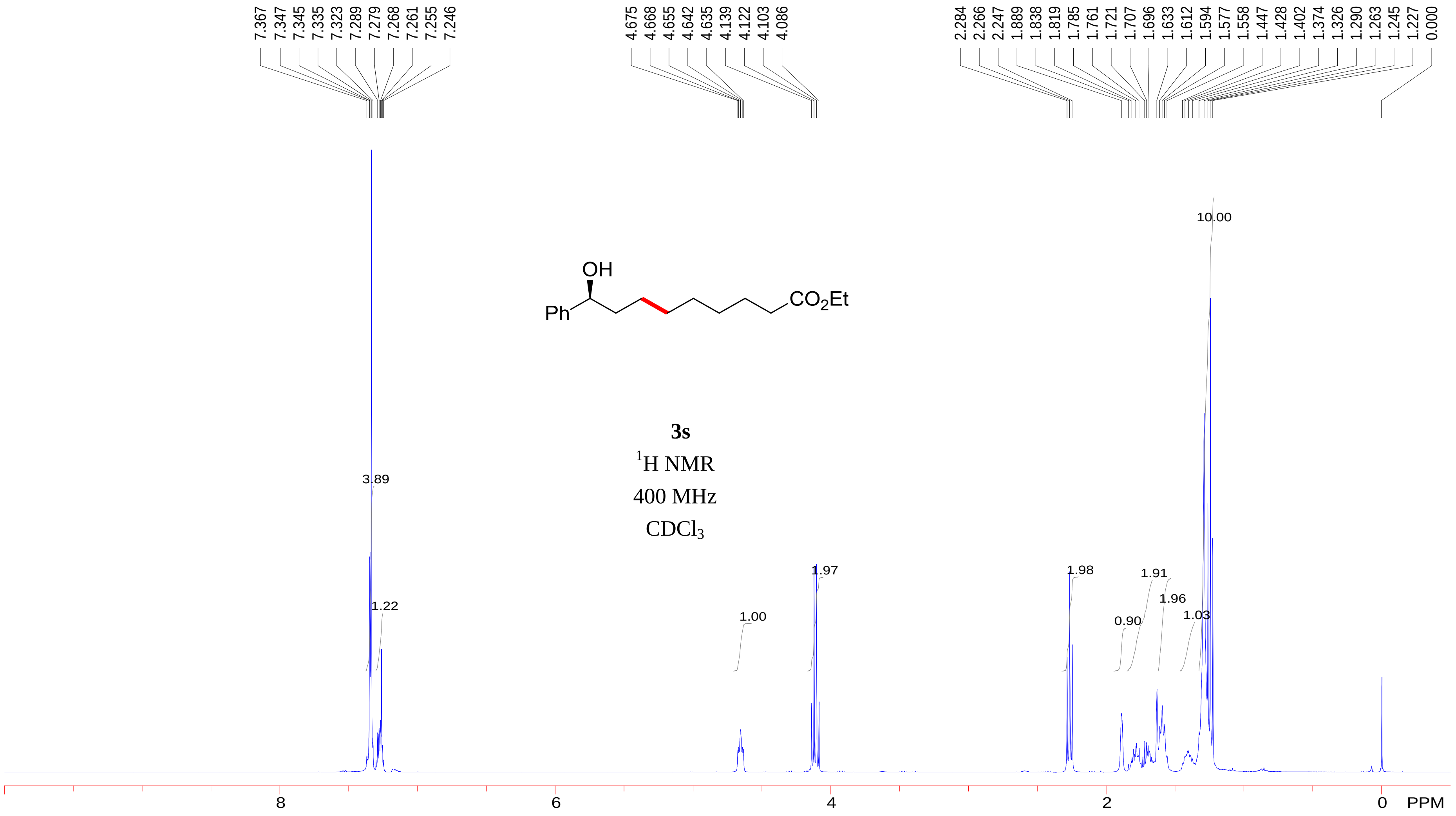

Supplementary Figure 164. ^{1}H NMR spectrum for **3s**

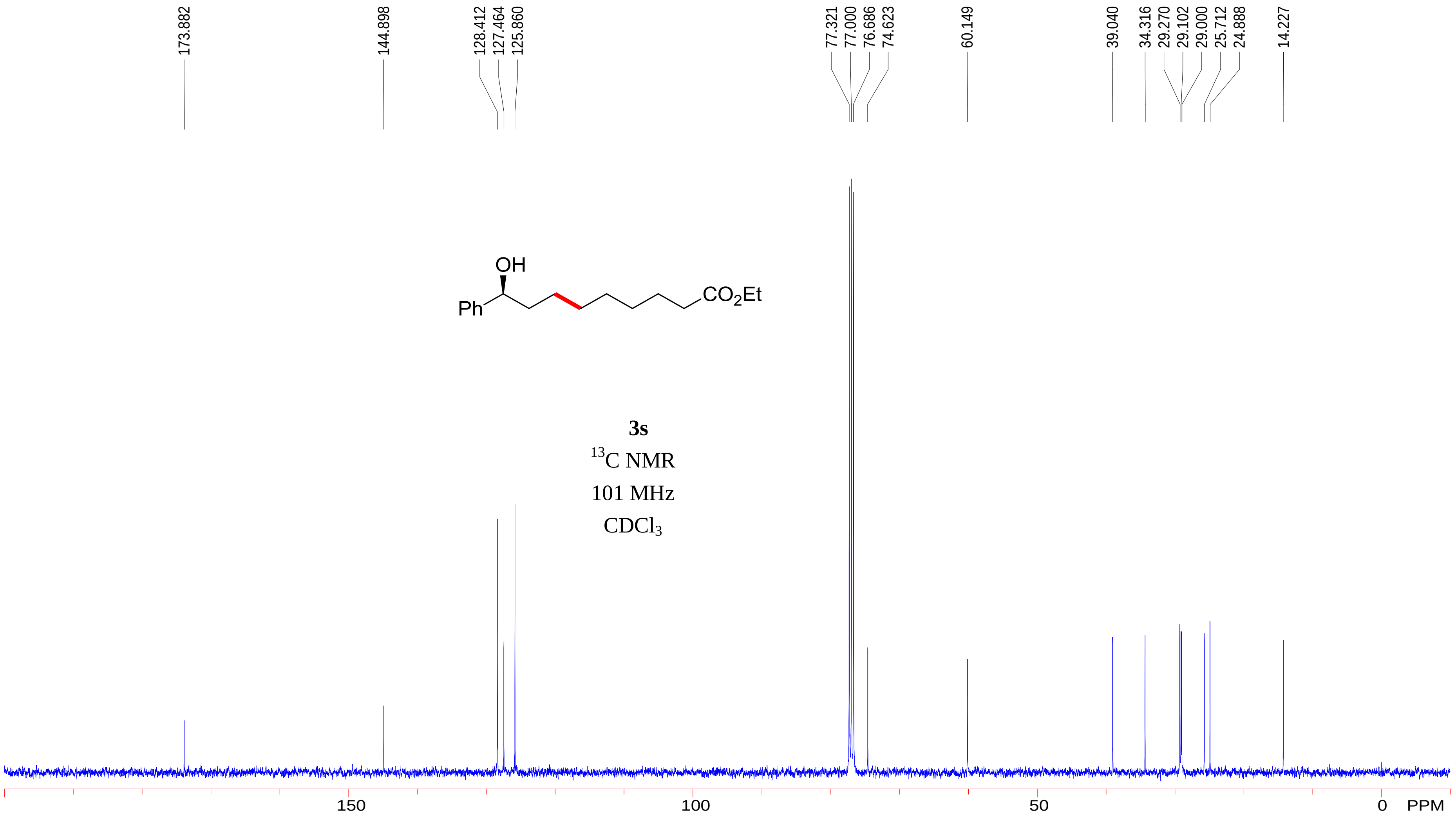

Supplementary Figure 165. ^{13}C NMR spectrum for **3s**

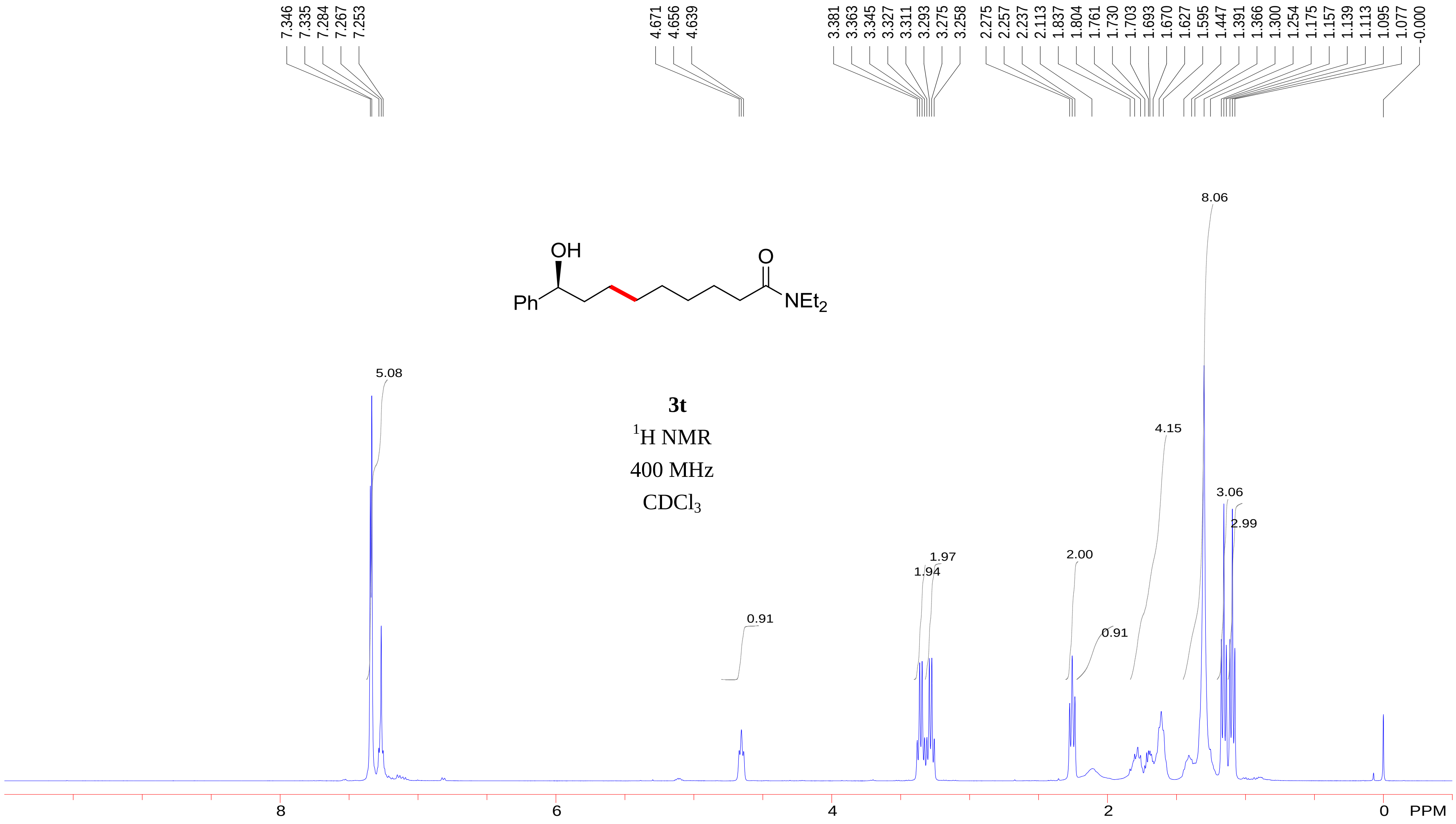

Supplementary Figure 166. ^{1}H NMR spectrum for **3t**

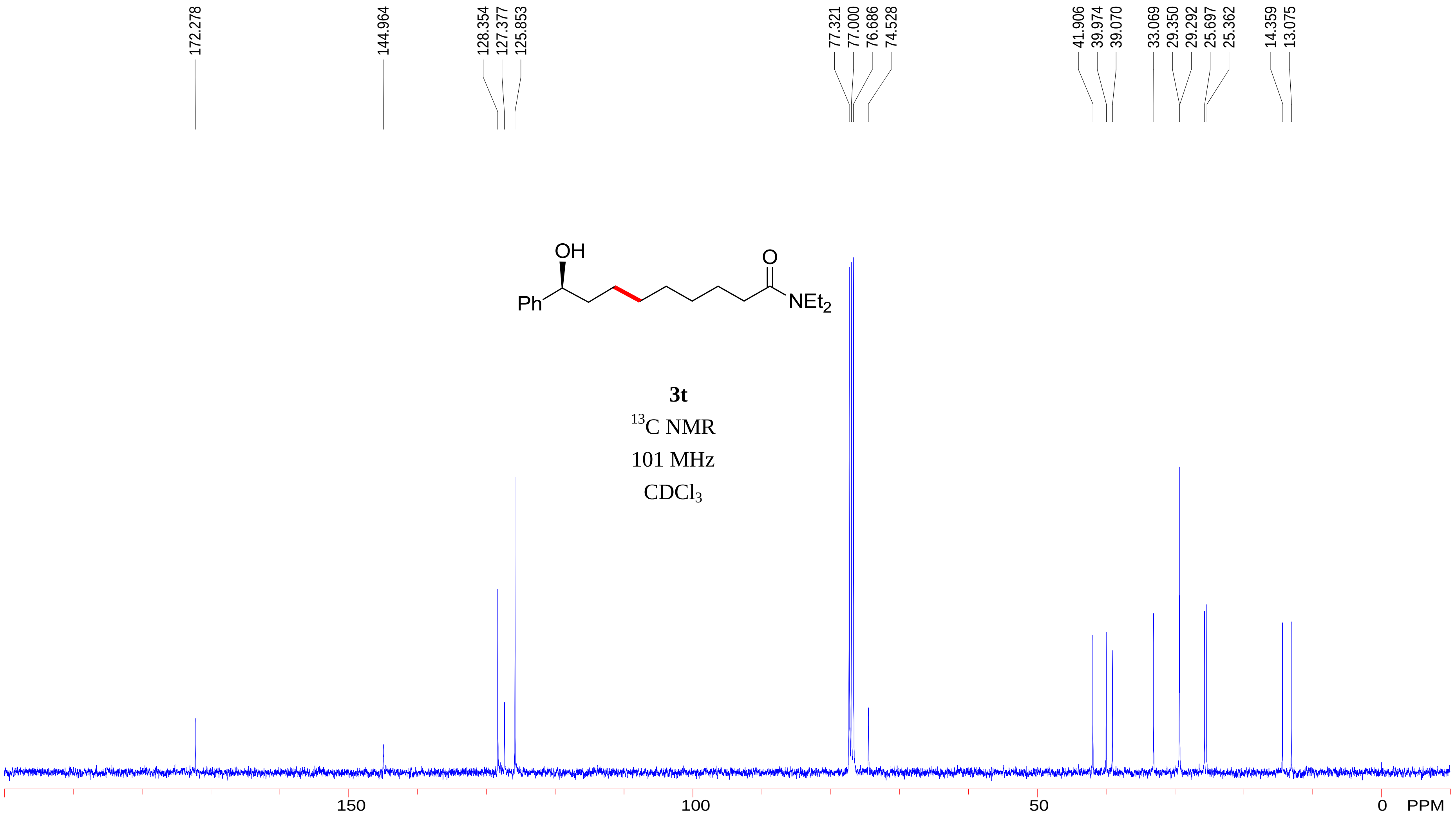

Supplementary Figure 167. ^{13}C NMR spectrum for **3t**

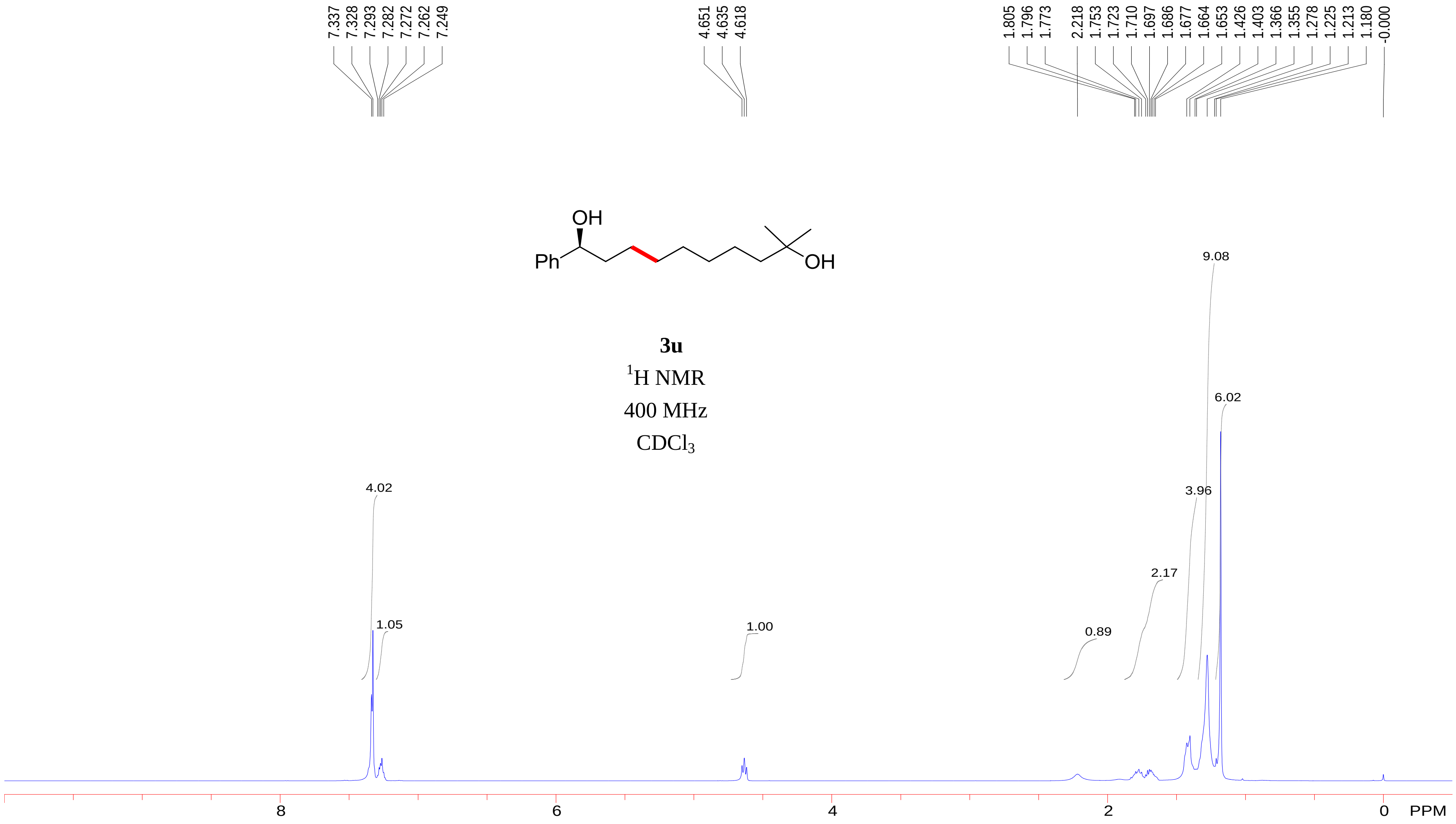

Supplementary Figure 168. ^{1}H NMR spectrum for **3u**

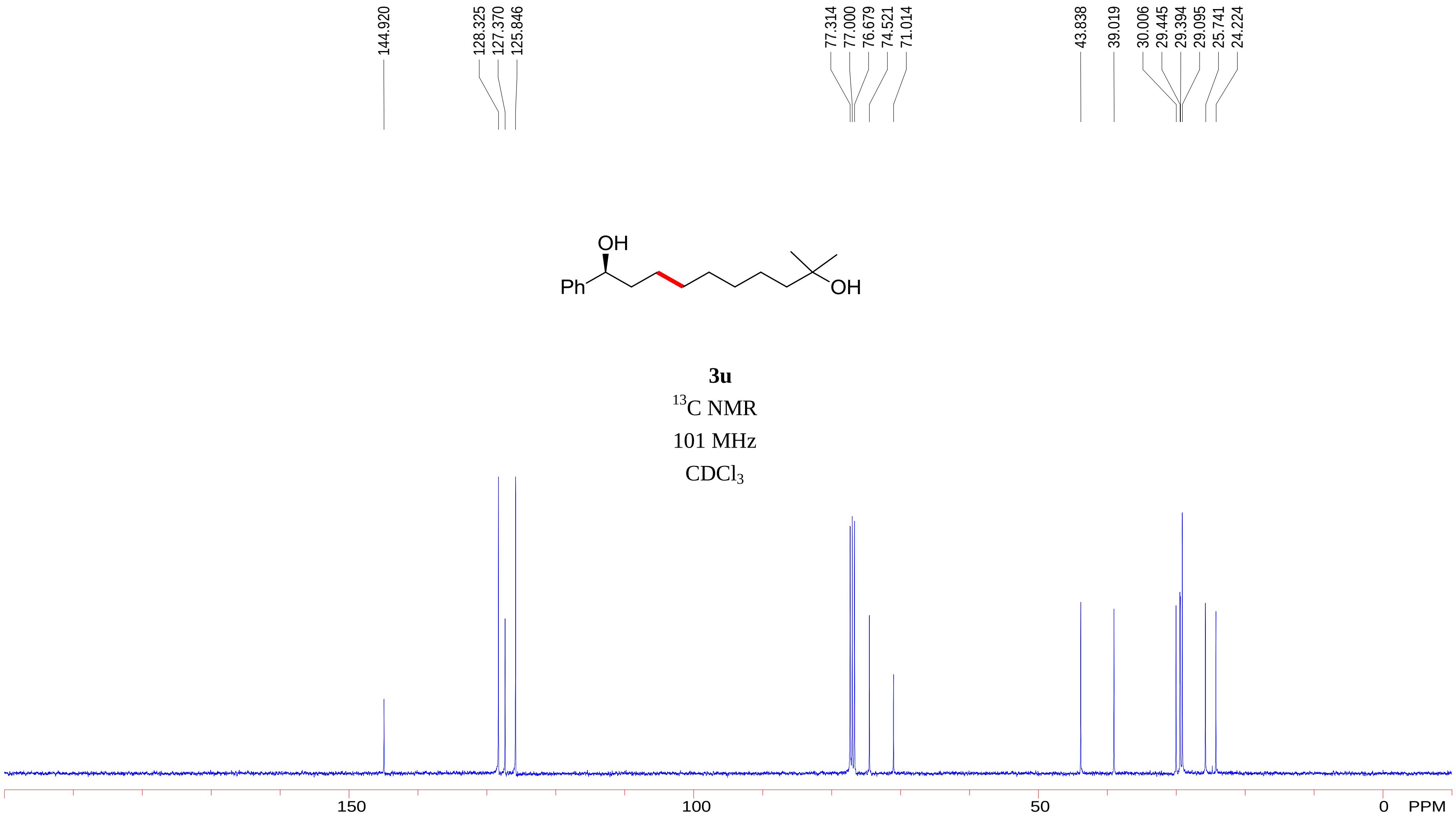

3u
^{13}C NMR
101 MHz
$CDCl_3$

Supplementary Figure 169. ^{13}C NMR spectrum for **3u**

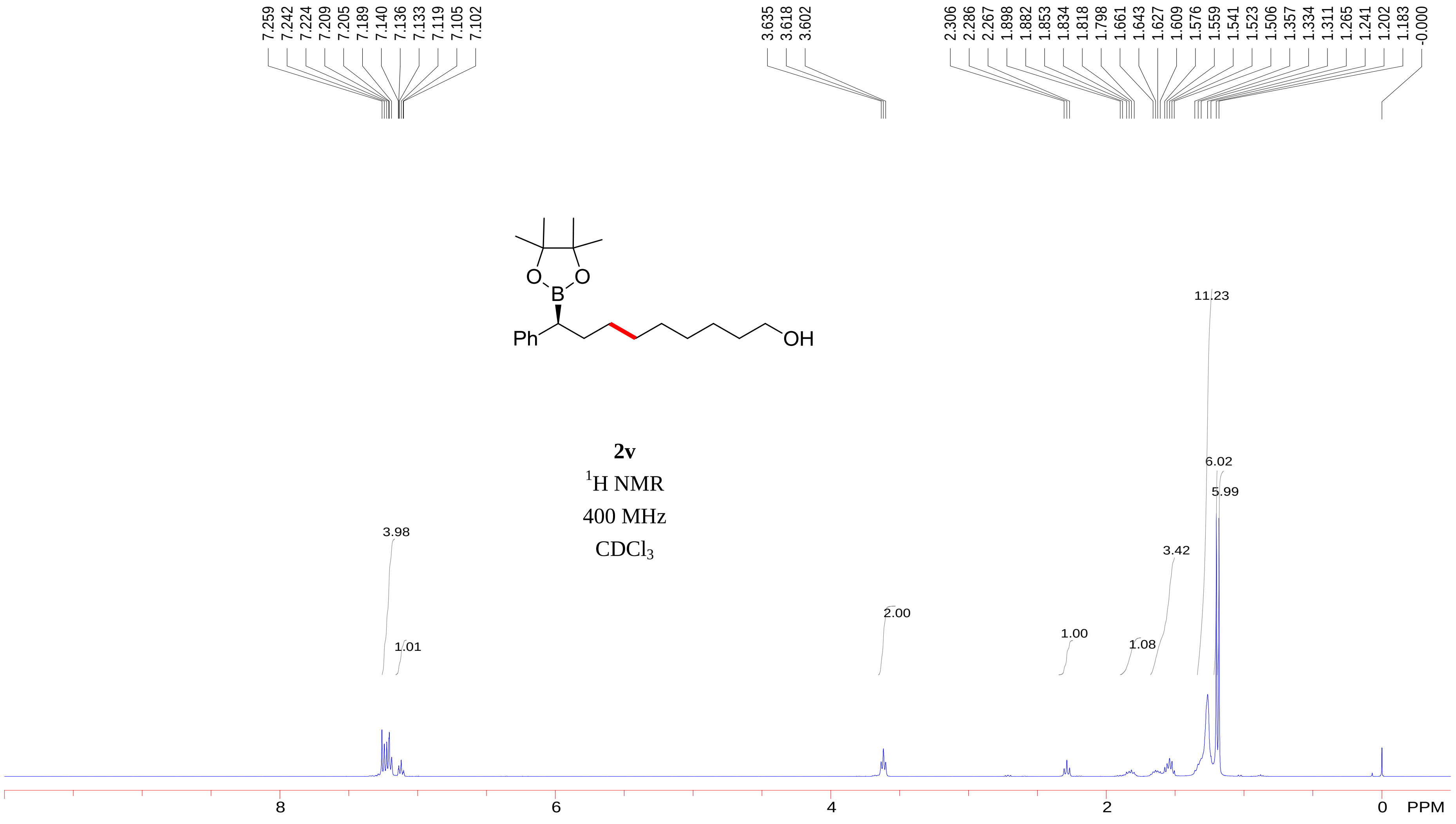

Supplementary Figure 170. ^{1}H NMR spectrum for **2v**

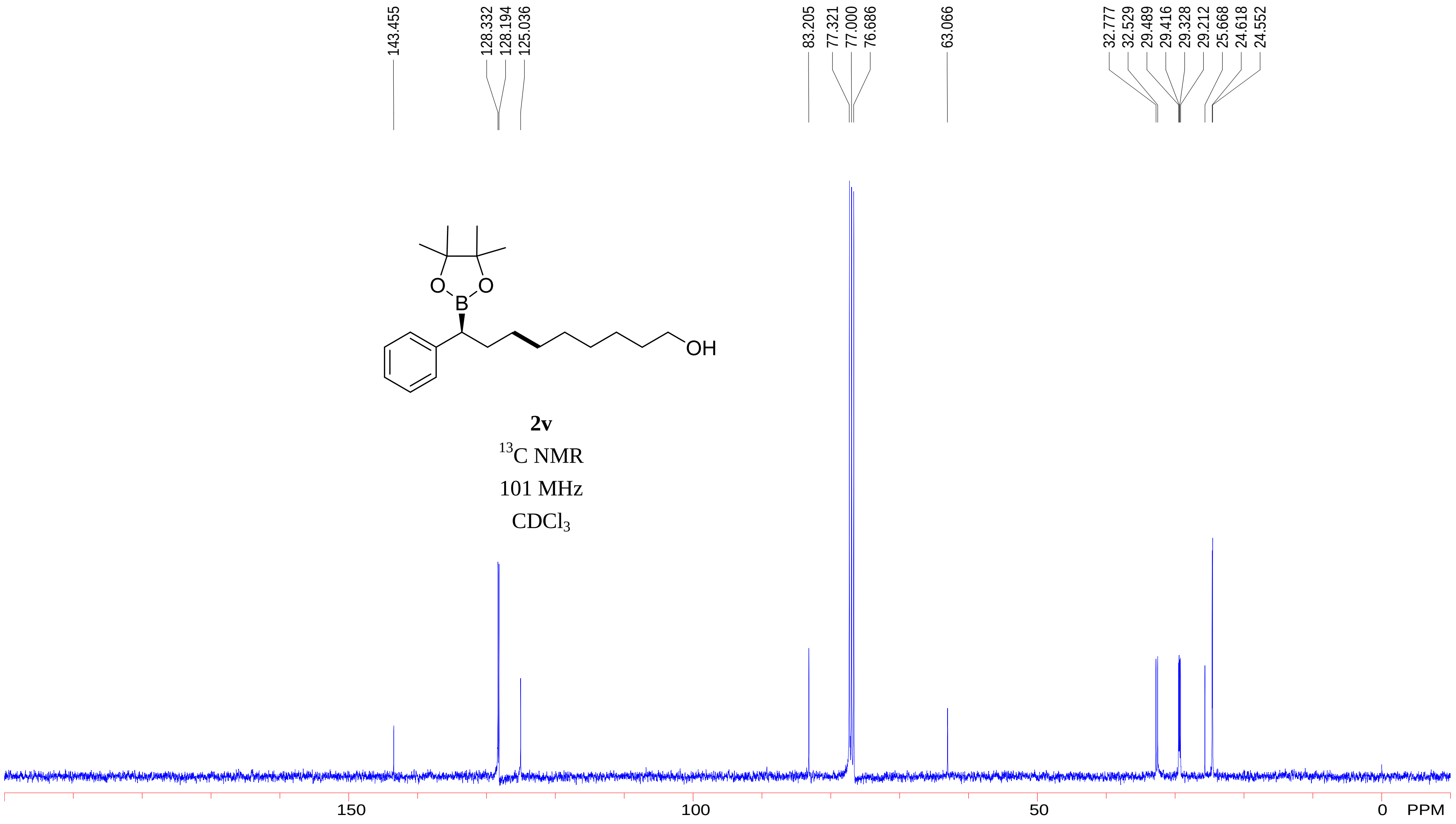

Supplementary Figure 171. ^{1}H NMR spectrum for **2v**

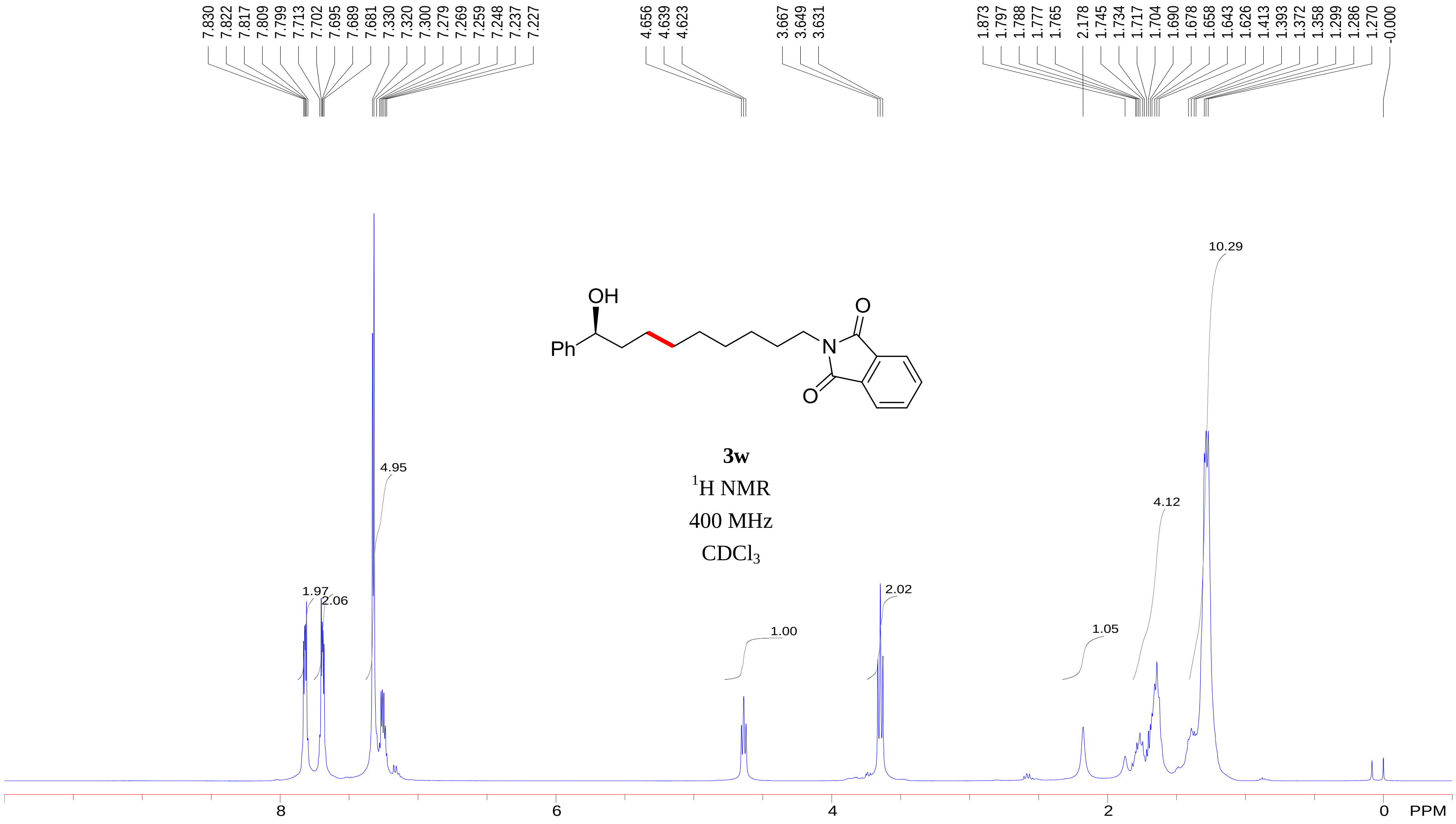

Supplementary Figure 172. ^{1}H NMR spectrum for **3w**

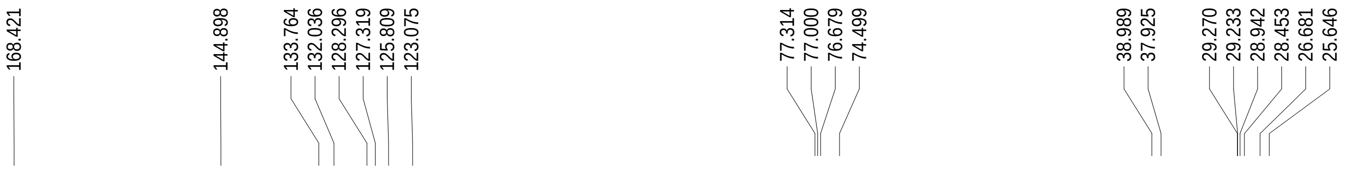

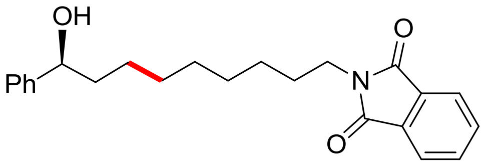

3w

^{13}C NMR

101 MHz

$CDCl_3$

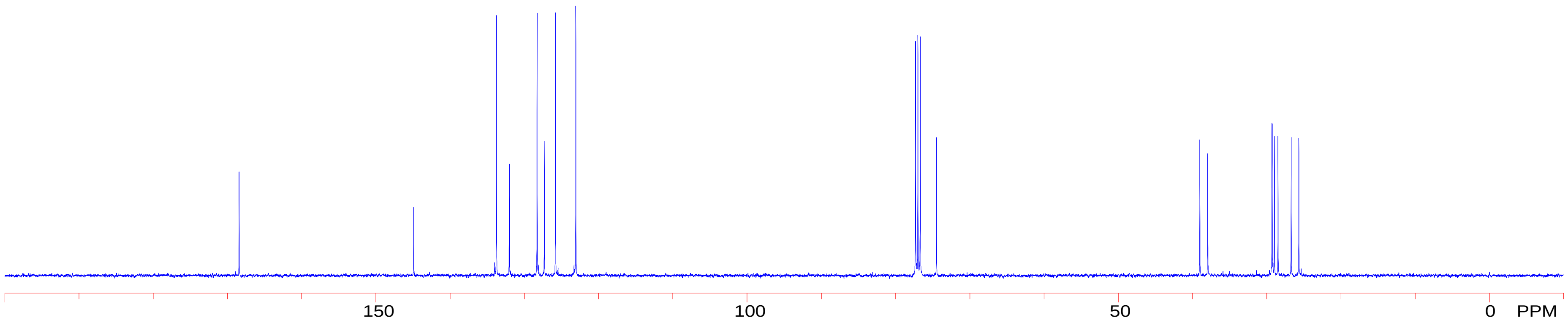

Supplementary Figure 173. ^{13}C NMR spectrum for **3w**

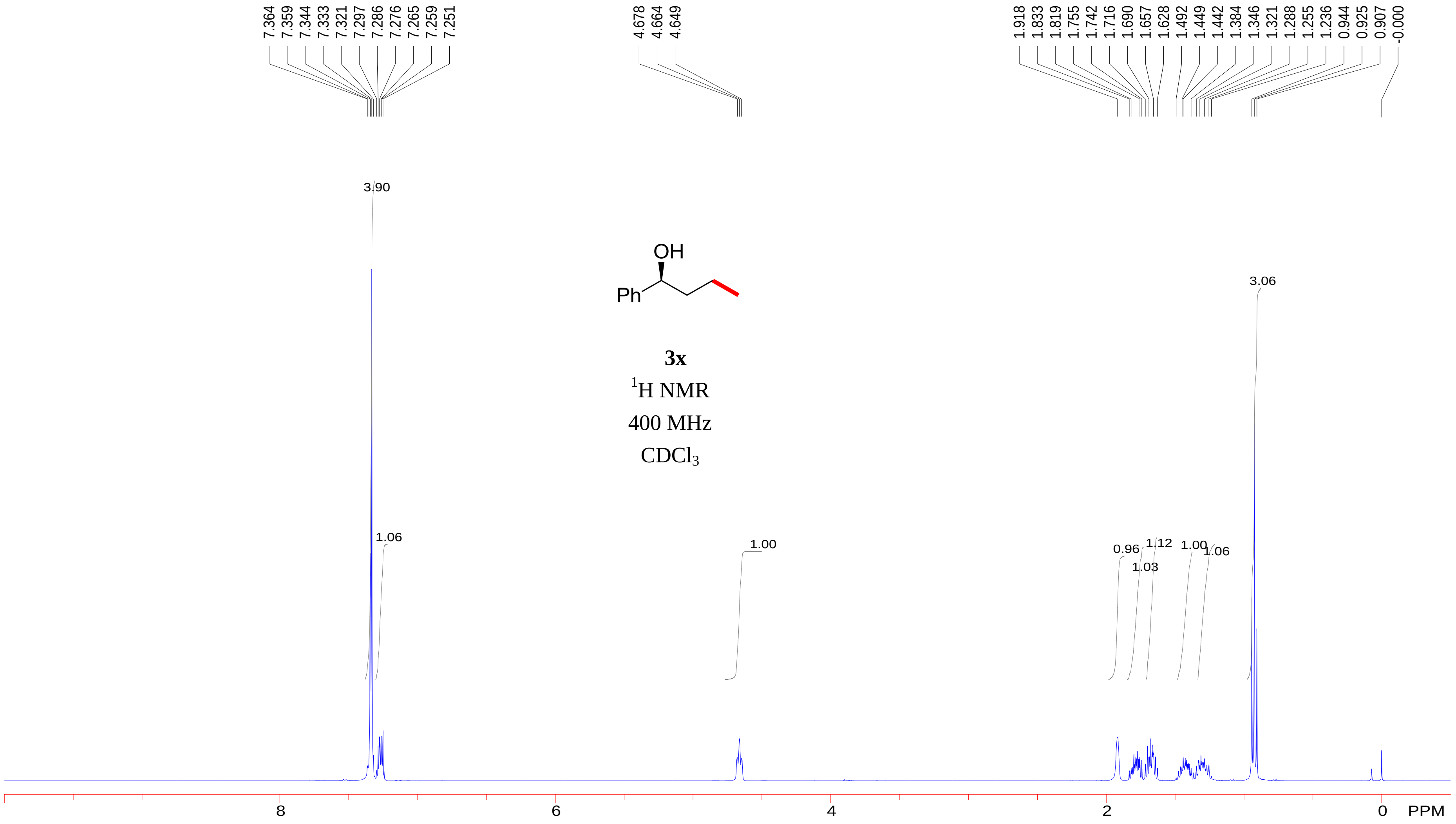

Supplementary Figure 174. ^{1}H NMR spectrum for **3x**

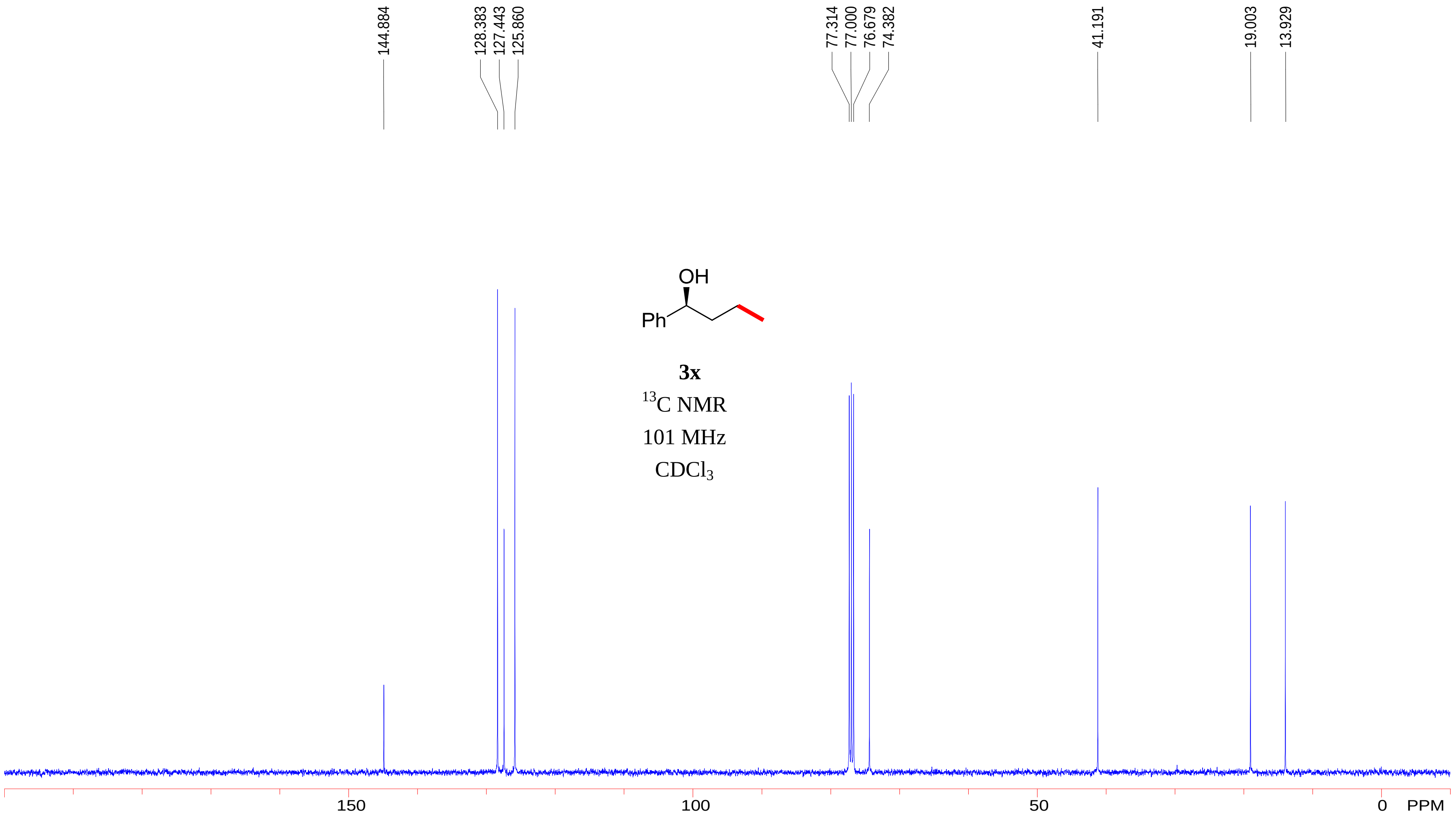

Supplementary Figure 175. ^{13}C NMR spectrum for **3x**

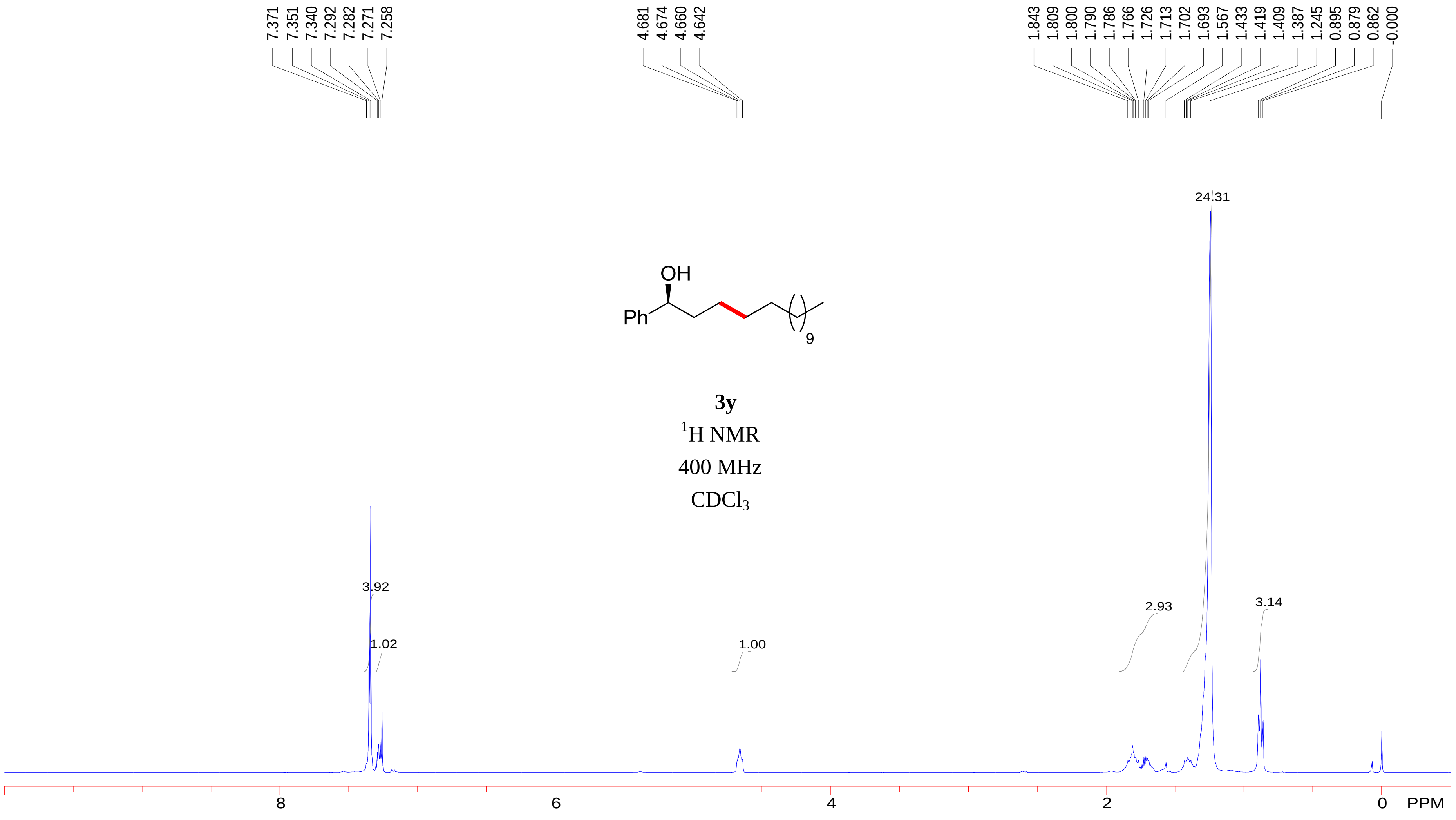

Supplementary Figure 176. ^{1}H NMR spectrum for **3y**

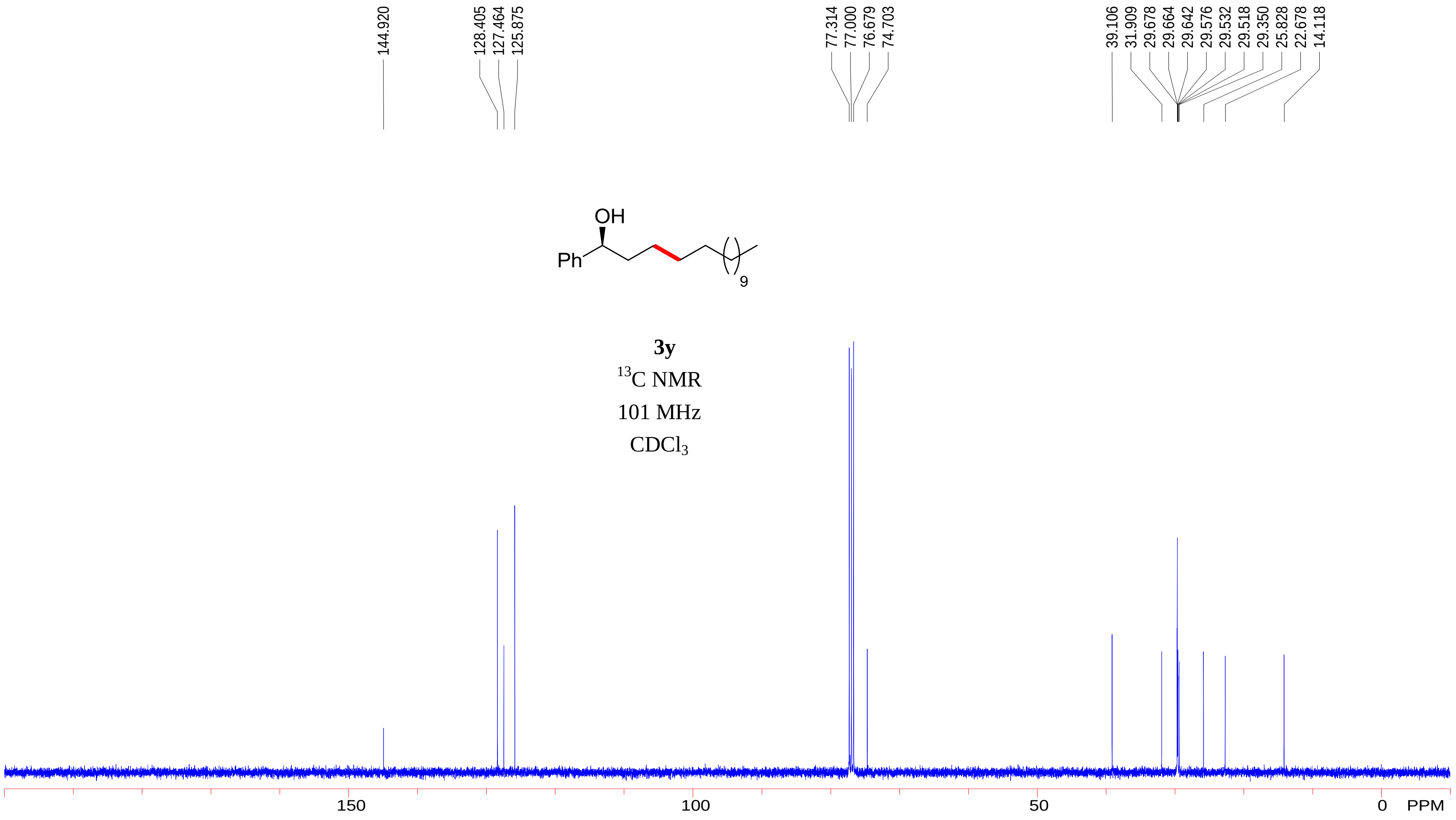

Supplementary Figure 177. ^{13}C NMR spectrum for **3y**

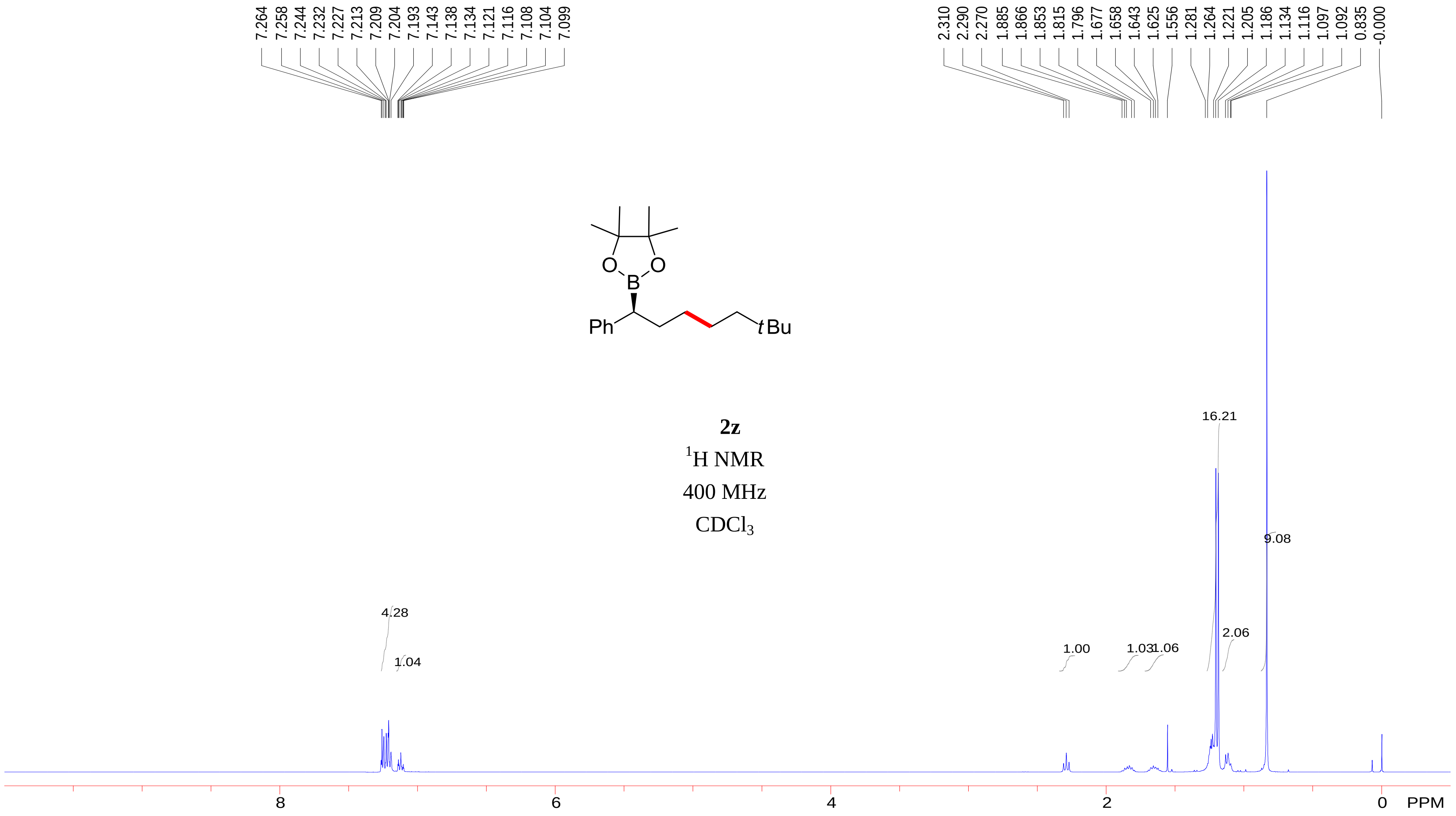

Supplementary Figure 178. ^{1}H NMR spectrum for **2z**

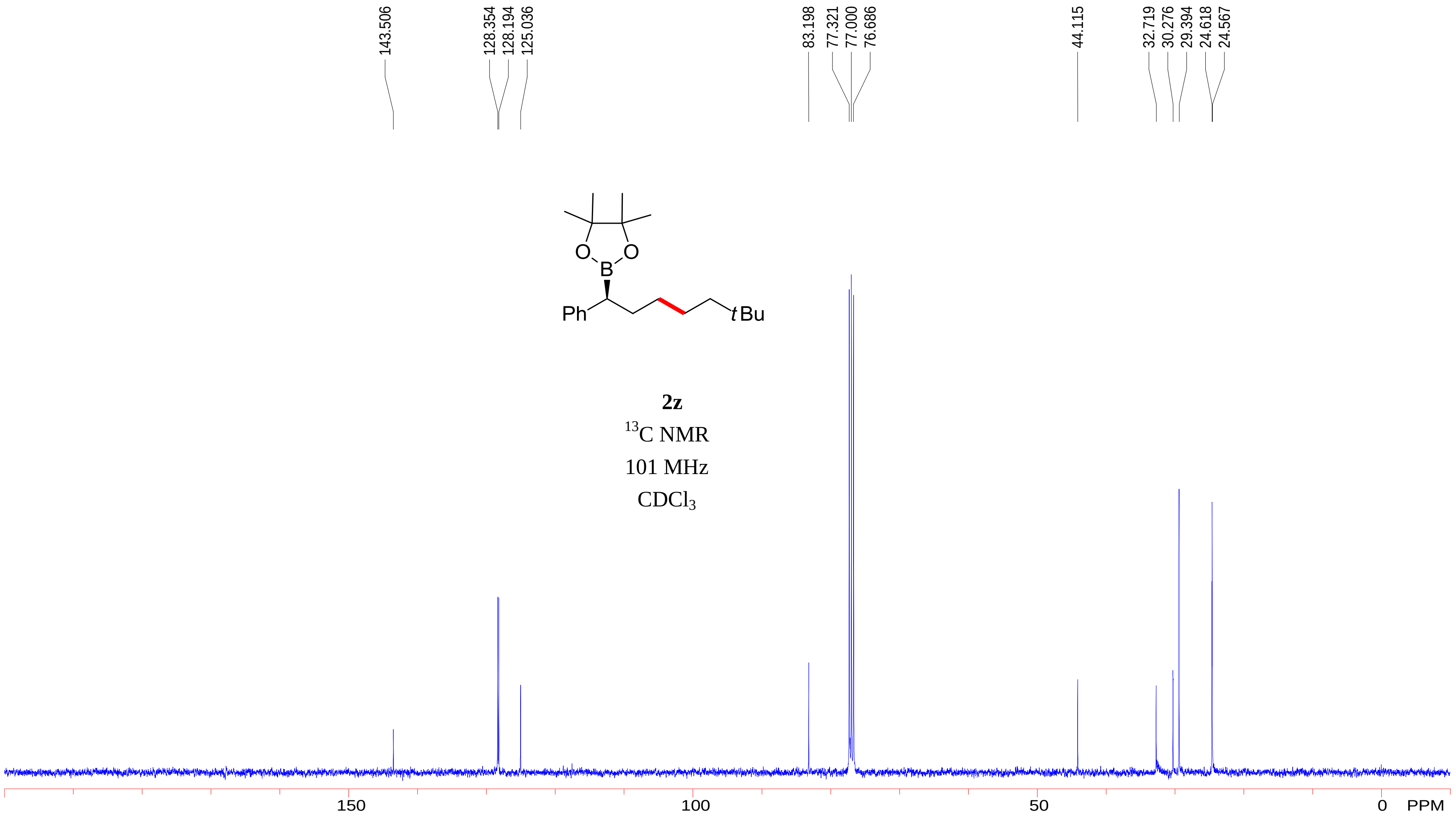

Supplementary Figure 179. ^{13}C NMR spectrum for **2z**

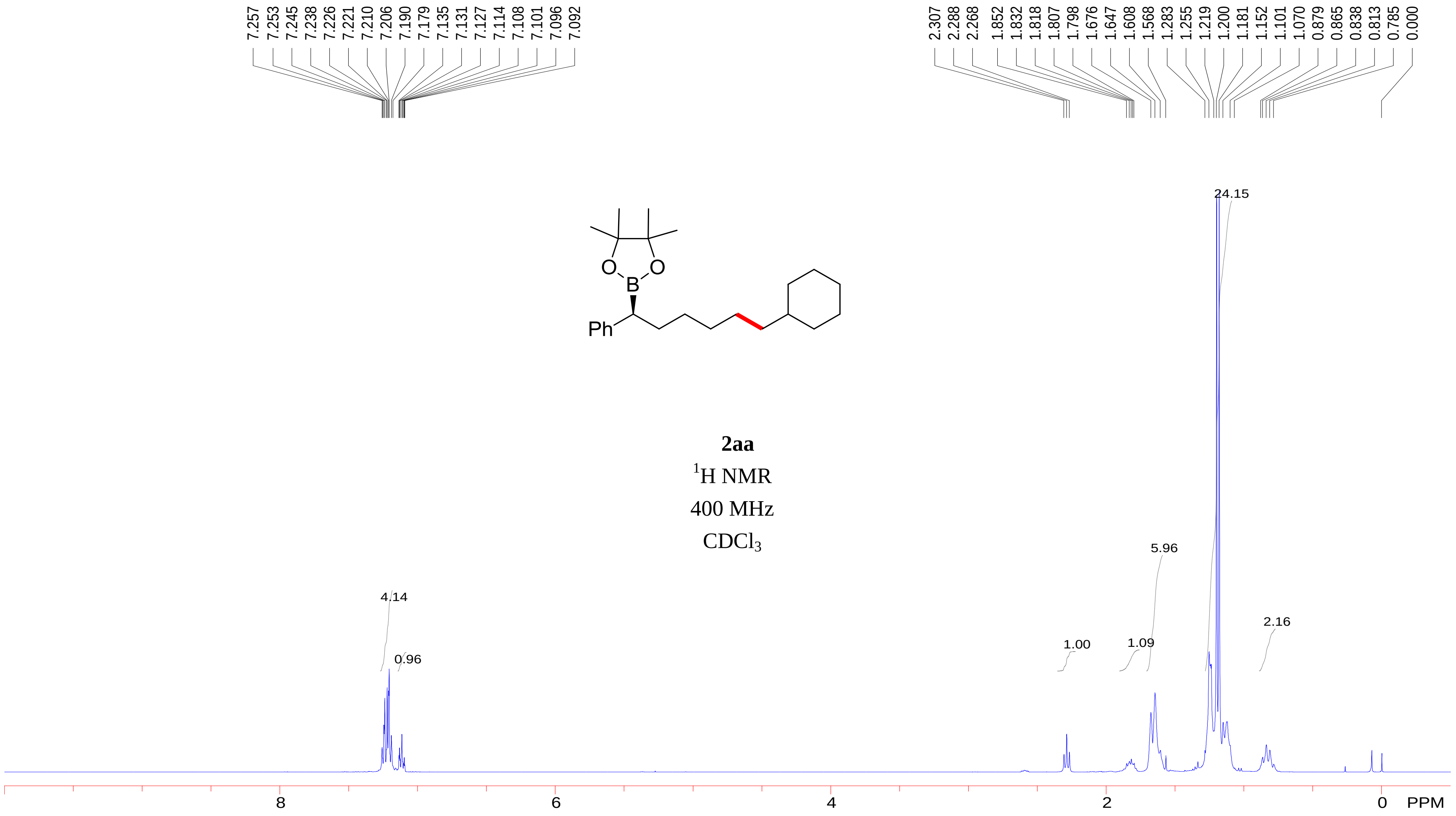

Supplementary Figure 180. ^{1}H NMR spectrum for **2aa**

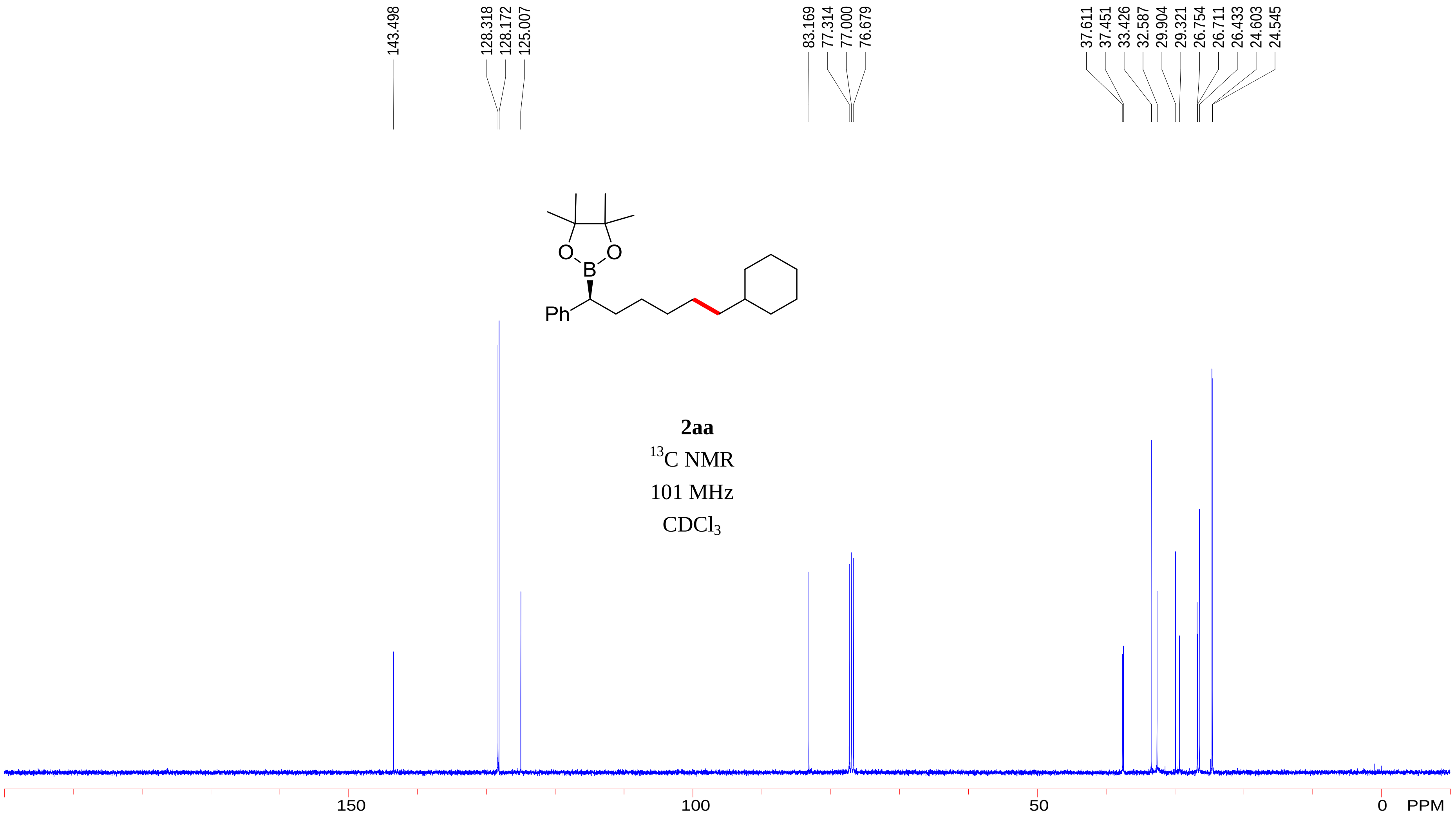

Supplementary Figure 181. ^{13}C NMR spectrum for **2aa**

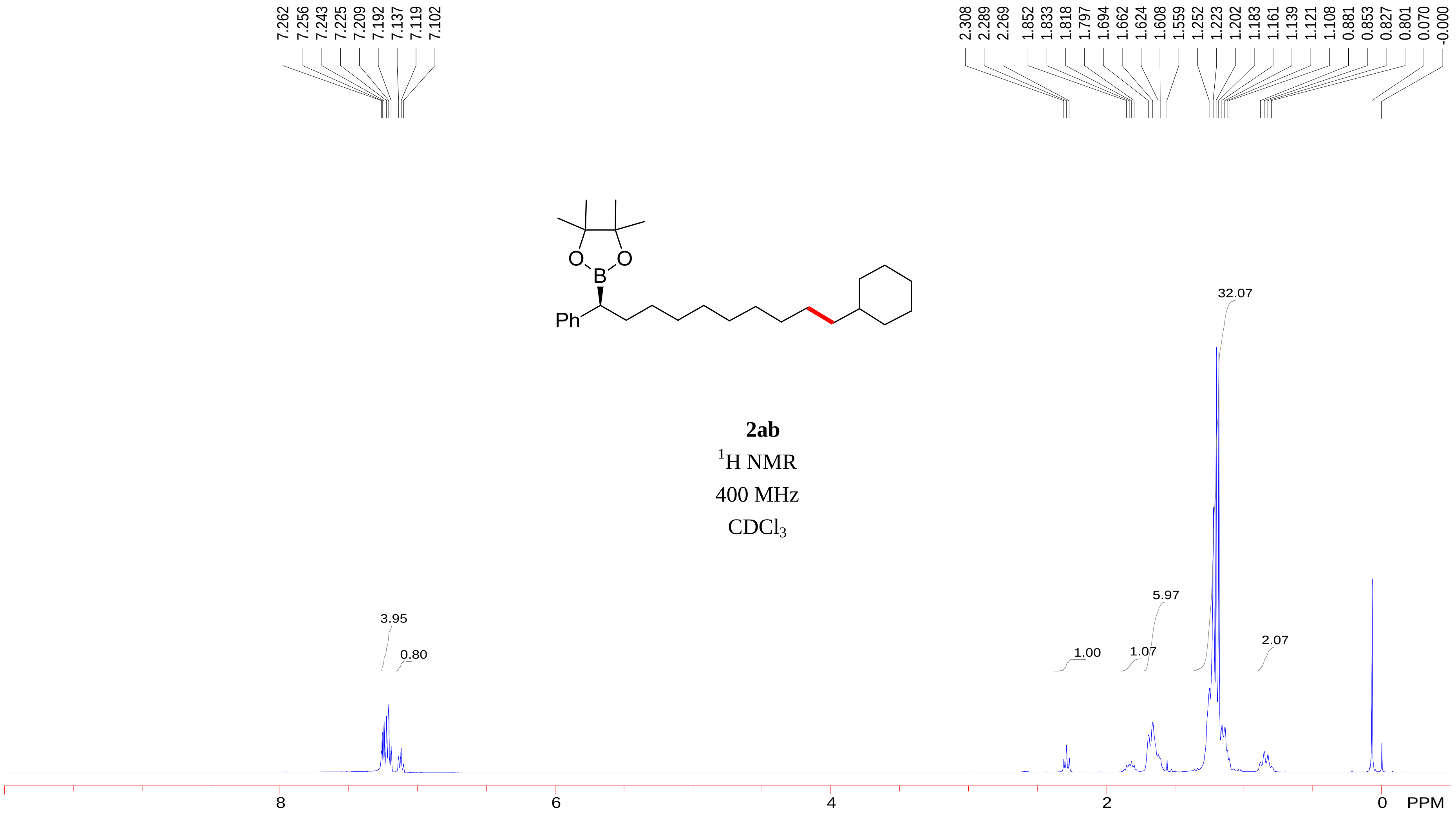

Supplementary Figure 182. ^{1}H NMR spectrum for **2ab**

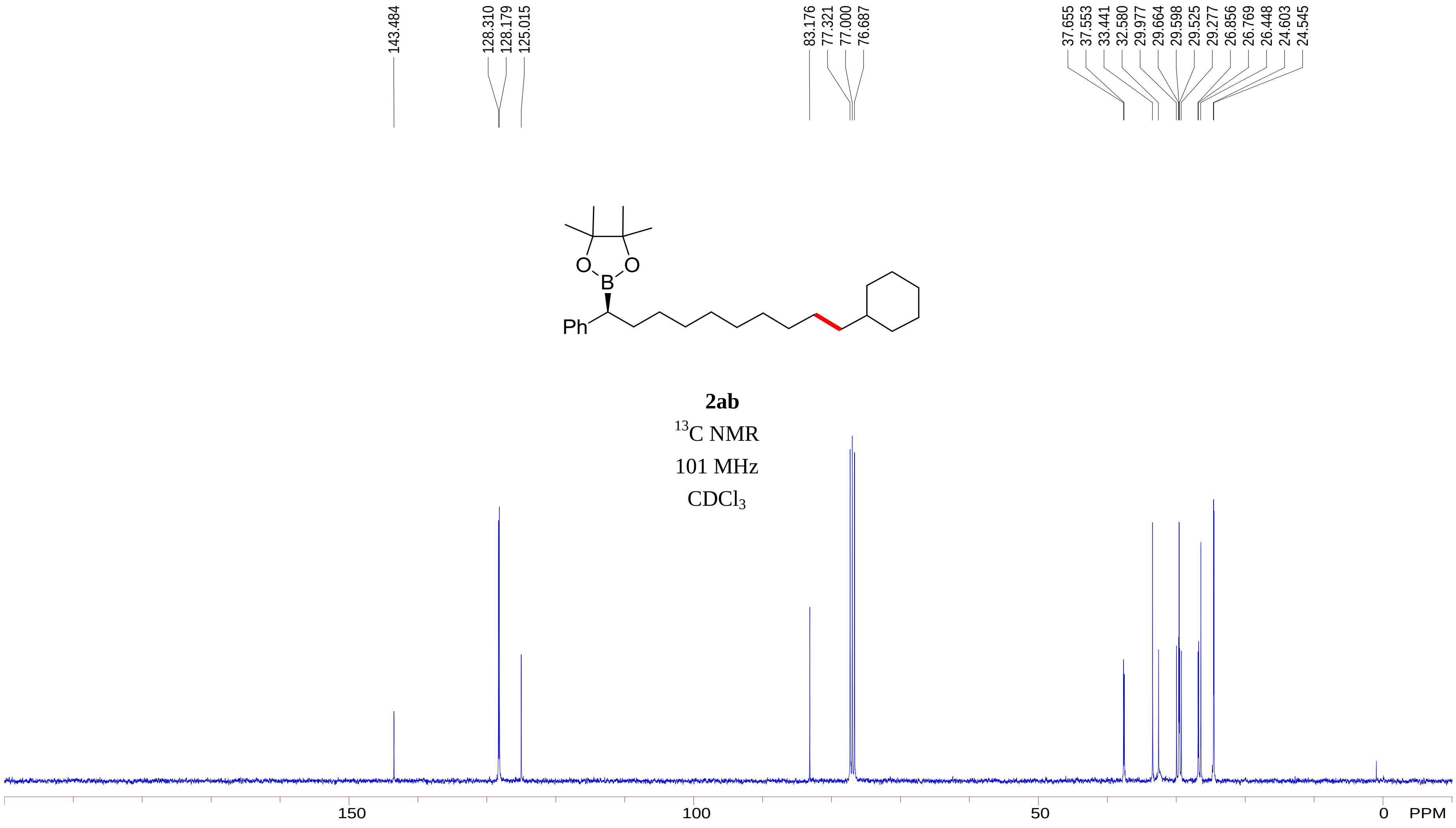

Supplementary Figure 183. ^{13}C NMR spectrum for **2ab**

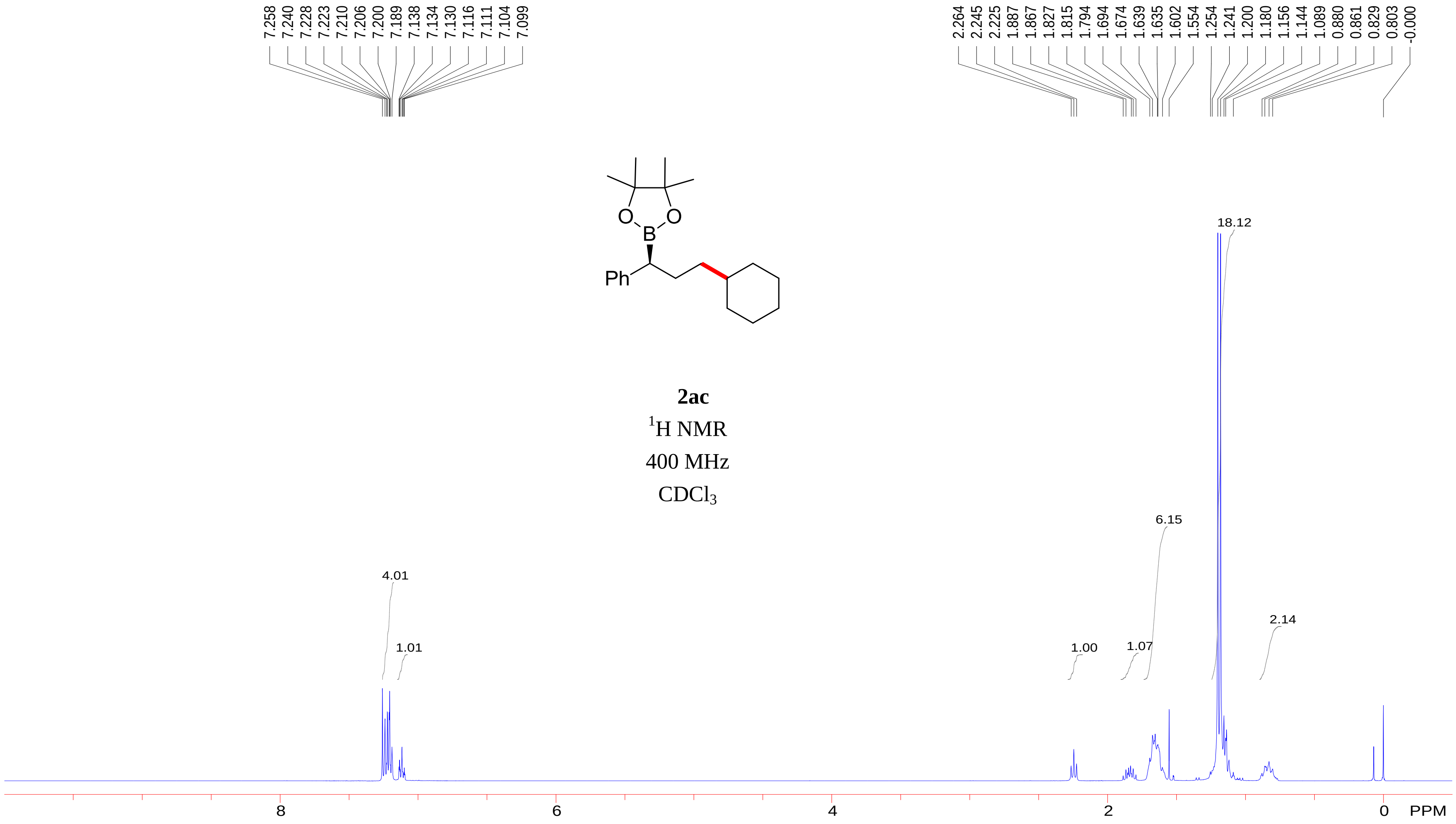

Supplementary Figure 184. ^{1}H NMR spectrum for **2ac**

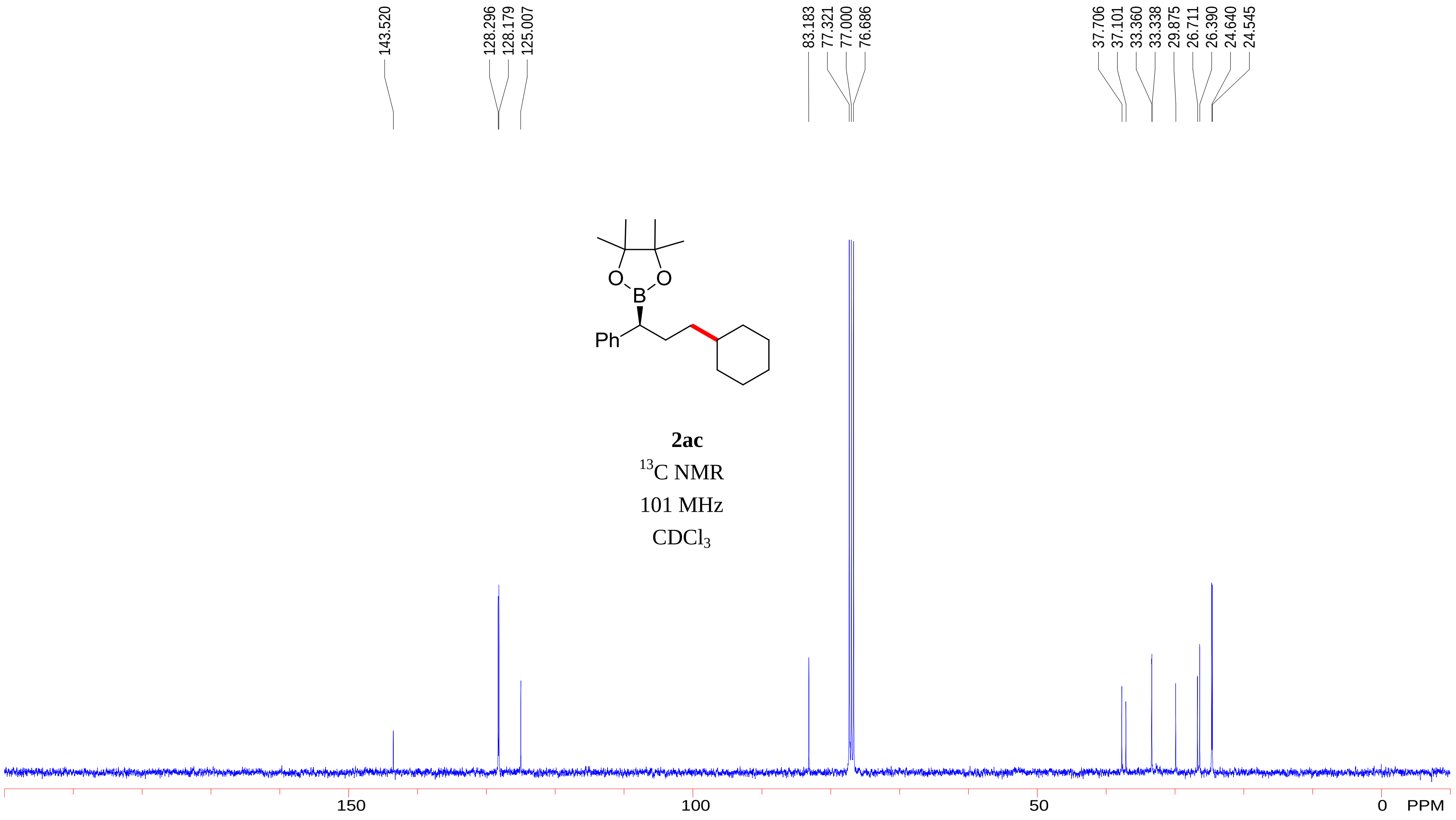

Supplementary Figure 185. ^{13}C NMR spectrum for **2ac**

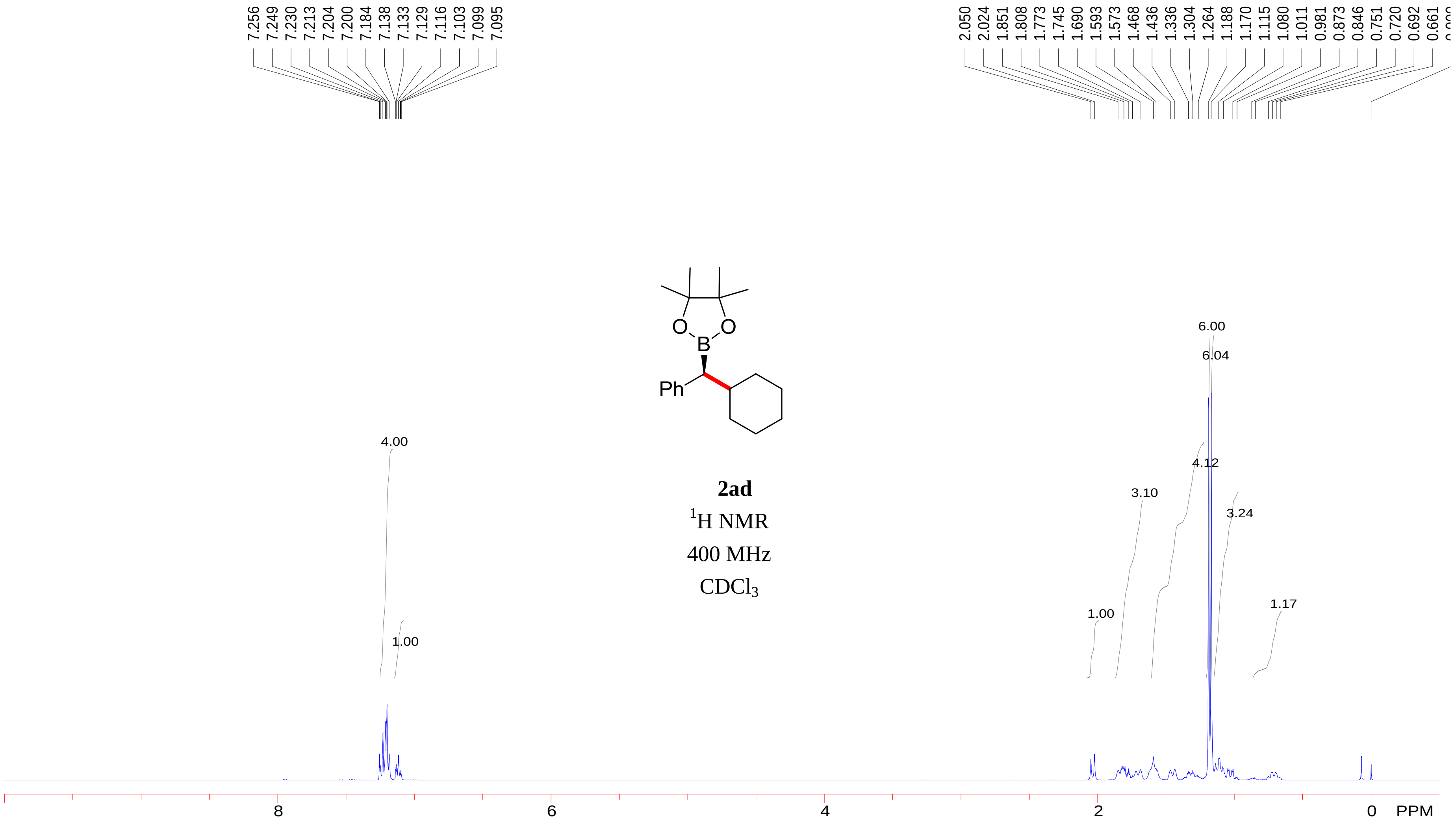

Supplementary Figure 186. ^{1}H NMR spectrum for **2ad**

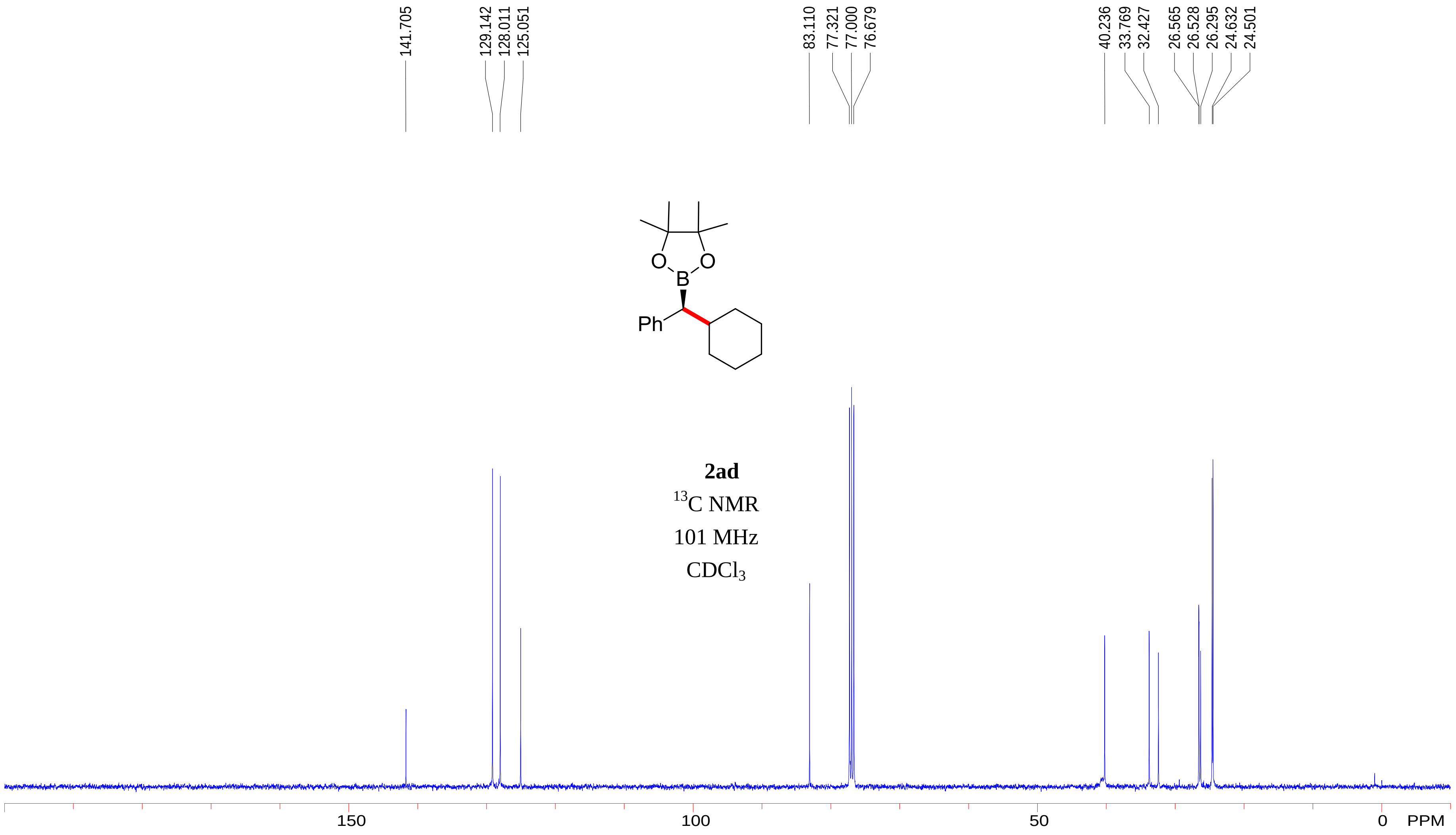

Supplementary Figure 187. ^{13}C NMR spectrum for **2ad**

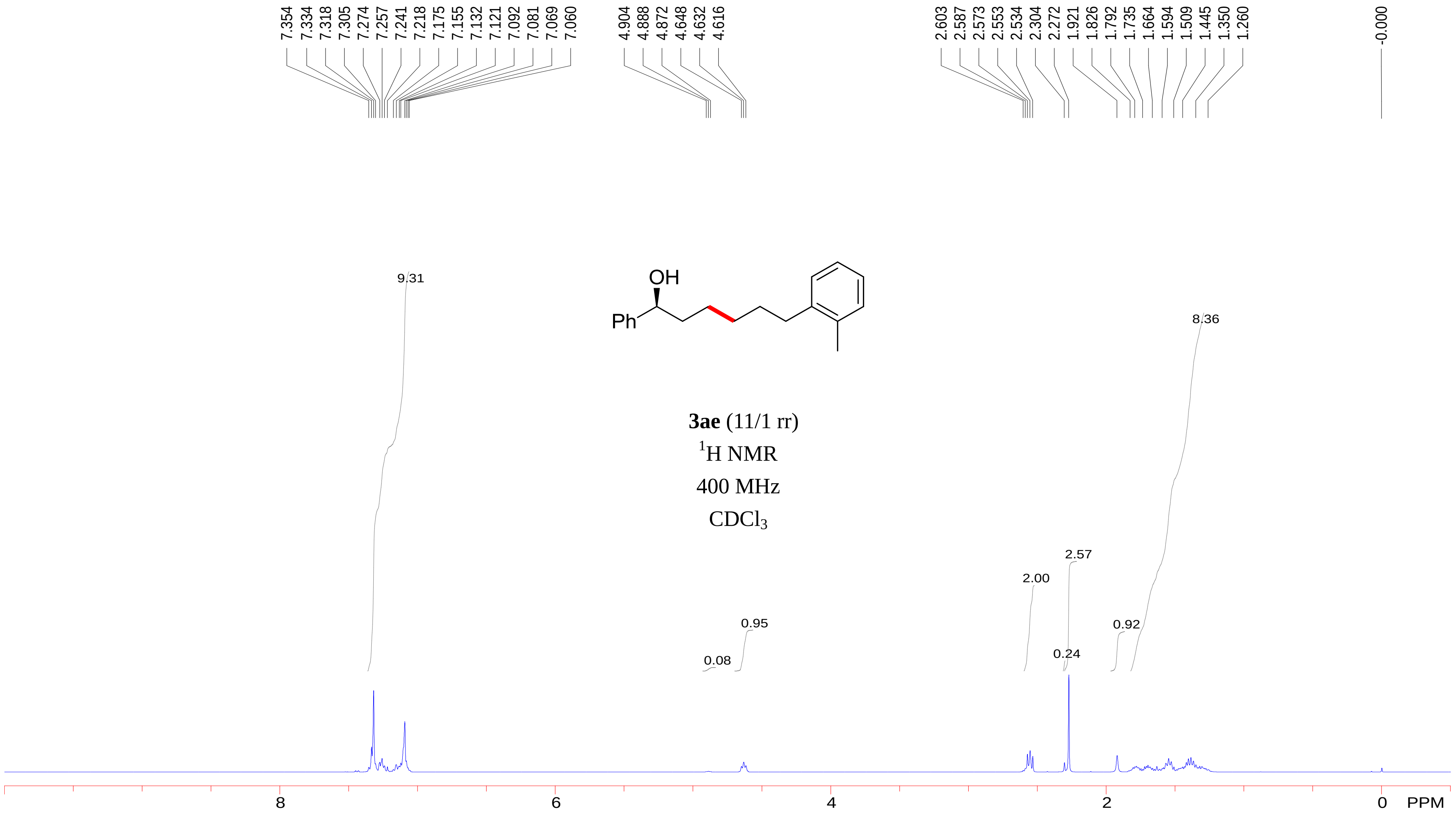

Supplementary Figure 188. ^{1}H NMR spectrum for **3ae**

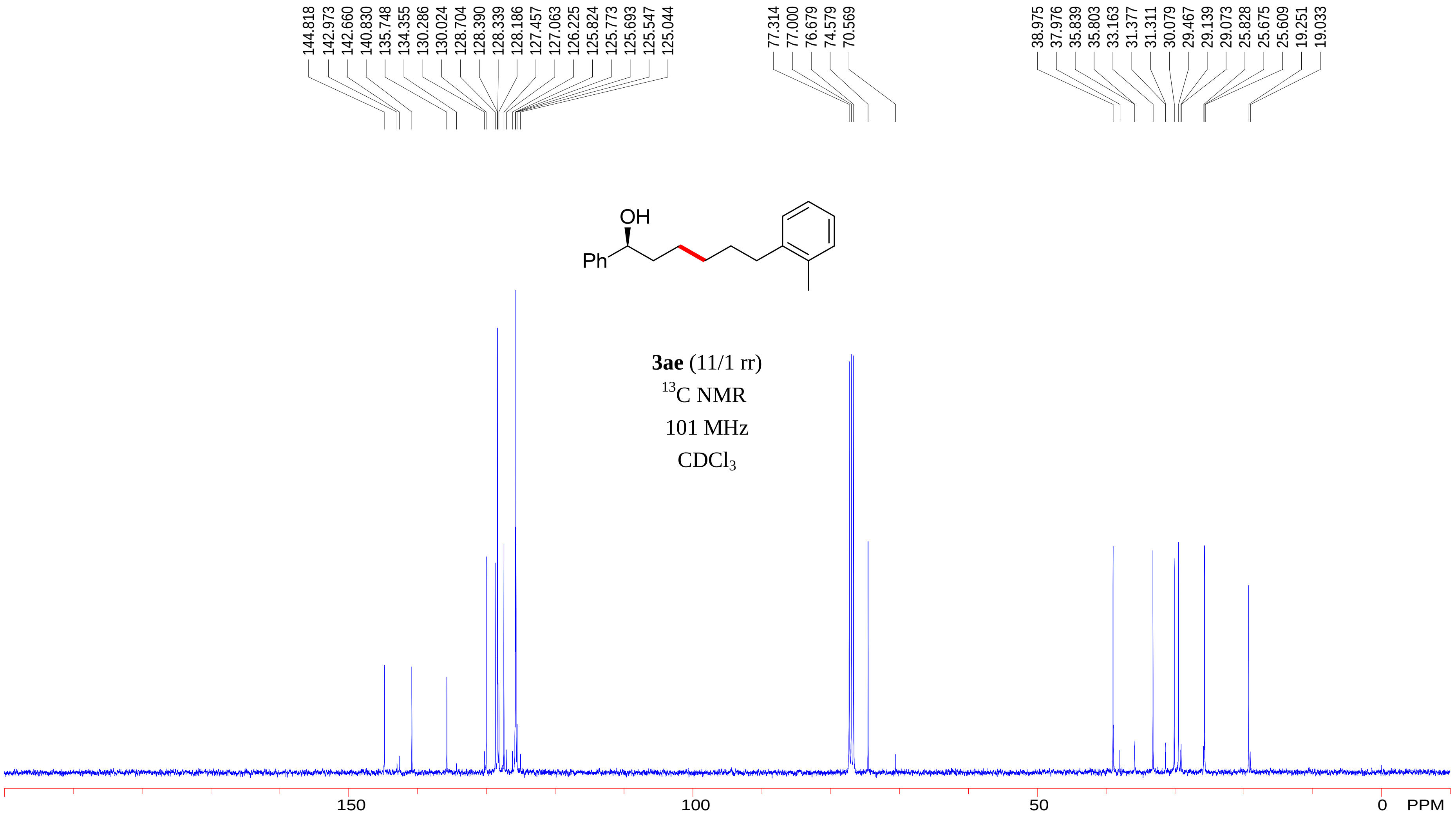

Supplementary Figure 189. ^{13}C NMR spectrum for **3ae**

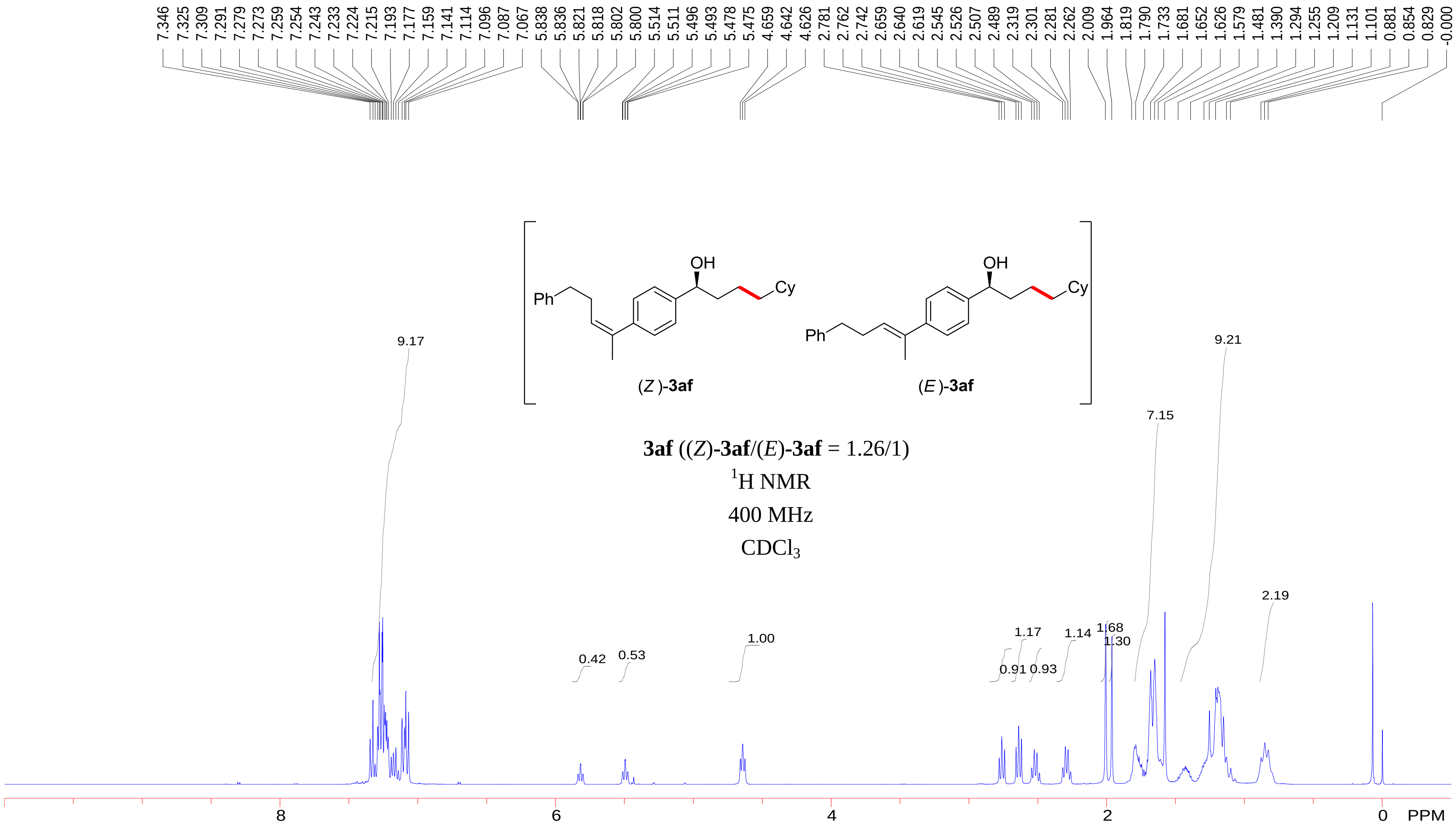

Supplementary Figure 190. ^{1}H NMR spectrum for **3af**

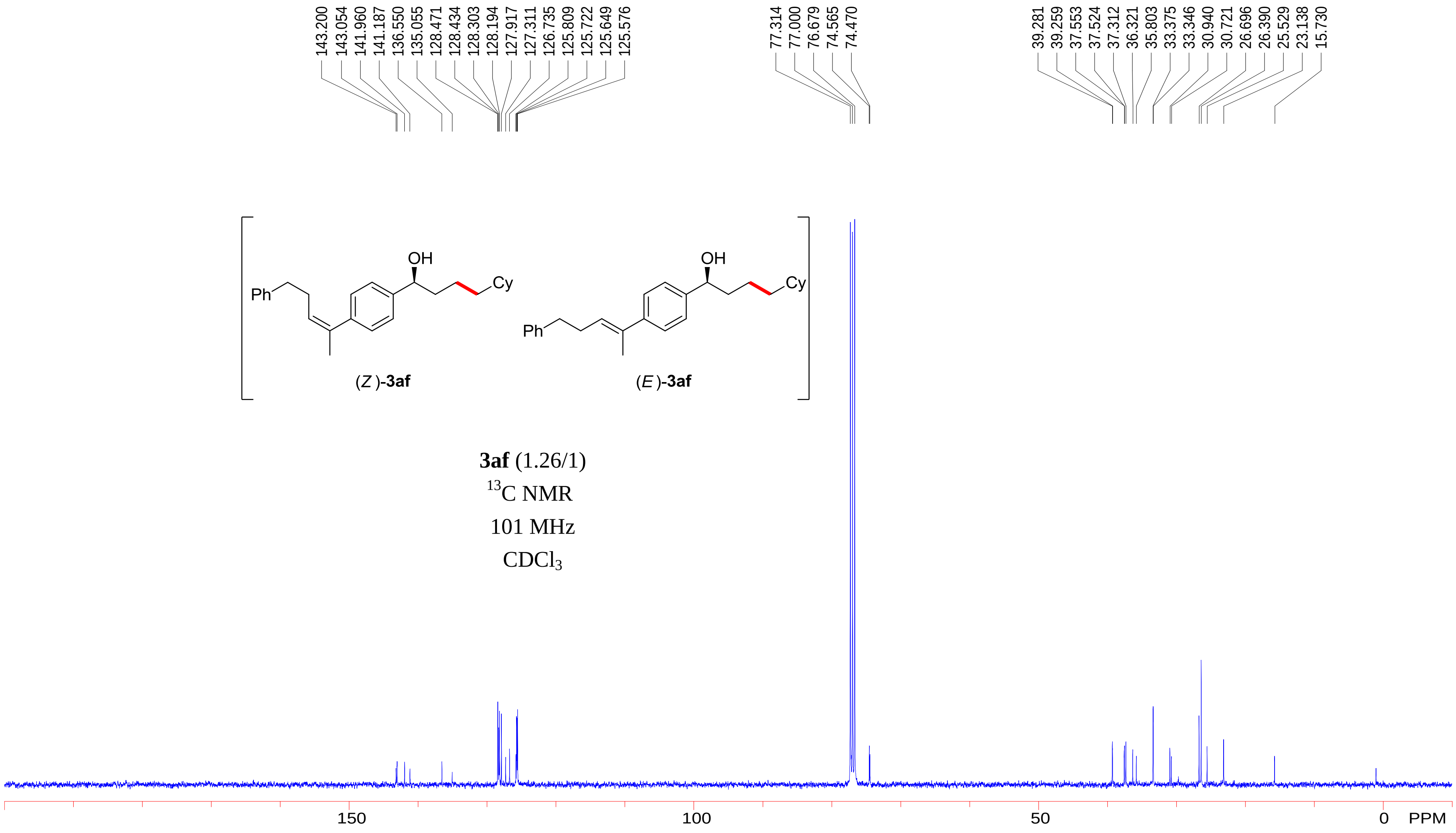

Supplementary Figure 191. ^{13}C NMR spectrum for **3af**

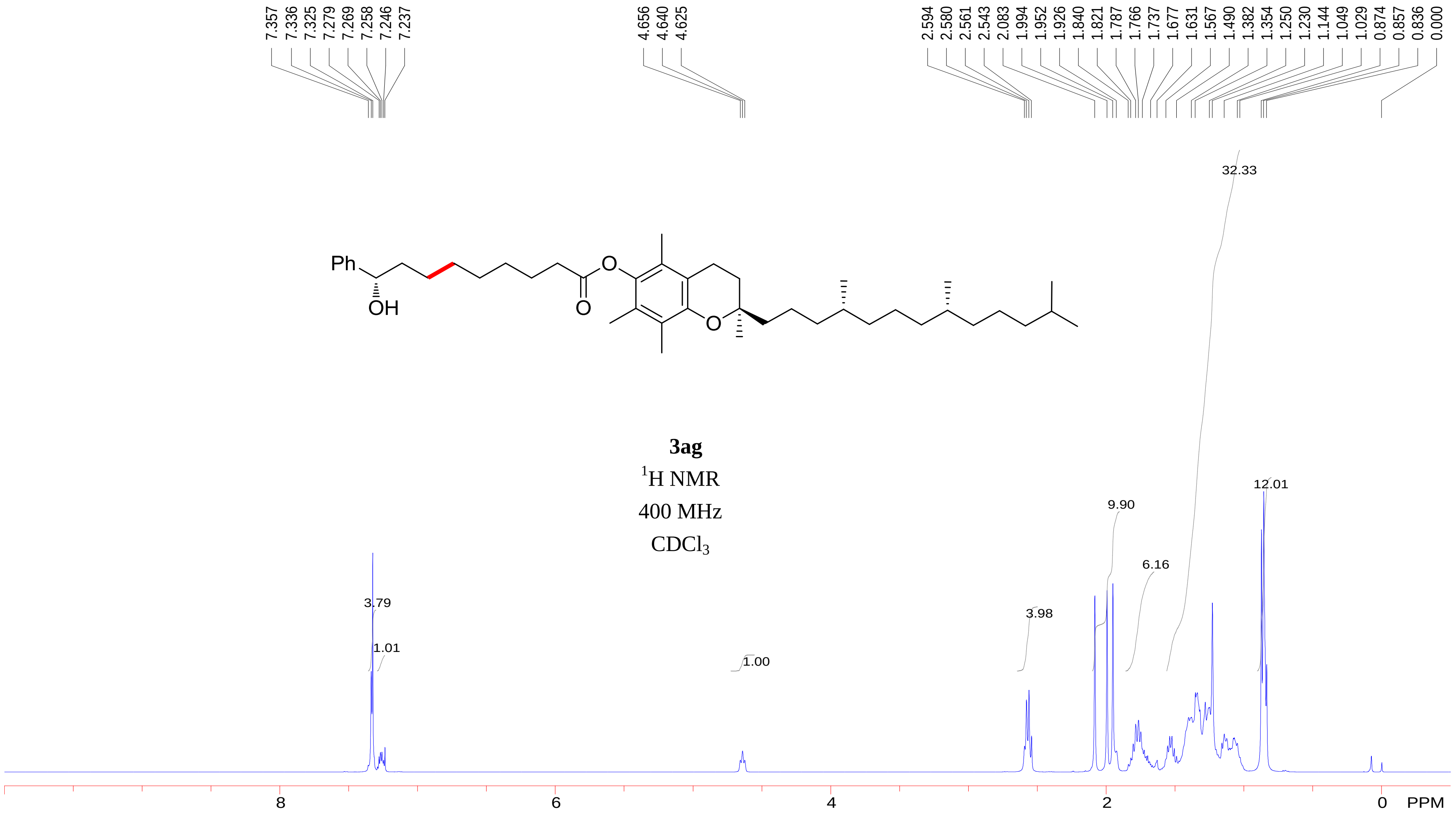

Supplementary Figure 192. ^{1}H NMR spectrum for **3ag**

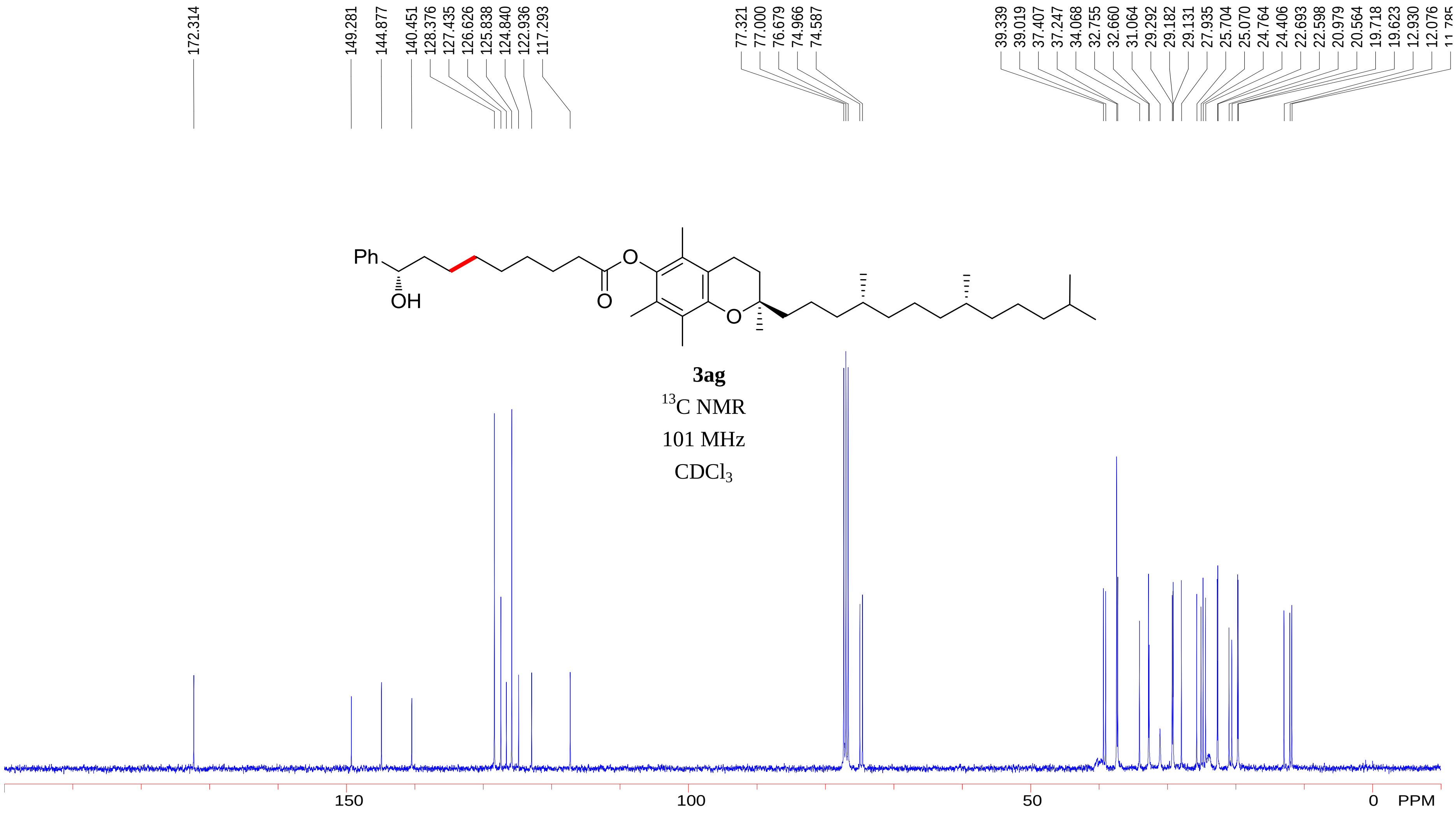

Supplementary Figure 193. ^{13}C NMR spectrum for **3ag**

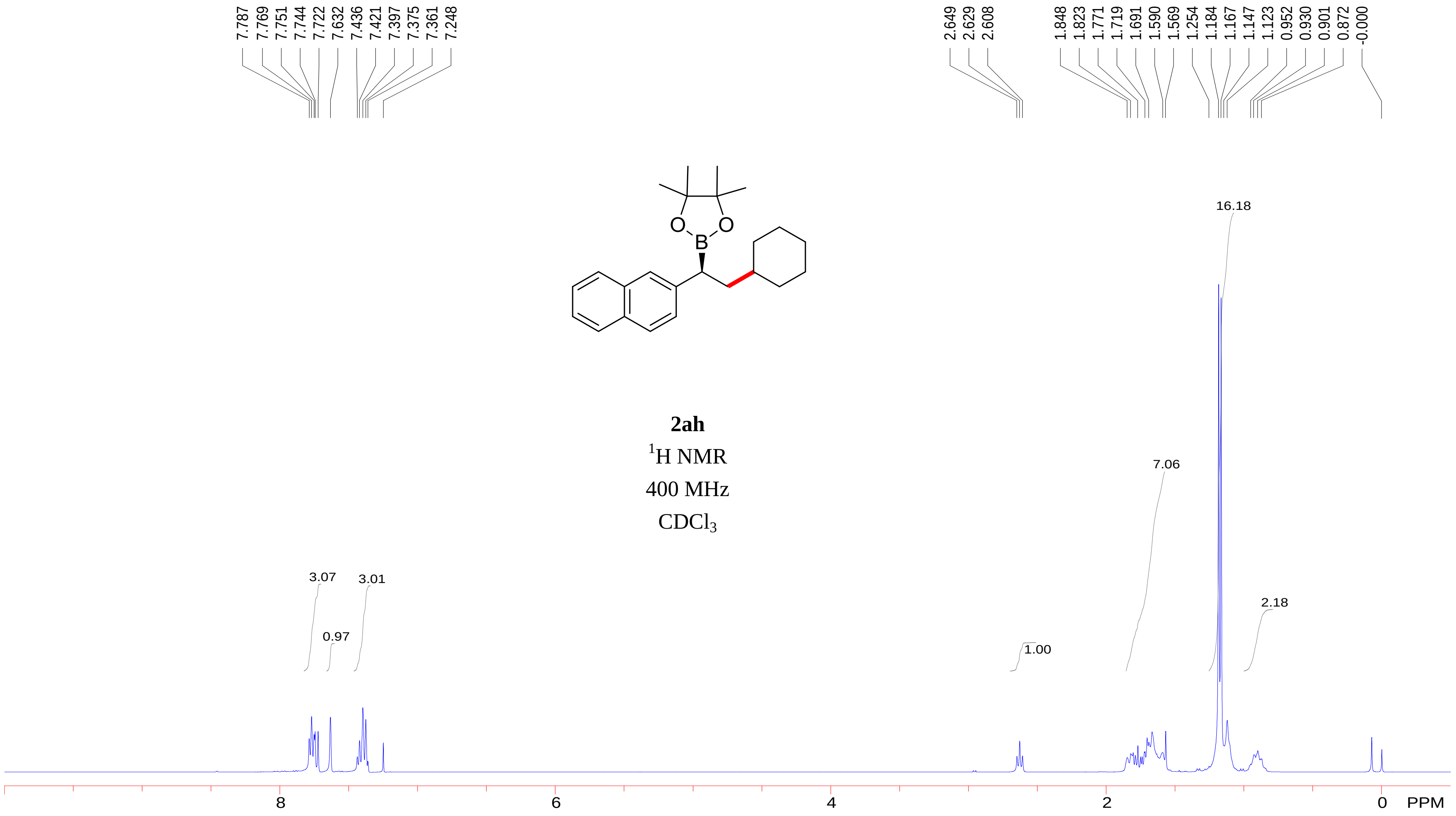

Supplementary Figure 194. ^{1}H NMR spectrum for **2ah**

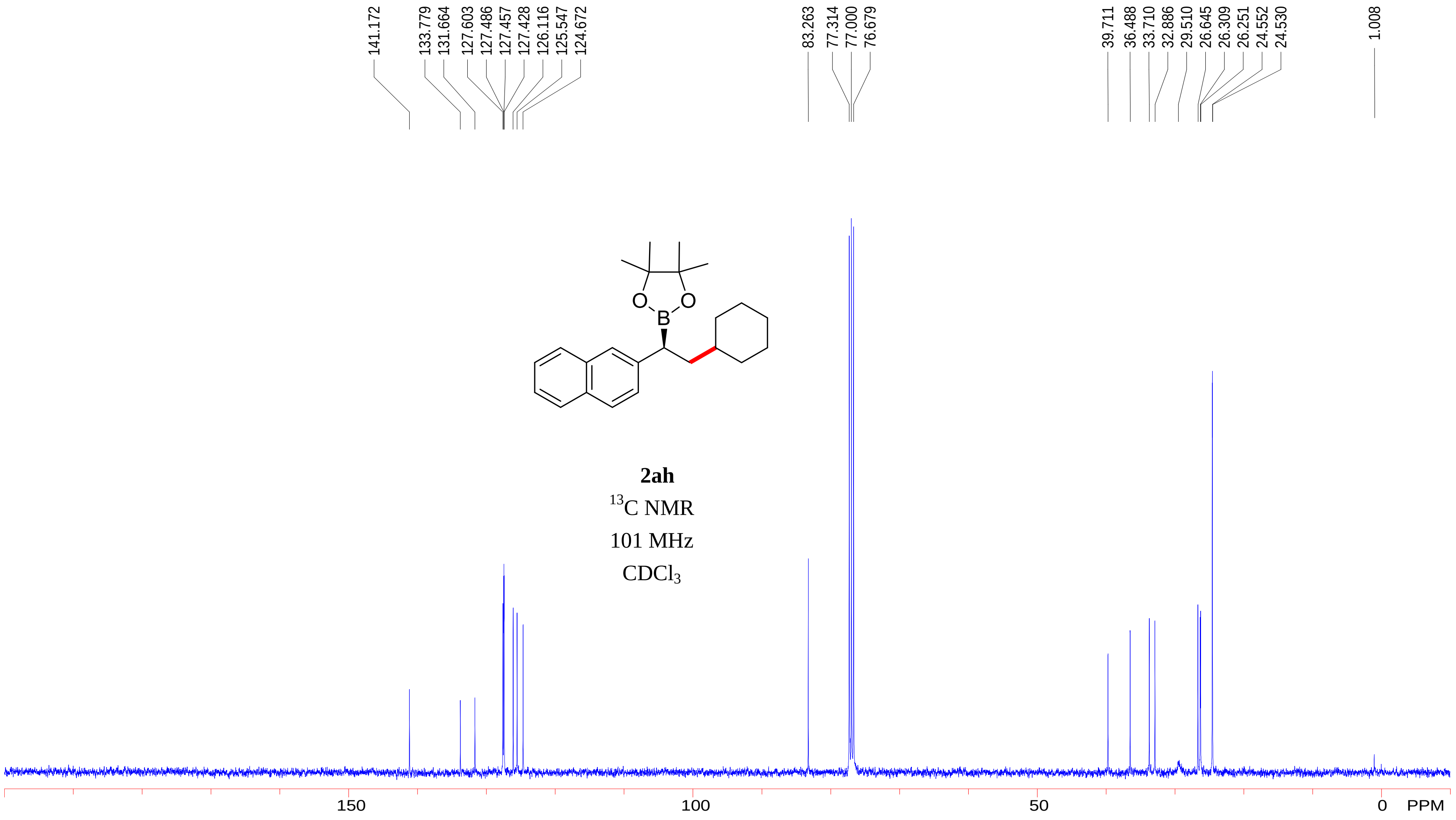

Supplementary Figure 195. ^{13}C NMR spectrum for **2ah**

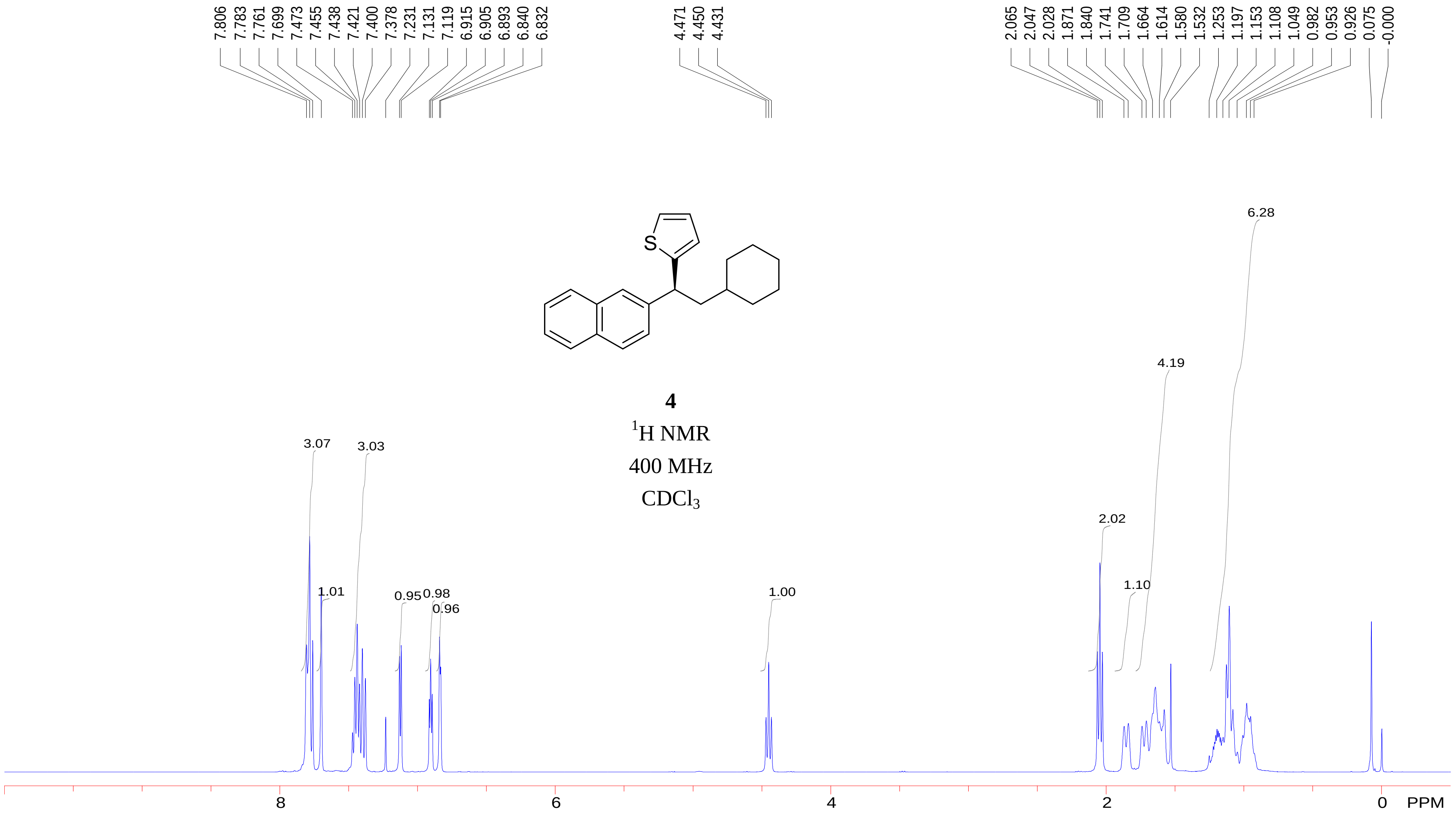

Supplementary Figure 196. ^{1}H NMR spectrum for **4**

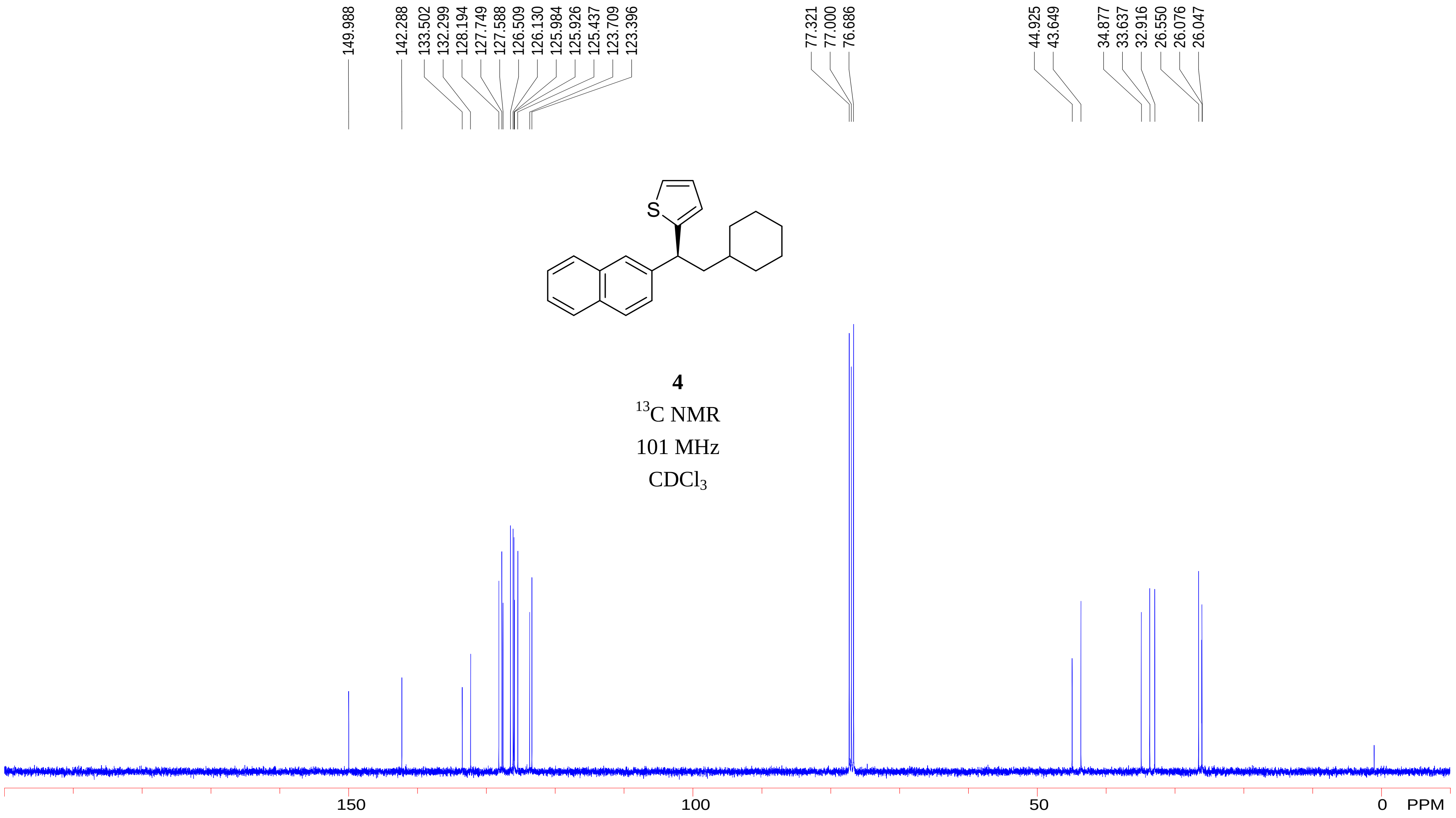

Supplementary Figure 197. ^{13}C NMR spectrum for **4**

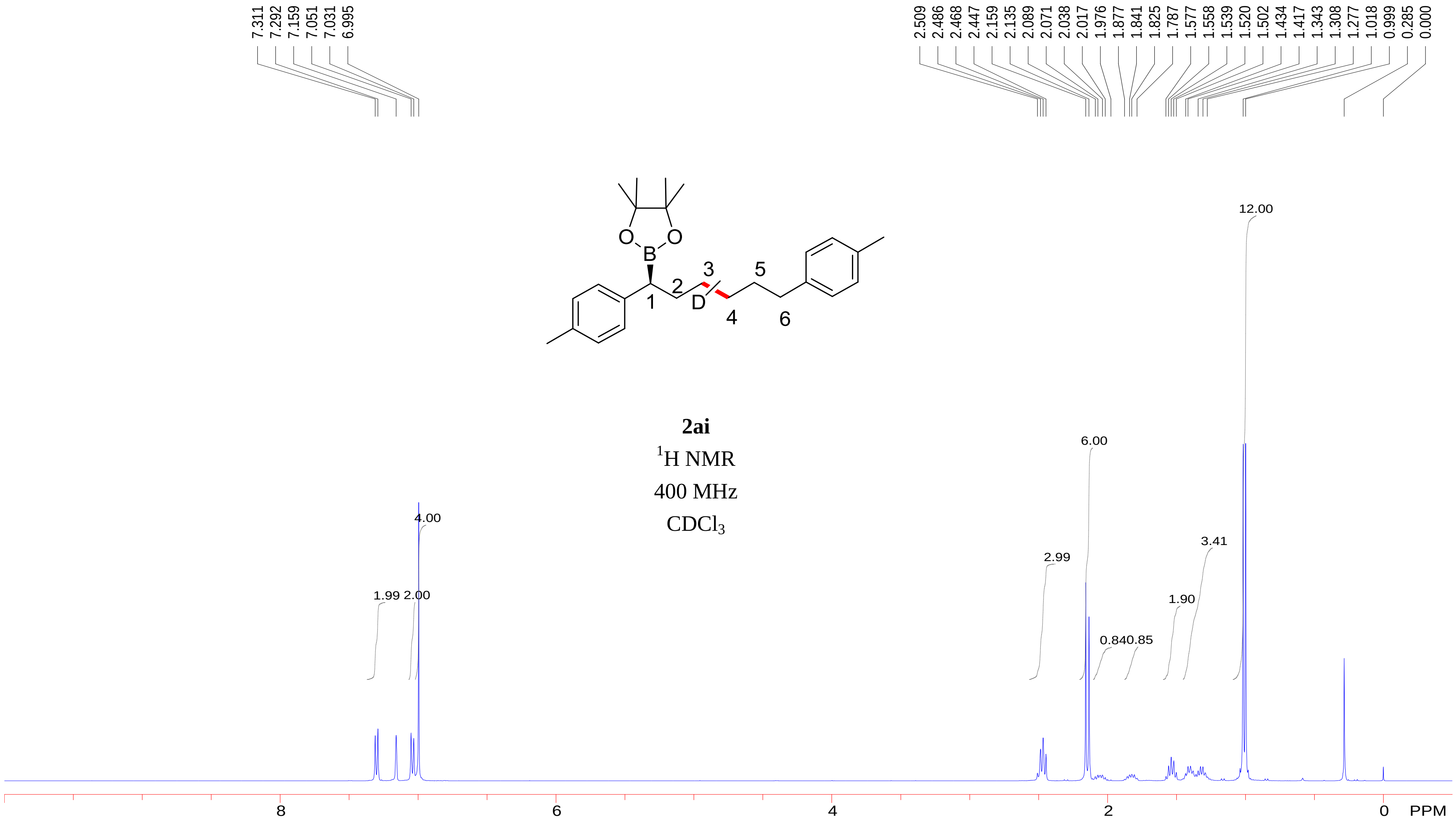

Supplementary Figure 198. ^{1}H NMR spectrum for **2ai**

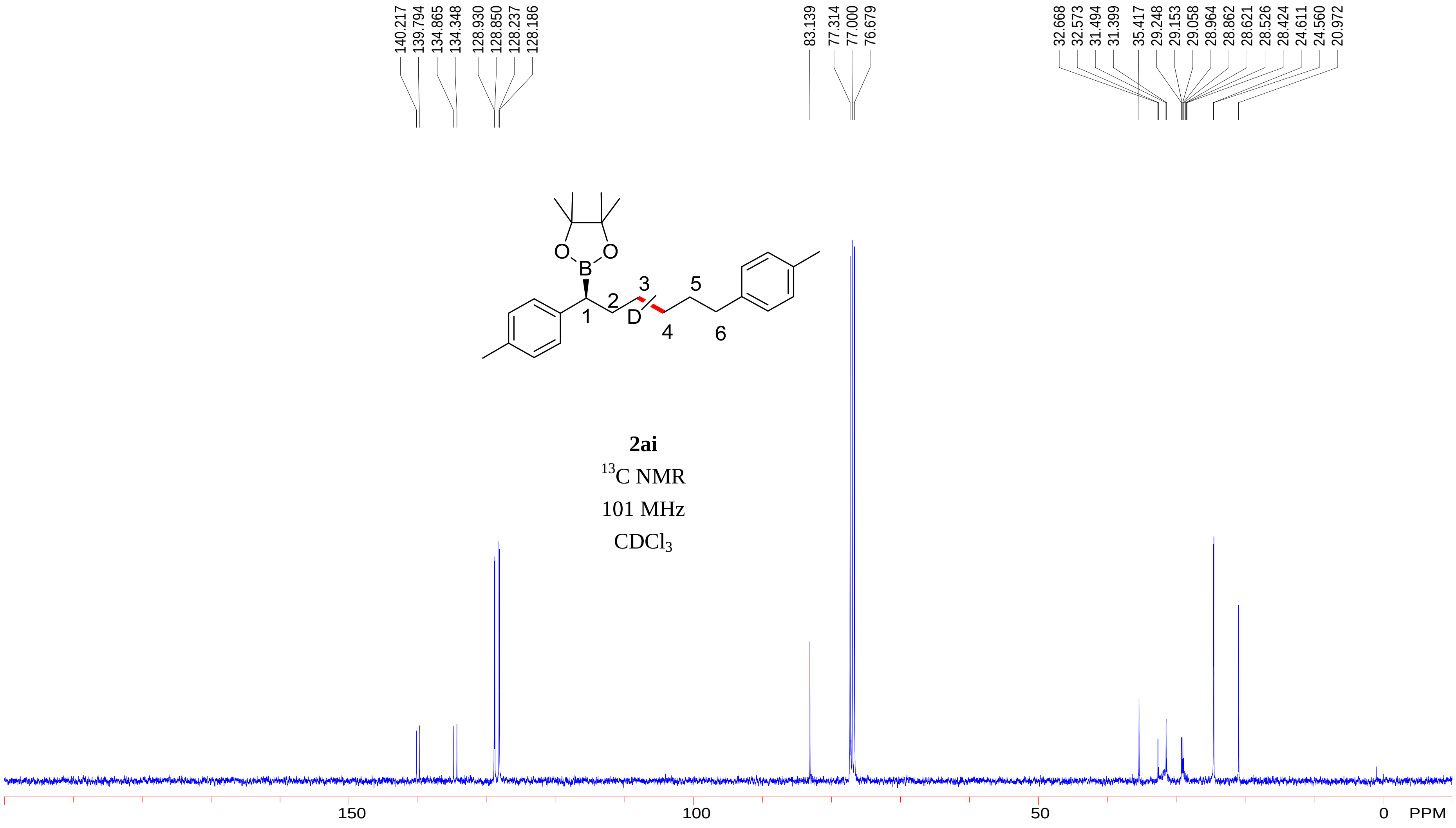

Supplementary Figure 199. ^{13}C NMR spectrum for **2ai**

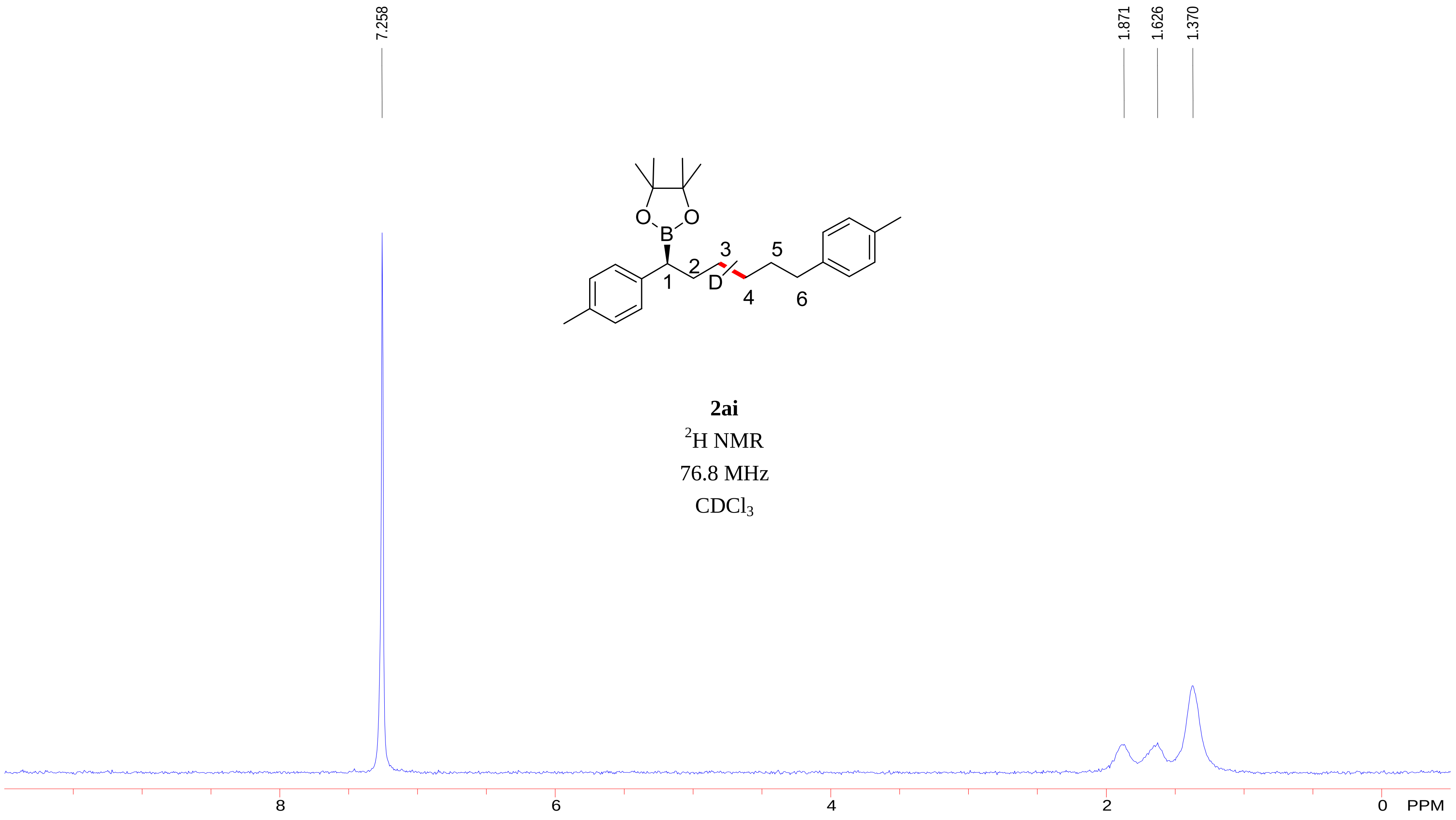

Supplementary Figure 200. ^{2}H NMR spectrum for **2ai**

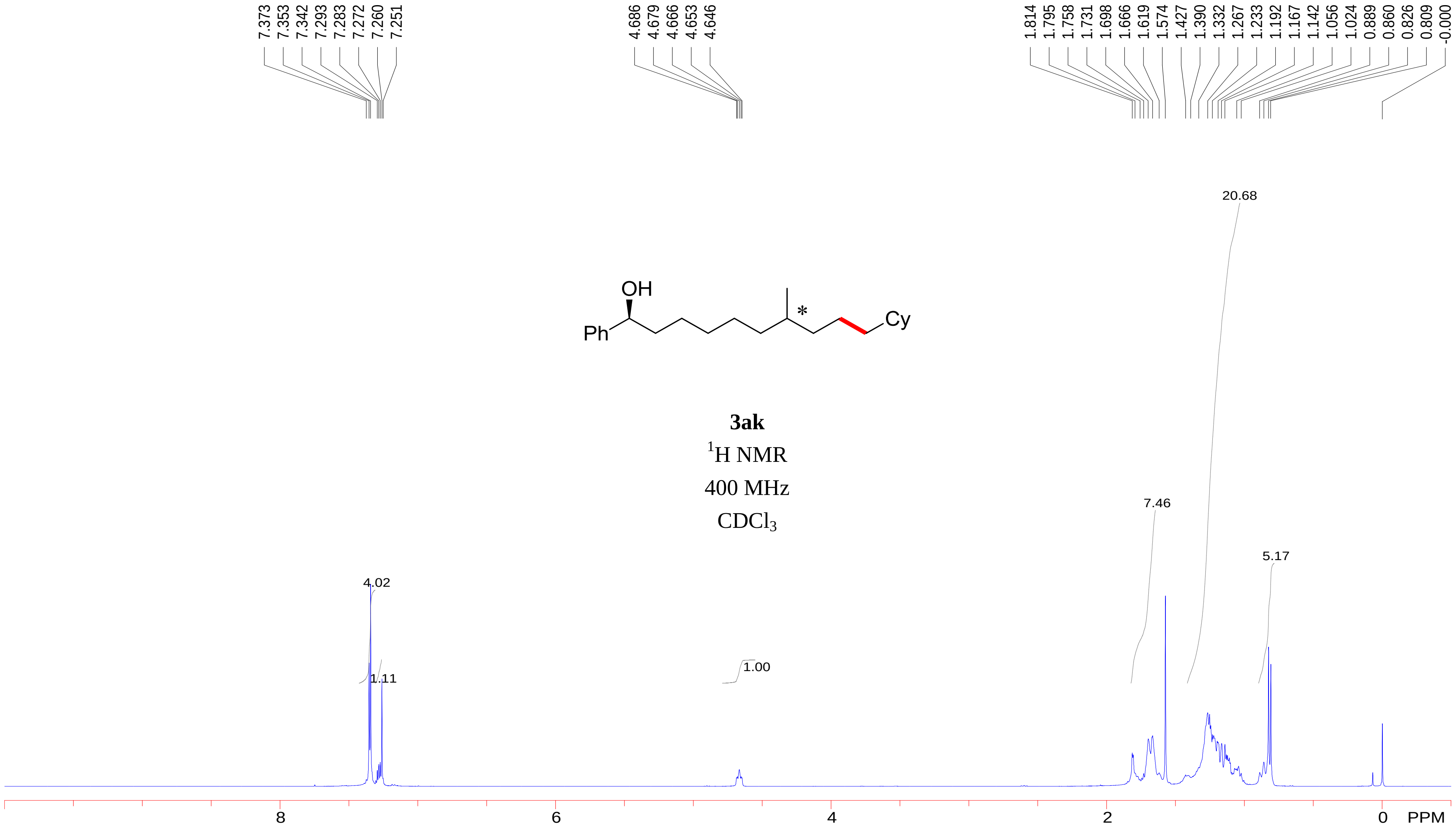

Supplementary Figure 201. ^{1}H NMR spectrum for **3ak**

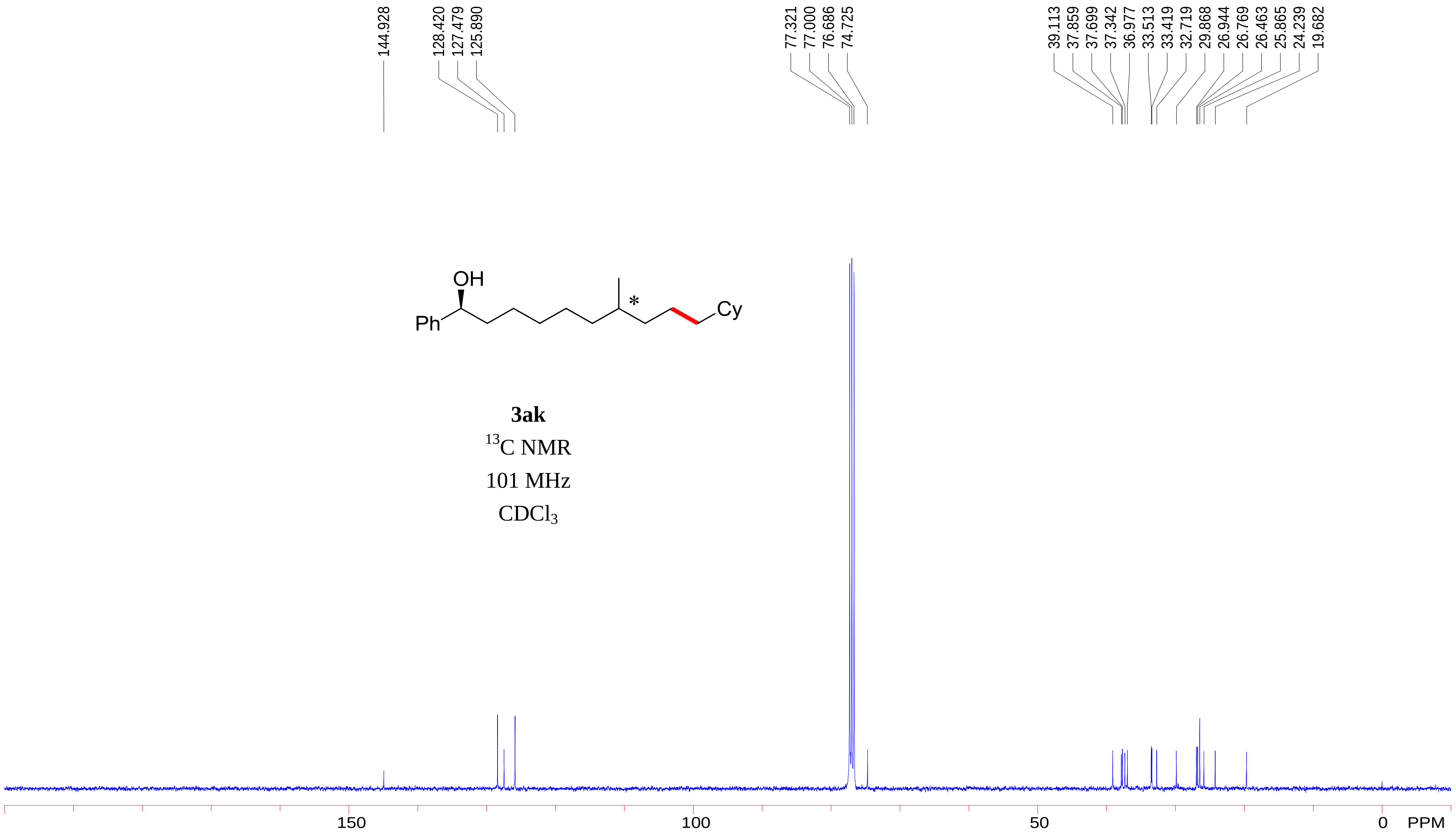

Supplementary Figure 202. ^{13}C NMR spectrum for **3ak**

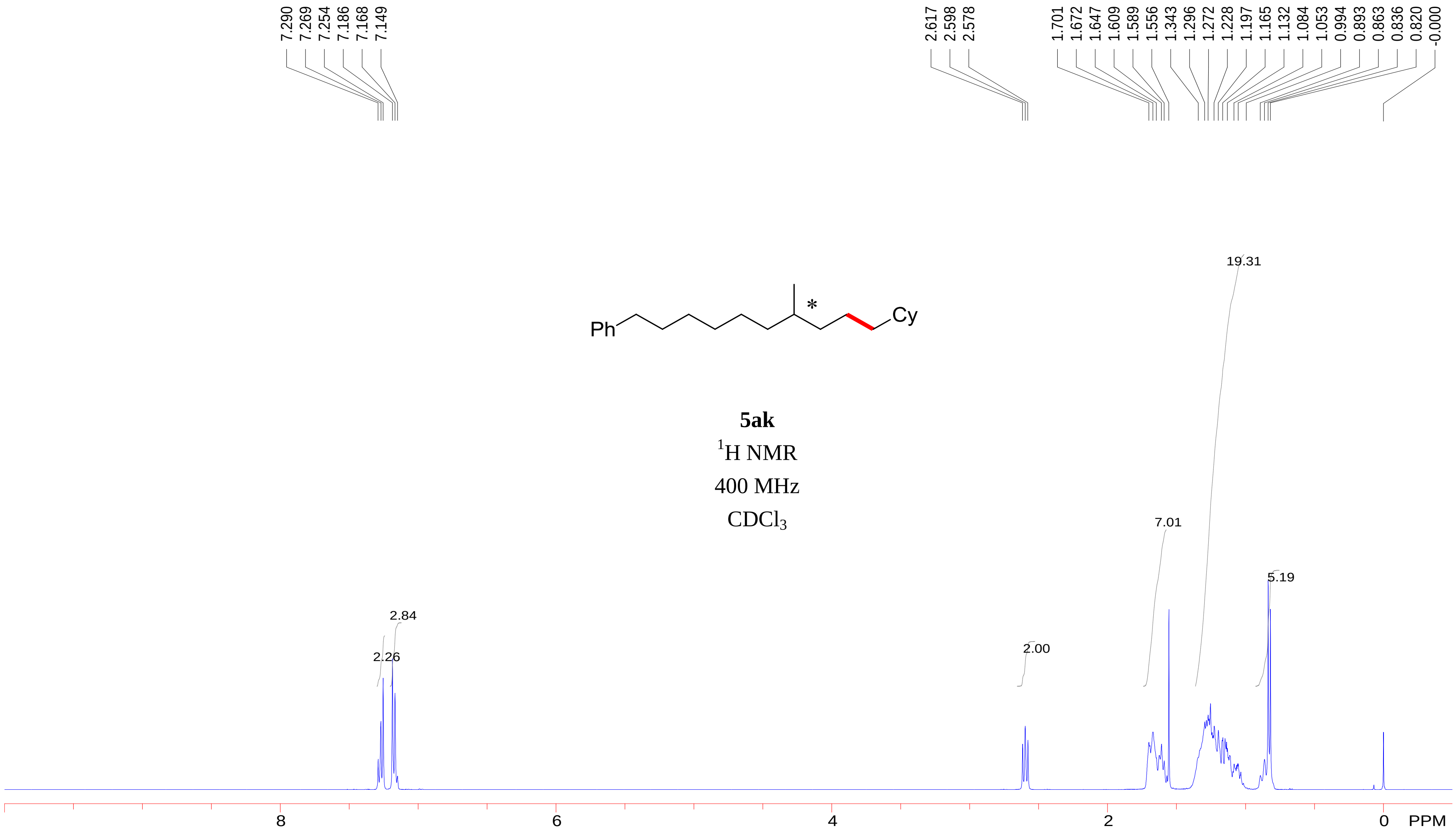

Supplementary Figure 203. ^{1}H NMR spectrum for **5ak**

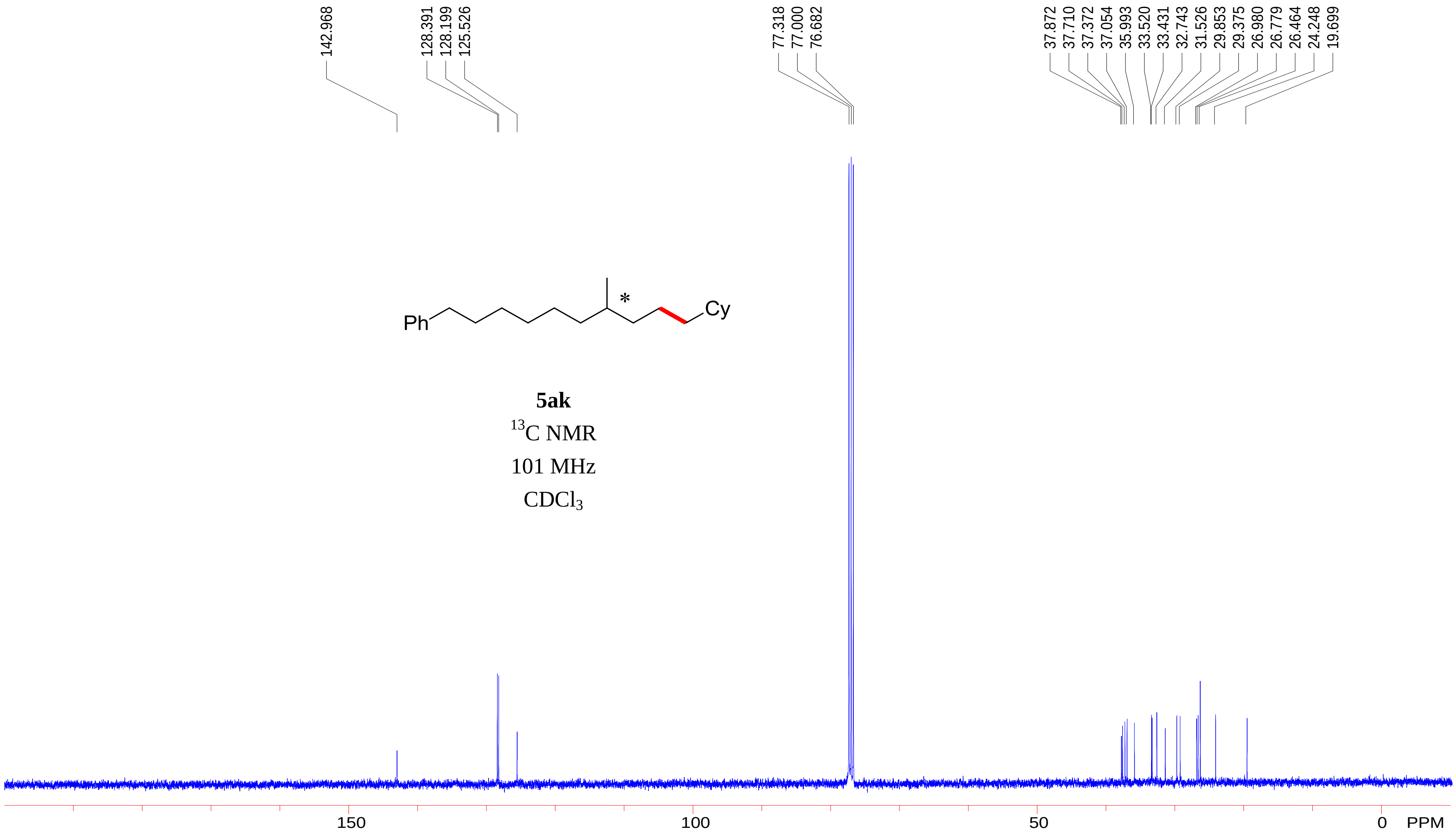

Supplementary Figure 204. ^{13}C NMR spectrum for **5ak**

Translation of Chinese Characters in HPLC spectra to English

描述：HPLC Condition　检测器：Detector　峰号：Peak　面积：Area

色谱图：HPLC Spectra　高度：Height 标记：Note 总计：Total

峰表：Area　Percent Report 保留时间：Remaining Time

描述　: AD-H, n-hexane:iPrOH = 98/2 1.0 ml/min, 220 nm

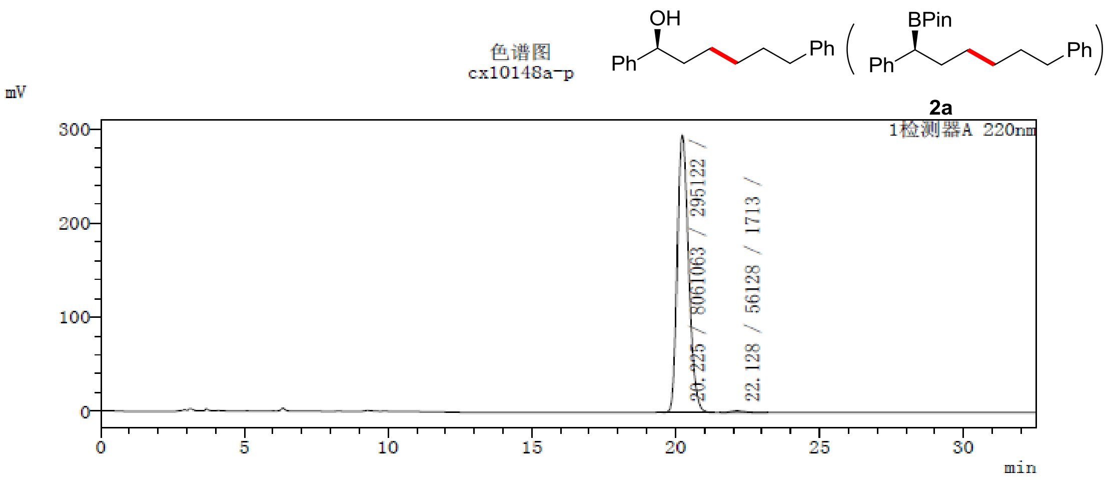

峰表

检测器A 220nm

峰号	保留时间	面积	高度	标记	面积%
1	20.225	8061063	295122		99.309
2	22.128	56128	1713		0.691
总计		8117191	296836		100.000

描述　: AD-H, n-hexane:iPrOH = 98/2 1.0 ml/min, 220 nm

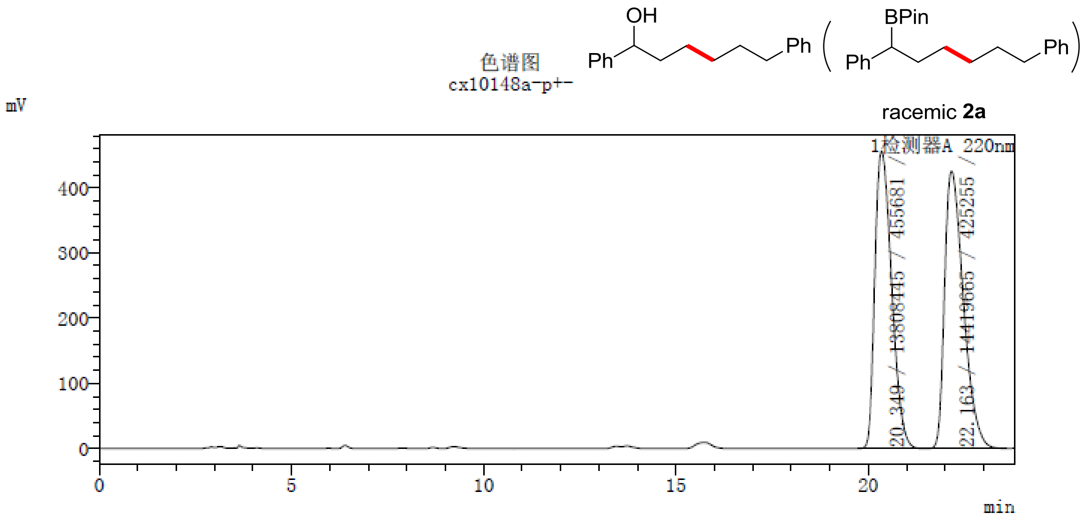

峰表

检测器A 220nm

峰号	保留时间	面积	高度	标记	面积%
1	20.349	13808445	455681		48.917
2	22.163	14419665	425255		51.083
总计		28228109	880936		100.000

Supplementary Figure 205. HPLC spectra for **2a**

Translation of Chinese Characters in HPLC spectra to English

描述：HPLC Condition　检测器：Detector　峰号：Peak　面积：Area
色谱图：HPLC Spectra　高度：Height　标记：Note　总计：Total
峰表：Area　Percent Report　保留时间：Remaining Time

描述　: AS-H, n-Hex/iPrOH = 98/2, 1.0 mL/min, 220 nm

色谱图

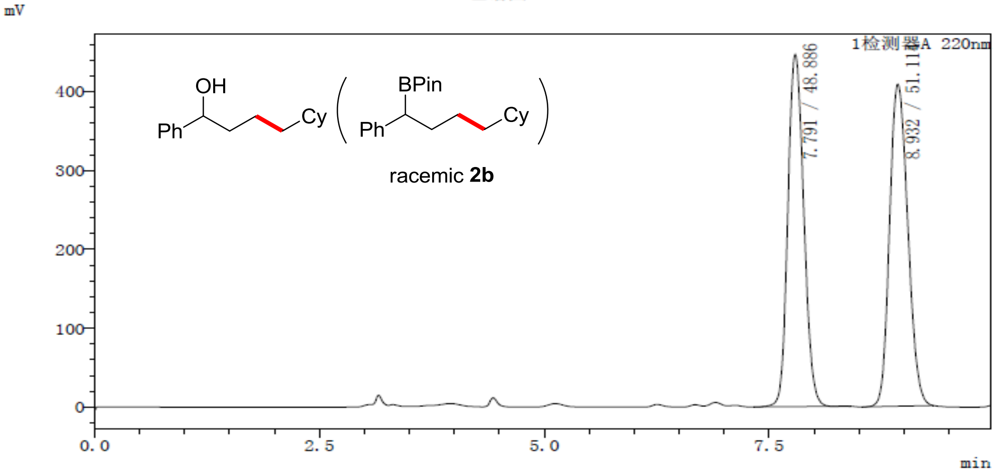

峰表

检测器A 220nm

峰号	保留时间	面积	高度	标记	面积%
1	7.791	5506005	446596	M	48.886
2	8.932	5756951	408522	M	51.114
总计		11262955	855117		100.000

描述　: AS-H, n-Hex/iPrOH = 98/2, 1.0 mL/min, 220 nm

色谱图

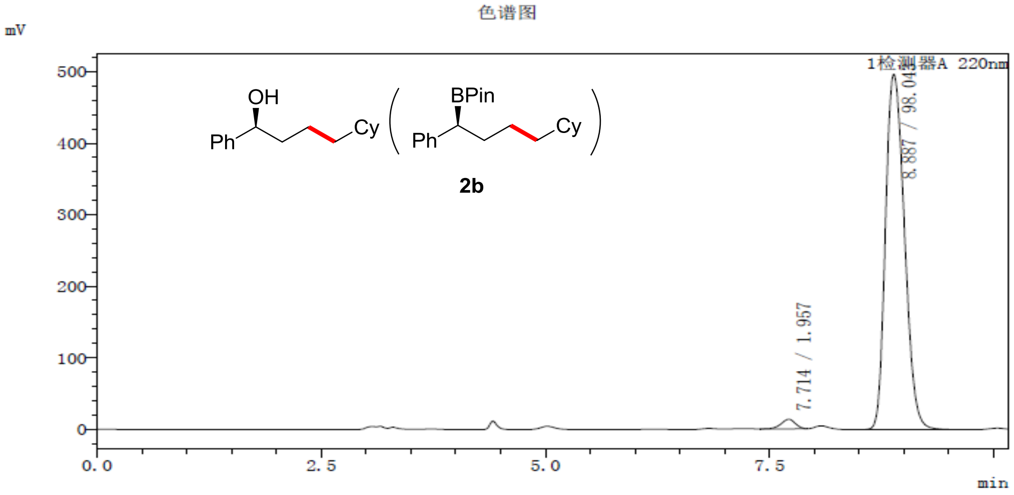

峰表

检测器A 220nm

峰号	保留时间	面积	高度	标记	面积%
1	7.714	145749	13327		1.957
2	8.887	7300265	496681		98.043
总计		7446013	510008		100.000

Supplementary Figure 206. HPLC spectra for **2b**

Translation of Chinese Characters in HPLC spectra to English

描述：HPLC Condition	检测器：Detector	峰号：Peak	面积：Area
色谱图：HPLC Spectra	高度：Height	标记：Note	总计：Total
峰表：Area Percent Report		保留时间：Remaining Time	

描述 : AS-H, n-hexane:iPrOH = 98/2, 1.0 ml/min, 220 nm

色谱图
cx11028a-p

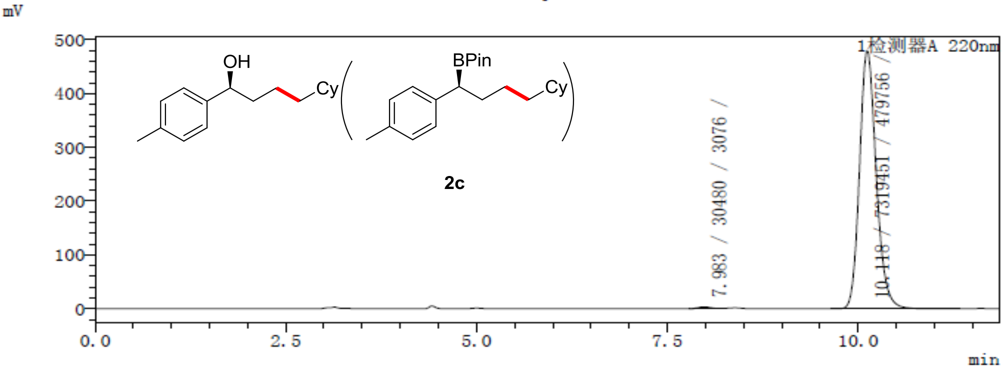

峰表

检测器A 220nm

峰号	保留时间	面积	高度	标记	面积%
1	7.983	30480	3076		0.415
2	10.118	7319451	479756		99.585
总计		7349931	482832		100.000

描述 : AS-H, n-hexane:iPrOH = 98/2, 1.0 ml/min, 220 nm

色谱图
cx11028a-p+-

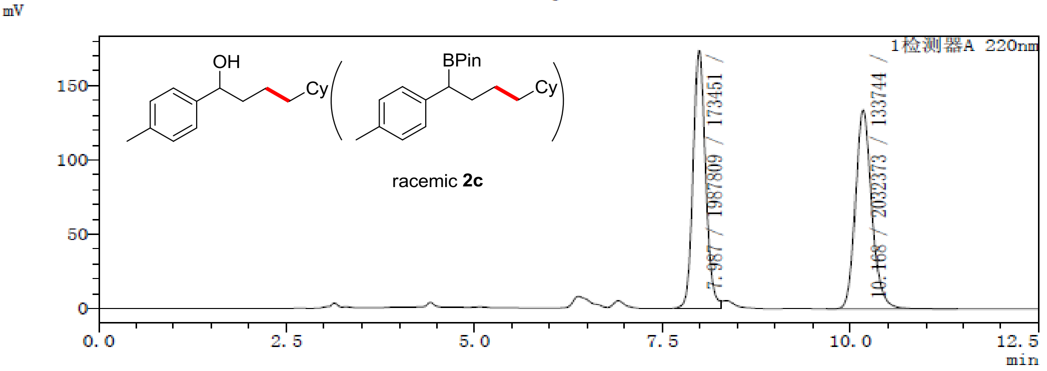

峰表

检测器A 220nm

峰号	保留时间	面积	高度	标记	面积%
1	7.987	1987809	173451	M	49.446
2	10.168	2032373	133744	S	50.554
总计		4020181	307195		100.000

Supplementary Figure 207. HPLC spectra for **2c**

Translation of Chinese Characters in HPLC spectra to English

描述：HPLC Condition　检测器：Detector　峰号：Peak　面积：Area

色谱图：HPLC Spectra　高度：Height　标记：Note　总计：Total

峰表：Area　Percent Report　保留时间：Remaining Time

描述　: AS-H, n-Hex/iPrOH = 98/2, 1.0 mL/min, 220 nm

色谱图

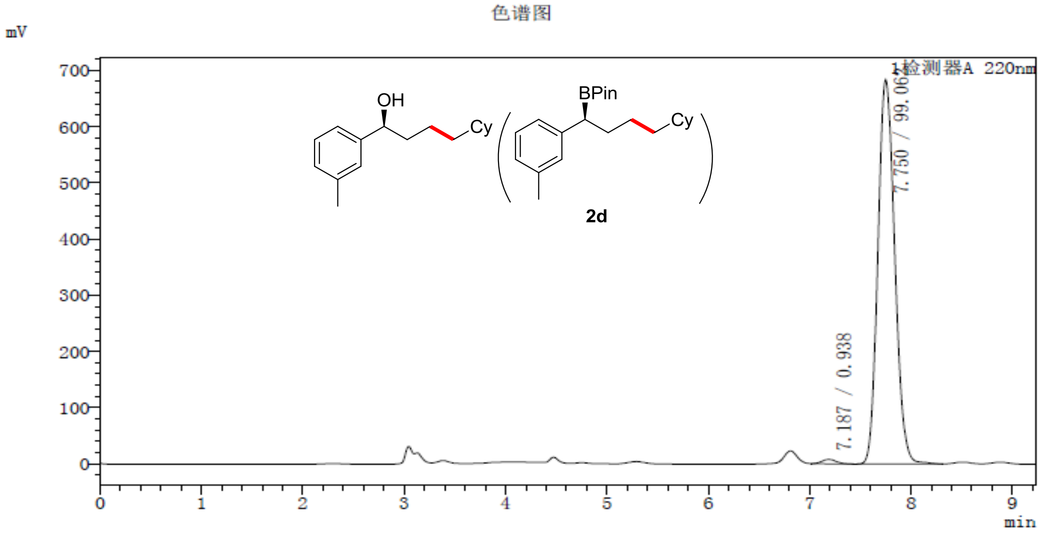

峰表

检测器A 220nm

峰号	保留时间	面积	高度	标记	面积%
1	7.187	74710	7788		0.938
2	7.750	7892991	683786		99.062
总计		7967701	691574		100.000

描述　: AS-H, n-Hex/iPrOH = 98/2, 1.0 mL/min, 220 nm

色谱图

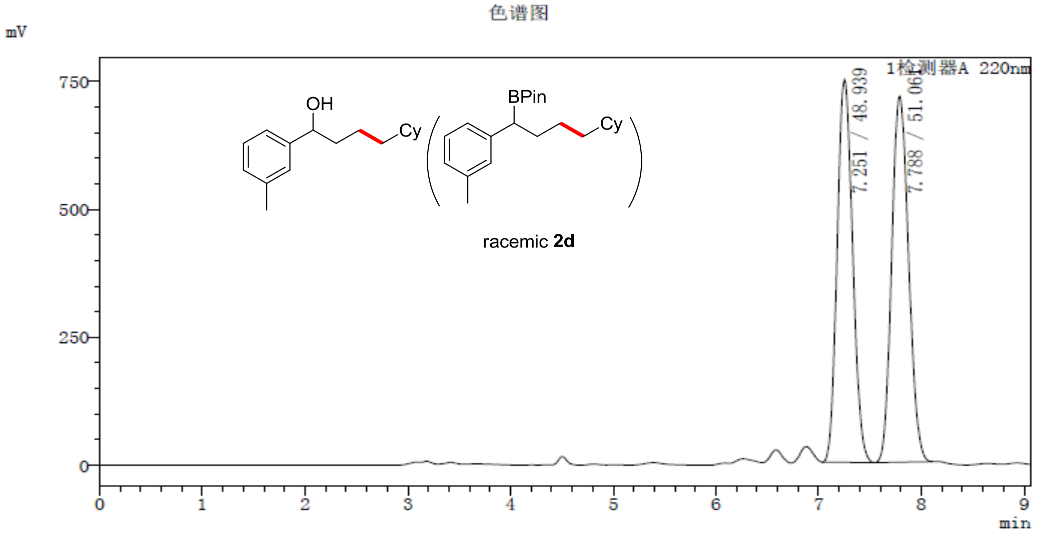

峰表

检测器A 220nm

峰号	保留时间	面积	高度	标记	面积%
1	7.251	7845733	748125		48.939
2	7.788	8185898	716031		51.061
总计		16031631	1464156		100.000

Supplementary Figure 208. HPLC spectra for **2d**

Translation of Chinese Characters in HPLC spectra to English

描述：HPLC Condition　检测器：Detector　峰号：Peak　面积：Area

色谱图：HPLC Spectra　高度：Height　标记：Note　总计：Total

峰表：Area Percent Report　保留时间：Remaining Time

描述 : OD-H, n-hexane:iPrOH = 98/2, 1.0 ml/min, 220 nm

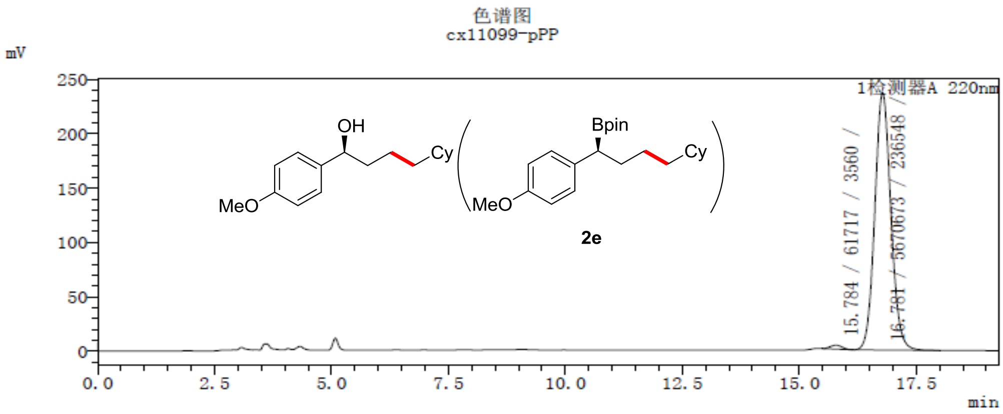

峰表

检测器A 220nm

峰号	保留时间	面积	高度	标记	面积%
1	15.784	61717	3560		1.077
2	16.781	5670673	236548		98.923
总计		5732390	240108		100.000

描述 : OD-H, n-hexane:iPrOH = 98/2, 1.0 ml/min, 220 nm

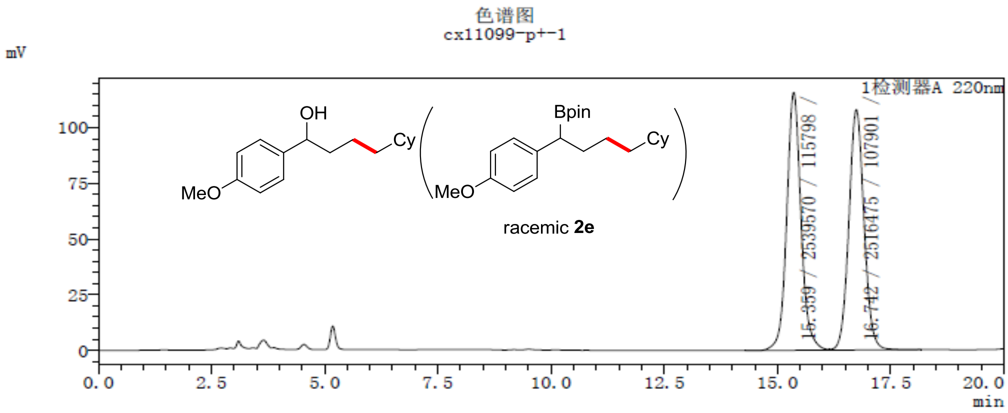

峰表

检测器A 220nm

峰号	保留时间	面积	高度	标记	面积%
1	15.359	2539570	115798		50.228
2	16.742	2516475	107901	V	49.772
总计		5056045	223700		100.000

Supplementary Figure 209. HPLC spectra for **2e**

Translation of Chinese Characters in HPLC spectra to English
描述：HPLC Condition　检测器：Detector　峰号：Peak　面积：Area
色谱图：HPLC Spectra　高度：Height 标记：Note 总计：Total
峰表：Area　Percent Report 保留时间：Remaining Time

描述 : AS-H, n-hexane:iPrOH = 98/2, 1.0 ml/min, 220 nm

色谱图
czy2048-pp

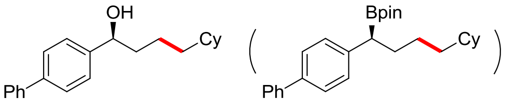

2f

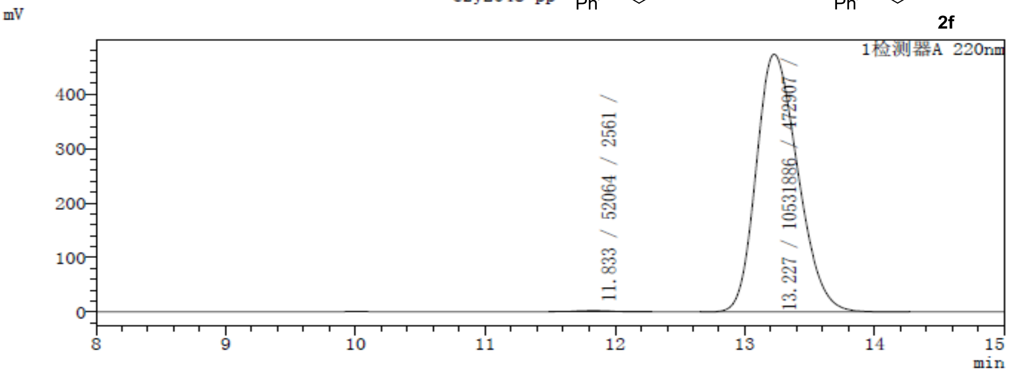

峰表

检测器A 220nm

峰号	保留时间	面积	高度	标记	面积%
1	11.833	52064	2561	M	0.492
2	13.227	10531886	472907		99.508
总计		10583950	475468		100.000

描述 : AS-H, n-hexane:iPrOH = 98/2, 1.0 ml/min, 220 nm

色谱图
czy2048-pp+

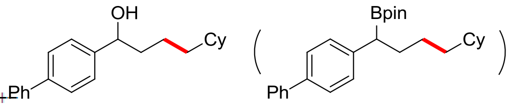

racemic 2f

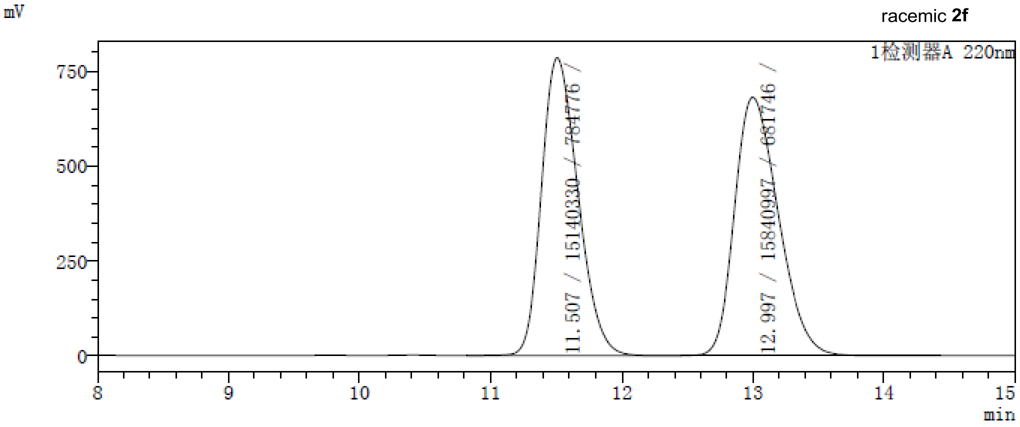

峰表

检测器A 220nm

峰号	保留时间	面积	高度	标记	面积%
1	11.507	15140330	784776	S	48.869
2	12.997	15840997	681746	V	51.131
总计		30981327	1466521		100.000

Supplementary Figure 210. HPLC spectra for **2f**

Translation of Chinese Characters in HPLC spectra to English

描述：HPLC Condition　检测器：Detector　峰号：Peak　面积：Area

色谱图：HPLC Spectra　高度：Height　标记：Note　总计：Total

峰表：Area　Percent Report　保留时间：Remaining Time

描述　: AS-H, n-Hex/iPrOH = 98/2, 1.0 mL/min, 220 nm

色谱图

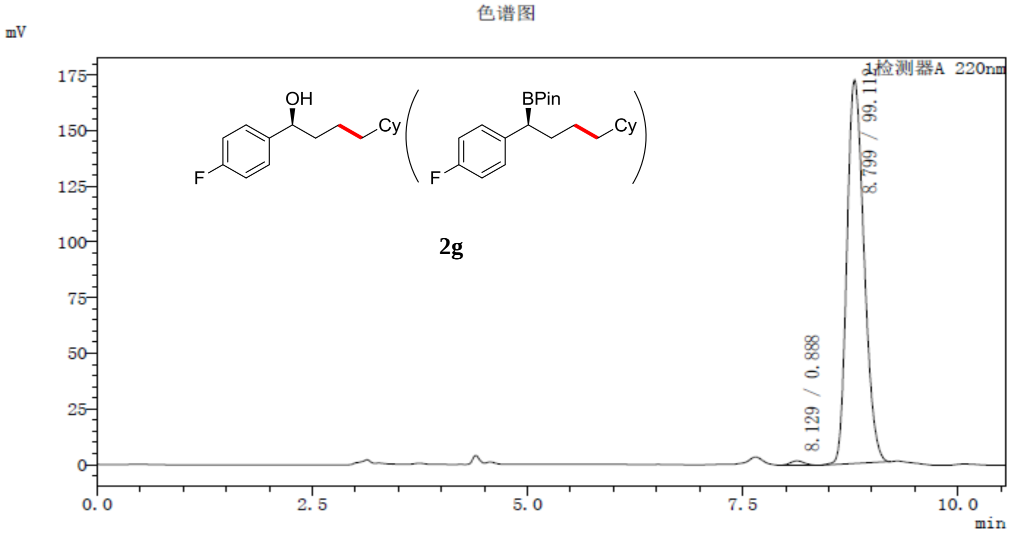

峰表

检测器A 220nm

峰号	保留时间	面积	高度	标记	面积%
1	8.129	21366	1942		0.888
2	8.799	2385469	172274		99.112
总计		2406835	174216		100.000

描述　: AS-H, n-Hex/iPrOH = 98/2, 1.0 mL/min, 220 nm

色谱图

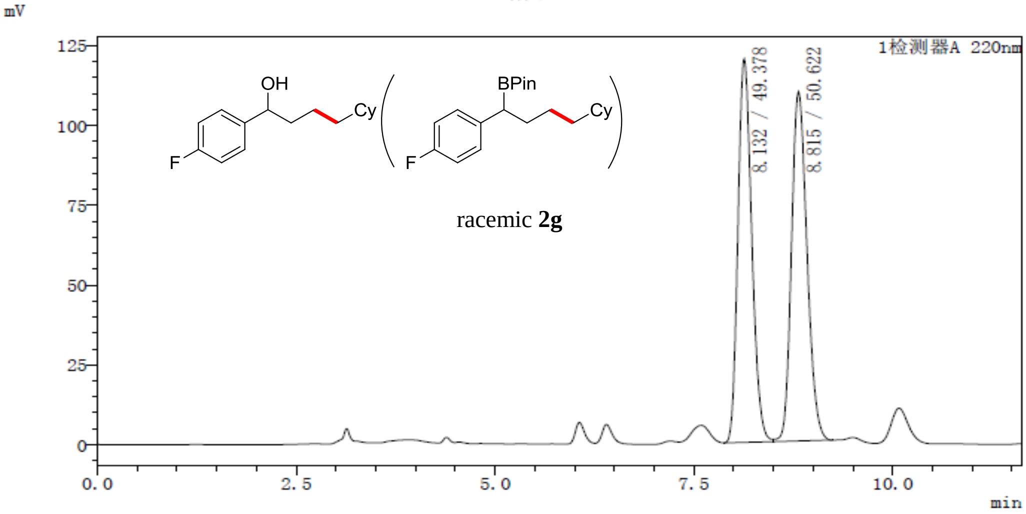

峰表

检测器A 220nm

峰号	保留时间	面积	高度	标记	面积%
1	8.132	1451129	120135		49.378
2	8.815	1487691	109416	V	50.622
总计		2938820	229551		100.000

Supplementary Figure 211. HPLC spectra for **2g**

Translation of Chinese Characters in HPLC spectra to English

描述：HPLC Condition　　检测器：Detector　　峰号：Peak　　面积：Area

色谱图：HPLC Spectra　　高度：Height　　标记：Note　　总计：Total

峰表：Area　Percent Report　　保留时间：Remaining Time

描述　　: AD-H, n-Hex/iPrOH = 98/2, 0.8 mL/min, 220 nm

色谱图

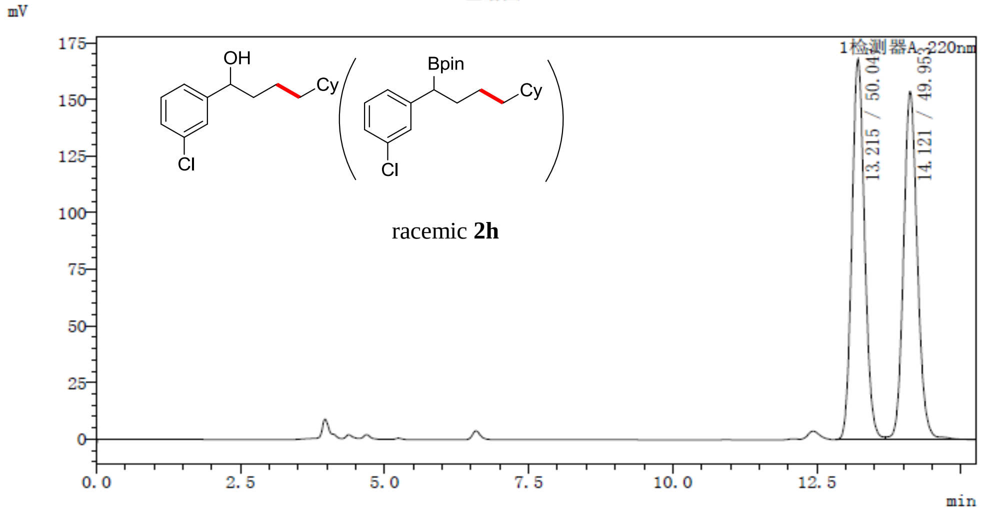

峰表

检测器A 220nm

峰号	保留时间	面积	高度	标记	面积%
1	13.215	2576254	168084		50.047
2	14.121	2571441	153662	V	49.953
总计		5147694	321746		100.000

描述　　: AD-H, n-Hex/iPrOH = 98/2, 0.8 mL/min, 220 nm

色谱图

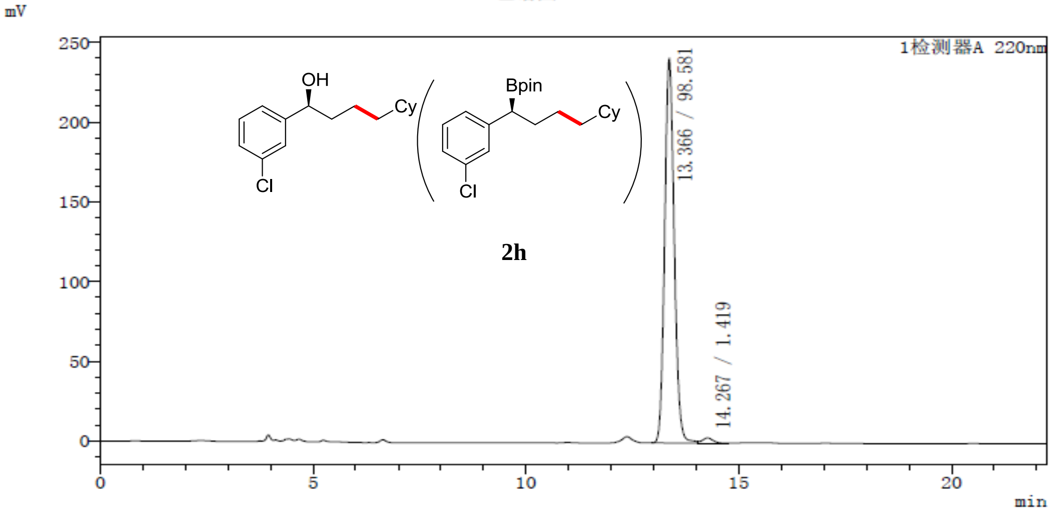

峰表

检测器A 220nm

峰号	保留时间	面积	高度	标记	面积%
1	13.366	3757687	240673		98.581
2	14.267	54091	3259	V	1.419
总计		3811778	243932		100.000

Supplementary Figure 212. HPLC spectra for **2h**

Translation of Chinese Characters in HPLC spectra to English
描述：HPLC Condition　检测器：Detector　峰号：Peak　面积：Area
色谱图：HPLC Spectra　高度：Height 标记：Note 总计：Total
峰表：Area　Percent Report 保留时间：Remaining Time

描述　: AS-H, n-hexane:iPrOH = 99/1, 0.8 ml/min, 220 nm

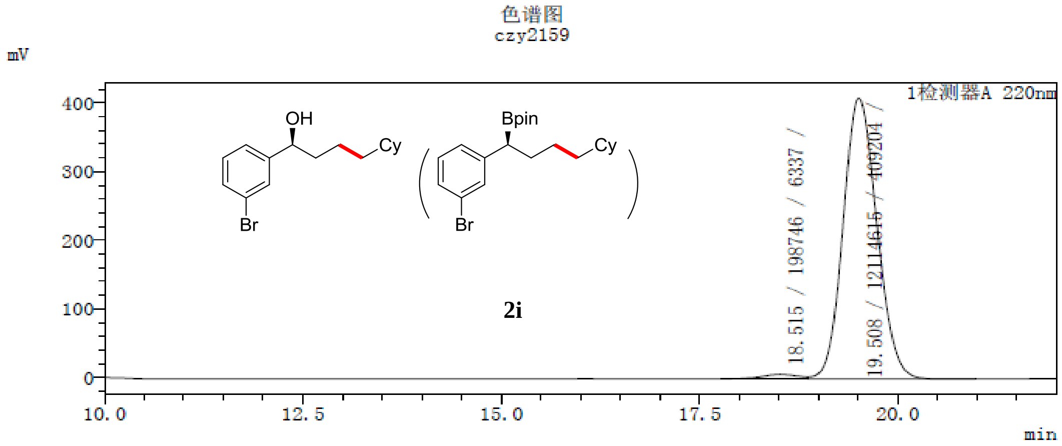

峰表

检测器A 220nm

峰号	保留时间	面积	高度	标记	面积%
1	18.515	198746	6337		1.614
2	19.508	12114615	409204	V	98.386
总计		12313362	415542		100.000

描述　: AS-H, n-hexane:iPrOH = 99/1, 0.8 ml/min, 220 nm

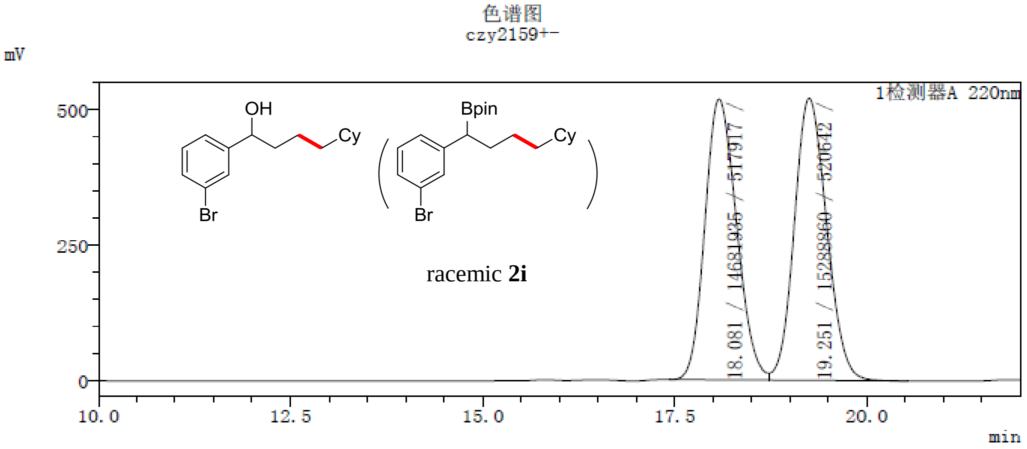

峰表

检测器A 220nm

峰号	保留时间	面积	高度	标记	面积%
1	18.081	14681935	517917		48.987
2	19.251	15288860	520642	V	51.013
总计		29970794	1038560		100.000

Supplementary Figure 213. HPLC spectra for **2i**

Translation of Chinese Characters in HPLC spectra to English

描述：HPLC Condition　检测器：Detector　峰号：Peak　面积：Area

色谱图：HPLC Spectra　高度：Height 标记：Note 总计：Total

峰表：Area　Percent Report 保留时间：Remaining Time

描述　　: AS-H, n-hexane:iPrOH = 98/2, 1.0 ml/min, 220 nm

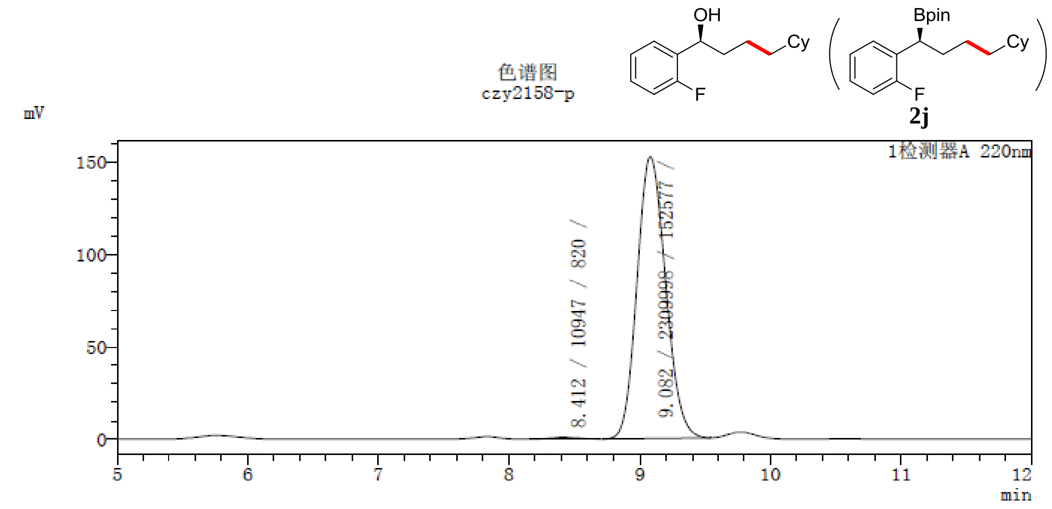

峰表

检测器A 220nm

峰号	保留时间	面积	高度	标记	面积%
1	8.412	10947	820		0.472
2	9.082	2309998	152577		99.528
总计		2320945	153397		100.000

描述　　: AS-H, n-hexane:iPrOH = 98/2, 1.0 ml/min, 220 nm

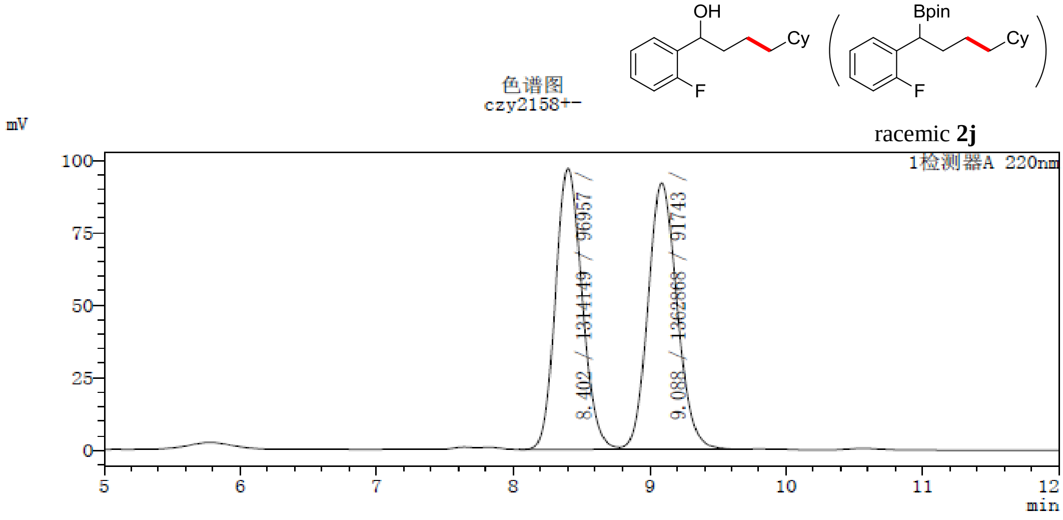

峰表

检测器A 220nm

峰号	保留时间	面积	高度	标记	面积%
1	8.402	1314149	96957		49.090
2	9.088	1362868	91743	V	50.910
总计		2677017	188700		100.000

Supplementary Figure 214. HPLC spectra for **2j**

Translation of Chinese Characters in HPLC spectra to English

描述：HPLC Condition　检测器：Detector　峰号：Peak　面积：Area

色谱图：HPLC Spectra　高度：Height 标记：Note 总计：Total

峰表：Area　Percent Report 保留时间：Remaining Time

描述　: AS-H, n-hexane:iPrOH = 98/2, 1.00 ml/min, 220 nm

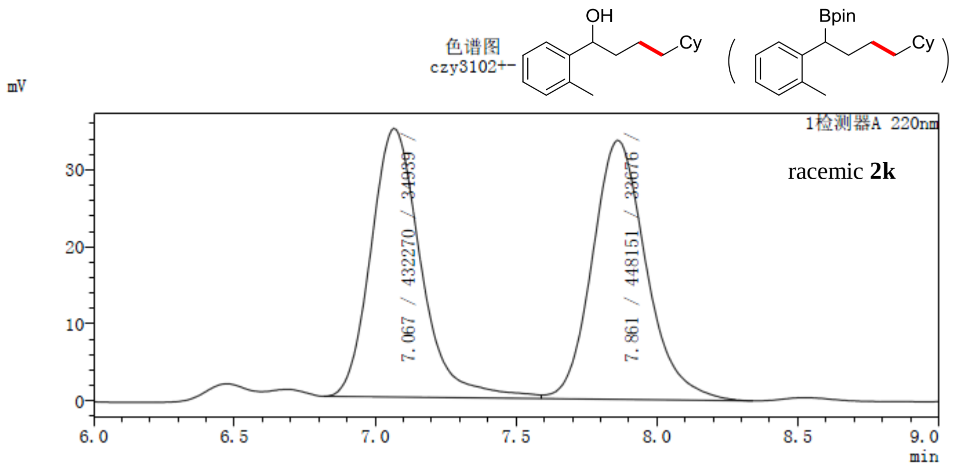

峰表

检测器A 220nm

峰号	保留时间	面积	高度	标记	面积%
1	7.067	432270	34939		49.098
2	7.861	448151	33676	V	50.902
总计		880421	68615		100.000

描述　: AS-H, n-hexane:iPrOH = 98/2, 1.00 ml/min, 220 nm

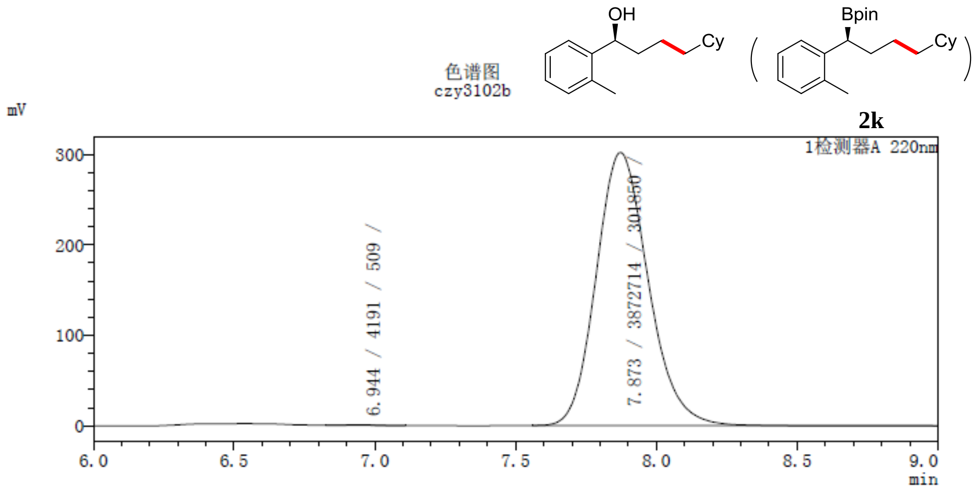

峰表

检测器A 220nm

峰号	保留时间	面积	高度	标记	面积%
1	6.944	4191	509		0.108
2	7.873	3872714	301850	S	99.892
总计		3876905	302359		100.000

Supplementary Figure 215. HPLC spectra for **2k**

Translation of Chinese Characters in HPLC spectra to English

描述：HPLC Condition　检测器：Detector　峰号：Peak　面积：Area

色谱图：HPLC Spectra　高度：Height　标记：Note　总计：Total

峰表：Area Percent Report　保留时间：Remaining Time

描述 : AD-H, n-hexane:iPrOH = 98/2, 1.0 ml/min, 220 nm

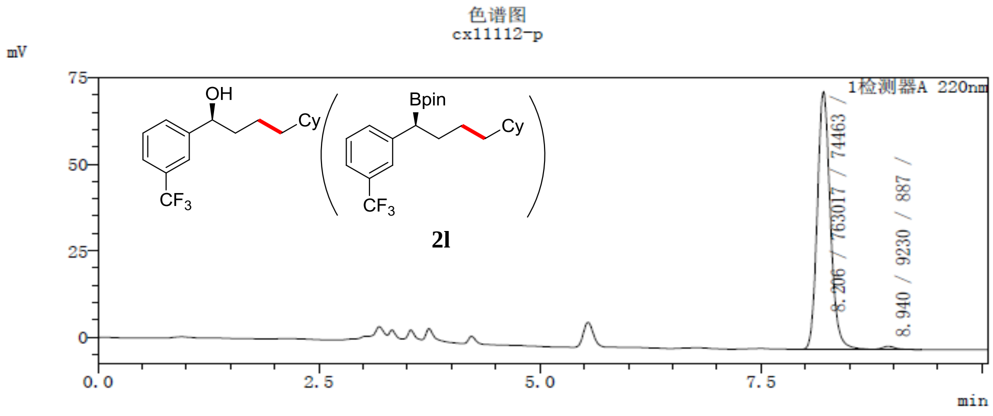

峰表

检测器A 220nm

峰号	保留时间	面积	高度	标记	面积%
1	8.206	763017	74463		98.805
2	8.940	9230	887		1.195
总计		772248	75350		100.000

描述 : AD-H, n-hexane:iPrOH = 98/2, 1.0 ml/min, 220 nm

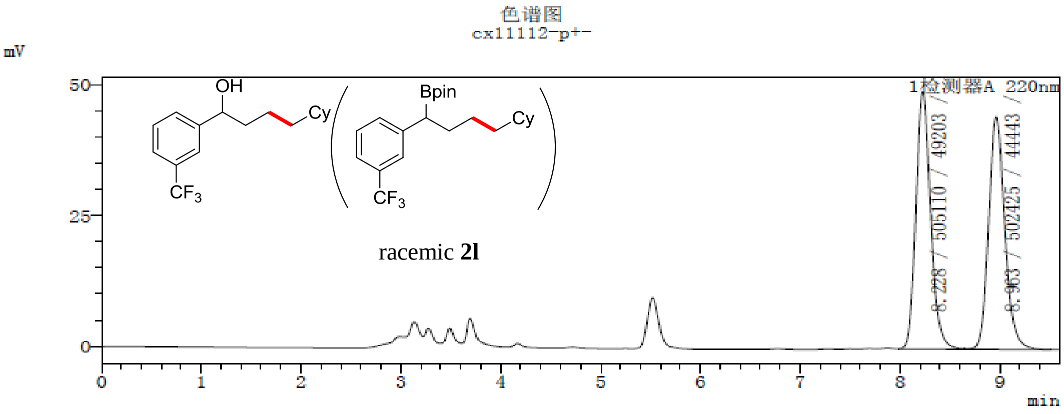

峰表

检测器A 220nm

峰号	保留时间	面积	高度	标记	面积%
1	8.228	505110	49203		50.133
2	8.963	502425	44443	V	49.867
总计		1007535	93646		100.000

Supplementary Figure 216. HPLC spectra for **2l**

Translation of Chinese Characters in HPLC spectra to English

描述：HPLC Condition　　检测器：Detector　峰号：Peak　　面积：Area

色谱图：HPLC Spectra　　高度：Height　　标记：Note　　总计：Total

峰表：Area　Percent Report　　　　　保留时间：Remaining Time

描述 [illegible] : AS-H, n-hexane:iPrOH = 98/2, 1.0 ml/min, 220 nm

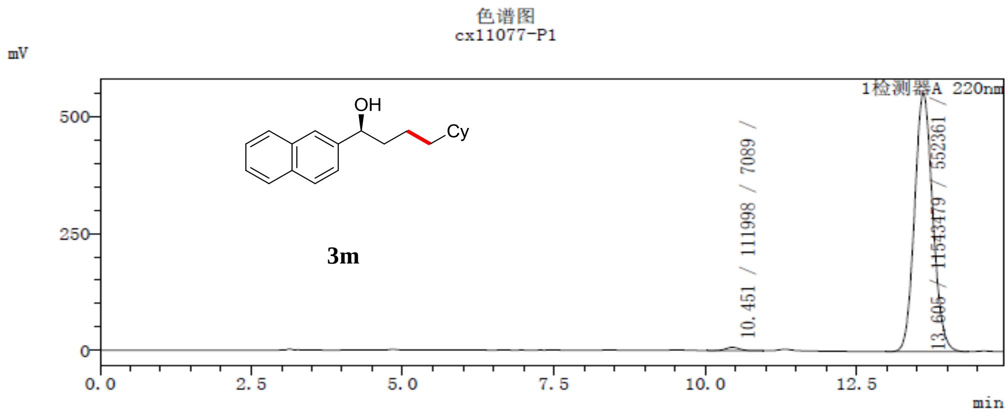

峰表

检测器A 220nm

峰号	保留时间	面积	高度	标记	面积%
1	10.451	111998	7089		0.961
2	13.605	11543479	552361		99.039
总计		11655477	559451		100.000

描述 [illegible] : AS-H, n-hexane:iPrOH = 98/2, 1.0 ml/min, 220 nm

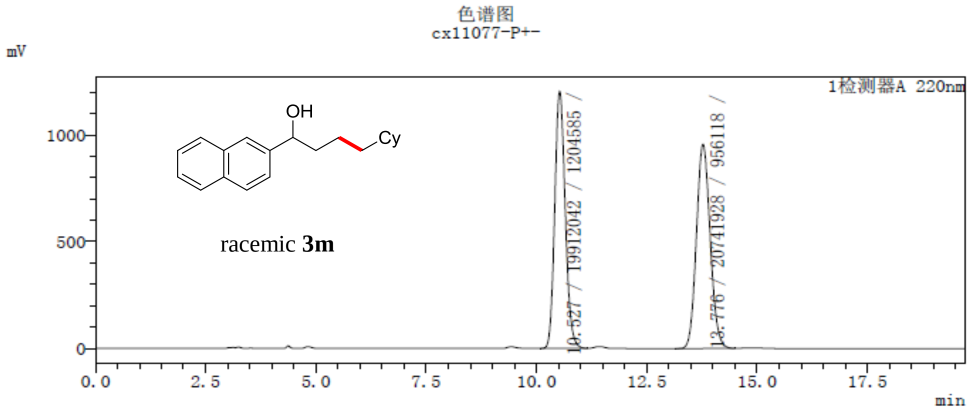

峰表

检测器A 220nm

峰号	保留时间	面积	高度	标记	面积%
1	10.527	19912042	1204585		48.979
2	13.776	20741928	956118		51.021
总计		40653971	2160704		100.000

Supplementary Figure 217. HPLC spectra for **3m**

Translation of Chinese Characters in HPLC spectra to English
描述：HPLC Condition　检测器：Detector　峰号：Peak　面积：Area
色谱图：HPLC Spectra　高度：Height 标记：Note 总计：Total
峰表：Area Percent Report 保留时间：Remaining Time

描述　　: AS-H, n-hexane:iPrOH = 98/2, 1.0 ml/min, 220 nm

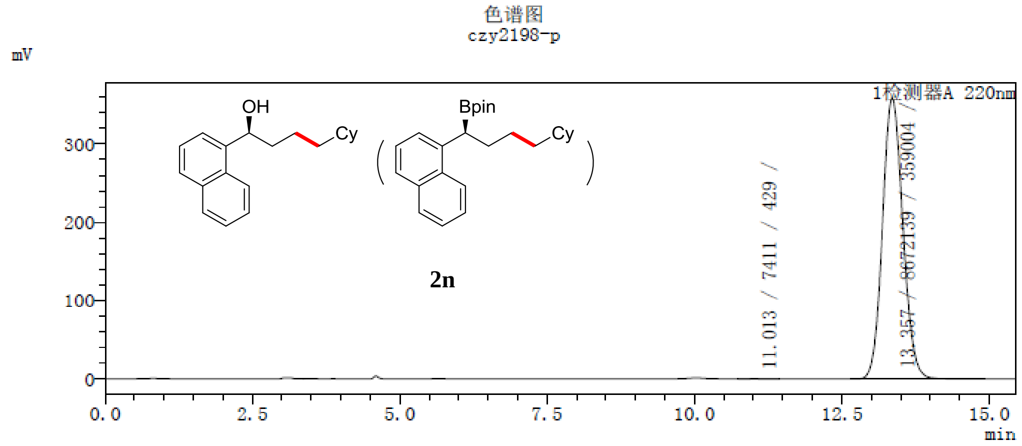

峰表

检测器A 220nm

峰号	保留时间	面积	高度	标记	面积%
1	11.013	7411	429		0.085
2	13.357	8672139	359004		99.915
总计		8679549	359433		100.000

描述　　: AS-H, n-hexane:iPrOH = 98/2, 1.0 ml/min, 220 nm

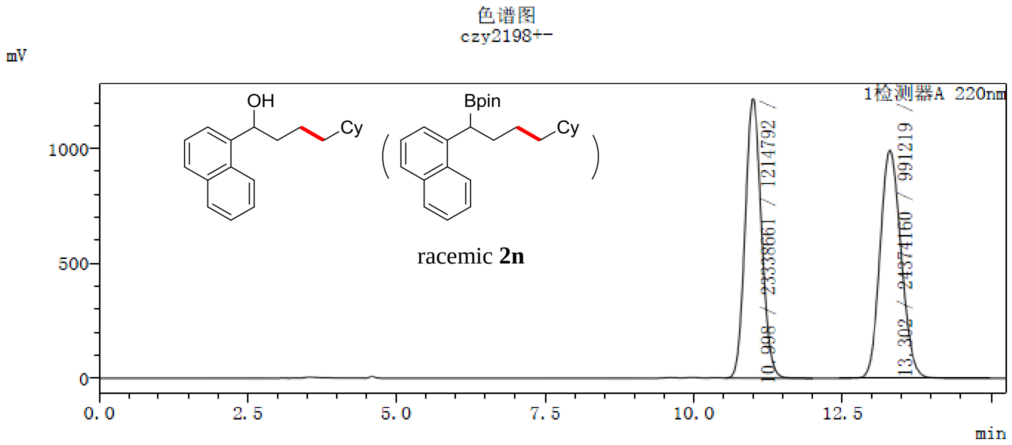

峰表

检测器A 220nm

峰号	保留时间	面积	高度	标记	面积%
1	10.998	23338661	1214792		48.915
2	13.302	24374160	991219		51.085
总计		47712820	2206012		100.000

Supplementary Figure 218. HPLC spectra for **2n**

Translation of Chinese Characters in HPLC spectra to English

描述：HPLC Condition　　检测器：Detector　　峰号：Peak　　面积：Area

色谱图：HPLC Spectra　　高度：Height　　标记：Note　　总计：Total

峰表：Area Percent Report　　保留时间：Remaining Time

描述 : Ad-H, n-hexane:iPrOH = 98/2, 1.0 ml/min, 220 nm

色谱图
cx11118-p

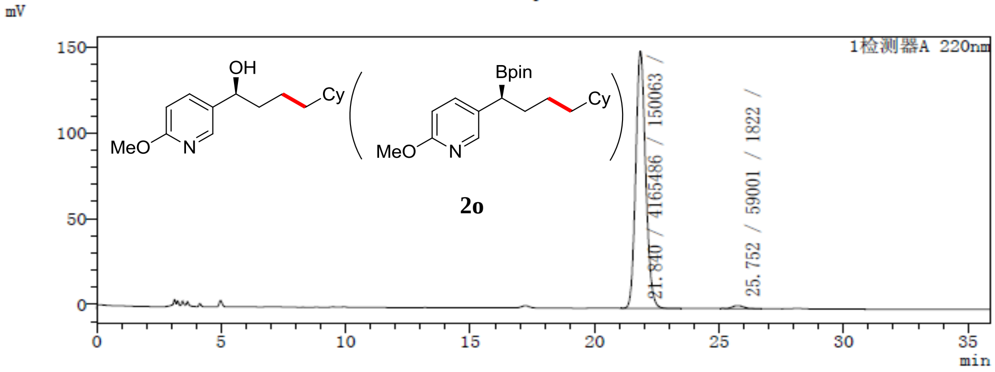

峰表

检测器A 220nm

峰号	保留时间	面积	高度	标记	面积%
1	21.840	4165486	150063		98.603
2	25.752	59001	1822		1.397
总计		4224487	151885		100.000

描述 : Ad-H, n-hexane:iPrOH = 98/2, 1.0 ml/min, 220 nm

色谱图
cx11118-p+-

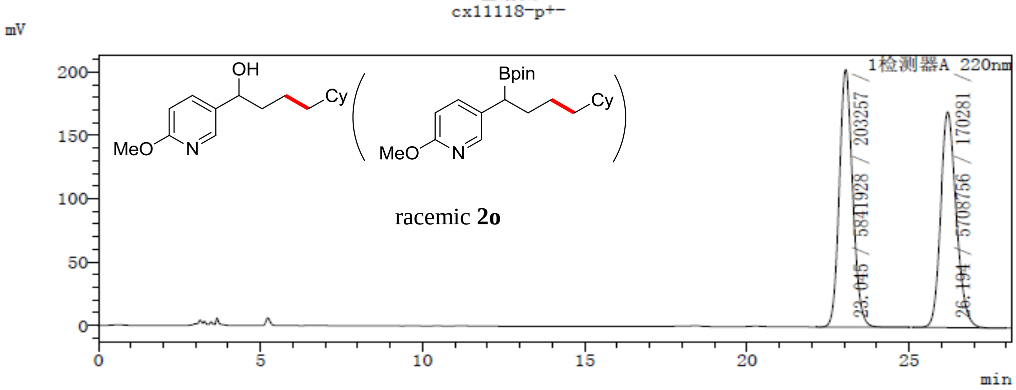

峰表

检测器A 220nm

峰号	保留时间	面积	高度	标记	面积%
1	23.045	5841928	203257		50.576
2	26.194	5708756	170281		49.424
总计		11550684	373539		100.000

Supplementary Figure 219. HPLC spectra for **2o**

Translation of Chinese Characters in HPLC spectra to English
描述：HPLC Condition　　检测器：Detector　峰号：Peak　　面积：Area
色谱图：HPLC Spectra　　高度：Height 标记：Note 总计：Total
峰表：Area　Percent Report 保留时间：Remaining Time

描述　　: AS-H, n-Hexane/iPrOH =99/1, 1 mL/min, 220 nm

色谱图

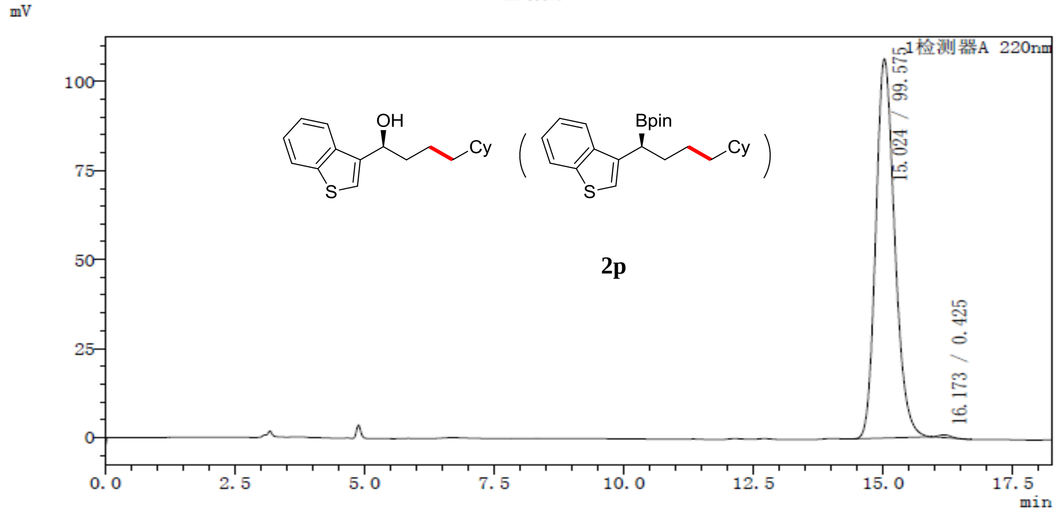

峰表

检测器A 220nm

峰号	保留时间	面积	高度	标记	面积%
1	15.024	2709225	106510		99.575
2	16.173	11566	762	M	0.425
总计		2720791	107272		100.000

描述　　: AS-H, n-Hexane/iPrOH =99/1, 1 mL/min, 220 nm

色谱图

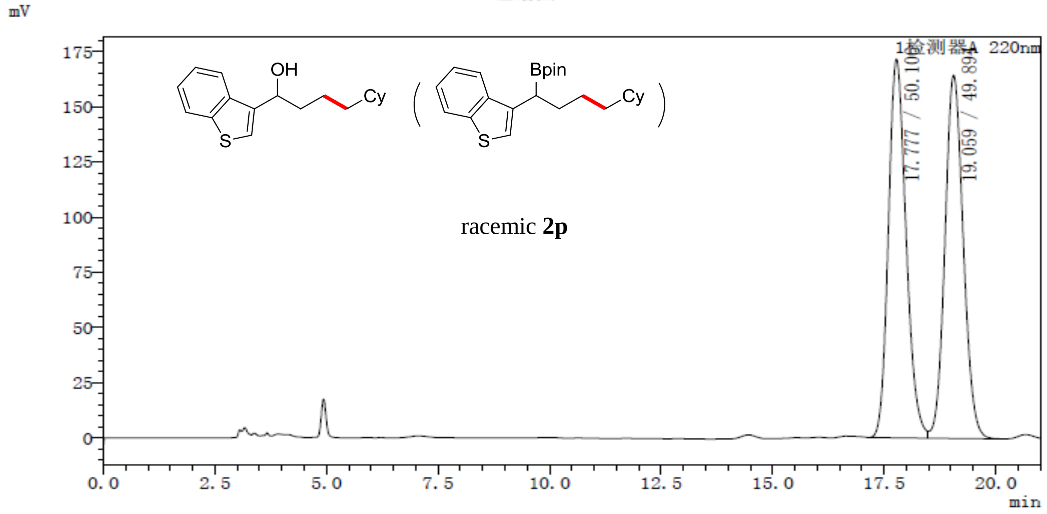

峰表

检测器A 220nm

峰号	保留时间	面积	高度	标记	面积%
1	17.777	4723790	171493		50.106
2	19.059	4703781	164478	V	49.894
总计		9427571	335971		100.000

Supplementary Figure 220. HPLC spectra for **2p**

Translation of Chinese Characters in HPLC spectra to English

描述：HPLC Condition　　检测器：Detector　　峰号：Peak　　面积：Area

色谱图：HPLC Spectra　　高度：Height　　标记：Note　　总计：Total

峰表：Area　Percent Report　　保留时间：Remaining Time

描述　　: AS-H, n-hexane:iPrOH = 98/2, 1.0 ml/min, 220 nm

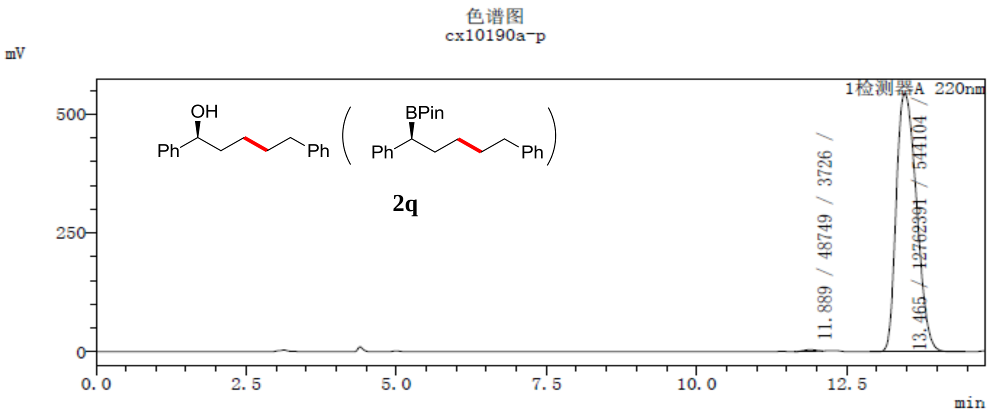

峰表

检测器A 220nm

峰号	保留时间	面积	高度	标记	面积%
1	11.889	48749	3726		0.381
2	13.465	12762391	544104		99.619
总计		12811141	547830		100.000

描述　　: AS-H, n-hexane:iPrOH = 98/2, 1.0 ml/min, 220 nm

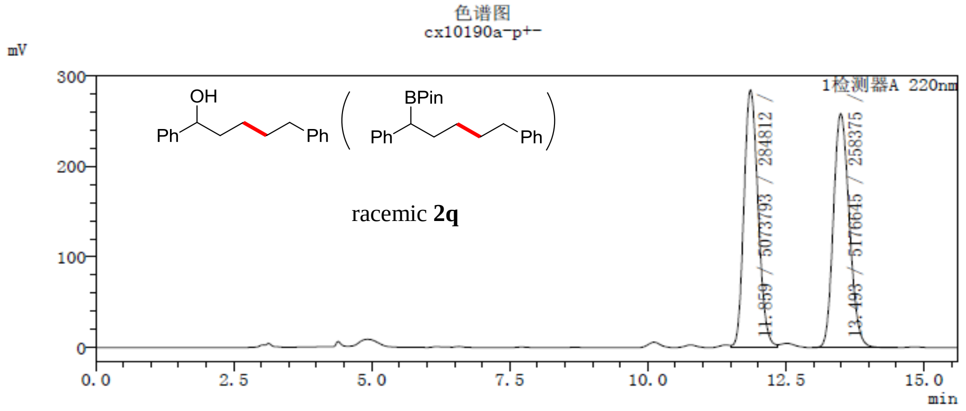

峰表

检测器A 220nm

峰号	保留时间	面积	高度	标记	面积%
1	11.859	5073793	284812	M	49.498
2	13.493	5176645	258375	S	50.502
总计		10250438	543187		100.000

Supplementary Figure 221. HPLC spectra for **2q**

Translation of Chinese Characters in HPLC spectra to English

描述：HPLC Condition	检测器：Detector	峰号：Peak	面积：Area
色谱图：HPLC Spectra	高度：Height	标记：Note	总计：Total
峰表：Area Percent Report		保留时间：Remaining Time	

[illegible]
描述 : OJ-H, n-hexane:iPrOH = 99/1, 0.5 ml/min, 220 nm

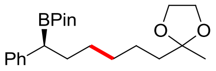

2r

色谱图
cx11074-p

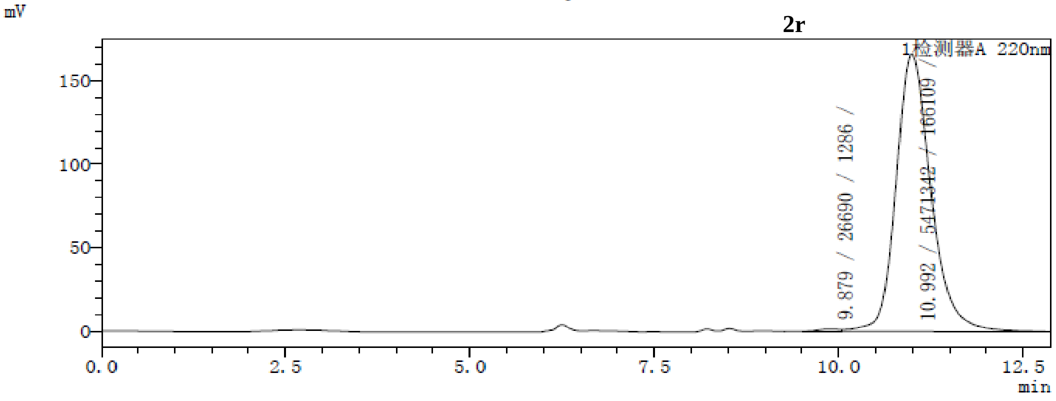

峰表

检测器A 220nm

峰号	保留时间	面积	高度	标记	面积%
1	9.879	26690	1286		0.485
2	10.992	5471342	166109	V	99.515
总计		5498032	167395		100.000

[illegible]
描述 : OJ-H, n-hexane:iPrOH = 99/1, 0.5 ml/min, 220 nm

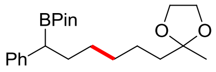

色谱图
cx11074-p+-1

racemic **2r**

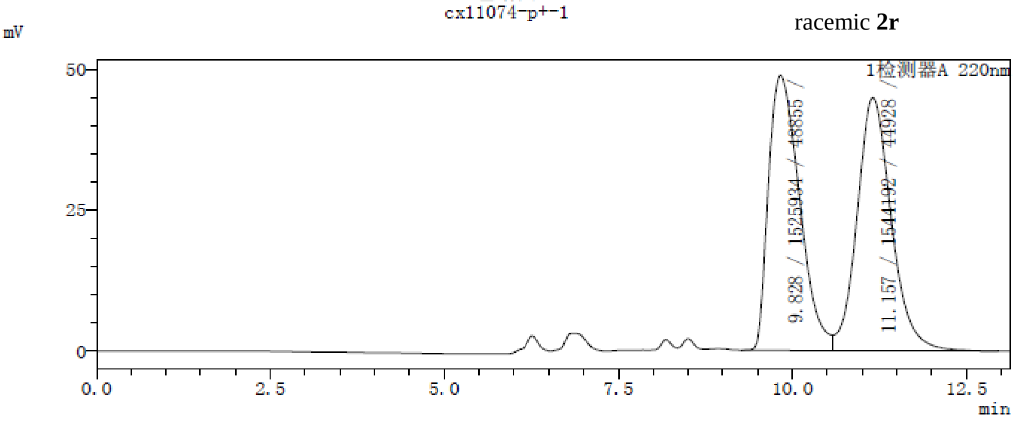

峰表

检测器A 220nm

峰号	保留时间	面积	高度	标记	面积%
1	9.828	1525934	48855		49.703
2	11.157	1544192	44928	V	50.297
总计		3070127	93784		100.000

Supplementary Figure 222. HPLC spectra for **2r**

Translation of Chinese Characters in HPLC spectra to English

描述：HPLC Condition　　检测器：Detector　　峰号：Peak　　面积：Area

色谱图：HPLC Spectra　　高度：Height　　标记：Note　　总计：Total

峰表：Area　Percent Report　　保留时间：Remaining Time

描述　　: AS-H, n-Hex/iPrOH = 98/2, 1.0 mL/min, 220 nm

色谱图

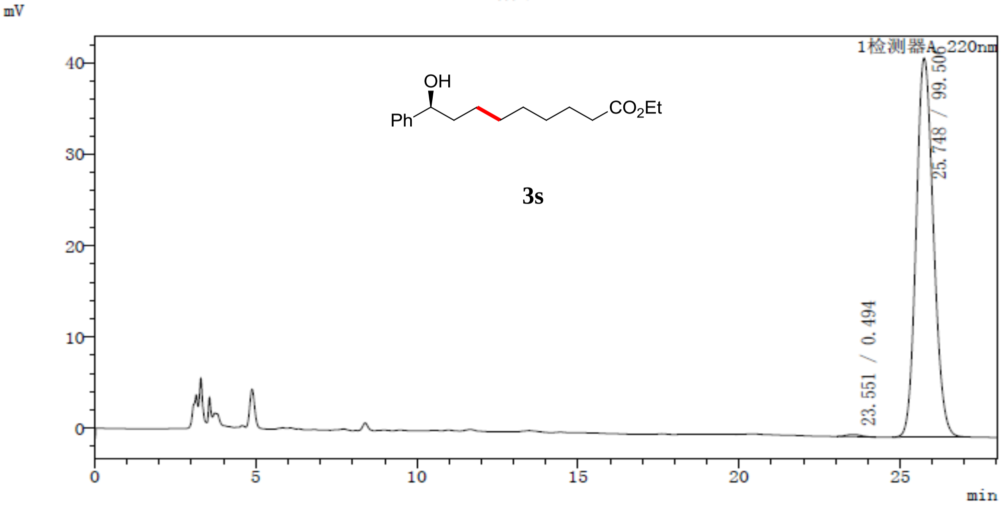

峰表

检测器A 220nm

峰号	保留时间	面积	高度	标记	面积%
1	23.551	7630	251		0.494
2	25.748	1537859	41487		99.506
总计		1545489	41738		100.000

描述　　: AS-H, n-Hex/iPrOH = 98/2, 1.0 mL/min, 220 nm

色谱图

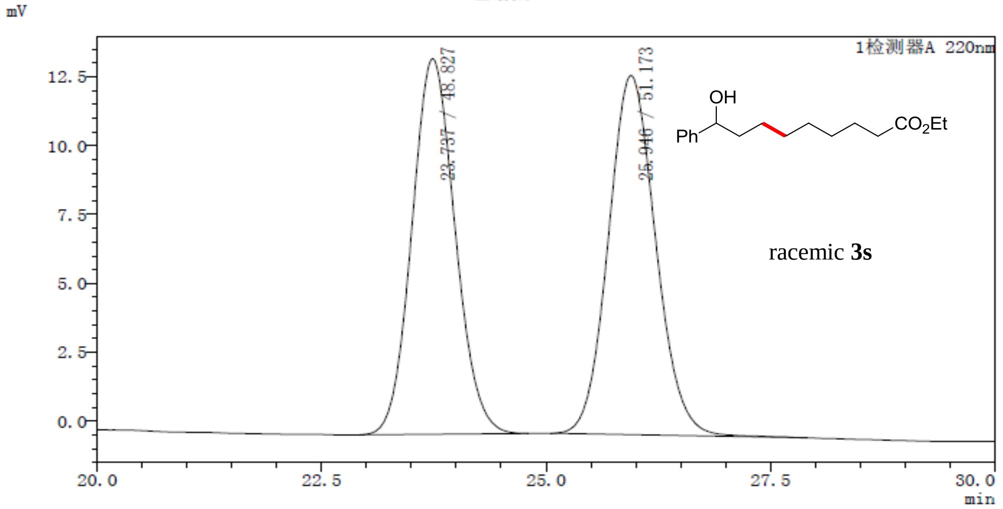

峰表

检测器A 220nm

峰号	保留时间	面积	高度	标记	面积%
1	23.737	456058	13629		48.827
2	25.946	477961	13034		51.173
总计		934019	26663		100.000

Supplementary Figure 223. HPLC spectra for **3s**

Translation of Chinese Characters in HPLC spectra to English

描述：HPLC Condition	检测器：Detector	峰号：Peak	面积：Area
色谱图：HPLC Spectra	高度：Height	标记：Note	总计：Total
峰表：Area Percent Report		保留时间：Remaining Time	

描述 : OD-H, n-Hexane/iPrOH = 90/10, 1.0 mL/min, 220 nm

色谱图

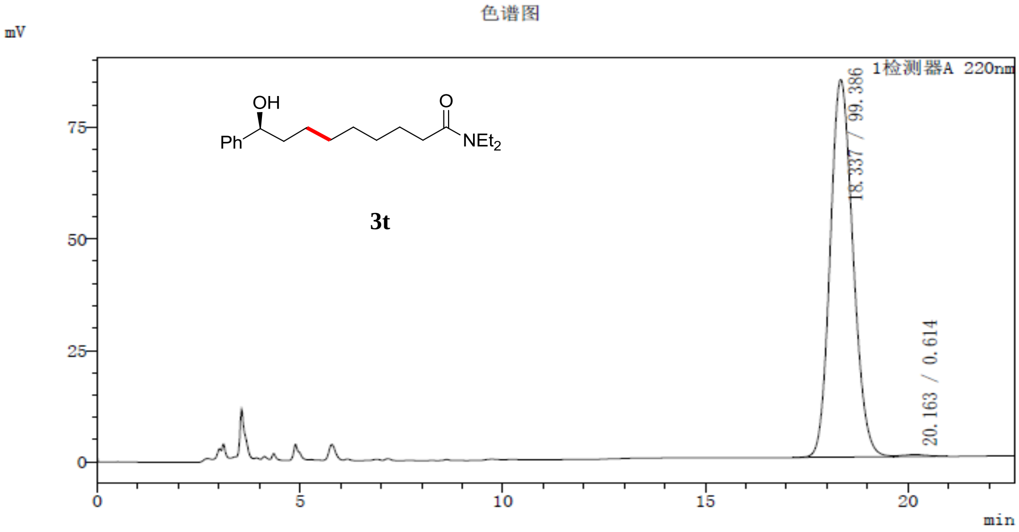

峰表

检测器A 220nm

峰号	保留时间	面积	高度	标记	面积%
1	18.337	3313378	84586		99.386
2	20.163	20476	470	V	0.614
总计		3333854	85056		100.000

描述 : OD-H, n-Hexane/iPrOH = 90/10, 1.0 mL/min, 220 nm

色谱图

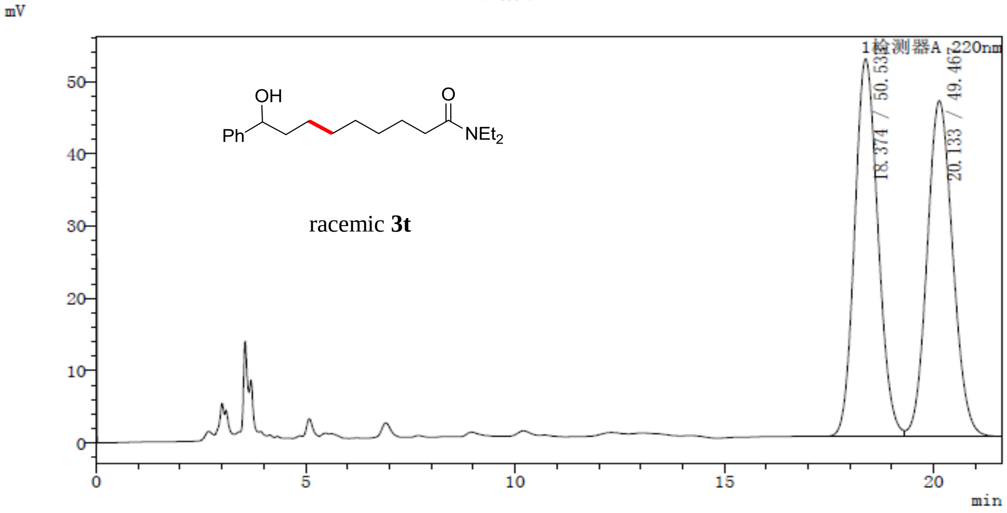

峰表

检测器A 220nm

峰号	保留时间	面积	高度	标记	面积%
1	18.374	2020718	52288		50.533
2	20.133	1978095	46478	V	49.467
总计		3998813	98766		100.000

Supplementary Figure 224. HPLC spectra for **3t**

Translation of Chinese Characters in HPLC spectra to English

描述：HPLC Condition　　检测器：Detector　　峰号：Peak　　面积：Area

色谱图：HPLC Spectra　　高度：Height　　标记：Note　　总计：Total

峰表：Area　Percent Report　　保留时间：Remaining Time

描述 : AS-H, n-hexane:iPrOH = 90/10, 1.0ml/min, 220nm

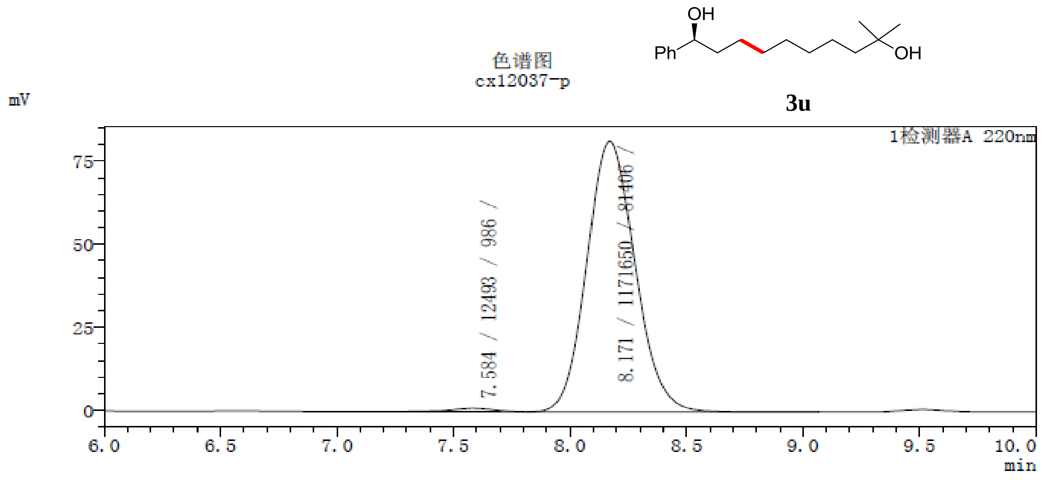

峰表

检测器A 220nm

峰号	保留时间	面积	高度	标记	面积%
1	7.584	12493	986	V	1.055
2	8.171	1171650	81406		98.945
总计		1184143	82392		100.000

描述 : AS-H, n-hexane:iPrOH = 90/10, 1.0ml/min, 220nm

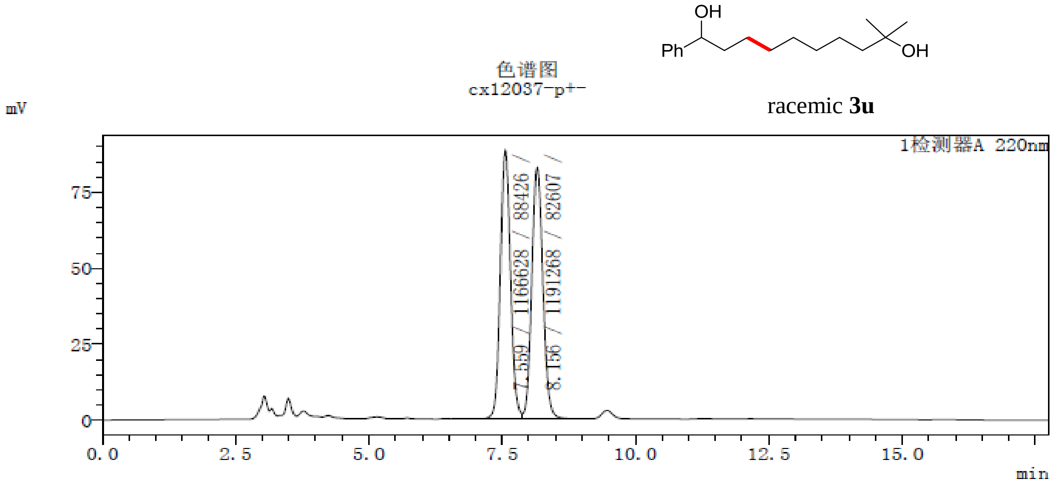

峰表

检测器A 220nm

峰号	保留时间	面积	高度	标记	面积%
1	7.559	1166628	88426		49.478
2	8.156	1191268	82607	V	50.522
总计		2357896	171033		100.000

Supplementary Figure 225. HPLC spectra for **3u**

Translation of Chinese Characters in HPLC spectra to English

描述：HPLC Condition　检测器：Detector　峰号：Peak　面积：Area

色谱图：HPLC Spectra　高度：Height 标记：Note 总计：Total

峰表：Area　Percent Report 保留时间：Remaining Time

描述　: AS-H, n-Hexane/iPrOH = 95/5, 1.0 mL/min, 220 nm

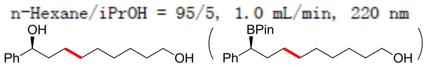

色谱图

3v

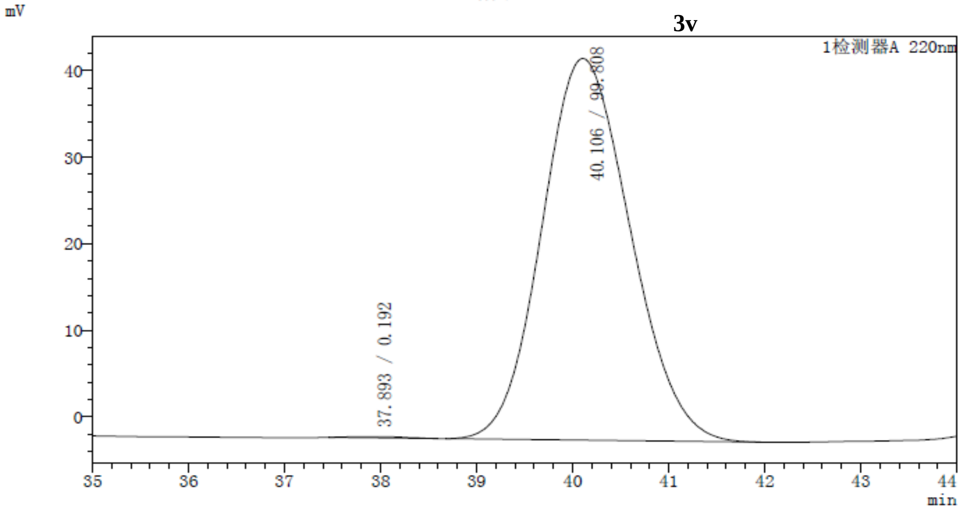

峰表

检测器A 220nm

峰号	保留时间	面积	高度	标记	面积%
1	37.893	5406	131		0.192
2	40.106	2814484	44049		99.808
总计		2819890	44181		100.000

描述　: AS-H, n-Hexane/iPrOH = 95/5, 1.0 mL/min, 220 nm

色谱图

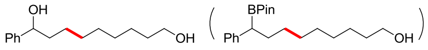

racemic **3v**

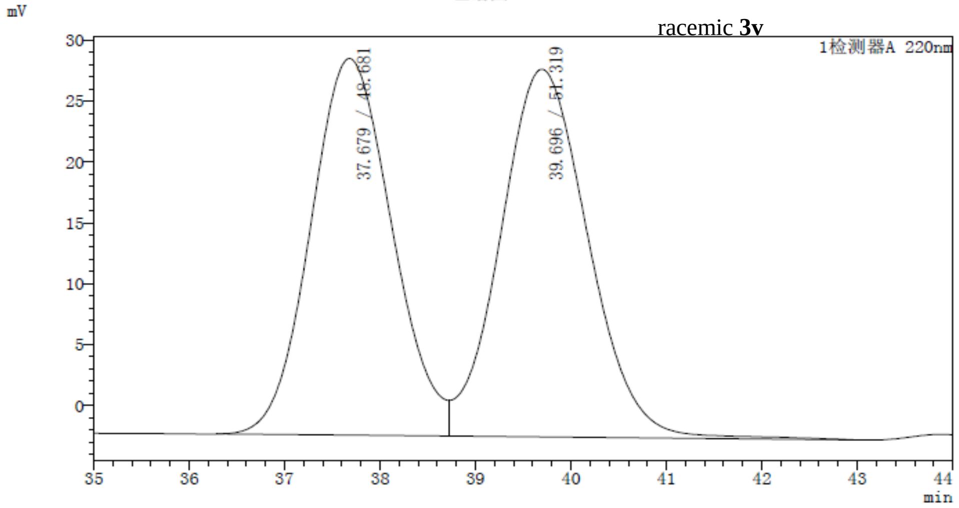

峰表

检测器A 220nm

峰号	保留时间	面积	高度	标记	面积%
1	37.679	1830871	30951		48.681
2	39.696	1930102	30214	V	51.319
总计		3760972	61165		100.000

Supplementary Figure 226. HPLC spectra for **3v**

Translation of Chinese Characters in HPLC spectra to English

描述：HPLC Condition　检测器：Detector　峰号：Peak　面积：Area
色谱图：HPLC Spectra　高度：Height　标记：Note　总计：Total
峰表：Area Percent Report　保留时间：Remaining Time

描述 : AS-H, n-Hex/iPrOH = 80/20, 1.0 mL/min, 220 nm

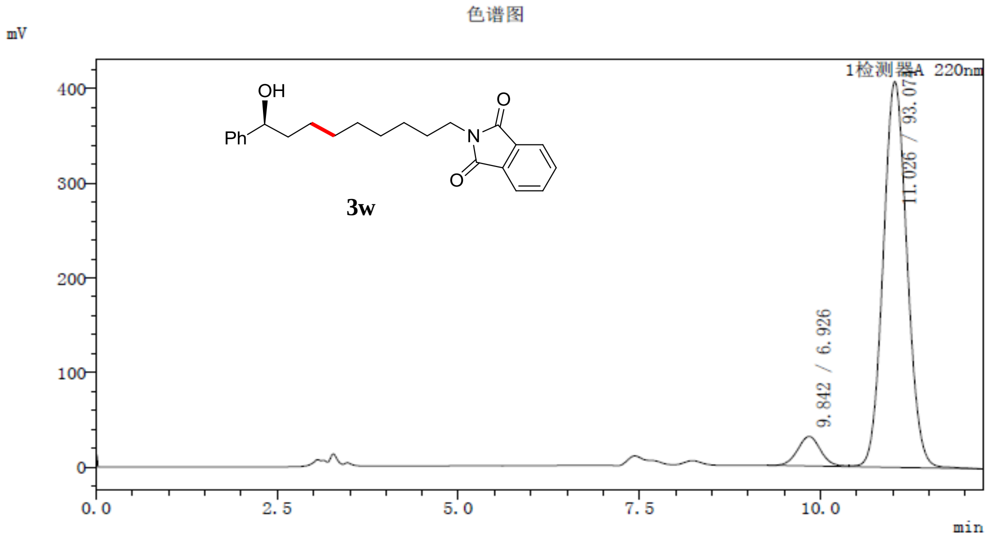

峰表

检测器A 220nm

峰号	保留时间	面积	高度	标记	面积%
1	9.842	695440	31229		6.926
2	11.026	9346282	407776	V	93.074
总计		10041722	439004		100.000

描述 : AS-H, n-Hex/iPrOH = 80/20, 1.0 mL/min, 220 nm

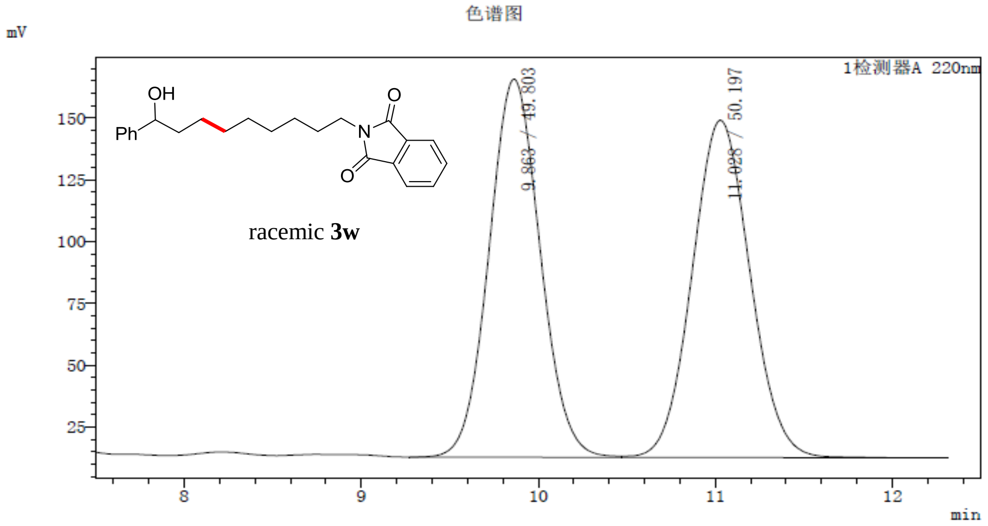

峰表

检测器A 220nm

峰号	保留时间	面积	高度	标记	面积%
1	9.863	3078782	153087		49.803
2	11.028	3103185	136550	V	50.197
总计		6181967	289637		100.000

Supplementary Figure 227. HPLC spectra for **3w**

Translation of Chinese Characters in HPLC spectra to English

描述：HPLC Condition　　检测器：Detector　峰号：Peak　　面积：Area

色谱图：HPLC Spectra　　高度：Height　　标记：Note　　总计：Total

峰表：Area　Percent Report　　　　　保留时间：Remaining Time

描述　　: AS-H, n-Hex/iPrOH = 98/2, 1.0 mL/min, 220 nm

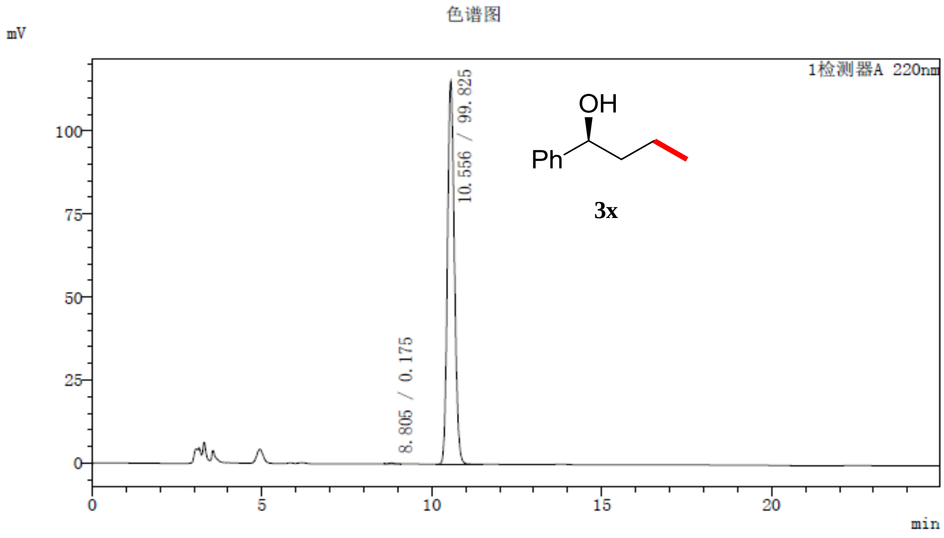

峰表

检测器A 220nm

峰号	保留时间	面积	高度	标记	面积%
1	8.805	2866	255		0.175
2	10.556	1639089	115395		99.825
总计		1641954	115650		100.000

描述　　: AS-H, n-Hex/iPrOH = 98/2, 1.0 mL/min, 220 nm

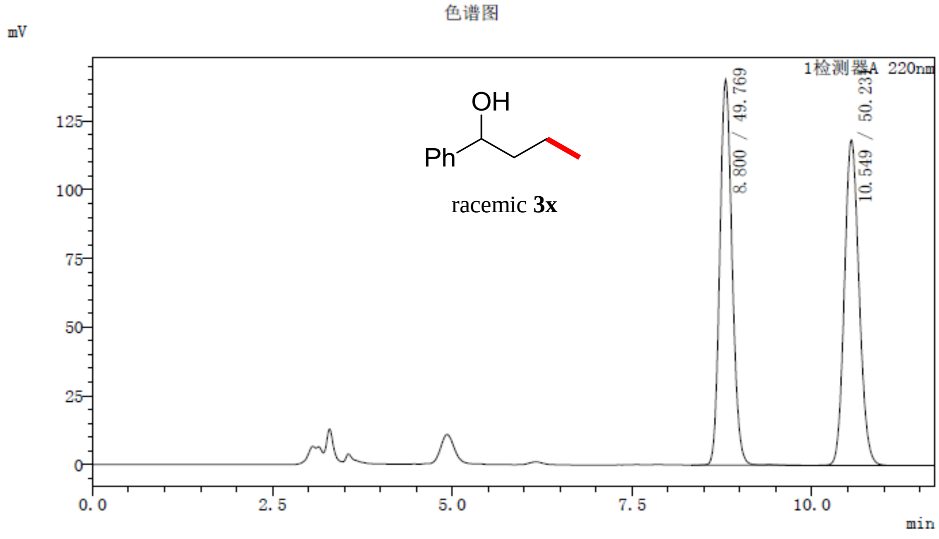

峰表

检测器A 220nm

峰号	保留时间	面积	高度	标记	面积%
1	8.800	1665567	140246	S	49.769
2	10.549	1681025	118453		50.231
总计		3346592	258699		100.000

Supplementary Figure 228. HPLC spectra for **3x**

Translation of Chinese Characters in HPLC spectra to English

描述：HPLC Condition　检测器：Detector　峰号：Peak　面积：Area

色谱图：HPLC Spectra　高度：Height　标记：Note　总计：Total

峰表：Area　Percent Report　保留时间：Remaining Time

描述　: OD-H, n-hexane:iPrOH = 98/2, 1.0 ml/min, 220 nm

色谱图
CX11159-P

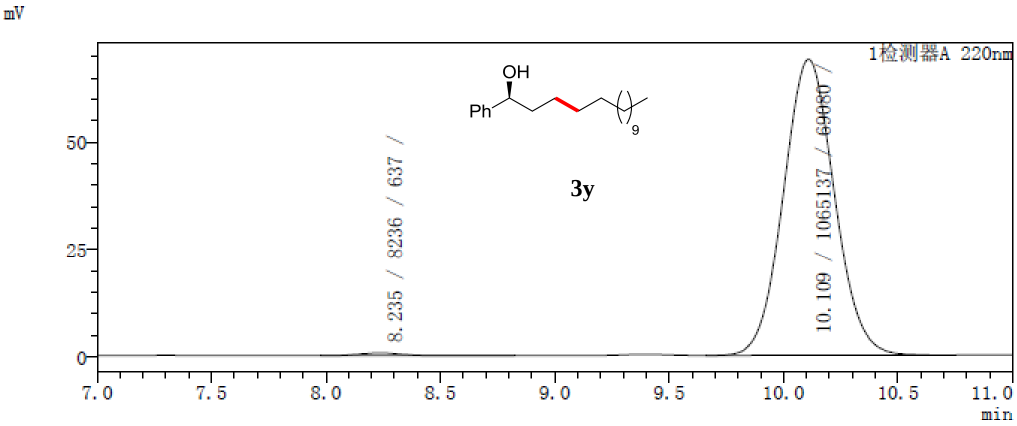

峰表

检测器A 220nm

峰号	保留时间	面积	高度	标记	面积%
1	8.235	8236	637		0.767
2	10.109	1065137	69080		99.233
总计		1073372	69718		100.000

描述　: OD-H, n-hexane:iPrOH = 98/2, 1.0 ml/min, 220 nm

色谱图
CX11159-P+-

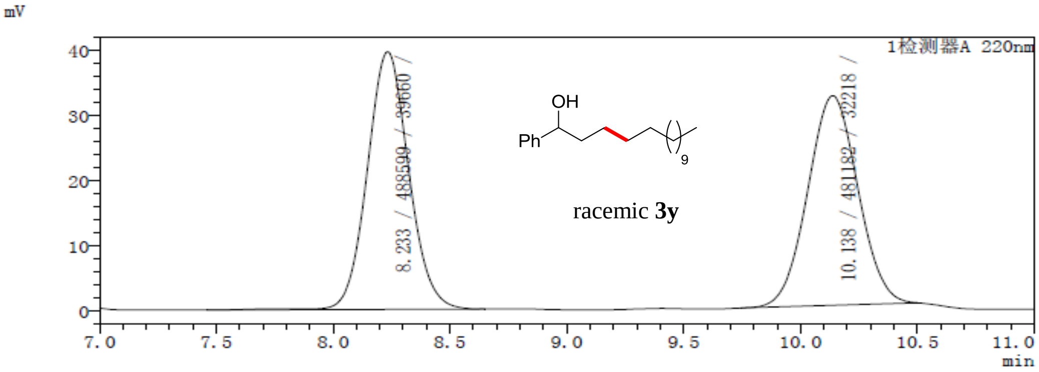

峰表

检测器A 220nm

峰号	保留时间	面积	高度	标记	面积%
1	8.233	488599	39660		50.382
2	10.138	481182	32218	M	49.618
总计		969781	71878		100.000

Supplementary Figure 229. HPLC spectra for **3y**

Translation of Chinese Characters in HPLC spectra to English

描述：HPLC Condition　检测器：Detector　峰号：Peak　面积：Area

色谱图：HPLC Spectra　高度：Height　标记：Note　总计：Total

峰表：Area Percent Report　保留时间：Remaining Time

描述 : AS-H, n-hexane:iPrOH = 98/2, 1.0 ml/min, 220 nm

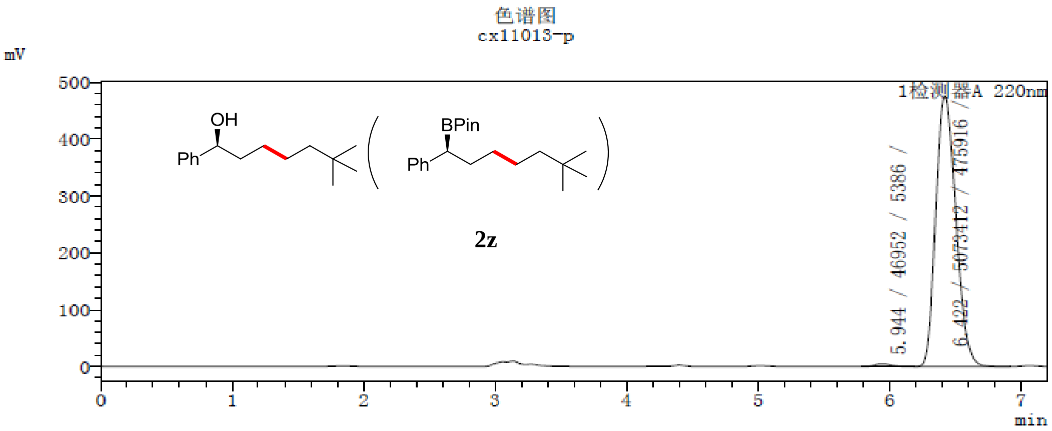

峰表

检测器A 220nm

峰号	保留时间	面积	高度	标记	面积%
1	5.944	46952	5386		0.917
2	6.422	5073412	475916		99.083
总计		5120364	481302		100.000

描述 : AS-H, n-hexane:iPrOH = 98/2, 1.0 ml/min, 220 nm

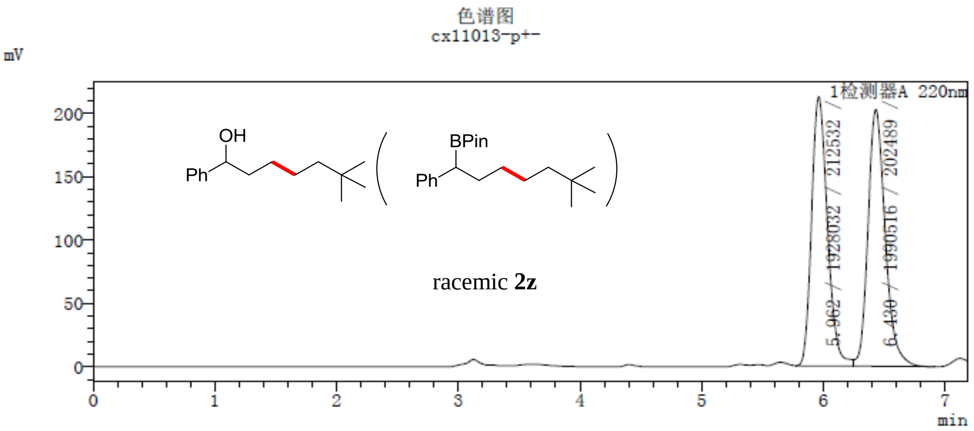

峰表

检测器A 220nm

峰号	保留时间	面积	高度	标记	面积%
1	5.962	1928032	212532		49.203
2	6.430	1990516	202489	V	50.797
总计		3918548	415021		100.000

Supplementary Figure 230. HPLC spectra for **2z**

Translation of Chinese Characters in HPLC spectra to English

描述：HPLC Condition　　检测器：Detector　峰号：Peak　　面积：Area

色谱图：HPLC Spectra　　高度：Height　　标记：Note　　总计：Total

峰表：Area　Percent Report　　　　　　保留时间：Remaining Time

描述　: AS-H, n-hexane:iPrOH = 98/2, 1.0 ml/min, 220 nm

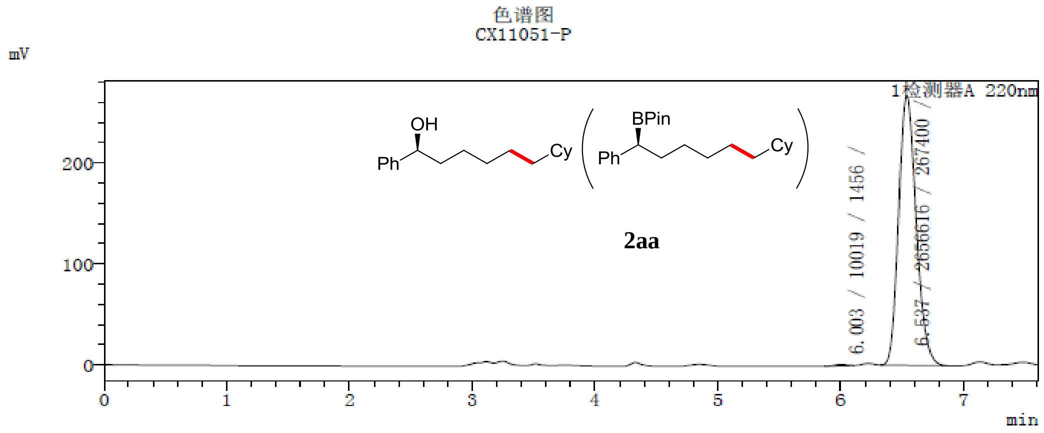

峰表

检测器A 220nm

峰号	保留时间	面积	高度	标记	面积%
1	6.003	10019	1456		0.376
2	6.537	2656616	267400		99.624
总计		2666635	268856		100.000

描述　: AS-H, n-hexane:iPrOH = 98/2, 1.0 ml/min, 220 nm

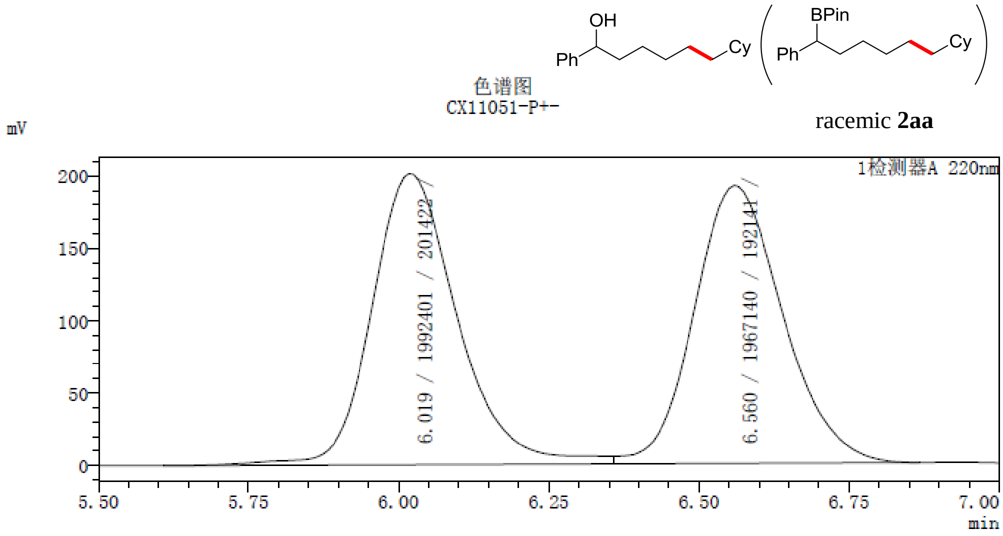

峰表

检测器A 220nm

峰号	保留时间	面积	高度	标记	面积%
1	6.019	1992401	201422		50.319
2	6.560	1967140	192141	V	49.681
总计		3959541	393562		100.000

Supplementary Figure 231. HPLC spectra for **2aa**

Translation of Chinese Characters in HPLC spectra to English

描述：HPLC Condition　检测器：Detector　峰号：Peak　面积：Area
色谱图：HPLC Spectra　高度：Height　标记：Note　总计：Total
峰表：Area　Percent Report　保留时间：Remaining Time

描述 : OD-H, n-hexane:iPrOH = 98/2, 1.0 ml/min, 220 nm

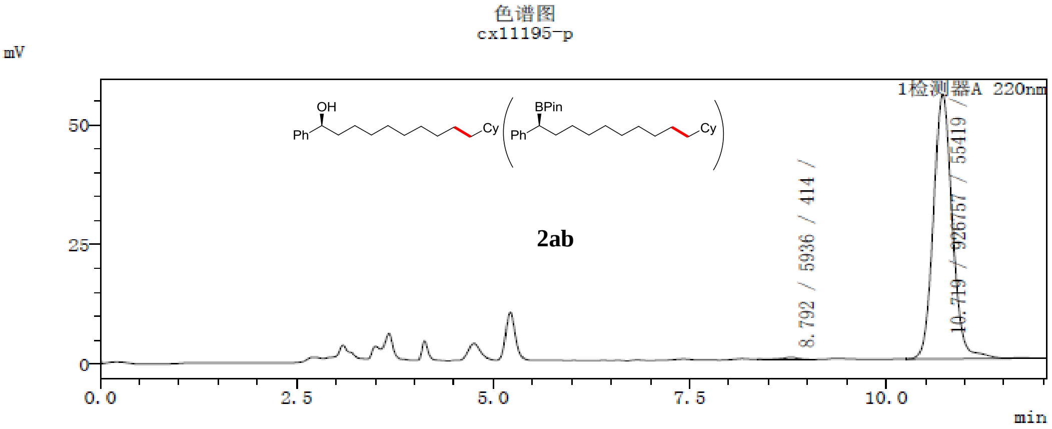

峰表

检测器A 220nm

峰号	保留时间	面积	高度	标记	面积%
1	8.792	5936	414		0.636
2	10.719	926757	55419	SV	99.364
总计		932693	55833		100.000

描述 : OD-H, n-hexane:iPrOH = 98/2, 1.0 ml/min, 220 nm

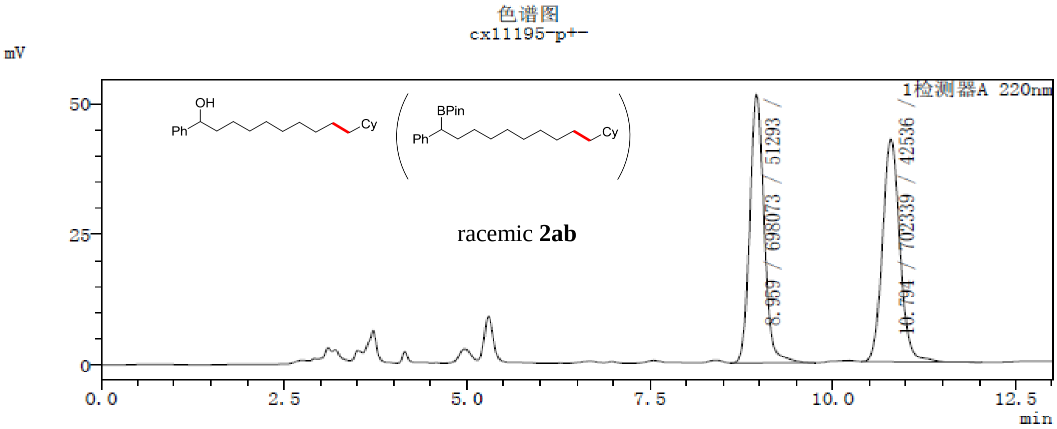

峰表

检测器A 220nm

峰号	保留时间	面积	高度	标记	面积%
1	8.959	698073	51293		49.848
2	10.794	702339	42536	S	50.152
总计		1400413	93829		100.000

Supplementary Figure 232. HPLC spectra for **2ab**

Translation of Chinese Characters in HPLC spectra to English

描述：HPLC Condition	检测器：Detector	峰号：Peak	面积：Area
色谱图：HPLC Spectra	高度：Height	标记：Note	总计：Total
峰表：Area Percent Report		保留时间：Remaining Time	

描述 : AS-H, n-Hex/iPrOH = 99.1/0.9, 1.0 mL/min, 220 nm

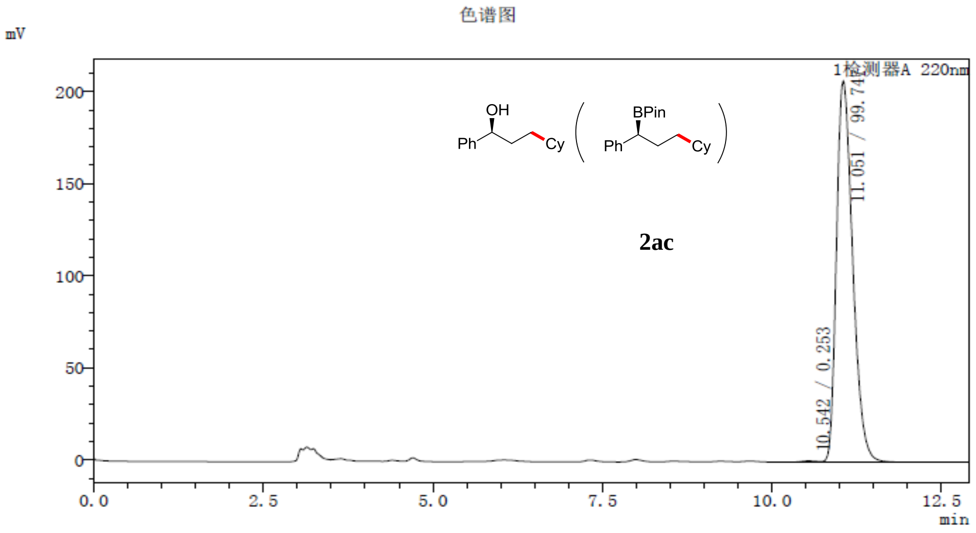

峰表

检测器A 220nm

峰号	保留时间	面积	高度	标记	面积%
1	10.542	8723	641		0.253
2	11.051	3436872	207014	V	99.747
总计		3445595	207655		100.000

描述 : AS-H, n-Hex/iPrOH = 99.1/0.9, 1.0 mL/min, 220 nm

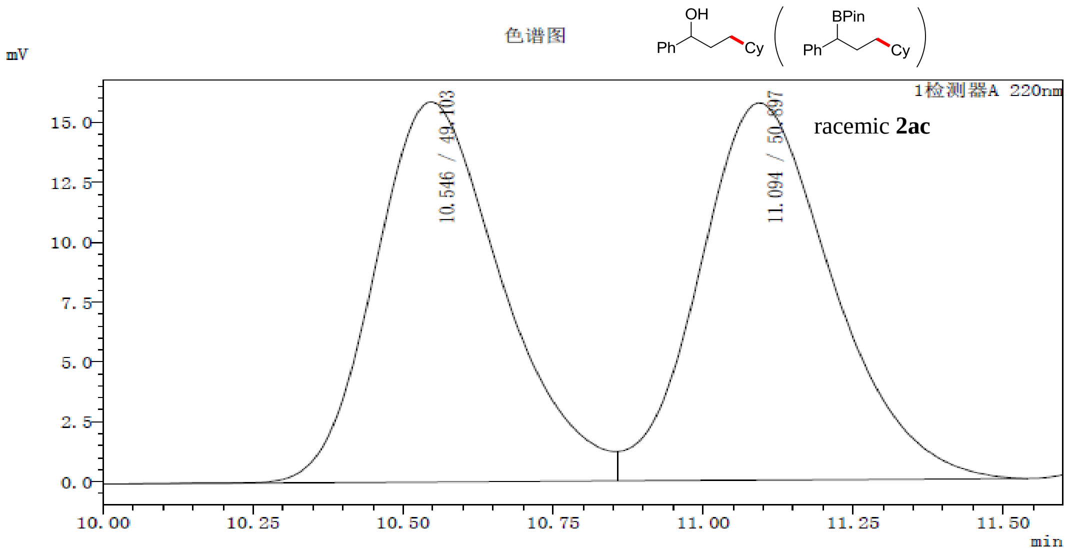

峰表

检测器A 220nm

峰号	保留时间	面积	高度	标记	面积%
1	10.546	234621	15877		49.103
2	11.094	243196	15755	V	50.897
总计		477817	31632		100.000

Supplementary Figure 233. HPLC spectra for **2ac**

Translation of Chinese Characters in HPLC spectra to English

描述：HPLC Condition　检测器：Detector　峰号：Peak　面积：Area

色谱图：HPLC Spectra　高度：Height 标记：Note 总计：Total

峰表：Area　Percent Report 保留时间：Remaining Time

描述　: AS-H, n-hexane/iPrOH = 98/2, 1.0 mL/min, 220 nm

色谱图

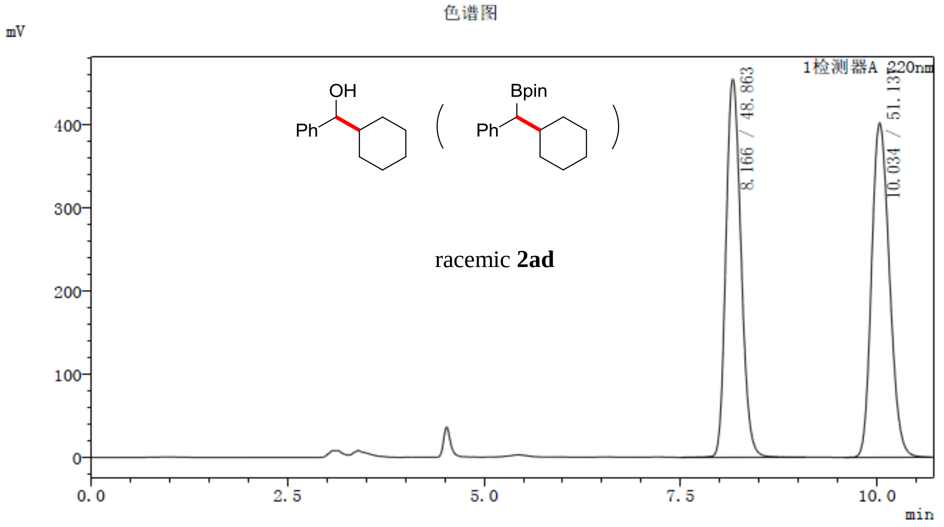

峰表

检测器A 220nm

峰号	保留时间	面积	高度	标记	面积%
1	8.166	6068979	455798		48.863
2	10.034	6351493	401952		51.137
总计		12420473	857750		100.000

描述　: AS-H, n-hexane/iPrOH = 98/2, 1.0 mL/min, 220 nm

色谱图

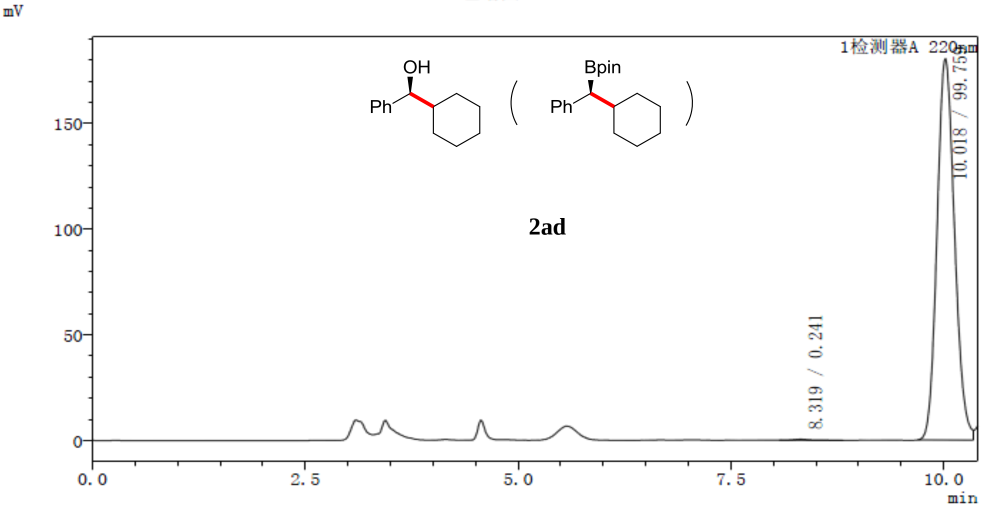

峰表

检测器A 220nm

峰号	保留时间	面积	高度	标记	面积%
1	8.319	6225	468		0.241
2	10.018	2576306	180554	M	99.759
总计		2582531	181022		100.000

Supplementary Figure 234. HPLC spectra for **2ad**

Translation of Chinese Characters in HPLC spectra to English

描述：HPLC Condition　　检测器：Detector　　峰号：Peak　　面积：Area

色谱图：HPLC Spectra　　高度：Height　　标记：Note　　总计：Total

峰表：Area　Percent Report　　保留时间：Remaining Time

描述　　: Oj-H, n-hexane:iPrOH = 98/2, 1.0　ml/min, 220 nm

色谱图
cx12027-p5

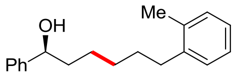

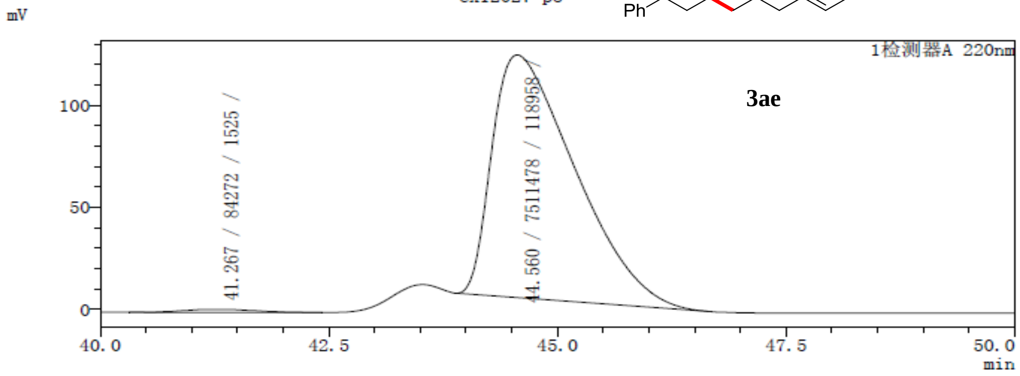

峰表

检测器A 220nm

峰号	保留时间	面积	高度	标记	面积%
1	41.267	84272	1525		1.109
2	44.560	7511478	118958		98.891
总计		7595750	120483		100.000

描述　　: Oj-H, n-hexane:iPrOH = 98/2, 1.0　ml/min, 220 nm

色谱图
cx12027-p4+-

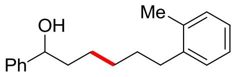

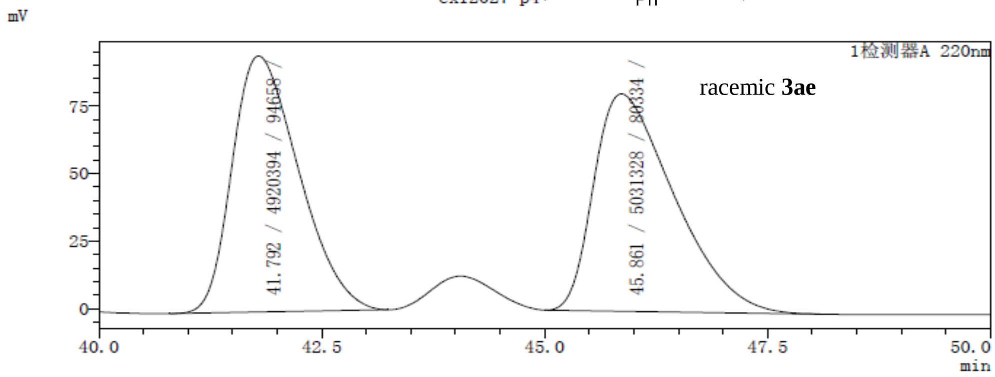

峰表

检测器A 220nm

峰号	保留时间	面积	高度	标记	面积%
1	41.792	4920394	94658		49.443
2	45.861	5031328	80334		50.557
总计		9951722	174993		100.000

Supplementary Figure 235. HPLC spectra for **3ac**

Translation of Chinese Characters in HPLC spectra to English

描述：HPLC Condition　　检测器：Detector　　峰号：Peak　　面积：Area

色谱图：HPLC Spectra　　高度：Height　　标记：Note　　总计：Total

峰表：Area　Percent Report　　保留时间：Remaining Time

描述 : AS-H, n-hexane:iPrOH = 98/2, 1.0 ml/min, 220nm

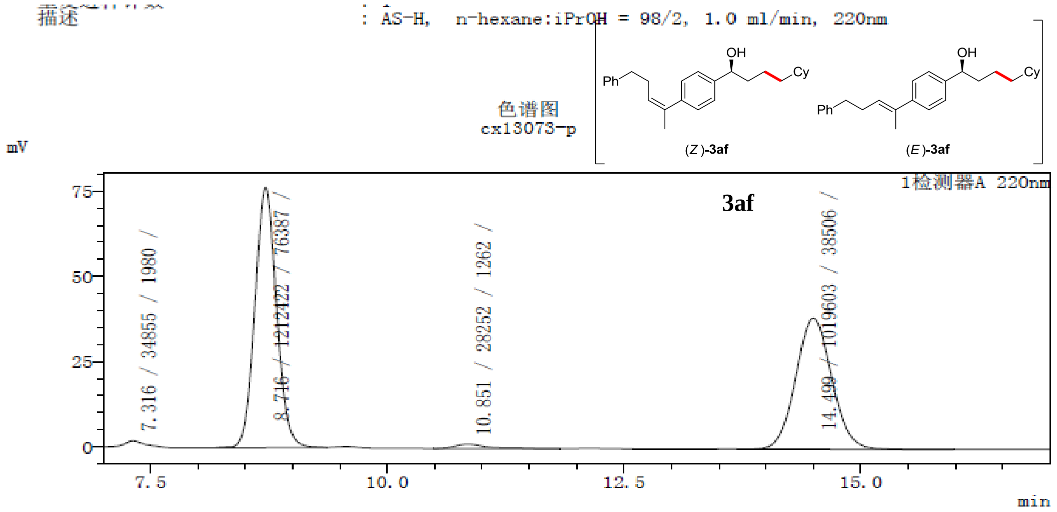

峰表

检测器A 220nm

峰号	保留时间	面积	高度	标记	面积%
1	7.316	34855	1980		1.519
2	8.716	1212422	76387		52.826
3	10.851	28252	1262		1.231
4	14.499	1019603	38506		44.425
总计		2295131	118135		100.000

描述 : AS-H, n-hexane:iPrOH = 98/2, 1.0 ml/min, 220nm

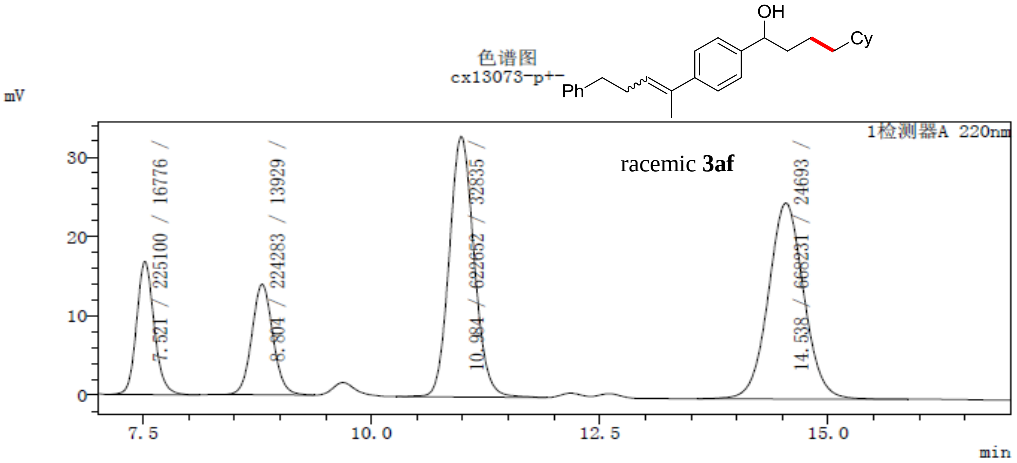

峰表

检测器A 220nm

峰号	保留时间	面积	高度	标记	面积%
1	7.521	225100	16776		12.935
2	8.804	224283	13929		12.888
3	10.984	622652	32835		35.779
4	14.538	668231	24693		38.398
总计		1740266	88232		100.000

Supplementary Figure 236. HPLC spectra for **3af**

Translation of Chinese Characters in HPLC spectra to English

描述：HPLC Condition　　检测器：Detector　　峰号：Peak　　面积：Area

色谱图：HPLC Spectra　　高度：Height　　标记：Note　　总计：Total

峰表：Area　Percent Report　　保留时间：Remaining Time

[illegible]

描述　　: AD-H, n-Hex/iPrOH = 95/5, 1.0 mL/min, 220 nm

色谱图

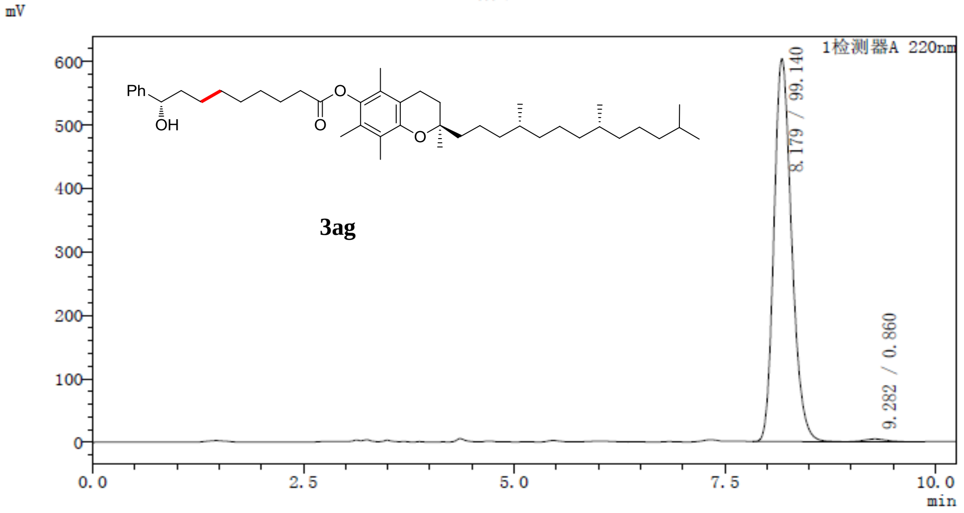

峰表

检测器A 220nm

峰号	保留时间	面积	高度	标记	面积%
1	8.179	8831789	603537		99.140
2	9.282	76590	4311	V	0.860
总计		8908379	607848		100.000

[illegible]

描述　　: AD-H, n-Hex/iPrOH = 95/5, 1.0 mL/min, 220 nm

色谱图

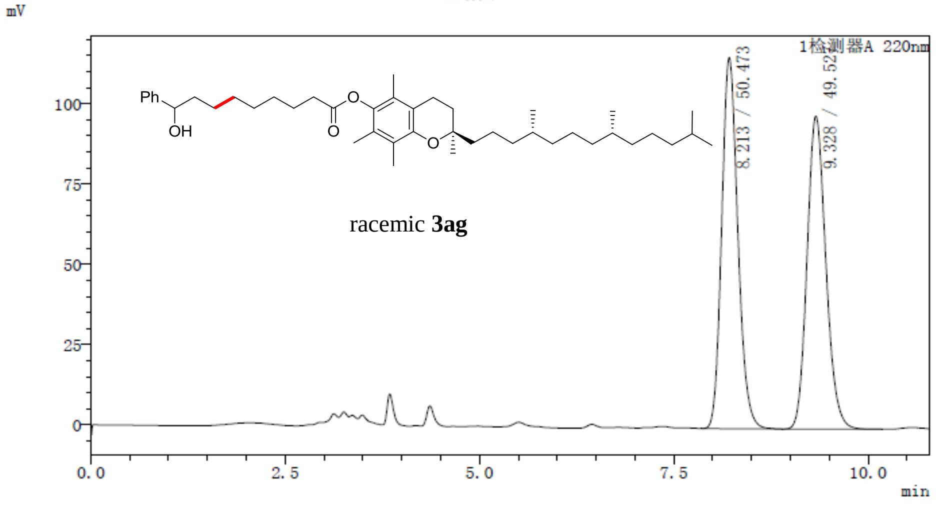

峰表

检测器A 220nm

峰号	保留时间	面积	高度	标记	面积%
1	8.213	1670874	115541		50.473
2	9.328	1639582	97449		49.527
总计		3310456	212990		100.000

Supplementary Figure 237. HPLC spectra for **3ag**

Translation of Chinese Characters in HPLC spectra to English

描述：HPLC Condition　　检测器：Detector　　峰号：Peak　　面积：Area

色谱图：HPLC Spectra　　高度：Height　　标记：Note　　总计：Total

峰表：Area　Percent Report　　保留时间：Remaining Time

描述　　: AS-H, n-hexane:iPrOH = 98/2, 1.0 ml/min, 220 nm

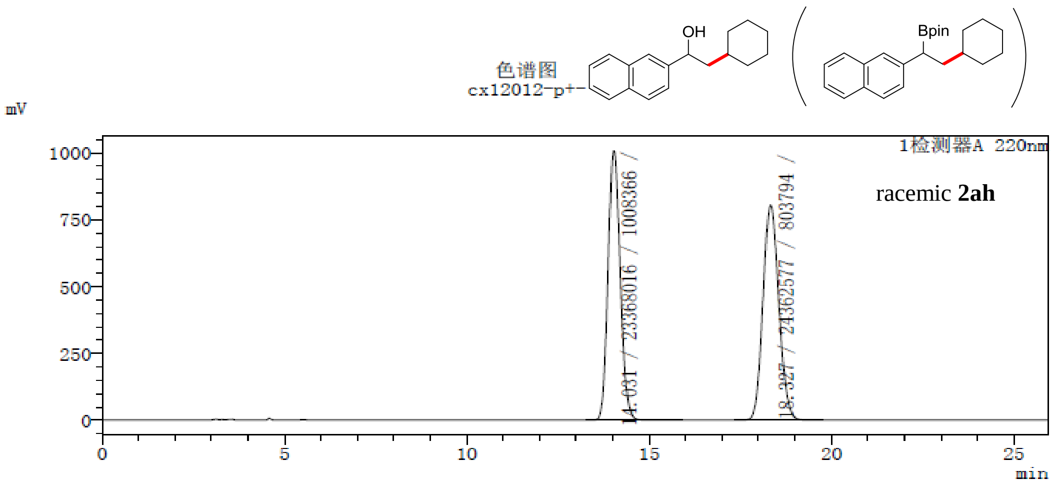

峰表

检测器A 220nm

峰号	保留时间	面积	高度	标记	面积%
1	14.031	23368016	1008366		48.958
2	18.327	24362577	803794		51.042
总计		47730593	1812160		100.000

描述　　: AS-H, n-hexane:iPrOH = 98/2, 1.0 ml/min, 220 nm

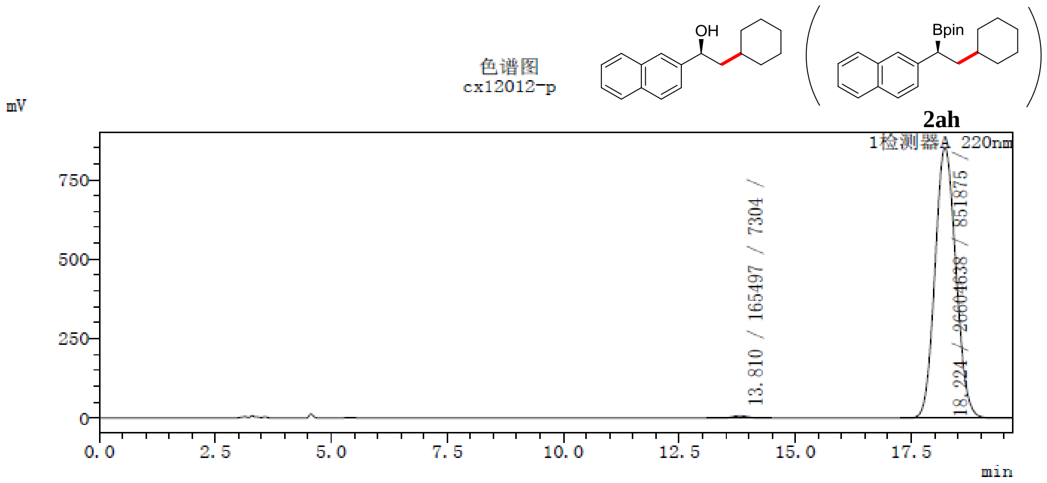

峰表

检测器A 220nm

峰号	保留时间	面积	高度	标记	面积%
1	13.810	165497	7304		0.618
2	18.224	26604638	851875		99.382
总计		26770135	859179		100.000

Supplementary Figure 238. HPLC spectra for **2ah**

Translation of Chinese Characters in HPLC spectra to Englis

描述：HPLC Condition　　检测器：Detector　　峰号：Peak　　面积：Area

色谱图：HPLC Spectra　　高度：Height　　标记：Note　　总计：Total

峰表：Area　Percent Report　　保留时间：Remaining Time

描述　　: OJ-H, n-hexane:iPrOH = 99/1, 0.5 ml/min, 220 nm

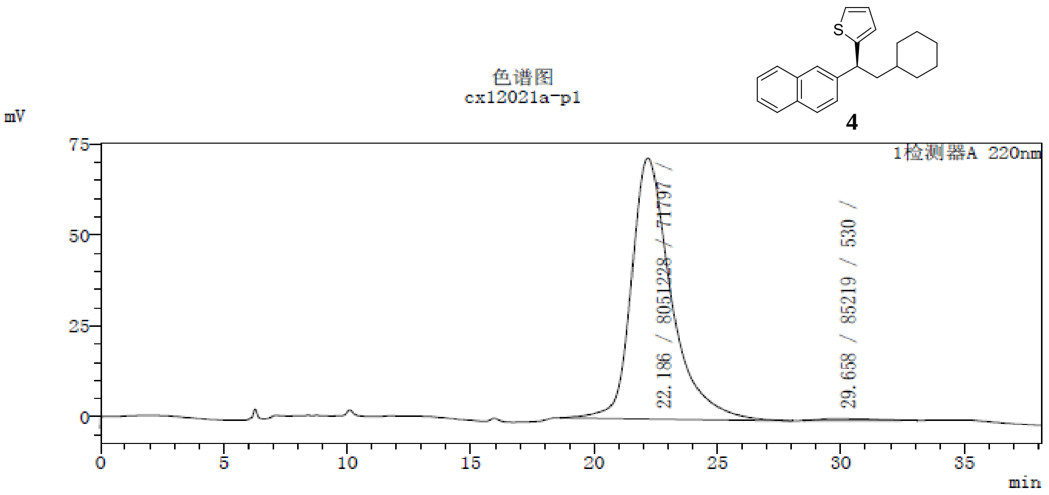

峰表

检测器A 220nm

峰号	保留时间	面积	高度	标记	面积%
1	22.186	8051228	71797		98.953
2	29.658	85219	530	M	1.047
总计		8136447	72327		100.000

描述　　: OJ-H, n-hexane:iPrOH = 99/1, 0.5 ml/min, 220 nm

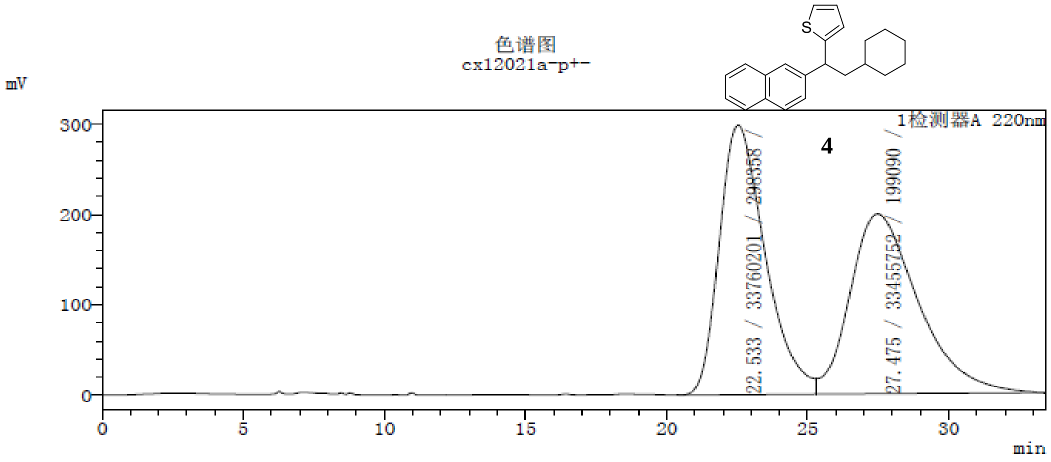

峰表

检测器A 220nm

峰号	保留时间	面积	高度	标记	面积%
1	22.533	33760201	298358		50.226
2	27.475	33455752	199090	V	49.774
总计		67215953	497448		100.000

Supplementary Figure 239. HPLC spectra for **4**

Translation of Chinese Characters in HPLC spectra to English

描述：HPLC Condition　　检测器：Detector　　峰号：Peak　　面积：Area

色谱图：HPLC Spectra　　高度：Height　　标记：Note　　总计：Total

峰表：Area　Percent Report　　保留时间：Remaining Time

描述　　: OJ-H, n-hexane:iPrOH = 99/1, 0.5 ml/min, 220 nm

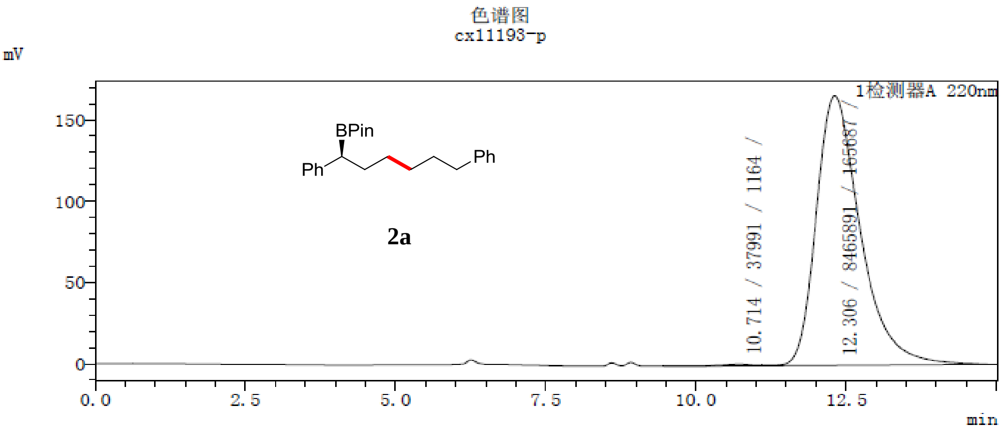

峰表

检测器A 220nm

峰号	保留时间	面积	高度	标记	面积%
1	10.714	37991	1164		0.447
2	12.306	8465891	165687	V	99.553
总计		8503882	166851		100.000

描述　　: OJ-H, n-hexane:iPrOH = 99/1, 0.5 ml/min, 220 nm

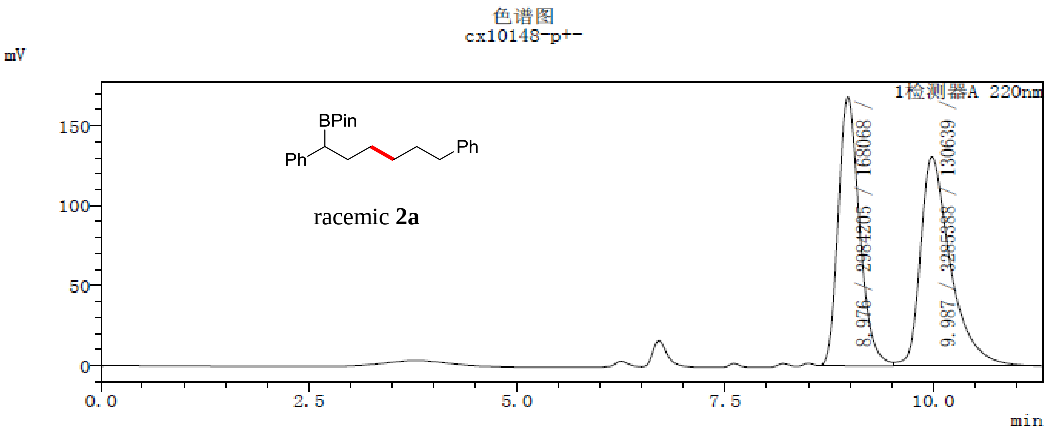

峰表

检测器A 220nm

峰号	保留时间	面积	高度	标记	面积%
1	8.976	2984205	168068		47.598
2	9.987	3285388	130639	V	52.402
总计		6269593	298707		100.000

Supplementary Figure 240. HPLC spectra for **2a** (gram scale raction)

Translation of Chinese Characters in HPLC spectra to English

描述：HPLC Condition　检测器：Detector　峰号：Peak　面积：Area

色谱图：HPLC Spectra　高度：Height　标记：Note　总计：Total

峰表：Area　Percent Report　保留时间：Remaining Time

描述　: AD-H, n-Hexane/iPrOH = 98/2, 1.0 mL/min, 220 nm

色谱图

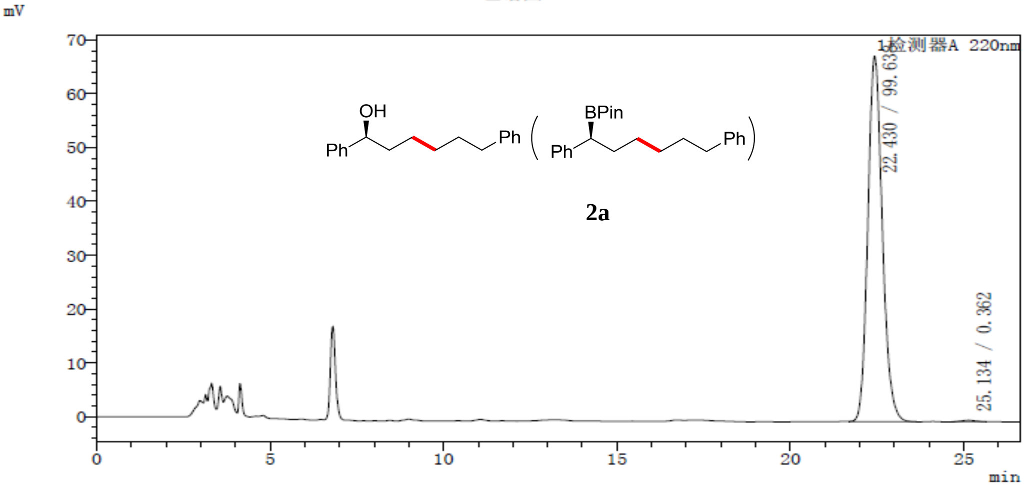

峰表

检测器A 220nm

峰号	保留时间	面积	高度	标记	面积%
1	22.430	1974890	67896	V	99.638
2	25.134	7169	261		0.362
总计		1982059	68157		100.000

描述　: AD-H, n-Hexane/iPrOH = 98/2, 1.0 mL/min, 220 nm

色谱图

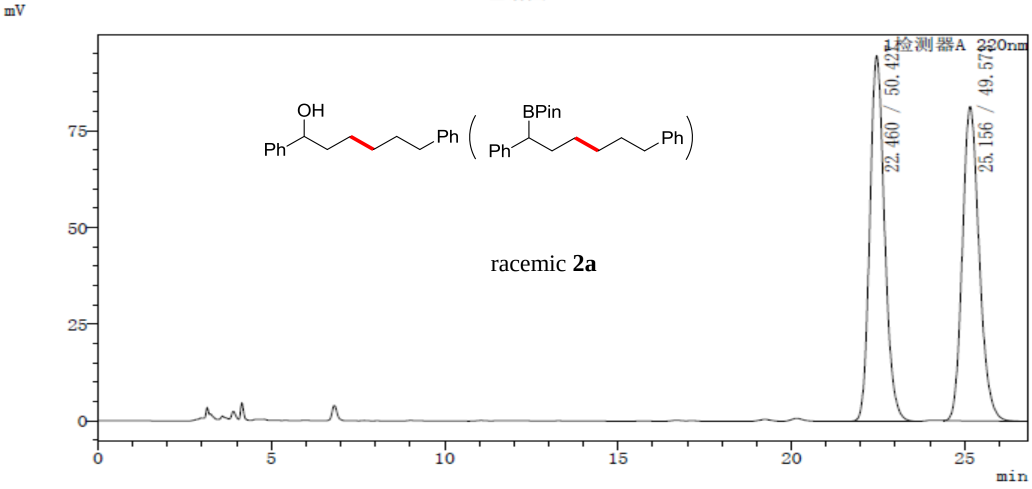

峰表

检测器A 220nm

峰号	保留时间	面积	高度	标记	面积%
1	22.460	2798402	94561		50.427
2	25.156	2751021	81383		49.573
总计		5549423	175943		100.000

Supplementary Figure 241. HPLC spectra for **2a** (utilization of alkene isomers)

Translation of Chinese Characters in HPLC spectra to English

描述：HPLC Condition　　检测器：Detector　峰号：Peak　　面积：Area

色谱图：HPLC Spectra　　高度：Height　　标记：Note　　总计：Total

峰表：Area　Percent Report　　　　　　保留时间：Remaining Time

描述 [illegible] : AS-H, n-hexane:iPrOH = 98/2, 1.0ml/min, 220nm

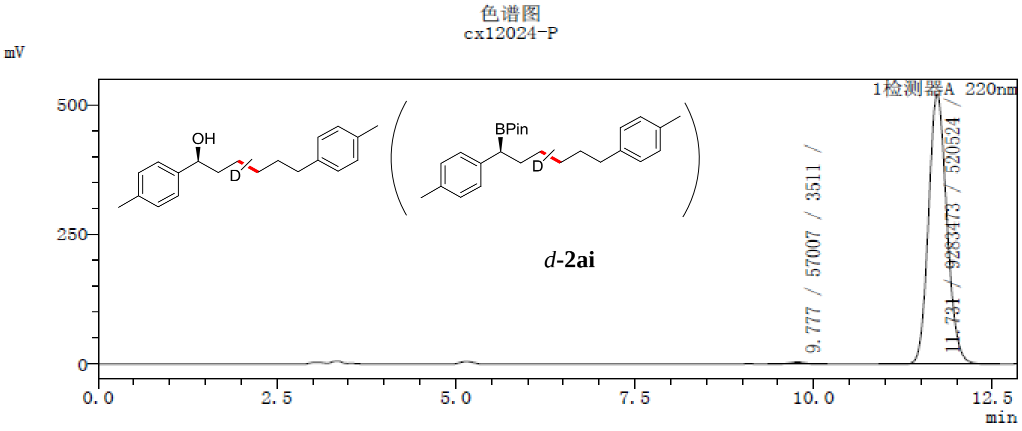

峰表

检测器A 220nm

峰号	保留时间	面积	高度	标记	面积%
1	9.777	57007	3511		0.610
2	11.731	9283473	520524		99.390
总计		9340480	524035		100.000

描述 [illegible] : AS-H, n-hexane:iPrOH = 98/2, 1.0ml/min, 220nm

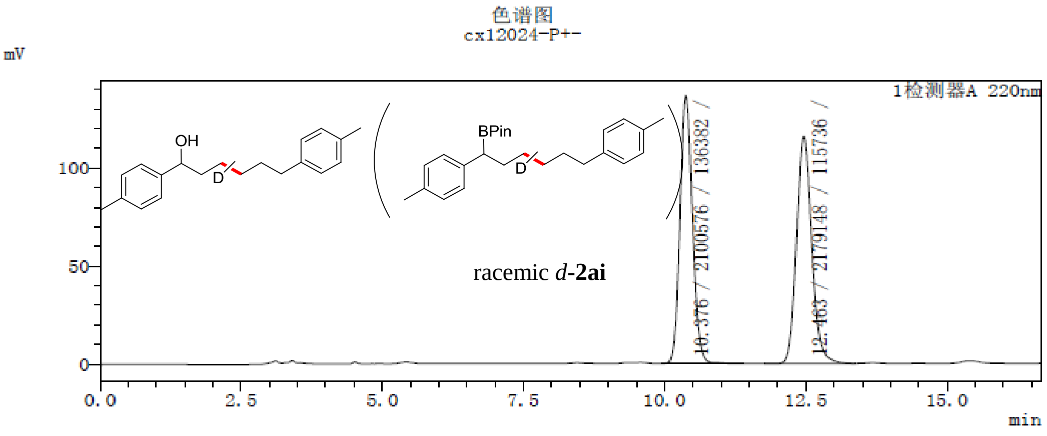

峰表

检测器A 220nm

峰号	保留时间	面积	高度	标记	面积%
1	10.376	2100576	136382		49.082
2	12.463	2179148	115736		50.918
总计		4279724	252118		100.000

Supplementary Figure 242. HPLC spectra for ***d*-2ai**

Translation of Chinese Characters in HPLC spectra to English
描述：HPLC Condition　检测器：Detector　峰号：Peak　面积：Area
色谱图：HPLC Spectra　高度：Height 标记：Note 总计：Total
峰表：Area　Percent Report 保留时间：Remaining Time

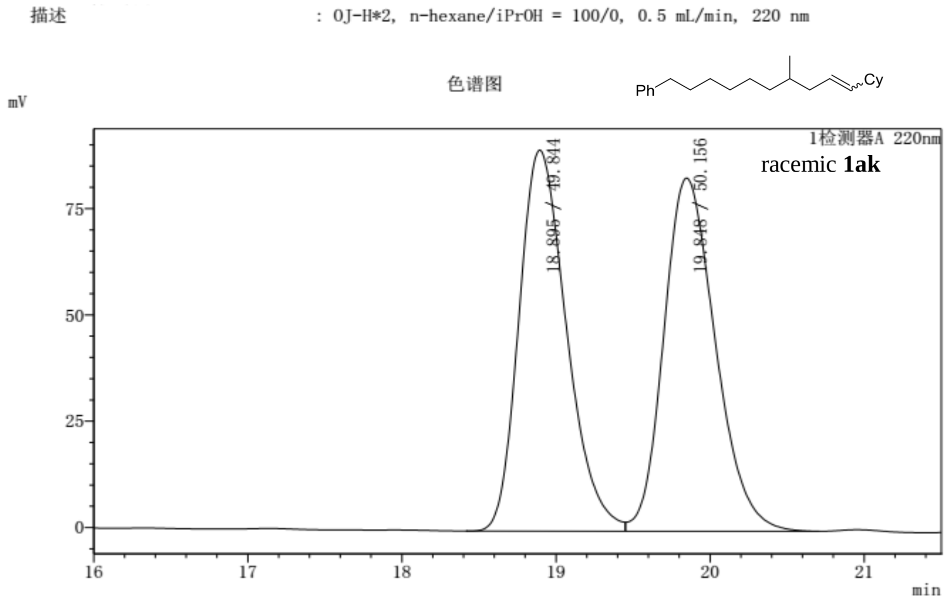

峰表
检测器A 220nm

峰号	保留时间	面积	高度	标记	面积%
1	18.895	1921522	89585		49.844
2	19.848	1933524	83060	V	50.156
总计		3855046	172645		100.000

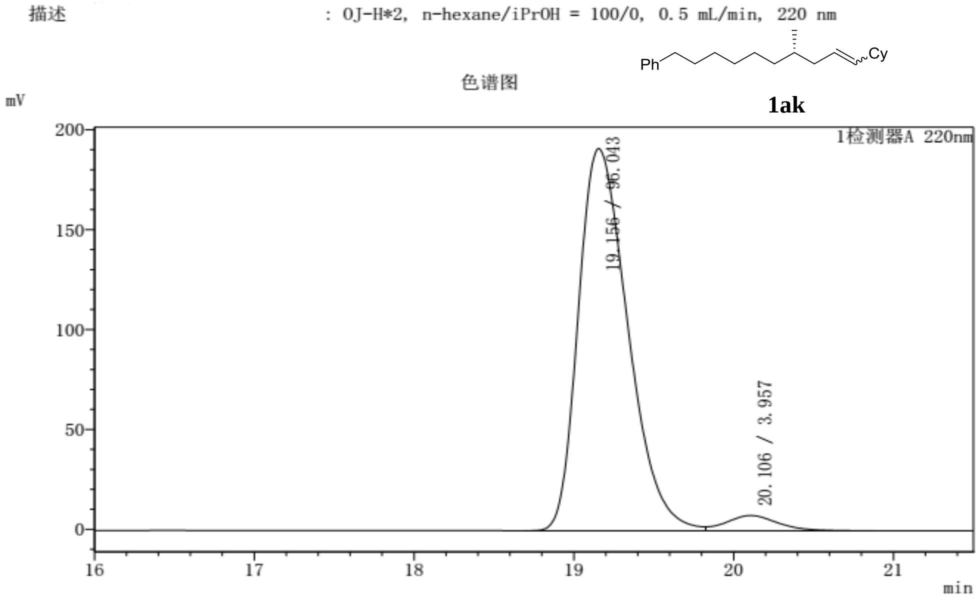

峰表
检测器A 220nm

峰号	保留时间	面积	高度	标记	面积%
1	19.156	4111879	191295		96.043
2	20.106	169405	7624	V	3.957
总计		4281284	198919		100.000

Supplementary Figure 243. HPLC spectra for **1ak**

Translation of Chinese Characters in HPLC spectra to English

描述：HPLC Condition　检测器：Detector　峰号：Peak　面积：Area

色谱图：HPLC Spectra　高度：Height 标记：Note 总计：Total

峰表：Area　Percent Report 保留时间：Remaining Time

描述　　: OD-H , n-hexane/iPrOH = 98/2, 1.0 mL/min, 220 nm

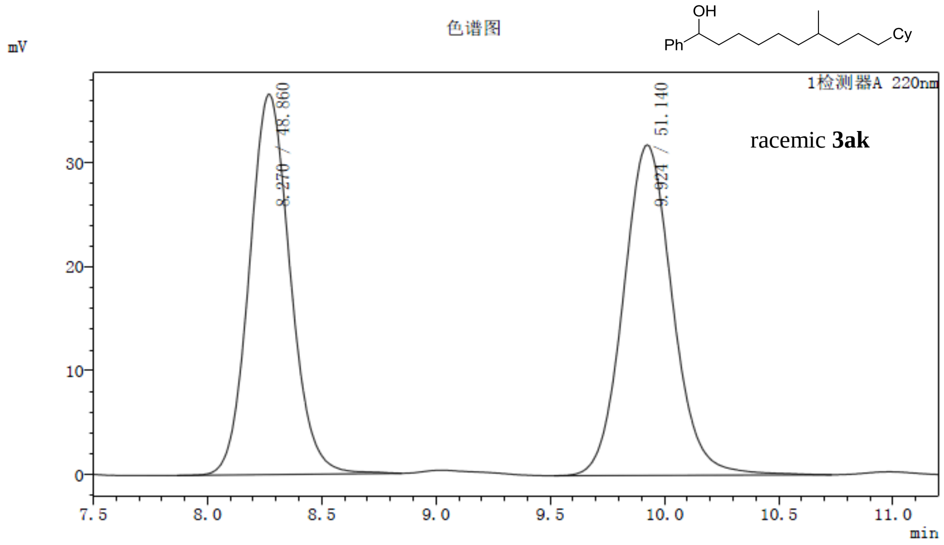

峰表

检测器A 220nm

峰号	保留时间	面积	高度	标记	面积%
1	8.270	455438	36611		48.860
2	9.924	476687	31806		51.140
总计		932124	68417		100.000

描述　　: OD-H , n-hexane/iPrOH = 98/2, 1.0 mL/min, 220 nm

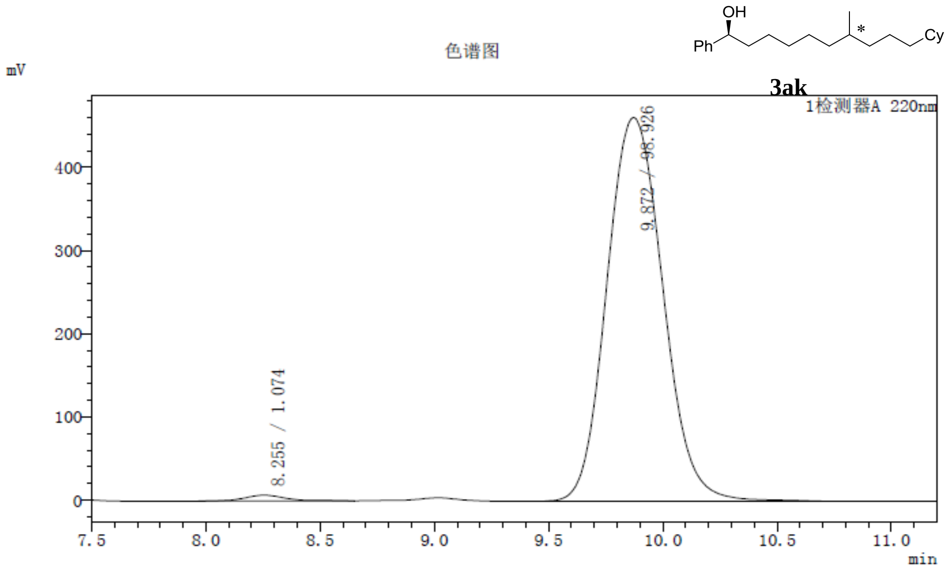

峰表

检测器A 220nm

峰号	保留时间	面积	高度	标记	面积%
1	8.255	85386	6897		1.074
2	9.872	7867627	460977		98.926
总计		7953013	467874		100.000

Supplementary Figure 244. HPLC spectra for **3ak**

Translation of Chinese Characters in HPLC spectra to English

描述：HPLC Condition　检测器：Detector　峰号：Peak　面积：Area

色谱图：HPLC Spectra　高度：Height 标记：Note 总计：Total

峰表：Area　Percent Report 保留时间：Remaining Time

描述　　: OJ-H*3 , n-hexane/iPrOH = 100/0, 0.25 mL/min, 220 nm

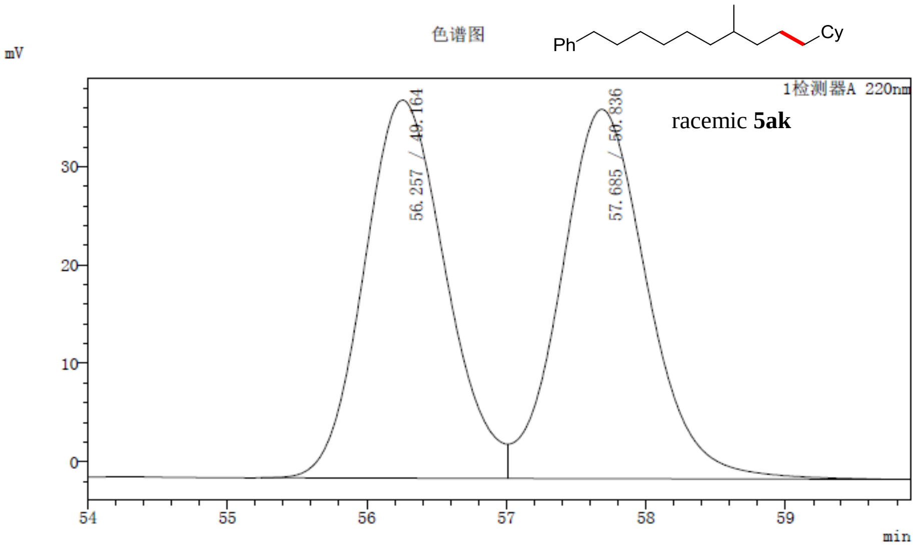

峰表

检测器A 220nm

峰号	保留时间	面积	高度	标记	面积%
1	56.257	1560687	38444		49.164
2	57.685	1613732	37560	V	50.836
总计		3174420	76004		100.000

描述　　: OJ-H*3 , n-hexane/iPrOH = 100/0, 0.25 mL/min, 220 nm

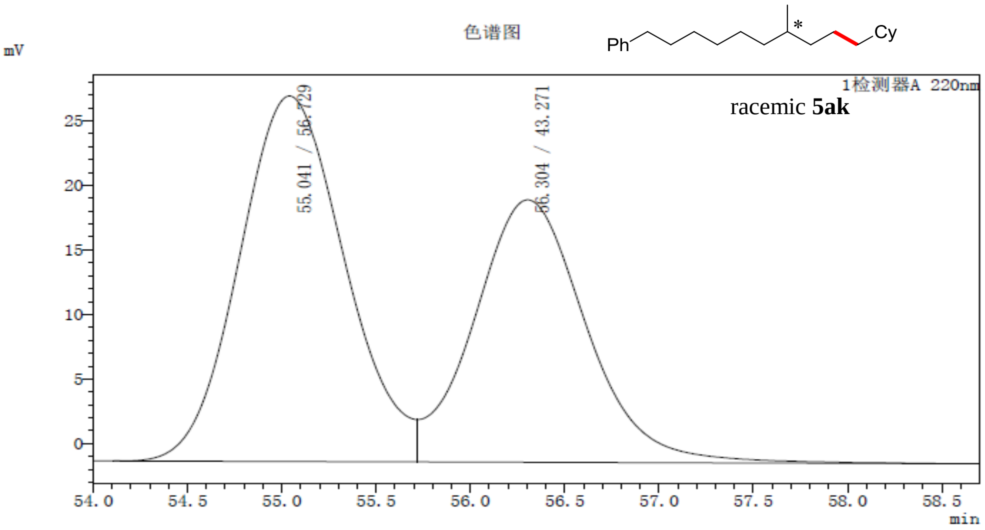

峰表

检测器A 220nm

峰号	保留时间	面积	高度	标记	面积%
1	55.041	1099414	28305		56.729
2	56.304	838605	20318	V	43.271
总计		1938019	48623		100.000

Supplementary Figure 245. HPLC spectra for **5ak**

Supplementary Tables

Supplementary Table 1 Optimization studies on different ligands, solvent.

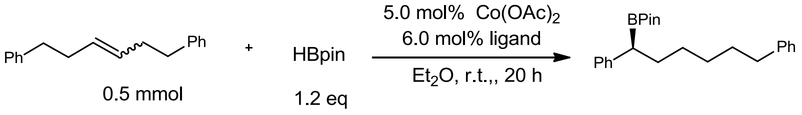

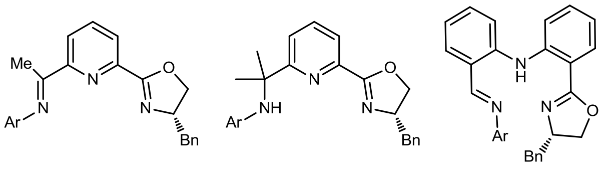

L1 L2 L3

Ar = 2,6-di*i*PrC$_6$H$_3$

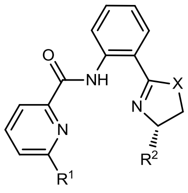

L4: R^1 = H, R^2 = Bn, X = O
L5: R^1 = Me, R^2 = Bn, X = O
L6: R^1 = Me, R^2 = Bn, X = NPh
L7: R^1 = Me, R^2 = *i*Pr, X = NPh
L8: R^1 = Me, R^2 = *t*Bu, X = NPh
L9: R^1 = Me, R^2 = Ph, X = NPh

Lsa: R^1 = H, R^2 = Ph, X = O
Lsb: R^1 = H, R^2 = indenyl, X = O
Lsc: R^1 = H, R^2 = *i*Pr, X = O
Lsd: R^1 = H, R^2 = *t*Bu, X = O
Lse: R^1 = Me, R^2 = Ph, X = O
Lsf: R^1 = Me, R^2 = indenyl, X = O
Lsg: R^1 = Me, R^2 = *i*Pr, X = O
Lsh: R^1 = Me, R^2 = *t*Bu, X = O

Entry	Ligand	Solvent	Yield/%[a]	*Ee*/%
1	**Lsa**	Et_2O	81	18.6
2	**Lsb**	Et_2O	96	32.4
3	**Lsc**	Et_2O	92	33.2
4	**Lsd**	Et_2O	88	31.0
5	**Lse**	Et_2O	98	85.8
6	**Lsf**	Et_2O	83	90.6
7	**Lsg**	Et_2O	71	89.6
8	**Lsh**	Et_2O	95	93.4
9	**L1**	Et_2O	99	<5
10	**L2**	Et_2O	19	61.8
11	**L3**	Et_2O	/	/
12	**L4**	Et_2O	75	22.6
13	**L5**	Et_2O	99	87.8
14	**L6**	Et_2O	54	95.8
15	**L7**	Et_2O	88	98.0

16	**L8**	Et_2O	96	99.6
17	**L9**	Et_2O	83	89.6
18	**L8**	MeCN	52	99.0
19	**L8**	DCM	53	99.2
20	**L8**	THF	84	99.6
21	**L8**	toluene	85	99.6
22	**L8**	dioxane	87	99.8

[a] Yield was determined by ^{1}H NMR by using TMSPh as internal standard.

Supplementary Table 2 Control experiments.

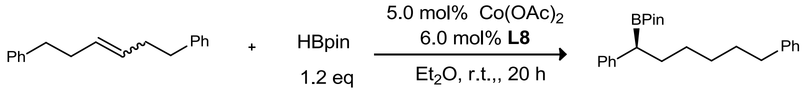

Entry	Changes from standard conditions	Yield[a]	*ee*
1	added 1.0 eq. H_2O	/	/
2	opened to air	/	/
3	no ligand	/	/
5	using $FeCl_2$ instead of $Co(OAc)_2$	/	/
6	using $Pd(OAc)_2$ instead of $Co(OAc)_2$	/	/
7	using $Rh_2(OAc)_2$ instead of $Co(OAc)_2$	/	/

[a] Yield was determined by ^{1}H NMR by using TMSPh as internal standard.

Supplementary Methods

General Information

Ether, tetrahydrofuran, 1,4-dioxane and toluene were distilled from sodium benzophenoneketyl prior to use and dichloromethane was distilled from CaH_2. Pinacolborane (HBpin) (97%) was purchased from TCI and used as received. $NaHBEt_3$ (1.0 M in THF) were purchased from Aldrich and used as received. $Co(OAc)_2$ (99%) were purchased from Alfa and used as received. The other commercial available chemicals were used as received. NMR spectra were recorded on a Bruker-400 instrument. ^{1}H NMR chemical shifts were referenced to tetramethylsilane signal (0

ppm),^{13}C NMR chemical shifts were referenced to the solvent resonance (77.00 ppm, $CDCl_3$). The following abbreviations (or combinations thereof) were used to explain multiplicities: s = singlet, d = doublet, t = triplet, q = quadruplet, m = multiplet, br = broad. IR spectra were recorded on a Perkin-Elmer Spectrum One FTIR spectrometer with diamond ATR accessory. High-resolution mass spectra (HRMS) were recorded on EI-TOF (electrospray ionization-time of flight). X-ray diffraction data was obtained on Gemini A Ultra.

Procedures for Preparation of Ligands

L1-L3 were prepared according to the methods reported by our group.[1-3] **S1-S5** were prepared according to the literature. [4]

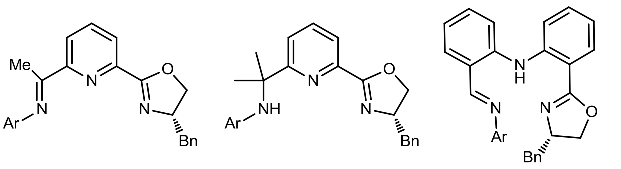

L1 **L2** **L3**

Ar = 2,6-di*i*PrC_6H_3

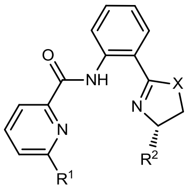

L4: R^1 = H, R^2 = Bn, X = O
L5: R^1 = Me, R^2 = Bn, X = O
L6: R^1 = Me, R^2 = Bn, X = NPh
L7: R^1 = Me, R^2 = *i*Pr, X = NPh
L8: R^1 = Me, R^2 = *t*Bu, X = NPh
L9: R^1 = Me, R^2 = Ph, X = NPh

Lsa: R^1 = H, R^2 = Ph, X = O
Lsb: R^1 = H, R^2 = indenyl, X = O
Lsc: R^1 = H, R^2 = *i*Pr, X = O
Lsd: R^1 = H, R^2 = *t*Bu, X = O
Lse: R^1 = Me, R^2 = Ph, X = O
Lsf: R^1 = Me, R^2 = indenyl, X = O
Lsg: R^1 = Me, R^2 = *i*Pr, X = O
Lsh: R^1 = Me, R^2 = *t*Bu, X = O

General procedure for the preparation of L4 –L5, Lsa-Lsh

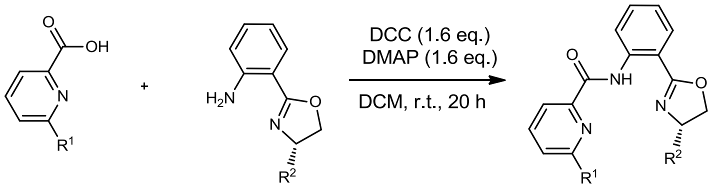

R^1 = H or Me

s1: R^2 = Bn
s2: R^2 = Ph
s3: R^2 = indenyl
s4: R^2 = *i*Pr
s5: R^2 = *t*Bu

L4-L5
Lsa-Lsh

A 50 mL oven-dried round-bottom flask was charged with amine **S1** (5.0 mmol), picolinic acid (8.0 mmol) and DCM (25 mL). After stirred at room temperature for 5 min, DCC (8.0 mmol) and DMAP (8 mmol) was added to this reaction mixture and stirred at room temperature for 24 h.

Then the reaction mixture was concentrated in vacuo and purified by column chromatography using PE/EA = 10/1 as the eluent to give the product **L4**.

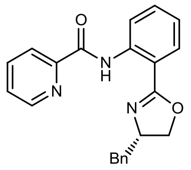

(*S*)-*N*-(2-(4-benzyl-4,5-dihydrooxazol-2-yl)phenyl)picolinamide (L4): Prepared according to the general procedure using picolinic acid (0.9618g, 7.8 mmol), **S1** (1.2550 g, 5.0 mmol), DCM (25 mL), DCC (1.6430 g, 8.0 mmol) and DMAP (0.9756 g, 8.0 mmol). After 24 h, the reaction mixture was concentrated in vacuo and purified by column chromatography using PE/EA = 10/1 as the eluent to afford **L4** (1.7209 g, 0.48 mmol, 97% yield) as a white solid. M.P.: 74.6-75.8 ^oC; IR (neat): 3060, 2925, 1681, 1642, 1581, 1528, 1448 cm^{-1}. Optical Rotation: $[\alpha]^{20}_D$ = +73.7 (c 1.0, $CHCl_3$). ^1H NMR (400 MHz, $CDCl_3$): δ13.78 (s, 1H), 9.03 (d, *J* = 8.0 Hz, 1H), 8.54 (d, *J* = 4.4 Hz, 1H), 8.29 (d, *J* = 7.6 Hz, 1H), 7.94-7.80 (m, 2H), 7.57-7.49 (m, 1H), 7.42 (dd, *J* = 7.2, 4.8 Hz, 1H), 7.32-7.19 (m, 5H), 7.13 (t, *J* = 7.2 Hz, 1H), 4.89-4.77 (m, 1H), 4.38 (t, *J* = 9.2 Hz, 1H), 4.11 (t, *J* = 8.0 Hz, 1H), 3.35 (dd, *J* = 14.0, 5.2 Hz, 1H), 2.92 (dd, *J* = 14.0, 8.0 Hz, 1H); ^{13}C NMR: (101 MHz, $CDCl_3$): δ164.1, 163.4, 151.0, 148.3, 139.5, 137.7, 137.2, 132.4, 129.30, 129.28, 128.5, 126.5, 126.0, 122.75, 122.66, 120.2, 114.6, 70.5, 67.9, 41.7. HRMS (ESI) calculated for $[M+Na]^+[C_{22}H_{19}N_3O_2Na]^+$ requires m/z 380.1375, found m/z 380.1377.

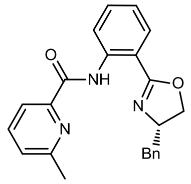

(*S*)-*N*-(2-(4-benzyl-4,5-dihydrooxazol-2-yl)phenyl)-6-methylpicolinamide (L5): Prepared according to the general procedure using 6-methylpicolinic acid (0.6582 g, 4.8 mmol), **S1** (0.7568 g, 3.0 mmol), DCM (15 mL), DCC (0.9918 g, 4.8 mmol) and DMAP (0.5816 g, 4.8 mmol). After 24 h, the reaction mixture was concentrated in vacuo and purified by column chromatography using PE/EA = 10/1 as the eluent to afford **L5** (0.9895 g, 2.7 mmol, 89% yield) as a white solid. M.P.: 101.8-103.5 ^oC; IR (neat): 3025, 2895, 1678, 1641, 1582, 1522, 1445 cm^{-1}. Optical Rotation: $[\alpha]^{20}_D$ = +116.3 (c 1.09, $CHCl_3$). ^1H NMR (400 MHz, $CDCl_3$):δ 13.71 (s, 1H), 9.05 (d, *J* = 8.4 Hz, 1H), 8.12 (d, *J* = 7.6 Hz, 1H), 7.87 (d, *J* = 7.6 Hz, 1H), 7.78 (t, *J* = 7.6 Hz, 1H), 7.53 (t, *J* = 7.6 Hz, 1H), 7.31 (d, *J* = 7.6 Hz, 1H), 7.27-7.17 (m, 5H), 7.12 (t, *J* = 7.6 Hz, 1H), 4.87-4.76 (m, 1H), 4.32 (t, *J* = 8.8 Hz, 1H), 4.16 (dd, *J* = 8.0, 7.6 Hz, 1H), 3.38 (dd, *J* = 13.6, 4.4 Hz, 1H), 2.87 (dd, *J* = 13.6, 9.2 Hz, 1H), 2.66 (s, 3H). ^{13}C NMR (101 MHz, $CDCl_3$) δ164.3, 163.4, 157.1, 150.4, 139.6, 137.5, 137.3, 132.3,

129.3, 129.2, 128.5, 126.5, 125.8, 122.5, 120.1, 119.9, 114.4, 70.3, 68.0, 41.7, 24.3. HRMS (ESI) calculated for $[M+Na]^+[C_{23}H_{21}N_3O_2Na]^+$ requires m/z 394.1531, found m/z 394.1527.

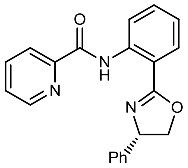

(*S*)-*N*-(2-(4-phenyl-4,5-dihydrooxazol-2-yl)phenyl)picolinamide (Lsa): Prepared according to the general procedure using picolinic acid (0.1979 g, 1.6 mmol), **S2** (0.2384 g, 1.0 mmol), DCM (5.0 mL), DCC (0.3329 g, 1.6 mmol) and DMAP (0.1968 g, 1.6 mmol). After 24 h, the reaction mixture was concentrated in vacuo and purified by column chromatography using PE/EA = 10/1 as the eluent to afford **Lsa** (0.3242 g, 0.94 mmol, 94% yield) as a white solid. M.P.: 92.0-93.1 °C; IR (neat): 3059, 2890, 1679, 1640, 1580, 1525, 1446 cm^{-1}; Optical Rotation: $[\alpha]^{20}_D$ = +256.0 (c 1.02, $CHCl_3$); ^1H NMR (400 MHz, $CDCl_3$): δ13.88 (s, 1H), 9.08 (dd, J = 8.8, 1.6 Hz, 1H), 8.26 (d, J = 7.2 Hz, 2H), 7.97 (dd, J = 8.0, 1.2 Hz, 1H), 7.88-7.78 (m, 1H), 7.61-7.50 (m, 3H), 7.42-7.28 (m, 4H), 7.21-7.12 (m, 1H), 5.67 (t, J = 9.6 Hz, 1H), 4.91-4.82 (m, 1H), 4.24 (t, J = 9.2 Hz, 1H); ^{13}C NMR: (101 MHz, $CDCl_3$): δ164.1, 164.0, 150.9, 148.2, 142.0, 139.7, 137.1, 132.6, 129.5, 128.6, 127.4, 126.7, 126.0, 122.7, 122.6, 120.2, 114.5, 73.1, 70.2; HRMS (ESI) calculated for $[M+Na]^+[C_{21}H_{17}N_3O_2Na]^+$ requires m/z 366.1218, found m/z 366.1220.

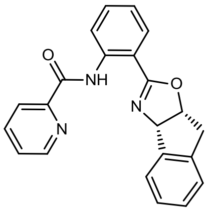

***N*-(2-((3a*S*,8a*R*)-8,8a-dihydro-3aH-indeno[1,2-*d*]oxazol-2-yl)phenyl)picolinamide (Lsb):** Prepared according to the general procedure using picolinic acid (0.7580 g, 6.1 mmol), **S3** (1.2248 g, 4.9 mmol), DCM (25 mL), EDCI (1.1618 g, 6.0 mmol) and DMAP (0.7428 g, 6.0 mmol). After 24 h, the reaction mixture was concentrated in vacuo and purified by column chromatography using PE/EA = 10/1 as the eluent to afford **Lsb** (0.8450 g, 2.4 mmol, 49% yield) as a white solid. M.P.: 194.6-196.9 °C; Optical Rotation: $[\alpha]^{20}_D$ = +280.0 (c 0.52, $CHCl_3$) (lit.[5]: $[\alpha]^{27}_D$ = -74 (c 0.5, CH_2Cl_2)); ^1H NMR (400 MHz, $CDCl_3$): δ13.73 (s, 1H), 8.99 (d, J = 8.4 Hz, 1H), 8.88 (d, J = 4.0 Hz, 1H), 8.29 (d, J = 8.0 Hz, 1H), 7.97-7.86 (m, 2H), 7.70-7.62 (m, 1H), 7.57-7.46 (m, 2H), 7.31-7.21 (m, 3H), 7.15-7.04 (m, 1H), 5.94 (d, J = 8.0 Hz, 1H), 5.52-5.42 (m, 1H), 3.54 (dd, J = 18.0, 6.8 Hz, 1H), 3.40 (d, J = 18.0 Hz, 1H); ^{13}C NMR: (101 MHz, $CDCl_3$): δ164.0, 163.4, 151.2, 148.2, 141.9, 139.6, 139.5, 137.2, 132.3, 129.4, 128.6, 127.3, 126.1, 125.5, 125.3, 122.9, 122.6, 120.2, 114.6, 81.8, 77.1, 39.7; HRMS (ESI) calculated for $[M+H]^+[C_{22}H_{18}N_3O_2]^+$ requires m/z

356.1399, found m/z 356.1408.

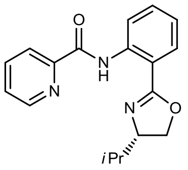

(*S*)-*N*-(2-(4-isopropyl-4,5-dihydrooxazol-2-yl)phenyl)picolinamide (Lsc): Prepared according to the general procedure using picolinic acid (0.1995 g, 1.6 mmol), **S4** (0.2065 g, 1.0 mmol), DCM (5.0 mL), DCC (0.3329 g, 1.6 mmol) and DMAP (0.1963 g, 1.6 mmol). After 24 h, the reaction mixture was concentrated in vacuo and purified by column chromatography using PE/EA = 10/1 as the eluent to afford **Lsc** (0.2674 g, 0.0.86 mmol, 86% yield) as a white solid. M.P.: 92.9-94.4 ^{o}C; IR (neat): 3062, 2958, 1680, 1643, 1581, 1524, 1447 cm^{-1}; Optical Rotation: $[\alpha]^{20}_{D}$ = +27.0 (c 1.01, $CHCl_3$); ^{1}H NMR (400 MHz, $CDCl_3$):δ13.74 (s, 1H), 9.06 (d, *J* = 8.4 Hz, 1H), 8.69-8.60 (m, 1H), 8.34-8.27 (m, 1H), 7.95-7.84 (m, 2H), 7.56-7.49 (m, 1H), 7.45 (ddd,*J* = 5.6, 4.4, 1.2 Hz, 1H), 7.17-7.09 (m, 1H), 4.47-4.39 (m, 1H), 4.31-4.20 (m, 1H), 4.11-4.02 (m, 1H), 1.91-1.80 (m, 1H), 1.20 (d, *J* = 6.4 Hz, 3H), 1.07 (d,*J* = 6.8 Hz, 3H); ^{13}C NMR: (101 MHz, $CDCl_3$): δ 164.1, 162.9, 151.1, 148.2, 139.6, 137.2, 132.2, 129.4, 126.1, 122.9, 122.7, 120.3, 114.7, 73.5, 69.5, 33.5, 19.2, 18.7; HRMS (ESI) calculated for $[M+Na]^+[C_{18}H_{19}N_3O_2Na]^+$ requires m/z 332.1375, found m/z 332.1376.

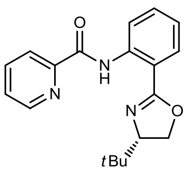

(*S*)-*N*-(2-(4-(tert-butyl)-4,5-dihydrooxazol-2-yl)phenyl)picolinamide (Lsd): Prepared according to the general procedure using picolinic acid (0.1995 g, 1.6 mmol), **S5** (0.2184 g, 1.0 mmol), DCM (5.0 mL), DCC (0.3334 g, 1.6 mmol) and DMAP (0.1951 g, 1.6 mmol). After 24 h, the reaction mixture was concentrated in vacuo and purified by column chromatography using PE/EA = 10/1 as the eluent to afford **Lsd** (0.2861 g, 0.88 mmol, 88% yield) as a white solid. M.P.: 100.7-102.1 ^{o}C; IR (neat): 3061, 2953, 1680, 1644, 1582, 1524, 1447 cm^{-1}; Optical Rotation: $[\alpha]^{20}_{D}$ = +38.1 (c 1.0, $CHCl_3$); ^{1}H NMR (400 MHz, $CDCl_3$): δ 13.70 (s, 1H), 9.07 (d, *J* = 8.4 Hz, 1H), 8.66-8.59 (m, 1H), 8.31 (d, *J* = 8.0 Hz, 1H), 7.96-7.83 (m, 2H), 7.56-7.49 (m, 1H), 7.48-7.40 (m, 1H), 7.18-7.09 (m, 1H), 4.38-4.24 (m, 2H), 4.18 (t, *J* = 7.6 Hz, 1H), 1.07 (s, 9H); ^{13}C NMR: (101 MHz, $CDCl_3$): δ164.2, 162.8, 151.1, 148.2, 139.6, 137.2, 132.2, 129.4, 126.1, 122.9, 122.7, 120.3, 114.6, 76.8, 67.3, 33.9, 25.9; HRMS (ESI) calculated for $[M+Na]^+[C_{19}H_{21}N_3O_2Na]^+$ requires m/z 346.1531, found m/z 346.1529.

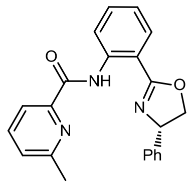

(*S*)-6-methyl-*N*-(2-(4-phenyl-4,5-dihydrooxazol-2-yl)phenyl)picolinamide (Lse): Prepared according to the general procedure using 6-methylpicolinic acid (1.0980 g, 8.0 mmol), **S2** (0.7658 g, 3.2 mmol), DCM (20 mL), DCC (1.6528 g, 8.0 mmol) and DMAP (0.9780 g, 8.0 mmol). After 24 h, the reaction mixture was concentrated in vacuo and purified by column chromatography using PE/EA = 10/1 as the eluent to afford **Lse** (1.0836 g, 3.0 mmol, 94% yield) as a white solid. M.P.: 92.1-93.4 ^{o}C. IR (neat): 3063, 3026, 1680, 1640, 1583, 1524, 1446 cm^{-1}. Optical Rotation: $[\alpha]^{20}_{D}$ = +239.7 (c 1.0, $CHCl_3$).^{1}H NMR (400 MHz, $CDCl_3$) δ 13.86 (s, 1H), 9.20-8.90 (m, 1H), 8.05 (d, J = 7.6 Hz, 1H), 7.99 (dd, J = 8.0, 1.6 Hz, 1H), 7.69 (t, J = 7.6 Hz, 1H), 7.60-7.52 (m, 1H), 7.41-7.26 (m, 5H), 7.20-7.13 (m, 2H), 5.76-5.49 (m, 1H), 4.81 (dd, J = 10.0, 8.4 Hz, 1H), 4.19 (t, J = 8.4 Hz, 1H), 2.04 (s, 3H); ^{13}C NMR (101 MHz, $CDCl_3$) δ164.38, 164.35, 157.4, 150.1, 142.2, 139.9, 137.2, 132.6, 129.5, 128.6, 127.6, 126.8, 125.7, 122.5, 120.2, 119.6, 114.3, 73.7, 70.4, 23.3; HRMS (ESI) calculated for $[M+Na]^+[C_{22}H_{19}N_3O_2Na]^+$ requires m/z 380.1375, found m/z 380.1367.

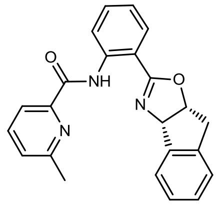

***N*-(2-((3a*S*,8a*R*)-8,8a-dihydro-3aH-indeno[1,2-d]oxazol-2-yl)phenyl)-6-methylpicolinamide (Lsf):** Prepared according to the general procedure using 6-methylpicolinic acid (0.1808 g, 1.3 mmol), **S3** (0.2506 g, 1.0 mmol), DCM (5.0 mL), DCC (0.2728 g, 1.3 mmol) and DMAP (0.1640 g, 1.3 mmol). After 24 h, the reaction mixture was concentrated in vacuo and purified by column chromatography using PE/EA = 10/1 as the eluent to afford **Lsf** (0.1760 g, 0.48 mmol, 48% yield) as a white solid. M.P.: 201.6-203.1 ^{o}C. IR (neat): 3023, 2923, 1682, 1638, 1583, 1529, 1447 cm^{-1}. Optical Rotation: $[\alpha]^{20}_{D}$ = +437.6 (c 0.92, $CHCl_3$).^{1}H NMR (400 MHz, $CDCl_3$) δ 13.71 (s, 1H), 8.97 (d, J = 8.4 Hz, 1H), 8.11 (d, J = 7.6 Hz, 1H), 7.89 (dd, J = 7.6, 1.2 Hz, 1H), 7.80 (t, J = 7.6 Hz, 1H), 7.54 (d, J = 7.2 Hz, 1H), 7.50-7.45 (m, 1H), 7.38 (d, J = 8.0 Hz, 1H), 7.29-7.18 (m, 3H), 7.12-7.02 (m, 1H), 5.96 (d, J = 7.6 Hz, 1H), 5.50-5.41 (m, 1H), 3.58-3.40 (m, 2H), 2.87 (s, 3H); ^{13}C NMR (101 MHz, $CDCl_3$) δ164.3, 163.6, 157.0, 150.6, 141.9, 139.62, 139.57, 137.4, 132.3, 129.4, 128.6, 127.4, 125.9, 125.4, 122.5, 120.4, 120.1, 114.7, 82.2, 39.4, 24.7; HRMS (ESI) calculated for $[M+Na]^+[C_{23}H_{19}N_3O_2Na]^+$ requires m/z 392.1375, found m/z 392.1376.

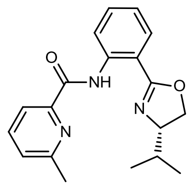

(*S*)-*N*-(2-(4-isopropyl-4,5-dihydrooxazol-2-yl)phenyl)-6-methylpicolinamide (Lsg): Prepared according to the general procedure using 6-methylpicolinic acid (0.6588 g, 4.8 mmol), **S4** (0.6168 g, 3.0 mmol), DCM (15 mL), DCC (0.9908 g, 4.8 mmol) and DMAP (0.5909 g, 4.8 mmol). After 24 h, the reaction mixture was concentrated in vacuo and purified by column chromatography using PE/EA = 10/1 as the eluent to afford **Lsg** (0.9244 g, 2.9 mmol, 95% yield) as a white solid. M.P.: 81.3-82.6 ^{o}C; IR (neat): 3091, 2960, 1679, 1642, 1582, 1520, 1444 cm^{-1}; Optical Rotation: $[\alpha]^{20}_{D}$ = +146.0 (c 1.03, $CHCl_3$); ^{1}H NMR (400 MHz, $CDCl_3$) δ 13.44 (s, 1H), 8.97 (d, J = 8.4 Hz, 1H), 8.10 (d, J = 7.6 Hz, 1H), 7.90 (d, J = 7.6 Hz, 1H), 7.76 (t, J = 7.6 Hz, 1H), 7.51 (t, J = 8.0 Hz, 1H), 7.31 (d, J = 7.6 Hz, 1H), 7.12 (t, J = 7.6 Hz, 1H), 4.42-4.28 (m, 2H), 4.20-4.12 (m, 1H), 2.66 (s, 3H), 2.05-1.90 (m, 1H), 1.02 (d, J = 6.8 Hz, 3H), 0.94 (d, J = 6.8 Hz, 3H); ^{13}C NMR (101 MHz, $CDCl_3$) δ164.4, 162.7, 157.2, 150.5, 139.4, 137.2, 132.0, 129.3, 125.8, 122.5, 120.4, 120.0, 114.8, 72.7, 68.4, 32.7, 24.4, 18.9, 17.8; HRMS (ESI) calculated for $[M+Na]^+[C_{19}H_{21}N_3O_2Na]^+$ requires m/z 346.1531, found m/z 346.1532.

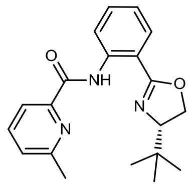

(*S*)-*N*-(2-(4-(tert-butyl)-4,5-dihydrooxazol-2-yl)phenyl)-6-methylpicolinamide (Lsh): Prepared according to the general procedure using 6-methylpicolinic acid (0.1287 g, 0.94 mmol), **S5** (0.1317 g, 0.6 mmol), DCM (5 mL), DCC (0.1989 g, 0.96 mmol) and DMAP (0.1145 g, 0.94 mmol). After 24 h, the reaction mixture was concentrated in vacuo and purified by column chromatography using PE/EA = 10/1 as the eluent to afford **Lsh** (0.1272 g, 0.38 mmol, 63% yield) as a white solid. M.P.: 67.2-68.4 ^{o}C. IR (neat): 3097, 2957, 1680, 1643, 1584, 1522, 1445 cm^{-1}. Optical Rotation: $[\alpha]^{20}_{D}$ = +200.0 (c 1.02, $CHCl_3$).^{1}H NMR (400 MHz, $CDCl_3$) δ13.23 (s, 1H), 8.92 (dd, J = 8.4, 0.4 Hz, 1H), 8.10 (d, J = 7.6 Hz, 1H), 7.90 (dd, J = 8.0, 1.6 Hz, 1H), 7.75 (t, J = 7.6 Hz, 1H), 7.55-7.48 (m, 1H), 7.30 (d, J = 7.6 Hz, 1H), 7.16-7.09 (m, 1H), 4.35-4.20 (m, 3H), 2.65 (s, 3H), 0.94 (s, 9H); ^{13}C NMR (101 MHz, $CDCl_3$) δ164.6, 162.7, 157.4, 150.8, 139.5, 137.3, 132.0, 129.3, 125.9, 122.7, 120.8, 120.3, 115.0, 76.6, 67.3, 34.2, 25.9, 24.6. HRMS (ESI) calculated for $[M+Na]^+[C_{20}H_{23}N_3O_2Na]^+$ requires m/z 360.1688, found m/z 360.1685.

Synthesis of **L6-L9:**

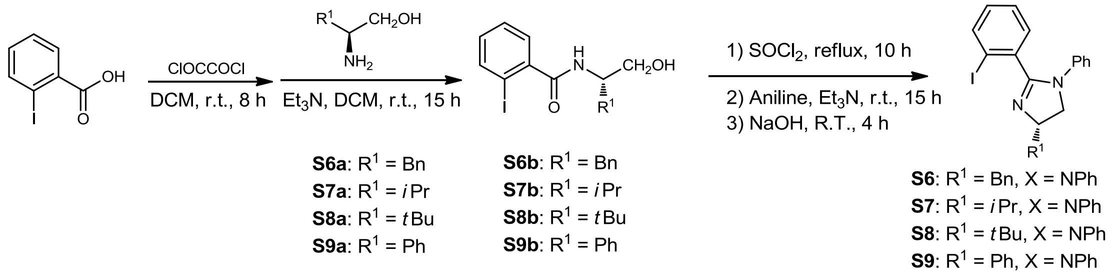

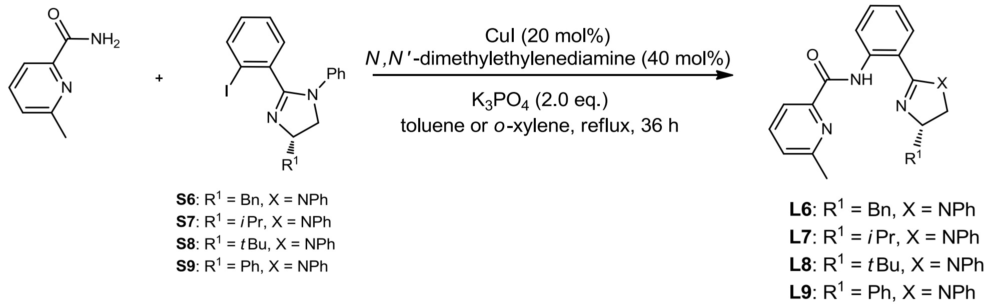

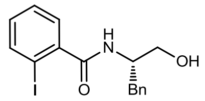

(*S*)-*N*-(1-hydroxy-3-phenylpropan-2-yl)-2-iodobenzamide (S6b): 2-Iodobenzoic acid (24.80 g, 100 mmol) was dissolved in dichloromethane (200 mL) in a 250 mL roundbottomed flask and cooled on an ice bath. Oxalyl chloride (13.0 mL, 150 mmol) and DMF (5 drops) was added in sequence under stirring, and the reaction was allowed to come to room temperature. After 8 hours the reaction mixture was evaporated to give the crude product without purification and used in the next step. The acyl chloride was dissolved in dichloromethane (30 mL) and added dropwise to a solution of the amino alcohol **S6a** (15.12 g, 100 mmol) in triethylamine (35.0 mL, 0.73 g/mL, 250 mmol) and dichloromethane (200 mL) at 0 ℃. Then the mixture was warmed to room temperature and stirred for 12 h. After completion of the reaction, the reaction mixture was then washed successively with a saturated aqueous solution of ammonium chloride, HCl (1 M), saturated aqueous solution of sodium hydrogen carbonate and brine. The resulting organic layer was then dried with anhydrous sodium sulfate and concentrated in vacuo to yield crude product as a pale yellow solid and washed with 120 mL of PE/EtOAc (5/1) to afford **S6b** (23.74 g, 62 mmol, 62% yield) as a white solid. M.P.: 133.7-135.3 ^{o}C. IR (neat): 3278, 1638, 1535, 1037 cm^{-1}. Optical Rotation: $[\alpha]^{20}_{D}$ = -21.6 (c 0.99, CH_2Cl_2). ^{1}H NMR (400 MHz, CD_3OD) δ 7.84 (d, *J* = 8.0 Hz, 1H), 7.40-7.27 (m, 5H), 7.24-7.19 (m, 1H), 7.15-7.04 (m, 2H), 4.35-4.29 (m, 1H), 3.69 (dd, *J* = 11.2, 5.6 Hz, 1H), 3.63 (dd, *J* = 11.2, 5.6 Hz, 1H), 3.06 (dd,

J = 13.6, 5.6 Hz, 1H), 2.83 (dd, J = 14.0, 8.8 Hz, 1H); ^{13}C NMR (101 MHz, CD_3OD) δ172.2, 144.2, 140.6, 139.9, 131.8, 130.4, 129.4, 129.0, 128.9, 127.4, 93.2, 64.2, 54.6, 37.9. HRMS (ESI) calculated for $[M+H]^+[C_{16}H_{17}INO_2]^+$ requires m/z 382.0304, found m/z 382.0308.

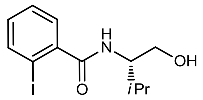

(*S*)-*N*-(1-hydroxy-3-methylbutan-2-yl)-2-iodobenzamide (S7b): 2-Iodobenzoic acid (19.89 g, 80 mmol) was dissolved in dichloromethane (200 mL) in a 250 mL round bottomed flask and cooled on an ice bath. Oxalyl chloride (10.0 mL, 120 mmol) and DMF (10 drops) was added in sequence under stirring, and the reaction was allowed to come to room temperature. After 8 hours the reaction mixture was evaporated to give the crude product without purification and used in the next step. The acyl chloride was dissolved in dichloromethane (20 mL) and added dropwise to a solution of the amino alcohol **S7a** (8.2890 g, 80 mmol) in triethylamine (28 mL, 0.73 g/mL, 200 mmol) and dichloromethane (200 mL) at 0 ℃. Then the mixture was warmed to room temperature and stirred for 12 h. After completion of the reaction, the reaction mixture was then washed successively with a saturated aqueous solution of ammonium chloride, HCl (1 M), saturated aqueous solution of sodium hydrogen carbonate and brine. The resulting organic layer was then dried with anhydrous sodium sulfate and concentrated in vacuo to yield crude product as a pale yellow solid and washed with 110 mL of PE/EtOAc (10/1) to afford **S7b** (24.31 g, 73 mmol, 91% yield) as a white solid. M.P.: 112.9-114.2 ^{o}C. IR (neat): 3272, 2960, 1637, 1537, 1017 cm^{-1}. Optical Rotation: $[\alpha]^{20}_D$ = -28.3 (c 0.98, CH_2Cl_2). ^{1}H NMR (400 MHz, CD_3OD) δ7.89 (d, J = 8.0 Hz, 1H), 7.47-7.37 (m, 2H), 7.15 (td, J = 7.6, 1.6 Hz, 1H), 3.91 (q, J = 6.0 Hz, 1H), 3.76-3.64 (m, 2H), 2.09-1.97 (m, 1H), 1.07 (d, J = 6.8 Hz, 3H), 1.04 (d, J = 6.8 Hz, 3H); ^{13}C NMR (101 MHz, CD_3OD) δ172.6, 144.5, 140.6, 131.8, 129.08, 129.05, 93.2, 62.9, 58.4, 29.9, 20.2, 19.0.HRMS (ESI) calculated for $[M+H]^+[C_{12}H_{17}INO_2]^+$ requires m/z 334.0304, found m/z 334.0306.

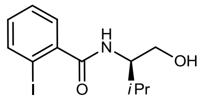

(*R*)-*N*-(1-hydroxy-3-methylbutan-2-yl)-2-iodobenzamide (ent-S7b): 2-Iodobenzoic acid (12.44 g, 50 mmol) was dissolved in dichloromethane (60 mL) in a 250 mL round-bottomed flask and cooled on an ice bath. Oxalyl chloride (6.3 mL, 75 mmol) and DMF (8 drops) was added in sequence under stirring, and the reaction was allowed to come to room temperature. After 7 hours the reaction mixture was evaporated to give the crude

product without purification and used in the next step. The acyl chloride was dissolved in dry dichloromethane (30 mL) and added dropwise to a solution of the amino alcohol (*R*)-2-amino-3-methylbutan-1-ol (5.18 g, 50 mmol) in triethylamine (17 mL, 0.73 g/mL, 125 mmol) and dichloromethane (30 mL) at 0 ℃. Then the mixture was warmed to room temperature and stirred for 12 h. After completion of the reaction, the reaction mixture was then washed successively with a saturated aqueous solution of ammonium chloride, HCl (1 M), saturated aqueous solution of sodium hydrogen carbonate and brine. The resulting organic layer was then dried with anhydrous sodium sulfate and concentrated in vacuo to yield crude product as a pale yellow solid and washed with 100 mL of PE/EtOAc (10/1) to afford **ent-S7b** (15.30 g, 46mmol, 92% yield) as a white solid. M.P.: 111.7-113.1°C. IR (neat): 3271, 2958, 1638, 1536, 1015 cm^{-1}. Optical Rotation: $[\alpha]^{20}_{D}$ = +30.6 (c 1.02, $CHCl_3$). ^{1}H NMR (400 MHz, $CDCl_3$) δ7.82 (d, *J* = 8.0 Hz, 1H), 7.41-7.29 (m, 2H), 7.12-7.03 (m, 1H), 6.21 (d, *J* = 7.6 Hz, 1H), 3.94-3.84 (m, 1H), 3.83-3.69 (m, 2H), 2.93 (brs, 1H), 2.02-1.93 (m, 1H), 1.02 (d, *J* = 6.8 Hz, 6H); ^{13}C NMR (101 MHz, CD_2Cl_2) δ169.4, 142.3, 139.3, 130.5, 127.7, 127.5, 91.8, 62.7, 57.1, 28.6, 18.9, 18.4; HRMS (ESI) calculated for $[M+H]^+[C_{12}H_{17}INO_2]^+$ requires334.0304 m/z, found m/z 334.0320.

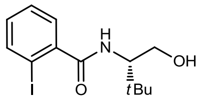

(*S*)-*N*-(1-hydroxy-3,3-dimethylbutan-2-yl)-2-iodobenzamide (S8b): 2-Iodobenzoic acid (19.80 g, 80 mmol) was dissolved in dichloromethane (200 mL) in a 250 mL roundbottomed flask and cooled on an ice bath. Oxalyl chloride (10.0 mL, 120 mmol) and DMF (10 drops) was added in sequence under stirring, and the reaction was allowed to come to room temperature. After 8 hours the reaction mixture was evaporated to give the crude product without purification and used in the next step. The acyl chloride was dissolved in dichloromethane (20 mL) and added dropwise to a solution of the amino alcohol **S8a** (9.3820 g, 80 mmol) in triethylamine (28 mL, 0.73 g/mL, 200 mmol) and dichloromethane (200 mL) at 0 ℃. Then the mixture was warmed to room temperature and stirred for 12 h. After completion of the reaction, the reaction mixture was then washed successively with a saturated aqueous solution of ammonium chloride, HCl (1 M), saturated aqueous solution of sodium hydrogen carbonate and brine. The resulting organic layer was then dried with anhydrous sodium sulfate and concentrated in vacuo to yield crude product as a pale yellow solid and recrystallized from PE/EtOAc to afford **S8b** (23.0441 g, 66 mmol, 83% yield) as a white solid. M.P.: 126.4-128.1°C. IR (neat): 3284, 2959,

1639, 1535, 1017 cm^{-1}. Optical Rotation: $[\alpha]^{20}_{D}$ = -13.9 (c 1.02, CH_2Cl_2).^{1}H NMR (400 MHz, CD_3OD) δ7.90 (d, *J* = 8.0 Hz, 1H), 7.47-7.40 (m, 2H), 7.19-7.11 (m, 1H), 3.97 (dd, *J* = 8.8, 4.0 Hz, 1H), 3.88 (dd, *J* = 11.6, 4.0 Hz, 1H), 3.57 (dd, *J* = 11.6, 8.8 Hz, 1H), 1.05 (s, 9H); ^{13}C NMR (101 MHz, CD_3OD) δ 173.0, 144.7, 140.7, 131.8, 129.4, 129.1, 93.1, 62.4, 61.5, 35.1, 27.7.HRMS (ESI) calculated for $[M+H]^+[C_{13}H_{19}INO_2]^+$ requires m/z 348.0460, found m/z 348.0463.

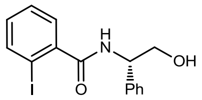

(*S*)-*N*-(2-hydroxy-1-phenylethyl)-2-iodobenzamide (S9b): 2-Iodobenzoic acid (19.81 g, 80 mmol) was dissolved in dichloromethane (150 mL) in a 250 mL round bottomed flask and cooled on an ice bath. Oxalyl chloride (10.0 mL, 120 mmol) and DMF (6 drops) was added in sequence under stirring, and the reaction was allowed to come to room temperature. After 8 hours the reaction mixture was evaporated to give the crude product without purification and used in the next step. The acyl chloride was dissolved in dichloromethane (30 mL) and added dropwise to a solution of the amino alcohol **S9a** (10.94 g, 80 mmol) in triethylamine (28 mL, 0.73 g/mL, 200 mmol) and dichloromethane (75 mL) at 0 ℃. Then the mixture was warmed to room temperature and stirred for 18 h. After completion of the reaction, the reaction mixture was then washed successively with a saturated aqueous solution of ammonium chloride, HCl (1 M), saturated aqueous solution of sodium hydrogen carbonate and brine. The resulting organic layer was then dried with anhydrous sodium sulfate and concentrated in vacuo to yield crude product as a pale yellow solid and washed with105 mL of PE/DCM (85/20) to afford **S9b** (14.6019 g, 40 mmol, 50% yield)asa white solid. M.P.: 152.1-153.3^{o}C. IR (neat): 3285, 1641, 1535, 1022 cm^{-1}. Optical Rotation: $[\alpha]^{20}_{D}$ = +23.8 (c 1.03, CH_2Cl_2). ^{1}H NMR (400 MHz, CD_3OD) δ7.89 (d, *J* = 7.2 Hz, 1H), 7.48-7.32 (m, 6H), 7.31-7.25 (m, 1H), 7.18-7.13 (m, 1H), 5.16 (t, *J* = 6.8 Hz, 1H), 3.84 (d, *J* = 6.8 Hz, 2H); ^{13}C NMR (101 MHz, CD_3OD) δ172.3, 144.2, 140.8, 140.8, 132.0, 129.5, 129.2, 129.1, 128.5, 128.4, 93.2, 66.0, 57.6. HRMS (ESI) calculated for $[M+H]^+[C_{15}H_{15}INO_2]^+$ requires m/z 368.0147, found m/z 368.0150.

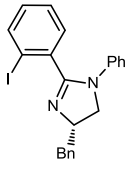

(*S*)-4-benzyl-2-(2-iodophenyl)-1-phenyl-4,5-dihydro-1*H*-imidazole (S6): Prepared according to a previously reported procedure with a slight modification,[6] a 50 mL flame-dried Schlenk flask was washed with nitrogen three times and charged with amide **S6b** (7.60 g, 20 mmol), toluene (15 mL) and $SOCl_2$ (5.0 mL, 1.60 g/mL, 67 mmol).

Then the mixture was refluxed for 12 h. After cooling to room temperature, the mixture was concentrated and the residue was dissolved in 150 mL Et_2O and transferred to a 250 mL flame-dried round bottomed flask. To this reaction mixture, triethylamine (31 mL, 0.73 g/mL, 224 mmol) and aniline (2.1 mL, 1.02 g/mL, 23 mmol) were added in sequence. After stirred at room temperature for 48 h, 10% NaOH (200 mL) was added and stirred for another 5 h. The organic layer was separated and the aqueous layer extracted with EtOAc (4× 20 mL). The combined organic phases were dried over anhydrous Na_2SO_4 and concentrated in vacuo and purified by flash column chromatography using PE/EtOAc (50/1 to 5/1) as the eluent to afford **S6** (4.7986 g, 11 mmol, 55% yield) as a yellow oil.IR (neat): 3058, 2972, 1601, 1576, 1498, 1476, 1384 cm^{-1};Optical Rotation: $[\alpha]^{20}_D$ = -9.8 (c 1.10, $CHCl_3$);^{1}H NMR (400 MHz, $CDCl_3$): δ7.77 (d, J = 8.0 Hz, 1H), 7.42-7.28 (m, 6H), 7.25-7.20 (m, 1H), 7.10-7.02 (m, 3H), 6.88 (t, J = 7.6 Hz, 1H), 6.58 (d, J = 8.0 Hz, 2H), 4.6-4.52 (m, 1H), 4.03-3.96 (m, 1H), 3.77 (dd, J = 8.8, 7.6 Hz, 1H), 3.34 (dd, J = 13.6, 4.8 Hz, 1H), 2.88 (dd, J = 13.6, 8.8 Hz, 1H);^{13}C NMR (101 MHz, $CDCl_3$): δ161.7, 140.5, 139.4, 138.4, 137.5, 130.7, 130.3, 129.4, 128.6, 128.4, 128.1, 126.3, 122.5, 119.5, 96.4, 65.3, 55.7, 41.9. HRMS (ESI) calculated for $[M+Na]^+[C_{22}H_{19}IN_2Na]^+$ requires m/z 461.0491, found m/z 461.0485.

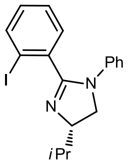

(*S*)-2-(2-iodophenyl)-4-isopropyl-1-phenyl-4,5-dihydro-1H-imidazole (S7): Prepared according to a previously reported procedure with a slight modification,[6] a 100 mL flame-dried Schlenk flask was washed with nitrogen three times and charged with amide **S7b** (8.3322 g, 25 mmol), toluene (10 mL) and $SOCl_2$ (10.0 mL, 1.60 g/mL, 134 mmol). Then the mixture was refluxed for 12 h. After cooling to room temperature, the mixture was concentrated and the residue was dissolved in 30mL DCM and transferred to a 250 mL flame-dried round bottomed flask. To this reaction mixture, triethylamine (28 mL, 0.73 g/mL, 200 mmol) and aniline (2.5 mL, 1.02 g/mL, 27.5 mmol) were added in sequence. After stirred at room temperature for 30 h, 10% NaOH (200 mL) was added and stirred for another 5 h. The organic layer was separated and the aqueous layer extracted with DCM (3× 200 mL). The combined organic phases were dried over anhydrous Na_2SO_4 and concentrated in vacuo and purified by flash column chromatography using PE/EtOAc (50/1 to 5/1) as the eluent to afford **S7**(9.4098 g, 24 mmol, 96% yield) as a yellow oil. IR (neat): 3057, 2956, 1599, 1579, 1498, 1475,

1382 cm^{-1}; Optical Rotation: $[\alpha]^{20}_{D}$ = -71.9 (c 1.20, $CHCl_3$); ^{1}H NMR (400 MHz, $CDCl_3$): δ7.76 (dd, J = 8.0, 0.8 Hz, 1H), 7.43 (dd, J = 7.6, 1.6 Hz, 1H), 7.36 (td, J = 7.6, 0.8 Hz, 1H), 7.12-7.02 (m, 3H), 6.92-6.86 (m, 1H), 6.64 (dd, J = 8.4, 0.8 Hz, 2H), 4.14-3.96 (m, 2H), 3.78 (t, J = 8.0 Hz, 1H), 2.07-1.94 (m, 1H), 1.12 (d, J = 6.8 Hz, 3H), 1.05 (d, J = 6.8 Hz, 3H); ^{13}C NMR (101 MHz, $CDCl_3$): δ161.0, 140.7, 139.3, 137.9, 130.5, 130.3, 128.5, 128.1, 122.1, 119.2, 96.3, 70.1, 53.7, 32.9, 19.1, 18.4. HRMS (ESI) calculated for $[M+Na]^+[C_{18}H_{19}IN_2Na]^+$ requires m/z 413.0491, found m/z 413.0490.

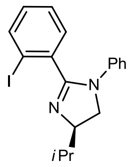

(*R*)-2-(2-iodophenyl)-4-isopropyl-1-phenyl-4,5-dihydro-1H-imidazole (ent-S7): Prepared according to a previously reported procedure with a slight modification,[6] a 50 mL flame-dried Schlenk flask was washed with nitrogen three times and charged with amide **ent-S7b** (6.6600 g, 20mmol), $SOCl_2$ (4.4 mL, 1.60 g/mL, 60 mmol). Then the mixture was refluxed for 12 h. After cooling to room temperature, the mixture was concentrated and the residue was dissolved in 20 mL DCM and transferred to a 250 mL flame-dried round-bottomed flask. To this reaction mixture, triethylamine (28 mL, 0.73 g/mL, 200 mmol) and aniline (2.0 mL, 1.02 g/mL, 22mmol) were added in sequence. After stirred at room temperature for 12 h, 10% NaOH (200 mL) was added and stirred for another 4 h. The organic layer was separated and the aqueous layer extracted with DCM (3 × 200 mL). The combined organic phases were dried over anhydrous Na_2SO_4 and concentrated in vacuo and purified by flash column chromatography using DCM to DCM/MeOH(30/1) as the eluent to afford **ent-S7** (7.2505 g, 19 mmol, 93% yield) as a brown oil. IR (neat): 3060, 2955, 1598, 1578, 1500, 1476, 1381 cm^{-1}; Optical Rotation: $[\alpha]^{20}_{D}$ = +75.1 (c 1.02, $CHCl_3$); ^{1}H NMR (400 MHz, $CDCl_3$): δ7.74 (d, J = 8.0 Hz, 1H), 7.43 (dd, J = 7.6, 0.8 Hz, 1H), 7.36 (t, J = 7.6 Hz, 1H), 7.10-7.01 (m, 3H), 6.91-6.86 (m, 1H), 6.64 (d, J =7.6 Hz, 2H), 4.11-3.98 (m, 2H), 3.78 (t, J = 8.0 Hz, 1H), 2.07-1.94 (m, 1H), 1.12 (d, J = 6.8 Hz, 3H), 1.05 (d, J = 6.4 Hz, 3H); ^{13}C NMR (101 MHz, $CDCl_3$): δ161.0, 140.6, 139.3, 137.8, 130.5, 130.3, 128.5, 128.0, 122.1, 119.2, 96.2, 70.0, 53.7, 32.8, 19.1, 18.4; HRMS (ESI) calculated for $[M+Na]^+[C_{18}H_{19}IN_2Na]^+$ requires m/z 413.0491, found m/z 413.0492.

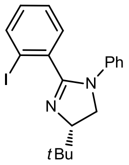

(*S*)-4-(tert-butyl)-2-(2-iodophenyl)-1-phenyl-4,5-dihydro-1H-imidazole (S8): Prepared according to a previously reported procedure with a slight modification,[6] a 100 mL flame-dried Schlenk flask was washed with nitrogen three times and charged with amide **S8b** (6.96 g, 20 mmol) and $SOCl_2$ (8.0 mL, 1.60 g/mL, 110 mmol). Then the mixture was refluxed for 12 h. After cooling to room temperature, the mixture was concentrated and the residue was dissolved in 60 mL of DCM/Et_2O(1/2) and transferred to a 250 mL flame-dried roundbottomed flask. To this reaction mixture, triethylamine (22 mL, 0.73 g/mL, 160 mmol) and aniline (2.0 mL, 1.02 g/mL, 22 mmol) were added in sequence. After stirred at room temperature for 11 h, 10% NaOH (200 mL) was added and stirred for another 5 h. The organic layer was separated and the aqueous layer extracted with DCM (3× 200 mL). The combined organic phases were dried over anhydrous Na_2SO_4 and concentrated in vacuo and purified by flash column chromatography using PE/EtOAc (50/1 to 5/1) as the eluent to afford **S8** (8.0620 g, 19.9 mmol, 99% yield) as a yellow oil. IR (neat): 3057, 2950, 1581, 1498, 1477, 1384 cm^{-1};Optical Rotation: $[\alpha]^{20}_D$ = -66.1 (c 0.98, $CHCl_3$); ^1H NMR (400 MHz, $CDCl_3$): δ7.75 (dd, *J* = 8.0, 0.8 Hz, 1H), 7.46 (dd, *J* = 7.6, 1.6 Hz, 1H), 7.37 (td, *J* = 7.6, 0.8 Hz, 1H), 7.12-7.01 (m, 3H), 6.89 (t, *J* = 7.6 Hz, 1H), 6.65 (dd, *J* = 8.4, 0.8 Hz, 2H), 4.06-3.95 (m, 2H), 3.88-3.70 (m, 1H), 1.07 (s, 9H);^{13}C NMR (101 MHz, $CDCl_3$): δ161.2, 140.8, 139.4, 138.0, 130.5, 128.5, 128.1, 122.2, 119.4, 96.2, 73.8, 52.4, 34.2, 26.3; HRMS (ESI) calculated for $[M+Na]^+[C_{19}H_{21}IN_2Na]^+$ requires m/z 427.0647, found m/z 427.0652.

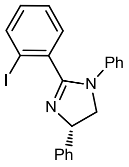

(*S*)-2-(2-iodophenyl)-1,4-diphenyl-4,5-dihydro-1*H*-imidazole (S9): Prepared according to a previously reported procedure with a slight modification,[6] a 100 mL flame-dried Schlenk flask was washed with nitrogen three times and charged with amide **S9b** (5.04 g, 13.7 mmol) and $SOCl_2$ (8.0 mL, 1.60 g/mL, 110 mmol). Then the mixture was refluxed for 11 h. After cooling to room temperature, the mixture was concentrated and the residue was dissolved in 150 mL DCM/Et_2O (1/2) and transferred to a 250 mL flame-dried roundbottomed flask. To this reaction mixture, triethylamine (22 mL, 0.73 g/mL, 160 mmol) and aniline (1.5 mL, 1.02 g/mL, 16.5 mmol) were added in sequence. After stirred at room temperature for 22 h, 10% NaOH (200 mL) was added and stirred for another 5 h. The organic layer was separated and the aqueous layer extracted with DCM (3× 200 mL). The combined organic phases were dried over

anhydrous Na_2SO_4 and concentrated in vacuo and purified by flash column chromatography using PE/EtOAc (50/1 to 5/1) as the eluent to afford **S9** (3.2776 g, 7.7 mmol, 56% yield) as a yellow oil. IR (neat): 3058, 2973, 1602, 1575, 1496, 1476, 1383 cm^{-1};Optical Rotation: $[\alpha]^{20}_{D}$ = -136.1 (c 0.99, $CHCl_3$);^{1}H NMR (400 MHz, $CDCl_3$): δ7.76 (d, J = 7.6 Hz, 1H), 7.53 (d, J = 7.2 Hz, 3H), 7.43-7.35 (m, 3H), 7.33-7.25 (m, 1H), 7.13-7.02 (m, 3H), 6.91 (t, J = 7.6 Hz, 1H), 6.67 (d, J = 8.0 Hz, 2H), 5.37 (t, J = 10.0 Hz, 1H), 4.39 (t, J = 10.0 Hz, 1H), 4.03 (t, J = 9.6 Hz, 1H); ^{13}C NMR (101 MHz, $CDCl_3$):δ162.4, 143.3, 140.4, 139.4, 137.5, 130.8, 130.5, 128.6, 128.5, 128.2, 127.2, 127.1, 122.7, 119.5, 96.3, 67.5, 59.2; HRMS (ESI) calculated for $[M+Na]^+[C_{22}H_{17}IN_2Na]^+$ requires m/z 447.0334, found m/z 447.0330.

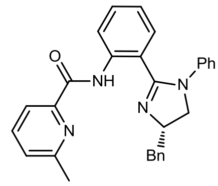

(*S*)-*N*-(2-(4-benzyl-1-phenyl-4,5-dihydro-1H-imidazol-2-yl)phenyl)-6-methylpicolinamide (L6): Prepared according to a previously reported procedure with a slight modification,[7] a 50 mL flame-dried Schlenk flask was washed with nitrogen three times andcharged with CuI (0.0765 g, 0.4 mmol), toluene (15 mL) and *N, N'*-dimethylethylenediamine (80 uL, 0.90 g/mL, 0.8 mmol). After the mixture was stirred at room temperature for 5 min, 6-methylpicolinamide (0.3408 g, 2.5 mmol), **S6** (0.8102 g, 1.85 mmol, 1.0 eq.) and K_3PO_4 (0.8480 g, 4.0 mmol) were added to the flask under N_2 atmosphere. The mixture was refluxed for 48 h. After cooling to room temperature, the mixture was filtered and washed with EtOAc. The combined filtrate were concentrated and purified by column chromatography using PE/EA = 10:1 as the eluent to afford the title compound (0.5128 g, 62% yield) as a light yellow solid. M.P.: 128.0-129.8 ^oC; IR (neat): 3062, 2874, 1679, 1585, 1517, 1446, 1379 cm^{-1}; Optical Rotation: $[\alpha]^{20}_{D}$ = +51.5 (c 1.00, $CHCl_3$);^{1}H NMR (400 MHz, $CDCl_3$): δ12.58 (s, 1H), 8.76 (d, J = 8.0 Hz, 1H), 8.13 (d, J = 7.6 Hz, 1H), 7.77 (t, J = 7.6 Hz, 1H), 7.39 (t, J = 7.6 Hz, 1H), 7.31 (d, J = 8.0 Hz, 1H), 7.29-7.25 (m, 4H), 7.23-7.17 (m, 1H), 7.14 (d, J = 7.6Hz, 1H), 7.08 (t, J = 7.6 Hz, 2H), 6.91 (dt, J = 10.8, 7.6 Hz, 2H), 6.65 (d, J = 8.0 Hz, 2H), 4.81-4.70 (m, 1H), 3.98 (t, J = 9.6 Hz, 1H), 3.85-3.70 (m, 1H), 3.49 (dd, J = 13.6, 4.4 Hz, 1H), 2.88 (dd, J = 13.6, 9.2 Hz, 1H), 2.68 (s, 3H);^{13}C NMR (101 MHz, $CDCl_3$): δ163.4, 159.9, 157.1, 150.0, 142.7, 138.2, 137.7, 137.5, 130.7, 130.0, 129.3, 128.7, 128.5, 126.4, 126.0, 123.5, 122.8, 122.3, 121.4, 119.9, 119.5, 66.1, 57.2, 42.4, 24.5; HRMS (ESI) calculated for $[M+Na]^+[C_{29}H_{26}N_4ONa]^+$ requires m/z 469.2004, found m/z 469.1997.

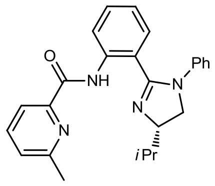

(*S*)-*N*-(2-(4-isopropyl-1-phenyl-4,5-dihydro-1H-imidazol-2-yl)phenyl)-6-methylpicolinamide (L7): Prepared according to a previously reported procedure with a slight modification,[7] a 50 mL flame-dried Schlenk flask was washed with nitrogen three times and charged with CuI (0.0362 g, 0.19 mmol), *o*-xylene (5.0 mL) and *N*, *N'*-dimethylethylenediamine (40 uL, 0.90 g/mL, 0.4 mmol). After the mixture was stirred at room temperature for 5 min, 6-methylpicolinamide (0.1602 g, 1.2 mmol), **S7** (0.3918 g, 1.0 mmol, 1.0 eq.) and K_3PO_4 (0.4345 g, 2.0 mmol) were added to the flask under N_2 atmosphere. The mixture was refluxed for 36 h. After cooling to room temperature, the mixture was filtered and washed with EtOAc. The combined filtrate were concentrated and purified by column chromatography using PE/EA = 10:1 as the eluent to afford the title compound (0.3090 g, 77% yield) as a light yellow solid. M.P.: 147.0-148.5 ^oC; IR (neat): 3064, 2874, 1679, 1586, 1516, 1445, 1377 cm^{-1}; Optical Rotation: $[\alpha]^{20}_D$ = -28.7 (c 0.90, MeOH); ^1H NMR (400 MHz, $CDCl_3$):δ 12.28 (s, 1H), 8.68 (d, *J* = 8.4 Hz, 1H), 8.10 (d, *J* = 8.0 Hz, 1H), 7.76 (t, *J* = 7.6 Hz, 1H), 7.42-7.34 (m, 1H), 7.31 (d, *J* = 8.0 Hz, 1H), 7.18 (dd, *J* = 7.6, 1.6 Hz, 1H), 7.11 (t, *J* = 7.6 Hz, 2H), 6.97-6.80 (m, 2H), 6.75 (d, *J* = 7.6 Hz, 2H), 4.35-4.28 (m, 1H), 4.00 (dd, *J* = 10.4, 9.2 Hz, 1H), 3.80 (dd, *J* = 9.2, 8.8 Hz, 1H), 2.67 (s, 3H), 2.20-2.06 (m, 1H), 1.10 (d, *J* = 6.8 Hz, 3H), 0.96 (d, *J* = 6.8 Hz, 3H);^{13}C NMR (101 MHz, $CDCl_3$):δ163.3, 159.3, 157.1, 150.0, 142.7, 137.40, 137.37, 130.4, 129.8, 128.6, 125.9, 123.3, 123.0, 122.1, 121.7, 120.2, 119.8, 70.8, 54.9, 32.9, 24.4, 19.4, 17.6. HRMS (ESI) calculated for $[M+Na]^+[C_{25}H_{26}N_4ONa]^+$ requires m/z 421.2004, found m/z 421.2000.

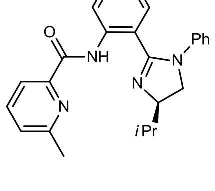

(*R*)-*N*-(2-(4-isopropyl-1-phenyl-4,5-dihydro-1H-imidazol-2-yl)phenyl)-6-methylpicolinamide (ent-L7): Prepared according to a previously reported procedure with a slight modification,[7] a 50 mL flame-dried Schlenk flask was washed with nitrogen three times and charged with CuI (0.1526 g, 0.8 mmol), xylene (12 mL) and *N*, *N'*-dimethylethylenediamine (0.1428 g, 1.6 mmol). After the mixture was stirred at room temperature for 5 min, 6-methylpicolinamide (0.6548 g, 4.8 mmol), **ent-S7** (1.5533 g, 4.0 mmol, 1.0 eq.) and K_3PO_4 (1.69 g, 8.0 mmol) were added to the flask under N_2 atmosphere. The mixture was refluxed for 96 h. After cooling to room temperature, the mixture was filtered and washed with EtOAc. The combined filtrate were concentrated and purified by

column chromatography using PE/EA = 10:1 as the eluent to afford the title compound (0.5870 g, 37% yield) as a light yellow solid. M.P.: 147.3-148.8°C; IR (neat): 2956, 2926, 1680, 1587, 1516, 1446, 1378 cm^{-1}; Optical Rotation: $[\alpha]^{20}_{D}$ = +29.1 (c 1.00, MeOH); ^{1}H NMR (400 MHz, $CDCl_3$):δ12.29 (s, 1H), 8.69 (d, *J* = 8.4 Hz, 1H), 8.10 (d, *J* = 7.6 Hz, 1H), 7.82-7.70 (m, 1H), 7.38 (dd, *J* = 8.4, 7.6 Hz, 1H), 7.30 (d, *J* = 8.0 Hz, 1H), 7.19 (dd, *J* = 7.6, 1.6 Hz, 1H), 7.10 (dd, *J* = 8.4, 7.6 Hz, 2H), 7.00-6.86 (m, 2H), 6.75 (d, *J* = 7.6 Hz, 2H), 4.40-4.25 (m, 1H), 4.00 (dd, *J* = 10.4, 9.6 Hz, 1H), 3.80 (dd, *J* = 9.2, 8.8 Hz, 1H), 2.66 (s, 3H), 2.16-2.07 (m, 1H), 1.10 (d, *J* = 6.8 Hz, 3H), 0.96 (d, *J* = 6.8 Hz, 3H); ^{13}C NMR (101 MHz, $CDCl_3$):163.3, 159.2, 157.0, 150.0, 142.7, 137.4, 137.3, 130.4, 129.8, 128.6, 125.9, 123.2, 122.9, 122.1, 121.6, 120.1, 119.8, 70.7, 54.9, 32.8, 24.3, 19.3, 17.6; HRMS (ESI) calculated for $[M+Na]^{+}[C_{25}H_{26}N_4ONa]^{+}$ requires m/z 421.2004, found m/z 421.2001.

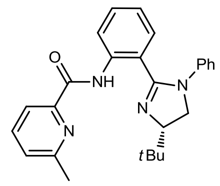

(*S*)-*N*-(2-(4-(tert-butyl)-1-phenyl-4,5-dihydro-1H-imidazol-2-yl)phenyl)-6-methylpicolinamide (L8): Prepared according to a previously reported procedure with a slight modification,[7] a 50 mL flame-dried Schlenk flask was washed with nitrogen three times and charged with CuI (0.0782 g, 0.4 mmol), toluene (15 mL) and *N, N'*-dimethylethylenediamine (80 uL, 0.90 g/mL, 0.8 mmol). After the mixture was stirred at room temperature for 5 min, 6-methylpicolinamide (0.3258 g, 2.4 mmol), **S8** (0.8059 g, 2.0 mmol, 1.0 eq.) and K_3PO_4 (0.8499 g, 4.0 mmol) were added to the flask under N_2 atmosphere. The mixture was refluxed for 36 h. After cooling to room temperature, the mixture was filtered and washed with EtOAc. The combined filtrate were concentrated and purified by column chromatography using PE/EA = 10:1 and PE/EA = 5:1 as the eluent to afford the title compound (0.3830 g, 47% yield) as a light yellow solid. M.P.: 150.3-151.9 °C; IR (neat): 3063, 2954, 1680, 1591, 1515, 1445, 1377 cm^{-1}; Optical Rotation: $[\alpha]^{20}_{D}$ = -22.7 (c 1.08, $CHCl_3$); ^{1}H NMR (400 MHz, $CDCl_3$):δ 11.96 (s, 1H), 8.58 (d, *J* = 8.4 Hz, 1H), 8.08 (d, *J* = 7.6 Hz, 1H), 7.76 (t, *J* = 7.6 Hz, 1H), 7.42-7.35 (m, 1H), 7.31 (d, *J* = 7.6 Hz, 1H), 7.22 (dd, *J* = 7.6, 1.2 Hz, 1H), 7.10 (t, *J* = 8.0 Hz, 2H), 6.97-6.90 (m, 2H), 6.75 (d, *J* = 7.6 Hz, 2H), 4.21 (dd, *J* = 10.4, 9.2 Hz, 1H), 3.96-3.80 (m, 2H), 2.67 (s, 3H), 1.03 (s, 9H); ^{13}C NMR (101 MHz, $CDCl_3$):δ163.4, 159.2, 157.1, 150.0, 142.8, 137.3, 137.1, 130.3, 129.7, 128.6, 126.0, 123.25, 123.17, 122.1, 120.8, 119.9, 74.5, 54.0, 34.2, 26.1, 24.6; HRMS (ESI) calculated for $[M+Na]^{+}[C_{26}H_{28}N_4ONa]^{+}$ requires m/z

435.2161, found m/z 435.2155.

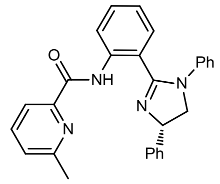

(*S*)-*N*-(2-(1,4-diphenyl-4,5-dihydro-1H-imidazol-2-yl)phenyl)-6-methylpicolinamide (L9): Prepared according to a previously reported procedure with a slight modification,[7] a 50 mL flame-dried Schlenk flask was washed with nitrogen three times andcharged with CuI (0.0380 g, 0.2 mmol), dioxane (8 mL) and ethylene diamine (15 uL, 0.90 g/mL, 0.2 mmol). After the mixture was stirred at room temperature for 5 min, 6-methylpicolinamide (0.1708 g, 1.3 mmol), **S9** (0.4245 g, 1.0 mmol, 1.0 eq.) and K_3PO_4 (0.4338 g, 2.0 mmol) were added to the flask under N_2 atmosphere. The mixture was refluxed for 48 h. After cooling to room temperature, the mixture was filtered and washed with EtOAc. The combined filtrate were concentrated and purified by column chromatography using PE/EA = 10:1 as the eluent to afford the title compound (0.2361 g, 53% yield) as a light yellow solid. M.P.: 178.0-180.2 ^{o}C; IR (neat): 3062, 2876, 1679, 1585, 1517, 1446, 1377 cm^{-1};Optical Rotation: $[\alpha]^{20}_{D}$ = +71.0 (c 1.06, $CHCl_3$);^{1}H NMR (400 MHz, $CDCl_3$): δ12.84 (s, 1H), 8.75 (d, *J* = 8.4 Hz, 1H), 8.06 (d, *J* = 7.6 Hz, 1H), 7.72 (t, *J* = 7.6 Hz, 1H), 7.45-7.37 (m, 3H), 7.30-7.16 (m, 5H), 7.13 (t, *J* = 8.0 Hz, 2H), 6.99-6.80 (m, 2H), 6.80 (dd, *J* = 8.4, 0.8 Hz, 2H), 5.59 (dd, *J* = 10.4, 8.8 Hz, 1H), 4.44 (dd, *J* = 10.8, 9.6 Hz, 1H), 3.94 (dd, *J* = 9.2, 8.8 Hz, 1H), 2.21 (s, 3H);^{13}C NMR (101 MHz, $CDCl_3$):δ163.5, 160.8, 157.3, 149.8, 143.6, 142.8, 138.0, 137.3, 130.9, 130.2, 128.8, 128.5, 127.3, 126.8, 125.7, 123.7, 122.8, 122.5, 121.5, 119.5, 119.2, 68.4, 61.0, 23.7; HRMS (ESI) calculated for $[M+Na]^{+}[C_{28}H_{24}N_4ONa]^{+}$ requires m/z 455.1848, found m/z 455.1842.

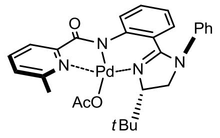

(L8-H) PdOAc. A 25 mL Schlenk flask was charged with 0.1131 g (0.5mmol) of $Pd(OAc)_2$, 8 mL of THF and 0.2263 g (0.55mmol) of **L8** under atmosphere of nitrogen. The mixture was stirred at room temperature for 17 h. The resulting solvent was concentrated *in vacuo* and the resulting residue was washed with ether and dried *in vacuo* to afford 0.2290 g (3.6mmol, 72% yield) of the title compound as a yellow powder. ^{1}H NMR (400 MHz, $CDCl_3$): δ8.18 (d, *J* = 8.4 Hz, 1H), 7.96 (d, *J* = 7.2 Hz, 1H), 7.85 (dd, *J* = 7.6, 8.0 Hz, 1H), 7.28-7.19 (m, 4H), 7.12 (dd, *J* = 7.6, 7.2 Hz, 1H), 6.98 (d, *J* = 8.0 Hz, 1H), 6.89 (d, *J* = 7.6 Hz, 2H), 6.61 (t, *J* = 7.6 Hz, 1H), 4.26 (t, *J* = 10.0 Hz, 1H), 3.88 (d, *J* =

10.4 Hz, 1H), 3.81 (d, *J* = 9.6 Hz, 1H), 2.63 (s, 3H), 2.04 (s, 3H), 1.16 (s, 9H); ^{13}C NMR (101 MHz, $CDCl_3$): δ177.6, 169.7, 161.1, 160.0, 156.5, 145.6, 142.3, 139.0, 132.1, 131.9, 129.1, 128.4, 125.5, 124.2, 124.0, 123.4, 121.0, 118.0, 77.2, 68.7, 55.8, 35.2, 26.1, 24.1, 23.5.

CCDC number of (**L8-H**) ᵽdOAc:1588226

X-ray structure of (**L8-H**) ᵽdOAc

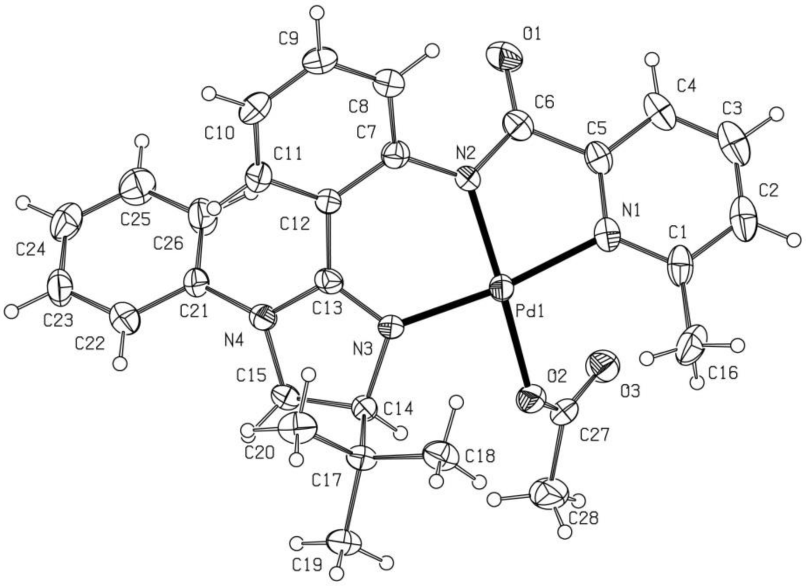

Supplementary Table 3. Crystal data and structure refinement of compound (**L8**-H) ᵽdOAc

Crystal data	(**L8**-H) ᵽdOAc
Empirical formula	C_{28} H_{30} N_4O_3Pd
Formula weight	576.96
Temperature (K)	293
Wavelength (Å)	0.71073
Crystal system	orthorhombic
space group	P 21 21 21
a (Å)	9.827 (2)
b (Å)	11.278 (2)
c(Å)	23.384 (5)
alpha	90

beta	90
gamma	90
Volume (Å^3)	2591.5 (9)
Z	4
Calculated density (mg/m^3)	1.479
Absorption coefficient (mm^{-1})	0.753
F(000)	1184.0
Theta range	2.884 - 25.349
Limiting indices	-11<=h<=9, -13<=k<=13, -28<=l<=23
Absorption correction	multi-scan
Max. and min. transmission	1.000 and 0.931
Data/restraints/parameters	4733 / 0 / 330
Goodness-of-fit on F^2	1.037
Final R indices [I>4sigma(I)]	R = 0.0369, wR = 0.0768
R indices (all data)	R = 0.0496, wR = 0.0836

Procedures for Synthesis of Starting Materials

General procedure A for preparation of alkenes: Under N_2 atmosphere, a 100 mL flame-dried Schlenk flask was charged with $RP^+Ph_3X^-$ (12mmol, 1.2 eq.) and 30 mL of THF. *n*-BuLi (14.4 mmol, 5.8 mL, 2.5 M in THF) was added dropwise over 10 min at -20 ^oC. After stirred at -20 ^oC for 40 min, the corresponding aldehyde in 10 mL of THF was added dropwise. Then the mixture was warmed to room temperature slowly and stirred for another 18 h. After completion of the reaction, the reaction mixture was quenched with saturated solution of NH_4Cl (25~30 mL) under ice cooling and the organic layer was separated. The aqueous layer was extracted with EtOAc (2x30 mL). The combined organic phases were dried over anhydrous Na_2SO_4 and concentrated in vacuo. The resulting crude product was purified by column chromatography (silica gel, 200-300 mesh) to afford the corresponding alkene (a mixture of *E/Z* isomer and the ratio is unknown).

General procedures B for preparation of alkenes: Under N_2 atmosphere, a 100 mL flame-dried Schlenk flask was charged with $RP^+Ph_3X^-$ (12mmol, 1.2 eq.), NaH (14.4 mmol) and 30 mL of THF. The mixture was refluxed for 2 h. Then the corresponding aldehyde in 10 mL of THF was

added dropwise at 0 ^oC. Then the mixture was warmed to room temperature and refluxed for 4-12 h. After completion of the reaction, the reaction mixture was quenched with saturated solution of NH_4Cl (25~30 mL) under ice cooling and the organic layer was separated. The aqueous layer was exracted with EtOAc (2x20 mL). The combined organic phases were dried over anhydrous Na_2SO_4 and concentrated in vacuo. The resulting crude product was purified by column chromatography (silica gel, 200-300 mesh) to afford the corresponding alkene (a mixture of *E/Z* isomer and the ratio is unknown).

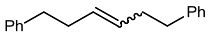

(*E*/*Z*)-1,6-diphenylhex-3-ene (1a): Prepared from phenylpropyl aldehyde according to the general procedure A. *Z/E* ratio: 4.1/1, 67% yield, colorless oil. ^{1}H NMR (400 MHz, $CDCl_3$): δ 7.30-7.22 (m, 4H), 7.21-7.12 (m, 6H), 5.49-5.37 (m, 2H), 2.68-2.62 (m, 0.80H), 2.62-2.53 (m, 3.26H), 2.35-2.25 (m, 4H). All the spectroscopic data were in agreement with the reported ones.[8]

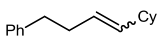

(*E*/*Z*)-(4-cyclohexylbut-3-en-1-yl)benzene (1b): Prepared from phenyl- propyl aldehyde according to the general procedure A. *Z/E* ratio: 4.7/1, 64% yield, colorless oil. ^{1}H NMR (400 MHz, $CDCl_3$): δ 7.30-7.23 (m, 2H), 7.20-7.14 (m, 3H), 5.41-5.36 (m, 0.34H), 5.33-5.18 (m, 1.64H),2.68-2.62 (m, 2H), 2.39-2.32 (m, 1.70H), 2.31-2.25 (m, 0.36H), 2.23-2.12 (m, 0.84H), 1.94-1.83 (m, 0.20H), 1.74-1.58 (m, 3.46H), 1.54-1.45 (m, 1.80H), 1.30-0.0.94 (m, 5H). All the spectroscopic data were in agreement with the reported ones.[9]

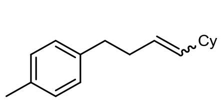

(*E*/*Z*)-1-(4-cyclohexylbut-3-en-1-yl)-4-methylbenzene (1c): Prepared from 3-(*p*-tolyl)propanal according to the general procedure A. *Z/E* ratio: 4.0/1, 42% yield, colorless oil. IR (neat): 3005, 2925, 2851, 1515, 1446 cm^{-1}; ^{1}H NMR (400 MHz, $CDCl_3$): δ 7.12-7.04 (m, 4H), 5.42-5.17 (m, 2H), 2.65-2.58 (m, 2H), 2.39-2.12 (m, 5.81H), 1.94-1.82 (m, 0.19H), 1.75-1.58 (m, 3.45H), 1.53-1.47 (m, 1.58H), 1.31-0.85 (m, 5H).HRMS (ESI) calculated for $[M+H]^+[C_{17}H_{25}]^+$ requires m/z 229.1956, found m/z 229.1951.

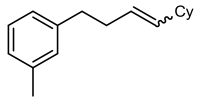

(*E*/*Z*)-1-(4-cyclohexylbut-3-en-1-yl)-3-methylbenzene (1d): Prepared from 3-(*m*-tolyl)propanal according to the general procedure A. *Z/E* ratio: 3.4/1, 26% yield, colorless oil. IR (neat): 3005, 2924, 2851, 1607, 1488, 1447 cm^{-1}; ^1H NMR (400 MHz, $CDCl_3$): δ 7.19-7.12 (m, 1H), 7.03-6.95 (m, 3H), 5.45-5.36 (m, 0.45H), 5.33-5.18 (m, 1.53H), 2.66-2.59 (m, 2H), 2.40- 2.14 (m, 5.83H), 2.01-1.82 (m, 0.27H), 1.73-1.58 (m, 3.43H), 1.55-1.46 (m, 1.65H), 1.31-0.84 (m, 5H).HRMS (ESI) calculated for $[M+H]^+[C_{17}H_{25}]^+$ requires m/z 229.1956, found m/z 229.1949.

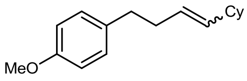

(*E*/*Z*)-1-(4-cyclohexylbut-3-en-1-yl)-4-methoxybenzene (1e): Prepared from 3-(4-methoxyphenyl)propanal according to the general procedure A. *Z/E* ratio: 2.9/1, 52% yield, colorless oil. IR (neat): 3002, 2924, 2849, 1513, 1450, 1247 cm^{-1}; ^1H NMR (400 MHz, $CDCl_3$): δ 7.13-7.06 (m, 2H), 6.85-6.80 (m, 2H), 5.43-5.36 (m, 0.50H), 5.32-5.17 (m, 1.46H), 3.78 (s, 3H), 2.64-2.55 (m, 2H), 2.37-2.28 (m, 1.50H), 2.26-2.10 (m, 1.25H), 1.95-1.84 (m, 0.28H), 1.74-1.46 (m, 5H), 1.30-0.84 (m, 5H). HRMS (ESI) calculated for $[M+Na]^+[C_{17}H_{24}ONa]^+$ requires m/z 267.1725, found m/z 267.1732.

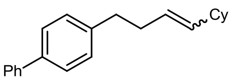

(*E*/*Z*)-4-(4-cyclohexylbut-3-en-1-yl)-1,1'-biphenyl (1f): Prepared from 3-([1,1'-biphenyl]-4-yl)propanal according to the general procedure A. *Z/E* ratio: 4.3/1, 62% yield, colorless oil.IR (neat): 2921, 2848, 1486, 1447 cm^{-1}; ^1H NMR (400 MHz, $CDCl_3$): δ 7.62-7.54 (m, 2H), 7.53-7.47 (m, 2H), 7.46-7.37 (dd, *J* = 8.0, 7.2 Hz, 2H), 7.34-7.20 (m, 3H), 5.49-5.0 (m, 2H), 2.70 (t, *J* = 8.0 Hz, 2H), 2.48-2.36 (m, 1.64H), 2.35-2.28 (m, 0.36H), 2.26-2.12 (m, 0.81H), 1.96-1.84 (m, 0.19H), 1.74-1.41 (m, 5H), 1.30-0.86 (m, 5H).HRMS (ESI) calculated for $[M+H]^+[C_{22}H_{27}]^+$ requires m/z 291.2113, found m/z 291.2120.

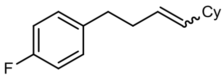

(*E*/*Z*)-1-(4-cyclohexylbut-3-en-1-yl)-4-fluorobenzene (1g): Prepared from 3-(4-fluorophenyl)propanal according to the general procedure A. *Z/E* ratio: 3.7/1, 33% yield, colorless oil. IR (neat): 3005, 2926, 2852, 1604, 1511, 1449, 1227 cm^{-1};^1H NMR (400 MHz, $CDCl_3$): δ 7.17-7.08 (m, 2H), 7.00-6.91 (m, 2H), 5.38-5.34 (m, 0.42H), 5.30-5.17 (m, 1.54H), 2.62 (t, *J* = 7.6 Hz, 2H), 2.37-2.30 (m, 1.60H), 2.28-2.22 (m, 0.43H), 2.21-2.10 (m, 0.79H), 1.97-1.79 (m, 0.25H), 1.73-1.58 (m, 3.47H), 1.52-1.43 (m, 1.61H), 1.30-0.84 (m, 5H). ^{19}F

NMR (376 MHz, $CDCl_3$): δ -118.0, -118.1.HRMS (ESI) calculated for $[M+Na]^+[C_{16}H_{21}FNa]^+$ requires m/z 255.1525, found m/z 255.1532.

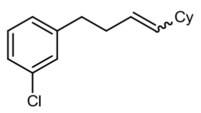

(*E*/*Z*)-1-chloro-3-(4-cyclohexylbut-3-en-1-yl)benzene (1h): Prepared from 3-(3-chlorophenyl)propanal according to the general procedure A. *Z*/*E* ratio: 4.0/1, 49% yield, colorless oil. IR (neat): 3003, 2926, 2851, 1598, 1478,1443 cm^{-1}; ^1H NMR (400 MHz, $CDCl_3$): δ 7.22-7.12 (m, 3H), 7.08-7.01 (m, 1H), 5.37-5.33 (m, 0.39H), 5.29-5.18 (m, 1.55H), 2.62 (dd, J = 8.0, 6.8 Hz, 1H), 2.39-2.30 (m, 1.62H), 2.29-2.22 (m, 0.42H), 2.21-2.09 (m, 0.79H), 1.93-1.82 (m, 0.22H), 1.74-1.57 (m, 3.48H), 1.51-1.43 (m, 1.61H), 1.31-0.94 (m, 5H).HRMS (ESI) calculated for $[M+H]^+[C_{16}H_{22}Cl]^+$ requires m/z 249.1410 found m/z 249.1417.

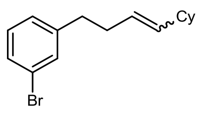

(*E*/*Z*)-1-bromo-3-(4-cyclohexylbut-3-en-1-yl)benzene (1i): Prepared from 3-(3-bromophenyl)propanal according to the general procedure A. *Z*/*E* ratio: 3.5/1, 67% yield, colorless oil. IR (neat): 3000, 2922, 2849, 1567,1447 cm^{-1}; ^1H NMR (400 MHz, $CDCl_3$): δ 7.38-7.27 (m, 2H), 7.16-7.06 (m, 2H), 5.40-5.30 (m, 0.44H), 5.29-5.17 (m, 1.56H), 2.65-2.58 (m, 2H), 2.38-2.31 (m, 1.62H), 2.29-2.23 (m, 0.46H), 2.20-2.09 (m, 0.78H), 1.92-1.83 (m, 0.22H), 1.71-1.56 (m, 3.44H), 1.51-1.43 (m, 1.62H), 1.29-0.95 (m, 5H); HRMS (EI) calculated for$[C_{16}H_{21}Br]^+$ requires m/z 292.0827, found m/z 292.0830.

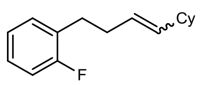

(*E*/*Z*)-1-(4-cyclohexylbut-3-en-1-yl)-2-fluorobenzene (1j): Prepared from 3-(2-fluorophenyl)propanal according to the general procedure A. *Z*/*E* ratio: 4.6/1, 64% yield, colorless oil. IR (neat): 3001, 2923, 2850, 1492, 1449, 1229 cm^{-1}; ^1H NMR (400 MHz, $CDCl_3$): δ 7.23-7.11 (m, 2H), 7.08-6.94 (m, 2H), 5.47-5.34 (m, 0.36H), 5.34-5.14 (m, 1.64H), 2.68 (t, J = 7.6 Hz, 2H), 2.42-2.32 (m, 1.64H), 2.31-2.24 (m, 0.37H), 2.23-2.04 (m, 0.82H), 1.92-1.83 (m, 0.18H), 1.72-1.57 (m, 3.43H), 1.50-1.42 (m, 1.66H), 1.29-1.10 (m, 3H), 1.06-0.91 (m, 2H); ^{19}F NMR: (376 MHz, $CDCl_3$) : δ -118.9; HRMS (EI) calculated for$[C_{16}H_{21}F]^+$ requires m/z 232.1627, found m/z 232.1631.

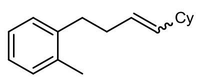

(*E*/*Z*)-1-(4-cyclohexylbut-3-en-1-yl)-2-methylbenzene (1k): Prepared from 3-(*o*-tolyl)propanal according to the general procedure A. *Z*/*E* ratio: 4.7/1, 65% yield, colorless oil. IR (neat): 3002, 2923, 2850, 1492, 1449 cm^{-1}; ^1H NMR (400 MHz, $CDCl_3$): δ7.19-7.01 (m, 4H), 5.48-5.37 (m, 0.35H), 5.37-5.15 (m, 1.65H), 2.67-2.59 (m, 2H), 2.36-2.15 (m, 5.84H), 1.94-1.84 (m, 0.17H), 1.74-1.57 (m, 3.40H), 1.56-1.44 (m, 1.71H), 1.30-0.94 (m, 5H); HRMS (ESI) calculated for $[M+H]^+[C_{17}H_{25}]^+$ requires m/z 229.1956, found m/z 229.1949.

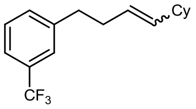

(*E*/*Z*)-1-(4-cyclohexylbut-3-en-1-yl)-3-(trifluoromethyl)benzene (1l): Prepared from 3-(3-(trifluoromethyl)phenyl)propanal according to the general procedure A. *Z*/*E* ratio: 3.6/1, 57% yield, colorless oil. IR (neat): 3005, 2930, 2853, 1447, 1329, 1167, 1129 cm^{-1}; ^1H NMR (400 MHz, $CDCl_3$): δ 7.47-7.31 (m, 4H), 5.39-5.32 (m, 0.43H), 5.30-5.18 (m, 1.54H), 2.72 (t, *J* = 7.6 Hz, 2H), 2.43-2.33 (m, 1.54H), 2.33-2.26 (m, 0.45H), 2.19-2.06 (m, 0.77H), 1.93-1.82 (m, 0.25H), 1.75-1.56 (m, 3.49H), 1.48-1.38 (m, 1.54H), 1.30-0.92 (m, 5H). ^{19}F NMR (376 MHz, $CDCl_3$): δ -62.5.HRMS (ESI) calculated for $[M+H]^+[C_{17}H_{22}F_3]^+$ requires m/z 283.1674 found m/z 283.1681.

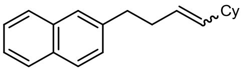

(*E*/*Z*)-2-(4-cyclohexylbut-3-en-1-yl)naphthalene (1m): Prepared from 3-(naphthalen-2-yl)propanal according to the general procedure A. *Z*/*E* ratio: 4.2/1, 56% yield, colorless oil. IR (neat): 3003, 2923, 2850, 1600, 1508, 1446 cm^{-1};^1H NMR (400 MHz, $CDCl_3$): δ 7.83-7.72 (m, 3H), 7.62 (s, 0.79H), 7.60 (s, 0.18H), 7.47-7.36 (m, 2H), 7.35-7.29 (m, 1H), 5.50-5.39 (m, 0.38H), 5.37-5.19 (m, 1.61H), 2.82 (t, *J* = 7.2 Hz, 2H), 2.50-2.42 (m, 1.64H), 2.40-2.33 (m, 0.4H), 2.28-2.12 (m, 0.81H), 1.93-1.83 (m, 0.21H), 1.73-1.56 (m, 3.39H), 1.54-1.44 (m, 1.83H), 1.30-0.85 (m, 5H). HRMS (ESI) calculated for $[M+Na]^+[C_{20}H_{24}Na]^+$ requires m/z 287.1776 found m/z 287.1780.

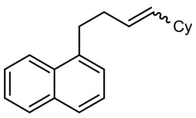

(*E*/*Z*)-1-(4-cyclohexylbut-3-en-1-yl)naphthalene (1n): Prepared from 3-(naphthalen-1-yl)propanal according to the general procedure A. *Z*/*E* ratio: 4.5/1, 67% yield, colorless oil.IR (neat): 2922, 2848, 1597, 1511, 1447 cm^{-1};^1H NMR (400 MHz, $CDCl_3$): δ8.13-7.99 (m, 1H), 7.92-7.79 (m, 1H), 7.78-7.63 (m, 1H), 7.58-7.43 (m, 2H), 7.42-7.35

(m, 1H), 7.35-7.26 (m, 1H), 5.60-4.98 (m, 2H), 3.20-3.02 (m, 2H), 2.57-2.46 (m, 1.66H), 2.46-2.37 (m, 0.34H), 2.22-1.98 (m, 0.81H), 1.97-1.81 (m, 0.18H), 1.76-1.38 (m, 5H), 1.32-0.88 (m, 5H). HRMS (ESI) calculated for $[M+Na]^+[C_{20}H_{24}Na]^+$ requires m/z 287.1776, found m/z 287.1783.

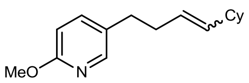

(*E*/*Z*)-5-(4-cyclohexylbut-3-en-1-yl)-2-methoxypyridine (1o): Prepared from 3-(6-methoxypyridin-3-yl)propanal according to the general procedure A. *Z*/*E* ratio: 3.9/1, 79% yield, colorless oil. IR (neat): 3004, 2925, 2851, 1609, 1494, 1452, 1391, 1287 cm^{-1}; ^1H NMR (400 MHz, $CDCl_3$): δ 7.99-7.93 (m, 1H), 7.44-7.35 (m, 1H), 6.67 (d, J = 8.4 Hz, 1H), 5.38-5.33 (m, 0.40H), 5.29-5.18 (m, 1.56H), 3.91 (s, 3H), 2.57 (t, J = 7.2 Hz, 2H), 2.36-2.28 (m, 1.57H), 2.27-2.20 (m, 0.42H), 2.18-2.07 (m, 0.79H), 1.93-1.82 (m, 0.25H), 1.73-1.56 (m, 3.38H), 1.51-1.42 (m, 1.60H), 1.31-0.80 (m, 5H).HRMS (ESI) calculated for $[M+H]^+[C_{16}H_{24}NO]^+$ requires m/z 246.1858, found m/z 246.1865.

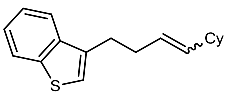

(*E*/*Z*)-3-(4-cyclohexylbut-3-en-1-yl)benzo[b]thiophene (1p): Prepared from 3-(benzo[b]thiophen-3-yl)propanal according to the general procedure A. *Z*/*E* ratio: 4.5/1, 55% yield, colorless oil. IR (neat): 2921, 2848, 1447, 1428cm^{-1}; ^1H NMR (400 MHz, $CDCl_3$): δ 7.85 (d, 1H), 7.80-7.70 (m, 1H), 7.44-7.27 (m, 2H), 7.10 (s, 0.78H), 7.08 (s, 0.17H), 5.50-5.00 (m, 2H), 2.89 (t, 2H), 2.56-2.46 (m, 1.68H), 2.46-2.38 (m, 0.32H), 2.24-2.09 (m, 0.81H), 1.96-1.80 (m, 0.18H), 1.74-1.44 (m, 5H), 1.36-0.82 (m, 5H).HRMS (ESI) calculated for $[M+H]^+[C_{18}H_{23}S]^+$ requires m/z 271.1520, found m/z 271.1526.

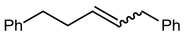

(*E*/*Z*)-pent-2-ene-1,5-diyldibenzene (1q): Prepared from phenylacetaldehyde according to the general procedure A. *Z*/*E* ratio: 5.5/1, 27% yield, colorless oil.^1H NMR (400 MHz, $CDCl_3$): δ 7.32-7.06 (m, 10H), 5.62-5.50 (m, 2H), 3.38-3.30 (m, 2H), 2.75-2.67 (m, 2H), 2.52-2.45 (m, 1.71H), 2.39-2.32 (m, 0.31H). All the spectroscopic data were in agreement with the reported ones.[10]

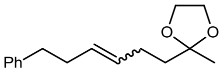

(*E*/*Z*)-2-methyl-2-(6-phenylhex-3-en-1-yl)-1,3-dioxolane (1r): Prepared

from 3-(2-methyl-1,3-dioxolan-2-yl)propanal[11] according to the general procedure A. *Z*/*E* ratio: 2.9/1, 67% yield, colorless oil. IR (neat): 2941, 2878, 1602, 1450, 1376, 1058 cm^{-1};^{1}H NMR (400 MHz, $CDCl_3$): δ 7.31-7.24 (m, 2H), 7.22-7.14 (m, 3H), 5.51-5.30 (m, 2H), 3.96-3.85 (m, 4H), 2.66 (t, *J* = 7.6 Hz, 2H), 2.40-2.33 (m, 1.47H), 2.32-2.25 (m, 0.50H), 2.12-2.03 (m, 2H), 1.72-1.65 (m, 0.53H), 1.63-1.56 (m, 1.53H), 1.31 (s, 0.80H), 1.30 (s, 2.22H).HRMS (ESI) calculated for $[M+Na]^+[C_{16}H_{22}O_2Na]^+$ requires m/z 269.1517, found m/z 269.1537.

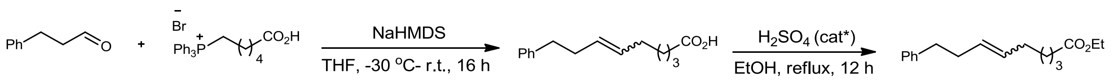

1ss 1s

(*E*/*Z*)-ethyl 9-phenylnon-6-enoate (1s): To a dry THF (30 mL) suspension of (6-carboxyhexyl) triphenylphosphonium bromide (13.76 g, 30 mmol), under N_2 atmosphere at -30 °C, NaHMDS (32.5 mL, 2.0 M in THF) was added dropwise over 20 minutes. After stirred at -30 °C for 1 h, phenylpropyl aldehyde (3.10g, 23 mmol) in 10 mL THF was added dropwise. Then the mixture was warmed to room temperature slowly and stirred for another 15 h. After completion of the reaction, pH was adjusted to 2 ~ 3 by addition of 1N hydrochloric acid, and the organic layer was separated. The aqueous layer was exracted with EtOAc (3x30 mL). The combined organic phases were dried over anhydrous Na_2SO_4 and concentrated in vacuo. The resulting crude product was purified by column chromatography using PE/EA = 5/1 as the eluent to afford the acid **1ss** (3.2339 g, 13.9 mmol, 60% yield) as a light yellow oil. ^{1}H NMR (400 MHz, $CDCl_3$): δ 10.39 (s, 1H), 7.31-7.23 (m, 2H), 7.21-7.13 (m, 3H), 5.50-5.30 (m, 2H), 2.65 (t, *J* = 7.2 Hz, 2H), 2.41-2.25 (m, 4H), 1.98 (q, *J* = 7.2 Hz, 2H), 1.65-1.51 (m, 2H), 1.42-1.23 (m, 2H). All the spectroscopic data were in agreement with the reported ones.[12]

To a 50 mL flame-dried Schlenk flask charged with acid **1ss** (1.8426 g, 8.0 mmol) and EtOH (15 mL) was added a few drops of concentrated sulfuric acid. The reaction mixture was refluxed for 20 h. After cooling to room temperature, the mixture was concentrated and purified by column chromatography using PE/EA = 20:1 as the eluent to afford alkene **1s** (1.5682 g, 6.0 mmol, 76% yield, *Z*/*E* ratio: 4.0/1) as a light yellow oil. IR (neat): 2935, 2859, 1736, 1453, 1179 cm^{-1}; ^{1}H NMR (400 MHz, C_6D_6): δ 7.22-7.15 (m, 2H), 7.12-7.04 (m, 3H), 5.46-5.25 (m, 2H), 4.02-3.90 (m, 2 H), 2.60-2.49 (m, 2H), 2.32-2.20 (m, 2H), 2.15-2.05 (m, 2H), 1.91-1.84 (m, 2H), 1.60-1.45 (m, 2 H), 1.28-1.21 (m, 0.41H), 1.20-1.10 (m, 1.64H), 1.02-0.92 (m, 3H). HRMS (ESI) calculated for

$[M+H]^+[C_{17}H_{25}O_2]^+$ requires m/z 261.1855, found m/z 261.1854.

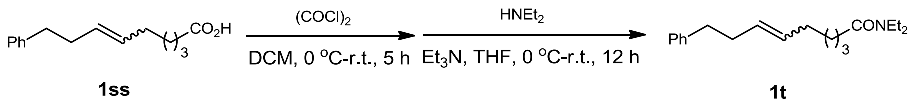

(*E*/*Z*)-*N,N*-diethyl-7-phenylhept-4-enamide (1t): acid **1ss** (2.32 g, 10.0 mmol) was dissolved in dichloromethane (10 mL) in a 50 mL round bottomed flask and cooled on an ice bath. Oxalyl chloride (1.70 mL, 20.0 mmol) and DMF (5 drops) was added in sequence under stirring, and the reaction was allowed to come to room temperature. After 5 hours the reaction mixture was evaporated to give the crude product without purification and used in the next step.

Under N_2 atmosphere, a 50 mL flame-dried Schlenk flask was charged with chloride and 30 mL THF. To this reaction mixture, Et_3N (2.8 mL, 0.73 g/mL, 20.0 mmol) and Diethylamine (1.5 mL, 0.71 g/mL, 15 mmol) was added in sequence. Then the mixture was warmed to room temperature and stirred for 12 h. After completion of the reaction, the reaction mixture was quenched with saturated solution of NH_4Cl (25-30 mL) and the organic layer was separated. The aqueous layer was exracted with EtOAc (3x30 mL). The combined organic phases were dried over anhydrous Na_2SO_4 and concentrated in vacuo. The resulting crude product was purified by column chromatography (silica gel, 200-300 mesh) using PE/EA = 5/1 as the eluent to afford the alkene **1t** (1.7729 g, 62% yield for two steps, *Z*/*E* ratio: 4.0/1) as a yellow oil. IR (neat): 2932, 2855, 1642, 1455, 1430 cm^{-1}; ^1H NMR (400 MHz, C_6D_6): δ 7.22-7.16 (m, 2H), 7.12-7.04 (m, 3H), 5.47-5.39 (m, 2H), 3.22 (q, J = 7.2 Hz, 2H), 2.71 (q, J = 7.2 Hz, 2H), 2.59-2.53 (m, 2H), 2.35-2.21 (m, 2H), 2.06-1.93 (m, 4H), 1.79-1.68 (m, 2H), 1.42-1.35 (m, 0.40H), 1.35-1.25 (m, 1.62H), 0.98 (t, J = 7.2 Hz, 3H), 0.70 (t, J = 7.2 Hz, 3H).HRMS (ESI) calculated for $[M+Na]^+[C_{19}H_{29}NONa]^+$ requires m/z 310.2147, found m/z 310.2156.

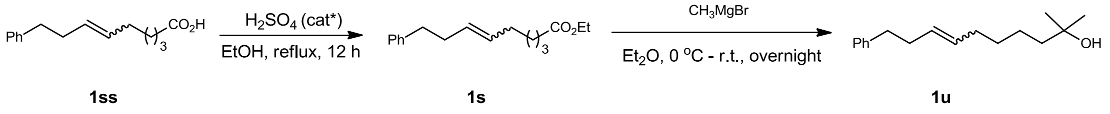

(*E*/*Z*)-2-methyl-10-phenyldec-7-en-2-ol (1u): To a 50 mL flame-dried Schlenk flask charged with acid **1ss** (1.16 g, 5.0 mmol) and EtOH (10 mL) was added two drops of concentrated sulfuric acid. The reaction mixture was refluxed for 20 h. After cooling to room temperature, the mixture was concentrated and purified by column chromatography using PE/EA = 20:1 as the eluent to afford

ester as a light yellow oil.

Under N_2 atmosphere, a 50 mL flame-dried Schlenk flask was charged with ester and 10 mL Et_2O. To this reaction mixture, CH_3MgBr (4.2 mL, 3.0 mol/L) was added slowly at 0 ^oC. Then the mixture was warmed to room temperature and stirred for 20 h. After completion of the reaction, the reaction mixture was quenched with saturated solution of NH_4Cl (25-30 mL) and the organic layer was separated. The aqueous layer was exracted with EtOAc (3x20 mL). The combined organic phases were dried over anhydrous Na_2SO_4 and concentrated in vacuo. The resulting crude product was purified by column chromatography (silica gel, 200-300 mesh) using PE/EA = 10/1 as the eluent to afford the alkene (1.1181 g, 90% yield for two steps, *Z*/*E* ratio: 4.0/1) as a colorless oil.^{1}H NMR (400 MHz, CD_2Cl_2) δ 7.32-7.14 (m, 2H), 7.24-7.14 (m, 3H), 5.49-5.44 (m, 0.40H), 5.43-5.36 (m, 1.59H), 2.66 (t, *J* = 7.6 Hz, 2H), 2.42-2.22 (m, 2H), 2.08-1.95 (m, 2H), 1.49-1.24 (m, 7H), 1.18 (s, 6H). All the spectroscopic data were in agreement with the reported ones.[13]

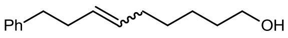

9-phenylnon-6-en-1-ol (1v): To a dry THF (30 mL) suspension of $LiAlH_4$ (1.25 g, 32.9 mmol), under N_2 atmosphere with ice cooling, acid **1ss** (5.10 g, 22.0 mmol) in THF (30 mL) was added dropwise over 10 minutes. After stirred at room temperature for 21 h, the reaction mixture was quenched with saturated solution of Na_2SO_4 (10 mL) under ice cooling. The mixture was extracted with EtOAc (3x60 mL) and the organic phases were dried over anhydrous Na_2SO_4 and concentrated in vacuo. The resulting crude product was purified by column chromatography using PE/EA = 5/1 as the eluent to afford alkene **1v** (4.2942 g, 19.7 mmol, 90% yield, *Z*/*E* ratio: 3.9/1) as a colorless oil. IR (neat): 3349, 2931, 2856, 1496, 1454 cm^{-1}; ^{1}H NMR (400 MHz, C_3D_6O): *δ* 7.30-7.12 (m, 5H), 5.46-5.32 (m, 2H), 3.56-3.40 (m, 3H), 2.65 (t, *J* = 7.6 Hz, 2H), 2.40-2.32 (m, 1.61H), 2.32-2.25 (m, 0.41H), 2.05-1.96 (m, 2H), 1.55-1.44 (m, 2H), 1.40-1.24 (m, 4H); HRMS (EI) calculated for$[C_{15}H_{22}O]^+$ requires m/z 218.1671, found m/z 218.1675.

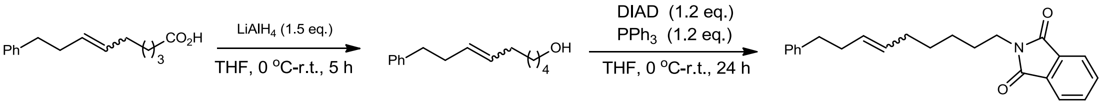

1ss **1v** **1w**

(*E*/*Z*)-2-(9-phenylnon-6-en-1-yl)isoindoline-1,3-dione (1w): To a dry THF (30 mL) suspension of $LiAlH_4$ (1.42 g, 37.5 mmol), under N_2 atmosphere with ice cooling, acid (5.80 g, 25.0 mmol) in

THF (20 mL) was added dropwise over 10 minutes. After stirred at room temperature for 5 h, the reaction mixture was quenched with saturated solution of Na_2SO_4 (10 mL) under ice cooling. Then the mixture was filtered and the filtrate was evaporated to give the crude alcohol without purification and used in the next step.

A 50 mL oven-dried round-bottom flask was charged with alcohol **1v** (1.30 g, 6.0 mmol), phthalamide (1.08 g, 7.2 mmol), Ph_3P (1.88 g, 7.2 mmol) and THF (20 mL). The flask was cooled to 0 ^{o}C, and DIAD (1.46 g, 7.2 mmol) was added dropwise. The mixture was allowed to warm to room temperature and was stirred for 24 h. Then the reaction mixture was quenched with saturated NH_4Cl (aq). The mixture was extracted with EtOAc (2x30 mL) and the organic phases were dried over anhydrous Na_2SO_4 and concentrated in vacuo. The resulting crude product was purified by column chromatography using PE/EA = 5/1 as the eluent to afford alkene **1w** (1.4980 g, 4.3 mmol, 72% yield, *Z*/*E* ratio: 4.0/1) as a colorless oil. IR (neat): 2935, 2856, 1772, 1714, 1399, 1365 cm^{-1};^{1}H NMR (400 MHz, CD_3CN): δ 7.84-7.70 (m, 4H), 7.28-7.10 (m, 5H), 5.47-5.29 (m, 2H), 3.58 (t, *J* = 7.2 Hz, 2H), 2.65-2.55 (m, 2H), 2.35-2.26 (m, 1.60H), 2.26-2.19 (m, 0.40H), 2.20-1.88 (m, 2H), 1.66-1.54 (m, 2H), 1.36-1.20 (m, 4H). HRMS (ESI) calculated for $[M+Na]^+[C_{23}H_{25}NO_2Na]^+$ requires m/z 370.1783, found m/z 370.1785.

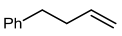

but-3-en-1-ylbenzene (1x): Prepared from phenylpropyl aldehyde according to the general procedure B. 29% yield, colorless oil. ^{1}H NMR (400 MHz, $CDCl_3$): δ 7.31-7.23 (m, 2H), 7.22-7.15 (m, 3H), 5.92-5.80 (m, 1H), 5.09-4.95 (m, 2H), 2.71 (t, J = 7.6 Hz, 2H), 2.42-2.33 (m, 2H).All the spectroscopic data were in agreement with the reported ones.[14]

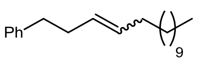

(*E*/*Z*)-hept-3-en-1-ylbenzene (1y): Prepared from phenylpropyl aldehyde according to the general procedure A. *Z*/*E* ratio: 2.8/1, 60% yield, colorless oil. IR (neat): 3017, 2925, 2854, 1496, 1458 cm^{-1};^{1}H NMR (400 MHz, $CDCl_3$): δ 7.31–7.23 (m, 2H), 7.22-7.14 (m, 3H), 5.46-5.41 (m, 0.53H), 5.41-5.32 (m, 1.47H), 2.71-2.61 (m, 2H), 2.42-2.25 (m, 2H), 2.05-1.93 (m, 2H), 1.38-1.20 (m, 18H), 0.88 (t, *J* = 6.8 Hz, 3H).HRMS (ESI) calculated for $[M+H]^+[C_{21}H_{35}]^+$ requires m/z 287.2739, found m/z 287.2746.

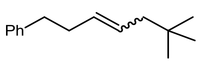 **(*E*/*Z*)-(6,6-dimethylhept-3-en-1-yl)benzene (1z):** Prepared from phenylpropyl aldehyde according to the general procedure A. *Z*/*E* ratio: 6.8/1, 24% yield, colorless oil. IR (neat): 3019, 2952, 2865, 1461, 1364 cm^{-1}; ^{1}H NMR (400 MHz, $CDCl_3$): δ 7.32-7.24 (m, 2H), 7.23-7.14 (m, 3H), 5.55-5.40 (m, 2H), 2.72-2.60 (m, 2H), 2.40-2.29 (m, 2H), 1.89 (d, J = 6.4 Hz, 1.83 H), 1.85(d, J = 6.4 Hz, 0.27H), 0.87 (s, 7.85H), 0.83 (s, 1.22H).HRMS (ESI) calculated for $[M+K]^{+}[C_{15}H_{22}K]^{+}$ requires m/z 241.1359, found m/z 241.1368.

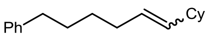 **(*E*/*Z*)-(6-cyclohexylhex-5-en-1-yl)benzene (1aa):** Prepared from 5-phenylpentanal according to the general procedure A. *Z*/*E* ratio: 4.2/1, 57% yield, colorless oil. IR (neat): 3000, 2926, 2852, 1495, 1450 cm^{-1};^{1}H NMR (400 MHz, $CDCl_3$): δ 7.30-7.22 (m, 1H), 7.20-7.13 (m, 2H), 5.36-5.30 (m, 0.38H), 5.28-5.16 (m, 1.60 H), 2.64-2.56 (m, 2H), 2.30-2.17 (m, 0.81H), 2.10-1.96 (m, 2H), 1.93-1.82 (m, 0.21 H), 1.74-1.53 (m, 7H), 1.44-0.96 (m, 7H).HRMS (ESI) calculated for $[M+K]^{+}[C_{18}H_{26}K]^{+}$ requires m/z 281.1672, found m/z 281.1655.

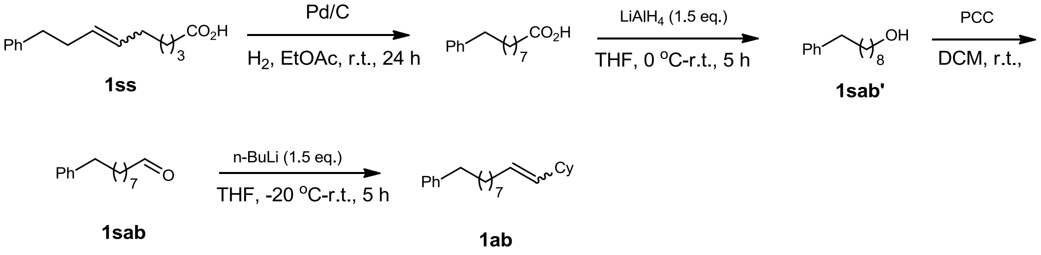

(*E*/*Z*)-(4-cyclohexylbut-3-en-1-yl)benzene (1ab): The mixture of acid **1ss** (9.2 g) and Pd/C (0.9 g, 10 %) in EtOAc (50 mL) was stirred under atmosphere of hydrogen for 26 h at ambient temperature. The suspension was filtrated and the solid was washed with THF for three times. The combined filtrate was concentrated to give crude product, the crude product in THF (20 mL) was added dropwise over 10 minutes to a dry THF (30 mL) suspension of $LiAlH_4$ (2.21 g, 58 mmol), under N_2 atmosphere with ice cooling. After stirred at room temperature for 5 h, the reaction mixture was quenched with saturated solution of Na_2SO_4 (10 mL) under ice cooling. Then the mixture was filtered and the filtrate was evaporated and purified by column chromatography using PE/EA = 10/1 as the eluent to afford the alcohol **1sab'** (8.0787 g, 36.7 mmol, 93% yield for two steps) as a colorless oil.^{1}H NMR (400 MHz, $CDCl_3$): δ7.33-7.26 (m, 2H), 7.23-7.14 (m, 3H), 3.63

(dd, J = 7.8, 7.6 Hz, 2H), 2.65-2.56 (m, 2H), 1.76-1.44 (m, 5H), 1.32 (s, 10H);^{13}C NMR: (101 MHz, $CDCl_3$):δ142.8, 128.3, 128.2, 125.5, 63.0, 35.9, 32.7, 31.5, 29.5, 29.38, 29.36, 29.2, 25.7. ^{1}HNMR data were in agreement with the reported ones.[15]

1.5 equivalents of PCC was suspended in DCM, and then the mixture was cooled in an ice bath. To the cooled suspension, 1 equivalent of alcohol dissolved in DCM was added dropwise. The reaction mixture was warmed to room temperature and stirred for about 5 h. Then the mixture was filtered over silica gel. The filtrate was concentrated and purified by column chromatography using PE/EA = 30/1 as the eluent to afford the aldehyde **1sab** (3.3903 g, 15.5 mmol, 43% yield) as a colorless oil. ^{1}H NMR (400 MHz, $CDCl_3$): 9.75 (t, J = 2.0 Hz, 1H), 7.32-7.22 (m, 2H), 7.22-7.10 (m, 3H), 2.59 (t, J = 7.6 Hz, 2H), 2.40 (dt, J = 7.2, 2.0 Hz, 2H), 1.68-1.56 (m, 4H), 1.38-1.25 (m, 8H); ^{13}C NMR: (101 MHz, $CDCl_3$): δ 202.9, 142.8, 128.4, 128.2, 125.5, 43.9, 35.9, 31.4, 29.24, 29.18, 29.1, 29.0, 22.0. All the spectroscopic data were in agreement with the reported ones.[16]

The alkene **1ab** was prepared from aldehyde **1sab** according to the general procedure A. *Z*/*E* ratio: 4.1/1, 71% yield, colorless oil. IR (neat): 2925, 2853, 1496, 1454 cm^{-1}; ^{1}H NMR (400 MHz, $CDCl_3$): δ 7.30-7.21 (m, 2H), 7.20-7.13 (m, 3H), 5.36-5.14 (m, 2H), 2.59 (t, J = 7.6 Hz, 2H), 2.30-2.16 (m, 0.82H), 2.10-1.80 (m, 2.20H), 1.77-1.52 (m, 7H), 1.40-0.96 (m, 15H). HRMS (ESI) calculated for $[M+Na]^+[C_{22}H_{34}Na]^+$ requires m/z 321.2558, found m/z 321.2564.

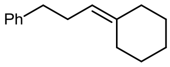

(3-cyclohexylidenepropyl)benzene (1ac): Prepared from cyclohexanone according to the general procedure A. 63% yield, colorless oil. ^{1}H NMR (400 MHz, $CDCl_3$): δ 7.30-7.24 (m, 2H), 7.21-7.14 (m, 3H), 5.11 (t, J = 7.2 Hz, 1H), 2.63 (t, J = 7.6 Hz, 2H), 2.30 (q, J = 7.6 Hz, 2H), 2.10-2.02 (m, 4H), 1.55-1.45 (m, 4H), 1.43-1.34 (m 2H). All the spectroscopic data were in agreement with the reported ones.[17]

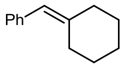

(cyclohexylidenemethyl)benzene (**1ad**): Prepared from cyclohexanone according to the general procedure A. 99% yield, colorless oil. ^{1}H NMR (400 MHz, $CDCl_3$): δ7.36-7.25 (m, 2H), 7.24-7.12 (m, 3H), 6.22 (s, 1H), 2.45-2.32 (m, 2H), 2.31-2.20 (m, 2H), 1.73-1.48 (m, 6H). All the spectroscopic data were in agreement with the reported ones.[18]

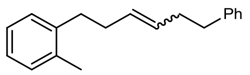

(*E*/*Z*)-1-methyl-2-(6-phenylhex-3-en-1-yl)benzene(1ae): Prepared from 3-(*o*-tolyl)propanal according to the general procedure A. *Z*/*E* ratio: 4.2/1, 55% yield, colorless oil. IR (neat): 3020, 2930, 2857, 1494, 1455 cm^{-1}; ^{1}H NMR (400 MHz, C_6D_6): *δ*7.20-7.14 (m, 3H), 7.12-7.00 (m, 7H), 5.48-5.35 (m, 2H), 2.58-2.50 (m, 0.77H), 2.48-2.40(m, 3.24H), 2.28-2.14 (m, 4H), 2.11 (s, 0.57H), 2.24 (s, 2.24H). HRMS (ESI) calculated for $[M+H]^+[C_{19}H_{23}]^+$ requires m/z 251.1800, found m/z 251.1806.

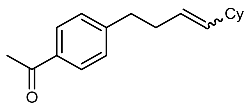

(*E*/*Z*)-1-(4-(4-cyclohexylbut-3-en-1-yl)phenyl)ethanone (s-1af): Prepared from 3-(4-acetylphenyl)propanal[19] according to the general procedure A. *Z*/*E* ratio: 3.9/1, 14% yield, colorless oil. IR (neat): 2923, 2850, 1683, 1607, 1447 cm^{-1}; ^{1}H NMR (400 MHz, C_6D_6) δ 7.80-7.76 (m, 2H), 6.97 (d, *J* = 8.4 Hz, 2H), 5.39-5.20 (m, 2H), 2.54-2.45 (m, 2H), 2.32-2.10 (m, 5.81H), 1.94-1.82 (m, 0.21H), 1.74-1.48 (m, 5H), 1.27-0.94 (m, 5H). HRMS (EI) calculated for$[C_{18}H_{24}O]^+$ requires m/z 256.1827, found m/z 256.1827.

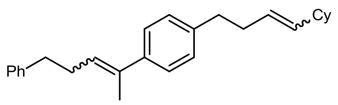

1-((*E*/*Z*)-4-cyclohexylbut-3-en-1-yl)-4-((*E*/*Z*)-5-phenylpent-2-en-2-yl)benzene (1af): Prepared from **s-1af** according to the general procedure A. 66% yield, colorless oil. IR (neat): 2922, 2850, 1604, 1448 cm^{-1}. ^{1}H NMR (400 MHz, $CDCl_3$): δ 7.31-6.99 (m, 9H), 5.83-5.73 (m, 0.19H), 5.52-5.48 (m, 0.81H), 5.42-5.18 (m, 2H), 2.75 (t, *J* = 7.6 Hz, 0.43H), 2.63 (t, *J* = 7.6 Hz, 3.64H), 2.57-2.46 (m, 0.43H), 2.40-2.26 (m, 3.60H), 2.22-2.11 (m, 0.83H), 2.00 (s, 2.45H), 1.94 (s, 0.57H), 1.89-1.82 (m, 0.20H), 1.74-1.55 (m, 3.45H), 1.49-1.42 (m, 1.59H), 1.30-0.86 (m, 5.0H). HRMS (EI) calculated for$[C_{27}H_{34}]^+$ requires m/z 358.2661, found m/z 358.2658.

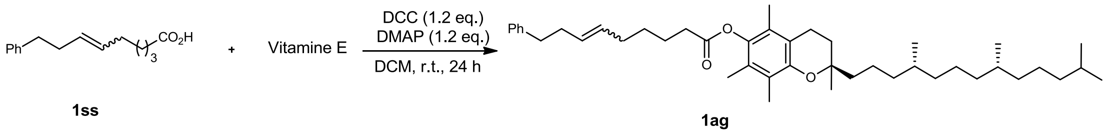

(*E*/*Z*)-(*R*)-2,5,7,8-tetramethyl-2-((4R,8R)-4,8,12-trimethyltridecyl)chroman-6-yl-9-phenylnon-6-enoate (1ag): Under N_2 atmosphere, a 50 mL flame-dried Schlenk flask was charged with acid (4.5 mmol, 1.0456 g), Vitamin E (4.5 mmol, 1.9489 g) and 20 mL DCM. Then DCC (5.4 mmol, 1.1149g) and DMAP (5.4 mmol, 0.6688 g) were added. The mixture was stirred at room

temperature for 24 h. After completion of the reaction, the reaction mixture was filtered and the organic phases were concentrated in vacuo. The resulting crude product was purified by column chromatography (silica gel, 200-300 mesh) using PE/EA = 40/1 as the eluent to afford the alkene (1.8383 g, 63% yield, *Z*/*E* ratio: 3.6/1) as a colorless oil. IR (neat): 2932, 2861, 1755, 1458, 1374, 1136 cm^{-1}; Optical Rotation: $[\alpha]^{20}_{D}$ = +2.4 (c 1.55, $CHCl_3$); ^{1}H NMR (400 MHz, CD_2Cl_2): δ 7.21-7.14 (m, 2H), 7.13-7.04 (m, 3H), 5.42-5.27 (m, 2H), 2.58 (t, *J* = 7.6 Hz, 2H), 2.53-2.44 (m, 4H), 2.32-2.25 (m, 1.63H), 2.25-2.15 (m, 0.45H), 2.02-1.93 (m, 5H), 1.89 (s, 3H), 1.85 (s, 3H), 1.77-1.60 (m, 4H), 1.50-0.97 (m, 26H), 0.80-0.74 (m, 12H). HRMS (ESI) calculated for $[M+Na]^+[C_{44}H_{68}O_3Na]^+$ requires m/z 667.5066, found m/z 667.5056.

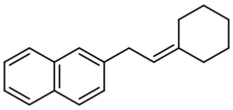

2-(2-cyclohexylideneethyl)naphthalene (1ah): Prepared from cyclohexanone according to the general procedure A. 88% yield, colorless oil. IR (neat): 2926, 2852, 1508, 1446 cm^{-1}; ^{1}H NMR (400 MHz, $CDCl_3$): δ 7.82-7.73 (m, 3H), 7.61 (s, 1H), 7.47-7.37 (m, 2H), 7.32 (dd, *J* = 8.8, 1.2 Hz, 1H), 5.34 (t, *J* = 7.6 Hz, 1H), 3.51 (d, *J* = 7.6 Hz, 2H), 2.33-2.26 (m, 2H), 2.18-2.12 (m, 2H), 1.64-1.54 (m, 6H). ^{13}C NMR: (101 MHz, $CDCl_3$): δ140.9, 139.5, 133.6, 131.9, 127.8, 127.6, 127.4, 126.1, 125.8, 125.0, 119.6, 37.2, 33.5, 28.8, 28.6, 27.9, 26.9. HRMS (ESI) calculated for $[M+H]^+[C_{18}H_{21}]^+$ requires m/z 237.1643, found m/z 237.1646.

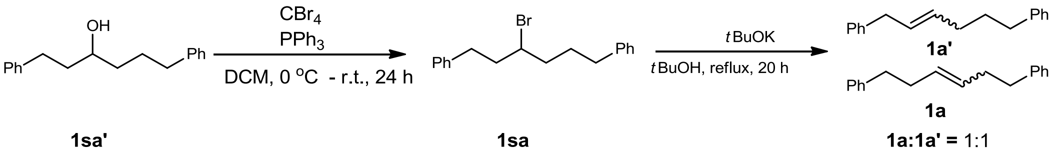

A 250 mL oven-dried three-neck flask was charged with 1,6-diphenylhexan-3-ol[20] (11.67g, 45.9 mmol), tetrabromomethane (18.49 g, 55.8 mmol), DCM (100 mL).Then, the flask was cooled to 0 °C, and triphenylphosphine (14.62 g, 55.7 mmol) was added portionwise. The mixture was allowed to warm to room temperature and was stirred for 24 h. The resulting solution was concentrated and purified by flash column chromatography using PE as the eluent to afford **1sa’** (3.85 g, 12.1 mmol, 26% yield) as a colorless oil. ^{1}H NMR (400 MHz, $CDCl_3$):δ7.32-7.23 (m, 4H), 7.21-7.11 (m, 6H), 4.07-3.85 (m, 1H), 2.92-2.82 (m, 1H), 2.77-2.68 (m, 1H), 2.65-2.54 (m, 2H), 2.15-2.00 (m, 2H), 1.96-1.79 (m, 3H), 1.78-1.68 (m, 1H);^{13}C NMR (101 MHz, $CDCl_3$): δ141.8,

140.9, 128.5, 128.4, 128.34, 128.33, 126.0, 125.8, 57.3, 40.7, 38.6, 35.1, 33.7, 29.2.

To a 50 mL flame-dried Schlenk flask charged with bromide **1sa'** (1.8993 g, 6.0 mmol) and *t*BuOH (10 mL) was added *t*BuOK (1.13 g, 10.0 mmol). The reaction mixture was refluxed for 20 h. After cooling to room temperature, the mixture was concentrated and purified by column chromatography using PE as the eluent to afford alkene (**1a**/**1a'** = 1/1) (0.7733 g, 3.3 mmol, 55% yield) as a colorless oil. ^{1}H NMR (400 MHz, $CDCl_3$): *δ* 7.32-7.23 (m, 4H), 7.21-7.12 (m, 6H), 5.64-5.39 (m, 2H), 3.44-3.26 (m, 1H), 2.71-2.52 (m, 3H), 2.36-2.26 (m, 2H), 2.24-2.02 (m, 1H), 1.79-1.65 (m, 1H).

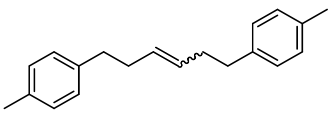

(*E*/*Z*)-1,6-di-p-tolylhex-3-ene (1ai): Prepared from 3-(p-tolyl)propanal according to the general procedure A. *Z*/*E* ratio: 4.7/1, 43% yield, colorless oil. IR (neat): 3005, 2921, 2857, 1515, 1451 cm^{-1}; ^{1}H NMR (400 MHz, $CDCl_3$): δ7.15-6.95 (m, 8H), 5.52-5.46 (m, 0.35H), 5.45-5.34 (m, 1.65H), 2.61 (dd, *J* = 8.4, 7.2 Hz, 0.7H), 2.54 (dd, *J* = 8.0, 7.6 Hz, 3.3H), 2.31 (s, 6H), 2.30-2.24 (m, 4H). HRMS (ESI) calculated for $[M+H]^+[C_{20}H_{25}]^+$ requires m/z 265.1956, found m/z 265.1960.

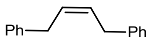

(*Z*)-1,4-diphenylbut-2-ene ((*Z*)-1aj): Prepared according to the literature.[21] 72% yield, colorless oil. ^{1}H NMR (400 MHz, $CDCl_3$) δ 7.34-7.26 (m, 4H), 7.26-7.16 (m, 6H), 5.78-5.66 (m, 2H), 3.52 (d, *J* = 5.2 Hz, 4H). All the spectroscopic data were in agreement with the reported ones.[21]

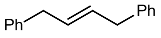

(*E*)-1,4-diphenylbut-2-ene ((*E*)-1aj): Prepared according to the literature.[21] 46% yield (a mixture of alkene and alkane, n_{alkene}/n_{alkane} = 2.8/1), *E*/*Z* ratio: 13/1, colorless oil. ^{1}H NMR (400 MHz, $CDCl_3$) δ 7.32-7.15 (m, 13.4H), 5.70-5.65 (m, 2H), 3.52 (d, *J* = 4.8 Hz, 0.28H), 3.37 (d, *J* = 3.2 Hz, 3.72H). All the spectroscopic data were in agreement with the reported ones.[21]

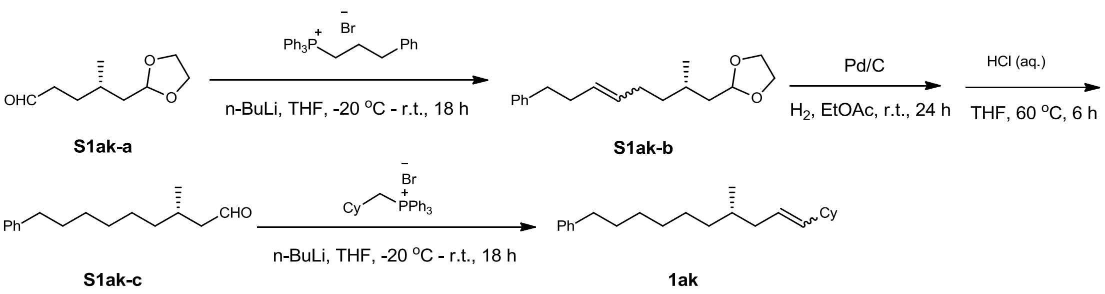

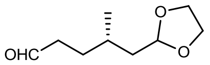

(*S*)-5-(1,3-dioxolan-2-yl)-4-methylpentanal (S1ak-a): Prepared from (-)-Citronellal according to the Literature.[22] 69% yield, colorless oil. IR (neat): 2926, 2722, 1724, 1411, 1139 cm^{-1}; $[\alpha]^{20}_D$ = -4.6 (c 1.06, $CHCl_3$); ^1H NMR (400 MHz, $CDCl_3$): δ 9.78 (d, *J* = 1.2, 1H), 4.90 (t, *J* = 4.4 Hz, 1H), 4.02-3.91 (m, 2H), 3.89-3.80 (m, 2H), 2.55-2.30 (m, 2H), 1.79-1.62 (m, 3H), 1.59-1.47 (m, 2H), 0.97 (d, *J* = 5.6 Hz, 3H); ^{13}C NMR (101 MHz, $CDCl_3$): δ 202.6, 103.4, 64.7, 64.6, 41.5, 40.5, 29.0, 28.9, 19.7. HRMS (EI) calculated for $[C_9H_{16}O_3]^+$ requires m/z 172.1099, found m/z 172.1097.

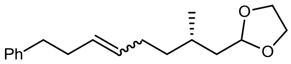

(*S*)-2-(2-methyl-8-phenyloct-5-en-1-yl)-1,3-dioxolane (S1ak- b): Prepared from **S1ak-a** according to the general procedure A. *Z*/*E* ratio: 5.1/1, 80% yield, colorless oil. IR (neat): 2923, 1603, 1495, 1454, 1132 cm^{-1}; $[\alpha]^{20}_D$ = +1.6 (c 0.43, $CHCl_3$); ^1H NMR (400 MHz, $CDCl_3$): δ 7.31-7.24 (m, 2H), 7.23-7.10 (m, 3H), 5.52-5.30 (m, 2H), 4.95-4.81 (m, 1H), 4.02-3.90 (m, 2H), 3.89-3.75 (m, 2H), 2.66 (t, *J* = 7.6 Hz, 2H), 2.43-2.31 (m, 1.67H), 2.31-2.24 (m, 0.33H), 2.10-1.91 (m, 2H), 1.72-1.61 (m, 2H), 1.53-1.44 (m, 1H), 1.42-1.29 (m, 1H), 1.24-1.10 (m, 1H), 1.00-0.89 (m, 3H). HRMS (EI) calculated for $[C_{18}H_{26}O_2]^+$ requires m/z 274.1933, found m/z 274.1936.

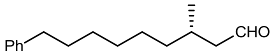

(*S*)-3-methyl-9-phenylnonanal (S1ak-c): To a stirred solution of **S1ak-b** (4.1713 g, 15.2 mmol) in EtOAc (40 mL) were added Pd/C (5%) (0.8241g, 20% wt/Pd) at room temperature. The resulting mixture was stirred at 25 ^oC for 24 h with H_2 balloon. Then, the resulting solution was filtered through a short pad of silica gel, washed by EtOAc (2×30 mL). The combined filtrates were concentrated under reduced pressure to give the crude hydrogenation product as a colorless oil, which was used for the next step without further purification. To a 250 mL flame-dried Schlenk flask charged with the crude hydrogenation product and THF (75 mL) was added 1 N HCl (a) (75 mL) at 25 ^oC. After stirring at 60 ^oC for 6 hours, the reaction mixture was cooled to room temperature and the solvent was removed under reduced pressure. EtOAc (80 mL) was added to the residue, and the solution was neutralized to pH = 7 with saturated Na_2CO_3 solution. The organic phase was separated and the aqueous phase was extracted with EtOAc (60 mL × 2). The combined organic phases were washed with saturated Na_2CO_3 solution (60 mL), brine (60 mL). The organic phases were dried over anhydrous Na_2SO_4

and concentrated in vacuo. The resulting crude product was purified by column chromatography (silica gel, 200-300 mesh) using PE/EA = 100/1 as the eluent to afford **S1ak-c** (3.0117 g, 86% yield for two steps) as a colorless oil. IR (neat): 2926, 2855, 1707, 1458 cm^{-1}; $[\alpha]^{20}_{D}$ = -6.9 (c 1.12, $CHCl_3$); ^1H NMR (400 MHz, $CDCl_3$): δ 9.74 (t, *J* = 2.0 Hz, 1H), 7.30-7.23 (m, 2H), 7.23-7.12 (m, 3H), 2.64-2.56 (t, *J* = 7.2 Hz, 2H), 2.38 (ddd, *J* = 16.0, 5.6, 1.6 Hz, 1H), 2.21 (ddd, *J* = 16.0, 8.0, 2.8 Hz, 1H), 2.10-1.95 (m, 1H), 1.67-1.54 (m, 2H), 1.41-1.18 (m, 8H), 0.95 (dd, *J* = 6.8, 1.6 Hz, 3H); ^{13}C NMR (101 MHz, $CDCl_3$): δ 203.0, 142.8, 128.4, 128.2, 125.6, 51.1, 36.9, 35.9, 31.4, 29.5, 29.2, 28.2, 26.8, 19.9. HRMS (EI) calculated for $[C_{16}H_{24}O]^+$ requires m/z 232.1827, found m/z 232.1826.

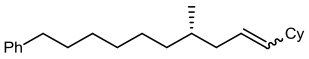

(*S*)-(10-cyclohexyl-7-methyldec-9-en-1-yl)benzene (1ak): Prepared from **S1ak-c** according to the general procedure A. *Z*/*E* ratio: 4.9/1, 83% yield, colorless oil. IR (neat): 2924, 2852, 1495, 1453, 1376 cm^{-1}; $[\alpha]^{20}_{D}$ = +4.0 (c 1.22, $CHCl_3$), 92% ee determined by HPLC, HPLC conditions: Chiralcel OJ-H*2, *n*-hexane/*i*-PrOH = 100/0, 0.5 mL/min, *n* = 220 nm, tr 19.2 (major), 20.1 (minor). ^1H NMR (400 MHz, $CDCl_3$): δ 7.33-7.23 (m, 2H), 7.22-7.10 (m, 3H), 5.40-5.30 (m, 0.34H), 5.30-5.13 (m, 1.66H), 2.66-2.55 (m, 2H), 2.30-2.16 (m, 0.83H), 2.07-1.76 (m, 2.21H), 1.74-1.55 (m, 7H), 1.48-0.98 (m, 14H), 0.90-0.82 (m, 3H). HRMS (EI) calculated for $[C_{23}H_{36}]^+$ requires m/z 312.2817, found m/z 312.2816.

General Procedures for Isomerization−Hydroboration of Alkenes: To a 25 mL flame-dried Schlenk flask cooled under nitrogen, $Co(OAc)_2$ (0.025 mmol), **L** (0.03 mmol), Et_2O (1 mL) were added. The mixture was stirred at room temperature for 5 min. Then, alkene (1.0 mmol), HBpin (180 μL, 1.2 mmol) were added in sequence and stirred at room temperature for 20 h. The resulting solution was filtered by a short pad of silica gel and washed by ether (10 mL × 2). The combined filtrate was concentrated and purified by flash column chromatography using PE/EtOAc = 20/1as the eluent to give the corresponding product.

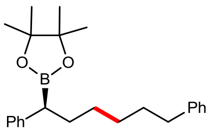

(*S*)-2-(1,6-diphenylhexyl)-4,4,5,5-tetramethyl-1,3,2-dioxaborolane (2a): Prepared according to the general procedure using $Co(OAc)_2$ (0.0045 g, 0.025 mmol), **L8** (0.0126 g, 0.031 mmol), Et_2O (1 mL), **1a** (0.2358 g, 1.0 mmol) and HBpin (180 µL, 1.2 mmol). After 20 h, the resulting solution was added 20 mL of ether and filtered through a pad of silica gel, washed by ether (10 mL × 2). The combined filtrates were concentrated and purified by flash column chromatography using PE/EtOAc = 20/1 as the eluent to afford **2a** (0.3259 g, 0.89 mmol, 90% yield) as a colorless oil. IR (neat): 2979, 2929, 2857, 1458, 1368, 1324, 1145 cm^{-1}; Optical Rotation: $[\alpha]^{20}_{D}$ = +16.1 (c 1.15, $CHCl_3$), 98% ee determined by HPLC after oxidized to the corresponding alcohol, HPLC conditions: Chiralcel AD-H, *n*-hexane/*i*-PrOH = 98/2, 1.0 mL/min, *n* = 220 nm, tr 20.2 (major), 22.1 (minor).^{1}H NMR (400 MHz, $CDCl_3$): δ 7.28-7.08 (m, 10H), 2.56 (t, *J* = 7.6 Hz, 2H), 2.28 (t, *J* = 7.6 Hz, 1H), 1.89-1.78 (m, 1H), 1.70-1.53 (m, 3H), 1.39-1.24 (m, 4H), 1.19 (s, 6H), 1.17 (s, 6H); ^{13}C NMR (101 MHz, $CDCl_3$): δ143.4, 142.8, 128.4, 128.3, 128.19, 128.16, 125.5, 125.1, 83.2, 35.9, 32.5, 31.3, 29.2, 29.1, 24.60, 24.55.HRMS (EI) calculated for$[C_{24}H_{33}BO_2]^+$ requires m/z 364.2574, found m/z 364.2574.

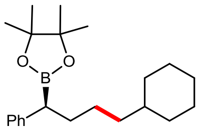

(*S*)-2-(4-cyclohexyl-1-phenylbutyl)-4,4,5,5-tetramethyl-1,3,2-dioxaborolane (2b): Prepared according to the general procedure using $Co(OAc)_2$ (0.0045 g, 0.025 mmol), **L8** (0.0126 g, 0.031 mmol), Et_2O (1 mL), **1b** (0.2120 g, 0.99 mmol) and HBpin (180 µL, 1.2 mmol). After 20 h, the resulting solution was added 20 mL of ether and filtered through a pad of silica gel, washed by ether (10 mL × 2). The combined filtrates were concentrated and purified by flash column chromatography using PE/EtOAc = 20/1 as the eluent to afford **2b** (0.2873 g, 0.84 mmol, 85% yield) as a colorless oil. IR (neat): 2978, 2925, 2853, 1454, 1368, 1324, 1145 cm^{-1}; Optical Rotation: $[\alpha]^{20}_{D}$ = +19.0 (c 0.92, $CHCl_3$), 96% ee determined by HPLC after oxidized to the corresponding alcohol, HPLC conditions: Chiralcel AS-H, *n*-hexane/*i*-PrOH = 98/2, 1.0 mL/min, *n* = 220 nm, tr 7.7 (minor), 8.9 (major).^{1}H NMR (400 MHz, $CDCl_3$): δ 7.27-7.18 (m, 4H), 7.12 (t, *J* = 7.2 Hz, 1H), 2.29 (t, *J* = 8.0 Hz, 1H), 1.87-1.75 (m, 1H), 1.70 -1.56 (m, 6H), 1.31-1.08 (m, 20H), 0.90-0.75 (m, 2H); ^{13}C NMR (101 MHz, $CDCl_3$): δ143.5, 128.3, 128.2, 125.0, 83.2, 37.47, 37.45, 33.43, 33.35, 32.9, 26.7, 26.5, 26.4, 24.63, 24.56. HRMS (EI) calculated for$[C_{22}H_{35}BO_2]^+$ requires m/z 342.2730, found m/z 342.2726.

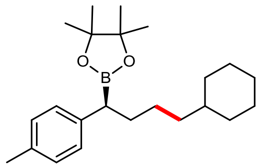

(*S*)-2-(4-cyclohexyl-1-(*p*-tolyl)butyl)-4,4,5,5-tetramethyl-1,3,2-dioxaborolane (2c): Prepared according to the general procedure using $Co(OAc)_2$ (0.0045 g, 0.025 mmol), **L8** (0.0126 g, 0.031 mmol), Et_2O (1 mL), **1c** (0.2288 g, 1.0 mmol) and HBpin (180 μL, 1.2 mmol). After 20 h, the resulting solution was added 20 mL of ether and filtered through a pad of silica gel, washed by ether (10 mL × 2). The combined filtrates were concentrated and purified by flash column chromatography using PE/EtOAc = 20/1 as the eluent to afford **2c** (0.2869 g, 0.80 mmol, 80% yield) as a colorless oil. IR (neat): 2979, 2924, 2852, 1453, 1366, 1323, 1145 cm^{-1}; Optical Rotation: $[\alpha]^{20}_{D}$ = +20.8 (c 1.18, $CHCl_3$); 99% ee determined by HPLC after oxidized to the corresponding alcohol, HPLC conditions: Chiralcel AS-H, *n*-hexane/*i*-PrOH = 98/2, 1.0 mL/min, *n* = 220 nm, tr 7.9 (minor), 10.1 (major).^{1}H NMR (400 MHz, $CDCl_3$): δ 7.11-7.02 (m, 4H), 2.29 (s, 3H), 2.25 (t, *J* = 7.6 Hz, 1H), 1.84-1.72 (m, 1H), 1.69-1.57 (m, 6H), 1.31-1.08 (m, 20H), 0.88-0.76 (m, 2H);^{13}C NMR (101 MHz, $CDCl_3$): δ140.4, 134.3, 128.9, 128.2, 83.1, 37.5, 33.44, 33.35, 33.1, 26.7, 26.5, 26.4, 24.64, 24.58, 21.0. HRMS (ESI) calculated for $[M+Na]^+[C_{23}H_{37}BO_2Na]^+$ requires m/z 379.2784, found m/z 379.2786.

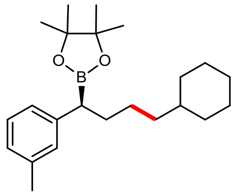

(*S*)-2-(4-cyclohexyl-1-(*m*-tolyl)butyl)-4,4,5,5-tetramethyl-1,3,2-dioxaborolane (2d): Prepared according to the general procedure using $Co(OAc)_2$ (0.0044 g, 0.025 mmol), **L8** (0.0126 g, 0.031 mmol), Et_2O (1 mL), **1d** (0.2288 g, 1.0 mmol) and HBpin (180 μL, 1.2 mmol). After 20 h, the resulting solution was added 20 mL of ether and filtered through a pad of silica gel, washed by ether (10 mL × 2). The combined filtrates were concentrated and purified by flash column chromatography using PE/EtOAc = 20/1 as the eluent to afford **2d** (0.2784 g, 0.78 mmol, 78% yield) as a colorless oil. IR (neat): 2979, 2924, 2854, 1453, 1366, 1323, 1146 cm^{-1}; Optical Rotation: $[\alpha]^{20}_{D}$ = +18.2 (c 0.90, $CHCl_3$); 98% ee determined by HPLC after oxidized to the corresponding alcohol, HPLC conditions: Chiralcel AS-H, *n*-hexane/*i*-PrOH = 98/2, 1.0 mL/min, *n* = 220 nm, tr 7.2 (minor), 7.8 (major).^{1}H NMR (400 MHz, $CDCl_3$): δ 7.13 (t, *J* = 7.6 Hz, 1H), 7.03-6.98 (m, 2H), 6.93 (d, *J* = 7.2 Hz, 1H), 2.30 (s, 3H), 2.25 (t, *J* = 8.0 Hz, 1H), 1.85-1.74 (m, 1H), 1.71-1.55 (m, 6H), 1.32-1.08 (m, 20H), 0.90-0.74 (m, 2H); ^{13}C NMR (101 MHz, $CDCl_3$): δ143.4, 137.6, 129.2, 128.0, 125.8, 125.3, 83.2, 37.5, 37.4, 33.4, 33.3, 33.0, 26.7, 26.6, 26.4, 24.61,

24.56, 21.4.HRMS (ESI) calculated for $[M+H]^+[C_{23}H_{38}BO_2]^+$ requires m/z 357.2965, found m/z 357.2959.

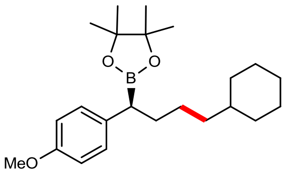

(*S*)-2-(4-cyclohexyl-1-(4-methoxyphenyl)butyl)-4,4,5,5-tetramethyl-1,3,2-dioxaborolane (2e): Prepared according to the general procedure using $Co(OAc)_2$ (0.0044 g, 0.025 mmol), **L8** (0.0126 g, 0.031 mmol), Et_2O (1 mL), **1e**(0.2448 g, 1.0 mmol) and HBpin (180 μL, 1.2 mmol). After 20 h, the resulting solution was added 20 mL of ether and filtered through a pad of silica gel, washed by ether (10 mL × 2). The combined filtrates were concentrated and purified by flash column chromatography using PE/EtOAc = 20/1 and PE/EtOAc = 10/1 as the eluent to afford **2e** (0.2608 g, 0.70 mmol, 70% yield) as a colorless oil. IR (neat): 2924, 2853, 1511, 1455, 1367, 1323, 1247, 1145 cm^{-1}; Optical Rotation: $[\alpha]^{20}_{D}$ = +26.8 (c 0.76, $CHCl_3$); 98% ee determined by HPLC after oxidized to the corresponding alcohol, HPLC conditions: Chiralcel OD-H, *n*-hexane/*i*-PrOH = 98/2, 1.0 mL/min, *n* = 220 nm, tr 15.8 (minor), 16.8 (major).^{1}H NMR (400 MHz, $CDCl_3$): δ 7.14-7.09 (m, 2H), 6.82-6.76 (m, 2H), 3.77 (s, 3H), 2.23 (t, *J* = 8.0 Hz, 1H), 1.82-1.71 (m, 1H), 1.67-1.51 (m, 6H), 1.30-1.10 (m, 20H), 0.88-0.75 (m, 2H); ^{13}C NMR (101 MHz, $CDCl_3$): δ157.2, 135.5, 129.1, 113.6, 83.1, 55.1, 37.5, 37.4, 33.4, 33.34, 33.13, 31.2, 26.7, 26.43, 26.40, 24.63, 24.56. HRMS (ESI) calculated for $[M+H]^+[C_{23}H_{38}BO_3]^+$ requires m/z 373.2914, found m/z 373.2912.

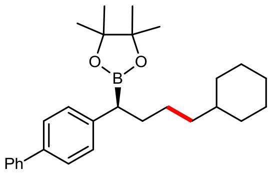

(*S*)-2-(1-([1,1'-biphenyl]-4-yl)-4-cyclohexylbutyl)-4,4,5,5-tetramethyl-1,3,2-dioxaborolane (2f): Prepared according to the general procedure using $Co(OAc)_2$ (0.0047 g, 0.027 mmol), **L8** (0.0126 g, 0.031 mmol), Et_2O (1 mL), **1f** (0.2925 g, 1.0 mmol) and HBpin (180 μL, 1.2 mmol). After 20 h, the resulting solution was added 20 mL of ether and filtered through a pad of silica gel, washed by ether (10 mL × 2). The combined filtrates were concentrated and purified by flash column chromatography using PE/EtOAc = 100/1 as the eluent to afford **2f** (0.3423 g, 0.82 mmol, 81% yield) as a white solid. IR (neat): 2922, 2851, 1486, 1448, 1361, 1322, 1143 cm^{-1};Optical Rotation: $[\alpha]^{20}_{D}$ = +23.7 (c 1.15, $CHCl_3$), 99% ee determined by HPLC after oxidized to the corresponding alcohol, HPLC conditions: Chiralcel AS-H, *n*-hexane/*i*-PrOH = 98/2, 1.0

mL/min, n = 220 nm, tr 11.8 (minor), 13.2 (major).^{1}H NMR (400 MHz, $CDCl_3$): δ7.63-7.54 (m, 2H), 7.53-7.45 (m, 2H), 7.41 (t, J = 7.6 Hz, 2H), 7.34-7.23 (m, 3H), 2.35 (t, J = 7.6 Hz, 1H), 1.90-1.79 (m, 1H), 1.72-1.57 (m, 6H), 1.34-1.10 (m, 20H), 0.91-0.76 (m, 2H); ^{13}C NMR (101 MHz, $CDCl_3$): δ142.7, 141.2, 137.8, 128.7, 128.6, 126.90, 126.86, 126.8, 83.2 37.5, 33.4, 33.3, 32.9, 26.7, 26.5, 26.4, 24.64, 24.57. HRMS (ESI) calculated for $[M+H]^+[C_{28}H_{40}BO_2]^+$ requires m/z 419.3121, found m/z 419.3129.

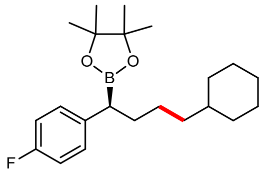

(*S*)-2-(4-cyclohexyl-1-(4-fluorophenyl)butyl)-4,4,5,5-tetramethyl-1,3,2-dioxaborolane (2g): Prepared according to the general procedure using $Co(OAc)_2$ (0.0043 g, 0.024 mmol), **L8** (0.0127 g, 0.031 mmol), Et_2O (1 mL), **1g** (0.2332 g, 1.0 mmol) and HBpin (180 μL, 1.2 mmol). After 20 h, the resulting solution was added 20 mL of ether and filtered through a pad of silica gel, washed by ether (10 mL × 2). The combined filtrates were concentrated and purified by flash column chromatography using PE/EtOAc = 20/1 as the eluent to afford **2g** (0.3024 g, 0.84 mmol, 84% yield) as a colorless oil. IR (neat): 2978, 2924, 2852, 1509, 1451, 1367, 1326, 1225, 1146 cm^{-1}; Optical Rotation: $[\alpha]^{20}_{D}$ = +18.4 (c 1.20, $CHCl_3$), 98% ee determined by HPLC after oxidized to the corresponding alcohol, HPLC conditions: Chiralcel AS-H, *n*-hexane/*i*-PrOH = 98/2, 1.0 mL/min, n = 220 nm, tr 8.1 (minor), 8.8 (major).^{1}H NMR (400 MHz, $CDCl_3$): δ 7.18-7.11 (m, 2H), 6.96-6.89 (m, 2H), 2.27 (t, J = 7.6 Hz, 1H), 1.83-1.72 (m, 1H), 1.70-1.53 (m, 6H), 1.29-1.08 (m, 20H), 0.88-0.75 (m, 2H); ^{13}C NMR (101 MHz, $CDCl_3$): δ160.9 (d, J = 243.0 Hz, 1C), 139.0 (d, J = 2.9 Hz, 1C), 129.5 (d, J = 7.4 Hz, 1C), 114.9 (d, J = 20.6 Hz, 1C), 83.3, 37.5, 37.4, 33.4, 33.3, 33.0, 26.7, 26.4, 24.62, 24.55; ^{19}F NMR (376 MHz, $CDCl_3$): δ -119.0.HRMS (ESI) calculated for $[M+H]^+[C_{22}H_{35}BFO_2]^+$ requires m/z 361.2714, found m/z 361.2697.

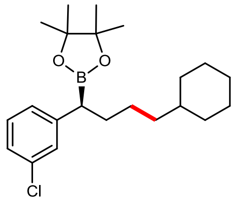

(*S*)-2-(1-(3-chlorophenyl)-4-cyclohexylbutyl)-4,4,5,5-tetramethyl-1,3,2-dioxaborolane (2h): Prepared according to the general procedure using $Co(OAc)_2$ (0.0045 g, 0.025 mmol), **L8** (0.0124 g, 0.030 mmol), Et_2O (1 mL), **1h** (0.2490 g, 1.0 mmol) and HBpin (180 μL, 1.2 mmol). After 20 h, the resulting solution was added 20 mL of ether and filtered through a pad of silica gel, washed by ether (10 mL × 2). The combined filtrates were concentrated and purified by flash column

chromatography using PE/EtOAc = 20/1 as the eluent to afford **2h** (0.2712 g, 0.72 mmol, 72% yield) as a colorless oil. IR (neat): 2976, 2925, 2853, 1464, 1367, 1328, 1145 cm^{-1}; Optical Rotation: $[\alpha]^{20}_{D}$ = +12.0 (c 0.90, $CHCl_3$), 97% ee determined by HPLC after oxidized to the corresponding alcohol, HPLC conditions: Chiralcel AD-H, *n*-hexane/*i*-PrOH = 98/2, 1.0 mL/min, *n* = 220 nm, tr 13.3 (major), 14.3 (minor).^{1}H NMR (400 MHz, $CDCl_3$): δ7.22-7.13 (m, 2H), 7.12-7.05 (m, 2H), 2.27 (t, *J* = 8.0 Hz, 1H), 1.85-1.73 (m, 1H), 1.71-1.54 (m, 6H), 1.30-1.08 (m, 20H), 0.92-0.75 (m, 2H);^{13}C NMR (101 MHz, $CDCl_3$): δ145.7, 133.9, 129.4, 128.4, 126.5, 125.2, 83.4, 37.41, 37.37, 33.4, 33.3, 32.6, 26.7, 26.4, 24.60, 24.55. HRMS (ESI) calculated for $[M+Na]^+[C_{22}H_{34}BClO_2Na]^+$ requires m/z 399.2238, found m/z 399.2231.

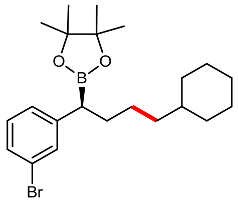

(*S*)-2-(1-(3-bromophenyl)-4-cyclohexylbutyl)-4,4,5,5-tetramethyl-1,3,2-dioxaborolane (2i): Prepared according to the general procedure using $Co(OAc)_2$ (0.0047 g, 0.027 mmol), **L8** (0.0125 g, 0.030 mmol), Et_2O (1 mL), **1i** (0.2963 g, 1.0 mmol) and HBpin (180 μL, 1.2 mmol). After 48 h, the resulting solution was added 20 mL of ether and filtered through a pad of silica gel, washed by ether (10 mL × 2). The combined filtrates were concentrated and purified by flash column chromatography using PE/EtOAc = 100/1 as the eluent to afford **2i** (0.3378 g, 0.80 mmol, 79 % yield) a colorless oil. IR (neat): 2923, 2852, 1471, 1360, 1325, 1143 cm^{-1}; Optical Rotation: $[\alpha]^{20}_{D}$ = +13.9 (c 1.02, $CHCl_3$),97 % ee determined by HPLC, HPLC conditions: Chiralcel AS-H, *n*-hexane/*i*-PrOH = 99/1, 0.8 mL/min, *n* = 220 nm, tr 18.5 (minor), 19.5 (major). ^{1}H NMR (400 MHz, $CDCl_3$): δ 7.41-7.31 (m, 1H), 7.27-7.22 (m, 1H), 7.18-7.06 (m, 2H), 2.26 (t, *J* = 7.6 Hz, 1H), 1.83-1.73 (m, 1H), 1.72-1.52 (m, 6H), 1.33-1.10 (m, 20H), 0.92-0.72 (m, 2H); ^{13}C NMR (101 MHz, $CDCl_3$): δ 146.0, 131.3, 129.7, 128.2, 127.0, 122.3, 83.4, 37.42, 37.36, 33.4, 33.3, 32.7, 26.7, 26.4, 24.60, 24.56; HRMS (EI) calculated for $[C_{22}H_{34}BBrO_2]^+$ requires m/z 420.1835, found m/z 420.1838.

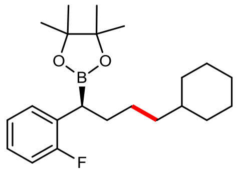

(*S*)-2-(4-cyclohexyl-1-(2-fluorophenyl)butyl)-4,4,5,5-tetramethyl-1,3,2-dioxaborolane (2j): Prepared according to the general procedure using $Co(OAc)_2$ (0.0176 g, 0.099 mmol), **L8** (0.0494 g, 0.120 mmol), Et_2O (1

mL), **1j** (0.2360 g, 1.0 mmol) and HBpin (180 μL, 1.2 mmol). After 48 h, the resulting solution was added 20 mL of ether and filtered through a pad of silica gel, washed by ether (10 mL × 2). The combined filtrates were concentrated and purified by flash column chromatography using PE/EtOAc = 100/1 as the eluent to afford **2j** (0.2322 g, 0.80 mmol, 63 % yield) a colorless oil. IR (neat): 2923, 2852,1453, 1370, 1324, 1144 cm^{-1}; OpticalRotation: $[\alpha]^{20}_{D}$ = +23.5 (c 1.05, $CHCl_3$), 99 % ee determined by HPLC, HPLC conditions: Chiralcel AS-H, *n*-hexane/*i*-PrOH = 98/2, 1.0 mL/min, *n* = 220 nm, tr 8.4 (minor), 9.1 (major). ^{1}H NMR (400 MHz, $CDCl_3$):7.23 (dt, *J* = 7.6, 1.6 Hz, 1H), 7.15-7.07 (m, 1H), 7.03 (dt, *J* = 7.2, 1.2 Hz, 1H), 7.01-6.93 (m, 1H), 2.52 (t, *J* = 7.6 Hz, 1H), 1.85-1.74 (m, 1H), 1.70-1.56 (m, 6H), 1.32-1.04 (m, 20H), 0.90-0.71 (m, 2H); ^{13}C NMR (101 MHz, $CDCl_3$): δ161.0 (d, *J* = 244.5 Hz, 1C), 130.6 (d, *J* = 15.5 Hz, 1C), 130.3 (d, *J* = 5.2 Hz, 1C), 126.5 (d, *J* = 8.1 Hz, 1C), 123.8 (d, *J* = 3.7 Hz, 1C), 115.0 (d, *J* = 22.8 Hz, 1C), 83.3, 37.5, 37.4, 33.4, 33.3, 31.5, 26.7, 26.4, 26.3, 24.7, 24.6; ^{19}F NMR: (376 MHz, $CDCl_3$) : δ -117.2; HRMS (EI) calculated for $[C_{22}H_{34}BFO_2]^+$ requires m/z 360.2636, found m/z 360.2636.

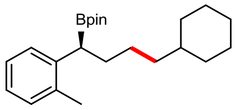

(*S*)-2-(4-cyclohexyl-1-(*o*-tolyl)butyl)-4,4,5,5-tetramethyl-1,3,2-dioxaborolane (2k): Prepared according to the general procedure using $Co(OAc)_2$ (0.0180 g, 0.10 mmol), **L8** (0.0496 g, 0.12 mmol), Et_2O (1 mL), **1k** (0.2298 g, 1.0 mmol) and HBpin (180 μL, 1.2 mmol). After 48 h, the resulting solution was added 20 mL of ether and filtered through a pad of silica gel, washed by ether (10 mL × 2). The combined filtrates were concentrated and purified by flash column chromatography using PE/EtOAc = 100/1 as the eluent to afford **2k** (0.1097 g, 0.31 mmol, 31% yield) as a colorless oil. >99% ee determined by HPLC after oxidized to the corresponding alcohol, HPLC conditions: Chiralcel AS-H, *n*-hexane/*i*-PrOH = 98/2, 1.0 mL/min, *n* = 220 nm, tr 7.0 (minor), 7.9 (major). IR (neat): 2923, 2852,1454, 1363, 1321, 1144 cm^{-1}; Optical Rotation: $[\alpha]^{20}_{D}$ = +9.7 (c 1.01, $CHCl_3$); ^{1}H NMR (400 MHz, $CDCl_3$): δ7.24-7.18 (m, 1H), 7.15-7.07 (m, 2H), 7.05-6.98 (m, 1H), 2.49 (t, *J* = 7.6 Hz, 1H), 2.32 (s, 3H), 1.88-1.77 (m, 1H), 1.71-1.56 (m, 6H), 1.36-1.09 (m, 20H), 0.91-0.76 (m, 2H); ^{13}C NMR (101 MHz, $CDCl_3$): δ142.0, 135.9, 130.0, 127.7, 125.8, 124.8, 83.1, 37.6, 37.5, 33.44, 33.38, 32.5, 26.8, 26.7, 26.4, 24.7, 24.6, 20.1; HRMS (ESI) calculated for$[M+Na]^+$ $[C_{23}H_{37}BO_2Na]^+$ requires m/z 379.2784, found m/z 379.2800.

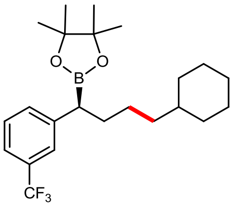

(*S*)-2-(4-cyclohexyl-1-(3-(trifluoromethyl)phenyl)butyl)-4,4,5,5-tetramethyl-1,3,2-dioxaborolane (2l): Prepared according to the general procedure using $Co(OAc)_2$ (0.0043 g, 0.024 mmol), **L8** (0.0126 g, 0.031 mmol), Et_2O (1 mL), **1l** (0.2839 g, 1.0 mmol) and HBpin (180 μL, 1.2 mmol). After 20 h, the resulting solution was added 20 mL of ether and filtered through a pad of silica gel, washed by ether (10 mL × 2). The combined filtrates were concentrated and purified by flash column chromatography using PE/EtOAc = 20/1 as the eluent to afford **2l** (0.2243 g, 0.55 mmol, 54% yield) as a colorless oil. IR (neat): 2925, 2854, 1451, 1370, 1329, 1132 cm^{-1}; Optical Rotation: $[\alpha]^{20}_{D}$ = +8.5 (c 0.95, $CHCl_3$), 97% ee determined by HPLC after oxidized to the corresponding alcohol, HPLC conditions: Chiralcel AD-H, *n*-hexane/*i*-PrOH = 98/2, 1.0 mL/min, *n* = 220 nm, tr 8.2 (major), 8.9 (minor). ^{1}H NMR (400 MHz, $CDCl_3$): *δ* 7.47 (s, 1H), 7.42-7.32 (m, 3H), 2.36 (t, *J* = 8.0 Hz, 1H), 1.88-1.76 (m, 1H), 1.70-1.57 (m, 6H), 1.32-1.08 (m, 20H), 0.90-0.75 (m, 2H); ^{13}C NMR (101 MHz, $CDCl_3$): *δ*144.5, 131.7, 130.4 (q, *J* = 31.6 Hz, 1C), 128.5, 125.0 (q, *J* = 3.7 Hz, 1C),124.4 (q, *J* = 273.2 Hz, 1C),122.0 (q, *J* = 2.9 Hz, 1C), 83.5, 37.4, 37.3, 33.4, 33.3, 32.8, 26.7, 26.4, 24.5; ^{19}F NMR (376 MHz, $CDCl_3$): *δ* -62.5.HRMS (ESI) calculated for $[M+Na]^+$ $[C_{23}H_{34}BF_3O_2Na]^+$ requires m/z 433.2502, found m/z 433.2498.

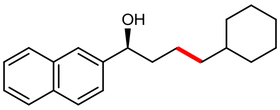

(*S*)-4-cyclohexyl-1-(naphthalen-2-yl)butan-1-ol (3m): Prepared according to the general procedure using $Co(OAc)_2$ (0.0045 g, 0.025 mmol), **L8** (0.0126 g, 0.031 mmol), Et_2O (1 mL), **1m** (0.2645 g, 1.0 mmol) and HBpin (180 μL, 1.2 mmol). After 20 h, the resulting solution was added 20 mL of ether and filtered through a pad of silica gel, washed by ether (10 mL × 2). The combined filtrates were concentrated and dissolved in ether (10 mL). The solution was cooled to 0 ℃ with an ice-water bath and NaOH (2 mL of a 2 M aqueous solution) was added followed by H_2O_2 (2 mL of a 30% aqueous solution). After stirring at room temperature for 2 hour, the reaction mixture was diluted with ether and water. The organic layer was separated and the aqueous layer extracted with EtOAc (3× 20 mL). The combined organic phases were dried over anhydrous Na_2SO_4 and concentrated in vacuo and purified by flash column chromatography using PE/EtOAc = 10/1 as the eluent to afford **3m** (0.2357 g, 0.83 mmol, 83% yield) as a colorless oil. IR (neat): 3264, 2922, 2852, 1453, 1371, 1313, 1048 cm^{-1}; Optical Rotation: $[\alpha]^{20}_{D}$ = -20.9 (c 0.90, $CHCl_3$), 98% ee determined by HPLC,

HPLC conditions: Chiralcel AS-H, *n*-hexane/*i*-PrOH = 98/2, 1.0 mL/min, *n* = 220 nm, tr 10.5 (minor), 13.6 (major).^{1}H NMR (400 MHz, $CDCl_3$): δ 7.86-7.80 (m, 3H), 7.77 (s, 1H), 7.52-7.43 (m, 3H), 4.87-4.79 (m, 1H), 1.95-1.57 (m, 8H), 1.51-1.38 (m, 1H), 1.36-1.05 (m, 7H), 0.91-0.77 (m, 2H); ^{13}C NMR (101 MHz, $CDCl_3$): δ142.3, 133.3, 132.9, 128.2, 127.9, 127.7, 126.1, 125.7, 124.6, 124.1, 74.8, 39.3, 37.5, 37.3, 33.4, 33.3, 26.7, 26.4, 23.1.HRMS (ESI) calculated for $[M+Na]^+[C_{20}H_{26}ONa]^+$ requires m/z 305.1881, found m/z 305.1893.

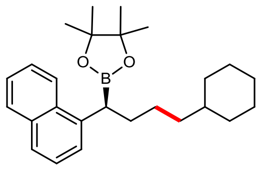

(*S*)-2-(4-cyclohexyl-1-(naphthalen-1-yl)butyl)-4,4,5,5-tetramethyl-1,3,2-dioxaborolane (2n): Prepared according to the general procedure using $Co(OAc)_2$ (0.0089 g, 0.050 mmol), **L8** (0.0251 g, 0.061 mmol), Et_2O (1 mL), **1n** (0.2661 g, 1.0 mmol) and HBpin (180 μL, 1.2 mmol). After 48 h, the resulting solution was added 20 ml of ether and filtered through a pad of silica gel, washed by ether (10 mL× 2). The combined filtrates were concentrated and purified by flash column chromatography using PE/EtOAc = 100/1 as the eluent to afford **2 n** (0.2761 g, 0.70 mmol, 70% yield) as a white solid. IR (neat):2922, 2851, 1322, 1143 cm^{-1}; Optical Rotation: $[\alpha]^{20}_D$ = +27.4 (c 0.90, $CHCl_3$), >99% ee determined by HPLC after oxidized to the corresponding alcohol, HPLC conditions: Chiralcel AS-H, *n*-hexane/*i*-PrOH = 98/2, 1.0 mL/min, *n* = 220 nm, tr 11.0 (minor), 13.4 (major). ^{1}H NMR (400 MHz, $CDCl_3$): δ8.16 (d, *J* = 8.4 Hz, 1H), 7.84-7.74 (m, 1H), 7.68-7.56 (m, 1H), 7.57-7.32 (m, 4H), 3.02 (dd, *J* = 8.0, 7.6 Hz, 1H), 2.10-1.98 (m, 1H), 1.82-1.73 (m, 1H), 1.69-1.57 (m, 5H), 1.42-1.30 (m, 2H), 1.24-1.04 (m, 18H), 0.89-0.75 (m, 2H);^{13}C NMR (101 MHz, $CDCl_3$): δ140.1, 134.0, 132.2, 128.7, 125.71, 125.67, 125.3, 125.13, 125.06, 124.2, 83.3,37.6, 37.5, 33.40, 33.35, 32.4, 26.8, 26.7, 26.4, 24.7, 24.5. HRMS (ESI) calculated for $[M+H]^+[C_{26}H_{38}BO_2]^+$ requires m/z 393.2965, found m/z 393.2967.

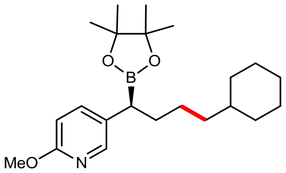

(*S*)-5-(4-cyclohexyl-1-(4,4,5,5-tetramethyl-1,3,2-dioxaborolan-2-yl) butyl)-2-methoxypyridine (2o): Prepared according to the general procedure using $Co(OAc)_2$ (0.0043 g, 0.024 mmol), **L8** (0.0127 g, 0.031 mmol), Et_2O (1 mL), **1o** (0.2458 g, 1.0 mmol) and HBpin (180 μL, 1.2 mmol). After 20 h, the resulting solution was added 20 mL of ether and filtered through a pad of silica gel, washed by ether (10 mL × 2). The combined filtrates were concentrated and

purified by flash column chromatography using PE/EtOAc = 10/1 as the eluent to afford **2o** (0.2405 g, 0.64 mmol, 64% yield) as a colorless oil. IR (neat): 2979, 2923, 2851, 1605, 1491, 1452, 1367, 1323, 1283, 1143 cm^{-1}; Optical Rotation: $[\alpha]^{20}_{D}$ = +17.1 (c 1.13, $CHCl_3$), 97% ee determined by HPLC after oxidized to the corresponding alcohol, HPLC conditions: Chiralcel AD-H, *n*-hexane/*i*-PrOH = 98/2, 1.0 mL/min, *n* = 220 nm, tr 21.8 (major), 25.8 (minor). ^{1}H NMR (400 MHz, $CDCl_3$): δ 7.97 (s, 1H), 7.45 (d, *J* = 8.4 Hz, 1H), 6.66 (d, *J* = 7.6 Hz, 1H), 3.90 (s, 3H), 2.22 (t, *J* = 7.2 Hz, 1H), 1.81-1.50 (m, 7H), 1.30-1.08 (m, 20H), 0.90-0.74 (m, 2H); ^{13}C NMR (101 MHz, $CDCl_3$): δ162.2, 146.0, 138.9, 131.4, 110.3, 83.4, 53.2, 37.5, 37.3, 33.4, 33.3, 33.0, 29.7, 28.3, 26.7, 26.4, 26.2, 24.65, 24.60. HRMS (ESI) calculated for $[M+Na]^+[C_{22}H_{36}BNO_3Na]^+$ requires m/z 396.2686, found m/z 396.2687.

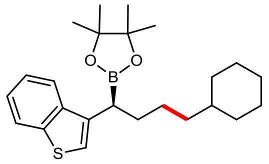

(*S*)-2-(1-(benzo[b]thiophen-3-yl)-4-cyclohexylbutyl)-4,4,5,5-tetramethyl-1,3,2-dioxaborolane (2p): Prepared according to the general procedure using $Co(OAc)_2$ (0.0086 g, 0.049mmol), **L8** (0.0246 g, 0.060mmol), Et_2O (1 mL), **1p** (0.2911 g, 1.0 mmol) and HBpin (180 μL, 1.2 mmol). After 24 h, the resulting solution was added 20 mL of ether and filtered through a pad of silica gel, washed by ether (10 mL × 2). The combined filtrates were concentrated and purified by flash column chromatography using PE/EtOAc = 100/1 as the eluent to afford **2p** (0.3361 g, 0.87mmol, 87% yield) as a white solid.IR (neat): 2921, 2850, 1368, 1323, 1142 cm^{-1}; Optical Rotation: $[\alpha]^{20}_{D}$ = +12.4 (c 1.02, $CHCl_3$), 99% ee determined by HPLC after oxidized to the corresponding alcohol, HPLC conditions: Chiralcel AS-H, *n*-hexane/*i*-PrOH = 99/1, 1.0 mL/min, *n* = 220 nm, tr15.02 (major), 16.17 (minor). ^{1}H NMR (400 MHz, $CDCl_3$): δ 7.92-7.74 (m, 2H), 7.38-7.28 (m, 2H), 7.14 (s, 1H), 2.73 (t, *J* = 7.6 Hz, 1H), 1.98-1.84 (m, 1H), 1.82-1.73 (m, 1H), 1.72-1.57 (m, 5H), 1.41-1.31 (m, 2H), 1.26-1.10 (m, 18H), 0.90-0.78 (m, 2H); ^{13}C NMR (101 MHz, $CDCl_3$): δ140.3, 139.2, 137.7, 123.9, 123.4, 122.7, 122.1, 120.3, 83.4, 37.49, 37.46, 33.4, 31.7, 26.71, 26.67, 26.4, 24.7, 24.6. HRMS (ESI) calculated for $[M+H]^+[C_{24}H_{36}BO_2S]^+$ requires m/z 399.2529, found m/z 399.2532.

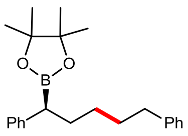

(*S*)-2-(1,5-diphenylpentyl)-4,4,5,5-tetramethyl-1,3,2-dioxaborolane (2q): Prepared according to the general procedure using $Co(OAc)_2$ (0.0045 g, 0.025

mmol), **L8** (0.0126 g, 0.031 mmol), Et_2O (1 mL), **1q** (0.2220 g, 1.0 mmol) and HBpin (180 μL, 1.2 mmol). After 20 h, the resulting solution was added 20 mL of ether and filtered through a pad of silica gel, washed by ether (10 mL×2). The combined filtrates were concentrated and purified by flash column chromatography using PE/EtOAc = 20/1 as the eluent to afford **2q** (0.3200 g, 0.91 mmol, 91% yield) as a colorless oil. IR (neat): 2978, 2929, 2858, 1458, 1368, 1325, 1145 cm^{-1}; Optical Rotation: $[\alpha]^{20}_{D}$ = +17.4 (c 1.29, $CHCl_3$) (lit.[23]: $[\alpha]^{20}_{D}$ = -11.8 (c 2.43, $CHCl_3$), 98% ee), 99% ee determined by HPLC after oxidized to the corresponding alcohol, HPLC conditions: Chiralcel AS-H, *n*-hexane/*i*-PrOH = 98/2, 1.0 mL/min, *n* = 220 nm, tr 11.9 (minor), 13.5 (major).^{1}H NMR (400 MHz, $CDCl_3$): δ 7.28-7.18 (m, 6H), 7.17-7.10 (m, 4H), 2.61-2.51 (m, 2H), 2.29 (t, *J* = 7.6 Hz, 1H), 1.93-1.80 (m, 1H), 1.75-1.59 (m, 3H),1.37-1.28 (m, 2H), 1.18 (s, 6H), 1.16 (s, 6H); ^{13}C NMR (101 MHz, $CDCl_3$): δ143.3, 142.8, 128.4, 128.3, 128.22, 128.16, 125.5, 125.1, 83.2, 35.8, 32.5, 31.4, 28.9, 24.6, 24.5.HRMS (EI) calculated for$[C_{23}H_{31}BO_2]^+$ requires m/z 350.2417, found m/z350.2414.

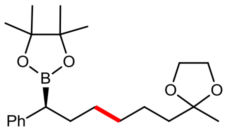

(*S*)-4,4,5,5-tetramethyl-2-(6-(2-methyl-1,3-dioxolan-2-yl)-1-phenylhexyl)-1,3,2-dioxaborolane (2r): Prepared according to the general procedure using $Co(OAc)_2$ (0.0045 g, 0.025 mmol), **L8** (0.0126 g, 0.031 mmol), Et_2O (1 mL), **1r** (0.2468 g, 1.0 mmol) and HBpin (180 μL, 1.2 mmol). After 20 h, the resulting solution was added 20 mL of ether and filtered through a pad of silica gel, washed by ether (10 mL × 2). The combined filtrates were concentrated and purified by flash column chromatography using PE/EtOAc = 20/1 as the eluent to afford **2r** (0.2664 g, 0.71 mmol, 71% yield) as a colorless oil. IR (neat): 2981, 2933, 1457, 1369, 1324, 1144, 1070 cm^{-1}; Optical Rotation: $[\alpha]^{20}_{D}$ = +15.9 (c 1.39, $CHCl_3$), 99% ee determined by HPLC, HPLC conditions: Chiralcel OJ-H, *n*-hexane/*i*-PrOH = 99/1, 0.5 mL/min, *n* = 220 nm, tr 9.9 (minor), 11.0 (major).^{1}H NMR (400 MHz, $CDCl_3$): δ 7.27-7.16 (m, 4H), 7.15-7.09 (m, 1H), 3.96-3.85 (m, 4H), 2.28 (t, *J* = 8.4 Hz, 1H), 1.89-1.78 (m, 1H), 1.70-1.56 (m, 3H), 1.39-1.22 (m, 9H), 1.20 (s, 6H), 1.18 (s, 6H); ^{13}C NMR (101 MHz, $CDCl_3$): δ143.4, 128.3, 128.2, 125.0, 110.2, 83.2, 64.5, 39.2, 32.4, 29.9, 29.2, 24.6, 24.5, 24.0, 23.7. HRMS (ESI) calculated for $[M+H]^+$ calculated for$[C_{22}H_{36}BO_4]^+$ requires m/z 375.2707, found m/z375.2706.

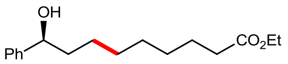

(*S*)-ethyl 9-hydroxy-9-phenylnonanoate (3s): Prepared according to the general procedure using $Co(OAc)_2$ (0.0088 g, 0.050 mmol), **L8** (0.0246 g, 0.060 mmol), Et_2O (1 mL), **1s** (0.2609 g, 1.0 mmol) and HBpin (180 μL, 1.2 mmol). After 20 h, the resulting solution was added 20 mL of ether and filtered through a pad of silica gel, washed by ether (10 mL x 3). The combined filtrates were concentrated and dissolved in ether (10 mL). The solution was cooled to 0 ℃ with an ice-water bath and NaOH (2 mL of a 2 M aqueous solution) was added followed by H_2O_2 (2 mL of a 30% aqueous solution). After stirring at room temperature for 2 hour, the reaction mixture was diluted with ether and water. The organic layer was separated and the aqueous layer extracted with EtOAc (3× 20 mL). The combined organic phases were dried over anhydrous Na_2SO_4 and concentrated in vacuo and purified by flash column chromatography using PE/EtOAc = 10/1 and PE/EtOAc = 5/1as the eluent to afford **3s** (0.1821 g, 0.65 mmol, 65% yield) as a colorless oil. IR (neat): 3446, 2930, 2858, 1731, 1456, 1376, 1188, 1034 cm^{-1}; Optical Rotation: $[\alpha]^{20}_{D}$ = -21.6 (c 1.22, $CHCl_3$), 99% ee determined by HPLC, HPLC conditions: Chiralcel AS-H, *n*-hexane/*i*-PrOH = 98/2, 1.0 mL/min, *n* = 220 nm, tr 23.6 (minor), 25.7 (major). ^1H NMR (400 MHz, $CDCl_3$): δ 7.38-7.31 (m, 4H), 7.30-7.23 (m, 1H), 4.70-4.60 (m, 1H), 4.11 (q, *J* = 6.8 Hz, 2H), 2.27 (t, *J* = 7.6 Hz, 2H), 1.89 (s, 1H), 1.84-1.67 (m, 2H), 1.62-1.54 (m, 2H), 1.46-1.36 (m, 1H), 1.33-1.21 (m, 10H); ^{13}C NMR (101 MHz, $CDCl_3$): δ173.9, 144.9, 128.4, 127.5, 125.9, 74.6, 60.1, 39.0, 34.3, 29.3, 29.1, 29.0, 25.7, 24.9, 14.2. HRMS (ESI) calculated for $[M+Na]^+[C_{17}H_{26}O_3Na]^+$ requires m/z 301.1780, found m/z 301.1781.

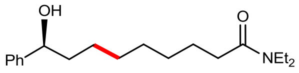

(*S*)-*N,N*-diethyl-9-hydroxy-9-phenylnonanamide (3t): Prepared according to the general procedure using $Co(OAc)_2$ (0.0090 g, 0.051 mmol), **L8** (0.0247 g, 0.060 mmol), Et_2O (1 mL), **1t** (0.2852 g, 0.99 mmol) and HBpin (300 μL, 2.0 mmol). After 36 h, the resulting solution was added 20 mL of ether and filtered through a pad of silica gel, washed by ether (10 mL x 3). The combined filtrates were concentrated and dissolved in THF/H_2O (10/10 mL). The solution was cooled to 0 ℃ with an ice-water bath and $NaBO_3$ $4H_2O$ (0.6188 g, 4.0 mmol) was added to this solution. After stirring at room temperature for 2 hour, the reaction mixture was diluted with ether and water. The organic layer was separated and the aqueous layer extracted with EtOAc (4× 20 mL). The combined organic phases were dried over anhydrous Na_2SO_4 and concentrated in vacuo and purified by flash column chromatography using

PE/EtOAc (5/1 to 2/1) as the eluent to afford **3t** (0.1950 g, 0.64 mmol, 64% yield) as a colorless oil. IR (neat): 3403, 2930, 2854, 1623, 1454 cm^{-1}; Optical Rotation: $[\alpha]^{20}_{D}$ = -21.0 (c 0.95, $CHCl_3$), 99% ee determined by HPLC, HPLC conditions: Chiralcel OD-H, *n*-hexane/*i*-PrOH = 90/10, 1.0 mL/min, *n* = 220 nm, tr 18.3 (major), 20.2 (minor). ^{1}H NMR (400 MHz, $CDCl_3$): δ 7.38-7.24 (m, 5H), 4.66 (dd, *J* = 6.8, 6.0 Hz, 1H), 3.35 (q, *J* = 7.2 Hz, 2H), 3.28 (q, *J* = 7.2 Hz, 2H), 2.26 (t, *J* = 7.6 Hz, 2H), 2.11 (s, 1H), 1.89-1.54 (m, 4H), 1.46-1.22 (m, 8H), 1.16 (t, *J* = 7.2 Hz, 3H), 1.10 (t, *J* = 7.2 Hz, 3H); ^{13}C NMR (101 MHz, $CDCl_3$): δ 172.3, 145.0, 128.4, 127.4, 125.9, 74.5, 41.9, 40.0, 39.1, 33.1, 29.4, 29.3, 25.7, 25.4, 14.4, 13.1.HRMS (ESI) calculated for $[M+Na]^+[C_{19}H_{31}NO_2Na]^+$ requires m/z 328.2252, found m/z 328.2256.

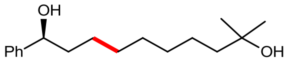

(*S*)-9-methyl-1-phenyldecane-1,9-diol (3u): Prepared according to the general procedure using $Co(OAc)_2$ (0.0045 g, 0.025 mmol), **L8** (0.0124 g, 0.03 mmol), Et_2O (1 mL), **1u** (0.1235 g, 0.5 mmol) and HBpin (180μL, 1.2 mmol). After 20 h, the resulting solution was added 20 mL of ether and filtered through a pad of silica gel, washed by ether (10 mL x 3). The combined filtrates were concentrated and dissolved in ether (10 mL). The solution was cooled to 0 ℃ with an ice-water bath and NaOH (2 mL of a 2 M aqueous solution) was added followed by H_2O_2 (2 mL of a 30% aqueous solution). After stirring at room temperature for 2 h, the reaction mixture was diluted with ether and water. The organic layer was separated and the aqueous layer extracted with EtOAc (3 × 20 mL). The combined organic phases were dried over anhydrous Na_2SO_4 and concentrated in vacuo and purified by flash column chromatography using PE/EtOAc = 5/1 as the eluent to afford **3u** (0.0610 g, 0.23mmol, 46% yield) as a colorless oil.IR (neat): 3360, 2931, 2856, 1454, 1378,1045 cm-1; Optical Rotation: $[\alpha]^{20}_{D}$ = -24.2 (c 2.10, $CHCl_3$), 99% ee determined by HPLC, HPLC conditions: Chiralcel AS-H, *n*-hexane/*i*-PrOH = 90/10, 1.0 mL/min, *n* = 220 nm, tr 7.6 (minor), 8.2 (major). ^{1}H NMR (400 MHz, $CDCl_3$): δ 7.38-7.29 (m, 4H), 7.30-7.22 (m, 1H), 4.63 (t, *J* = 6.4 Hz, 1H), 2.22 (s, 1H), 1.86-1.61 (m, 2H), 1.48-1.34 (m, 4H), 1.36 -1.21 (m, 9H), 1.18 (s, 6H); ^{13}C NMR (101 MHz, $CDCl_3$): δ144.9, 128.3, 127.4, 125.8, 74.5, 71.0, 43.8, 39.0, 30.0, 29.45, 29.40, 29.1, 25.7, 24.2. HRMS (ESI) calculated for $[M+Na]^+[C_{17}H_{28}O_2Na]^+$ requires m/z 287.1987, found m/z 287.1987.

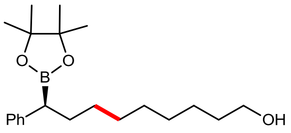

(*S*)-9-phenyl-9-(4,4,5,5-tetramethyl-1,3,2-dioxaborolan-2-yl)nonan-1-ol (2v): Prepared according to the general procedure using $Co(OAc)_2$ (0.0087 g, 0.049 mmol), **L8** (0.0249 g, 0.060 mmol), Et_2O (1 mL), **1v** (0.0.2183 g, 1.0 mmol) and HBpin (180 μL, 1.2 mmol). After 24 h, the resulting solution was added 20 ml of ether and filtered through a pad of silica gel, washed by ether (20 ml x 3). The combined filtrates were concentrated and purified by flash column chromatography using PE/EtOAc = 3/1 as the eluent to afford **2v** (0.1500 g, 0.43 mmol, 43% yield) as a colorless oil. IR (neat): 3376, 2926, 2854, 1453, 1370, 1323, 1143 cm^{-1}; Optical Rotation: $[\alpha]^{20}_D$ = +14.5 (c 1.04, $CHCl_3$), >99% ee determined by HPLC, HPLC conditions: Chiralcel AS-H, *n*-hexane/*i*-PrOH = 95/5, 1.0 mL/min, *n* = 220 nm, tr 37.9 (minor), 40.1 (major). ^1H NMR (400 MHz, $CDCl_3$): δ 7.26-7.17 (m, 4H), 7.16-7.07 (m, 1H), 3.62 (t, *J* = 6.4 Hz, 2H), 2.29 (t, *J* = 7.6 Hz, 1H), 1.92-1.77 (m, 1H), 1.67-1.49 (m, 3H), 1.35-1.22 (m, 11H), 1.20 (s, 6H), 1.18 (s, 6H); ^{13}C NMR (101 MHz, $CDCl_3$): δ 143.5, 128.3, 128.2, 125.0, 83.2, 63.1, 32.8, 32.5, 29.5, 29.4, 29.3, 29.2, 25.7, 24.62, 24.55; HRMS (EI) calculated for $[C_{21}H_{35}BO_3]^+$ requires m/z 346.2679, found m/z 346.2677.

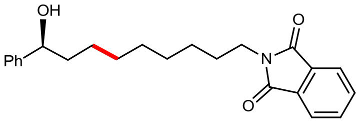

(*S*)-2-(9-hydroxy-9-phenylnonyl)isoindoline-1,3-dione (3w): Prepared according to the general procedure using $Co(OAc)_2$ (0.0045 g, 0.025 mmol), **L8** (0.0125 g, 0.030 mmol), Et_2O (1 mL), **1w** (0.1740 g, 0.5 mmol) and HBpin (180 μL, 1.2 mmol). After 20 h, the resulting solution was added 20 mL of ether and filtered through a pad of silica gel, washed by ether (10 mL x 3). The combined filtrates were concentrated and dissolved in ether (10 mL). The solution was cooled to 0 ℃ with an ice-water bath and NaOH (2 mL of a 2 M aqueous solution) was added followed by H_2O_2 (2 mL of a 30% aqueous solution). After stirring at room temperature for 2 hour, the reaction mixture was diluted with ether and water. The organic layer was separated and the aqueous layer extracted with EtOAc (3 × 20 mL). The combined organic phases were dried over anhydrous Na_2SO_4 and concentrated in vacuo and purified by flash column chromatography using PE/EtOAc = 5/1 as the eluent to afford **3w** (0.1029 g, 0.28 mmol, 56% yield) as a colorless oil. IR (neat): 3471, 2928, 2857, 1771, 1710, 1400, 1062 cm^{-1}; Optical Rotation: $[\alpha]^{20}_D$ = -10.9 (c 1.20, $CHCl_3$), 86% ee determined by HPLC, HPLC conditions: Chiralcel AS-H, *n*-hexane/*i*-PrOH = 80/20, 1.0 mL/min, *n* = 220 nm, tr 9.8

(minor), 11.0 (major). ^{1}H NMR (400 MHz, $CDCl_3$): δ 7.86-7.78 (m, 2H), 7.74-7.66 (m, 2H), 7.40-7.20 (m, 5H), 4.64 (t, J = 6.8 Hz, 1H), 3.65 (t, J = 7.2 Hz, 2H), 2.18 (s, 1H), 1.83-1.56 (m, 4H), 1.45-1.15 (m, 10H); ^{13}C NMR (101 MHz, $CDCl_3$): δ168.4, 144.9, 133.8, 132.0, 128.3, 127.3, 125.8, 123.1, 74.5, 39.0, 37.9, 29.3, 29.2, 28.9, 28.5, 26.7, 25.6. HRMS (ESI) calculated for $[M+Na]^+[C_{23}H_{27}NO_3Na]^+$ requires m/z 388.1889, found m/z 388.1892.

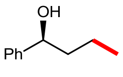

3x

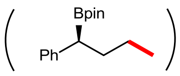

2x

(*S*)-1-phenylbutan-1-ol (3x): Prepared according to the general procedure using $Co(OAc)_2$ (0.0045 g, 0.025 mmol), **L8** (0.0125 g, 0.030 mmol), Et_2O (1 mL), **1x** (0.1328 g, 1.0 mmol) and HBpin (180 μL, 1.2 mmol). After 20 h, the resulting solution was added 20 mL of ether and filtered through a pad of silica gel, washed by ether (10 mL × 2). The combined filtrates were concentrated and dissolved in ether (10 mL). The solution was cooled to 0 ℃ with an ice-water bath and NaOH (2 mL of a 2 M aqueous solution) was added followed by H_2O_2 (2 mL of a 30% aqueous solution). After stirring at room temperature for 2 hour, the reaction mixture was diluted with ether and water. The organic layer was separated and the aqueous layer extracted with EtOAc (3 × 20 mL). The combined organic phases were dried over anhydrous Na_2SO_4 and concentrated in vacuo and purified by flash column chromatography using PE/EtOAc = 10/1 as the eluent to afford **3x** (0.0526 g, 0.35 mmol, 35% yield) as a colorless oil. IR (neat): 3381, 2957, 2869, 1455, 1025 cm^{-1}; Optical Rotation: $[\alpha]^{20}_D$ = -51.3 (c 0.83, $CHCl_3$) (lit.[24]: $[\alpha]^{20}_D$ = -47.6 (c 0.5, $CHCl_3$), 99% ee), >99% ee determined by HPLC, HPLC conditions: Chiralcel AS-H, *n*-hexane/*i*-PrOH = 98/2, 1.0 mL/min, *n* = 220 nm, tr 8.8 (minor), 10.6 (major).^{1}H NMR (400 MHz, $CDCl_3$): δ 7.39-7.32 (m, 4H), 7.30-7.24 (m, 1H), 4.66 (t, J = 6.4 Hz, 1H), 1.92 (s, 1H), 1.84-1.74 (m, 1H), 1.72-1.62 (m, 1H), 1.50-1.38 (m, 1H), 1.36-1.22 (m, 1H), 0.93 (t, J = 7.2 Hz, 3H);^{13}C NMR (101 MHz, $CDCl_3$): δ144.9, 128.4, 127.4, 125.9, 74.4, 41.2, 19.0, 13.9. All the spectroscopic data were in agreement with the reported ones.[24]The absolute configuration of **2x** was verified by comparison of the optical rotation of **3x** with previously reported data[24] and the other products were then assigned by analogy.

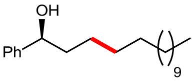

(*S*)-1-phenylpentadecan-1-ol (3y): Prepared according to the general procedure using $Co(OAc)_2$ (0.0045 g, 0.025 mmol), **L8** (0.0126 g, 0.031 mmol), Et_2O (1 mL), **1y** (0.2880 g, 1.0 mmol) and HBpin (180 μL, 1.2 mmol). After 20 h, the

resulting solution was added 20 mL of ether and filtered through a pad of silica gel, washed by ether (10 mL × 2). The combined filtrates were concentrated and dissolved in ether (10 mL). The solution was cooled to 0 ℃ with an ice-water bath and NaOH (2 mL of a 2 M aqueous solution) was added followed by H_2O_2 (2 mL of a 30% aqueous solution). After stirring at room temperature for 2 hour, the reaction mixture was diluted with ether and water. The organic layer was separated and the aqueous layer extracted with EtOAc (3 × 20 mL). The combined organic phases were dried over anhydrous Na_2SO_4 and concentrated in vacuo and purified by flash column chromatography using PE/EtOAc = 10/1 as the eluent to afford **3y** (0.2008 g, 0.66 mmol, 65% yield) as a colorless oil. IR (neat): 3368, 2919, 2852, 1463, 1062 cm^{-1};OpticalRotation: $[\alpha]^{20}_{D}$ = -18.3 (c 1.03, $CHCl_3$), 98% ee determined by HPLC, HPLC conditions: Chiralcel OD-H, *n*-hexane/*i*-PrOH = 98/2, 1.0 mL/min, *n* = 220 nm, tr 8.2 (minor), 10.1 (major).^{1}H NMR (400 MHz, $CDCl_3$): δ 7.38-7.32 (m, 4H), 7.31-7.24 (m, 1H), 4.70-4.63 (m, 1H), 1.90-1.65 (m, 3H), 1.44-1.20 (m, 24H), 0.88 (t, *J* = 6.8 Hz, 3H); ^{13}C NMR (101 MHz, $CDCl_3$): δ144.9, 128.4, 127.5, 125.9, 74.7, 39.1, 31.9, 29.68, 29.66, 29.64, 29.57, 29.53, 29.52, 29.4, 25.8, 22.7, 14.1. HRMS (ESI) calculated for $[M+H]^+[C_{21}H_{37}O]^+$ requires m/z 305.2844, found m/z 305.2831.

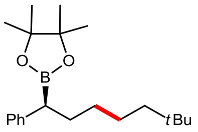

(*S*)-2-(6,6-dimethyl-1-phenylheptyl)-4,4,5,5-tetramethyl-1,3,2-dioxaborolane (2z): Prepared according to the general procedure using $Co(OAc)_2$ (0.0046 g, 0.026 mmol), **L8** (0.0125 g, 0.030 mmol), Et_2O (1 mL), **1z** (0.2020 g, 1.0 mmol) and HBpin (180 μL, 1.2 mmol). After 20 h, the resulting solution was added 20 mL of ether and filtered through a pad of silica gel, washed by ether (10 mL × 2). The combined filtrates were concentrated and purified by flash column chromatography using PE/EtOAc = 20/1 as the eluent to afford **2z** (0.2741 g, 0.83 mmol, 83% yield) as a colorless oil. IR (neat): 2934, 1465, 1367, 1324, 1146 cm^{-1}; Optical Rotation: $[\alpha]^{20}_{D}$ = +17.8 (c 1.02, $CHCl_3$), 98% ee determined by HPLCafter oxidized to the corresponding alcohol, HPLC conditions: Chiralcel AS-H, *n*-hexane/*i*-PrOH = 98/2, 1.0 mL/min, *n* = 220 nm, tr 5.9 (minor), 6.4 (major).^{1}H NMR (400 MHz, $CDCl_3$): δ 7.28-7.18 (m, 4H), 7.15-7.09 (m, 1H), 2.29 (t, *J* = 8.0 Hz, 1H), 1.91-1.79 (m, 1H), 1.70-1.60 (m, 1H), 1.29-1.17 (m, 16H), 1.15-1.08 (m, 2H), 0.84 (s, 9H); ^{13}C NMR (101 MHz, $CDCl_3$): δ143.5, 128.4, 128.2, 125.0, 83.2, 44.1, 32.7, 30.3, 29.4, 24.62, 24.57.HRMS (ESI) calculated for $[M+H]^+[C_{21}H_{36}BO_2]^+$ requires m/z 331.2808, found m/z 331.2812.

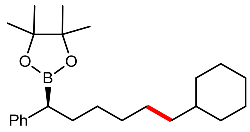

(*S*)-2-(6-cyclohexyl-1-phenylhexyl)-4,4,5,5-tetramethyl-1,3,2-dioxaborolane (2aa): Prepared according to the general procedure using $Co(OAc)_2$ (0.0045 g, 0.025 mmol), **L8** (0.0123 g, 0.030 mmol), Et_2O (1 mL), **1aa** (0.2426 g, 1.0 mmol) and HBpin (180 μL, 1.2 mmol). After 20 h, the resulting solution was added 20 mL of ether and filtered through a pad of silica gel, washed by ether (10 mL × 2). The combined filtrates were concentrated and purified by flash column chromatography using PE/EtOAc = 20/1 as the eluent to afford **2aa** (0.3203 g, 0.86 mmol, 86% yield) as a colorless oil. IR (neat): 2981, 2925, 2852, 1454, 1364, 1324, 1145 cm^{-1}; Optical Rotation: $[\alpha]^{20}_{D}$ = +14.2 (c 0.85, $CHCl_3$), 99% ee determined by HPLC after oxidized to the corresponding alcohol, HPLC conditions: Chiralcel AS-H, *n*-hexane/*i*-PrOH = 98/2, 1.0 mL/min, *n* = 220 nm, tr 6.0 (minor), 6.5 (major). ^1H NMR (400 MHz, $CDCl_3$): *δ* 7.27-7.17 (m, 4H), 7.14-7.08 (m, 1H), 2.29 (t, *J* = 7.6 Hz, 1H), 1.87-1.78 (m, 1H), 1.71-1.57 (m, 6H), 1.31-1.06 (m, 24H), 0.90-0.77 (m, 2H); ^{13}C NMR (101 MHz, $CDCl_3$): *δ*143.5, 128.3, 128.2, 125.0, 83.2, 37.6, 37.5, 33.4, 32.6, 29.9, 29.3, 26.8, 26.7, 26.4, 24.6, 24.5. HRMS (ESI) calculated for $[M+H]^+[C_{24}H_{40}BO_2]^+$ requires m/z 371.3121, found m/z 371.3128.

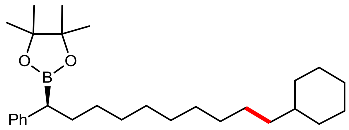

(*S*)-2-(10-cyclohexyl-1-phenyldecyl)-4,4,5,5-tetramethyl-1,3,2-dioxaborolane (2ab): Prepared according to the general procedure using $Co(OAc)_2$ (0.0089 g, 0.050 mmol), **L8** (0.0249 g, 0.060 mmol), Et_2O (1 mL), **1ab** (0.2980 g, 1.0 mmol) and HBpin (180 μL, 1.2 mmol). After 20 h, the resulting solution was added 20 mL of ether and filtered through a pad of silica gel, washed by ether (10 mL × 2). The combined filtrates were concentrated and purified by flash column chromatography using PE/EtOAc = 20/1 as the eluent to afford **2ab** (0.2482 g, 0.58 mmol, 58% yield) as a colorless oil. IR (neat): 2925, 2853, 1455, 1366, 1324, 1145 cm^{-1}; Optical Rotation: $[\alpha]^{20}_{D}$ = +14.3 (c 1.11, $CHCl_3$); 99% ee determined by HPLC after oxidized to the corresponding alcohol, HPLC conditions: Chiralcel OD-H, *n*-hexane/*i*-PrOH = 98/2, 1.0 mL/min, *n* = 220 nm, tr 8.8 (minor), 10.7 (major). ^1H NMR (400 MHz, $CDCl_3$): *δ* 7.27-7.18 (m, 4H), 7.15-7.08 (m, 1H), 2.29 (t, *J* = 8.0 Hz, 1H), 1.86-1.77 (m, 1H), 1.75-1.57 (m, 6H), 1.31-1.08 (m, 32H), 0.90-0.78 (m, 2H); ^{13}C NMR (101 MHz, $CDCl_3$): *δ*143.5, 128.3, 128.2, 125.0, 83.2, 37.7, 37.6, 33.4, 32.6, 30.0, 29.7, 29.6, 29.5, 29.3, 26.9, 26.8, 26.4, 24.6, 24.5. HRMS (ESI) calculated for

$[M+Na]^+[C_{28}H_{47}BO_2Na]^+$ requires m/z 449.3567, found m/z 449.3577.

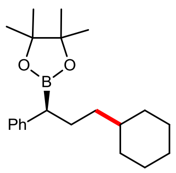

(*S*)-2-(3-cyclohexyl-1-phenylpropyl)-4,4,5,5-tetramethyl-1,3,2-dioxaborolane (2ac): Prepared according to the general procedure using $Co(OAc)_2$ (0.0045 g, 0.025 mmol), **L8** (0.0125 g, 0.030 mmol), Et_2O (1 mL), **1ac** (0.2006 g, 1.0 mmol) and HBpin (180 μL, 1.2 mmol). After 20 h, the resulting solution was added 20 mL of ether and filtered through a pad of silica gel, washed by ether (10 mL × 2). The combined filtrates were concentrated and purified by flash column chromatography using PE/EtOAc = 20/1 as the eluent to afford **2ac** (0.2443 g, 0.74 mmol, 74% yield) as a colorless oil. IR (neat): 2980, 2924, 2852, 1453, 1368, 1324, 1147 cm^{-1}; Optical Rotation: $[\alpha]^{20}_D$ = +15.1 (c 1.1, $CHCl_3$), 99% ee determined by HPLC after oxidized to the corresponding alcohol, HPLC conditions: Chiralcel AS-H, *n*-hexane/*i*-PrOH = 99.1/0.9, 1.0 mL/min, *n* = 220 nm, tr 10.5 (minor), 11.1 (major). ^1H NMR (400 MHz, $CDCl_3$): *δ* 7.27-7.18 (m, 4H), 7.14-7.08 (m, 1H), 2.25 (t, *J* = 7.6 Hz, 1H), 1.89-1.79 (m, 1H), 1.73-1.58 (m, 6H), 1.26 -1.07 (m, 18H), 0.90-0.78 (m, 2H); ^{13}C NMR (101 MHz, $CDCl_3$): *δ*143.5, 128.3, 128.2, 125.0, 83.2, 37.7, 37.1, 33.4, 33.3, 29.9, 26.7, 26.4, 24.6, 24.5. HRMS (EI) calculated for$[C_{21}H_{33}BO_2]^+$ requires m/z 328.2574, found m/z 328.2574.

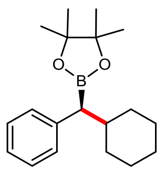

(*S*)-2-(cyclohexyl(phenyl)methyl)-4,4,5,5-tetramethyl-1,3,2-dioxaborolane (2ad): Prepared according to the general procedure using $Co(OAc)_2$ (0.0179 g, 0.010mmol), **L8** (0.0495 g, 0.12mmol), Et_2O (1 mL), **1ad** (0.1744 g, 1.0 mmol) and HBpin (180 μL, 1.2 mmol). After 48 h, the resulting solution was added 20 ml of ether and filtered through a pad of silica gel, washed by ether (10 mL × 2). The combined filtrates were concentrated and purified by flash column chromatography using PE/EtOAc = 100/1 as the eluent to afford **2ad** (0.1670 g, 0.55 mmol, 55% yield) a colorless oil. IR (neat): 2922, 2851, 1449, 1356, 1318, 1143 cm^{-1}**;** Optical Rotation: $[\alpha]^{20}_D$ = +13.0 (c 0.99, $CHCl_3$), >99 % ee determined by HPLC, HPLC conditions: Chiralcel AS-H, *n*-hexane/*i*-PrOH = 98/2, 1.0 mL/min, *n* = 220 nm, tr 8.3 (minor), 10.0 (major). ^1H NMR (400 MHz, $CDCl_3$): *δ* 7.25-7.16 (m, 4H), 7.15-7.08 (m, 1H), 2.04 (d, *J* = 10.4 Hz, 1H), 1.87-1.66 (m, 3H), 1.48-1.23 (m, 4H), 1.19 (s, 6H), 1.17 (s, 6H), 1.15-0.95 (m, 3H), 0.86-0.68 (m, 1H); ^{13}C NMR (101 MHz, $CDCl_3$): *δ* 141.7, 129.1, 128.0, 125.1, 83.1, 40.2, 33.8, 32.4, 26.6, 26.5, 26.3, 24.6, 24.5; HRMS

(EI) calculated for $[C_{19}H_{29}BO_2]^+$ requires m/z 300.2261, found m/z 300.2258.

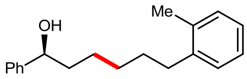

(*S*)-1-phenyl-6-(o-tolyl)hexan-1-ol (3ae): Prepared according to the general procedure using $Co(OAc)_2$ (0.0045 g, 0.025 mmol), **L8** (0.0126 g, 0.031 mmol), Et_2O (1 mL), **1ae** (0.1259 g, 0.5 mmol) and HBpin (90 μL, 0.6 mmol). After 20 h, the resulting solution was added 20 mL of ether and filtered through a pad of silica gel, washed by ether (10 mL × 2). The combined filtrates were concentrated and dissolved in ether (10 mL). The solution was cooled to 0 ℃ with an ice-water bath and NaOH (2 mL of a 2 M aqueous solution) was added followed by H_2O_2 (2 mL of a 30% aqueous solution). After stirring at room temperature for 2 hour, the reaction mixture was diluted with ether and water. The organic layer was separated and the aqueous layer extracted with EtOAc (3 × 20 mL). The combined organic phases were dried over anhydrous Na_2SO_4 and concentrated in vacuo and purified by flash column chromatography using PE/EtOAc = 10/1 as the eluent to afford **3ae** (0.0903 g, 0.34mmol, 67% yield, 11/1 ratio) as a colorless oil.IR (neat): 3349, 2930, 2857, 1492, 1454, 1027 cm^{-1}; Optical Rotation: $[\alpha]^{20}_D$ = -21.3 (c 1.40, $CHCl_3$), 98% ee determined by HPLC, HPLC conditions: Chiralcel OJ-H, *n*-hexane/*i*-PrOH = 98/2, 1.0 mL/min, *n* = 220 nm,tr 41.3 (minor), 44.6 (major). ^1H NMR (400 MHz, $CDCl_3$) δ 7.38-7.03 (m,9H), 4.89 (t, *J* = 6.4 Hz, 0.08H), 4.63 (t, *J* = 6.4 Hz, 0.95H), 2.63-2.52 (m, 2H), 2.30 (s, 0.24H), 2.27 (s, 2.55H), 1.92 (s, 1H), 1.86-1.23 (m, 8H).HRMS (ESI) calculated for $[M+Na]^+[C_{19}H_{24}ONa]^+$ requires m/z 291.1725, found m/z 291.1735.

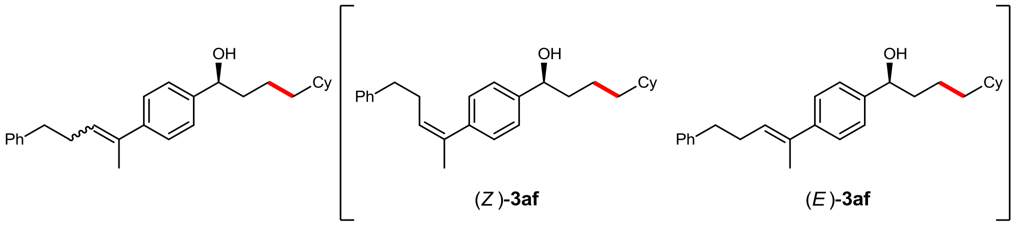

(*Z*)-**3af** (*E*)-**3af**

Prepared according to the general procedure using $Co(OAc)_2$ (0.0023 g, 0.013 mmol), **L8** (0.0063 g, 0.015 mmol), Et_2O (0.5 mL), **1af** (0.0901 g, 0.25 mmol) and HBpin (40 μL, 0.26 mmol). After 20 h, the resulting solution was added 20 mL of ether and filtered through a pad of silica gel, washed by ether (10 mL x 3). The combined filtrates were concentrated and dissolved in ether (10 mL). The solution was cooled to 0 ℃ with an ice-water bath and NaOH (1.0 mL of a 2 M aqueous solution) was added followed by H_2O_2 (1.0 mL of a 30% aqueous solution). After stirring at room

temperature for 2 hour, the reaction mixture was diluted with ether and water. The organic layer was separated and the aqueous layer extracted with EtOAc (3× 20 mL). The combined organic phases were dried over anhydrous Na_2SO_4 and concentrated in vacuo and purified by flash column chromatography using PE/EtOAc = 20/1 and PE/EtOAc = 10/1 as the eluent to afford a mixture of (*Z*)-**3af** and (*E*)-**3af** (0.0504 g, 0.13 mmol, 54% yield, (*Z*)-**3af** /(*E*)-**3af** = 1.26/1) as a colorless oil. 94 % ee for (*Z*)-**3af** and 94% ee for (*E*)-**3af** determined by HPLC, HPLC conditions: Chiralcel AS-H, *n*-hexane/*i*-PrOH = 98/2, 1.0 mL/min, *n* = 220 nm, $tr_{(Z)\text{-}3af}$ 7.3 (minor), 8.7 (major) and $tr_{(E)\text{-}3af}$ 10.9 (minor), 14.5 (major). ^{1}H NMR (400 MHz, $CDCl_3$) δ 7.36-7.05 (m, 9H), 5.86-5.78 (m, 0.42H), 5.53-5.45 (m, 0.53H), 4.64 (t, *J* = 6.8 Hz, 1H), 2.76 (t, *J* = 7.6 Hz, 0.91H), 2.64 (t, *J* = 7.6 Hz, 1.17H), 2.57-2.48 (m, 0.93H), 2.34-2.24 (m, 1.14H), 2.01 (s, 1.68H), 1.96 (s, 1.30H), 1.84-1.61 (m, 7H), 1.49-1.10 (m, 9H), 0.92-0.78 (m, 2H).

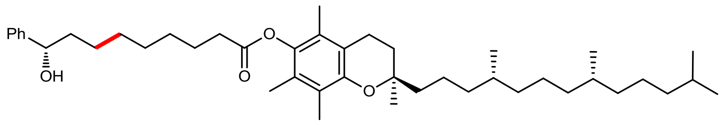

(*S*)-(R)-2,5,7,8-tetramethyl-2-((4R,8R)-4,8,12-trimethyltridecyl)chroman-6-yl-9-hydroxy-9-phenylnonanoate (3ag): Prepared according to the general procedure using $Co(OAc)_2$ (0.0045 g, 0.025 mmol), **L8** (0.0126 g, 0.031 mmol), Et_2O (1 mL), **1ag** (0.3220 g, 0.5 mmol) and HBpin (90 µL, 0.6 mmol). After 20 h, the resulting solution was added 20 mL of ether and filtered through a pad of silica gel, washed by ether (10 mL x 3). The combined filtrates were concentrated and dissolved in ether (10 mL). The solution was cooled to 0 ℃ with an ice-water bath and NaOH (2 mL of a 2 M aqueous solution) was added followed by H_2O_2 (2 mL of a 30% aqueous solution). After stirring at room temperature for 2 hour, the reaction mixture was diluted with ether and water. The organic layer was separated and the aqueous layer extracted with EtOAc (3× 20 mL). The combined organic phases were dried over anhydrous Na_2SO_4 and concentrated in vacuo and purified by flash column chromatography using PE/EtOAc = 5/1 as the eluent to afford **3ag** (0.1989 g, 0.30 mmol, 60% yield) as a colorless oil. IR (neat): 3559, 2930, 2857, 1753, 1459, 1148, 1102 cm^{-1}; Optical Rotation: $[\alpha]^{20}_D$ = -7.0 (c 0.91, $CHCl_3$), 98% de determined by HPLC, HPLC conditions: Chiralcel AD-H, *n*-hexane/*i*-PrOH = 95/5, 1.0 mL/min, *n* = 220 nm, tr 8.2 (major), 9.3 (minor).^{1}H NMR (400 MHz, $CDCl_3$): *δ* 7.37-7.30 (m, 4H), 7.30-7.22 (m, 1H), 4.64 (t, *J* = 6.4 Hz, 1H), 2.62-2.51 (m, 4H), 2.12-1.90 (m, 10H), 1.86-1.63 (m, 6H), 1.59-0.98 (m, 32H), 0.90-0.82 (m,

12H); ^{13}C NMR (101 MHz, $CDCl_3$): δ 172.3, 149.3, 144.9, 140.5, 128.4, 127.4, 126.6, 125.8, 124.8, 122.9, 117.3, 75.0, 74.6, 39.3, 39.0, 37.4, 37.2, 34.1, 32.8, 32.7, 31.1, 29.3, 29.2, 29.1, 27.9, 25.7, 25.1, 24.8, 24.4, 22.7, 22.6, 21.0, 20.6, 19.7, 19.6, 12.9, 12.1, 11.8.HRMS (ESI) calculated for $[M+Na]^+[C_{44}H_{70}O_4Na]^+$ requires m/z 685.5172, found m/z 685.5176.

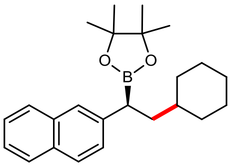

(*S*)-2-(2-cyclohexyl-1-(naphthalen-2-yl)ethyl)-4,4,5,5-tetramethyl-1,3,2-dioxaborolane (2ah): Prepared according to the general procedure using $Co(OAc)_2$ (0.0045 g, 0.025 mmol), **L8** (0.0125 g, 0.030 mmol), Et_2O (1 mL), **1ah** (0.2369 g, 1.0 mmol) and HBpin (180 µL, 1.2 mmol). After 20 h, the resulting solution was added 20 mL of ether and filtered through a pad of silica gel, washed by ether (10 mL × 2). The combined filtrates were concentrated and purified by flash column chromatography using PE/EtOAc = 20/1 as the eluent to afford **2ah** (0.1990 g, 0.55 mmol, 55% yield) as a white solid. IR (neat): 2922, 2850, 1448, 1323, 1141 cm^{-1}; Optical Rotation: $[\alpha]^{20}_D$ = +20.9 (c 1.15, $CHCl_3$), 99% ee determined by HPLC after oxidized to the corresponding alcohol, HPLC conditions: Chiralcel AS-H, *n*-hexane/*i*-PrOH = 98/2, 1.0 mL/min, *n* = 220 nm, tr 13.8 (minor), 18.2 (major).^{1}H NMR (400 MHz, $CDCl_3$): δ 7.82-7.70 (m, 3H), 7.63 (s, 1H), 7.46-7.35 (m, 3H), 2.63 (t, J = 8.0 Hz, 1H), 1.90-1.56 (m, 7H), 1.28-1.16 (m, 16H), 1.00-0.82 (m, 2H); ^{13}C NMR (101 MHz, $CDCl_3$): δ141.2, 133.8, 131.7, 127.6, 127.49, 127.46, 127.4, 126.1, 125.5, 124.7, 83.3, 39.7, 36.5, 33.7, 32.9, 29.5, 26.6, 26.31, 26.25, 24.6, 24.5.HRMS (ESI) calculated for $[M+Na]^+[C_{24}H_{33}BO_2Na]^+$ requires m/z 387.2471, found m/z 387.2478.

Gram scale reaction:

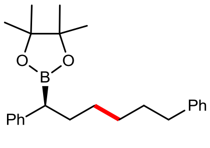

(*S*)-2-(1,6-diphenylhexyl)-4,4,5,5-tetramethyl-1,3,2-dioxaborolane (2a): Prepared according to the general procedure using $Co(OAc)_2$ (0.0088 g, 0.050 mmol), **L8** (0.0249 g, 0.060 mmol), Et_2O (5.0 mL), **1a** (1.1822 g, 5.0 mmol) and HBpin (0.90 µL, 6.0 mmol). After 20 h, the resulting solution was added 20 mL of ether and filtered through a pad of silica gel, washed by ether (10 mL x 4). The combined filtrates were concentrated and purified by flash column chromatography using PE/EtOAc = 20/1 as the eluent to afford **2a** (1.7324 g, 4.76 mmol, 95% yield) as a colorless oil. Optical Rotation: $[\alpha]^{20}_D$ = +15.8 (c 1.04, $CHCl_3$); 99% ee determined by HPLC after oxidized to the corresponding alcohol,

HPLC conditions: Chiralcel OJ-H, *n*-hexane/*i*-PrOH = 99/1, 0.5 mL/min, *n* = 220 nm, tr 10.7 (minor),12.3 (major).

Applications of chiral boronic ester

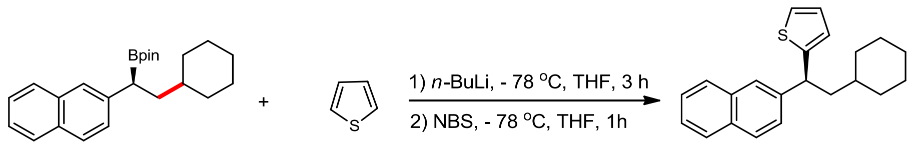

(*S*)-2-(2-cyclohexyl-1-(naphthalen-2-yl)ethyl)thiophene (4): Prepared according to the previous reported literature,[25] a solution of thiophene (32 μL, 0.40 mmol, 1.3 eq.) in THF (2.0 mL) was cooled to -78 ℃ and treated with *n*-BuLi (160 μL, 0.40 mmol, 2.5 M in hexanes, 1.3 eq.). The mixture was stirred at -78 ℃ for 10 min and at room temperature for 3 h. The mixture was cooled to –78 ℃ and a solution of **2ah** (0.1098 g, 0.30 mmol, 1.0 eq.) in THF (1.5 mL) was added dropwise. The mixture was stirred at -78 ℃ for 1 h and a solution of NBS (0.0720 g, 0.4 mmol, 1.3 eq.) in THF (1.0 mL) was added dropwise. After 1 h at -78 ℃, $Na_2S_2O_3$ sat. was added and the reaction mixture was allowed to warm to room temperature. The organic layer was separated and the aqueous layer was exracted with EtOAc (2x30 mL). The combined organic phases were dried over anhydrous Na_2SO_4 and concentrated in vacuo. The resulting crude product was purified by column chromatography (silica gel, 200-300 mesh) using PE/EtOAc = 50/1 as the eluent to afford **4** (0.0454 g, 47% yield) as a colorless oil. IR (neat): 2921, 2850, 1447, 1262 cm^{-1}; Optical Rotation:$[\alpha]^{20}_{D}$ = +51.6 (c 1.0, $CHCl_3$), 98% ee determined by HPLC, HPLC conditions: Chiralcel OJ-H, *n*-hexane/*i*-PrOH = 99/1, 0.5 mL/min, *n* = 220 nm, tr 22.2 (major), 29.7 (minor).^{1}H NMR (400 MHz, $CDCl_3$): δ 7.82-7.74 (m, 3H), 7.70 (s, 1H), 7.50-7.34 (m, 3H), 7.12 (d, J = 4.8 Hz, 1H), 6.91 (dd, J = 4.8, 4.0 Hz, 1H), 6.84 (d, J = 7.2 Hz, 1H), 4.45 (dd, J = 8.4, 7.6 Hz, 1H), 2.05 (dd, J = 7.6, 7.2 Hz, 2H), 1.85 (d, J = 12.4 Hz, 1H), 1.75-1.54 (m, 4H), 1.29–0.89 (m, 6H); ^{13}C NMR (101 MHz, $CDCl_3$): δ 150.0, 142.3, 133.5, 132.3, 128.2, 127.7, 127.6, 126.5, 126.1, 126.0, 125.9, 125.4, 123.7, IR (neat): 2981, 2933, 1457, 1369, 1324, 1144, 1070 cm-1;123.4, 44.9, 43.6, 34.9, 33.6, 32.9, 26.6, 26.1, 26.0.All the spectroscopic data were in agreement with the reported ones.[26]

Utilization of a mixture of geometrical and positional alkene isomers

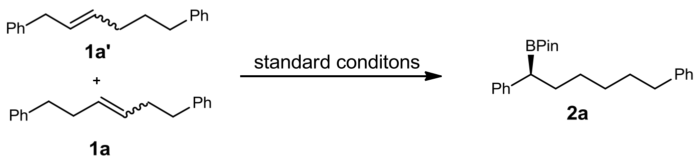

Prepared according to the general procedure using $Co(OAc)_2$ (0.0045 g, 0.025 mmol), **L8** (0.0127 g, 0.031 mmol), Et_2O (1 mL), **1a/1a'** (0.2360 g, 1.0 mmol, 1/1 mixture) and HBpin (180 μL, 1.2 mmol). After 20 h, the resulting solution was added 20 mL of ether and filtered through a pad of silica gel, washed by ether (10 mL × 2). The combined filtrates were concentrated and purified by flash column chromatography using PE/EtOAc = 20/1 as the eluent to afford **2a** (0.3456 g, 0.95 mmol, 95% yield) as a colorless oil. Optical Rotation:$[\alpha]^{20}_D$ = +15.4 (c 1.35, $CHCl_3$), 99% ee determined by HPLC after oxidized to the corresponding alcohol, HPLC conditions: Chiralcel AD-H, *n*-hexane/*i*-PrOH = 98/2, 1.0 mL/min, *n* = 220 nm, tr 22.4 (major), 25.1 (minor). ^{1}H NMR (400 MHz, $CDCl_3$): δ 7.30-7.08 (m, 10H), 2.56 (t, J = 7.6 Hz, 2H), 2.28 (t, J = 7.6 Hz, 1H), 1.90-1.78 (m, 1H), 1.70-1.53 (m, 3H), 1.39-1.24 (m, 4H), 1.20 (s, 6H), 1.18 (s, 6H).

Deuterium labeling experiment

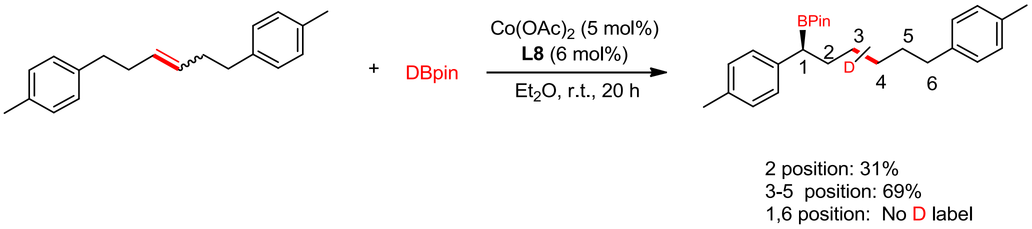

Prepared according to the general procedure using $Co(OAc)_2$ (0.0088 g, 0.050 mmol), **L8** (0.0249 g, 0.060 mmol), Et_2O (1 mL), **1ai** (0.2649 g, 1.0 mmol) and DBpin(180 μL,1.2 mmol). After 20 h, the resulting solution was added 20 mL of ether and filtered through a pad of silica gel, washed by ether (10 mL × 2). The combined filtrates were concentrated and purified by flash column chromatography using PE/EtOAc = 20/1 as the eluent to afford **2ai** (0.3828 g, 0.97 mmol, 97% yield) as a colorless oil.IR (neat): 2925, 2855, 1366, 1322, 1144 cm^{-1}; Optical Rotation: $[\alpha]^{20}_D$ = +17.7 (c 0.90, $CHCl_3$), 99% ee determined by HPLC after oxidized to the corresponding alcohol, HPLC conditions: Chiralcel AS-H, *n*-hexane/*i*-PrOH = 98/2, 1.0 mL/min, *n* = 220 nm, tr 9.7 (minor), 11.7 (major). ^{1}H NMR (400 MHz, C_6D_6) δ 7.30 (d, J = 7.6 Hz, 2H), 7.04 (d, J = 8.0 Hz, 2H), 7.01-6.98 (m, 4H), 2.52-2.42 (m, 3H), 2.16 (s, 3H),2.14 (s, 3H), 2.11-1.98 (m, 0.84 H), 1.89-1.76 (m, 0.85H), 1.60-49 (m, 1.90 H), 1.47-1.24 (m, 3.41H), 1.02 (s, 6H), 1.00 (s, 6H); ^{2}H

NMR (76.8 MHz, $CHCl_3$+$CDCl_3$) δ, 1.87, 1.63, 1.37.

The parallel reaction of the alkene (*E*)-1aj and (*Z*)-1aj

Prepared according to the general procedure using $Co(OAc)_2$ (0.0044 g, 0.025 mmol), **L8** (0.0124 g, 0.03 mmol), Et_2O (1.0 mL), (*Z*)-**1aj** (0.1040 g, 0.5 mmol) and HBpin (90 μL, 0.6 mmol). After the reaction was complete, the resulting solution was added 20 ml of ether and filtered through a pad of silica gel, washed by ether (10 ml x 2). The combined filtrates were concentrated and analysed by ^{1}H NMR using TMSPh (10 uL) as the internal standard.

Supplementary Table 4.The parallel reaction of the alkene (*E*)-**1aj** and (*Z*)-**1aj**

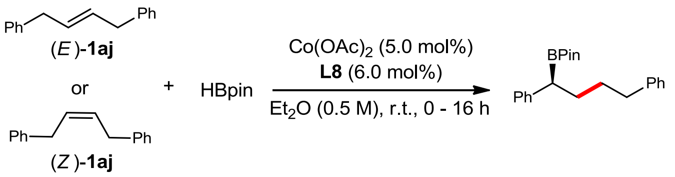

Entry	Time/h	Yield (from (*E*)-**1aj**)	Yield (from (*Z*)-**1aj**)
1	0	0%	0%
2	3	34%	33%
3	5	54%	52%
4	7	72%	71%
5	11	88%	85%
6	16	96%	97%

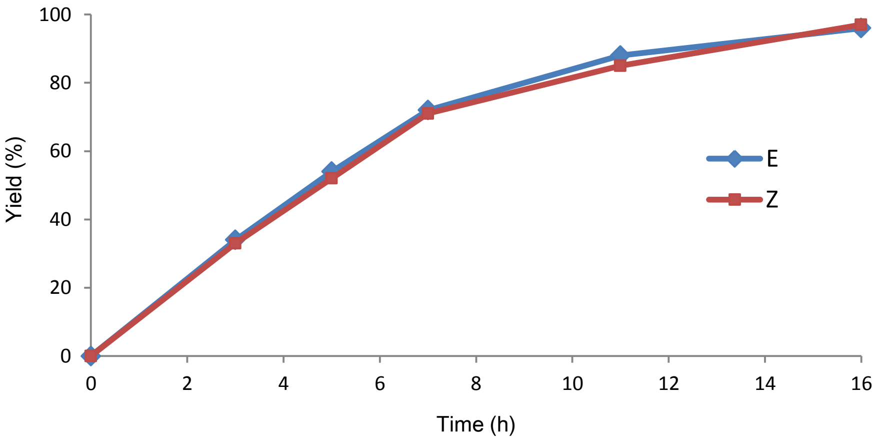

Supplementary Figure 246 The effect of stereochemistry of alkenes on the kinetics of the reaction

Time course reaction:

Prepared according to the general procedure using $Co(OAc)_2$ (0.0022 g, 0.0125 mmol), **L8** (0.0062 g, 0.015 mmol), Et_2O (0.5 mL), **1b** (0.1072 g, 0.5 mmol) and HBpin (90 μL, 0.6 mmol). After the reaction was complete, the resulting solution was added 20 ml of ether and filtered through a pad of silica gel, washed by ether (10 ml x 2). The combined filtrates were concentrated and analysed by ^1H NMR using TMSPh (10 uL) as the internal standard.

Supplementary Table 5. Time course of alkene isomeration-hydroboration

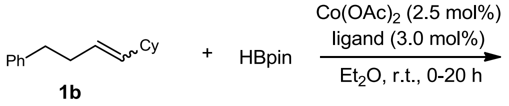

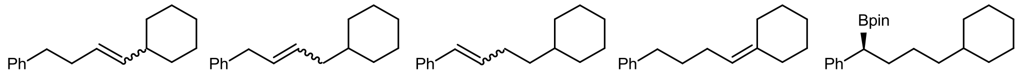

Entry	time/h	1b[a]	1ba[a]	1bb[a]	1bc[a]	2b[a]
1	0	100.0%	0.0%	0.0%	0.0%	0.0%
2	2	53.5%	21.0%	0.2%	16.5%	9.0%
3	4	33.3%	17.5%	0.1%	23%	19.5%
4	6	22.0%	13.7%	1.6%	23%	36.0%
5	8	14.5%	9.3%	0.3%	25.5%	43.0%
6	10	3.5%	2.3%	0.0%	24.5%	64.5%
7	13	2.0%	0.7%	0.0%	14.5%	77.0%
8	16	0.0%	0.0%	0.0%	2.1%	89.5%
9	20	0.0%	0.0%	0.0%	0.85%	91.0%

[a]Yields were determined by ^1H NMR using TMSPh as the internal standard, and is an average of two runs (0.5 mmol scale).

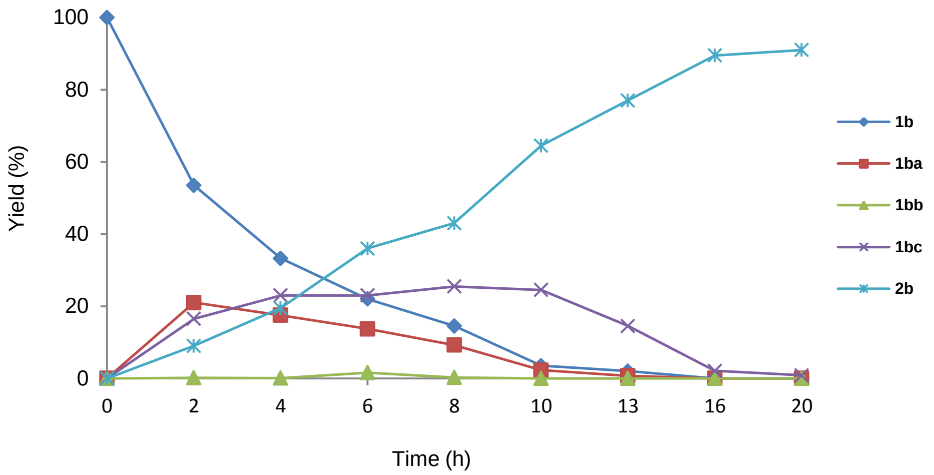

Supplementary Figure 247 The time course study of **1b**.

Isomerization through alkene bearing branched chains with a pre-exisiting stereocenter.

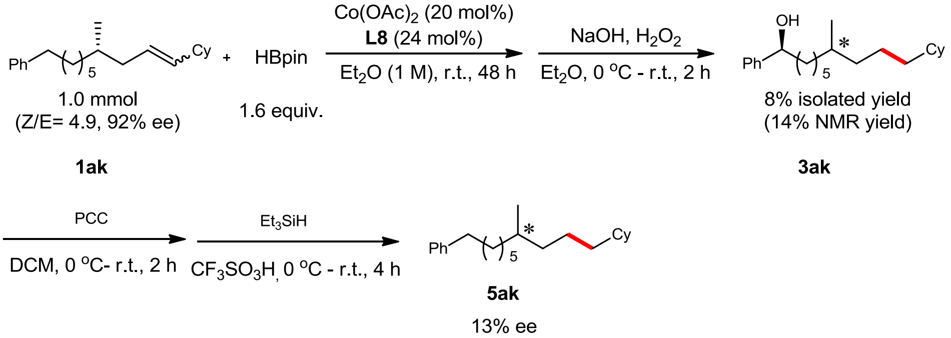

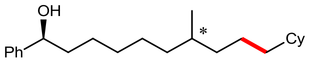

(1*S*,7*S*)-10-cyclohexyl-7-methyl-1-phenyldecan-1-ol (3ak): Prepared according to the general procedure using $Co(OAc)_2$ (0.0356 g, 0.20 mmol), **L8** (0.0990 g, 0.24 mmol), Et_2O (1 mL), **1ak** (0.3139 g, 1.0 mmol) and HBpin (240 μL, 1.6 mmol). After 48 h, the resulting solution was added 20 mL of ether and filtered through a pad of silica gel, washed by ether (10 mL × 2). The combined filtrates were concentrated and dissolved in ether (10 mL). The solution was cooled to 0 ℃ with an ice-water bath and NaOH (2 mL of a 2 M aqueous solution) was added followed by H_2O_2 (2 mL of a 30% aqueous solution). After stirring at room temperature for 2 hour, the reaction mixture was diluted with ether and water. The organic layer was separated and the aqueous layer extracted with EtOAc (3 × 20 mL). The combined organic phases were dried over anhydrous Na_2SO_4 and concentrated in vacuo and purified by flash column chromatography using PE/EtOAc = 40/1 as the eluent to afford **3ak** (0.0285 g, 0.86 mmol, 9% yield) as a colorless oil. IR (neat): 3355, 2923, 2852, 1454,

cm[-1]; 98% de determined by HPLC, HPLC conditions: Chiralcel OD-H, n-hexane/i-PrOH = 98/2, 1.0 mL/min, n = 220 nm, tr 8.3 (minor), 9.9 (major). ^{1}H NMR (400 MHz, $CDCl_3$): δ 7.40-7.32 (m, 4H), 7.31-7.26 (m, 1H), 4.75-4.60 (m, 1H), 1.82-1.64 (m, 7H), 1.41-1.02 (m, 20H), 0.90-0.79 (m, 5H); ^{13}C NMR (101 MHz, $CDCl_3$): δ 144.9, 128.4, 127.5, 125.9, 74.7, 39.1, 37.9, 37.7, 37.3, 37.0, 33.5, 33.4, 32.7, 29.9, 26.9, 26.8, 26.5, 25.9, 24.2, 19.7. HRMS (EI) calculated for $[C_{23}H_{38}O]^+$ requires m/z 330.2923, found m/z 330.2921.

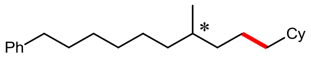

(*S*)-(10-cyclohexyl-7-methyldecyl)benzene (5ak): Alkane **5ak** was prepared from **3ak** according to the literature[27, 28] to determine the ee value of alkyl chain on **3ak**. 13% ee determined by HPLC, HPLC conditions: Chiralcel OJ-H*3, n-hexane/i-PrOH = 100/0, 0.25 mL/min, *n* = 220 nm, tr 55.0 (major), 56.3 (minor). ^{1}H NMR (400 MHz, $CDCl_3$): δ 7.30-7.24 (m, 2H), 7.21-7.13 (m, 3H), 2.60 (t, J = 7.6 Hz, 2H), 1.74-1.57 (m, 7H), 1.36-1.01 (m, 19H), 0.93-0.76 (m, 5H); ^{13}C NMR (101 MHz, $CDCl_3$): δ 143.0, 128.4, 128.2, 125.5, 37.9, 37.7, 37.4, 37.1, 36.0, 33.5, 33.4, 32.7, 31.5, 29.9, 29.4, 27.0, 26.8, 26.5, 24.2, 19.7. HRMS (EI) calculated for $[C_{23}H_{38}]^+$ requires m/z 314.2974, found m/z 314.2971 .

Linear effect reaction

L7 with different ee value was prepared by mixing **L7** and **L7$_{rac}$** in THF solution.

Prepared according to the general procedure using $Co(OAc)_2$ (0.025 mmol), **L7** (0.030 mmol), Et_2O (1 mL), **1a** (0.5 mmol) and HBpin (90 μL, 0.6 mmol). After 20 h, the resulting solution was added 20 mL of ether and filtered through a pad of silica gel, washed by ether (10 mL × 2). The combined filtrates were concentrated and purified by flash column chromatography using PE/EtOAc = 20/1 as the eluent to afford **2a** as a colorless oil and ee value was determined by HPLC.

Supplementary Table 6. Linear effect reaction

Entry	ee value of ligand[a]	ee value of product[a]
1	0.0%	0.0%
2	21.3%	20.8%
3	41.3%	35.1%
4	60.4%	53.3%

5	80.5%	73.6%
6	99.6%	98.0%

[a]ee value was determined by HPLC, and is an average of two runs (0.5 mmol scale).

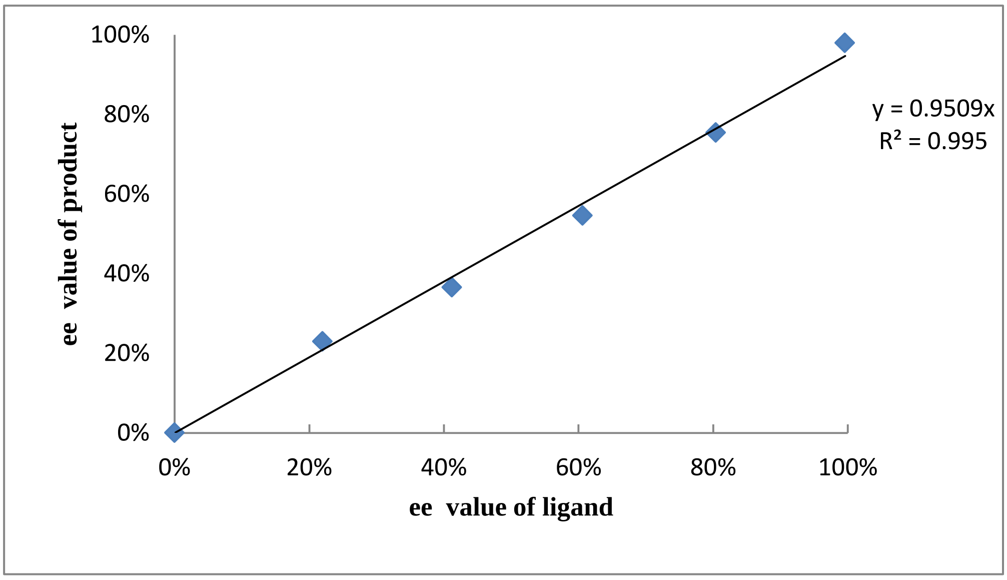

Supplementary Figure 248 The Linear effect reaction

Supplementary References

1. Chen, J. H., Xi, T., Ren, X., Cheng, B., Guo, J., Lu, Z. Asymmetric cobalt catalysts for hydroboration of 1,1-disubstituted alkenes. *Org. Chem. Front.* **1**, 1306-1309 (2014).

2. Zhang, H. Y., Lu, Z. Dual-stereocontrol asymmetric cobalt-catalyzed hydroboration of sterically hindered Styrenes. *ACS Catal.* **6**, 6596-6600 (2016).

3. Chen, X., Lu, Z. Iminophenyl oxazolinylphenylamine for enantioselective cobalt-catalyzed hydrosilylation of aryl ketones. *Org. Lett.* **18**, 4658-4661 (2016).

4. Wolinska, E. Chiral oxazoline ligands containing a 1,2,4-triazine ring and their application in the Cu-catalyzed asymmetric Henry reaction. *Tetrahedron* **69**, 7269-7278 (2013).

5. Decken, A., Gossage, R. A., Yadav, P. N. Oxazoline chemistry. Part VIII. Synthesis and characterization of a new class of pincer ligands derived from the 2-(*o*-anilinyl)-2-oxazoline skeleton - Applications to the synthesis of group X transition metal catalysts. *Can. J. Chem.* **83**, 1185-1189 (2005).

6. Hao, X. Q., Shen, M. Z., Jian, N. G., Pang, W., Shen, X. J., Zhu, X. J., Song, M. P. Synthesis of chiral *S*,*N*-thioimidazoline ligands for palladium-catalyzed asymmetric allylic allcylations.

Tetrahedron: Asymmetry **27**, 163-170 (2016).

7. Klapars, A., Antilla, J. C., Huang, X. H., Buchwald, S. L. A general and efficient copper catalyst for the amidation of aryl halides and the *N*-arylation of nitrogen heterocycles. *J. Am. Chem. Soc.* **123**, 7727-7729 (2001).

8. Nakagiri, T., Murai, M., Takai, K. Stereospecific deoxygenation of aliphatic epoxides to alkenes under Rhenium catalysis. *Org. Lett.* **17**, 3346-3349 (2015).

9. Sawada, Y., Matsumoto, K., Katsuki, T. Titanium-catalyzed asymmetric epoxidation of non-activated olefins with hydrogen peroxide. *Angew. Chem., Int. Ed.* **46**, 4559-4561 (2007).

10. Ohmiya, H., Makida, Y., Tanaka, T., Sawamura, M. Palladium-catalyzed γ-selective and stereospecific allyl-aryl coupling between allylic acetates and arylboronic acids. *J. Am. Chem. Soc.* **130**, 17276-17277 (2008).

11. Ogura, T., Usuki, T. Total synthesis of acerogenins E, G and K, and centrolobol. *Tetrahedron* **69**, 2807-2815 (2013).

12. Otto, W., Sierdzinski, J., Krol, M., Wolinska, E., Feliksbrot-Bratosiewicz, M., Wilkowojska, U. The value of tumor angiogenesis activity for stratification of HCC patients. *Int. J. Clin. Exp. Med.* **10**, 4200-4213 (2017).

13. Lu, X., Xiao, B., Zhang, Z. Q., Gong, T. J., Su, W., Yi, J., Fu, Y., Liu, L. Practical carbon-carbon bond formation from olefins through nickel-catalyzed reductive olefin hydrocarbonation. *Nat. Commun.* **7**, 11129 (2016).

14. Liu, R., Lu, Z. H., Hu, X. H., Li, J. L., Yang, X. J. Monocarboxylation and intramolecular coupling of butenylated arenes via Palladium-catalyzed C-H activation process. *Org. Lett.* **17**, 1489-1492 (2015).

15. Jefferson, A., Sargent, M. V., Wangchareontrakul, S. Synthesis and identification of ω-p henylalkylcatechols in Burmese Lac. *Aust. J. Chem.* **41**, 19-25 (1988).

16. De Esch, I. J. P., Gaffar, A., Menge, W. M. P. B., Timmerman, H. Synthesis and histamine H_3 receptor activity of 4-(*n*-alkyl)-1*H*-imidazoles and 4-(ω-phenylalkyl)-1*H*-imidazoles. *Bioorg. Med. Chem.* **7**, 3003-3009 (1999).

17. Dai, W. P., Zhang, X. X., Zhang, J., Lin, Y. Y., Cao, S. Synthesis of exocyclic trisubstituted alkenes via nickel-catalyzed Kumada-type cross-coupling reaction of gem-difluoroalkenes with di-Grignard Reagents. *Adv. Synth. Catal.* **358**, 183-187 (2016).

18. Pitteloud, J. P., Zhang, Z. T., Liang, Y., Cabrera, L., Wnuk, S. F. Fluoride-promoted cross-coupling of chloro (mono-, di-, or triphenyl) germanes with aryl halides in "moist" toluene. multiple transfer of the phenyl group from organogermane substrates and comparison of the coupling efficiencies of chloro(phenyl)germanes with their corresponding stannane and silane counterparts. *J. Org. Chem.* **75**, 8199-8212 (2010).

19. Yumino, S., Hashimoto, T., Tahara, A., Nagashima, H. Me_2S-induced highly selective reduction of aldehydes in the presence of ketones involving aldehyde-selective rate enhancement: a triruthenium cluster-catalyzed hydrosilylation. *Chem. Lett.* **43**, 1829-1831 (2014).

20. Too, P. C., Tnay, Y. L., Chiba, S. Copper-catalyzed aerobic aliphatic C-H oxygenation with hydroperoxides. *Beilstein J. Org. Chem.* **9**, 1217-1225 (2013).

21. Wagner, T., Lange, J., Grote, D., Sander, W., Schaumann, E., Adiwidjaja, G., Adam, A., Kopf, J. Organylthio(silyl)carbenes. *Eur. J. Org. Chem.*, 5198-5207 (2009).

22. Nishikawa, Y., Kitajima, M., Kogure, N., Takayama, H. A divergent approach for the total syntheses of cernuane-type and quinolizidine-type Lycopodium alkaloids. *Tetrahedron* **65**, 1608-1617 (2009).

23. Lovinger, G. J., Morken, J. P. Ni-catalyzed enantioselective conjunctive coupling with $C(sp^3)$ electrophiles: a radical-ionic mechanistic dichotomy. *J. Am. Chem. Soc.* **139**, 17293-17296 (2017).

24. Li, D., He, A. Y., Falck, J. R. Enantioselective, organocatalytic reduction of ketones using bifunctional thiourea-amine catalysts. *Org. Lett.* **12**, 1756-1759 (2010).

25. Bonet, A., Odachowski, M., Leonori, D., Essafi, S., Aggarwal, V. K. Enantiospecific sp^2-sp^3 coupling of secondary and tertiary boronic esters. *Nat. Chem.* **6**, 584-589 (2014).

26. Yonova, I. M., Johnson, A. G., Osborne, C. A., Moore, C. E., Morrissette, N. S., Jarvo, E. R. Stereospecific nickel-catalyzed cross-coupling reactions of alkyl Grignard reagents and identification of selective *anti*-breast-cancer agents. *Angew. Chem., Int. Ed.* **53**, 2422-2427 (2014).

27. Houjeiry, T. I., Poe, S. L., McQuade, D. T. Synthesis of optically active 4-substituted 2-cyclohexenones. *Org. Lett.* **14**, 4394-4397 (2012).

28. Olah, G. A., Arvanaghi, M., Ohannesian, L. Synthetic methods and reactions .126. trifluoromethanesulfonic acid triethylsilane - a new ionic hydrogenation reagent for the reduction of diaryl and alkyl aryl ketones to hydrocarbons. *Synthesis*. **9**, 770-772 (1986).
